# 6S RNA plays a role in recovery from nitrogen depletion in *Synechocystis* sp. PCC 6803

**DOI:** 10.1186/s12866-017-1137-9

**Published:** 2017-12-08

**Authors:** Beate Heilmann, Kaisa Hakkila, Jens Georg, Taina Tyystjärvi, Wolfgang R. Hess, Ilka M. Axmann, Dennis Dienst

**Affiliations:** 1 Institute for Synthetic Microbiology, Cluster of Excellence on Plant Sciences (CEPLAS), Heinrich Heine University Düsseldorf, Universitätsstrasse 1, 40225 Düsseldorf, Germany; 2 Department of Biochemistry, University of Turku, FI-20014 Turku, Finland; 3 Genetics and Experimental Bioinformatics, Faculty of Biology, University of Freiburg, Schänzlestr. 1, 79104 Freiburg, Germany; 4 Department of Biotechnology, Beuth University of Applied Sciences, Seestr. 64, 13347 Berlin, Germany; 5 Department of Chemistry - Microbial Chemistry, Ångström and Science for Life Laboratory, Uppsala University, Box 523, SE-751 20 Uppsala, Sweden

**Keywords:** Transcriptional regulation, Small RNA, σ factor, RNA polymerase, Cyanobacteria

## Abstract

**Background:**

The 6S RNA is a global transcriptional riboregulator, which is exceptionally widespread among most bacterial phyla. While its role is well-characterized in some heterotrophic bacteria, we subjected a cyanobacterial homolog to functional analysis, thereby extending the scope of 6S RNA action to the special challenges of photoautotrophic lifestyles.

**Results:**

Physiological characterization of a 6S RNA deletion strain (Δ*ssaA*) demonstrates a delay in the recovery from nitrogen starvation. Significantly decelerated phycobilisome reassembly and glycogen degradation are accompanied with reduced photosynthetic activity compared to the wild type. Transcriptome profiling further revealed that predominantly genes encoding photosystem components, ATP synthase, phycobilisomes and ribosomal proteins were negatively affected in Δ*ssaA*. In vivo pull-down studies of the RNA polymerase complex indicated that the presence of 6S RNA promotes the recruitment of the cyanobacterial housekeeping σ factor SigA, concurrently supporting dissociation of group 2 σ factors during recovery from nitrogen starvation.

**Conclusions:**

The combination of genetic, physiological and biochemical studies reveals the homologue of 6S RNA as an integral part of the cellular response of *Synechocystis* sp. PCC 6803 to changing nitrogen availability. According to these results, 6S RNA supports a rapid acclimation to changing nitrogen supply by accelerating the switch from group 2 σ factors SigB, SigC and SigE to SigA-dependent transcription. We therefore introduce the cyanobacterial 6S RNA as a novel candidate regulator of RNA polymerase sigma factor recruitment in *Synechocystis* sp. PCC 6803. Further studies on mechanistic features of the postulated interaction should shed additional light on the complexity of transcriptional regulation in cyanobacteria.

**Electronic supplementary material:**

The online version of this article (10.1186/s12866-017-1137-9) contains supplementary material, which is available to authorized users.

## Background

Cyanobacteria are photoautotrophic prokaryotes essentially relying on the availability of sunlight and CO_2_ as their major energy and carbon source, respectively. Due to their autotrophic lifestyle and versatile metabolism, cyanobacteria are suited as economical cellular chassis for diverse biotechnological applications like energy feedstock accumulation [1], third generation biofuel production [2–5] and commodity product biosynthesis [6–8]. However, the long-term mass cultivation for these applications at high cell densities also causes stress conditions that arise from redirecting metabolism, accumulation of potentially toxic compounds and the concomitant tendency for nutrient limitation (reviewed by Dexter et al. [2]). Hence, the requirements for molecular tools and well-defined targets for manipulating the cellular program towards optimized nutrient utilization are of growing interest. Within the diversity of their natural terrestrial and aquatic habitats, cyanobacteria have certainly developed extensive regulatory systems to acclimate to nutrient and light limiting conditions, which are also involving small regulatory RNA molecules (sRNAs, e.g. [9–14]).

One of the best-characterized prokaryotic sRNA regulators is the widely conserved 6S RNA that responds to changes of the nutritional status [15–18]. When cells of *Escherichia coli* (*E. coli*) enter the stationary growth phase, this highly structured RNA specifically regulates transcription by promoter mimicry, as the RNA polymerase (RNAP) holoenzyme carrying the housekeeping sigma factor σ^70^ binds to 6S RNA instead of the promoter regions of the household genes [19–22]. Upon nutrient-induced outgrowth from the stationary phase, which includes enhancement of NTP levels, 6S RNA acts as a template for the de novo synthesis of a ~20 nt product RNA (pRNA) [23, 24]. pRNA synthesis triggers the release of 6S RNA from RNAP reverting 6S RNA-dependent inhibition [23, 25]. 6S RNA lacking mutants of *E. coli* and *Bacillus subtilis* (*B. subtilis*) show significant phenotypes under long-term nutrient deprivation [20], stress conditions, like alkaline stress [26, 27] and during outgrowth from stationary phase [28, 29]. Furthermore, the presence of 6S RNA leads to an upregulation of σ^38^-activity, while overexpression of 6S RNA in σ^38^-deficient cells results in reduced viability in late stationary phase [19].

Homologues of 6S RNA have been identified in several freshwater cyanobacteria, including the model organism *Synechocystis* sp. PCC 6803 (*Synechocystis* 6803) [15, 30, 31]. While in vitro studies indicate a conservation of basic 6S RNA mechanisms like transcription inhibition and pRNA synthesis, little is known about the functional relevance of the cyanobacterial 6S RNA in vivo [32].

Since phototrophic growth of cyanobacteria does essentially rely on atmospheric or enriched CO_2_ supplementation and – importantly – light, plain dilution experiments would rather evoke responses to changing light availability (and its relation to CO_2_ availability). Accordingly, a specified experimental setup was required for functional characterization of cyanobacterial 6S RNA during outgrowth from nutritional deprivation. Regarding the status of an inorganic nitrogen source – i.e. nitrate or ammonia – non-diazotrophic cyanobacteria like *Synechocystis* 6803 exhibit a well-controlled acclimation behavior that is also accompanied by a stationary growth plateau [33]. Acclimation of cyanobacteria to changing nitrogen availability is a tightly controlled process that has been well described in the literature. One of the most pronounced physiological responses is the remodeling of the photosynthetic machinery, leading to distinctly and visibly reduced amounts of the phycobilisome antenna complex and – delayed in time – chlorophyll *a* breakdown [33–36]. These biochemical mechanisms are influenced by elaborate underlying events of gene expression control. This network essentially involves the collaborative action of the global transcriptional regulator NtcA and the proteins PII and PipX [37].

Moreover, several group 2 σ factors appear to play a role within the nitrogen regulatory network. Besides the nitrogen stress response σ factor SigE, these are SigB, involved in the NtcA-dependent nitrogen-related gene expression during exponential growth and SigC, in the response of stationary phase cultures to nitrogen starvation [38–40].

Several reports described the transcriptomic characteristics of nitrogen-starving cyanobacteria [41, 42] as well as the response upon nitrogen repletion in a time-resolved manner [36, 43]. The recovery process after long-term starvation is initiated by the upshift of respiratory gene expression and switching on the translational machinery as well as nitrogen assimilation. Further, until full physiological restoration, the cells successively reactivate photosynthetic complexes, carboxysomes and the cell division machinery [43]. Cells without any group 2 σ factors are not able to recover from nitrogen deficiency [40].

Here we describe the involvement of the sRNA regulator 6S RNA in the dynamics of nitrogen acclimation in *Synechocystis* 6803, elaborating the example of NO_3_^−^-mediated recovery from long-term nitrogen starvation.

## Results

### Inactivation of 6S RNA affects photosynthetic parameters and storage of carbohydrates upon nitrogen starvation and recovery

**Fig. 1:**
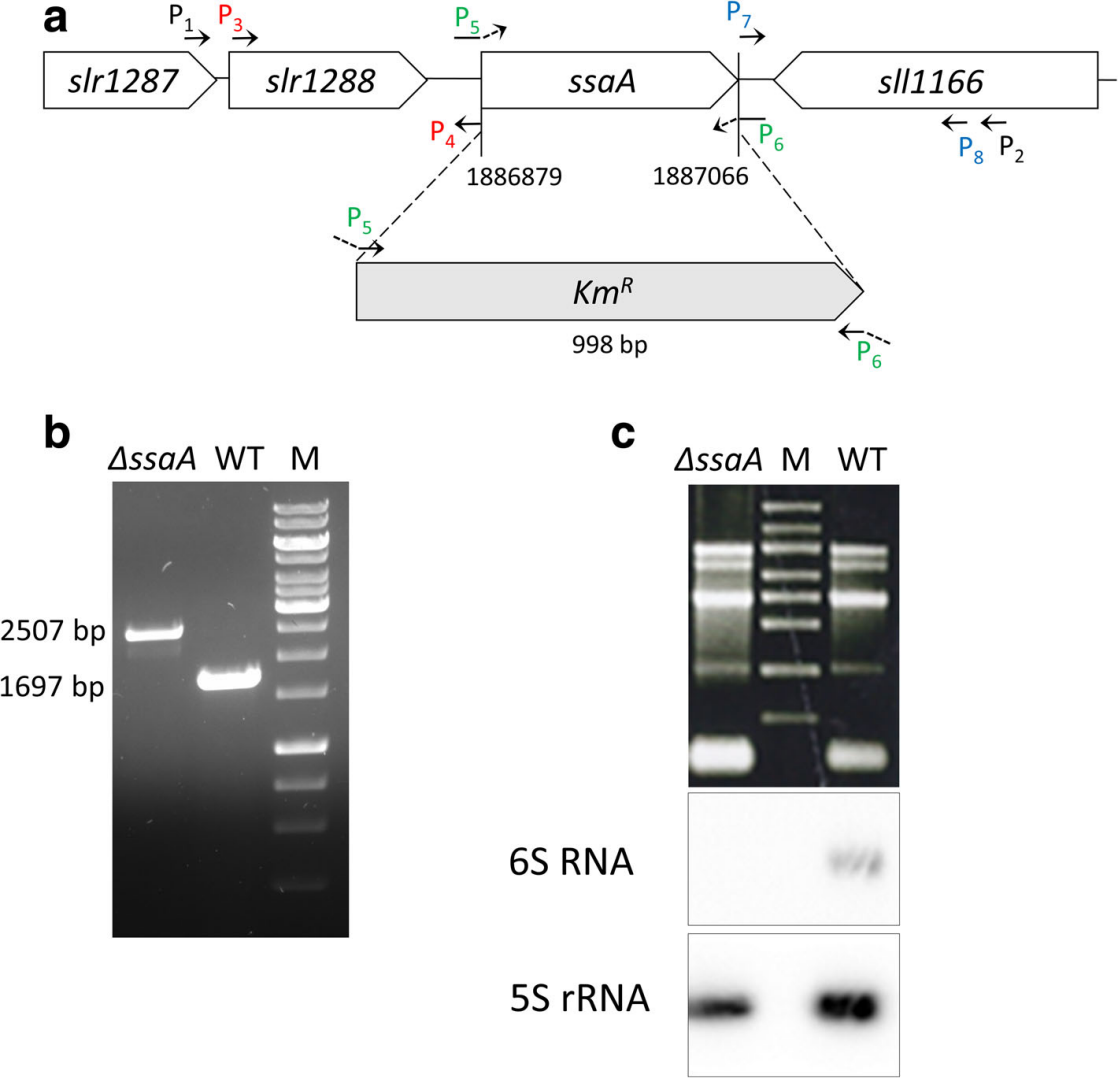
Strategy and confirmation of the *Synechocystis* 6803 Δ*ssaA* mutant. **a** Strategy for the construction of the Δ*ssaA* mutant. The *ssaA* gene was replaced by a kanamycin resistance cassette (Km^R^) and its promoter region (grey box) at the indicated genome position (*ssaA* locus: 1,886,879…1,887,066). The construction was realized by overlap extension PCR of three fragments using primer pairs P3 and P4 (red), P5 and 6 (green, P5: 15 bp 5′-end overlap with P4, 17 bp 3′-end overlap with Km^R^; P6: 14 bp 5’end overlap with P7, 24 bp 3’end overlap with Km^R^) and P7 and P8 (blue). The construct was further used to replace *ssaA* in the genome of *Synechocystis* 6803 by homologous recombination. P1 and P2 represent the location of primers used to verify complete segregation of the mutant strain. The location of genes *slr1287, slr1288* and *sll1166* are depicted upstream and downstream of the *ssaA* gene. **b** Complete segregation of the Δ*ssaA* mutant gene copies and replacement with a kanamycin resistance cassette (Km^R^) and its flanking regions (998 nt) was tested by PCR analysis, using primers P1 and P2. **c** Deletion of 6S RNA in Δ*ssaA* mutant was confirmed by Northern blot analysis using a radioactive [^32^P]-labelled oligonucleotide for 6S RNA. 5S rRNA was hybridized as a loading control

To analyze the functional role of 6S RNA in cyanobacteria, we deleted the *ssaA* gene encoding 6S RNA from the genome of *Synechocystis* 6803. The *ssaA* gene was replaced with a kanamycin resistance cassette (Fig. 1a) and complete segregation of the resulting Δ*ssaA* mutant was confirmed by colony PCR (Fig. 1b) as well as by Northern Blot analysis (Fig. 1c).

Under standard growth conditions, the ∆*ssaA* strain did not show any clear phenotype. Referring to 6S RNA function in *E. coli* and *B. subtilis*, where it regulates RNAP activity during the stationary growth phase and outgrowth [17, 18], the following mutant characterization experiments were conducted under nitrogen depletion (which leads to a stationary state of cell culture) and subsequent recovery by repletion with 17.6 mM NaNO_3_ (corresponding to outgrowth).

**Fig. 2:**
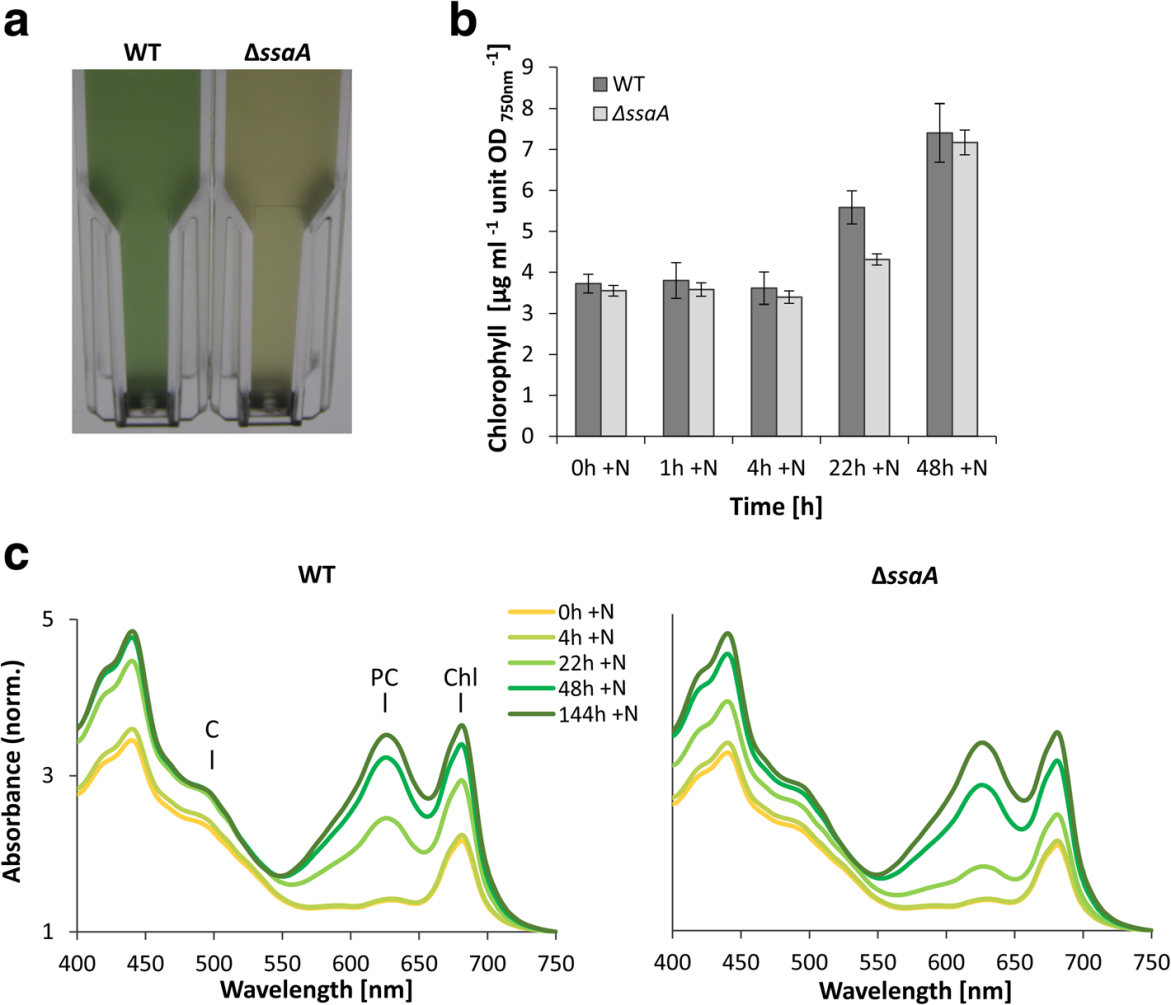
Comparative physiological characterization of the *Synechocystis* 6803 WT and Δ*ssaA* mutant strain and phenotype studies of the mutant strain. Cultures were grown under continuous white light at an irradiance of 80 μmol photons m^−2^ s^−1^, at 30 °C, in nitrogen-depleted medium for 189 h before recovery was initiated by adding nitrogen. **a** Photograph of cultures of *Synechocystis* 6803WT and Δ*ssaA* mutant strain during recovery from nitrogen starvation, *t* = 22 h + N. Cultures were adjusted so that their cell densities were the same (as estimated by the OD_750_), transferred to 1-cm cuvettes, and photographed with illumination from behind. **b** Chlorophyll content of *Synechocystis* 6803 WT and Δ*ssaA* measured after cultivation in nitrogen deficiency for 189 h (*t* = 0 h + N) and at recovery time points *t* = 1 h, *t* = 4 h, t = 22 h and *t* = 48 h (+N). The illustrated data represent the mean from three independent biological replicates and the standard deviation was calculated. **c** Details of whole cell absorbance spectra of *Synechocystis* 6803 WT and Δ*ssaA* strains are shown for the time point t = 0 h + N, which corresponds to 189 h under nitrogen deficiency (−N) and for the time points t = 4, t = 22, t = 48 and *t* = 144 h after nitrogen addition (+N). The spectra were normalized at 750 nm. C: carotenoids; PC: phycocyanine; Chl: chlorophyll *a*

The acclimation to nitrogen depletion proceeded similarly in the Δ*ssaA* strain as in wild type (WT), demonstrated by similar whole-cell absorption spectra of Δ*ssaA* and WT cells after seven days of nitrogen deficiency (Fig. 2c, 0 h + N samples; Additional file 1: Figure S1a). Furthermore, light-saturated photosynthetic activities (Additional file 1: Figure S2a) and 77 K fluorescence spectra (Additional file 1: Figure S2b) were similar in both strains in the middle of the nitrogen deficiency treatment.

Visible differences between the strains were detected during the recovery phase. After 22 h of recovery, WT showed normal green color of a viable *Synechocystis* 6803 culture while the Δ*ssaA* mutant still remained yellow-orange, which is a typical characteristic of nitrogen depleted cultures (Fig. 2a).

Whole-cell absorption spectra were measured to follow changes in all major photosynthetic pigments. The color of nitrogen depleted cells is due to prominent loss of blue phycobilins (absorption maxima at 620 - 635 nm), clear loss of green chlorophyll *a* (Chl *a*, absorption maxima at 680 nm)*,* but only minor reduction of yellow-orange carotenoid pigments (Fig. 2c; compare 0 h + N and the fully recovered 144 h + N samples). Re-synthesis of pigments takes hours after nitrogen supply. In both strains phycobilin (Fig. 2c) and Chl *a* (Fig. 2b and c) contents remained low after 4 h of recovery. After 22 h both phycobilins and Chl *a* showed significant retrieval in WT while in ∆*ssaA,* the recovery process was still in an early stage (see Additional file 1: Figure S1 for pairwise comparison of WT and Δ*ssaA* at each time point). According to statistical analysis (one-way ANOVA), the Chl *a* content was significantly lower in Δ*ssaA* than in WT at 22 h + N (*p =* 0.0067). A delay in phycobilisome reconstruction in ∆*ssaA* was also seen as reduced accumulation of the main phycobiliproteins, phycocyanin and allophycocyanin, after 22 h of nitrogen repletion (Additional file 1: Figure S4a and b). The phycocyanin content was significantly lower in Δ*ssaA* than in WT at 22 h + N (*p* = 0.0183, one-way ANOVA; Additional file 1: Figure S4a). However, Δ*ssaA* cells completely recovered over extended periods of 48 h or longer (Fig. 2b and c). A Δ*ssaA* complementation strain (∆*ssaA*-c), which expresses the native *ssaA* gene from a self-replicative vector, recovered from the nitrogen deficiency similarly as WT (Additional file 1: Figure S3), confirming that this phenotype is a specific result from the absence of 6S RNA, rather than from polar effects.

**Fig. 3:**
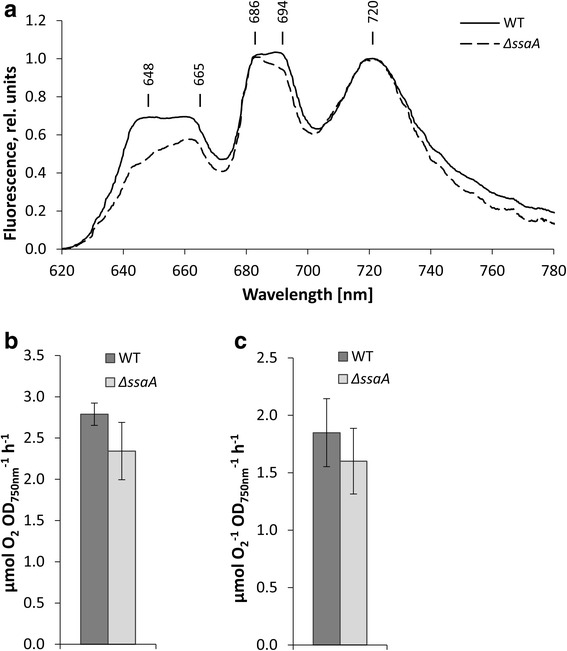
Photosynthetic parameters of WT and ∆*ssaA* mutant during recovery from nitrogen starvation. **a** Orange light-excited fluorescence emission spectra of WT and ∆*ssaA* mutant at 77 K after 14 h of recovery (+N). The spectra were normalized by dividing by the peak value of PSI at 721 nm and setting this value to 1. Oxygen evolution rates of electron transport in PSII (**b**) and photosynthesis (**c**) measured in vivo at time point 16 - 22 h + N under light-saturated conditions (3000 μmol photons m^−2^ s^−1^). Three independent biological replicates were measured and the error bars represent the standard deviation

To further compare the recovery of the photosynthetic reaction centres and light harvesting complexes of the strains, we measured 77 K fluorescence spectra using orange light phycobilisome excitation 14 h after nitrogen addition. This time point was selected because in the experimental setup the chlorotic lag of Δ*ssaA* was apparent particularly during the period between 12 h and 24 h after addition of nitrate. The lower peaks originating from phycobilisome rods (648 nm) and cores (665 nm) in Δ*ssaA* cells indicate lower amounts of both phycocyanin and allophycocyanin in the mutant strain, thus confirming the decelerated recovery of phycobilisome antenna in the mutant (Fig. 3a). In addition, the ratio of CP47 peak (originates from the inner antenna protein CP47 of PSII) at ~ 694 nm to CP43 peak (originates from the terminal emitter of the phycobilisome antenna and from the PSII inner antenna protein CP43) at ~ 658-686 nm was lower in the Δ*ssaA* mutant than in WT, indicating reduced efficiency of light harvesting for PSII in Δ*ssaA* (Fig. 3a). In accordance with that the light-saturated PSII and photosynthetic activities were 10 to 15% lower in Δ*ssaA* than in WT after 22 h of recovery in nitrogen replete conditions (Fig. 3b and c). However, due to high variations between biological replicates these differences between the strains are not statistically significant.

Since nitrogen starvation limits consumption of carbon skeletons, starved *Synechocystis* 6803 cells store carbon polymers like glycogen. Under nitrogen starvation both strains stored similar amounts of glycogen (Additional file 1: Figure S5). After nitrogen addition glycogen decreased in both strains, where a delayed degradation in Δ*ssaA* is indicated by comparatively high levels after 22 h (Additional file 1: Figure S5). Our results indicate that although 6S RNA is not completely essential for the cells to recover from nitrogen starvation, the presence of 6S RNA accelerates the recovery process for hours.

**Fig. 4:**
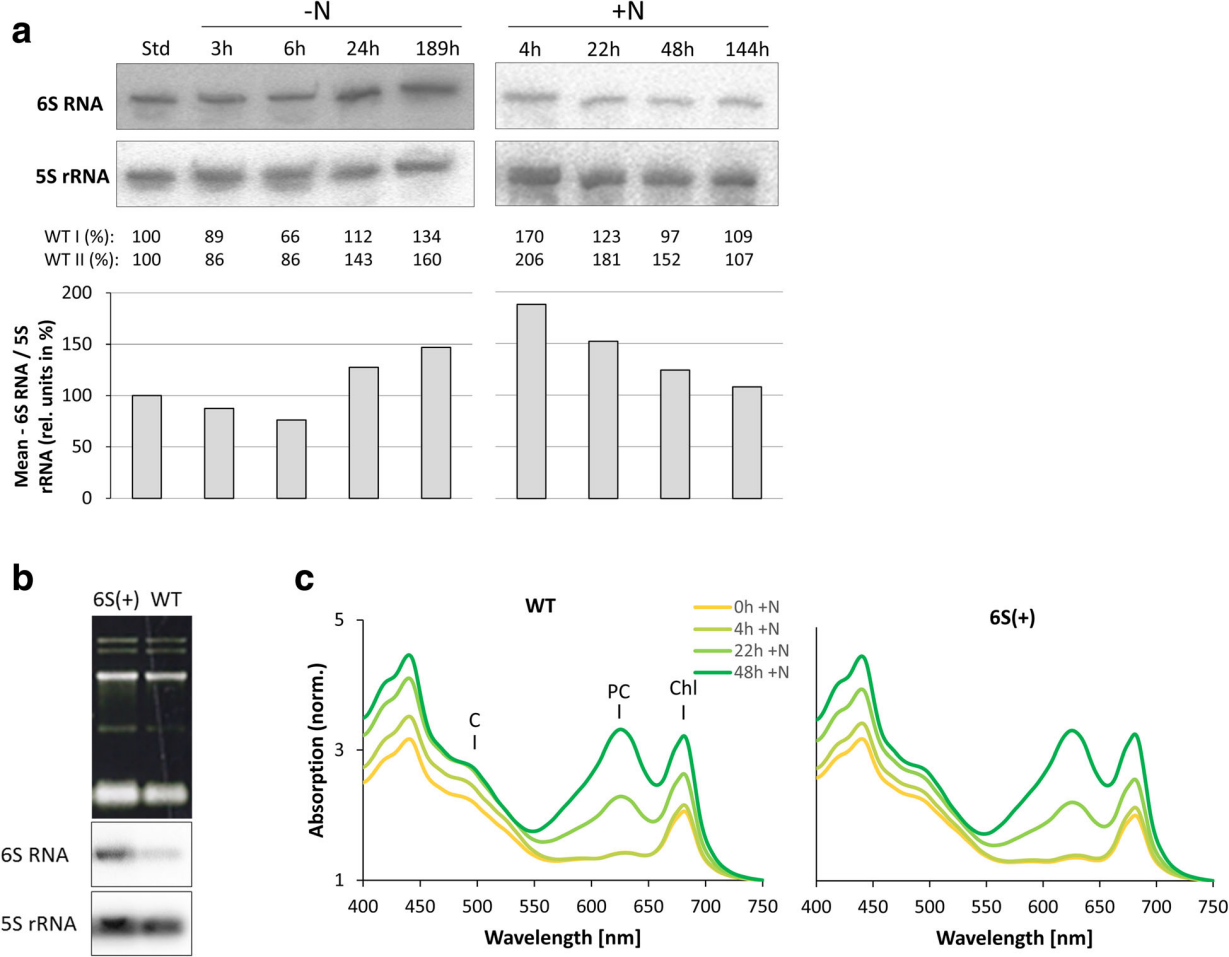
Analysis of 6S RNA transcript levels during nitrogen starvation and recovery in *Synechocystis* 6803 WT (**a**) and physiological characterization of the *Synechocystis* 6803 mutant strain overexpressing 6S RNA (6S(+)) (**b** and **c**). **a** Total RNA was isolated from cell cultures grown at standard growth conditions (t = Std), under nitrogen depleted conditions (*t* = 3 h -N, *t* = 6 h -N, *t* = 24 h -N, *t* = 189 h -N) and under nitrogen replete conditions (t = 4 h + N, t = 22 h + N, t = 48 h + N, t = 144 h + N) and samples with 5 μg RNA were analyzed by Northern blot hybridization. Autoradiograms are illustrated for WT I. 6S RNA and 5S rRNA signals of two biological replicates (WT I, WT II) were quantitated and 5S rRNA signal was used as an internal reference. The mean values are illustrated in the chart. Values of t = Std were set to 100 and samples of all other time points were calculated, respectively. **b** Northern Blot analysis to confirm overexpression of 6S RNA in 6S(+) mutant. 2 μg of total RNA was separated by electrophoresis on 1.3% agarose-formaldehyde gel. The amount of 6S RNA and 5S rRNA was detected by using radioactive [^32^P]-labeled oligonucleotides. **c** Physiological characterization of the 6S(+) strain. Absorbance spectra of the *Synechocystis* 6803 wild-type and the 6S(+) strain are illustrated for the time points t = 0 h + N, t = 4 h + N, t = 22 h + N and t = 48 h + N. The spectra were normalized at 750 nm. C: carotenoids; PC: phycocyanine; Chl: chlorophyll *a*

Further investigation was carried out to analyze the 6S RNA transcript level under nitrogen starvation and recovery. Northern Blot analysis of 6S RNA revealed that the accumulation of 6S RNA increased in WT in the course of long term (189 h -N) nitrogen depletion by ~40 - 55% and returned to the original level, when normalized to 5S rRNA levels (Fig. 4a). However, since ribosomal RNA is rather expected to decrease during chlorosis [44] and consequently to increase again during recovery, 6S RNA accumulation is rather constant or slightly upregulated (Fig. 4a).

Interestingly, overexpression of 6S RNA, as implemented in the 6S(+)-mutant, did not further accelerate recovery after nitrogen depletion (Fig. 4b and c).

### 6S RNA accelerates expression of household genes upon recovery from nitrogen starvation

Comparative transcriptome analysis was performed to infer a functional signature from the mutant-specific transcript profiles (raw data is available in Additional files 2 and 3). For this purpose, microarray analysis was used to probe total RNA from WT and ∆*ssaA* cultures that were starved for nitrogen for seven days and recovered over a period of 22 h. Sampling time points were t_1_ = 0 h (7d -N; reference), t_2_ = 1 h + N, t_3_ = 4 h + N and t_4_ = 22 h + N. At t_1_ = 0 h a total set of 65 features showed differential accumulation (log2 fold change (FC) of >1 (adjusted *p*-value of ≤0.05) in Δ*ssaA*, the majority of which were downregulated. Most of the latter comprised non-coding transcripts like asRNAs, but also eight mRNAs showed lower levels. Among them were several mRNAs encoding subunits of ATP synthase, the mRNA for cell division protein SepF as well as ferredoxin encoding *petF* and the high-light inducible *hliA*. The positively regulated genes included the heat stress responsive histidine kinase *hik34* and the chaperone encoding genes *dnaJ* and *groEL-2* (Additional file 1: Figure S6).

Temporally altered transcript levels in Δ*ssaA* from t_1_ = 0 h to all three analyzed time points of recovery were plotted as a function of the corresponding temporal changes in WT, distinctively comparing the recovery in both strains (Additional file 1: Figures S7-S9). Comparison of transcript profiles indicated that only few genes were activated or repressed in the early phase of recovery, whereas 22 h after addition of nitrogen, numerous genes responded, most of which were upregulated (Additional file 1: Figure S9).

**Fig. 5:**
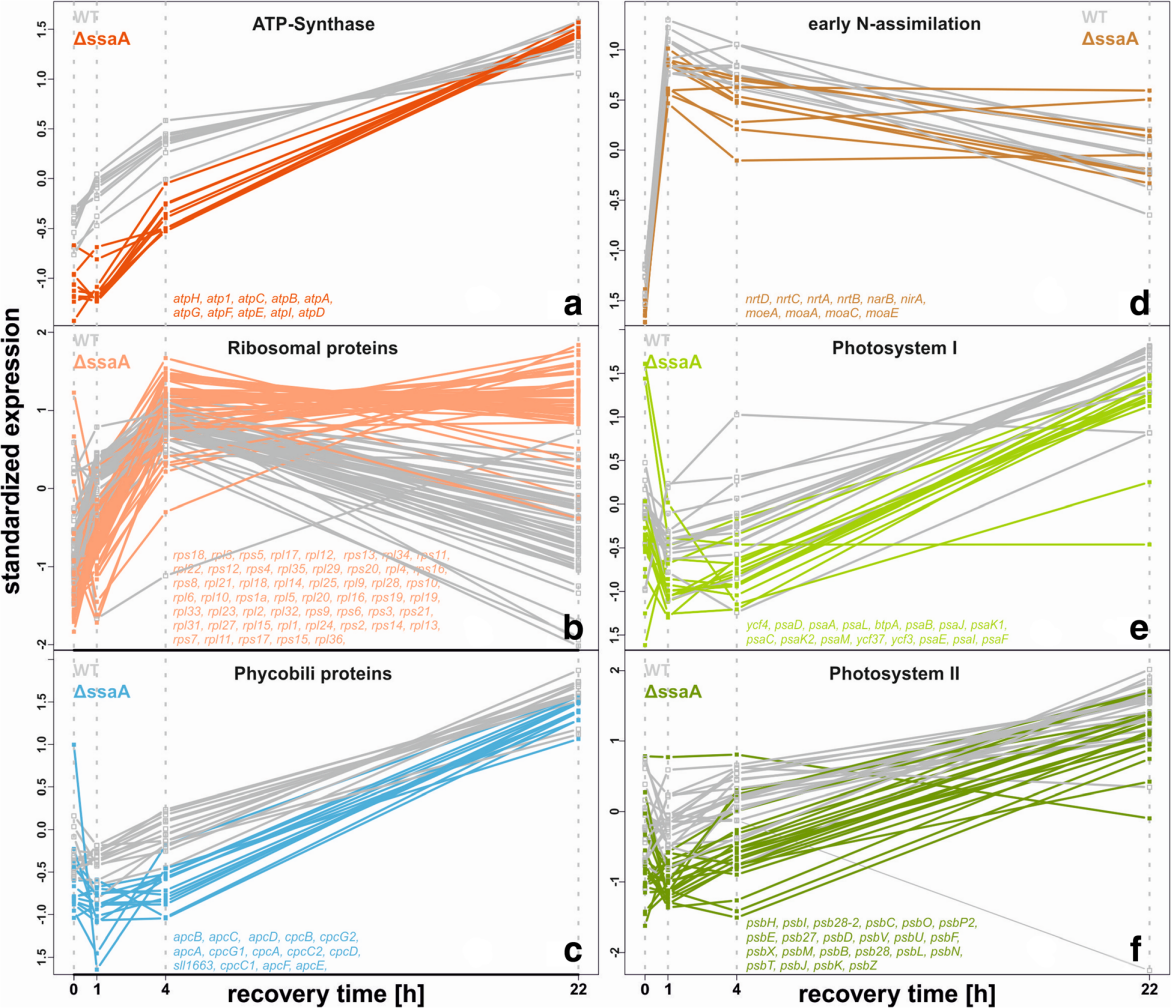
Temporal response of functional groups to nitrogen re-addition in the WT and Δ*ssaA*. Transcriptional profile of genes related to ATP synthase (**a**), ribosomal proteins (**b**), phycobili proteins (**c**), early nitrogen assimilation (**d**), PSI (**e**) and PSII (**f**) are grouped respectively. (x-axis) time after nitrogen addition. (y-axis) standardized normalized gene expression. To be able to compare the dynamics of the different genes in one plot the expression levels were standardized gene wise for both strains together, so that the average expression value is 0 and the standard deviation is one. WT expression is shown in grey and colored for Δ*ssaA*. The color code refers to the displayed functional categories (red: ATP-synthase, salmon: Ribosomal proteins, cyan: Phycobili- proteins, brown: N-assimilation, light green: Photosystem I, dark green: Photosystem II). For comparability the same color code is used in (Figs. S7-9). The genes which are plotted for each group are indicated in each sub-figure

Normalized expression values of selected groups of functionally related genes have been standardized to be able to compare the expression patterns changes of genes with different expression levels and are further depicted in time courses in Fig. 5a-f. In WT cells transcriptional upshift of ATP synthase genes was seen already 1 h after nitrogen addition and continued for whole recovery phase (Fig. 5a). In Δ*ssaA,* upregulation of *atp* transcripts was delayed, keeping *atp* transcripts at low levels for the first 4 h. However, these differences between both strains vanished after 22 h of recovery (Fig. 5a). Likewise, the transcriptional upshift of ribosomal subunits was delayed in Δ*ssaA* (Fig. 5b).

During the first 4 h after nitrogen repletion many genes encoding proteins for the major photosynthetic complexes photosystem I (*psa* genes*,* Fig. 5e), photosystem II (*psb* genes, Fig. 5f) and light harvesting phycobilisome antenna genes (Fig. 5c) produced less transcripts in Δ*ssaA* than in WT. After 22 h of recovery these effects were not that clear anymore. For genomic context of *apc* and *cpc* operons*,* see Additional file 1: Figure S10. Altogether, the transcriptomics data is in good agreement with the delayed physiological response of the mutant as reflected by the data shown in Figs. 2 and 3.

Notably, levels of important ‘marker’ transcripts of nitrogen recovery encoding components of the nitrate uptake (*nrtABCD*) and early assimilation machinery (*narB*, *nirA*, *moaACE*) increased rapidly in both strains (Fig. 5d). Over the complete period, abundance of e.g. nitrate reductase encoding *nirA* kept stable in the mutant, whereas a moderate decline after the first hour was observed for all transcripts in the WT and likewise – for the majority of this group – in Δ*ssaA* (Fig. 5d).

**Fig. 6:**
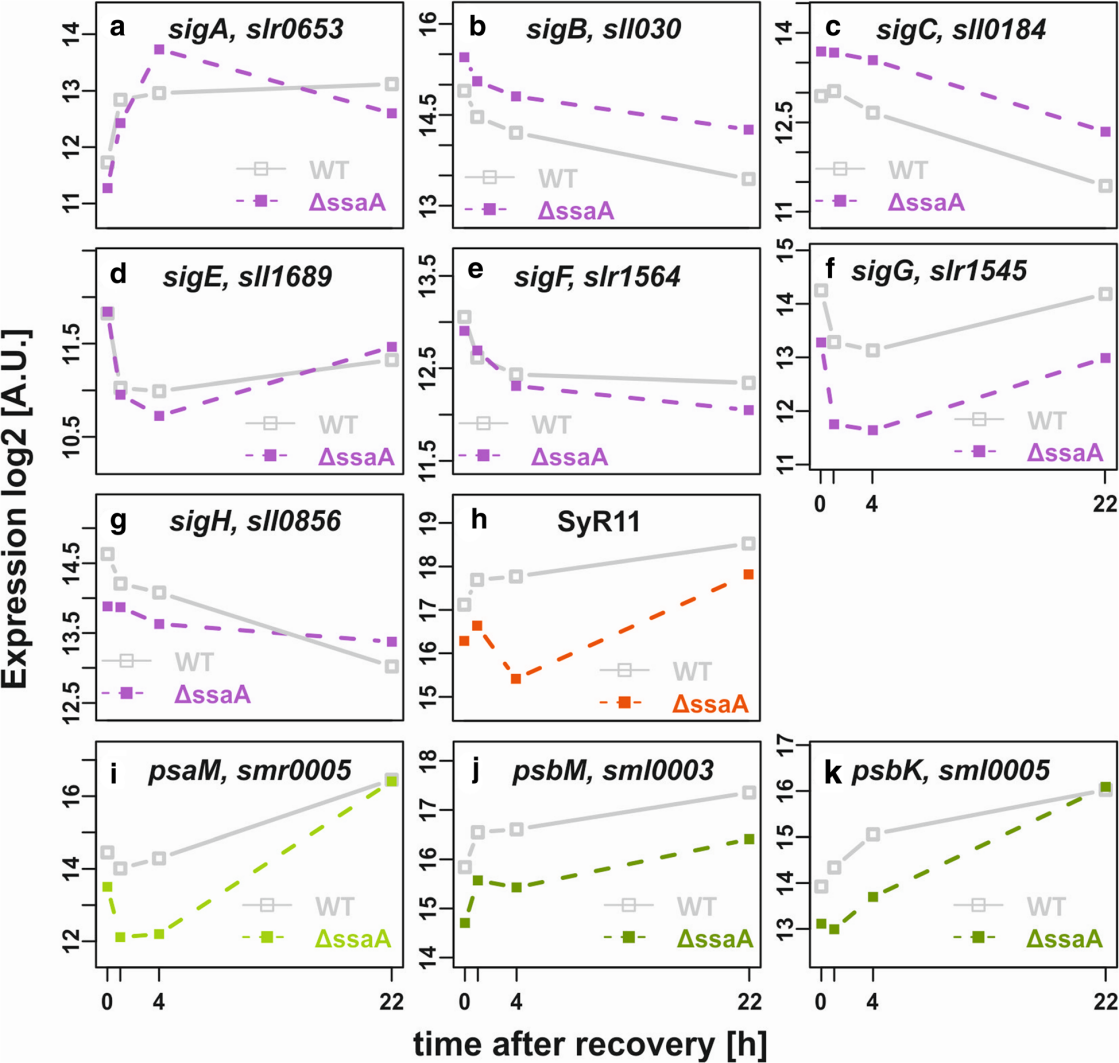
Temporal response of selected genes to nitrogen re-addition. Transcriptional profile of σ factors including group 1 σ factor SigA (**a**), group 2 σ factors SigB (**b**), SigC (**c**) and SigE (**d**) and group 3 σ factors SigF (**e**), SigG (**f**) and SigH (**g**) as well as of sRNA SyR11 (**h**). Representative mRNAs encoding photosystem genes (*psaM* (**i**); *psbK* (**j**) and *psbM* (**k**)) are shown to resolve the global effects illustrated in Fig. 5 exemplarily. (x-axis) time after nitrogen addition. (y-axis) normalized log2 expression. WT expression is shown in grey and Δ*ssaA* expression is color coded regarding to the color code in the scatter plots (Figs. S7-9)

For possible transcriptional regulators, we focused on genes encoding the regulatory σ factors. The *sigA* transcript (encoding primary σ factor SigA) was upregulated in Δ*ssaA* compared to WT after 4 h of recovery, while transcripts for group 2 σ factors SigB and SigC were slightly up at all time points in the mutant. Levels of *sigG*, encoding an ECF-type σ factor, were slightly down (Fig. 6a-g). Note that the mRNA accumulation of σ factor encoding genes cannot be considered a measure of σ factor recruitment by RNAP, but rather an effect of the latter.

In addition to protein-coding genes, a few small non-coding RNAs (sRNAs) showed altered levels in the mutant, including the previously characterized PmgR [45], NsiR4 and PsrR1 [11], as well as several hypothetical riboregulators that were identified by differential RNA sequencing (dRNA-seq) [46]. Among these transcripts, SyR11 exhibited the most diverging accumulation kinetics (Fig. 6h; see also Northern Blot validation in Additional file 1: Figure S11). Additionally, Fig. 6i-k illustrate the mRNA abundances of three exemplarily selected transcripts (*psaM, psbK, psbM*) of PSI and PSII, revealing the decelerated upregulation of components of the photosynthetic apparatus in Δ*ssaA* in detail.

### 6S RNA influences the allocation of SigA, SigB, SigC and SigE in the RNA polymerase holoenzyme

We next studied if the absence of 6S RNA influences the content of the primary or group 2 σ factors and/or recruitment of different σ factors by the RNAP core to form the holoenzyme during nitrogen starvation and recovery phase. To that end, the γ-subunit of RNAP core was replaced with His-tagged γ-subunit in WT and Δ*ssaA* as described recently for a non-motile *Synechocystis* 6803 strain [47]. The WT RNAP-His and Δ*ssaA* RNAP-His strains were grown under standard conditions or subjected to nitrogen depletion for 6 days; recovery samples were taken 1 h, 4 h or 22 h after nitrogen addition. The His-tagged RNAP complexes were collected from one-half of each sample and the soluble proteins were isolated from the other half.

**Fig. 7:**
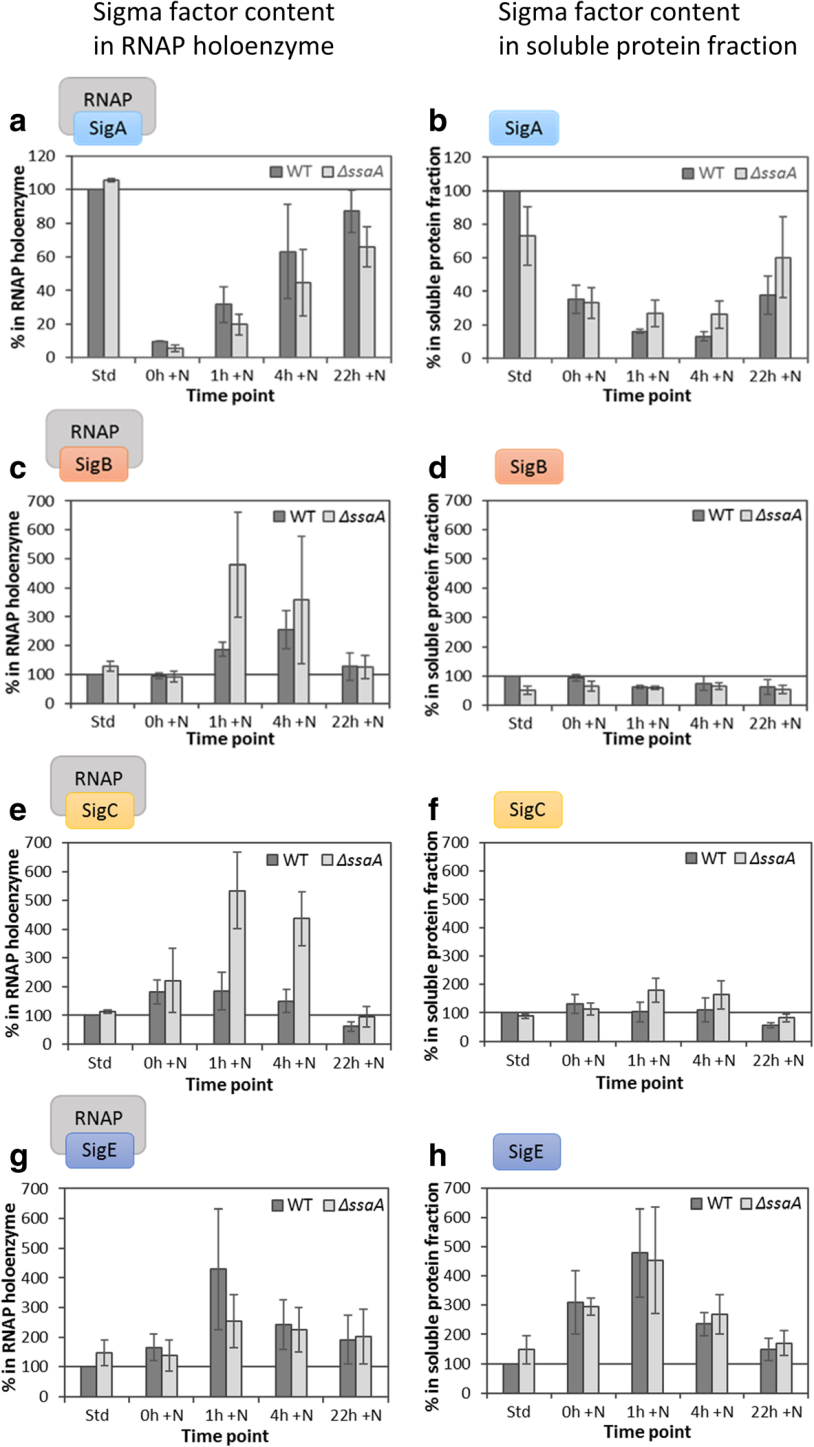
Relative content of different sigma factors (σ) in RNAP holoenzyme (left panel; **a**, **c**, **e**, **g**) and in the soluble protein fraction (right panel; **b**, **d**, **f**, **h**) of WT-RNAP-HIS (WT) and Δ*ssaA*-RNAP-HIS mutant (Δ*ssaA*) strain under standard conditions (Std), under nitrogen deficiency for 6 days (t = 0 h + N) and during recovery (t = 1 h + N, t = 4 h + N and t = 22 h + N). Equal amounts of collected RNAP complexes or soluble proteins were separated on SDS-PAGE, group 1 σ factor SigA (**a**, **b**) and group 2 σ factors SigB (**c**, **d**), SigC (**e**, **f**) and SigE (**g**, **h**) were detected by immunoblot analysis. The σ factor content was normalized to α or β RNAP core subunit. For each σ factor the value of the WT-RNAP-HIS strain at standard conditions (Std) was set to 100 and the other samples were compared respectively. Three independent biological replicates were analyzed and the error bars represent the standard error

To measure possible changes in SigA content and/or in recruitment efficiencies by the RNAP core, isolated soluble proteins and collected RNAP complexes were separate on SDS-PAGE, respectively. Subsequently, the SigA content was detected using a SigA specific antibody [47]. Then membranes were re-probed with the antibody against the α (or β) subunit of the RNAP core and the SigA content was normalized to the RNAP core content. Finally, after each treatment, the amount of SigA was compared to the amount of SigA in cultures from standard (nitrogen replete) conditions. SigA almost vanished from the RNAP holoenzyme during nitrogen depletion in both strains (Fig. 7a), but SigA protein content decreased by only 65% (Fig. 7b) indicating that the recruitment of SigA by RNAP core reduced more drastically than SigA content. After nitrogen addition, RNAP core progressively recruited more SigA (Fig. 7a). The recovery of the SigA recruitment occurred more slowly in Δ*ssaA* than in WT (Fig. 7a), although the SigA content increased more rapidly during recovery in Δ*ssaA* than in WT (Fig. 7b). Poor recruitment of SigA in Δ*ssaA* is an obvious reason for slow activation of household genes in this strain.

When group 2 σ factors were analyzed, using the same method as described for the primary σ factor, the results showed that in nitrogen starved WT cells (0 h + N) both recruited and total SigB remained at the same level as under standard conditions (Fig. 7c and d). Although the total amount of SigB decreased moderately during the recovery period, this σ factor was recruited in particular during the early recovery phase (Fig. 7c and d). In Δ*ssaA,* SigB was more efficiently recruited by RNAP core both under standard conditions and during early recovery than in WT (Fig. 7c and d). Thus, the presence of 6S RNA seems to restrict recruitment of SigB by the RNAP core. Like SigB, SigC was highly recruited during recovery phase, however SigC was already recruited efficiently in nitrogen depleted cells (Fig. 7e and f). Very high levels of SigC recruitment were observed in the mutant strain at early time points of recovery. The amount of SigD was very low or below detection limit in all samples and quantification of SigD results was not possible. Unlike the other σ factors, the SigE protein content was clearly higher in nitrogen depleted samples, which was also observed in samples from the early recovery phase. Increased SigE content resulted in enhanced recruitment of SigE during nitrogen recovery, especially in early phase (Fig. 7g and h).

## Discussion

### 6S RNA promotes the recovery process from N starvation

The acclimation processes of cyanobacteria to changing nitrogen availability are underlain by a complex regulatory network, involving signal-driven transcriptional regulons and differential σ factor activity, refined with a layer of sRNA-mediated post-transcriptional regulation [48–50].

Here we demonstrate that the cyanobacterial 6S RNA homologue plays a role within the transcriptional response during recovery from nitrogen depletion. The Δ*ssaA* mutant of *Synechocystis* 6803 showed a more sustained bleaching phenotype linked with a generally decelerated transcriptional and physiological response during recovery. Thus, within this gradual acclimation process 6S RNA containing cells have a substantial selective advantage over cells missing 6S RNA. On the other hand, overproduction of 6S RNA in *Synechocystis* 6803 did not accelerate recovery from nitrogen starvation, suggesting that this riboregulator is not a limiting factor in the native acclimation system.

The behavior of 6S RNA during nitrogen-deficiency-induced bleaching and subsequent recovery in *Synechocystis* 6803 differs from that reported for stationary phase in heterotrophic bacteria. In *Synechocystis* 6803, 6S RNA increases only slightly during nitrogen starvation (Fig. 4a) and 6S RNA is quite abundant under standard conditions [46, 51]. For the cyanobacterium *Synechococcus* sp. PCC 6301 it has been reported previously, that 6S RNA levels decrease during stationary phase [31]. In *E. coli,* 6S RNA molecules enrich 10-fold from early exponential growth phase to late stationary phase [19] and binding of RNAP-σ^70^ holoenzyme to 6S RNA keeps the RNAP-σ^70^ holoenzyme complex abundant although it becomes inactive. The importance of the regulatory function of 6S RNA in *E. coli* is obvious as deletion of 6S RNA affects expression of ~ 800 genes in stationary phase. Contrary to that, 6S RNA might play only a minor role in nitrogen starved *Synechocystis* 6803 cells as only very few genes show different expression in nitrogen starved WT and Δ*ssaA* cells (Additional file 1: Figure S6). Furthermore, the amount of the RNAP-SigA complex drastically reduces during nitrogen starvation in *Synechocystis* 6803 indicating that formation of inactive RNAP-SigA-6S RNA holoenzyme complexes is not the main regulatory mechanism behind reduced transcription of household genes. It is possible that 6S RNA exhibits some functional diversity within different bacterial phyla. Interestingly, *Bacillus subtilis* produces two different 6S RNAs, 6S-1 RNA and 6S-2 RNA [52]. The 6S-1 RNA (*bsrA*) is highly upregulated in stationary phase just like 6S RNA in *E. coli* while 6S-2 RNA (*bsrB*) transcript level decrease towards stationary phase [53]. Therefore, *Synechocystis* 6803 6S RNA possibly resembles 6S-2 RNA in matters of transcriptional regulation.

In accordance with the physiological data, transcriptome profiling demonstrated that particularly genes encoding ATP synthase, PSI and PSII and phycobilisomes as well as the translational machinery were overall negatively affected by the absence of 6S RNA during recovery from nitrogen starvation. The resuscitation process from severe long-term nitrogen starvation has recently been described in detail for *Synechocystis* 6803 by Klotz and co-workers [43]. The recovery process from nitrogen starvation can be divided into two phases. Phase 1 is characterized by regeneration of basic cellular functions, including respiration, translation and nitrogen assimilation, which could be clearly confirmed by our transcriptome study. Here, certainly expression of ATP synthase and ribosomal genes showed a delay of general upshift from 1 h to 4 h after nitrogen addition in the Δ*ssaA* mutant. The kinetics of photosynthesis-related mRNA accumulation further emphasizes the generally delayed recovery: the early phase 2 response, which is partially initiated after 4 h in WT, rather arises later in the mutant, largely approaching WT levels after 22 h. Moreover, due to delayed transcriptional activation of phase 1 and phase 2 genes, recovery of physiological processes like phycobilisome reassembly, photosynthetic activity and glycogen degradation were delayed in Δ*ssaA*.

Our results show that 6S RNA has an effect on the recruitment of σ factors especially during early recovery from nitrogen depletion but does not affect expression of NtcA controlled genes like *amt1*, *glnA*, *glnB* or *sigE*, indicating an additional regulatory circuit that acts independently and putatively supplementary to the well-characterized PipX-PII-NtcA network [44]. Although none of the group 2 σ factors alone is essential for acclimation to nitrogen deficiency and subsequent recovery, cells without functional group 2 σ factors were more sensitive to nitrogen depletion [40].

During recovery from nitrogen depletion WT cells recruited SigA more efficiently than Δ*ssaA* cells, indicating that SigA recruitment was promoted by 6S RNA when cells switched from nitrogen depletion to repletion.

The amount of RNAP-SigC increased in both strains during nitrogen deficiency and in the beginning of recovery phase. However, the SigC recruitment during the early recovery phase was considerably more pronounced in Δ*ssaA* than in WT. Cells containing SigC as the only functional group 2 σ factor (the Δ*sigBDE* strain) showed a delayed recovery from nitrogen starvation [40] and SigC has been suggested to regulate nitrogen metabolism in stationary phase [54]. Altogether, these results suggest that recruitment of SigC by RNAP core prevent exit of cells from the stress-adapted state.

Likewise, SigB was recruited in larger quantities in Δ*ssaA* at the beginning of recovery. Cells without stress inducible SigB are vulnerable to many stress conditions including heat [55] and high salt [56, 57], and SigB helps to keep photosynthesis active under nitrogen deficiency [40]. It remains to be elucidated which role SigB might play in activation of the *sigA* gene during recovery from nitrogen deficiency induced stationary phase. However, a possible connection can be derived from previous experiments with dark/ light transitions [58].

The amount of SigE increases during nitrogen starvation and early recovery in both strains (Fig. 7h). However, the recruitment of SigE was less efficient in Δ*ssaA* than in WT in the early recovery phase. SigE has been earlier suggested to function in nitrogen stress responses [36, 39], and its role in triggering sugar catabolism is well-characterized [39, 41, 59]. Since *glgX* and *glgP* are positively controlled by SigE [60], the observed effect on SigE recruitment might underlie the decelerated glycogen degradation of the mutant strain. An anti-σ factor, ChlH, has been proposed to inhibit SigE recruitment in a light-dependent manner [61]. Moreover, the sRNA SyR11, which is presumably involved in post-transcriptional activation of *sigE* expression (unpublished results), showed significantly reduced levels in Δ*ssaA*, particularly during early recovery. Therefore, more detailed research on the involvement of integrated post-translational effects and - certainly - riboregulatory events will further disclose the complexity of the cyanobacterial σ factor network during physiological transitions. While not a conspicuous feature in this comparative study, the asRNA slr0653-as4 appears to be a potent inhibitor of *sigA* expression during starvation [43], exemplifying the significance of the RNA-regulatory layer in this process.

## Conclusion

Based on our current knowledge, we postulate a possible mechanism for 6S RNA-dependent regulation within the acclimation process to changing nitrogen supply in *Synechocystis* 6803. Formation of RNAP-SigA holoenzymes drastically decreases during nitrogen deprivation while recruitment of different group 2 σ factors either increases (SigC and SigE) or remains at the same level (SigB and SigD) as under standard conditions. After prolonged nitrogen deficiency, transcription activity is low in general [43], most probably because expression of household genes is depressed due to low formation of RNAP-SigA holoenzymes. During the recovery phase, group 2 σ factors are first to be efficiently recruited by RNAP core, in order to be replaced later on by the primary σ factor. This replacement process appears to be facilitated by 6S RNA. Due to a delayed formation of RNAP-SigA holoenzymes in the Δ*ssaA* strain, activation of many household genes occurs at a slower rate resulting in a decelerated re-synthesis and activation of light harvesting and photosynthetic complexes.

However, comprehensive binding studies and in vitro transcription analysis will be necessary to provide evidence for the direct regulatory impact of 6S RNA on σ factor dynamics in *Synechocystis* 6803. Thus far, this RNA was shown to functionally substitute for its homologue from *E. coli* in vitro, as it binds to *E. coli* RNAP, exhibits inhibitory activity on transcription and serves as a template for pRNA synthesis [28]. According studies of the native system – considering the plurality of group 2 σ factors – will certainly help to elucidate the mechanistic features of 6S RNA mediated transcriptional response to nitrogen availability in cyanobacteria.

## Methods

### Bacterial strains, growth conditions and experimental setup

The *Synechocystis* sp. PCC 6803 strain PCC-M [62] used in this study was provided by S. Shestakov (Moscow State University) and cultivated on 1% (*w*/*v*) agar (Bacto agar; Difco) plates containing BG-11 mineral medium [63]. Liquid cultures of WT and mutant strains were grown in BG-11 medium containing 10 mM TES buffer, pH 8.0, under continuous illumination with white light at 40-80 μmol photons m^−2^ s^−1^ at 30 °C, supplemented with a continuous stream of ambient air. BG11 plates for the cultivation of mutant strains were supplemented with 40 μg ml^−1^ kanamycin (∆*ssaA*) and 10 μg ml^−1^ chloramphenicol (WT-RNAP-HIS) or both antibiotics (Δ*ssaA*-RNAP-HIS), respectively. No antibiotics were added to the liquid cultures. Deviating growth conditions are specified in the corresponding figure legends or in the text. For nitrogen depletion, NaNO_3_ was omitted from the medium and cells were first washed twice and then resuspended in nitrogen-free medium. Cultures were incubated in nitrogen-depleted medium for 3-8 days. To induce nitrogen recovery, starved cells were supplemented with 17.6 mM NaNO_3_. Cells were harvested after cultivation in nitrogen-repleted medium for the indicated times (1 h, 4 h, 22 h, 48 h, 144 h).

### Construction of 6S RNA mutants and RNAP-HIS mutants

For the construction of a 6S RNA deletion mutant (Δ*ssaA*) the whole *ssaA* gene of *Synechocystis* 6803 was deleted by replacement with a kanamycin-resistance cassette (Km^R^). As distinguished from the previously described Δ*ssaA* mutant [32], the entire Km^R^ cassette from cloning vector pUC4K [64] harboring its own promoter was used for replacement of the chromosomal *ssaA* locus (1,886,879..1887066). The construct was prepared by overlap extension PCR of three fragments: (i) 697 bp region upstream of *ssaA* to chromosomal nucleotide position 1,886,182 (primer pair *slr1288_*fw/ 3’*slr1288*_rv, cf. Additional file 1: Table S1), (ii) 1027 bp Km^R^ cassette including 15 bp overhangs at both termini (primer pair Km_fw/ Km_rv), and (iii) 699 bp downstream of *ssaA* to chromosomal nucleotide position 1,887,765 (primer pair *sll1166*_fw/ 3’*sll1166*_rv). The ~700 bp flanking regions contained non-disrupted segments of *loci slr1288* and *sll1166,* and were used for homologous recombination. The Δ*ssaA-*mutant was selected on BG11 agar plates containing 50 μg ml^−1^ kanamycin. PCR analysis was used to verify complete segregation of WT chromosomal copies (Fig. 1b). The primers P1 (Δ*ssaA*_700up_fw) and P2 (Δ*ssaA*_700down_rv) are listed below in Additional file 1: Table S1. To create a strain overexpressing 6S RNA (6S(+)), the *ssaA* gene locus and its promoter region (~150 bp of upstream region) were inserted into the self-replicating vector pVZ321 [65], containing a chloramphenicol resistence gene using XhoI and XbaI. The vector was then transferred to WT cells by conjugation. Exconjugants were selected on BG11 agar plates containing 10 μg ml^−1^ chloramphenicol. Complementation of the Δ*ssaA* strain (Δ*ssaA*-c) was achieved by transferring the pVZ321 vector containing the *ssaA* gene plus ~150 bp of its upstream region and a chloramphenicol resistance gene to Δ*ssaA* mutant cells by conjugation [64]. The Δ*ssaA*-c-mutant was selected on BG11 agar plates containing 50 μg ml^−1^ kanamycin and 10 μg ml^−1^ chloramphenicol. For the generation of a WT-RNAP-HIS and Δ*ssaA*-RNAP-HIS mutant strain, *Synechocystis* 6803 WT and Δ*ssaA* cells were transformed using the plasmid pC-HIS-rpoC1-CAP-I-I (containing a nine amino acids long His-tag attached to the C terminal end of the γ subunit, and a chloramphenicol resistance cassette after the *rpoC1* gene) which was described earlier by Koskinen et al. [47]. WT-RNAP-HIS and Δ*ssaA*-RNAP-HIS mutants were probed on BG11 plates containing 10 μg ml^−1^ chloramphenicol and full segregation of the mutants was tested by PCR analysis using primers rpoC1His5 and rpoC1His6 (Additional file 1: Table S1) from Koskinen et al. [47].

### Analysis of growth, pigmentation and photosynthetic capacity

Whole cell absorbance spectra were recorded from 400 to 750 nm on a Specord® 200 PLUS spectrophotometer (Analytik Jena, Jena, Germany) and were corrected for a residual scattering at 750 nm. Chlorophyll was extracted in 90% methanol and the content was measured by spectrophotometry [66]. Contents of phycocyanin and allophycocyanin were determined in the soluble protein fraction according to Tandeau de Marsac and Houmard [66]. The pigment contents were normalized to the optical density at 750 nm. Photosynthetic capacity of cells was measured as light saturated oxygen evolution rates with a Clark-type oxygen electrode (Hansatech) in BG-11 medium in vivo in the presence of 10 mM NaHCO_3_ at 32 °C, illuminated with white light (photosynthetic photon flux density 3000 μmol m^−2^ s^−1^) [56]. In measurements of the maximum rate of photosynthesis, oxygen production by PSII is limited by the Calvin-Benson cycle, and addition of NaHCO_3_ allows the cycle to function at its maximal rate. To determine PSII capacity, the samples were supplemented with artificial electron acceptor 2,6-dimethoxybenzoquinone (DMBQ, 1 mM) and ferricyanide (1 mM). These chemicals are added in excess, and thus electron transfer through PSII is limited only by the maximum rate of PSII itself. In preparation for 77 K fluorescence emission spectroscopy, cells were concentrated to 35 μg Chl ml ^−1^, and 50 μl samples were immediately frozen in liquid nitrogen. The fluorescence was measured at 77 K with orange light excitation (580 nm) using an Ocean Optics S2000 spectrometer. The spectra were corrected with a moving median, subtraction of the dark level and division by the peak value of PSI at 721 nm and setting this value to 1. Differences in chlorophyll, allophycocyanin and phycocyanin content as well as of oxygen evolution rates were statistically analyzed using one-way ANOVA.

### Determination of glycogen content

The content of glycogen per ml cell suspension was determined as described by Gründel et al. [67]. Cell pellets of 0.5 - 1.5 ml culture were suspended in 30% (*w*/*v*) KOH and incubated for 2 h at 95 °C. An addition of ice-cold ethanol to a final concentration of 75% (*v*/v) and a following incubation on ice for at least 1.5 h were required for the precipitation of glycogen. The isolated glycogen was centrifuged for 15 min (16,000 *g*, 4 °C) and the pellet was washed with 70% and 98% (v/v) ice-cold ethanol and dried for 10 min at 60 °C. For the enzymatic hydrolysis of glycogen to glucose, the pellet was suspended in 100 mM sodium acetate (pH 4.5) including 2 mg/ml amyloglycosidase and incubated for 4 h at 95 °C. To determine the content of glucose, a hexokinase reagent was used according to the manufacturer’s protocol and measured spectrophotometrically.

### Isolation of RNA polymerase complex and immunoblot analysis

Cells from WT-RNAP-HIS and Δ*ssaA*-RNAP-HIS mutant strain (50 ml, OD_750nm_ = 0.8 - 1.1) were collected from growth at standard conditions, after 6d of growth in nitrogen depleted medium or after 1 h, 4 h and 22 h of recovery in nitrogen replete medium. The samples were broken and the soluble protein fraction was obtained as described in Koskinen et al. [47]. For the isolation of RNAP the Dynabeads®HIS-Tag Isolation and Pulldown Kit was applied according to the manufacturer’s instructions. 900 μg of soluble protein fraction was used for the pulldown and RNAP complexes were eluted to 75 μl of elution buffer [47]. 10 μl of His-tag purified RNAP samples were subjected to 10% NEXT GEL SDS-PAGE according to the protocol of Koskinen et al. [47]. RNAP complexes were transferred to Immobilon-P membrane using the semidry blotting method followed by incubation with specific polyclonal antibodies against σ factors SigA, SigB, SigC and SigE [47, 68, 69]. Proteins were visualized and quantified as described by Koskinen et al. [47]. Membranes were re-probed with antibodies against α or β subunits of the RNAP core [69] and the σ factor content was normalized to α (SigA, SigC, SigE) or β subunit content (SigB). Similarly, 5 μg of soluble proteins for SigA, 25 μg for SigB, 15 μg for SigC and 25 μg for SigE were used for separation on SDS-PAGE and were detected as described above.

### RNA isolation and northern blot hybridization

For the isolation of RNA, samples from discrete stages of cultivation were taken at the indicated times and were immediately put on ice and spun down at 4 °C. The preparation of total RNA from *Synechocystis* 6803 was essentially performed as described previously [70], using the Hot Trizol method, followed by 2-propanol precipitation. To determine the concentration and to check the quality of total RNA, we made use of a NanoDrop N-10000 spectrophotometer and electrophoretic separation on 1.3% agarose-formaldehyde gels. For Northern Blot detection, RNA samples were separated by electrophoresis either on 1.3% agarose-formaldehyde gels or 10% polyacrylamide-urea gels (urea-PAGE) and transferred to Hybond™-N^+^ membranes. The correct deletion of the *ssaA* gene was tested by northern blot analysis using a directed [^32^P]-labelled DNA oligonucleotide for 6S RNA. Alternatively, digoxigenin (DIG)-labelled RNA oligonucleotides (riboprobes) for 6S RNA, 5S rRNA as well as for SyR11 were used to analyze RNA accumulation levels. DIG-labelled riboprobes were prepared according to the manufacturer’s instructions using in vitro transcription with the AmpliScribe kit (T7-Flash Transcription kit, Epicentre) from a PCR product containing a T7 promotor. The PCR fragment was amplified with the primer pairs 6803ssaA-FwS/ T7-6S_6803_rev, 5S_6803_fw/ T7-5S_6803_rev and SyR11_fw/ SyR11_T7_rev, respectively (Additional file 1: Table S1).

Riboprobe hybridization was accomplished by over-night incubation at 62 °C, followed by chemiluminescent immunodetection using the DIG Northern Starter Kit (Roche).

### Microarray analysis

The microarray design, hybridization procedure and data analysis have been described previously [71]. The array is a whole genome gene expression array covering all known RNA features from tiling array microarray and dRNAseq transcriptome studies [46, 71]. Each feature is covered by up to four individual probes. All probes exist as technical replicates. For the microarray analysis total RNA samples from two biological replicates were used. Raw data were processed with the R package limma. Median signal intensity was cyclic loess normalized. The *p* values were generated by limma [72] and adjusted for multiple testing with the Benjamini Hochberg method.

## Additional files

Additional file 1: Table S1. Sequences of primers used for construction and PCR-based verification of Δ*ssaA*- and HIS-mutant strains, as well as for PCR-based generation of riboprobe templates. **Figure S1**. Pairwise comparison of whole cell absorbance spectra of *Synechocystis* 6803 WT and Δ*ssaA* strain shown in Fig. 2c. **Figure S2**.Photosynthetic parameters of wild type (WT) and ∆*ssaA* mutant during nitrogen starvation. **Figure S3.** Verification of the genetic complementation of *ssaA* gene disruption. **Figure S4**. Pigment analysis of WT and Δ*ssaA*. **Figure S5.** Glycogen consumption during recovery from nitrogen starvation. **Figure S6.** Volcanoplot showing the gene expression differences in WT and Δ*ssaA* after prolonged nitrogen depletion and before nitrogen re-addition (t_1_ = 0 h + N). **Figure S7.** Expression patterns of transcripts with significantly altered levels in the Δ*ssaA* mutant strain at early recovery from nitrogen depletion (t_2_ = 1 h + N). **Figure S8.** Expression patterns of transcripts with significantly altered levels in the Δ*ssaA* mutant strain at recovery from nitrogen depletion (t_3_ = 4 h + N.). **Figure S9.** Expression patterns of transcripts with significantly altered levels in the Δ*ssaA* mutant strain at recovery from nitrogen depletion (t_4_ = 22 h + N). **Figure S10.** Microarray data analysis showing exemplary details of gene expression in *Synechocystis* 6803 WT and ∆*ssaA* strain (depicted as d6S) at time points t_1_ = 0 h + N (7d –N); t_2_ = 1 h + N, t_3_ = 4 h + N and t_4_ = 22 h + N. **Figure S11**. Validation of Microarray data analysis. Expression of SyR11 in *Synechocystis* 6803. (DOCX 1909 kb)

Additional file 2: Overview of the genome-wide expression profile of *Synechocystis* 6803 wild type (WT) and Δ*ssaA* (d6S) strain under nitrogen depleted conditions (t_1_ = 0 h + N) and at recovery from nitrogen depletion (t_2_ = 1 h + N, t_3_ = 4 h + N, t_4_ = 22 h + N) by combining the log2 expression values (left scale) with read numbers of 454 sequencing (right scale). (PDF 42993 kb)

Additional file 3 Raw data of microarray analysis of *Synechocystis* 6803 wild type (WT) and Δ*ssaA* (d6S) strain. (XLSX 5837 kb)

## Abbreviations

*B. subtilis*: *Bacillus subtilis*
Chl: Chlorophyll *a*
Cm^r^: Chloramphenicol resistance cassette
DIG: Digoxygenin
DMBQ: 2,6-dimethoxybenzoquinone
dRNA-seq: differential RNA sequencing
*E. coli*: *Escherichia coli*
Km^r^: Kanamycin resistance cassette
N: Nitrogen
ncRNA: Non-coding RNA
nt: Nucleotide
OD: Optical density
PBS: Phycobilisome
pRNA: Product RNA
PSI: Photosystem I
PSII: Photosystem II
RNAP: RNA polymerase
sRNA: Small RNA
std: Standard
*Synechocystis* 6803: *Synechocystis* sp. PCC 6803
WT: Wild type
σ: Sigma factor
