# Supplementary material for: 6S RNA plays a role in recovery from nitrogen depletion in Synechocystis sp. PCC 6803
Source: BMC Microbiol. 2017 Dec 8;17:229. doi: 10.1186/s12866-017-1137-9 (PMC5721685; doi:10.1186/s12866-017-1137-9)
Supplement: Supplementary file 1 — Sequences of primers used for construction and PCR-based verification of ΔssaA- and HIS-mutant strains, as well as for PCR-based generation of riboprobe templates. Figure S1. Pairwise comparison of whole cell absorbance spectra of Synechocystis 6803 WT and ΔssaA strain shown in Fig. 2c. Figure S2.Photosynthetic parameters of wild type (WT) and ∆ssaA mutant during nitrogen starvation. Figure S3. Verification of the genetic complementation of ssaA gene disruption. Figure S4. Pigment analysis of WT and ΔssaA. Figure S5. Glycogen consumption during recovery from nitrogen starvation. Figure S6. Volcanoplot showing the gene expression differences in WT and ΔssaA after prolonged nitrogen depletion and before nitrogen re-addition (t1 = 0 h + N). Figure S7. Expression patterns of transcripts with significantly altered levels in the ΔssaA mutant strain at early recovery from nitrogen depletion (t2 = 1 h + N). Figure S8. Expression patterns of transcripts with significantly altered levels in the ΔssaA mutant strain at recovery from nitrogen depletion (t3 = 4 h + N.). Figure S9. Expression patterns of transcripts with significantly altered levels in the ΔssaA mutant strain at recovery from nitrogen depletion (t4 = 22 h + N). Figure S10. Microarray data analysis showing exemplary details of gene expression in Synechocystis 6803 WT and ∆ssaA strain (depicted as d6S) at time points t1 = 0 h + N (7d –N); t2 = 1 h + N, t3 = 4 h + N and t4 = 22 h + N. Figure S11. Validation of Microarray data analysis. Expression of SyR11 in Synechocystis 6803. (DOCX 1909 kb) [file 12866_2017_1137_MOESM1_ESM.docx]

6S RNA plays a role in recovery from nitrogen depletion in *Synechocystis* sp. PCC 6803

Beate Heilmann, Kaisa Hakkila, Jens Georg, Taina Tyystjärvi, Wolfgang R. Hess, Ilka M. Axmann, Dennis Dienst

**Table S1**

Sequences of primers used for construction and PCR-based verification of Δ*ssaA*- and HIS-mutant strains, as well as for PCR-based generation of riboprobe templates. The T7 promoter sequence is each underlined. Oligonucleotides used for DNA 5´end-labeling.

| Name | Sequence (5´- 3´) |
| --- | --- |
| Primer | |
| Δ*ssaA*_700up_fw (P1) | caccgcctaccctgaatatctg |
| Δ*ssaA*_700down_rv (P2)  *slr1288*_fw (P3)  3´*slr1288*_rv (P4)  Km_fw (P5)  Km_rv (P6)  *sll1166*_fw (P7)  3´*sll1166*_rv (P8) | Caaggaattagaccgcatcgg  GGAGCCCCTAAGCCGTACC  CCTGCCATTATAACCCCATCCTTCG  GGTTATAATGGCAGGGGGGGGGGGGGGAAAGC  ACCTGAGAAGCAGAGCCGTCCCGTCAAGTCAGCGTAAT  CTCTGCTTCTCAGGTTTATCCCTCC  GGTAGATACCCTGGTCATTCTGGTTTTG |
| rpoC1His5 | GAGGTCATCAAAACCGAGGA |
| rpoC1His6 | CAAAGGGAGCAGGATCAAAA |
| 6803ssaA-FwS  T7-6S_6803_rev  5S_6803_fw  T7-5S_6803_rev  SyR11_fw | GAAGTAACTACCGCGTTGGTGACTG  TAATACGACTCACTATAGGGAGAAGCACCACCACGCCG  TCTTGGTGTCTTTAGCGTCATGGAAC  TAATACGACTCACTATAGGGACTTGGCATCGGACTATTGTGC  AACGAAATAACACGGGGTCAC |
| SyR11_T7_rev | TAATACGACTCACTATAGGGacaattttaaagggatcaag |
| Oligonucleotides | |
| 5S rRNA-Oligo | GCATCGGACTATTGTGCCGTG |
| 6S RNA-Oligo | caccacgccgttttacct |

**
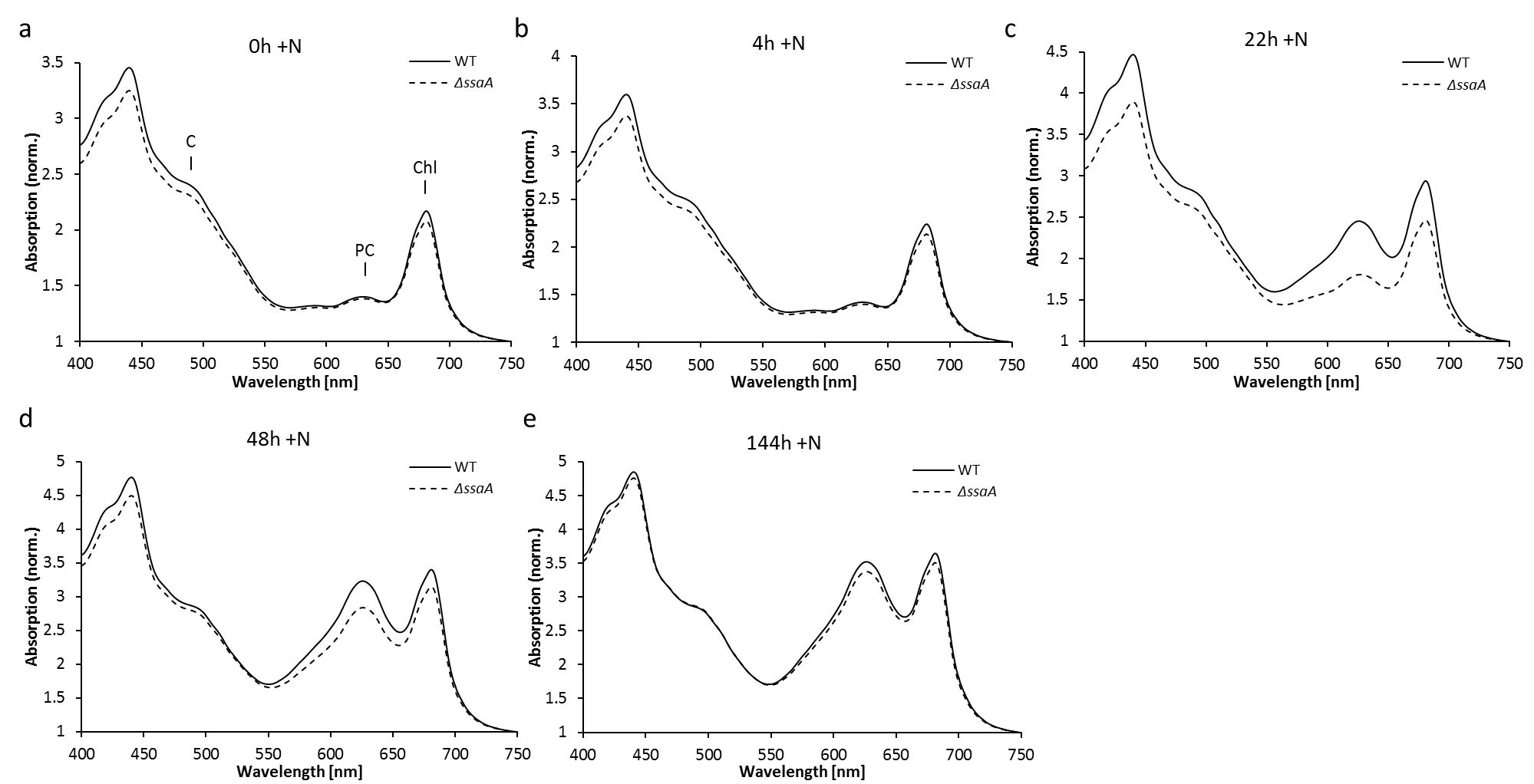
**

**Figure S1**

Pairwise comparison of whole cell absorbance spectra of *Synechocystis* 6803 WT and Δ*ssaA* strain shown in Fig. 2c. Cultures were grown under continuous white light at an irradiance of 80 µmol photons m^-2^ s^-1^, at 30 °C, in nitrogen-depleted medium for 189 hours before recovery was initiated by adding nitrogen. Whole cell absorbance spectra were measured at nitrogen deficiency for 189h (t= 0h +N; (a)) and at recovery time points t= 4h, (b); t= 22h, (c); t= 48h, (d) and 144h (+N), (e). The spectra were normalized at 750 nm. C: carotenoids; PC: phycocyanine; Chl: chlorophyll *a*


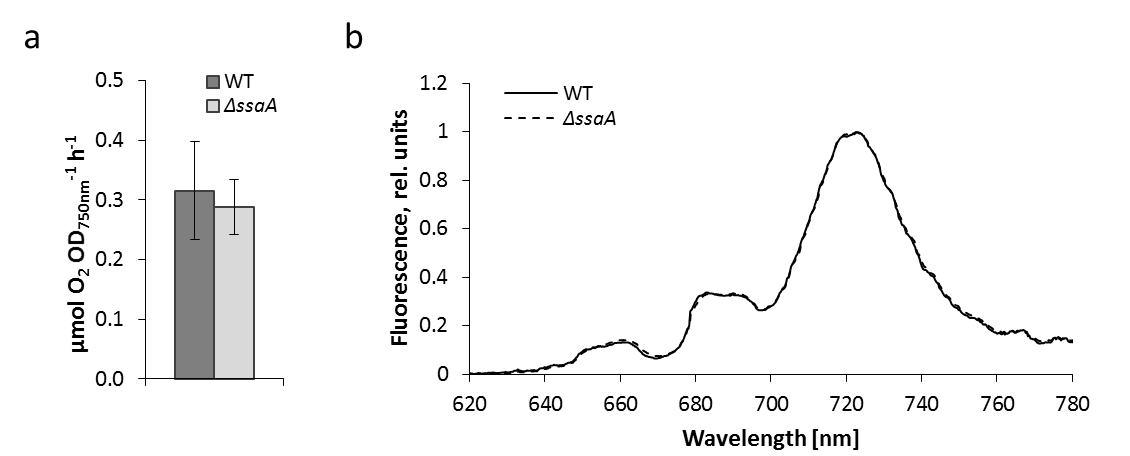


**Figure S2**

Photosynthetic parameters of wild type (WT) and ∆*ssaA* mutant during nitrogen starvation. (a) Oxygen evolution rates of photosynthesis examined *in vivo* at time point t= 72h -N under light-saturated conditions (3000 µmol photons m^-2^ s^-1^). Three technical replicates were measured of two independent biological replicates of each strain. The error bars represent the standard deviation (SD). (b) Orange light-excited fluorescence emission spectra of WT and ∆*ssaA* mutant measured at 77 K under nitrogen deficiency at time point t= 96h -N. Three independent biological replicates were measured and the spectra were normalized by dividing by the peak value of PSI at 721 nm and setting this value to 1.

**
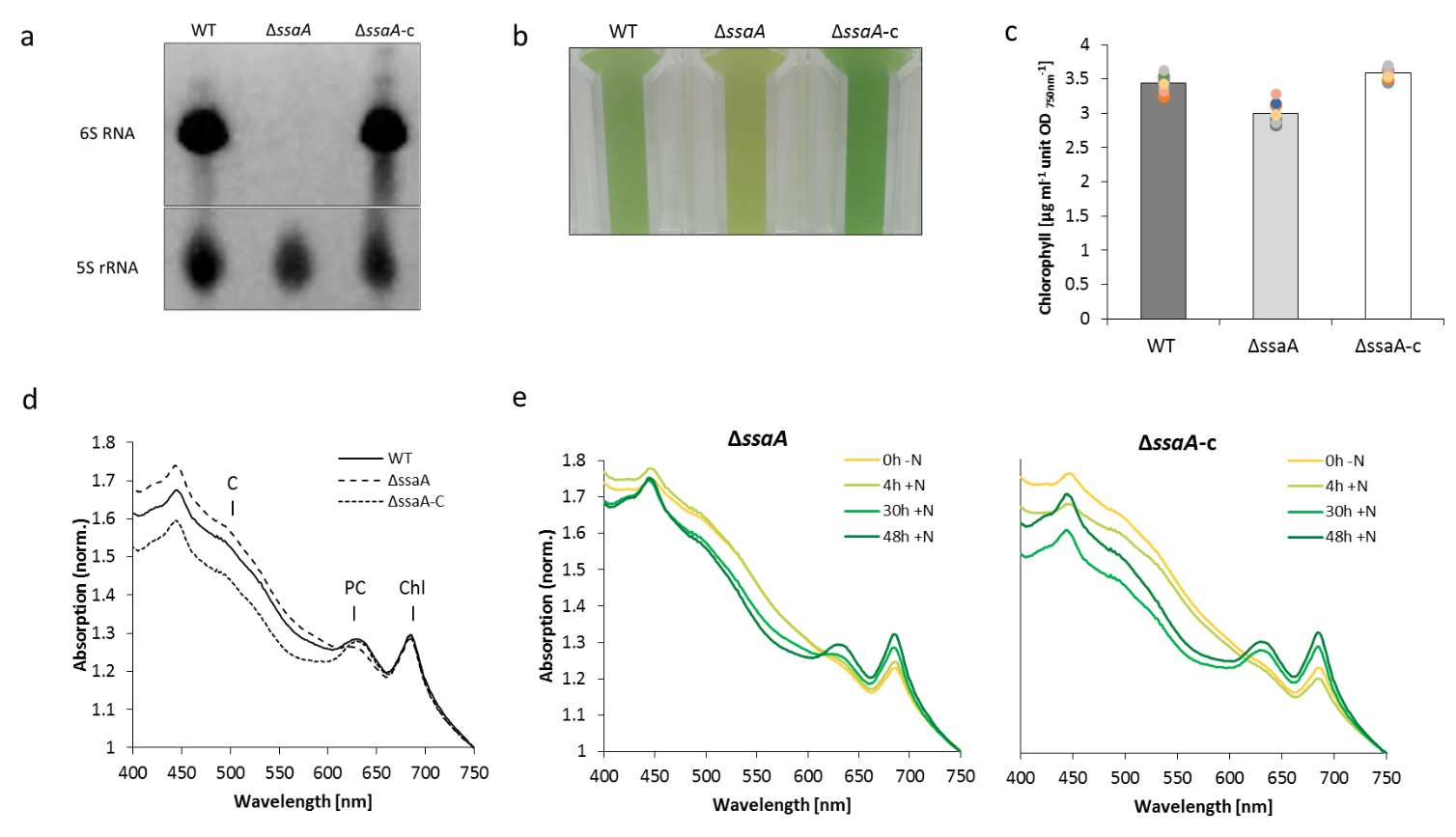
**

**Figure S3**

Verification of the genetic complementation of *ssaA* gene disruption. (a) Complementation of *ssaA* deletion in strain Δ*ssaA*-c was confirmed by Northern Blot analysis. Each 5 µg of total RNA isolated from *Synechocystis* 6803 WT, Δ*ssaA* and Δ*ssaA*-C were separated by urea-PAGE and transferred to a positively charged nylon membrane. Hybridization of 6S RNA and 5S rRNA (loading control) was performed using DIG-labeled riboprobes. (b – e) Comparative physiological characterization of WT, Δ*ssaA* and Δ*ssaA*-C during recovery from nitrogen starvation. Cultures were grown at 30 °C in shaking flasks at an irradiance of 40 µmol photons m^-2^ s^-1^. (b) Photograph of cultures of WT, Δ*ssaA* and Δ*ssaA*-C strain at time point 30h +N during recovery. (c) Chlorophyll *a* content of the cultures at recovery time point 30h +N. The bars in the illustrated diagram represent the mean from three replicate cultures that were each measured in triplicate (n= 9). Additionally, the single values are illustrated separately as colored dots in the diagram. (d) Comparison of whole cell absorption spectra of WT, Δ*ssaA* and Δ*ssaA*-C strain at recovery time point 30h +N. C: carotenoids; PC: phycocyanine; Chl: chlorophyll *a*. (e) Absorbance spectra of Δ*ssaA* and Δ*ssaA*-C mutant strains are illustrated for the time point t= 0h +N, which corresponds to 188h under nitrogen deficiency (-N) and for the time points t= 4, t= 30 and t= 48 hours after nitrogen addition (+N). The spectra were normalized to the optical density at 750 nm.

**
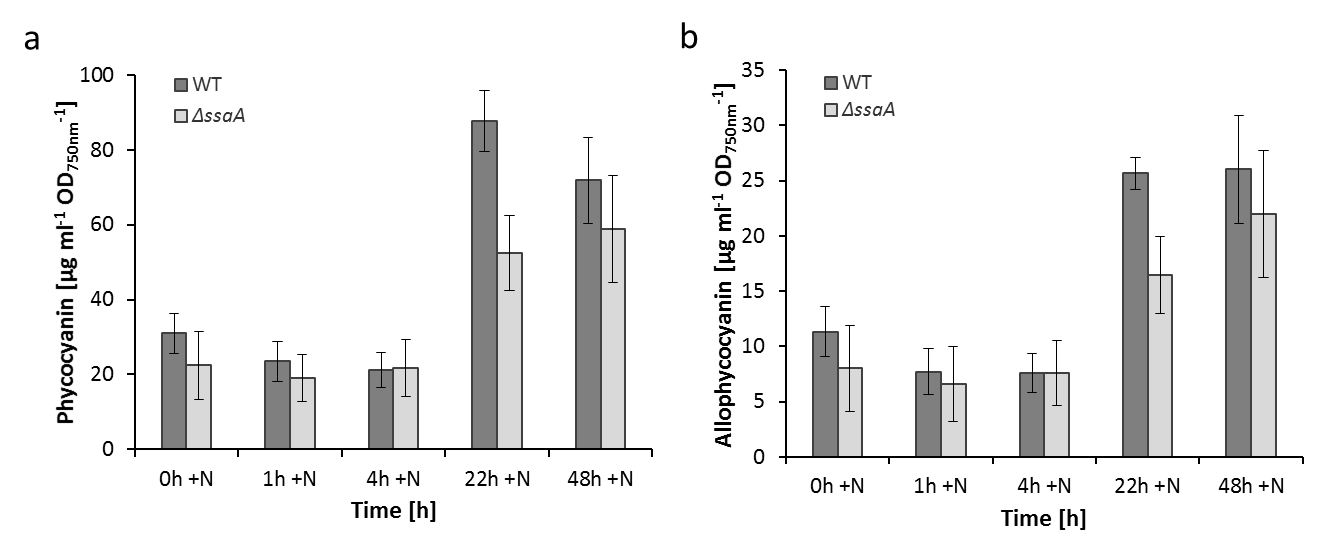
**

**Figure S4**

Pigment analysis of WT and Δ*ssaA*. Phycocyanin (a) and allophycocyanin (b) were measured from the soluble protein fraction 158h under nitrogen deficiency (t= 0h +N) and at recovery time points t= 1h +N, t= 4h +N, t= 22h +N and t= 48h +N. The data illustrate the mean from three independent biological replicates and the error bars represent the SD.


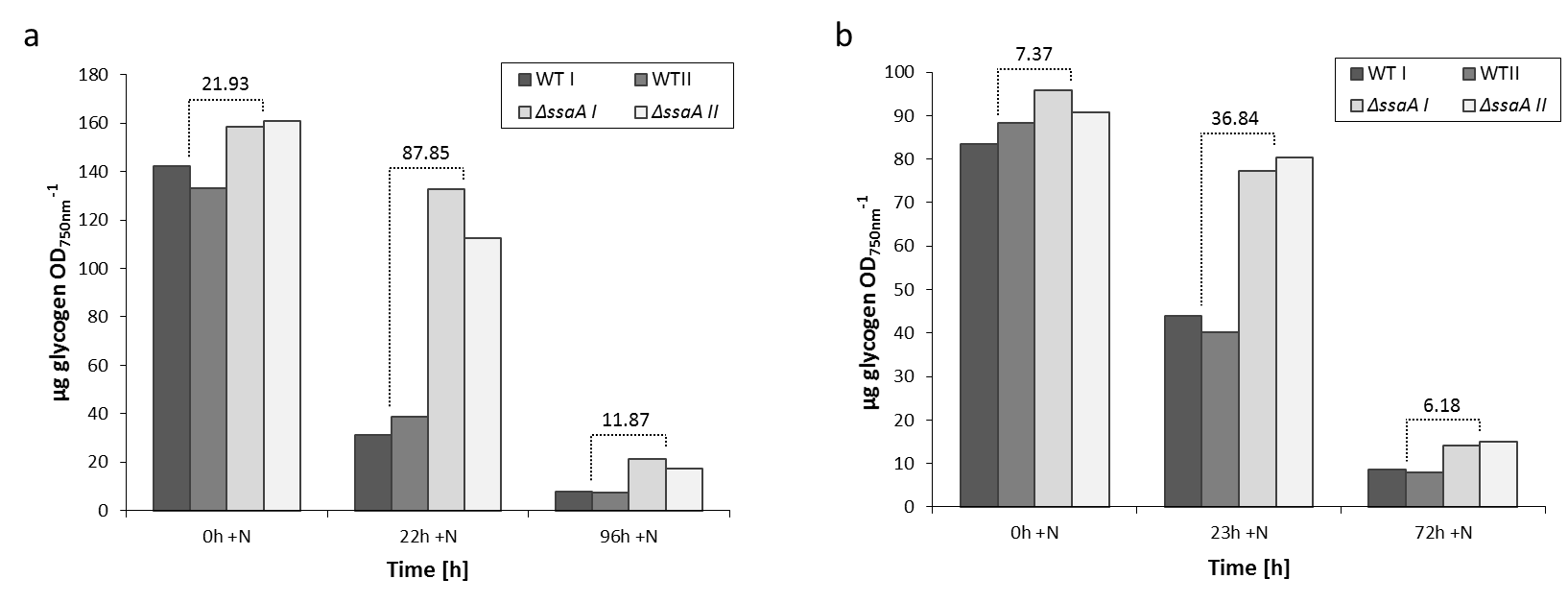


Figure S5

Glycogen consumption during recovery from nitrogen starvation. Glycogen content was determined for two independent experimental setups ((a) and (b)) in which *Synechocystis* 6803 WT and Δ*ssaA* were cultivated in nitrogen-depleted medium for at least 6 days before recovery process was induced by transferring cells to NaNO_3_-supplemented medium. (a) Corresponding data of the microarray analysis (Figs. 5 and 6), originating from the same experimental cycle. Glycogen content was measured after 7 days under nitrogen deficiency (0h +N) and during recovery at time points 22h and 96h +N. (b) Cells were cultivated under nitrogen deprivation for 6 days (0h +N) before recovery was triggered by nitrogen addition. Glycogen content was analyzed at recovery time points 23h +N and 72h +N. Two biological replicates of each strain were analyzed, named as I and II, respectively. The average difference for each time point is indicated by the values above the bars.


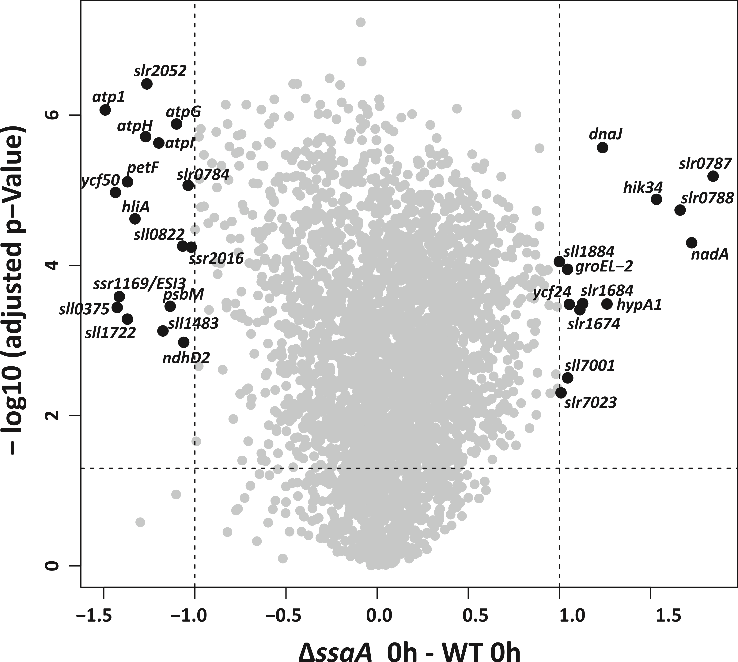


Figure S6

Volcanoplot showing the gene expression differences in WT and Δ*ssaA* after prolonged nitrogen depletion and before nitrogen re-addition (t_1_= 0h +N). Significantly differentially expressed RNA features (|log2 FC| ≥ 1, adj. p-Value ≤ 0.05) are drawn as black points. For detailed data see supplementary files 2 and 3.


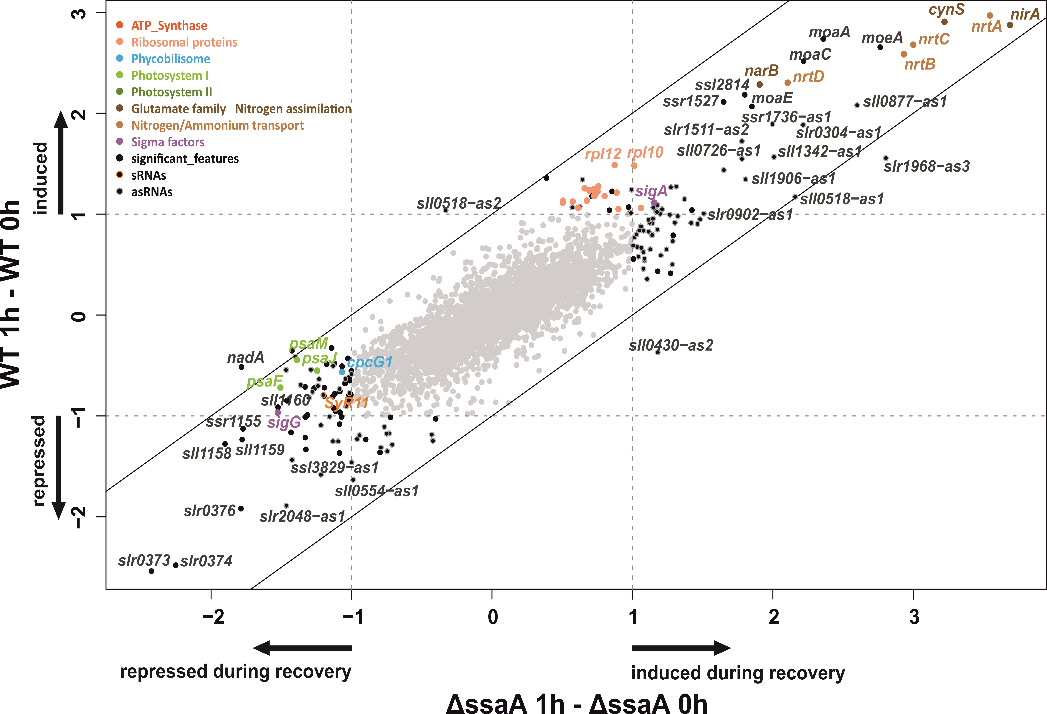


Figure S7

Expression patterns of transcripts with significantly altered levels in the Δ*ssaA* mutant strain at early recovery from nitrogen depletion. Total RNA from the sampling time point t_2_= 1h +N was subjected to microarray analysis. The scatter plot shows the nitrogen recovery response in Δ*ssaA* (x-axis) versus the response in WT (y-axis) for t_2_= 1h +N in comparison to the 0h time point (see Fig. S6 for comparison). Significantly differentially expressed RNA features (|log2 FC| ≥ 1, adj. p-Value ≤ 0.05) are drawn as black points. Points beyond the transverse black lines indicate a different response to the recovery (|(WT xh – WT 0h) – (Δ*ssaA* xh – Δ*ssaA* 0h)| ≥ 1). Selected functional groups regarding to the Cyanobase GO classification are color coded. The nitrogen/ammonium transport group was defined manually and contains *nrtA, nrtB, nrtC, nrtD, glnH, sll0536, amt3, amt2, amt1* and *amiC*. Differentially expressed asRNAs and sRNAs are indicted by a grey or orange border. See also Figs. S8 and S9. For detailed data see supplementary files 2 and 3.


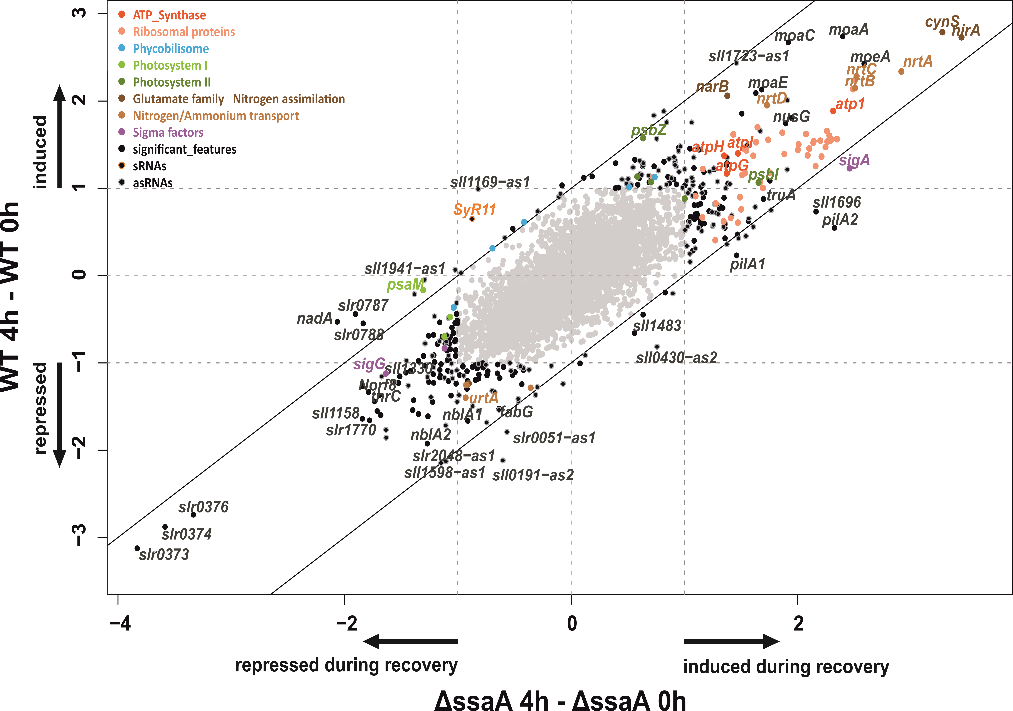


Figure S8

Expression patterns of transcripts with significantly altered levels in the Δ*ssaA* mutant strain at recovery from nitrogen depletion. Total RNA from the sampling time point t_3_= 4h +N was subjected to microarray analysis. The scatter plot shows the nitrogen recovery response in Δ*ssaA* (x-axis) versus the response in WT (y-axis) for t_3_= 4h +N in comparison to the 0h time point (see Fig. S6 for comparison). Significantly differentially expressed RNA features (|log2 FC| ≥ 1, adj. p-Value ≤ 0.05) are drawn as black points. Points beyond the transverse black lines indicate a different response to the recovery (|(WT xh – WT 0h) – (Δ*ssaA* xh – Δ*ssaA* 0h)| ≥ 1). Selected functional groups regarding to the Cyanobase GO classification are color coded. The nitrogen/ammonium transport group was defined manually and contains *nrtA, nrtB, nrtC, nrtD, glnH, sll0536, amt3, amt2, amt1* and *amiC*. Differentially expressed asRNAs and sRNAs are indicted by a grey or orange border. See also Figs. S7 and S9. For detailed data see supplementary files 2 and 3.


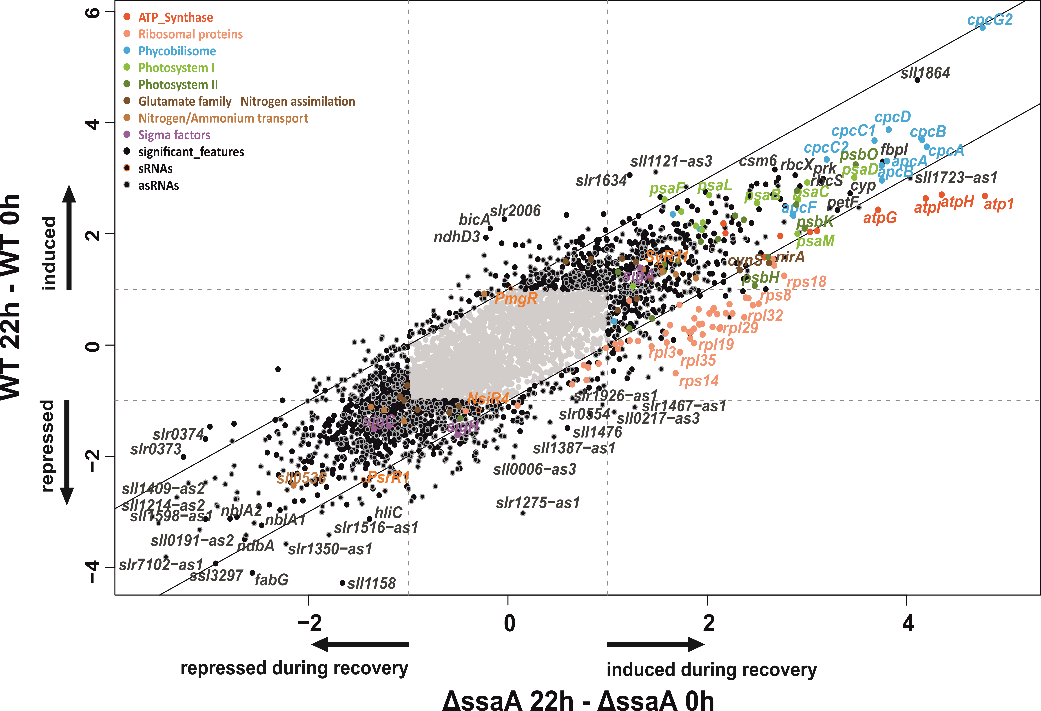


Figure S9

Expression patterns of transcripts with significantly altered levels in the Δ*ssaA* mutant strain at recovery from nitrogen depletion. Total RNA from the sampling time point t_4_= 22h +N was subjected to microarray analysis. The scatter plot shows the nitrogen recovery response in Δ*ssaA* (x-axis) versus the response in WT (y-axis) for t_4_= 22h +N in comparison to the 0h time point (see Fig. S6 for comparison). Significantly differentially expressed RNA features (|log2 FC| ≥ 1, adj. p-Value ≤ 0.05) are drawn as black points. Points beyond the transverse black lines indicate a different response to the recovery (|(WT xh – WT 0h) – (Δ*ssaA* xh – Δ*ssaA* 0h)| ≥ 1). Selected functional groups regarding to the Cyanobase GO classification are color coded. The nitrogen/ammonium transport group was defined manually and contains *nrtA, nrtB, nrtC, nrtD, glnH, sll0536, amt3, amt2, amt1* and *amiC*. Differentially expressed asRNAs and sRNAs are indicted by a grey or orange border. See also Figs. S7 and S8. For detailed data see supplementary files 2 and 3.

**
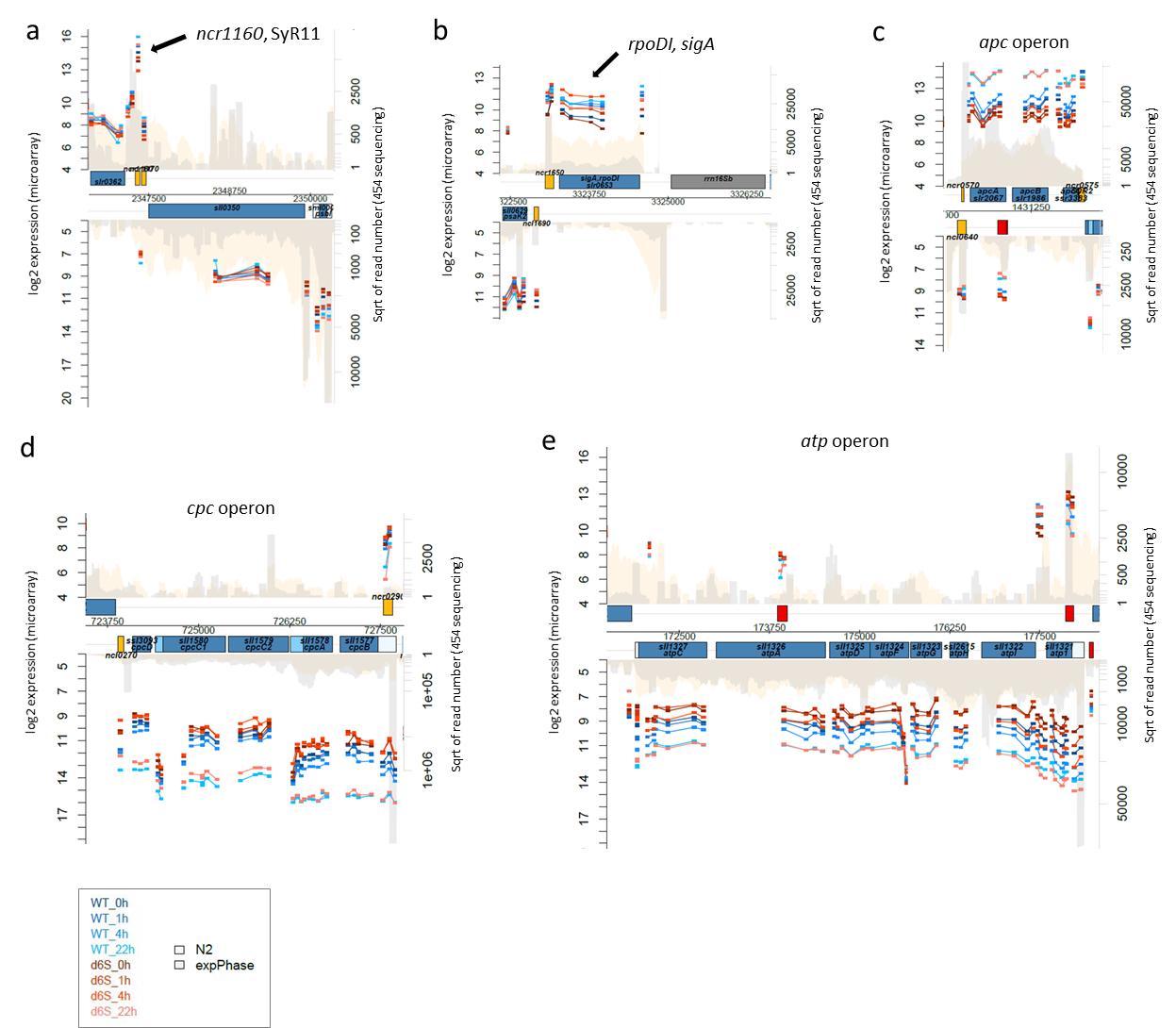
**

**Figure S10**

Microarray data analysis showing exemplary details of gene expression in *Synechocystis* 6803 WT and *∆ssaA* strain (depicted as d6S) at time points t_1_=0h +N (7d –N); t_2_=1h +N, t_3_=4h +N and t_4_=22h +N. The following genome sections are pictured: (a) SyR11 gene (*ncr116*0; indicated by a black arrow), (b) sigA gene (*rpoDI*; indicated by a black arrow), (c) Allophycocyanin genes (*apcA* and *apcB*) and phycobilisome small linker polypeptide (*apcC*), (d) Phycobilisome small rod linker polypeptide genes (*cpcD*, *cpcC1*, *cpcC2*) and genes encoding for phycocyanin α subunit (*cpcA*) and β subunit (*cpcB*) and (e) ATP synthase gene operon (genes *atpC*, *atpA*, *atpD*, *atpF*, *atpG*, *atpH*, *atpI*, *atp1*).


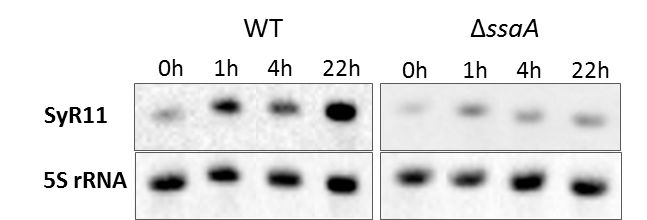


**Figure S11**

Validation of Microarray data analysis. Expression of SyR11 in *Synechocystis* 6803. Verification experiment for the accumulation of SyR11 transcript levels at time points t_1_= 0h, t_2_= 1h, t_3_= 4h and t_4_= 22h +N. 2µg RNA were subjected to Northern blot hybridization using a digoxigenin-labeled RNA oligonucleotide specific for SyR11. 5S rRNA was probed as a loading control.
