# Supplementary figures and images for: 6S RNA plays a role in recovery from nitrogen depletion in Synechocystis sp. PCC 6803
Source: BMC Microbiol. 2017 Dec 8;17:229. doi: 10.1186/s12866-017-1137-9 (PMC5721685; doi:10.1186/s12866-017-1137-9)

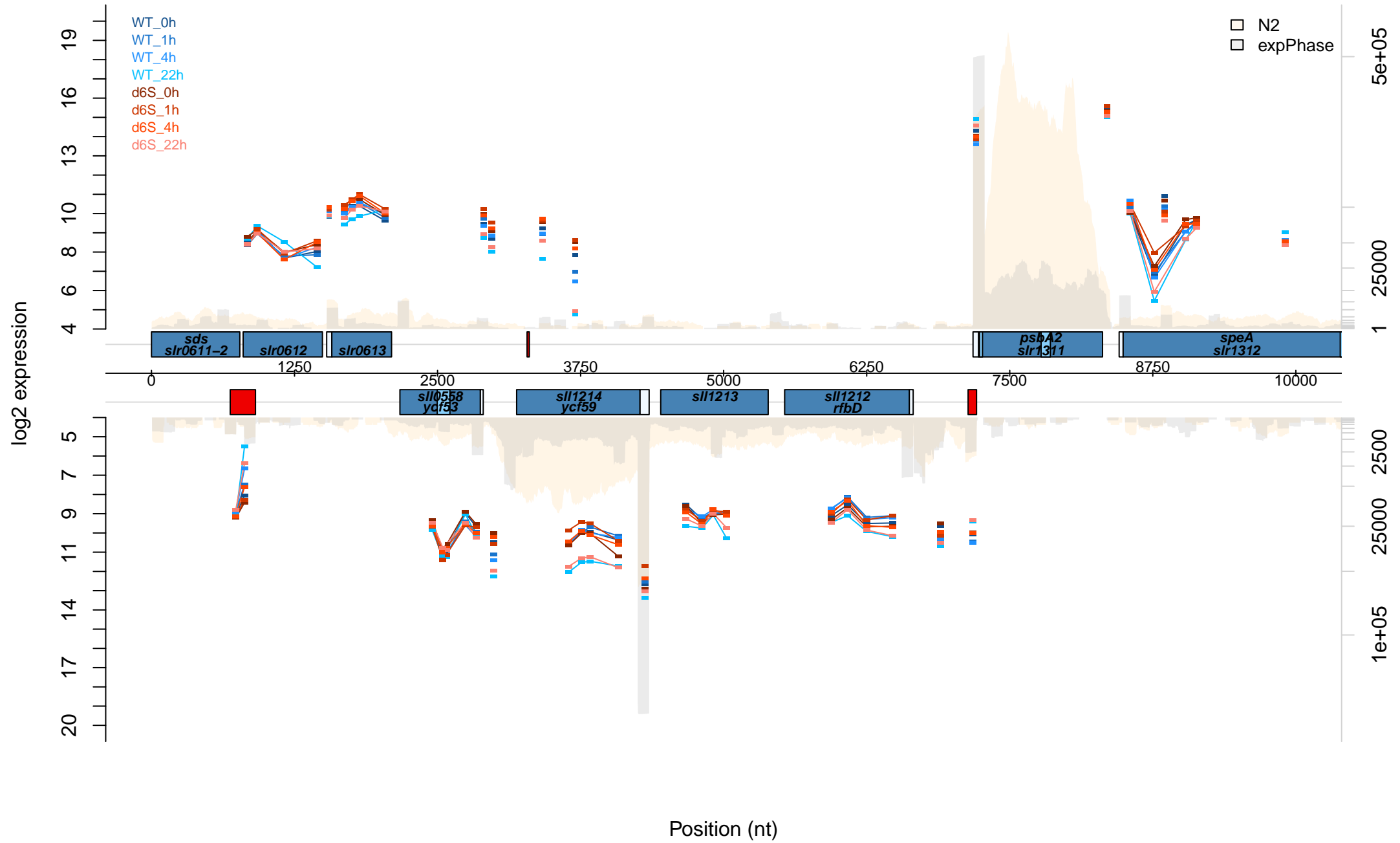

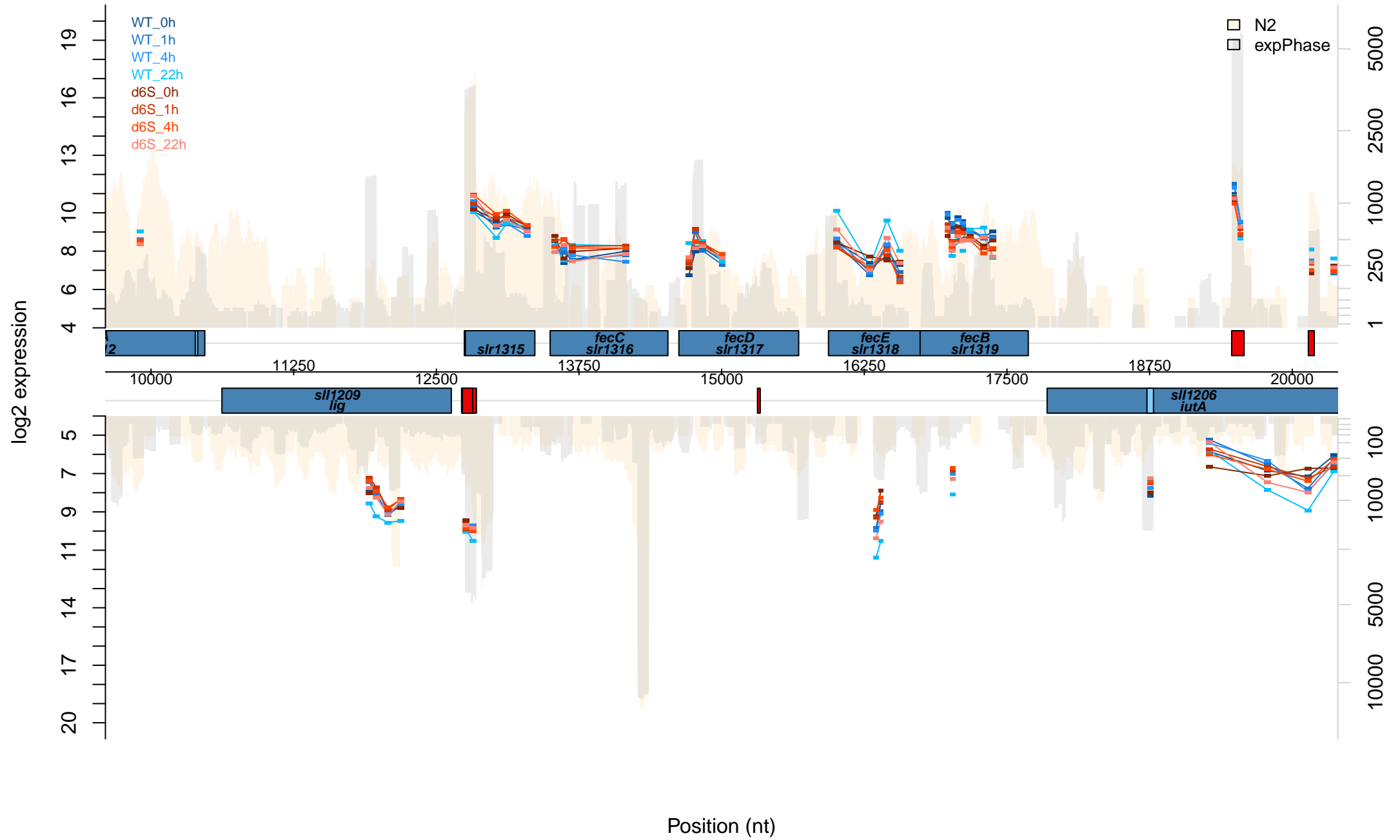

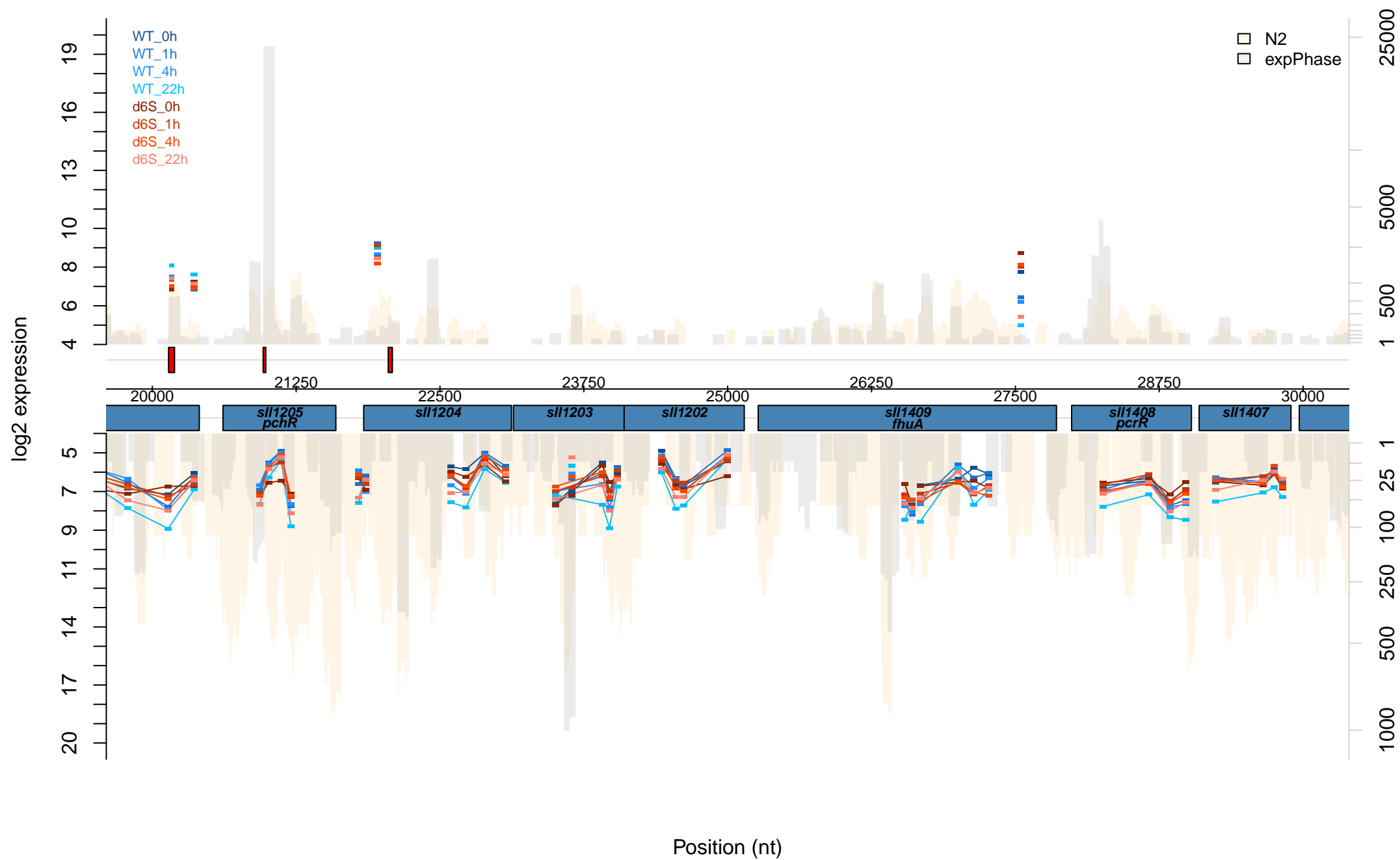

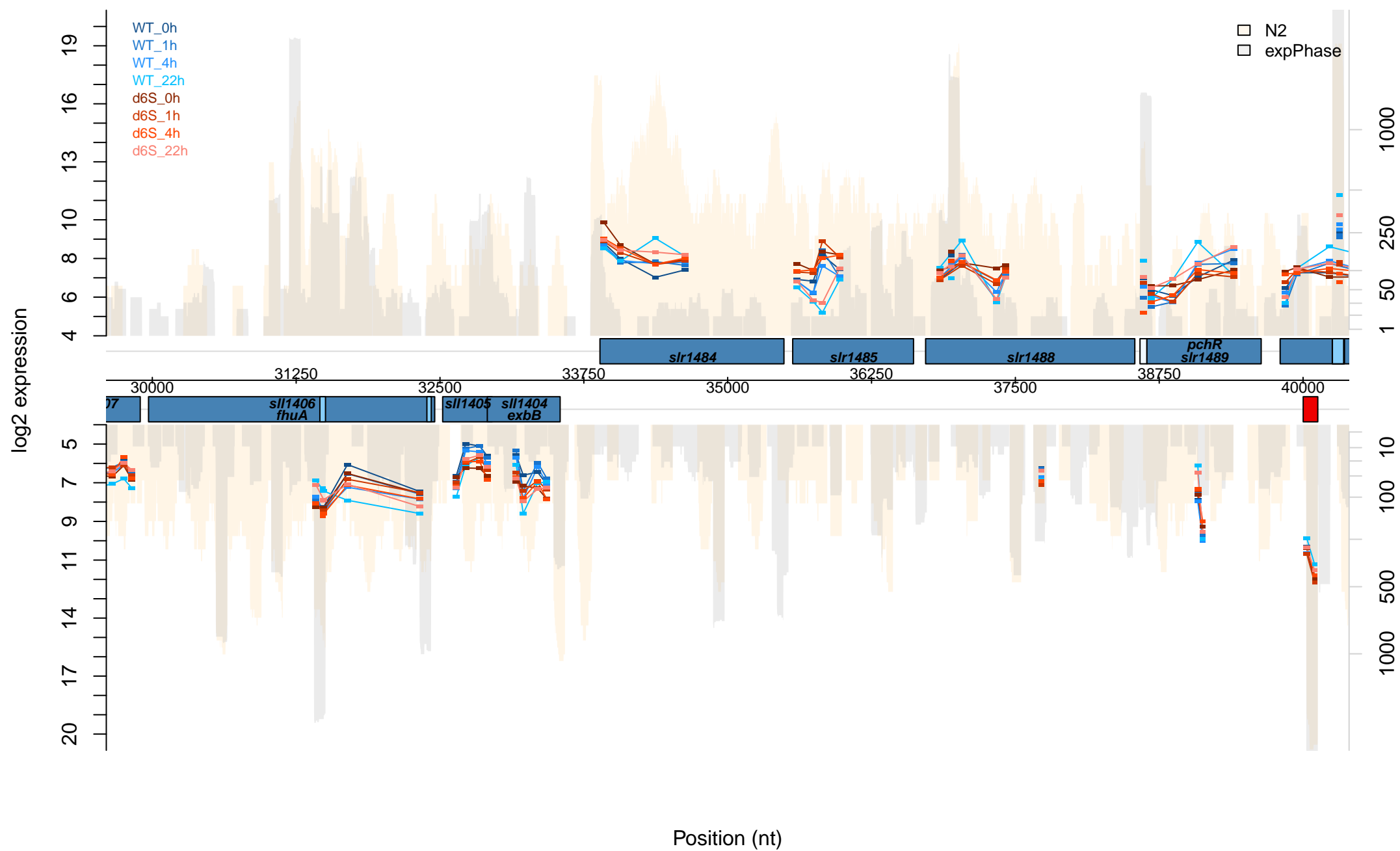

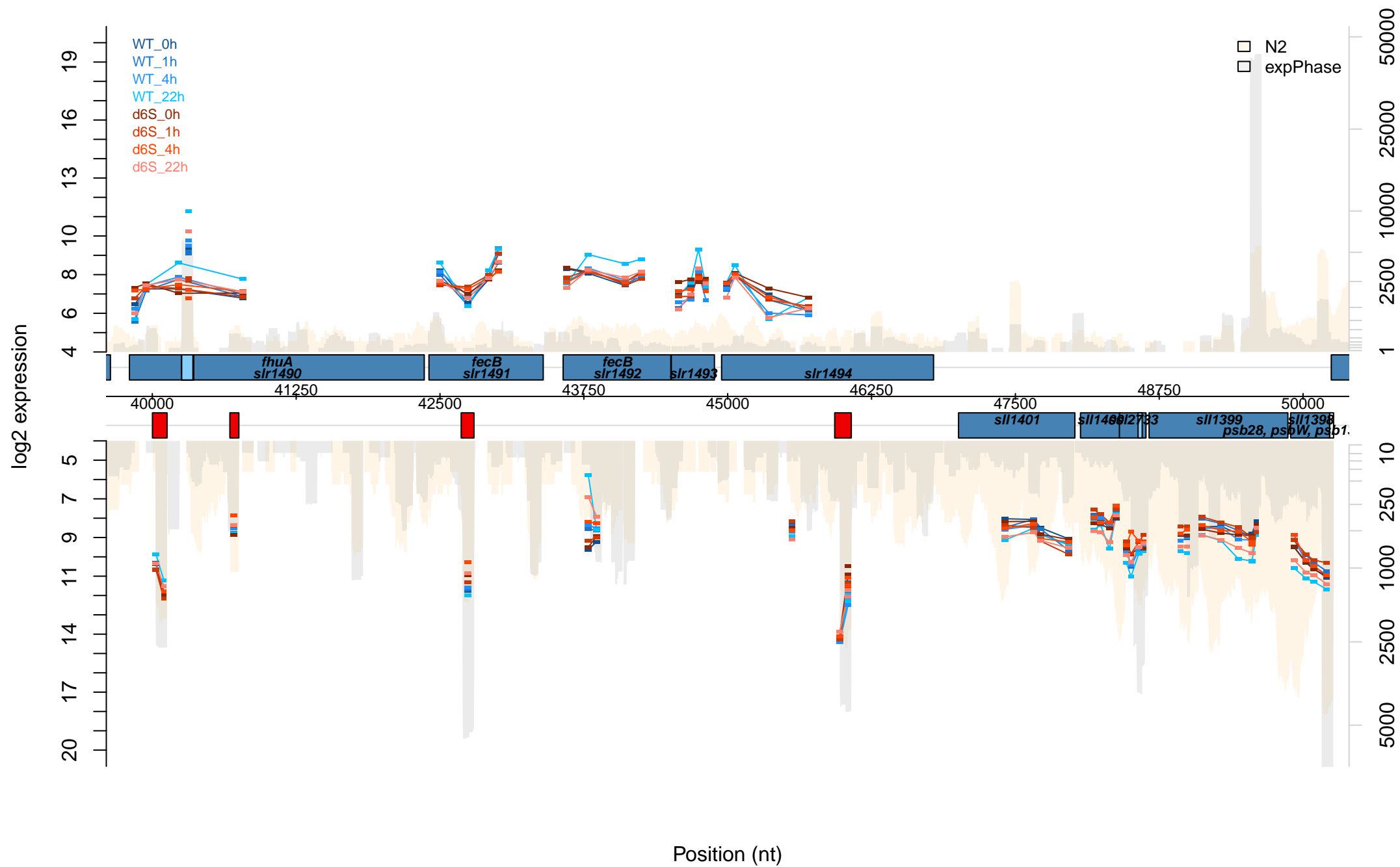

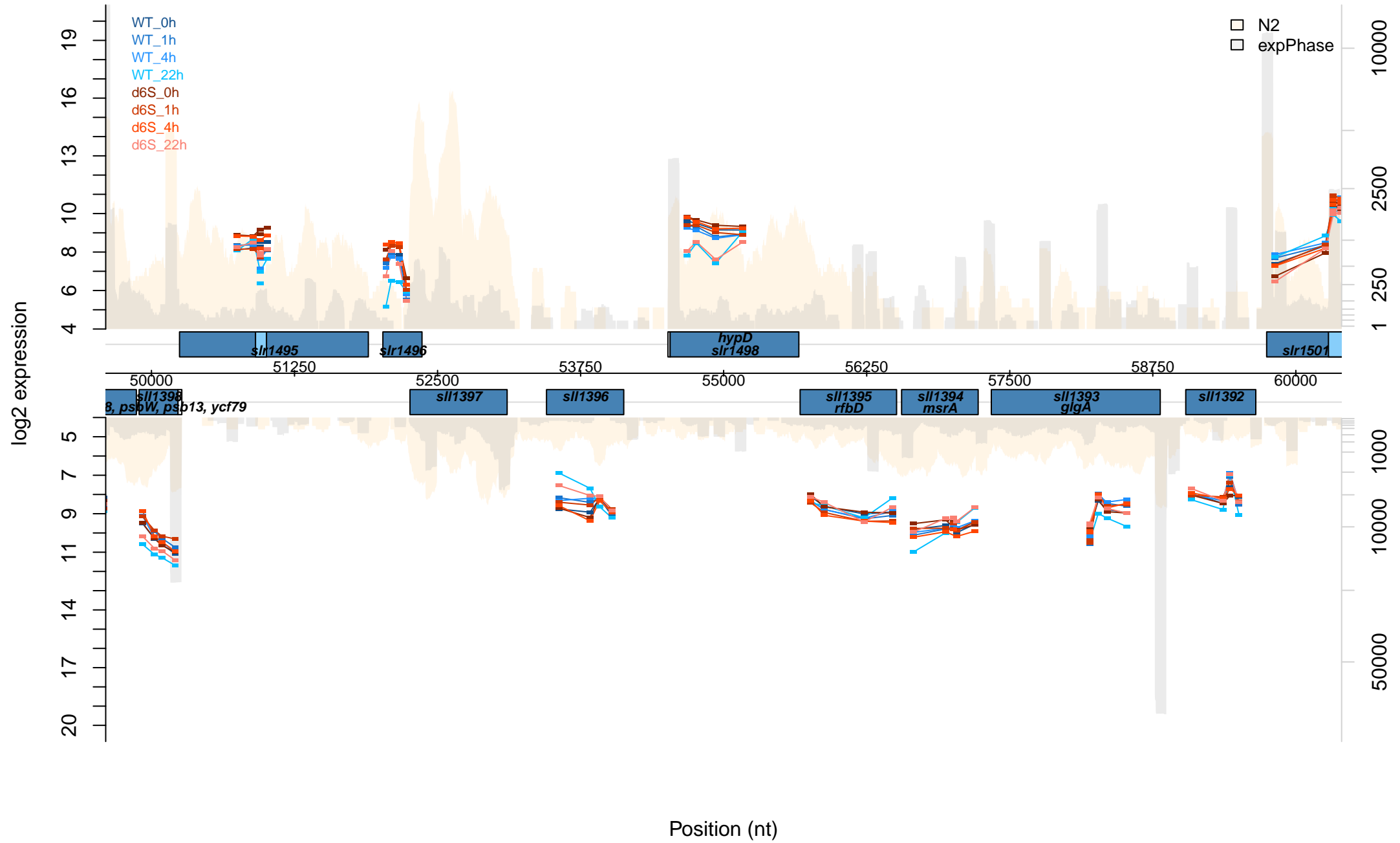

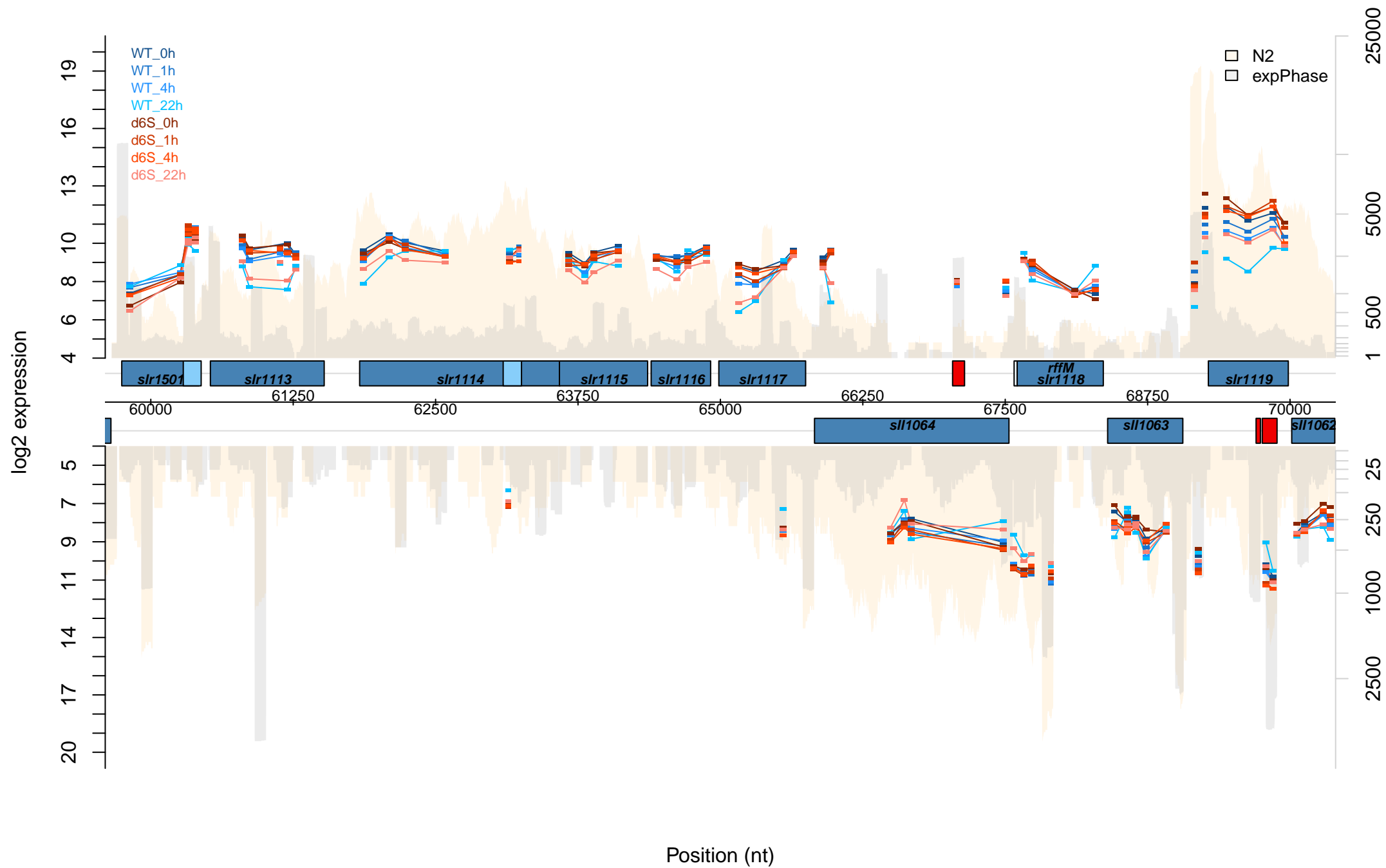

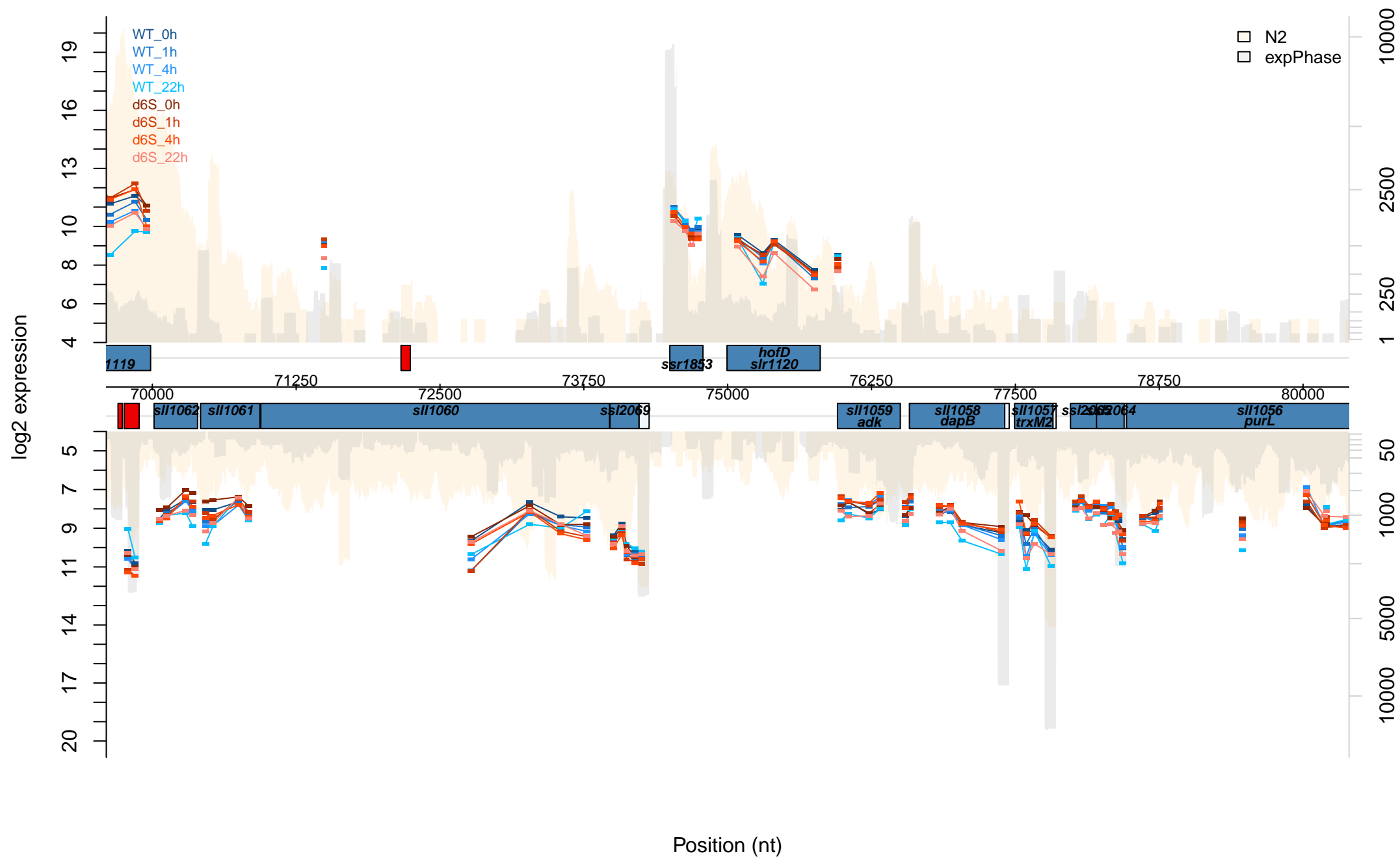

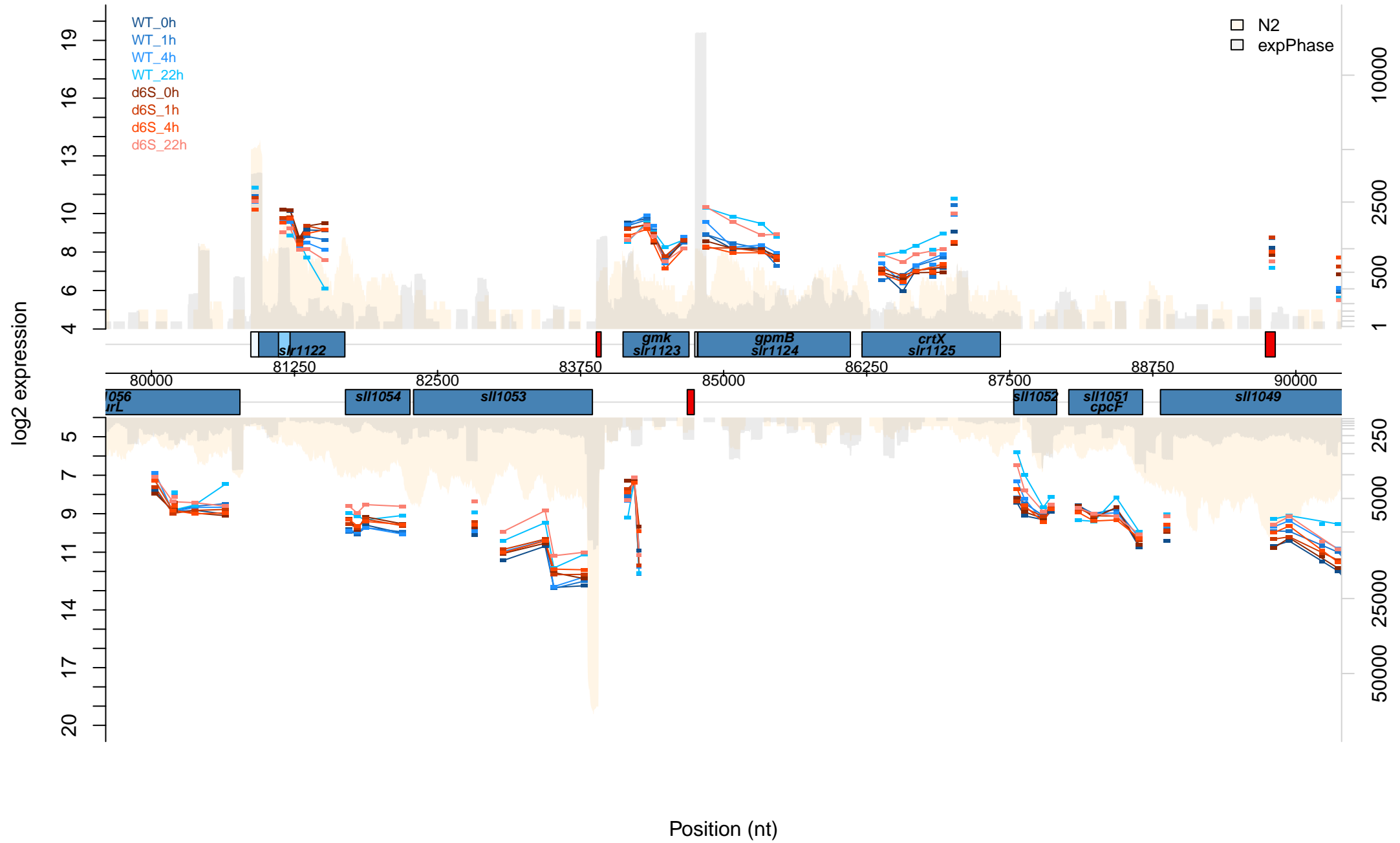

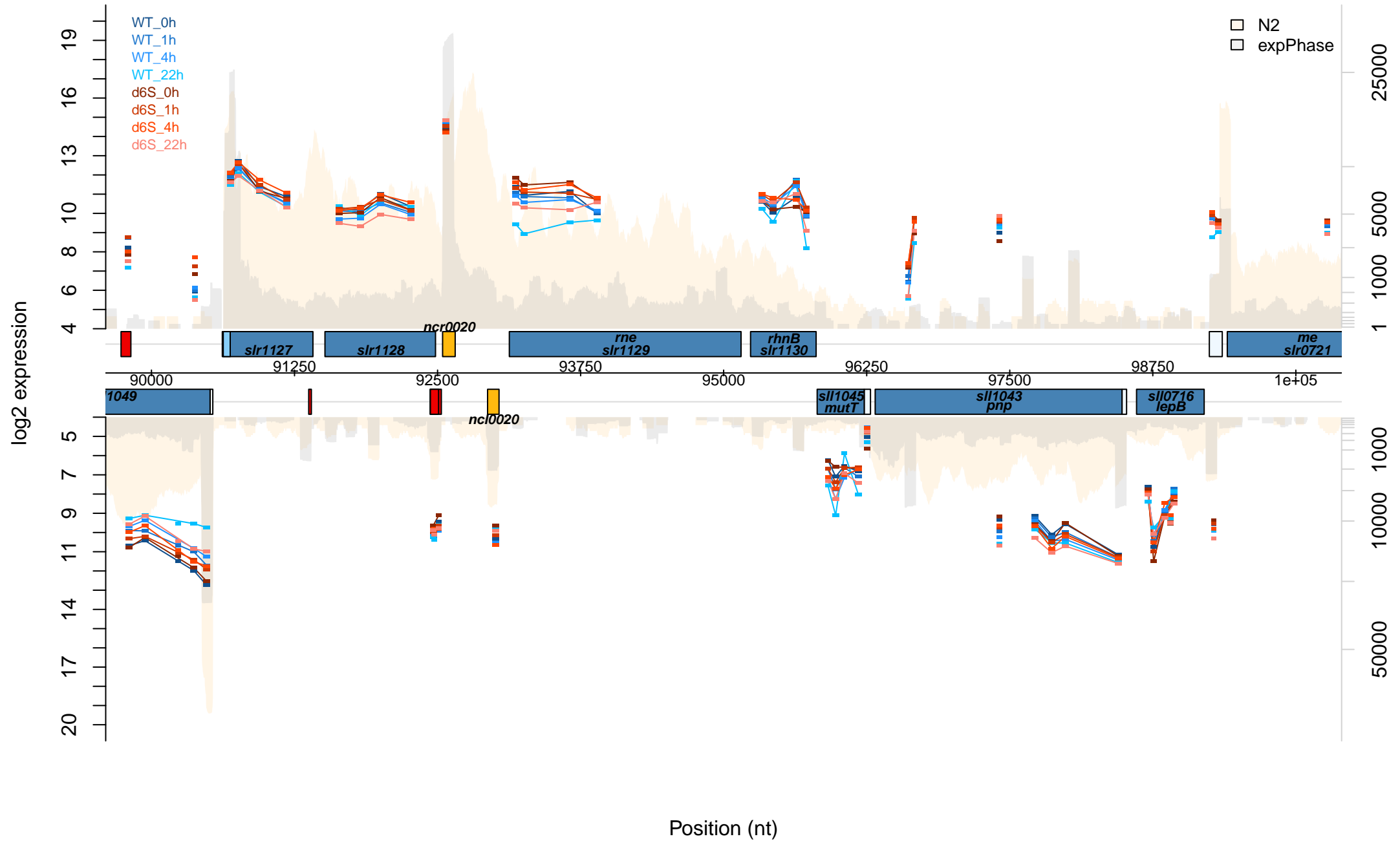

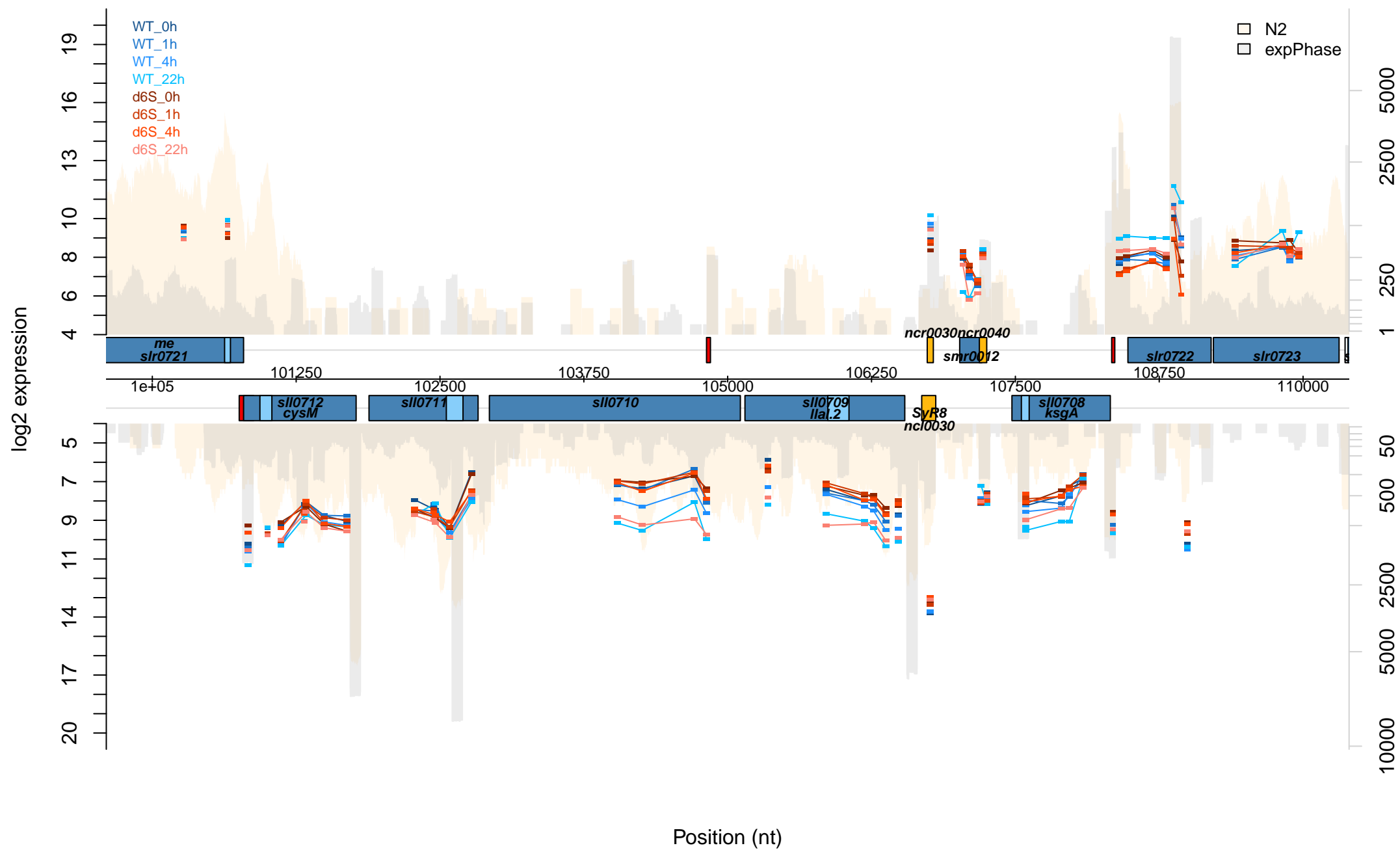

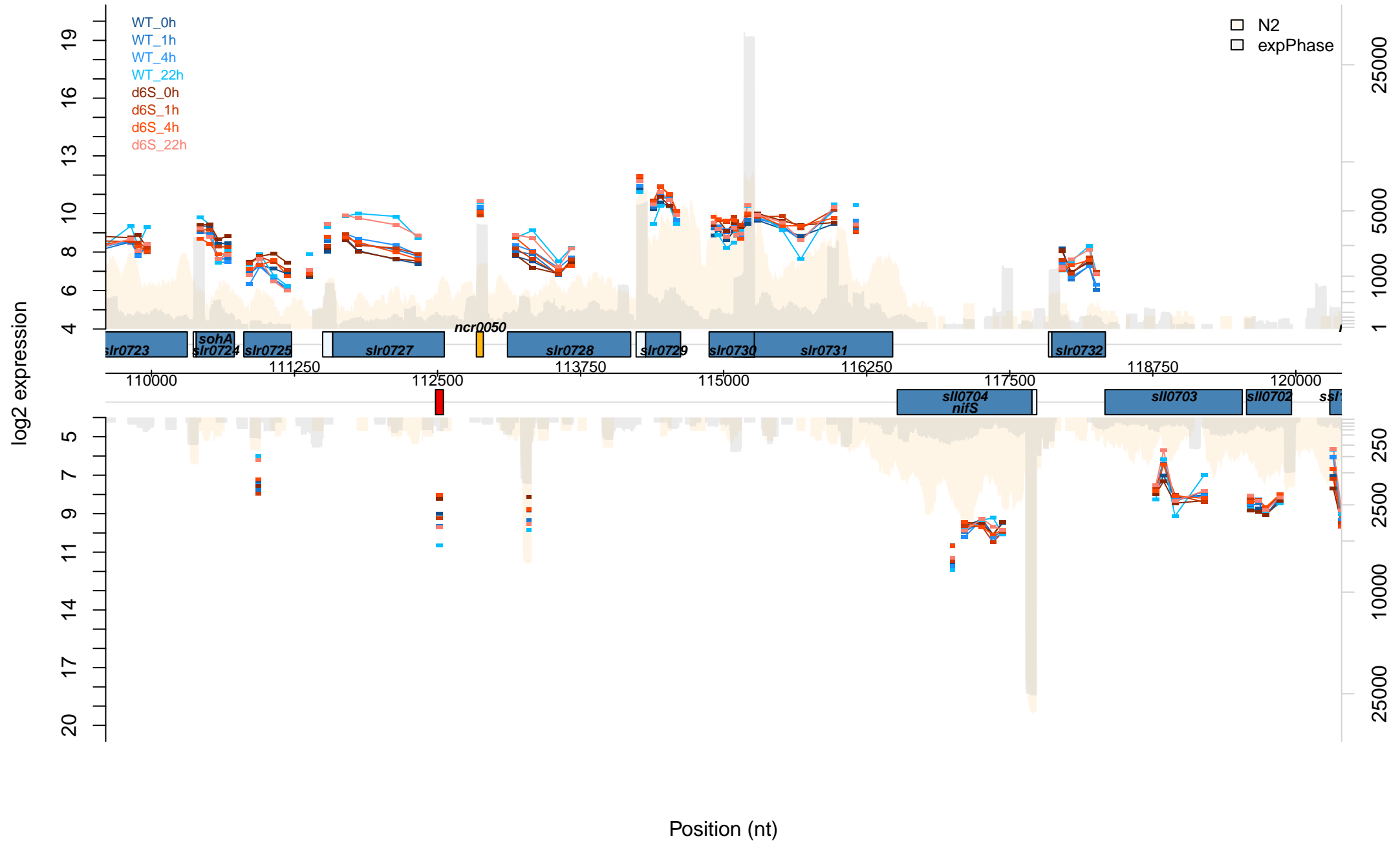

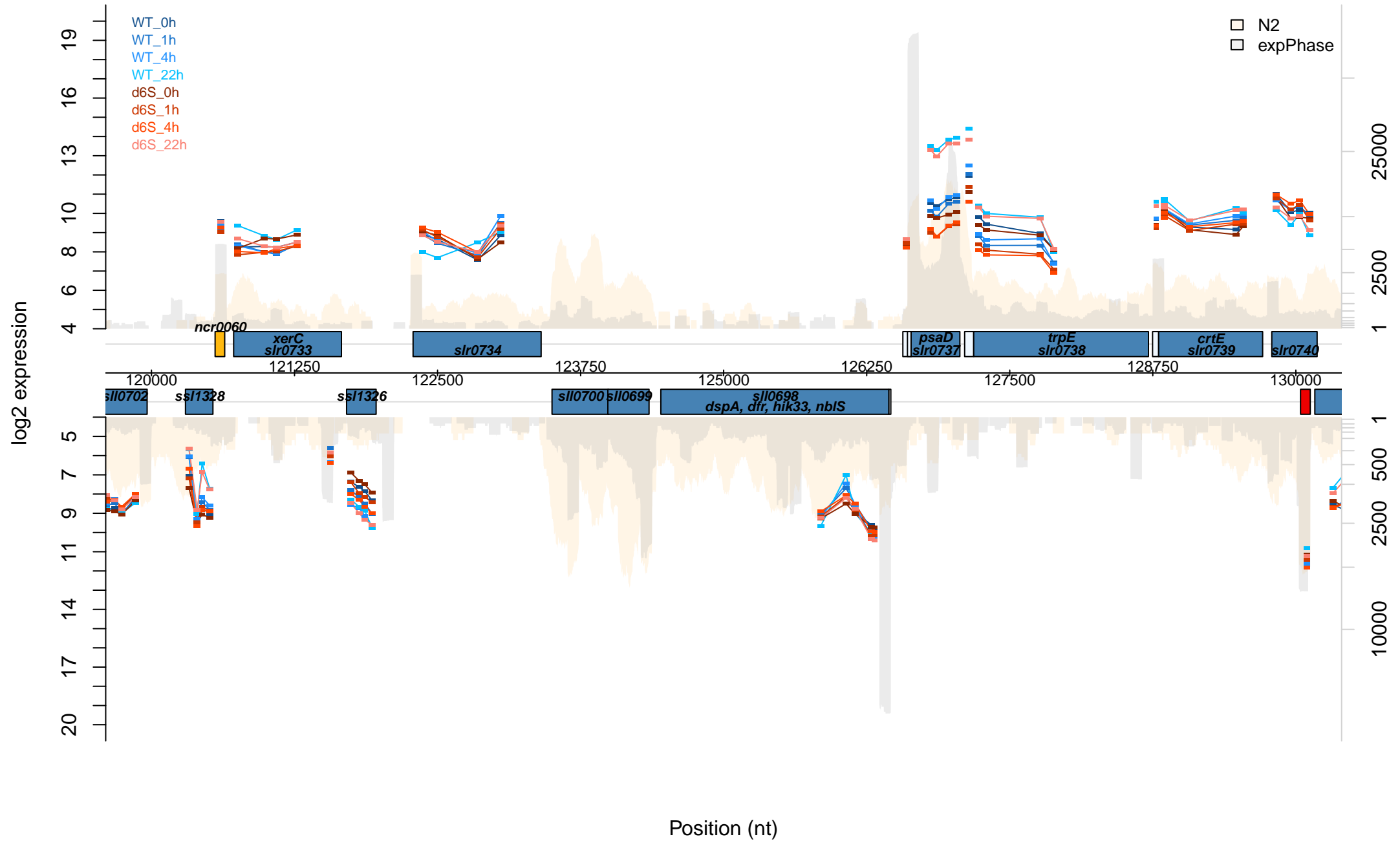

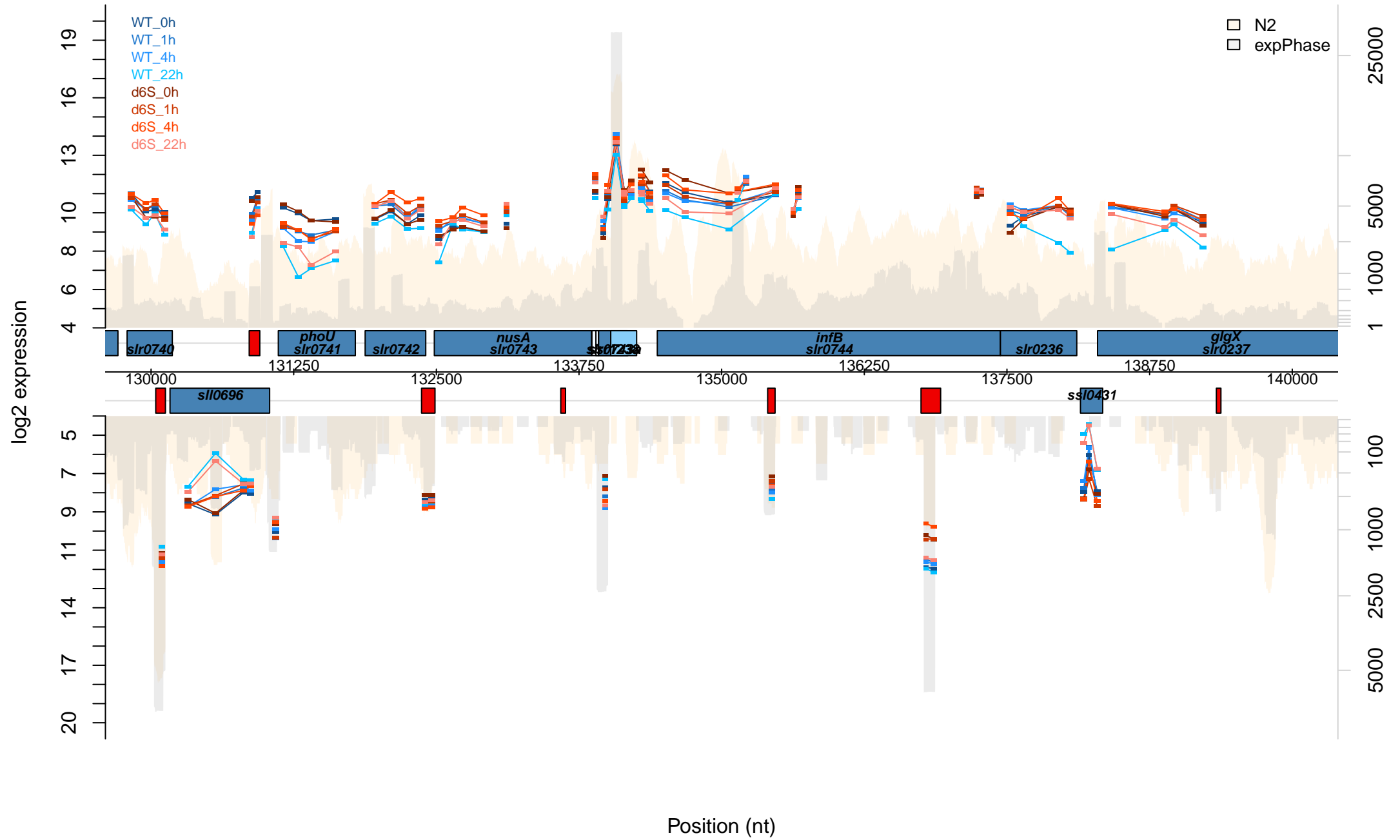

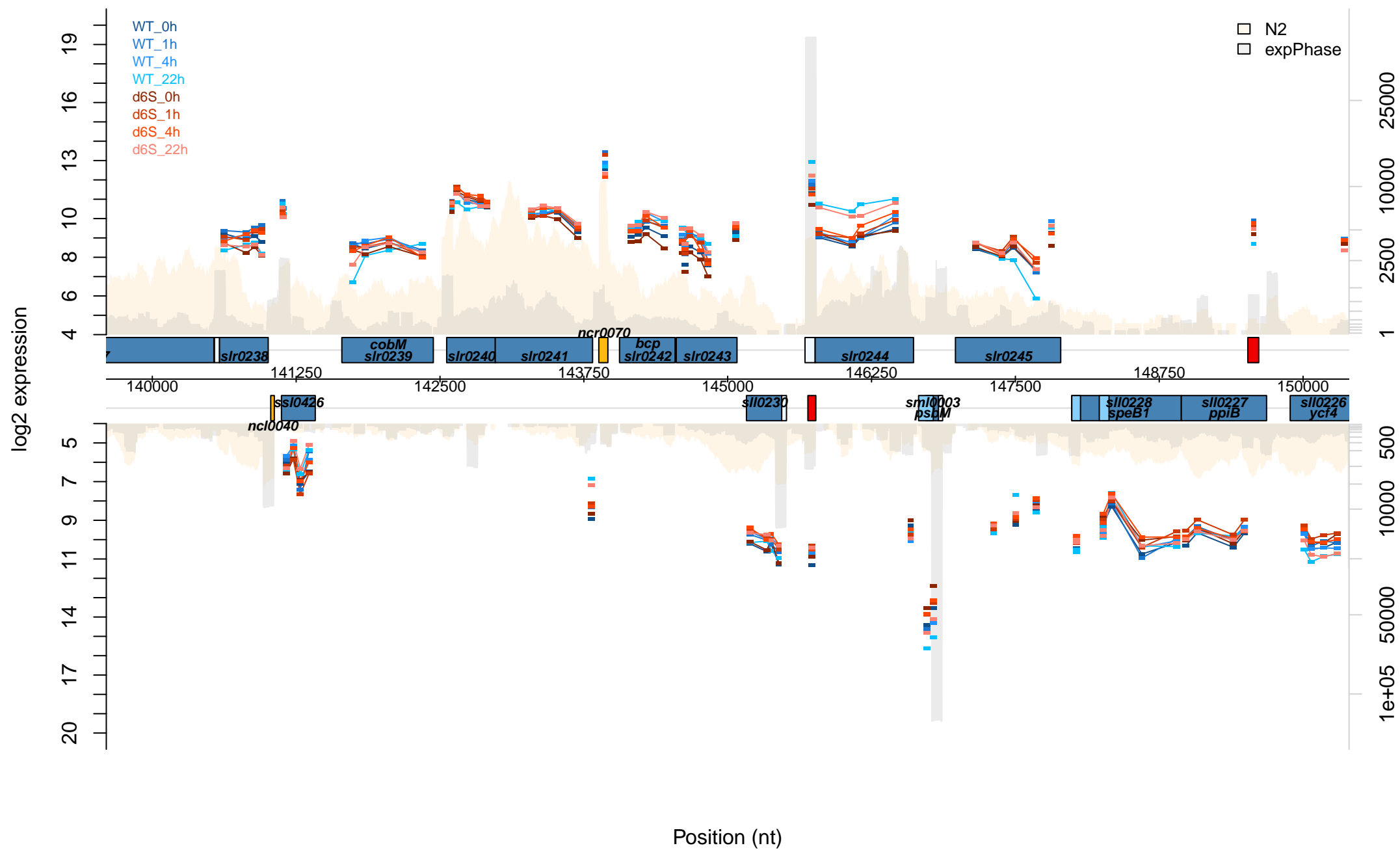

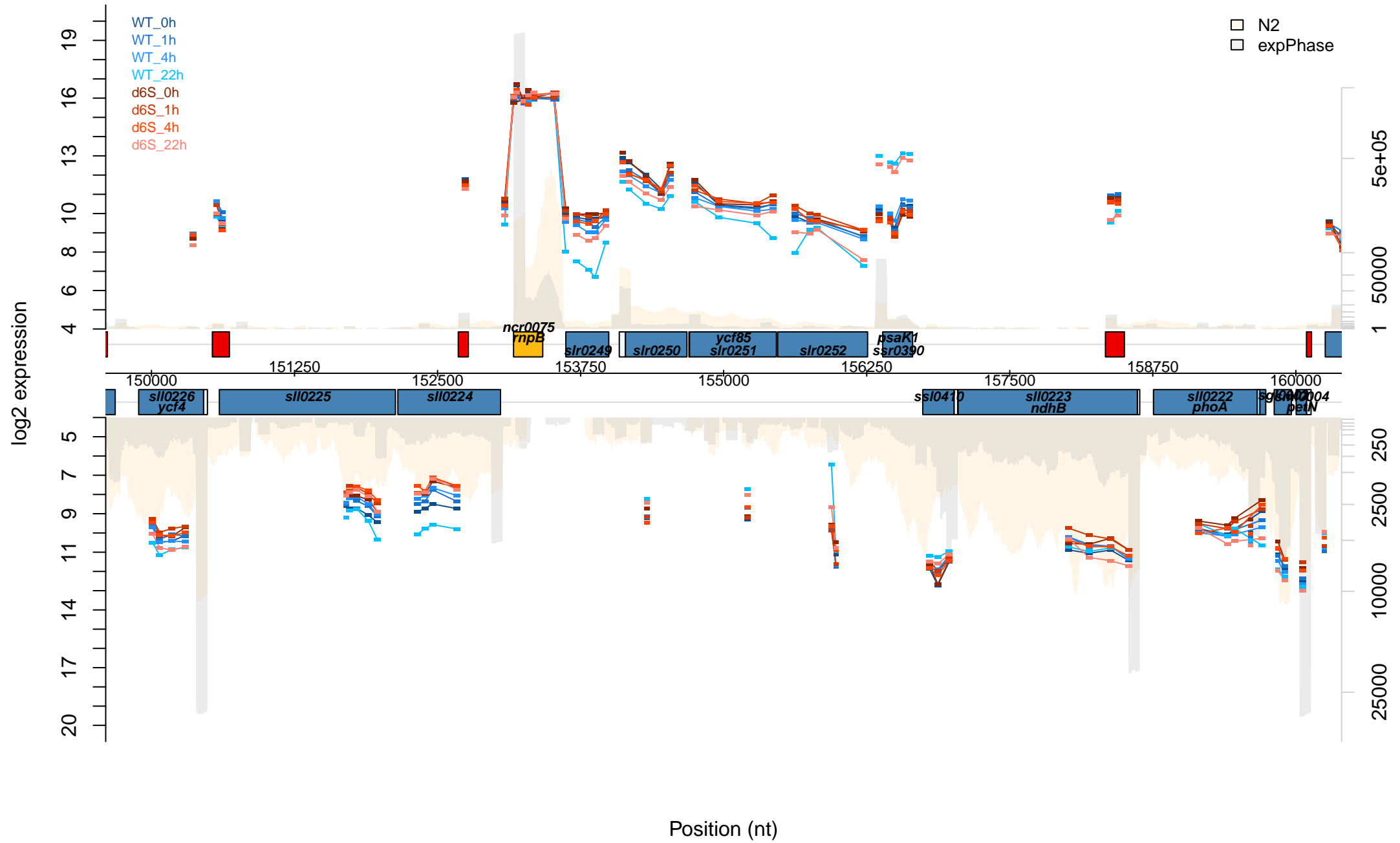

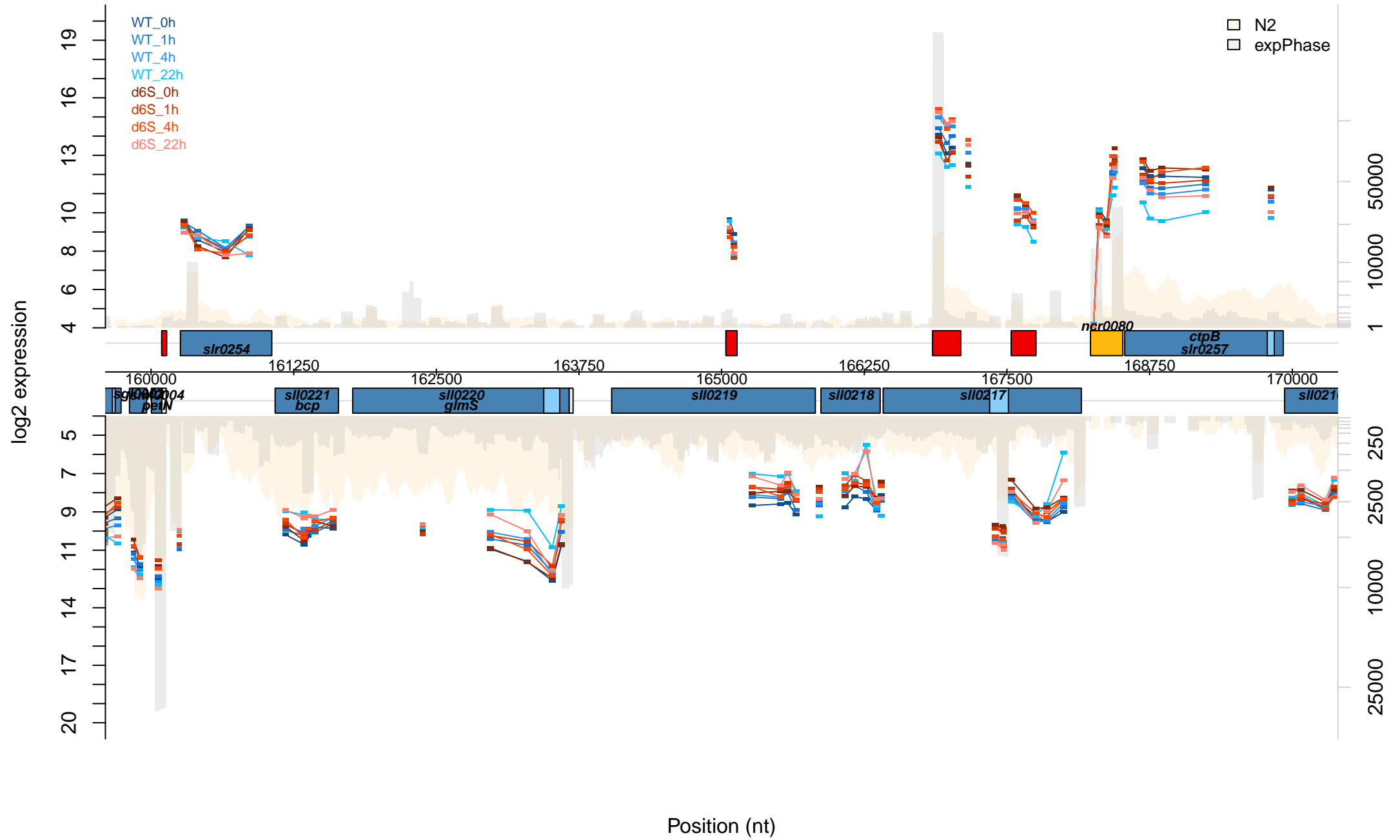

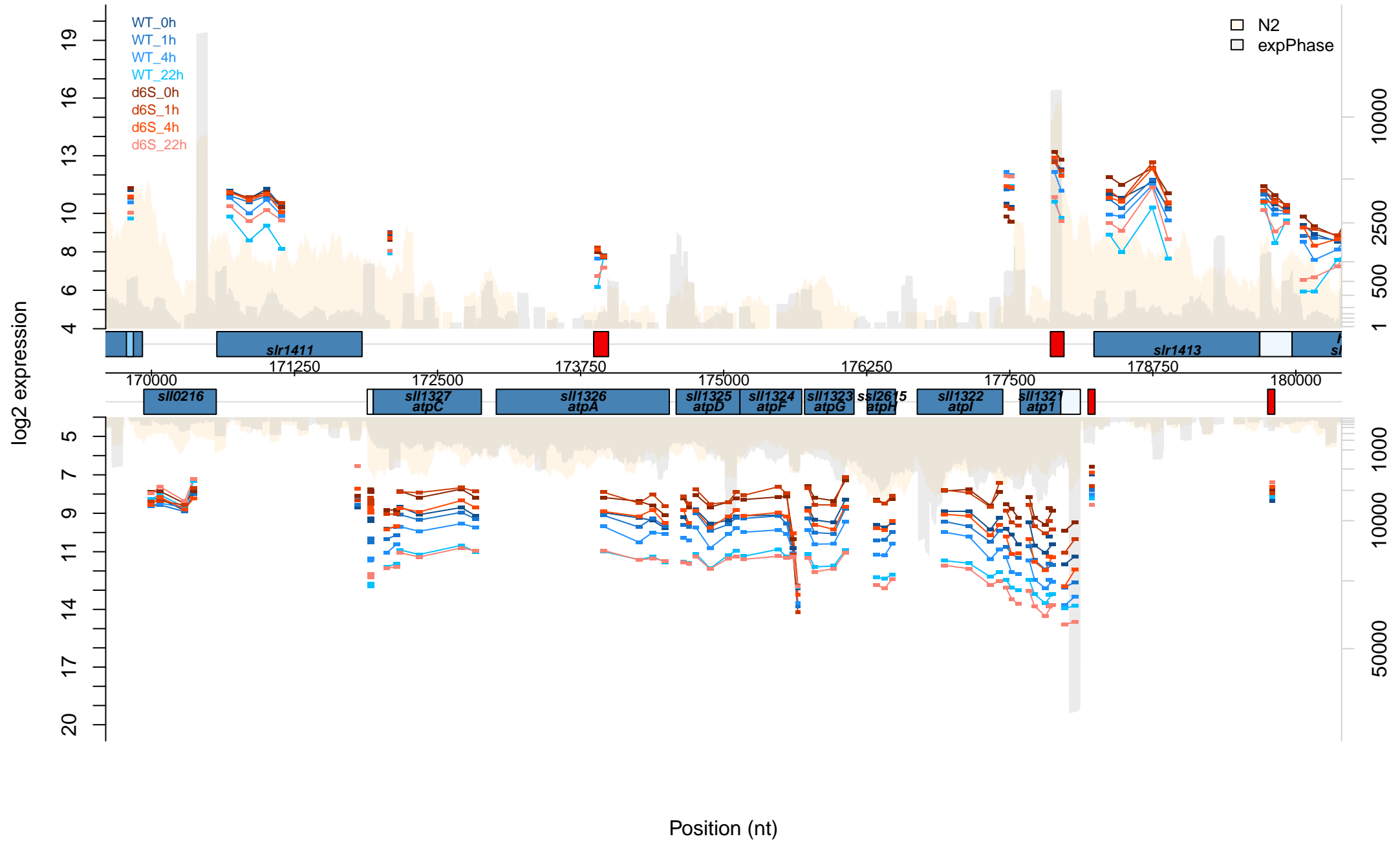

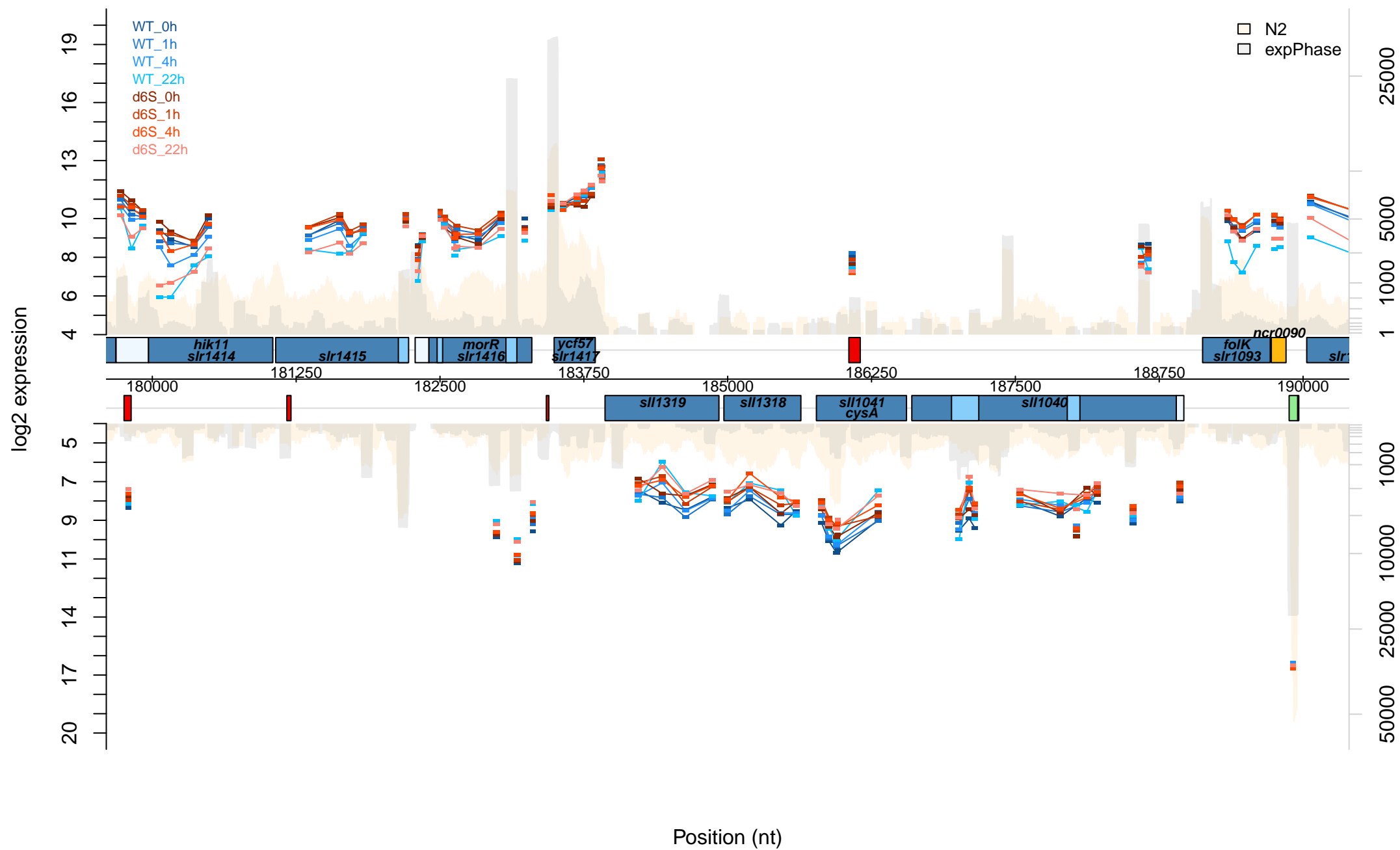

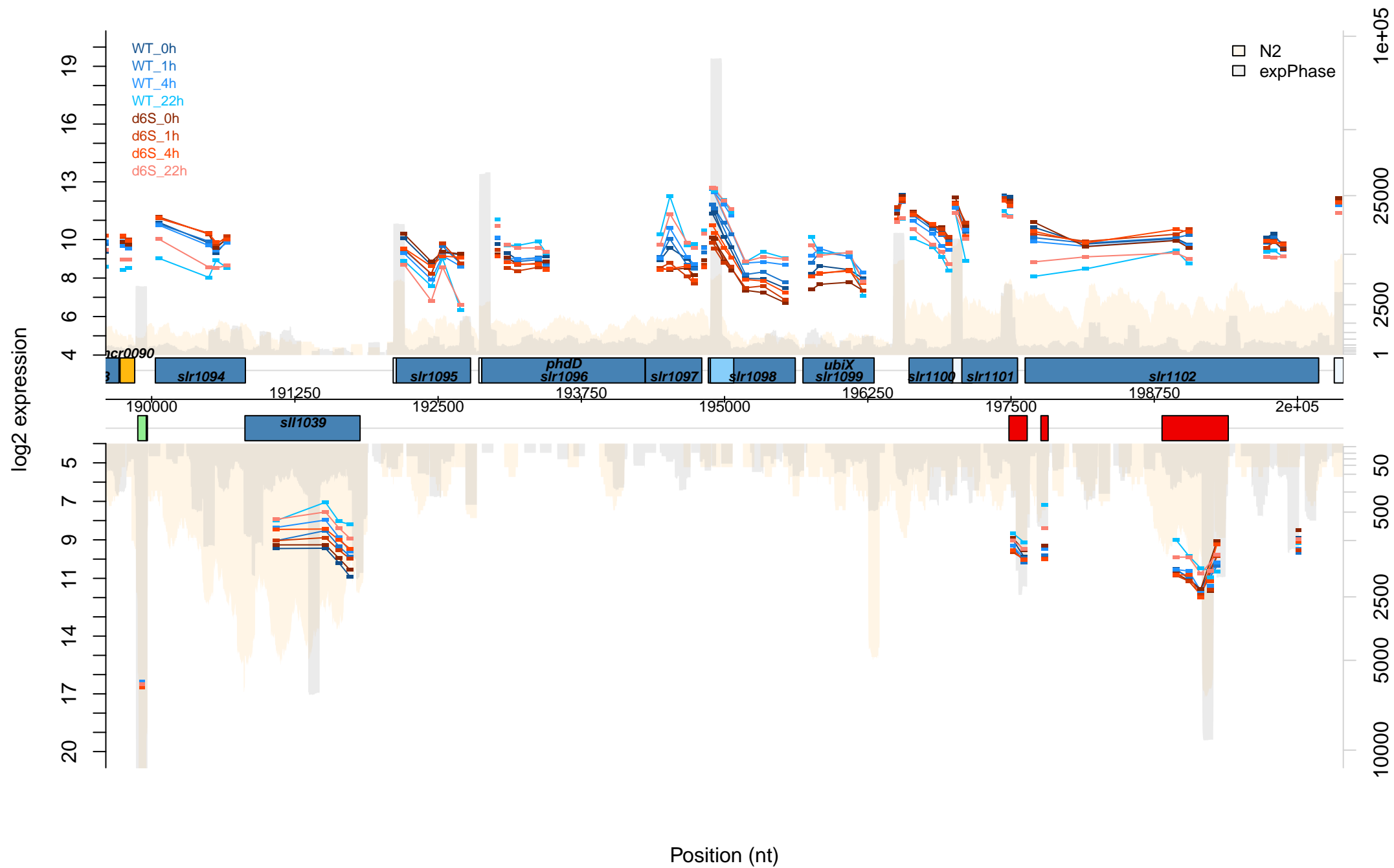

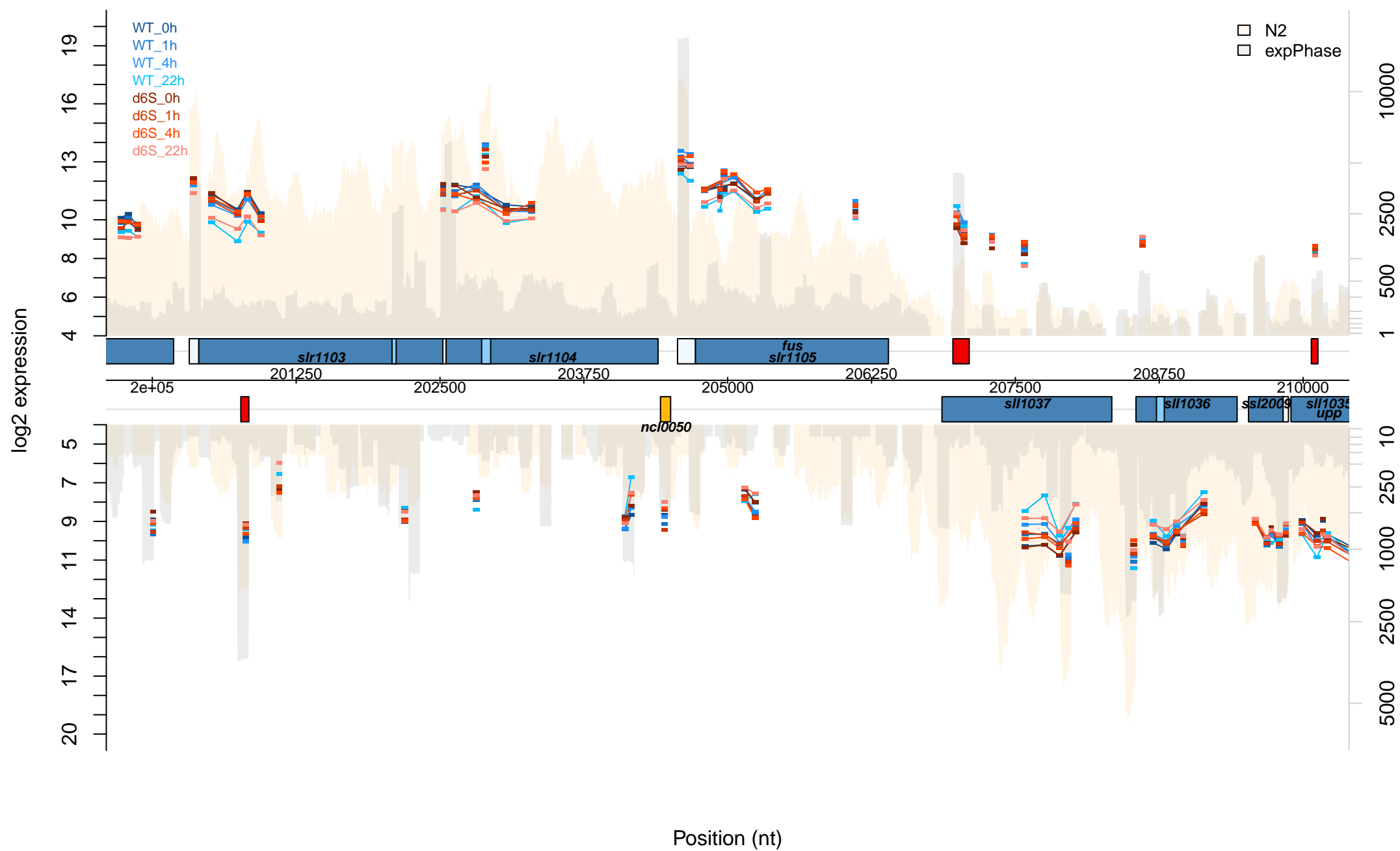

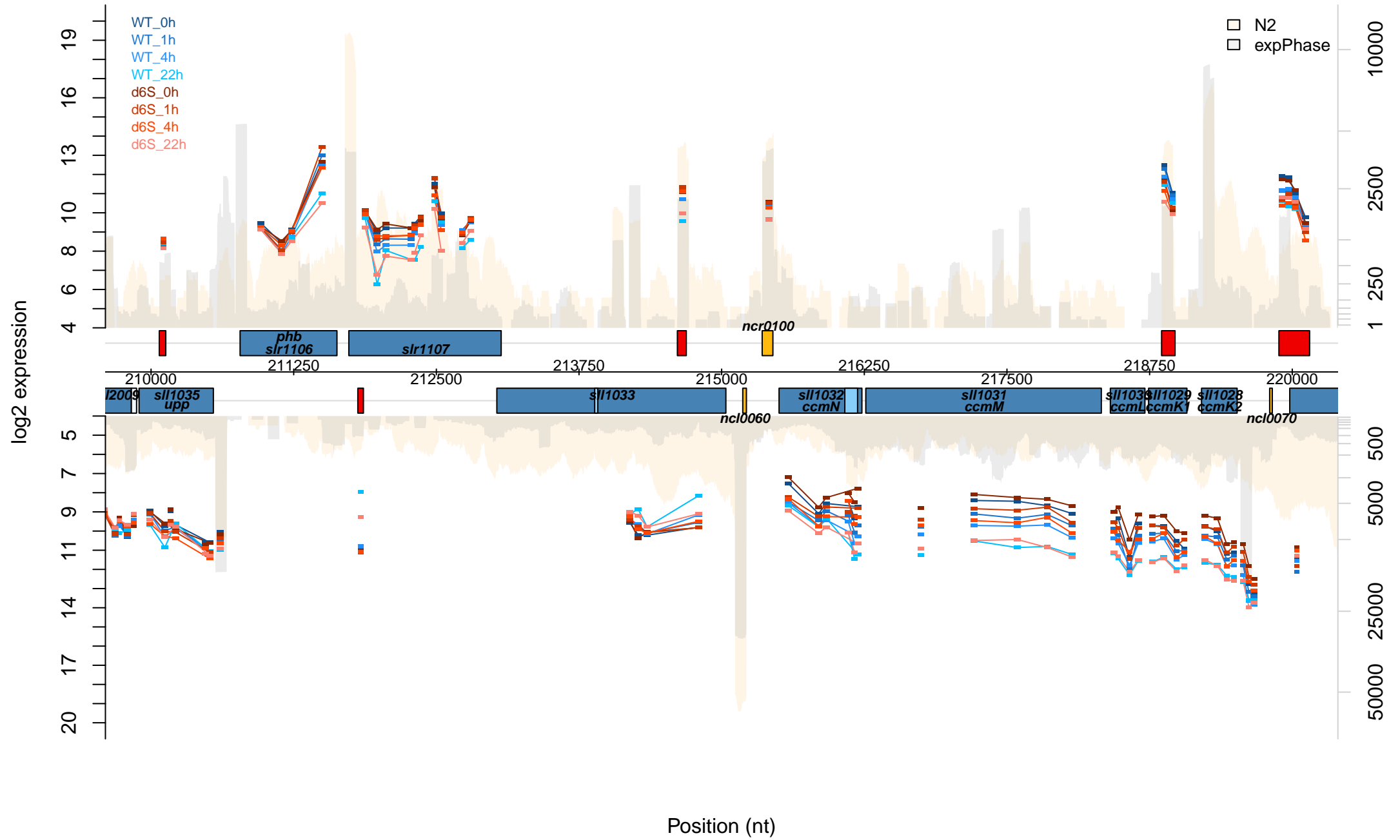

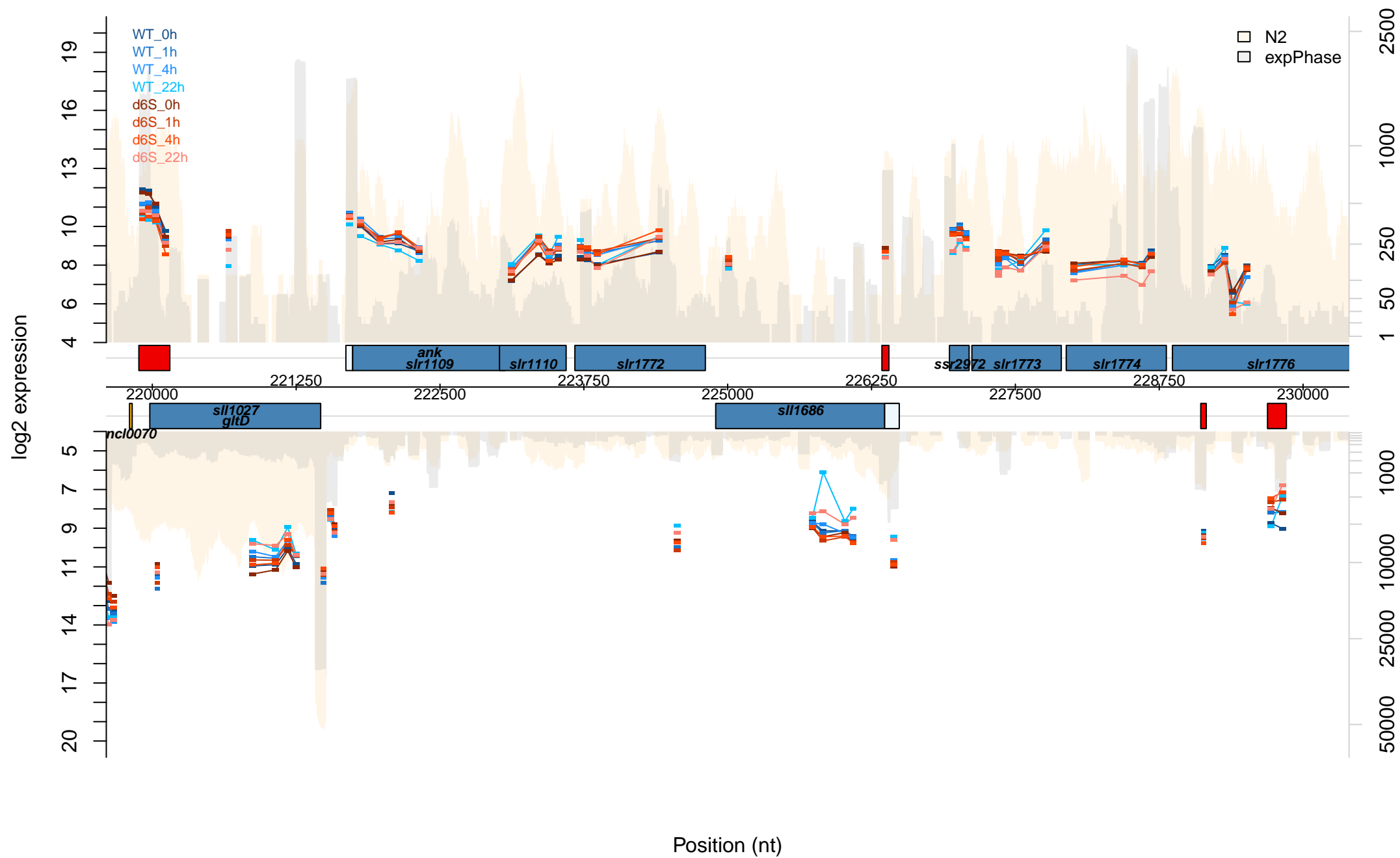

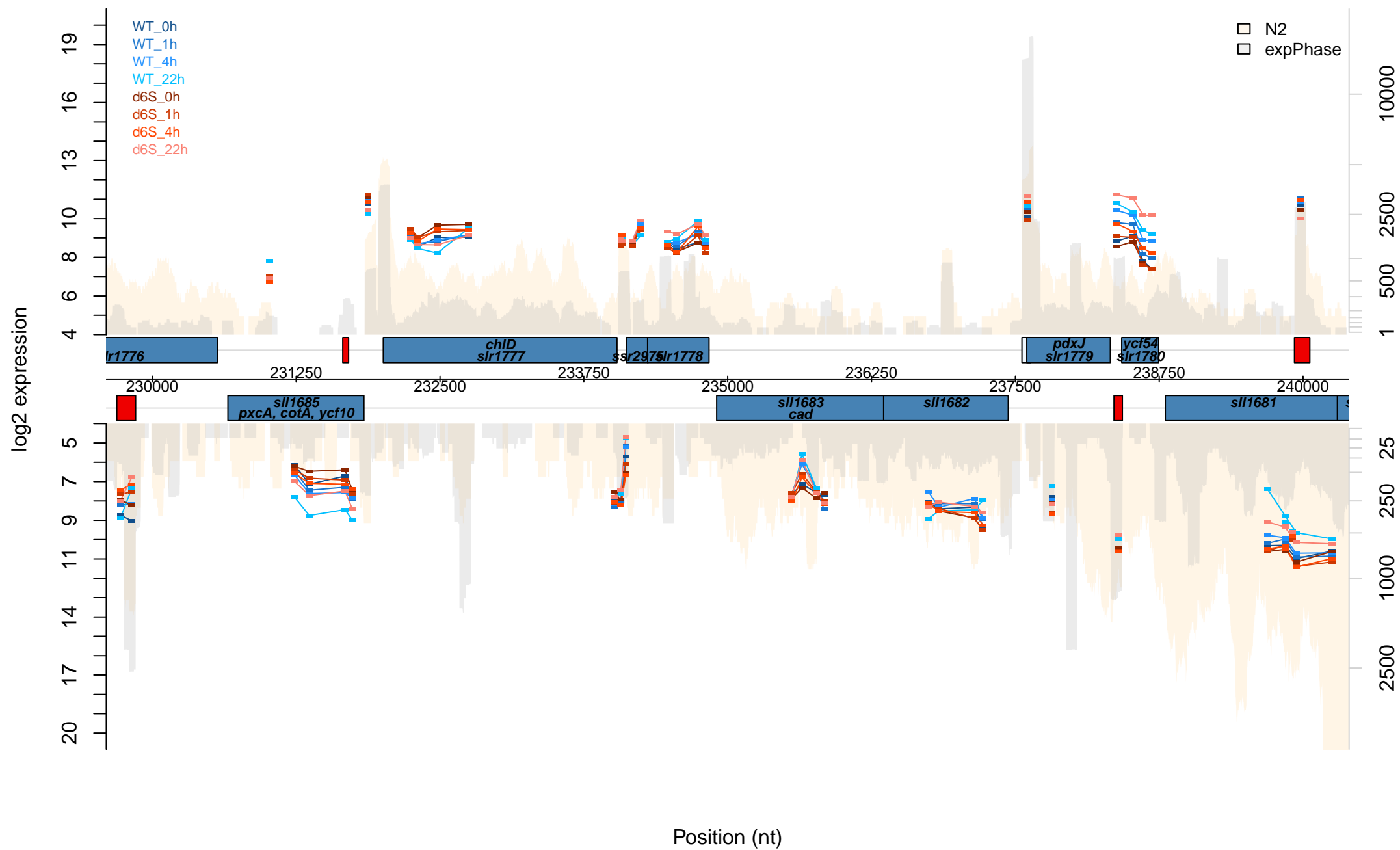

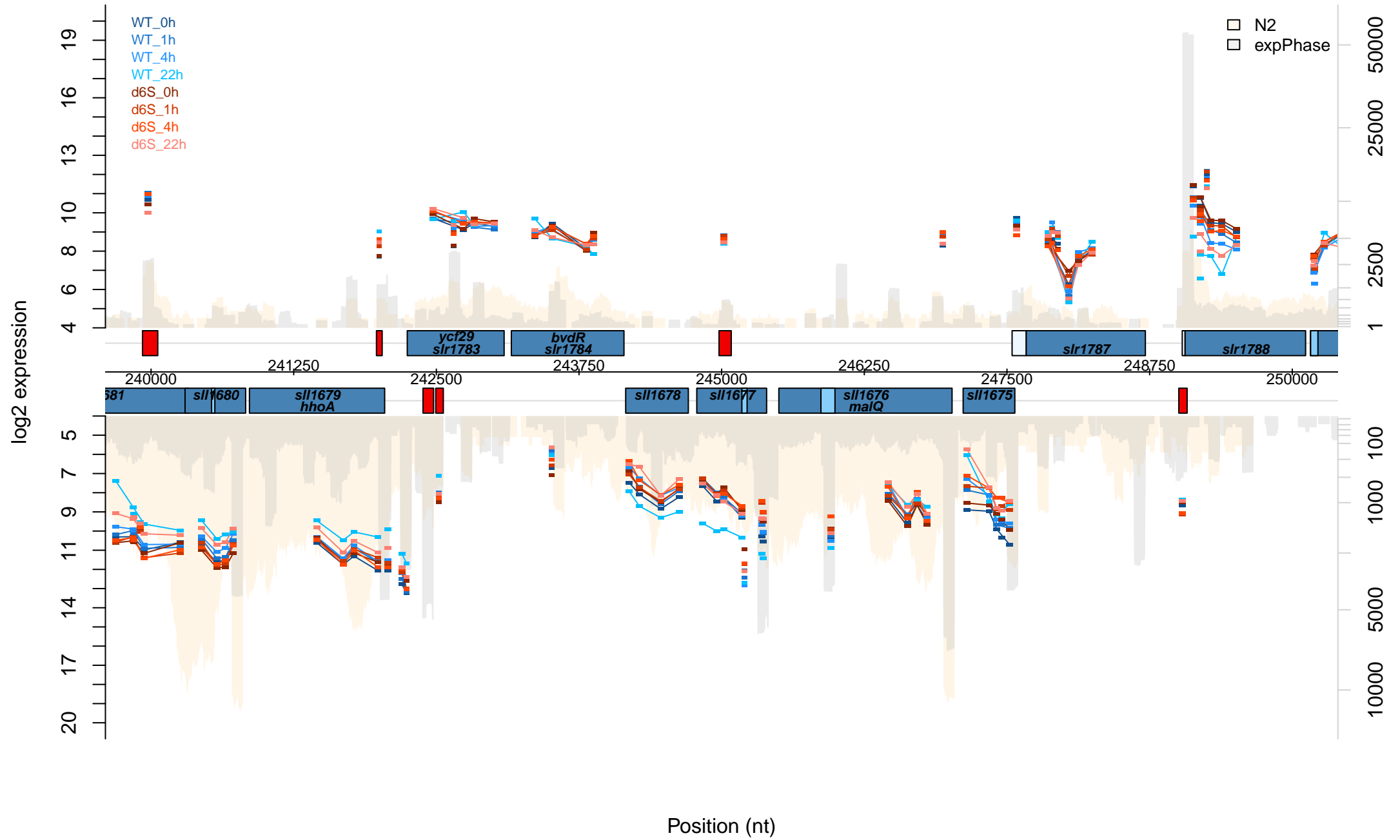

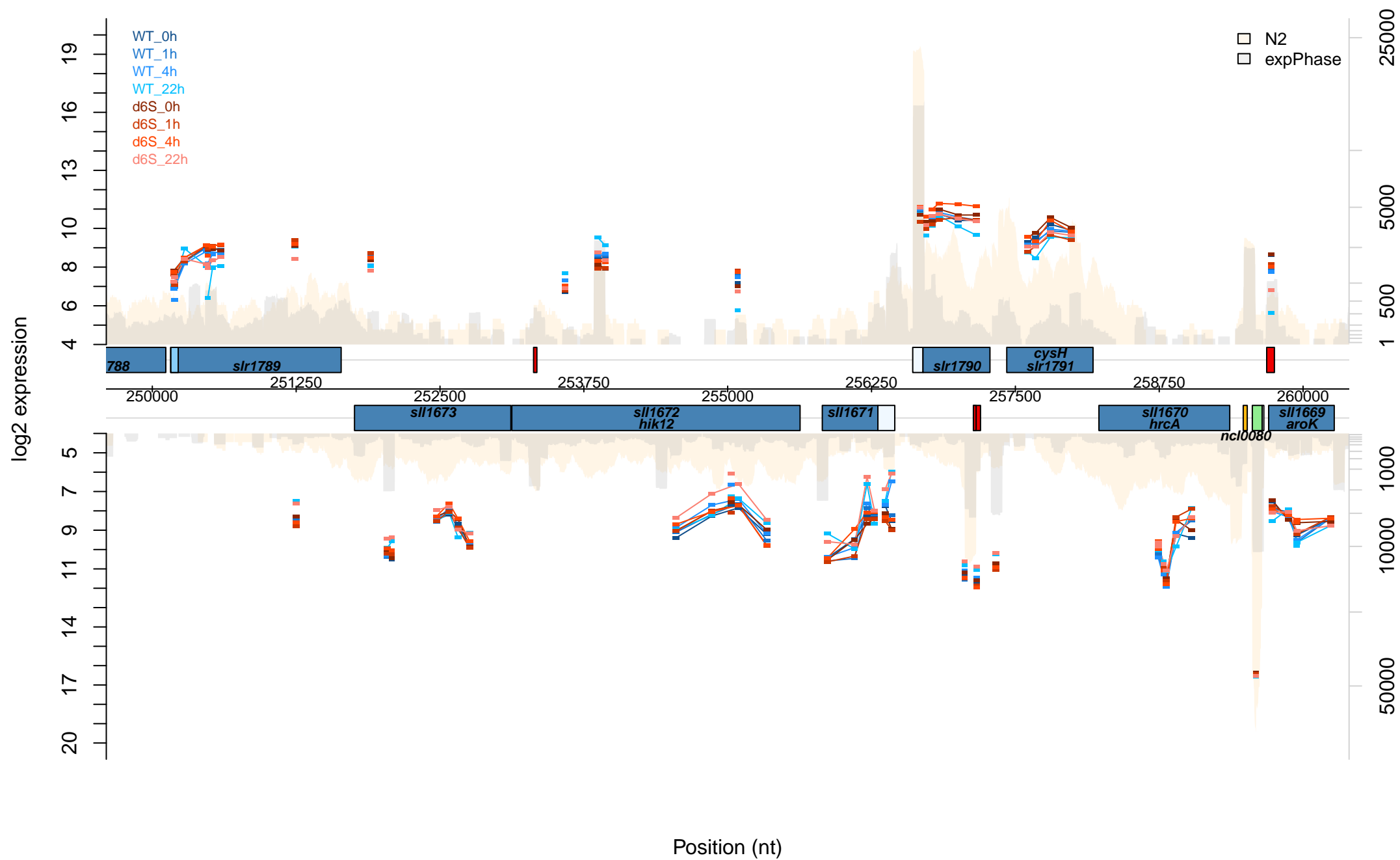

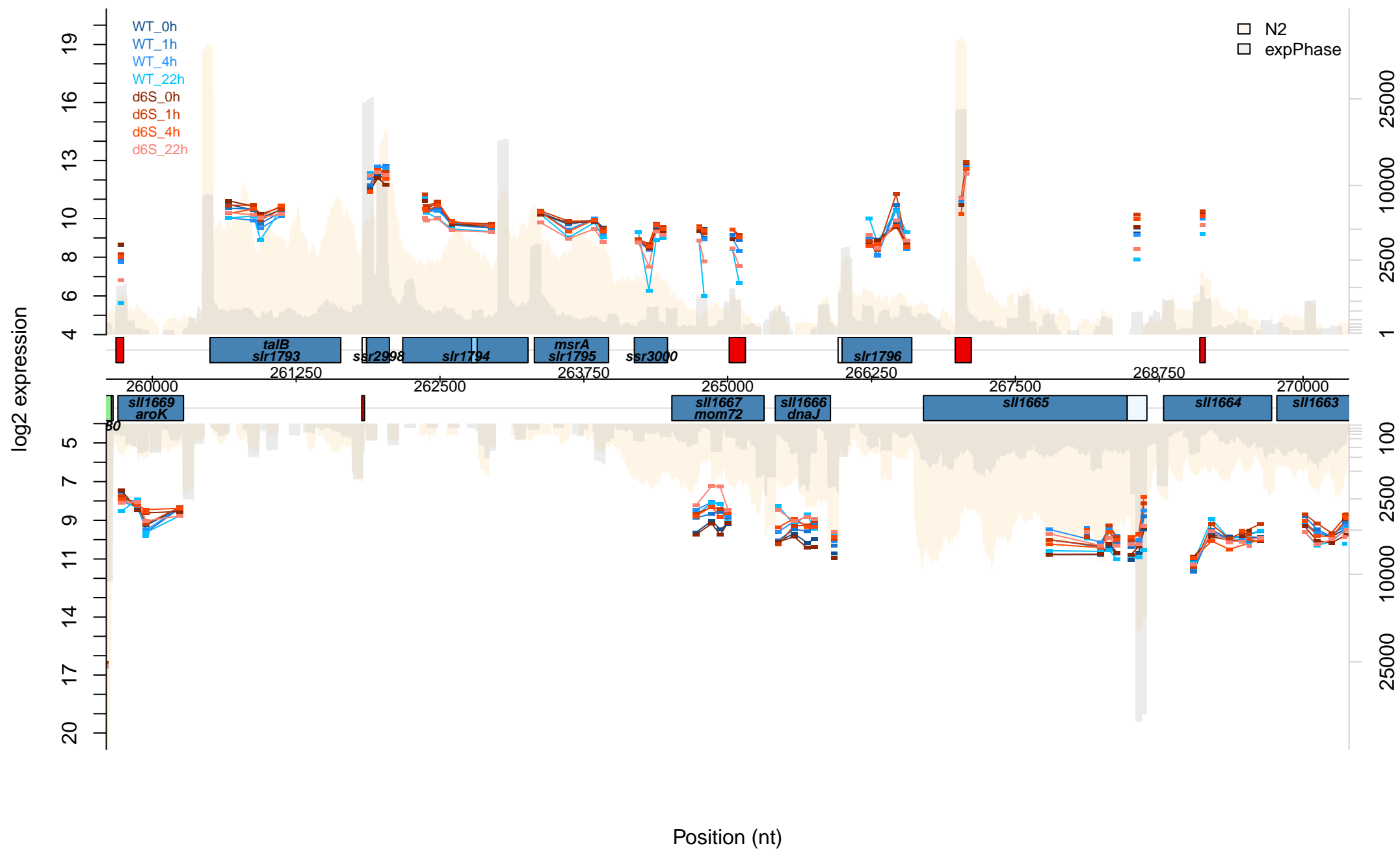

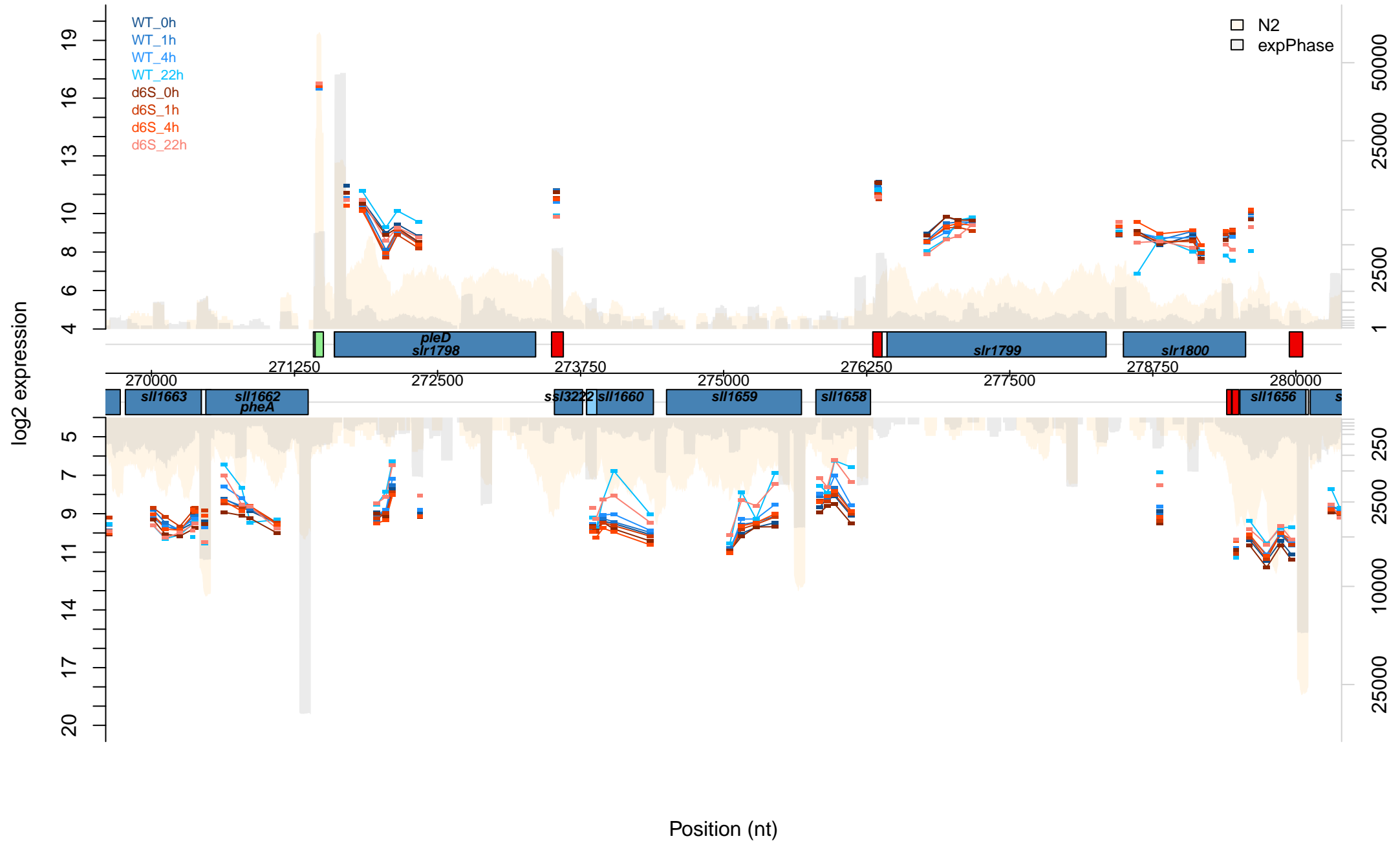

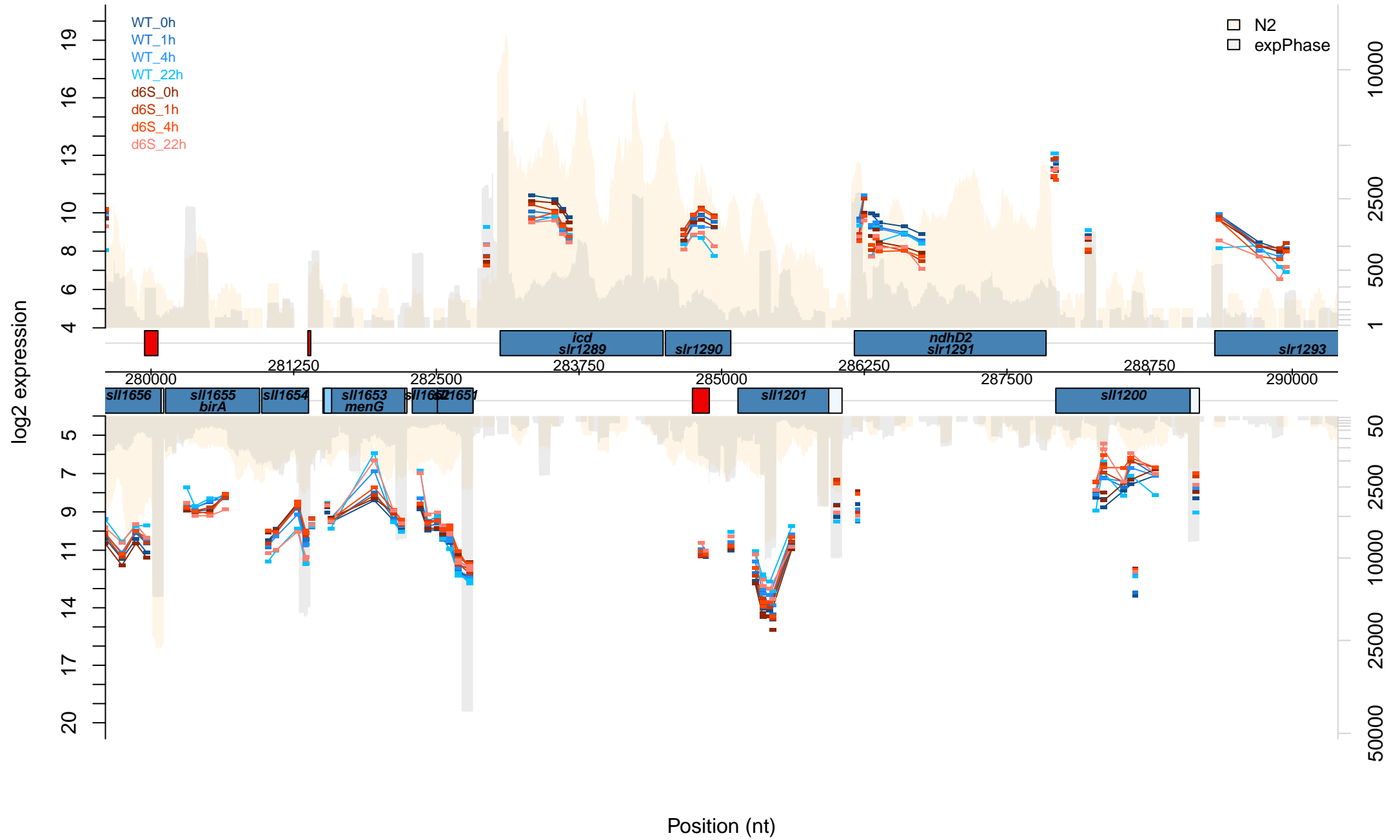

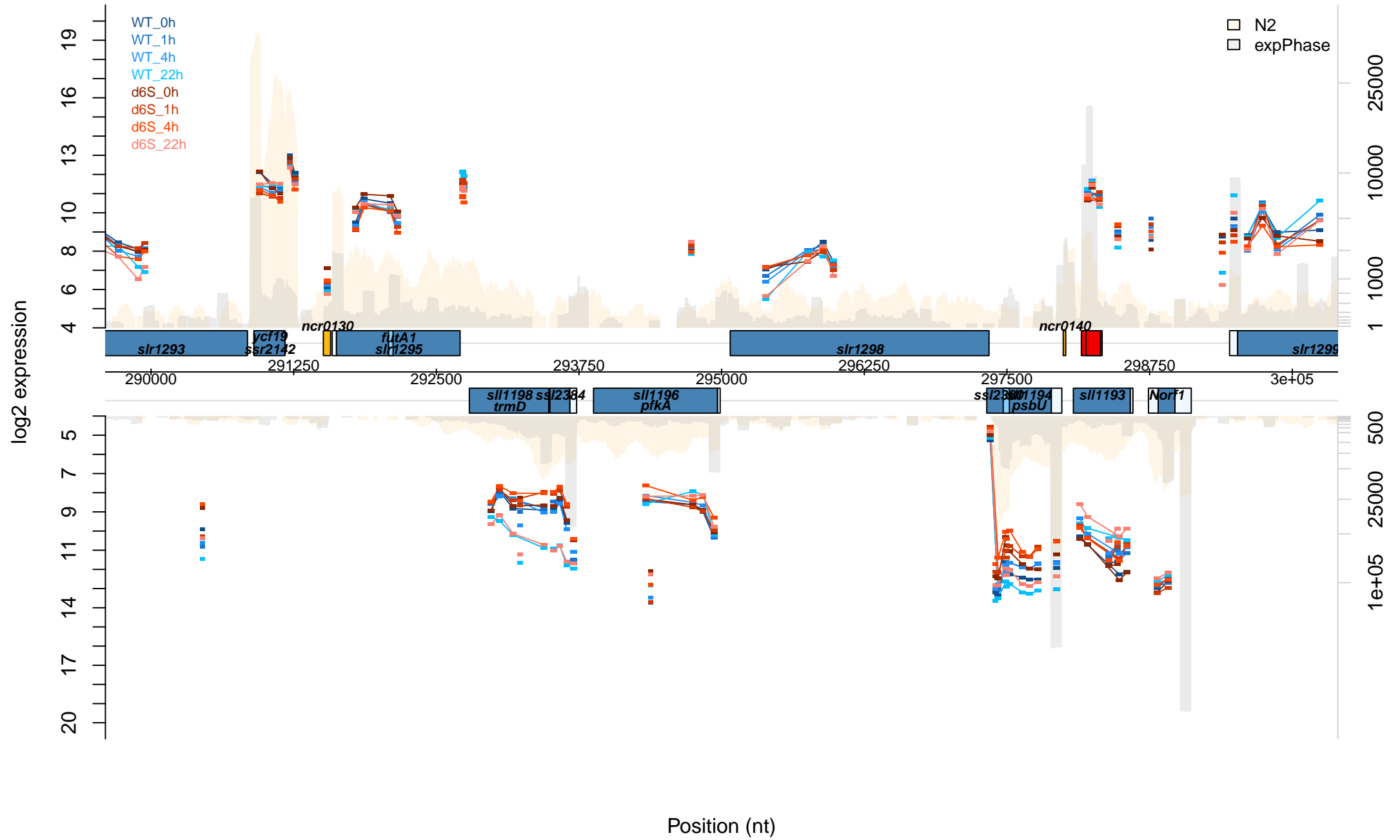

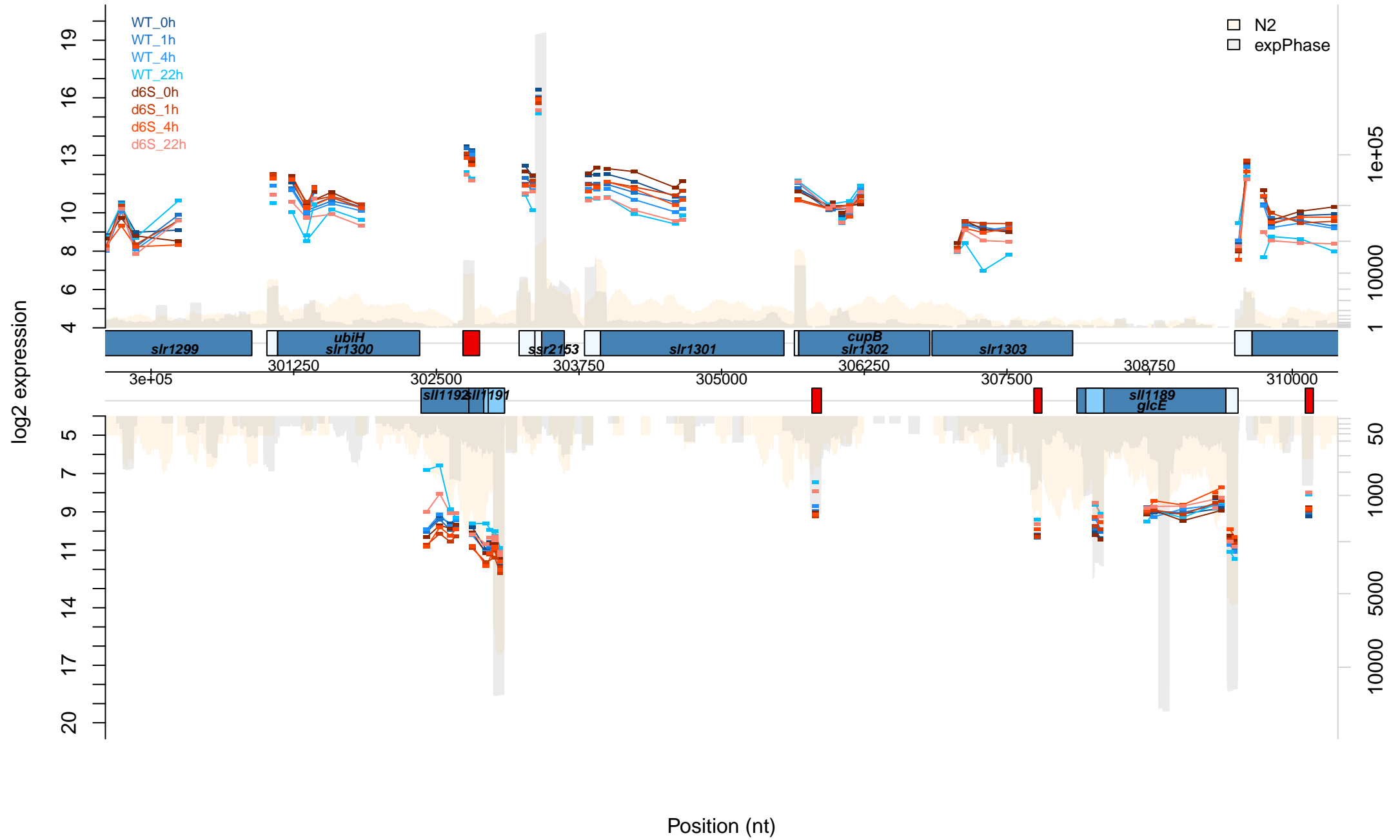

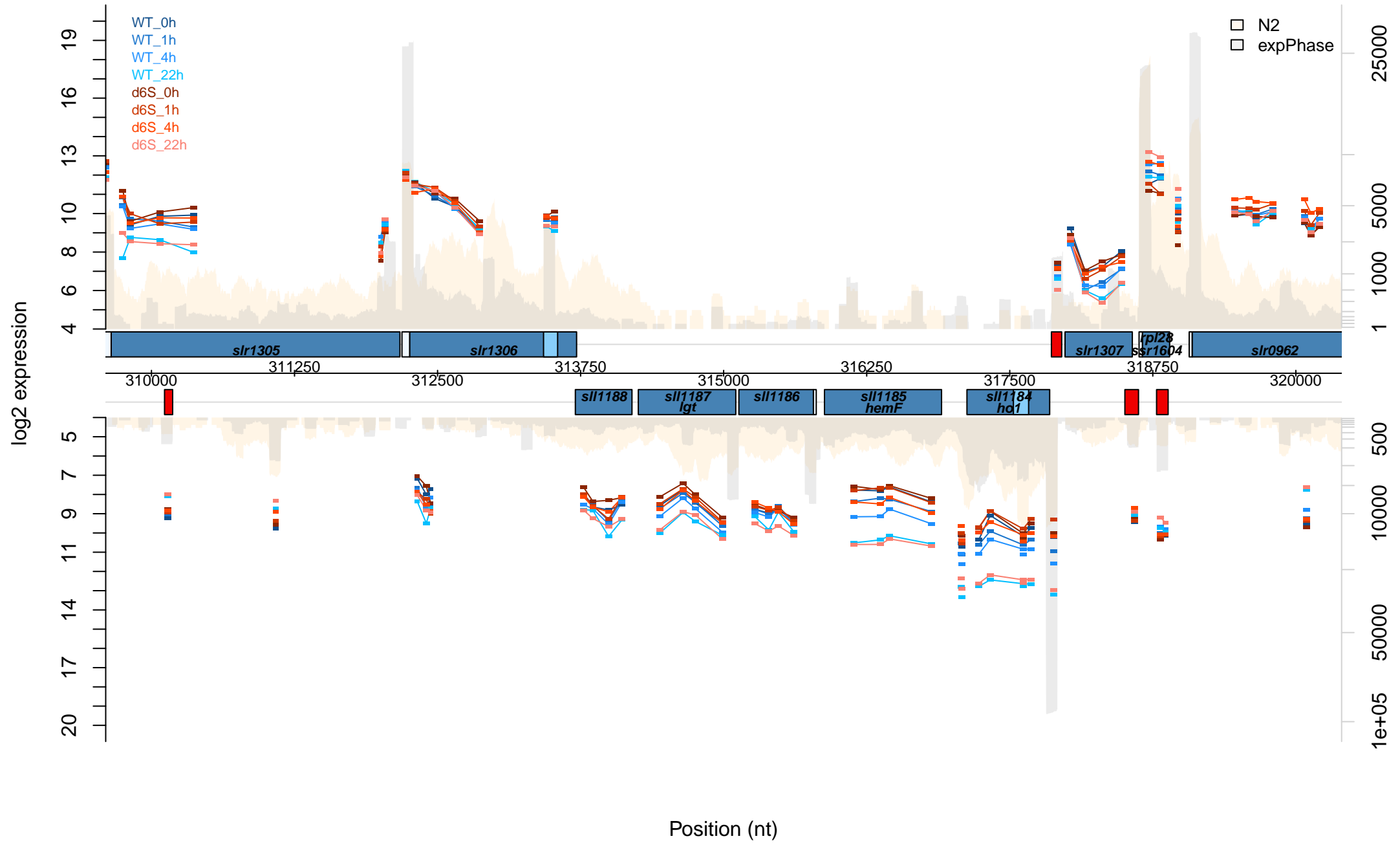



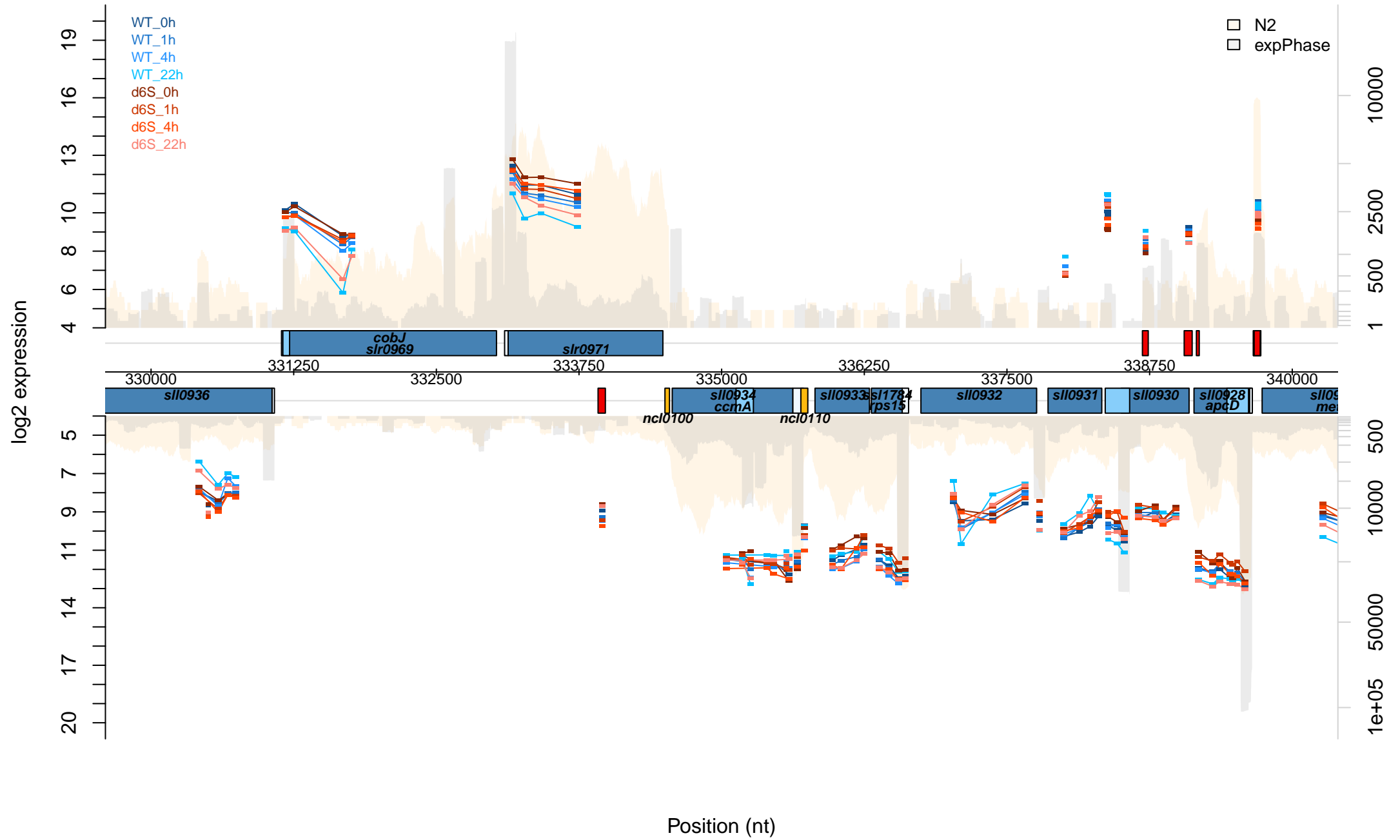

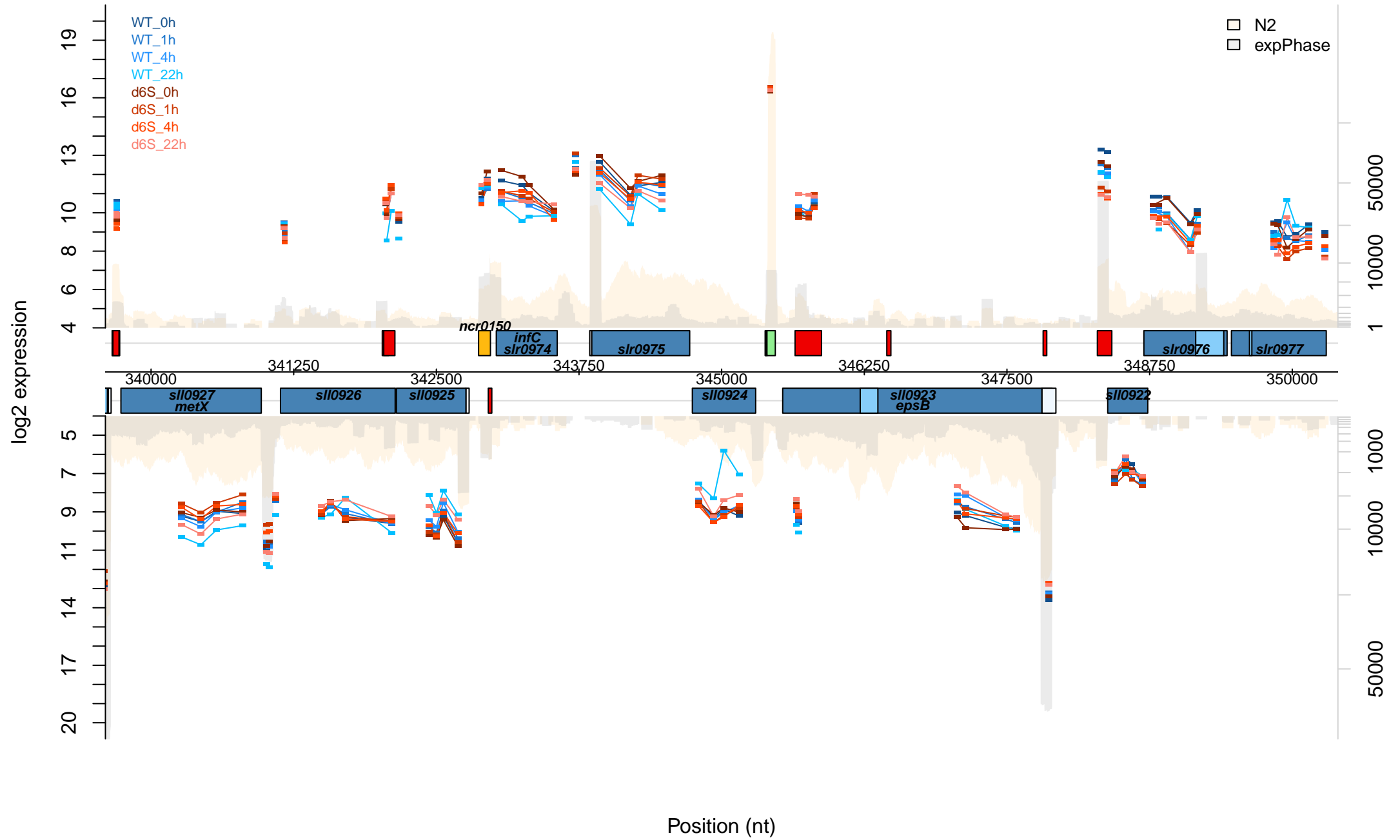

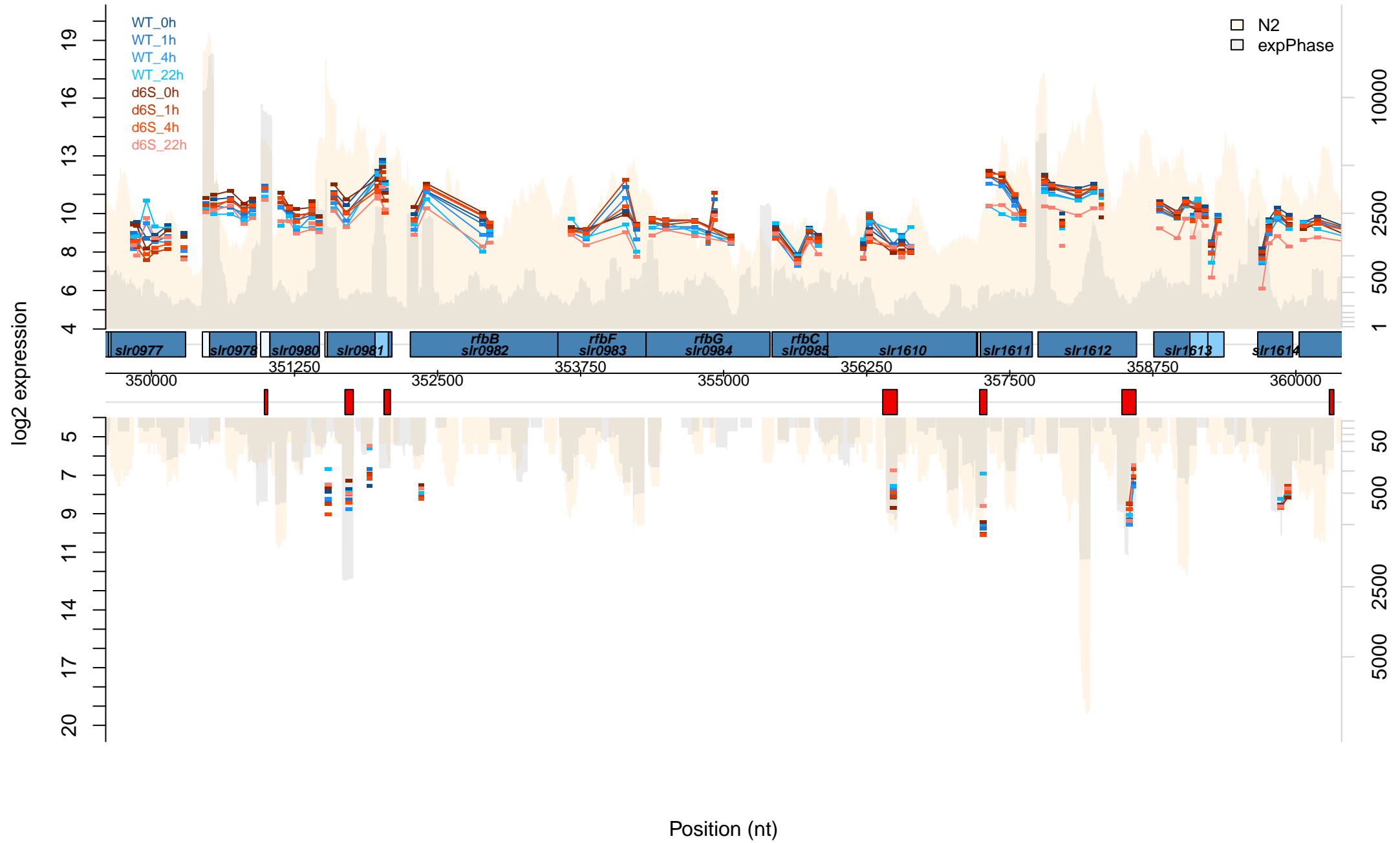

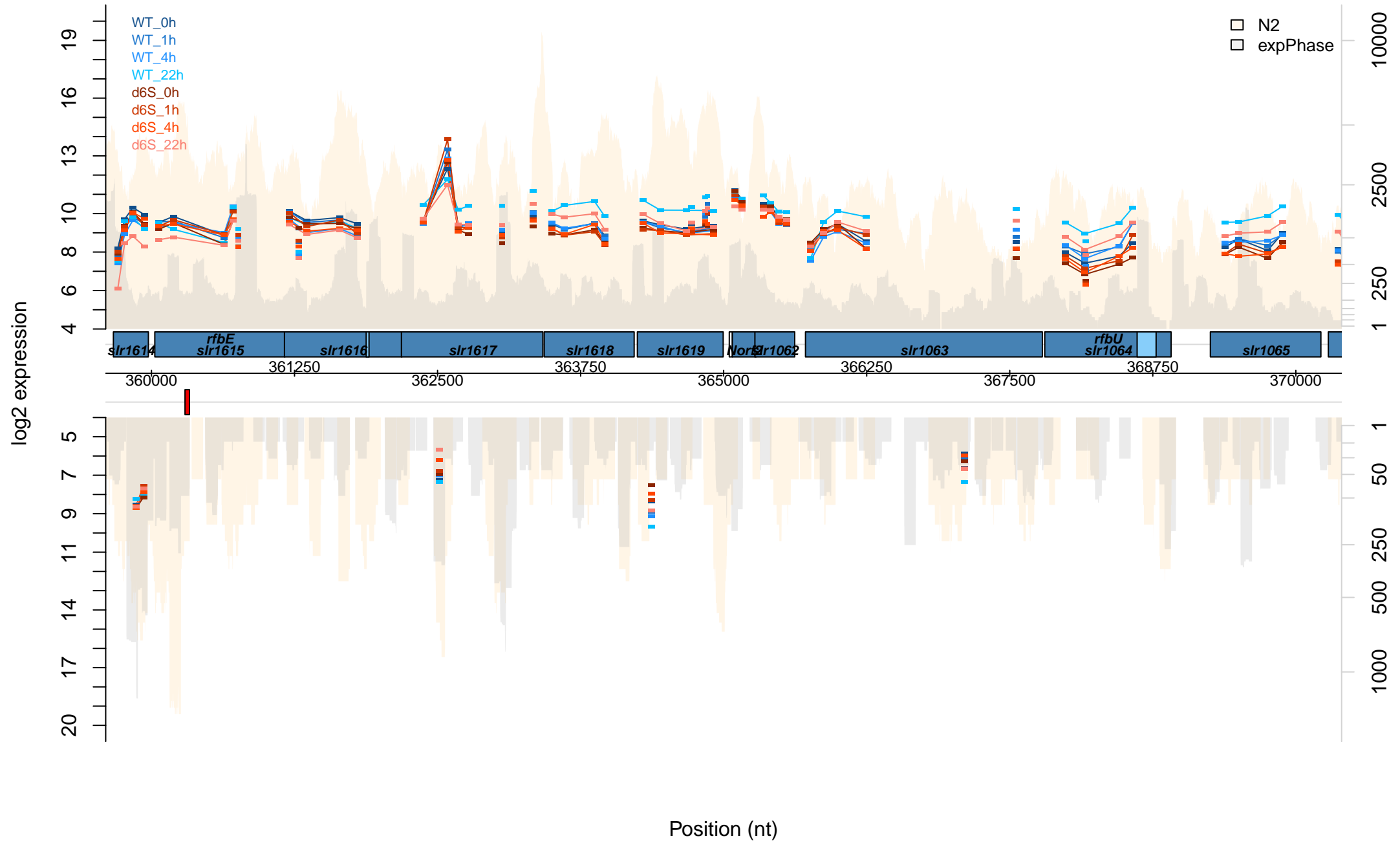

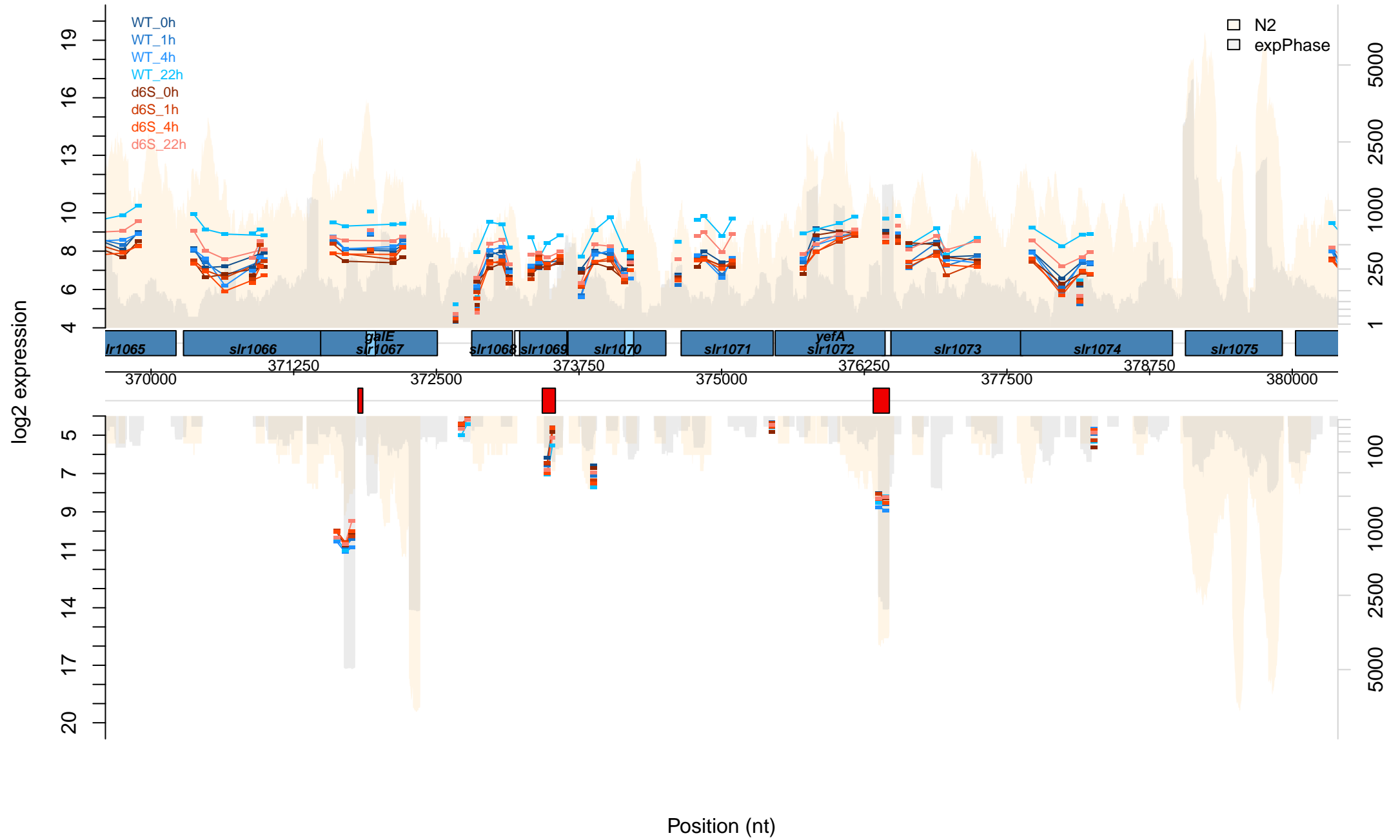

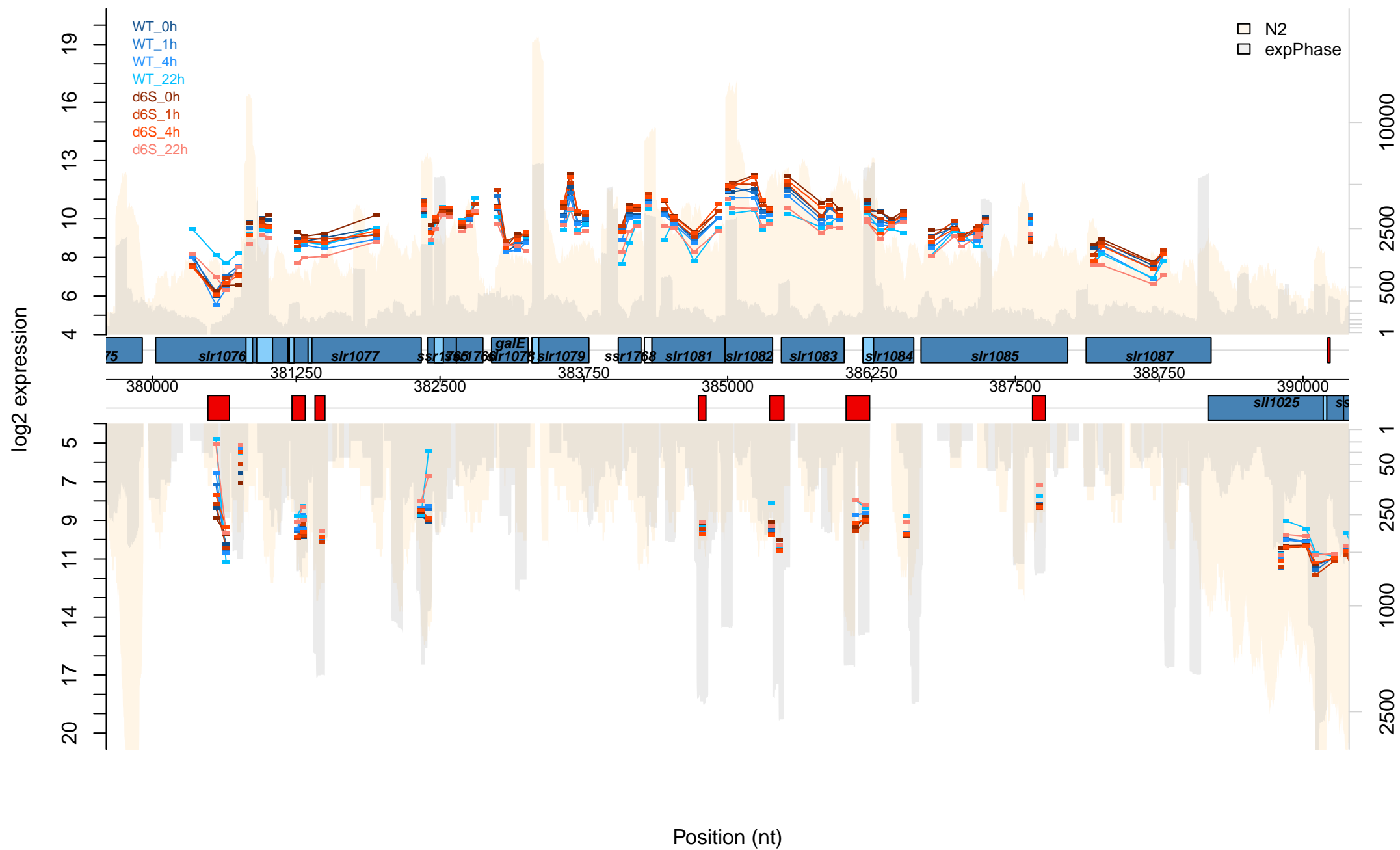

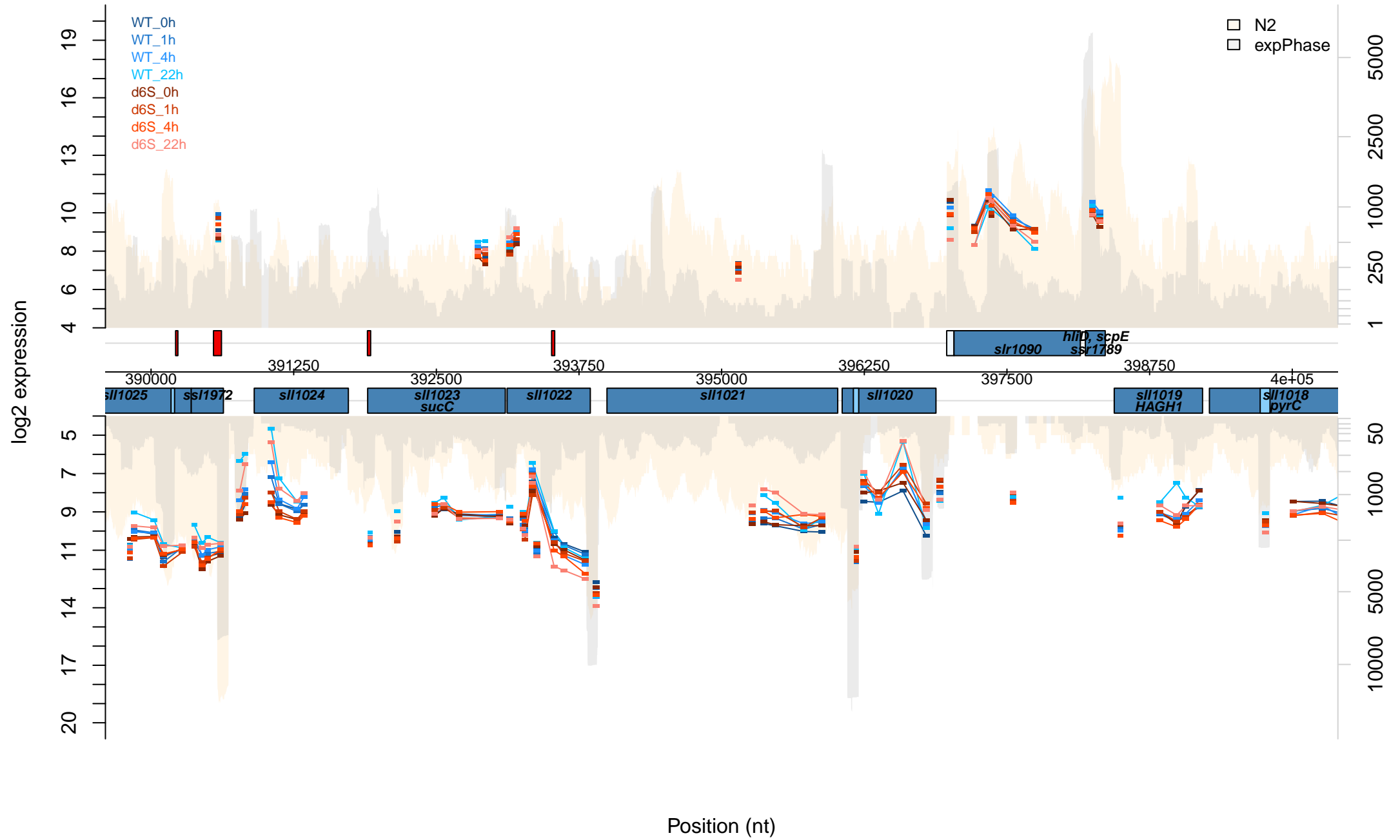

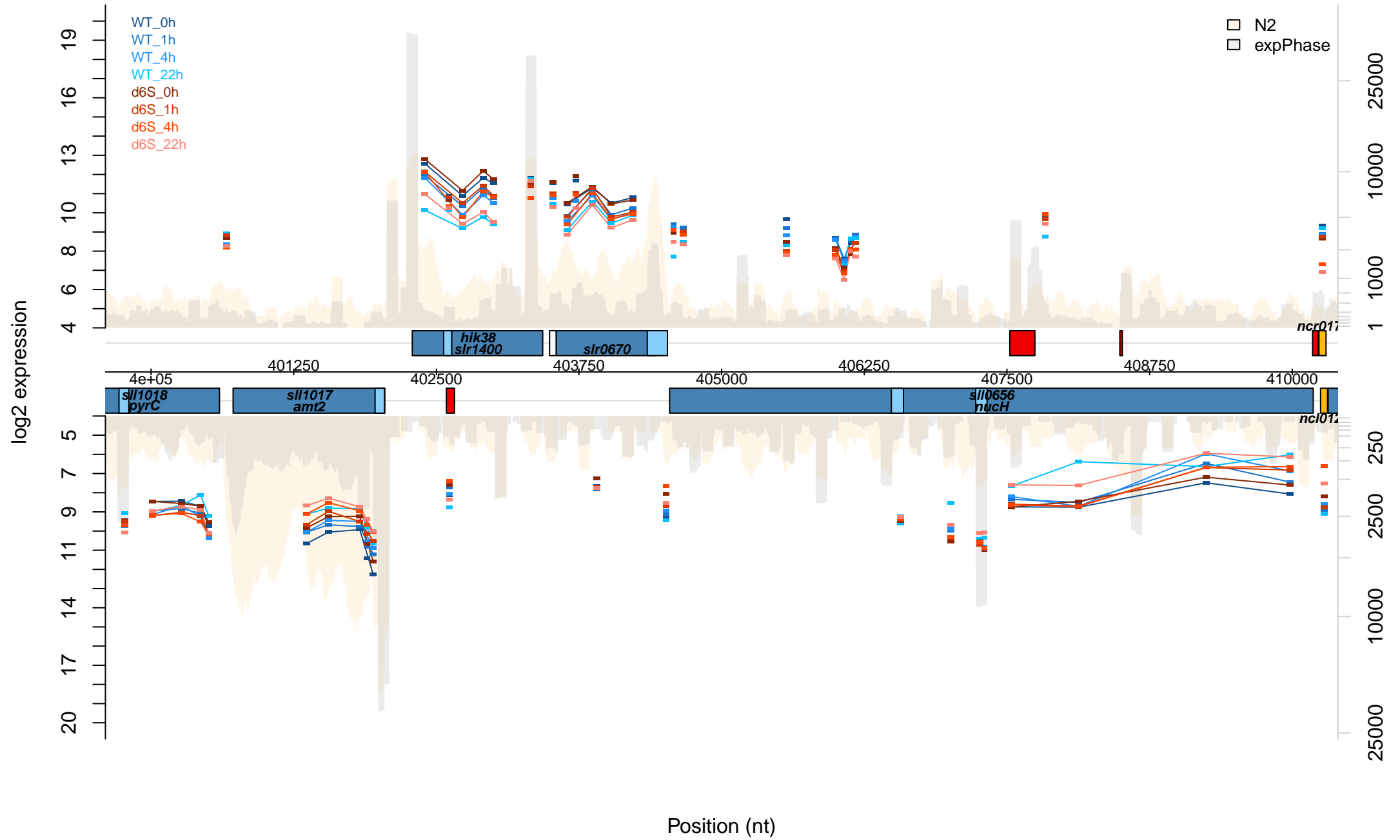

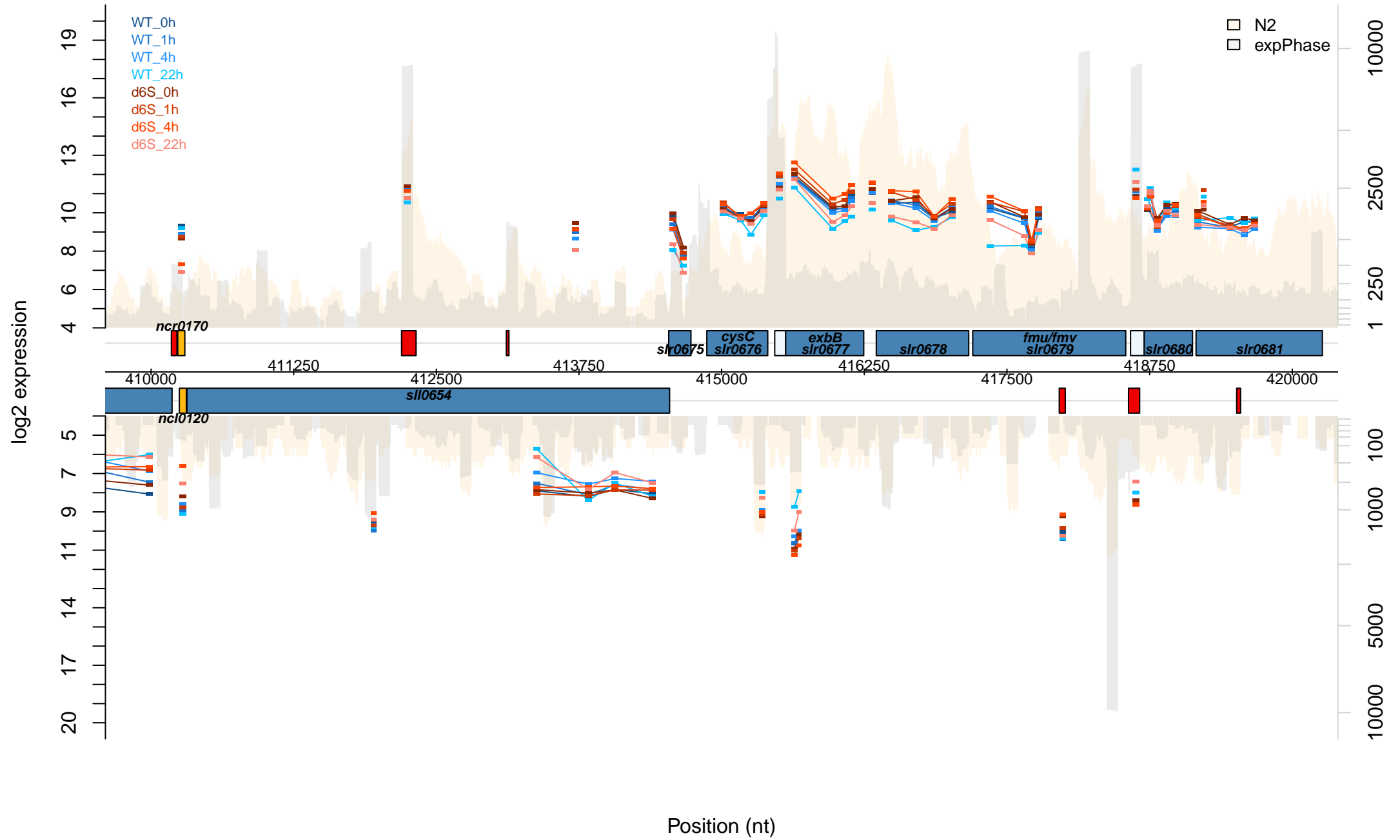

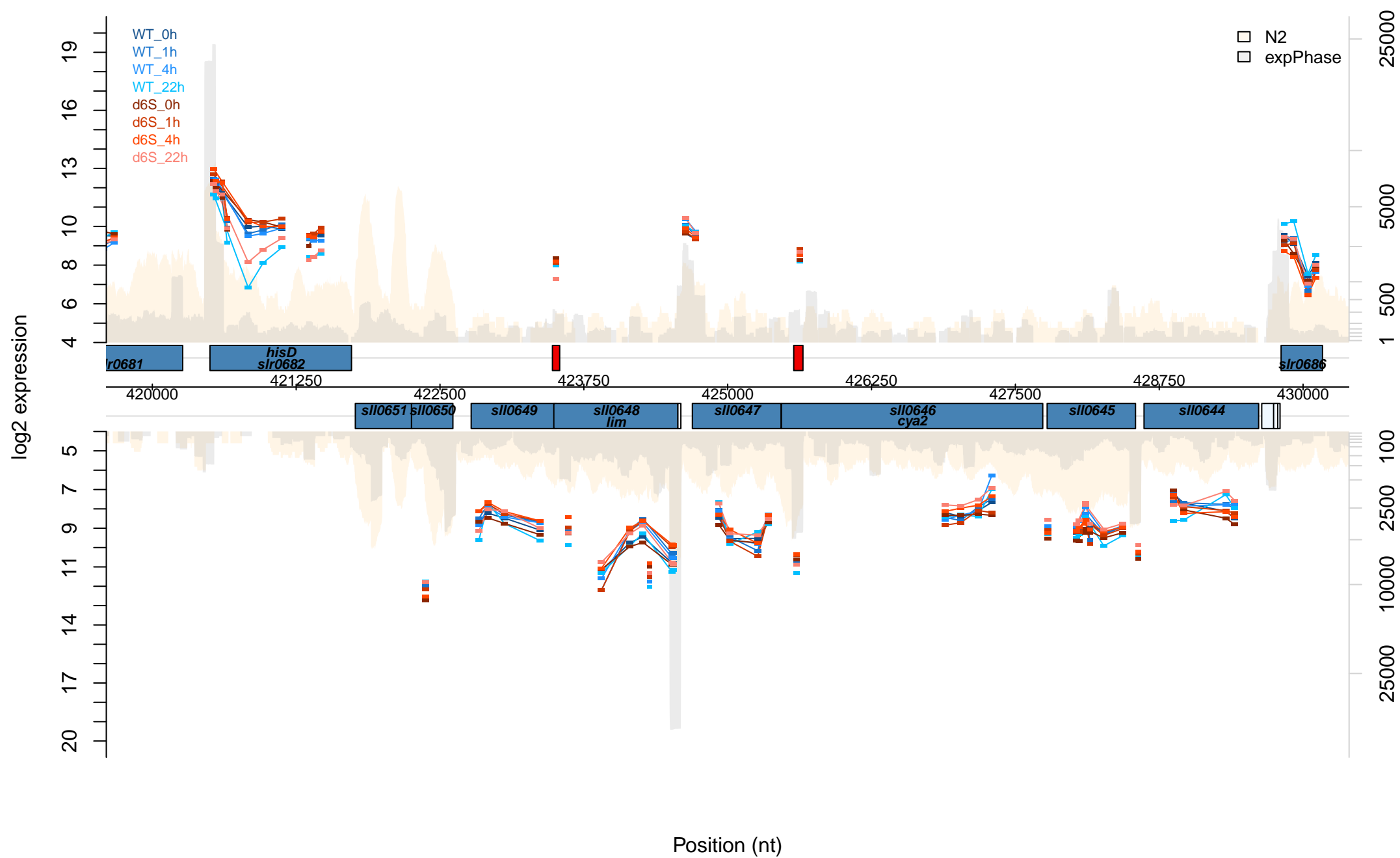

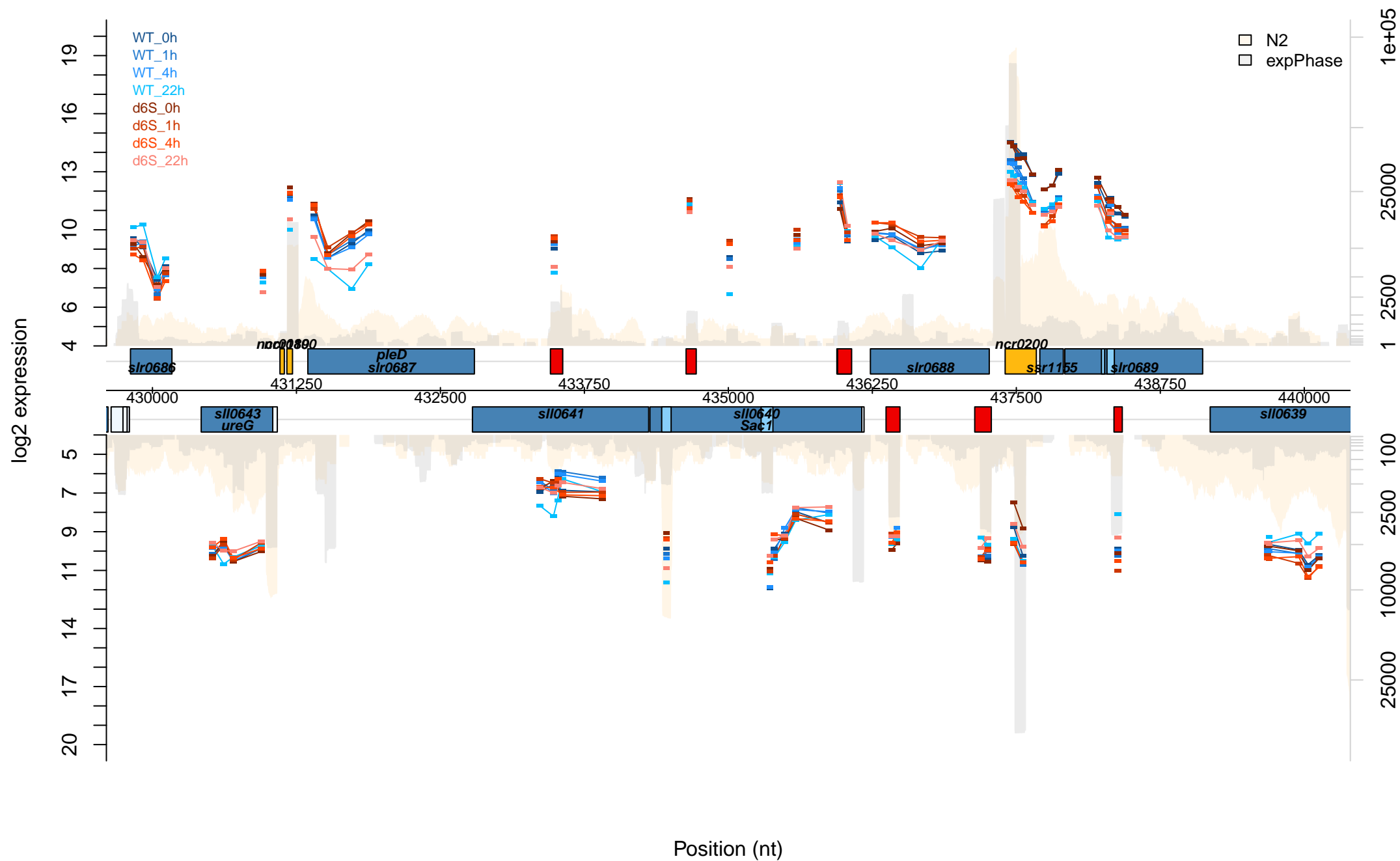

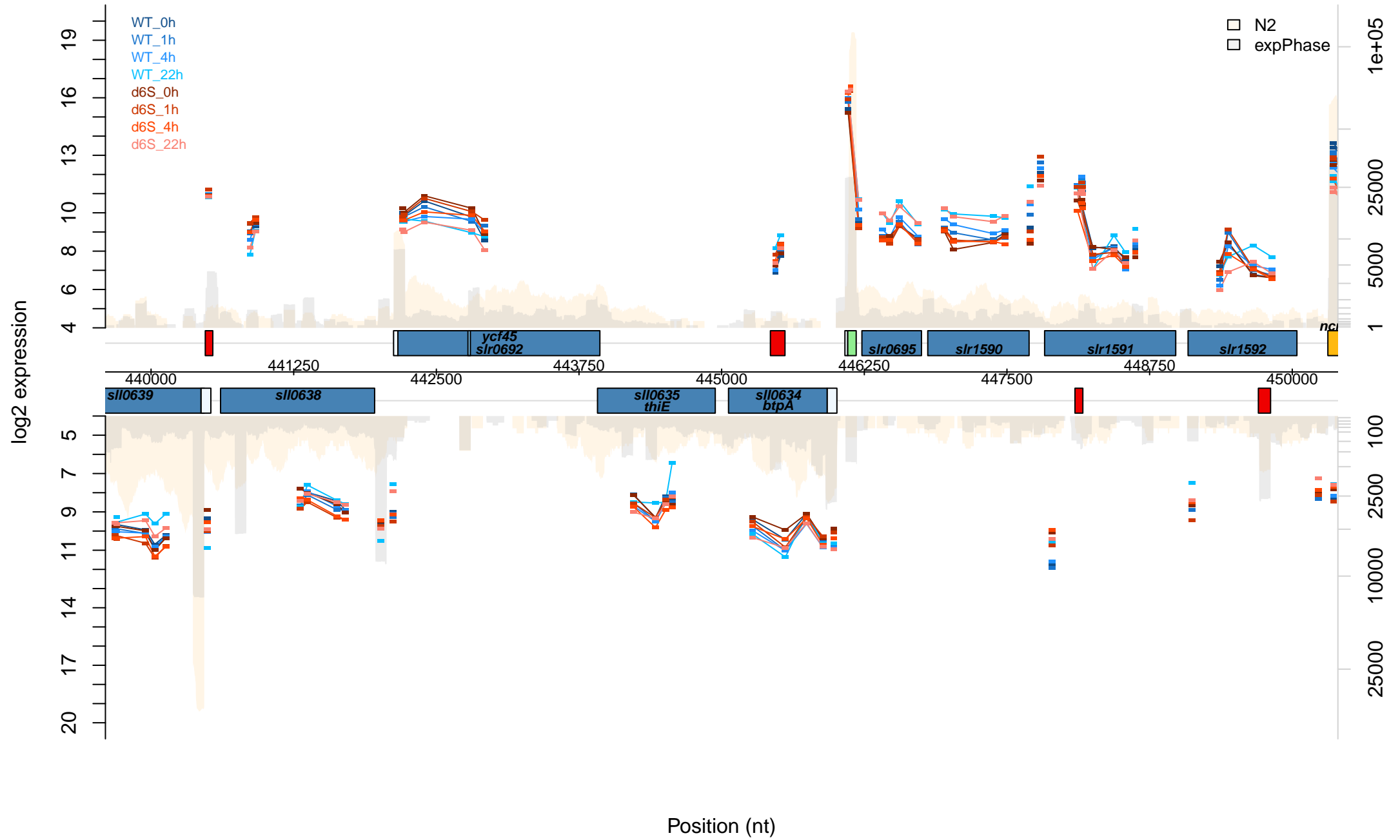

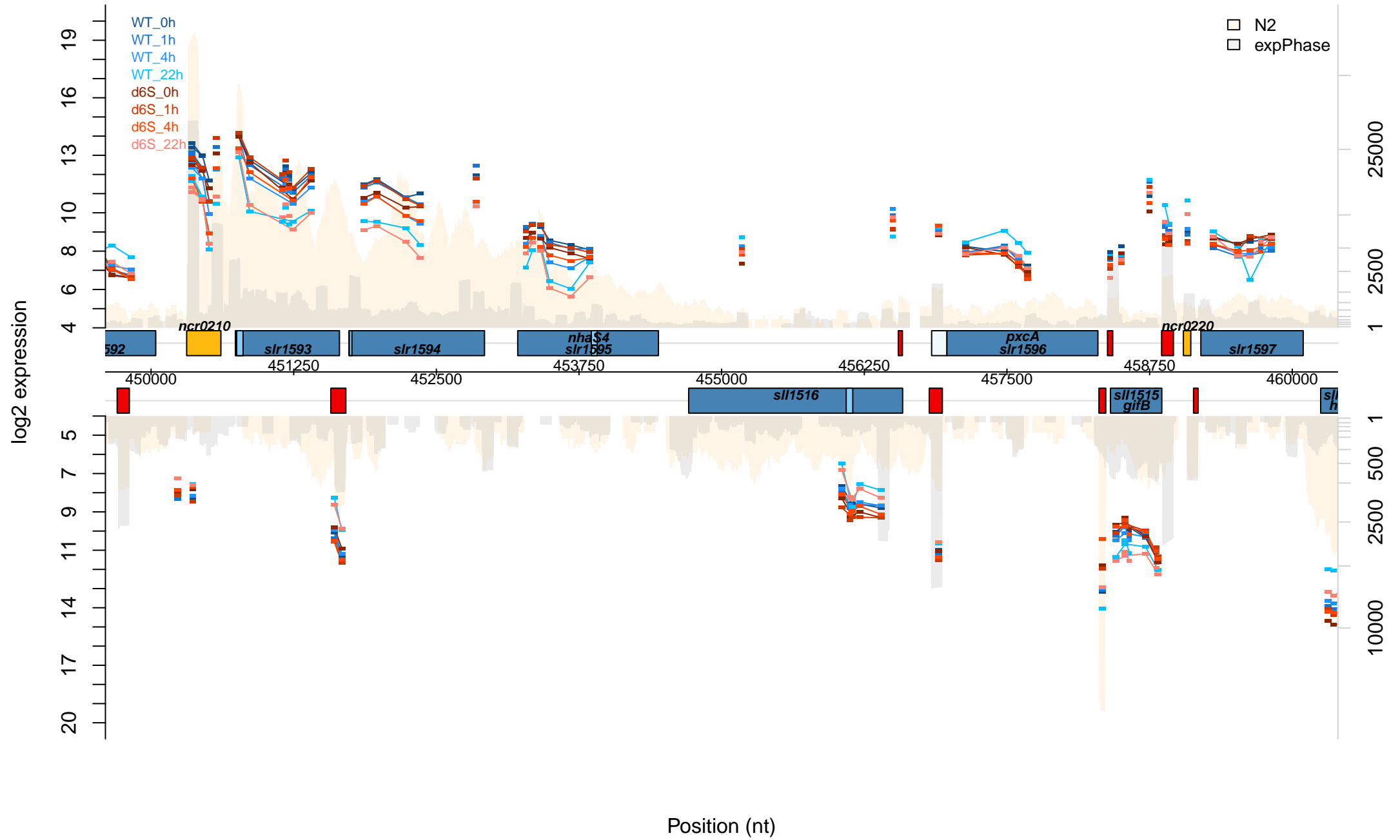

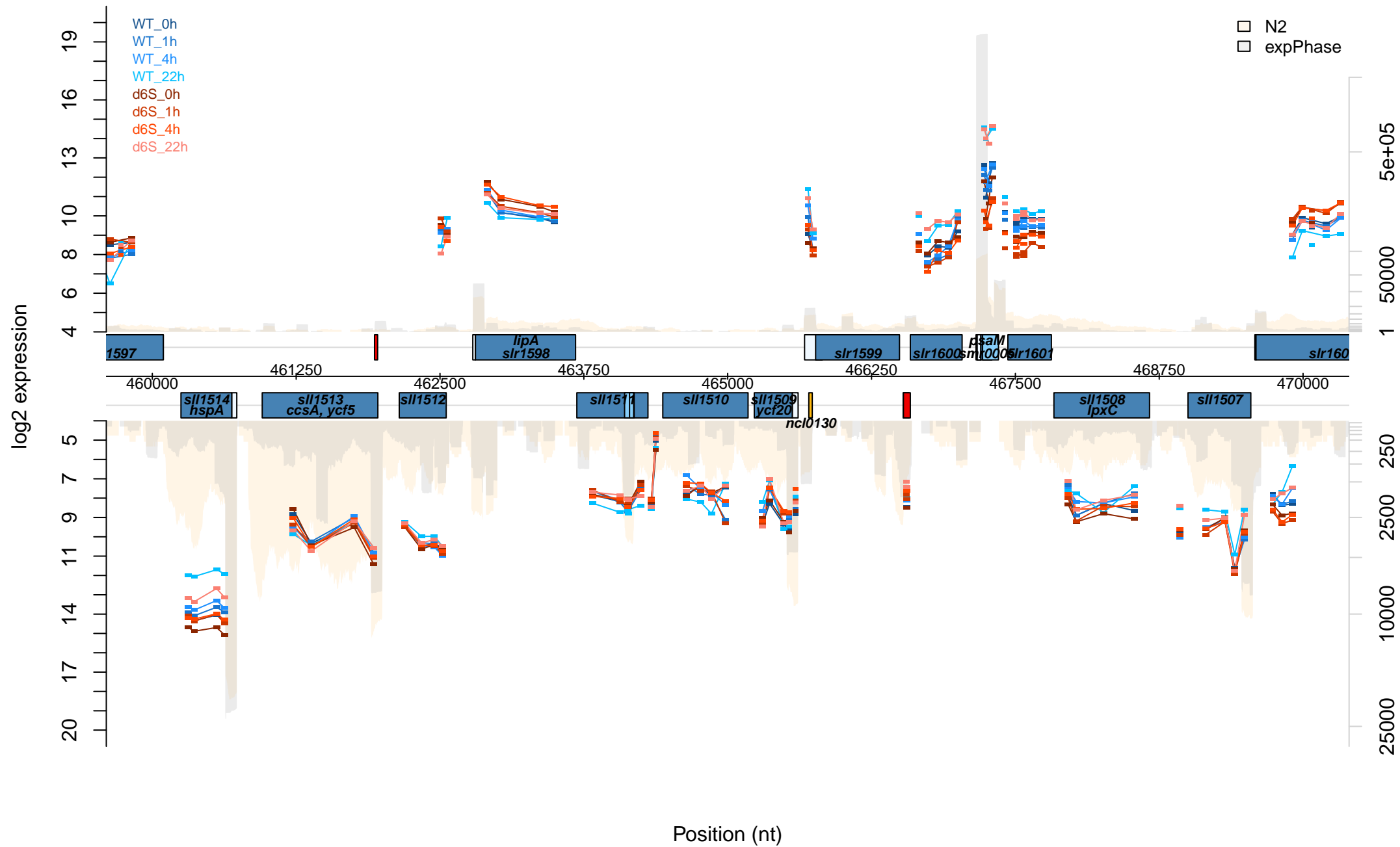

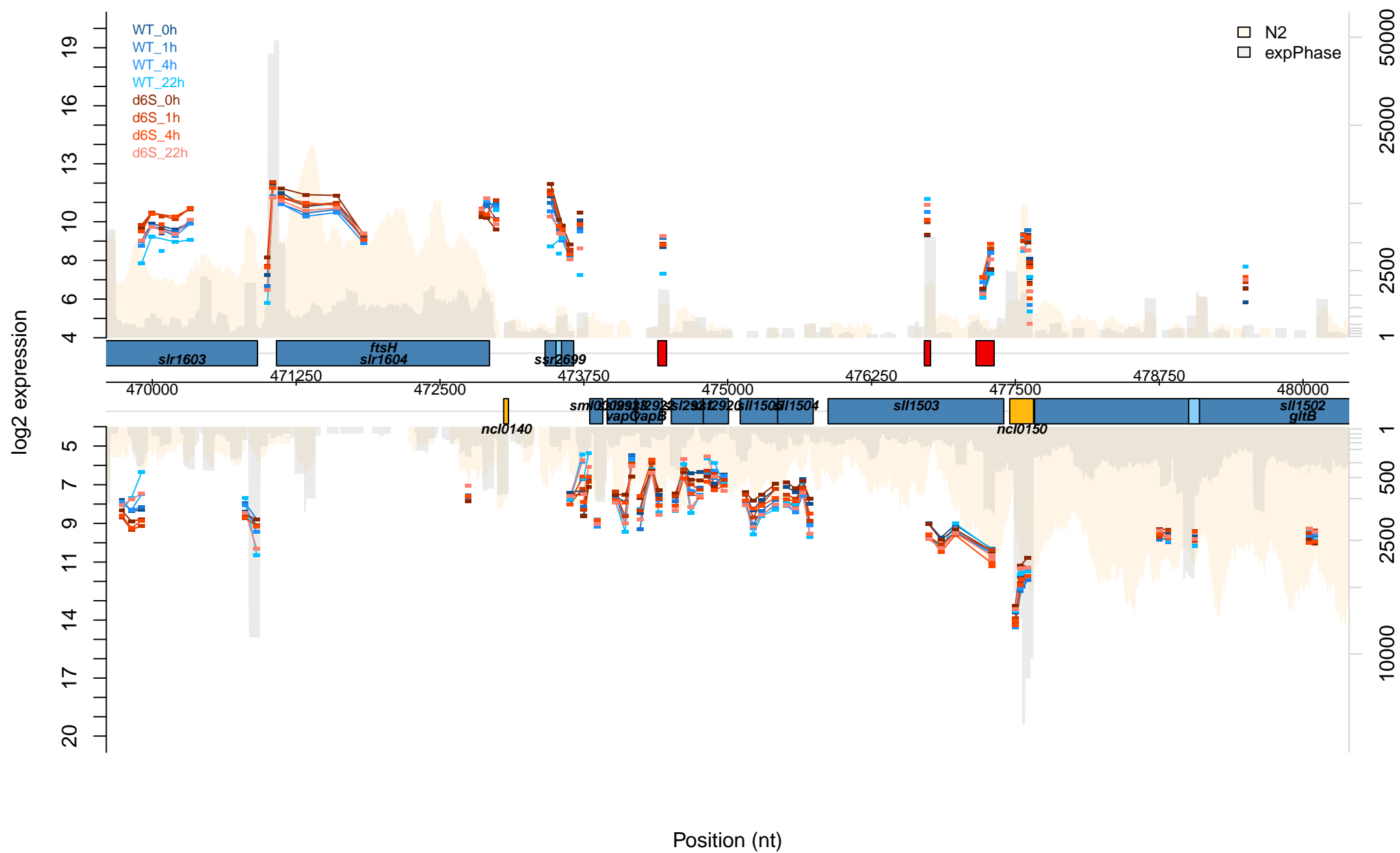

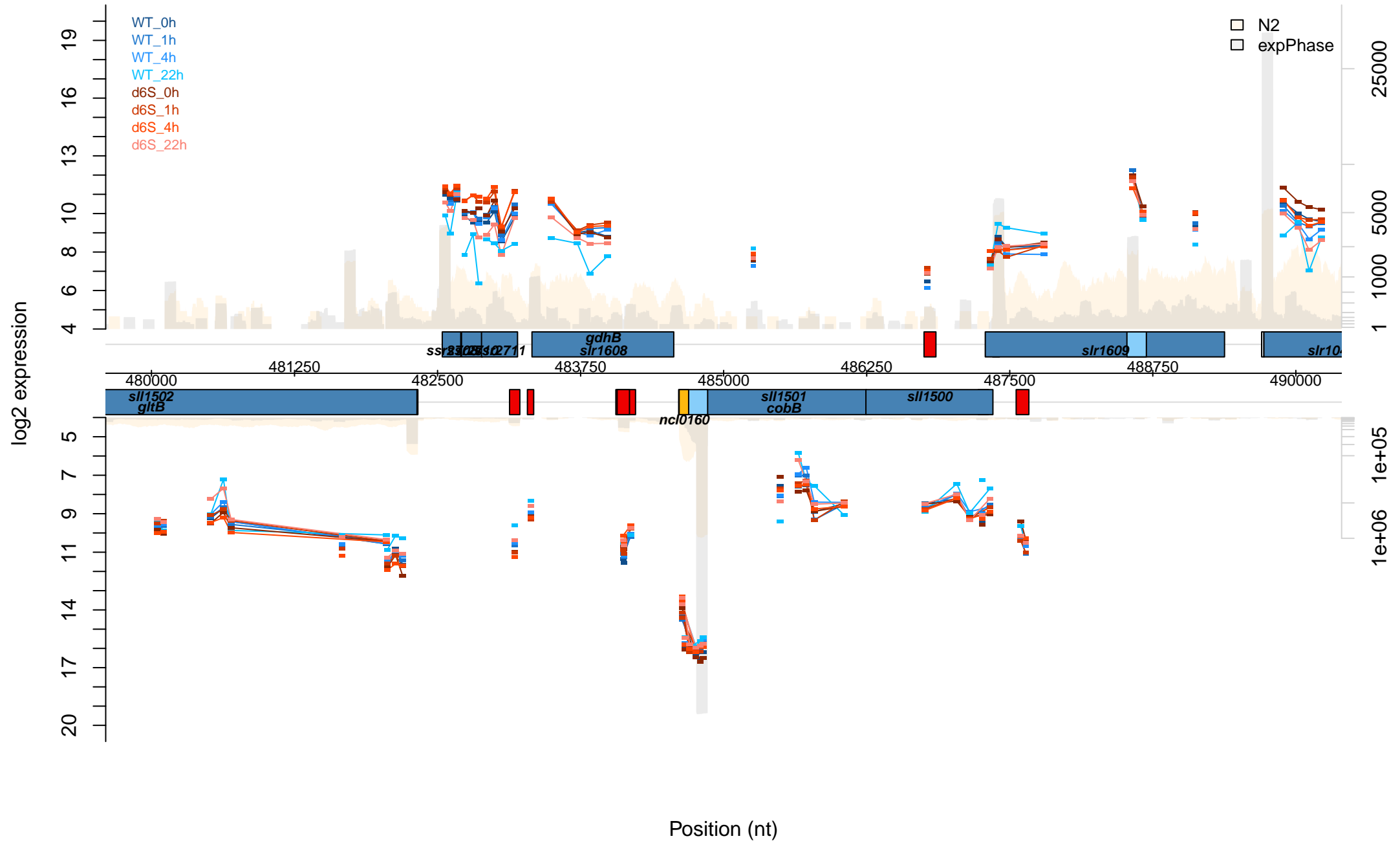

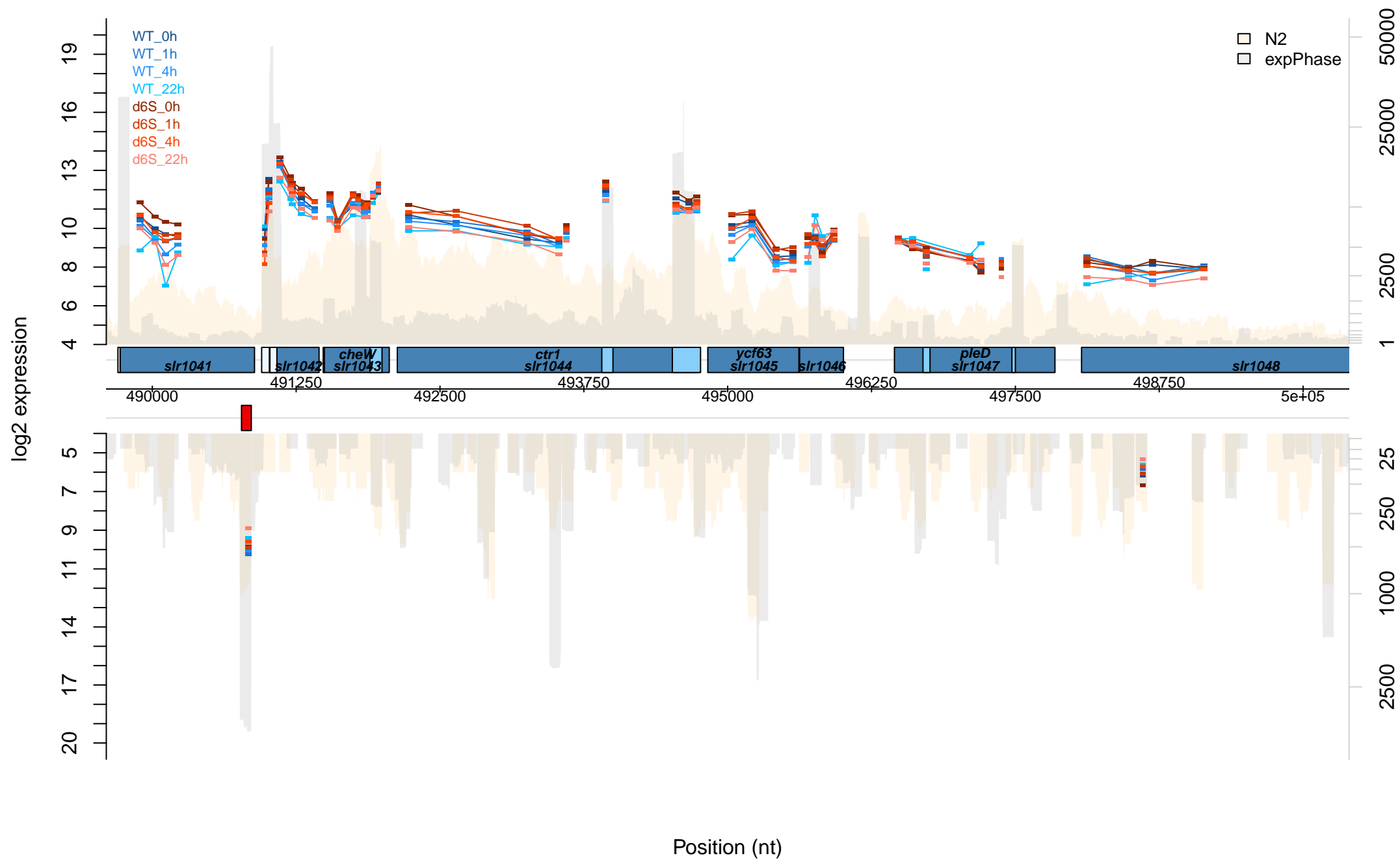

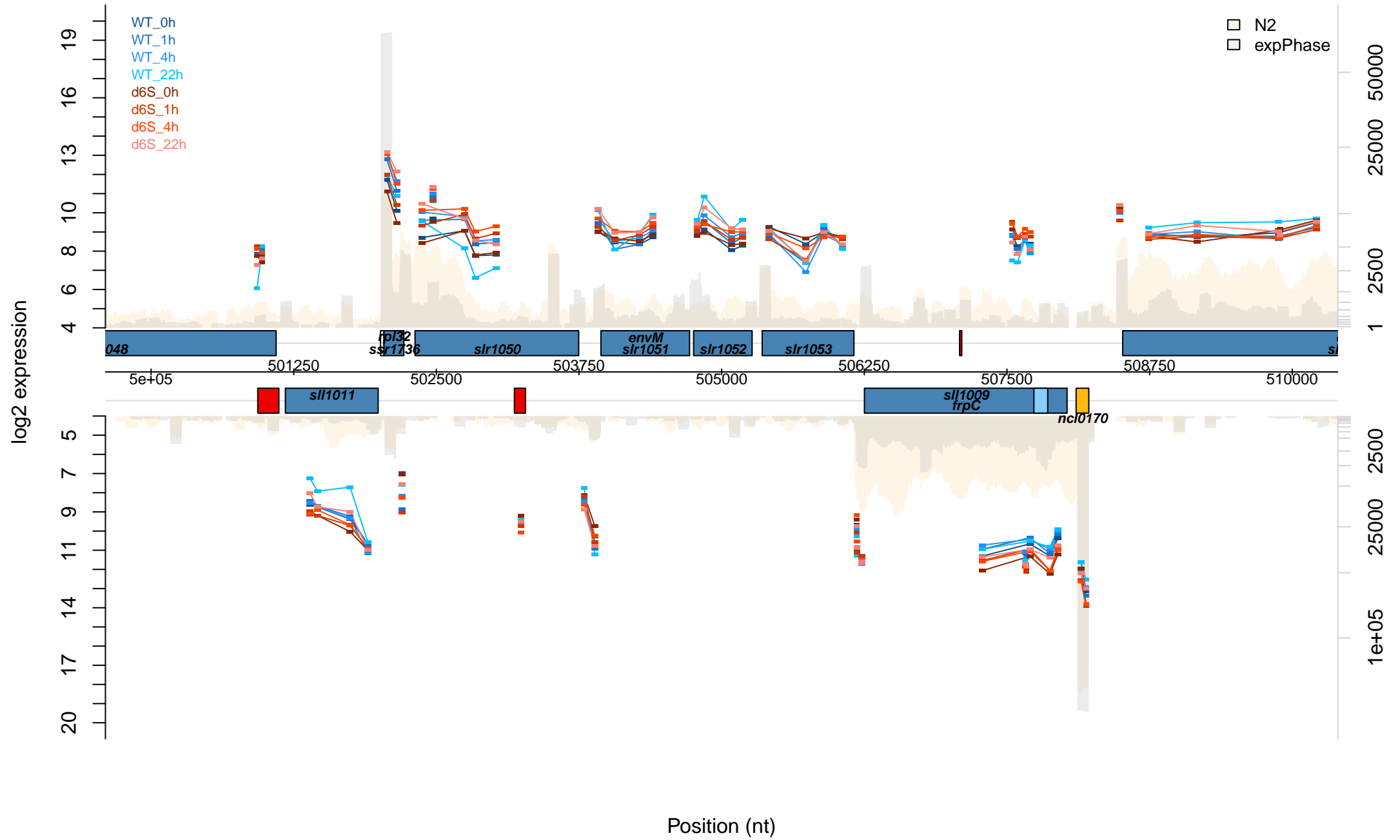

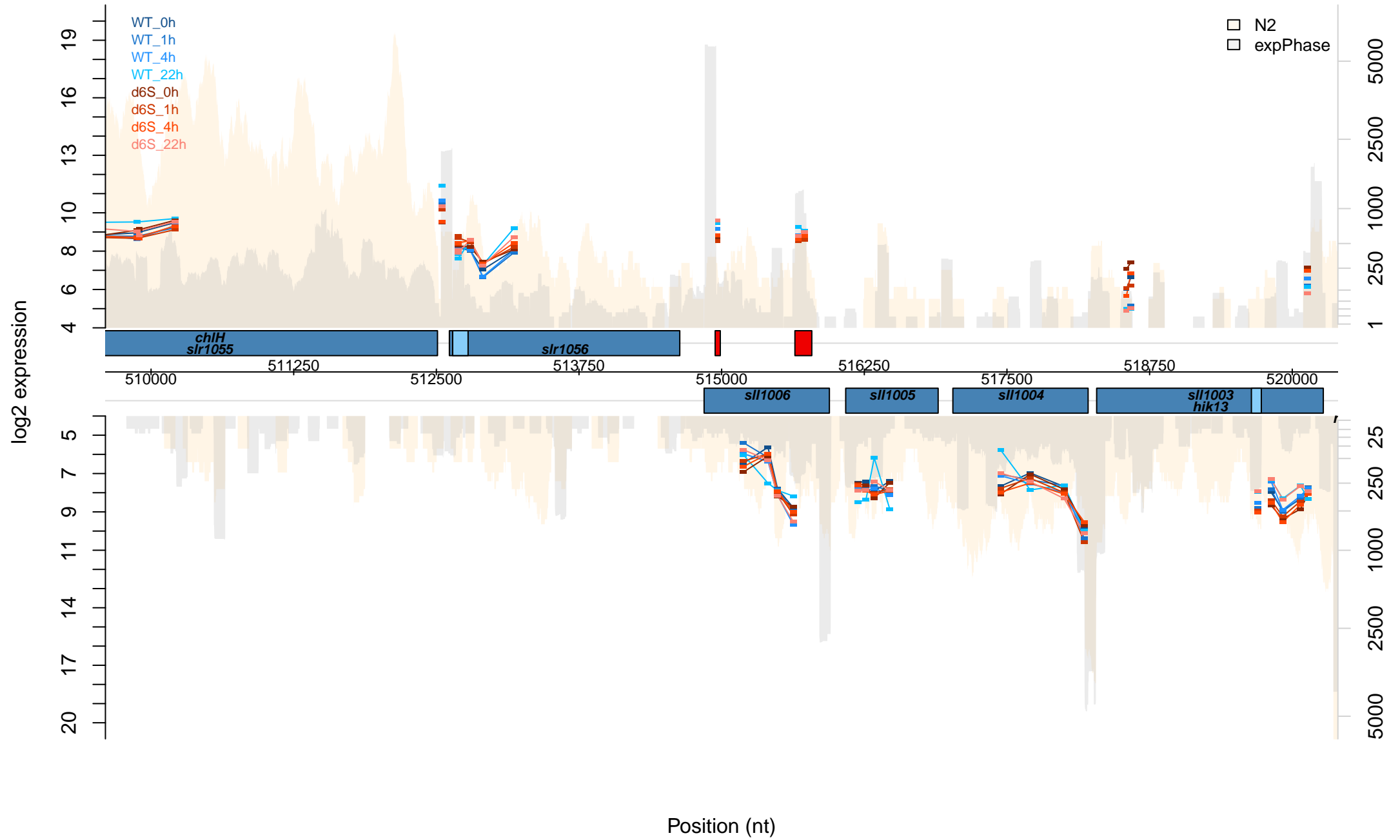

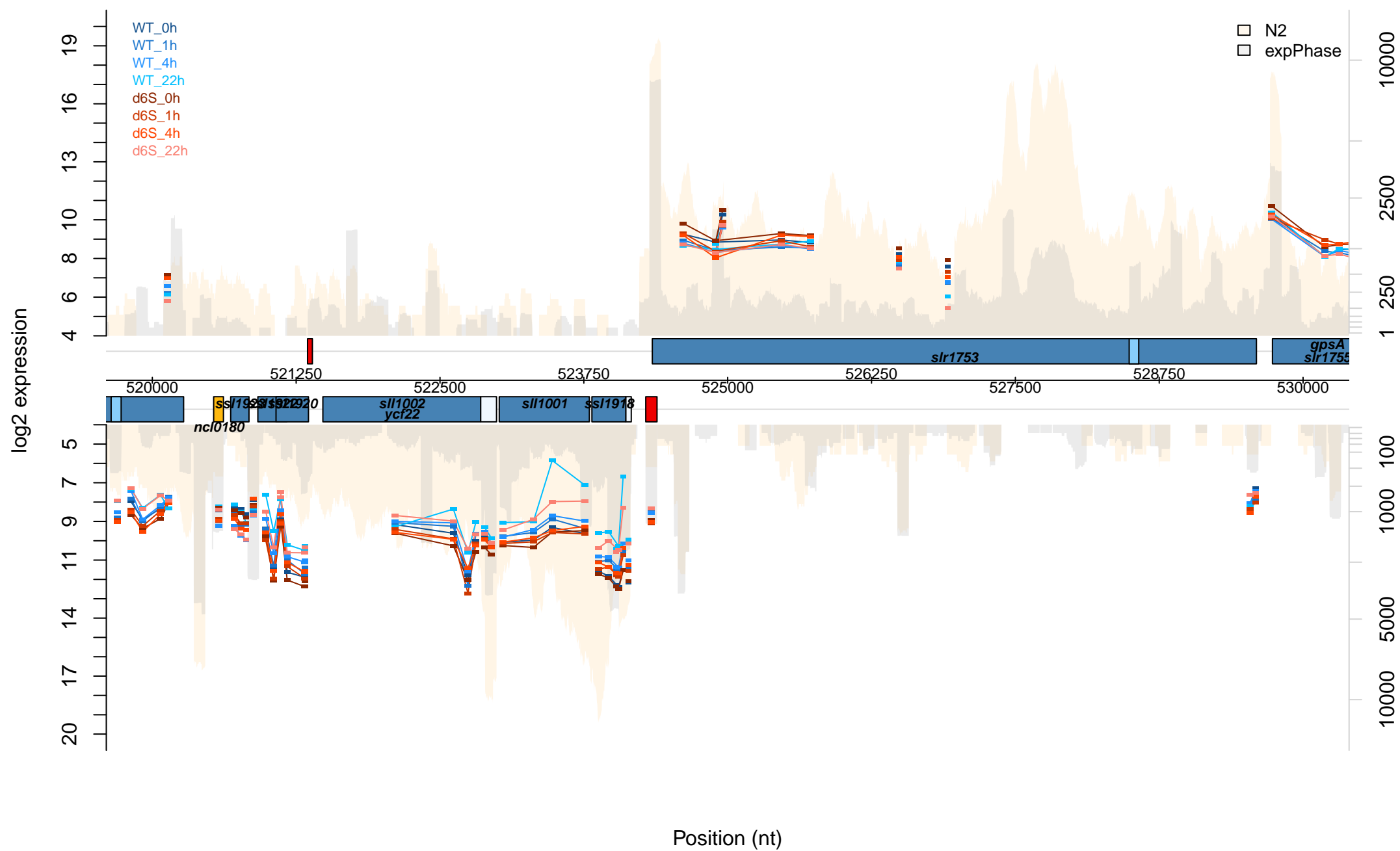

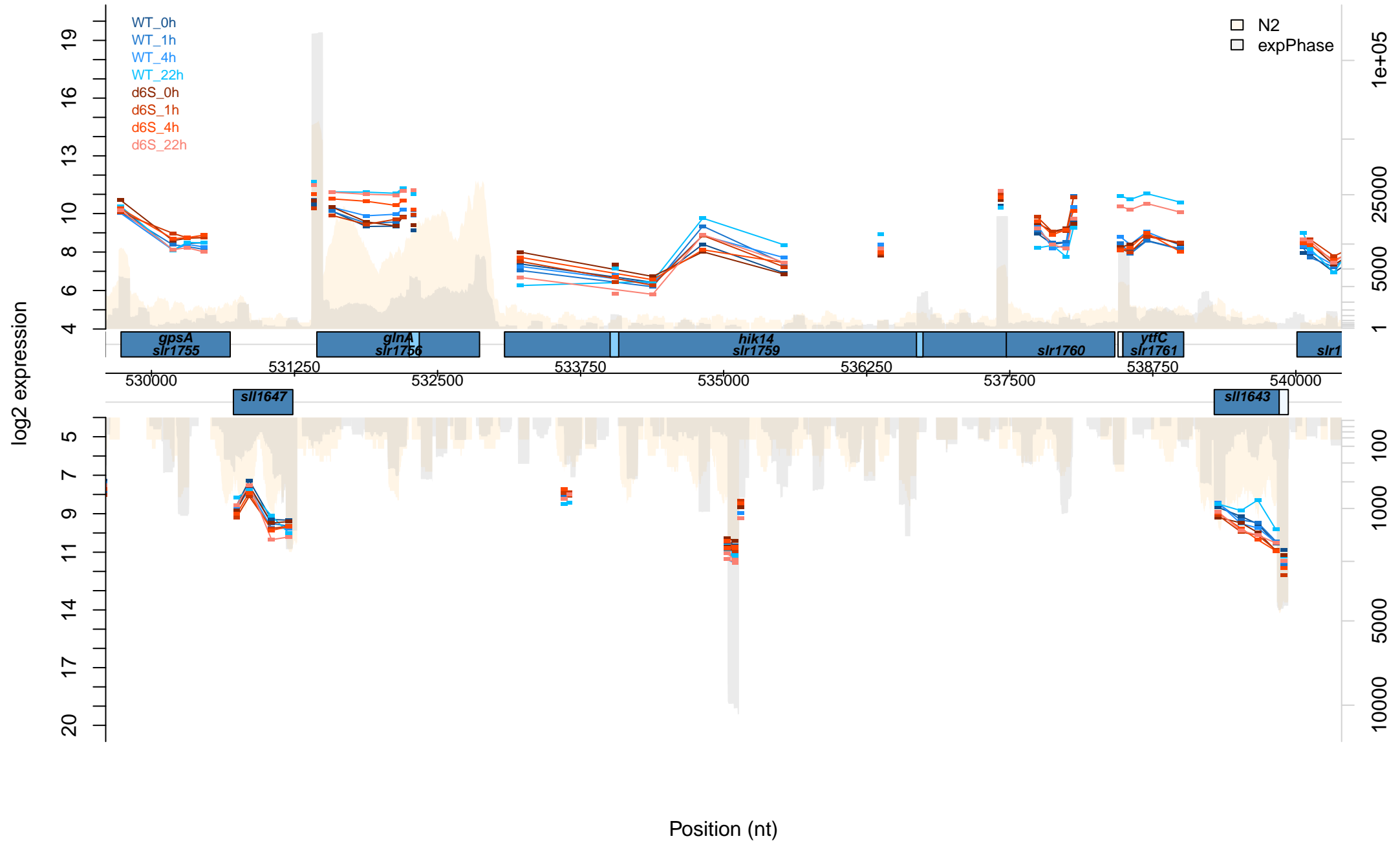

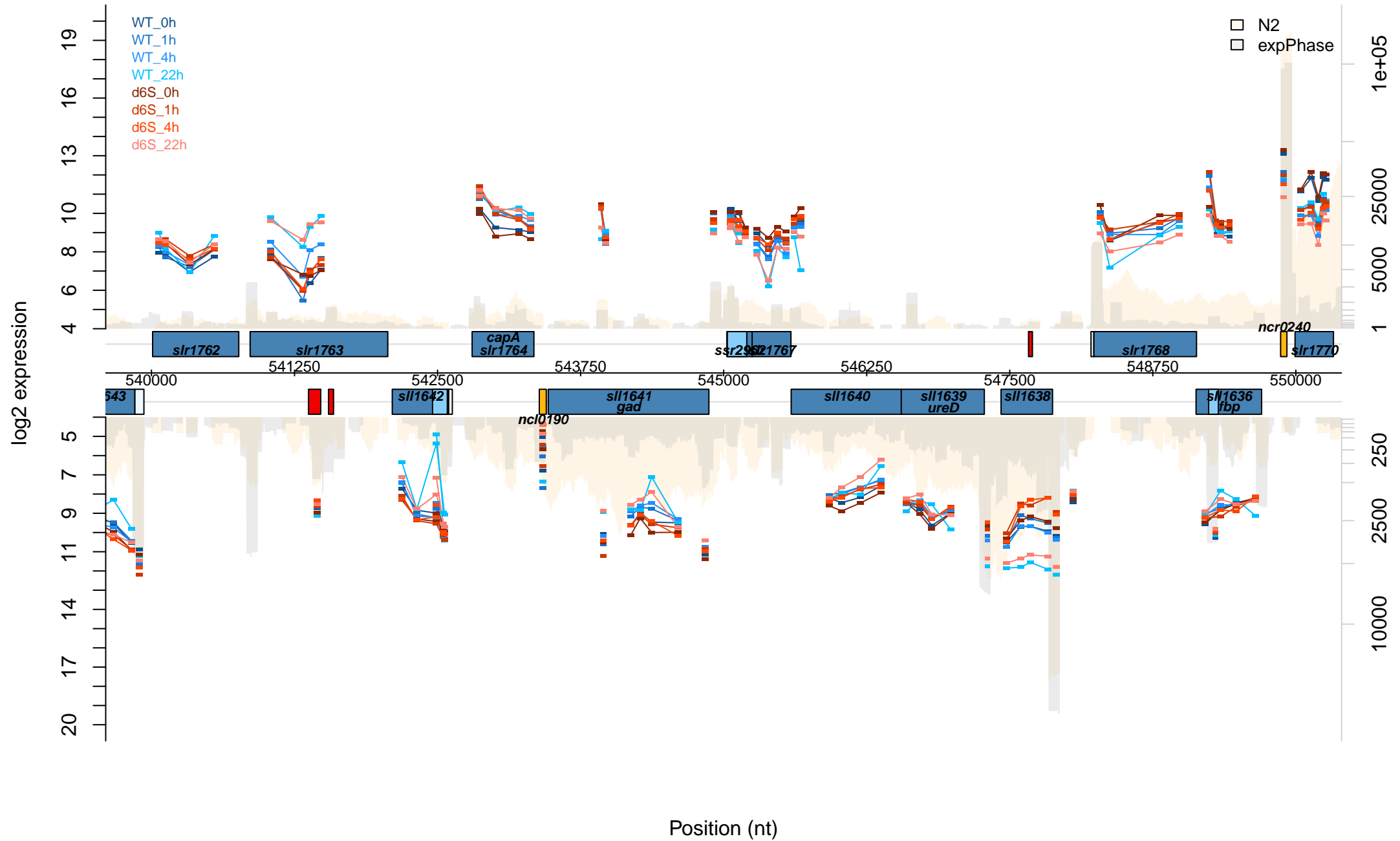

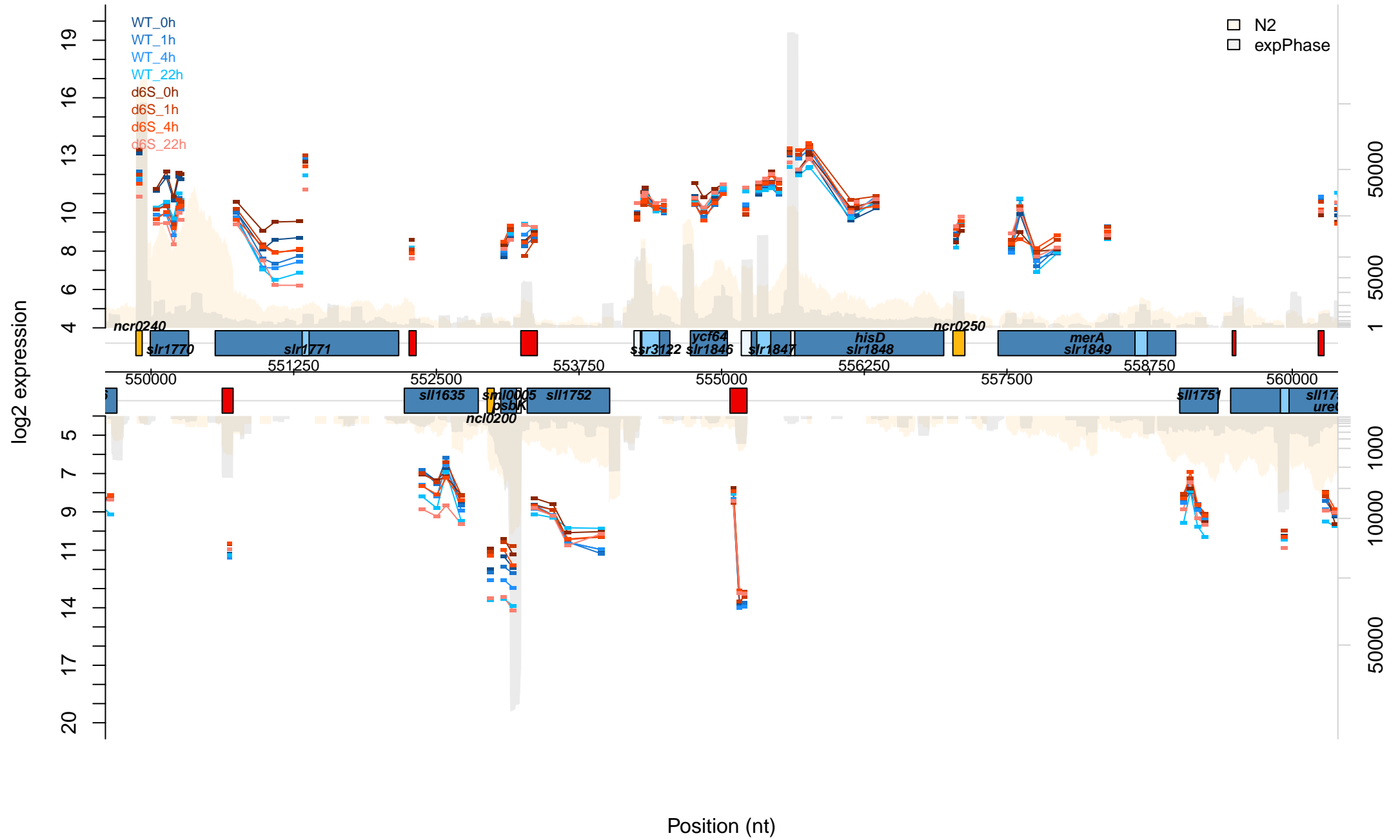

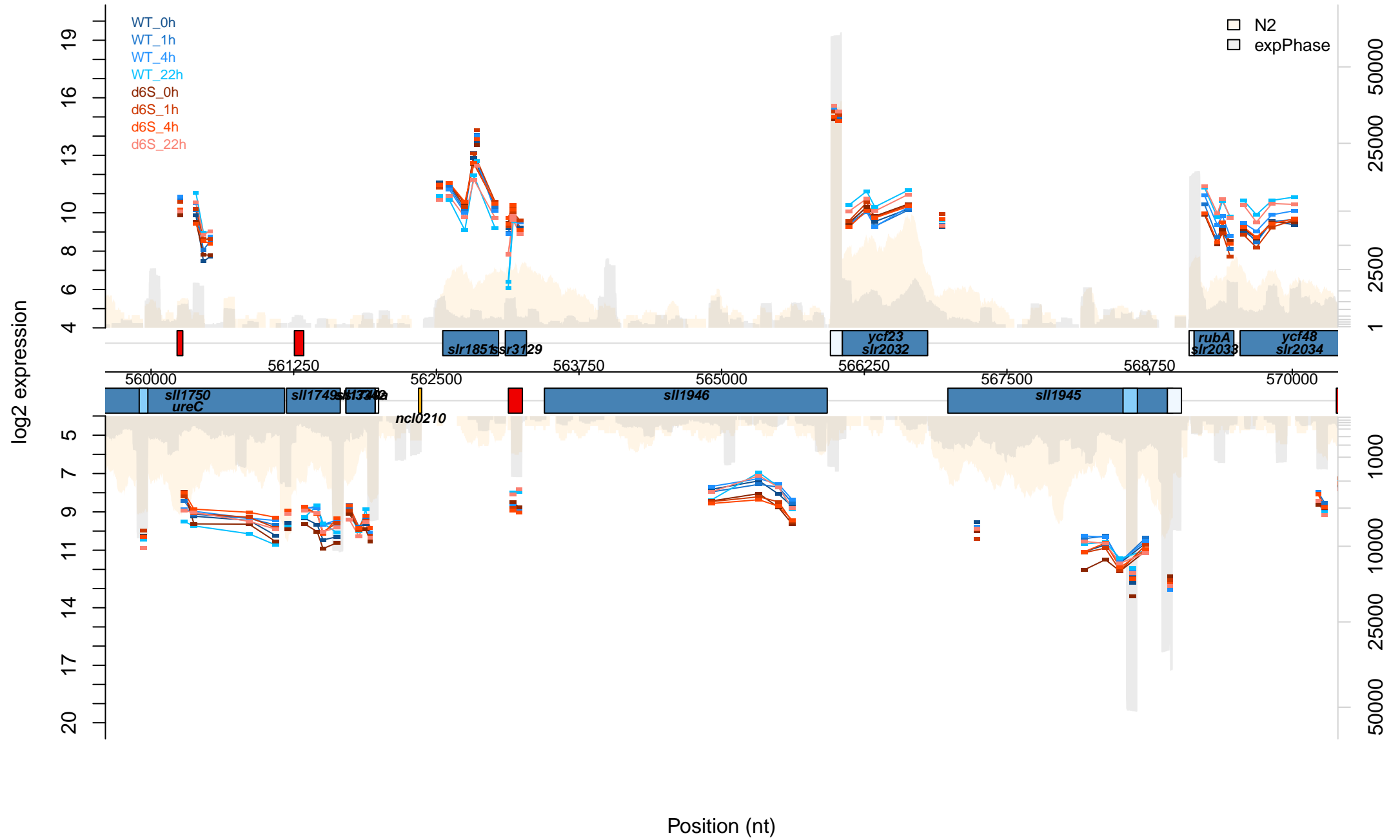

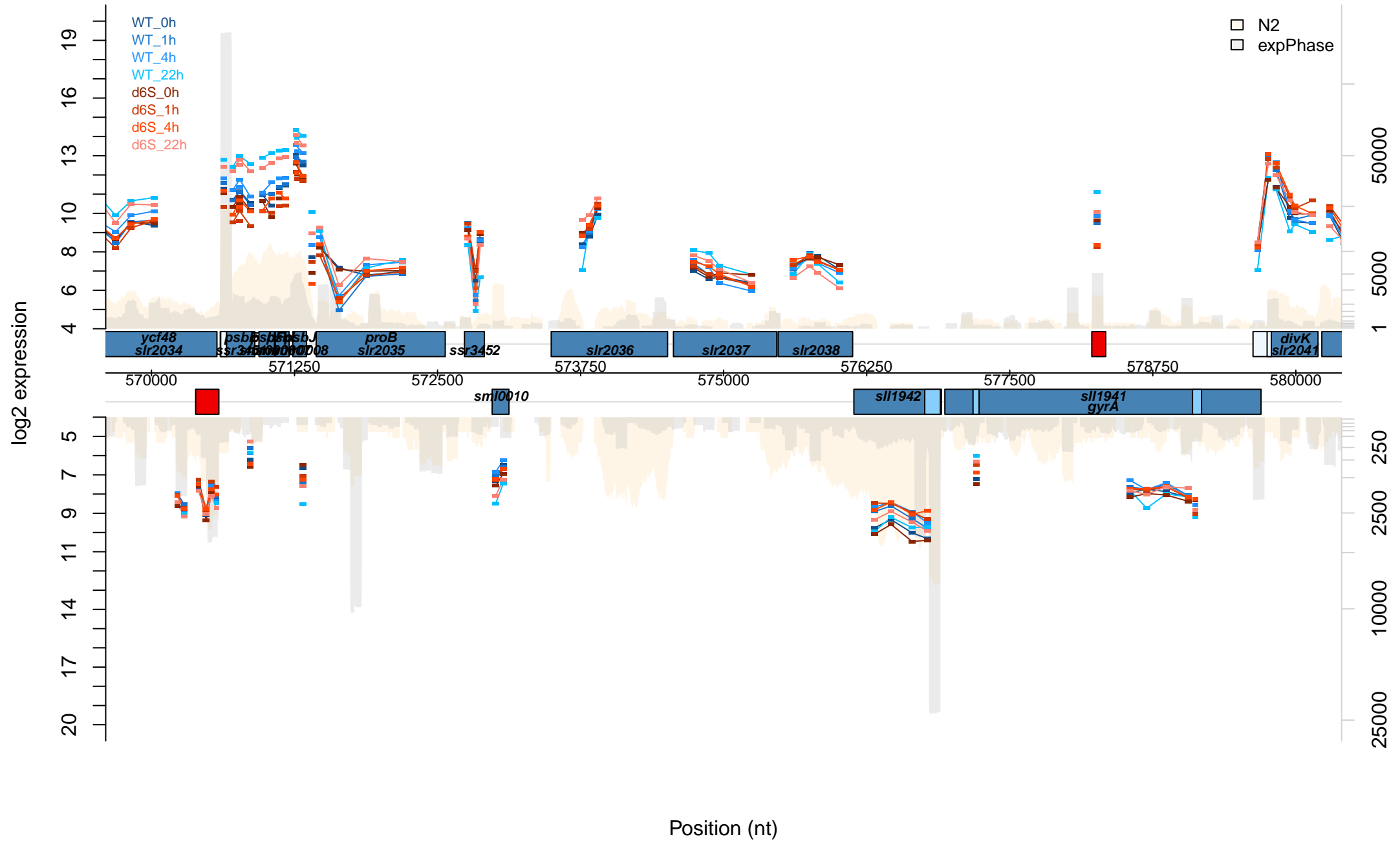

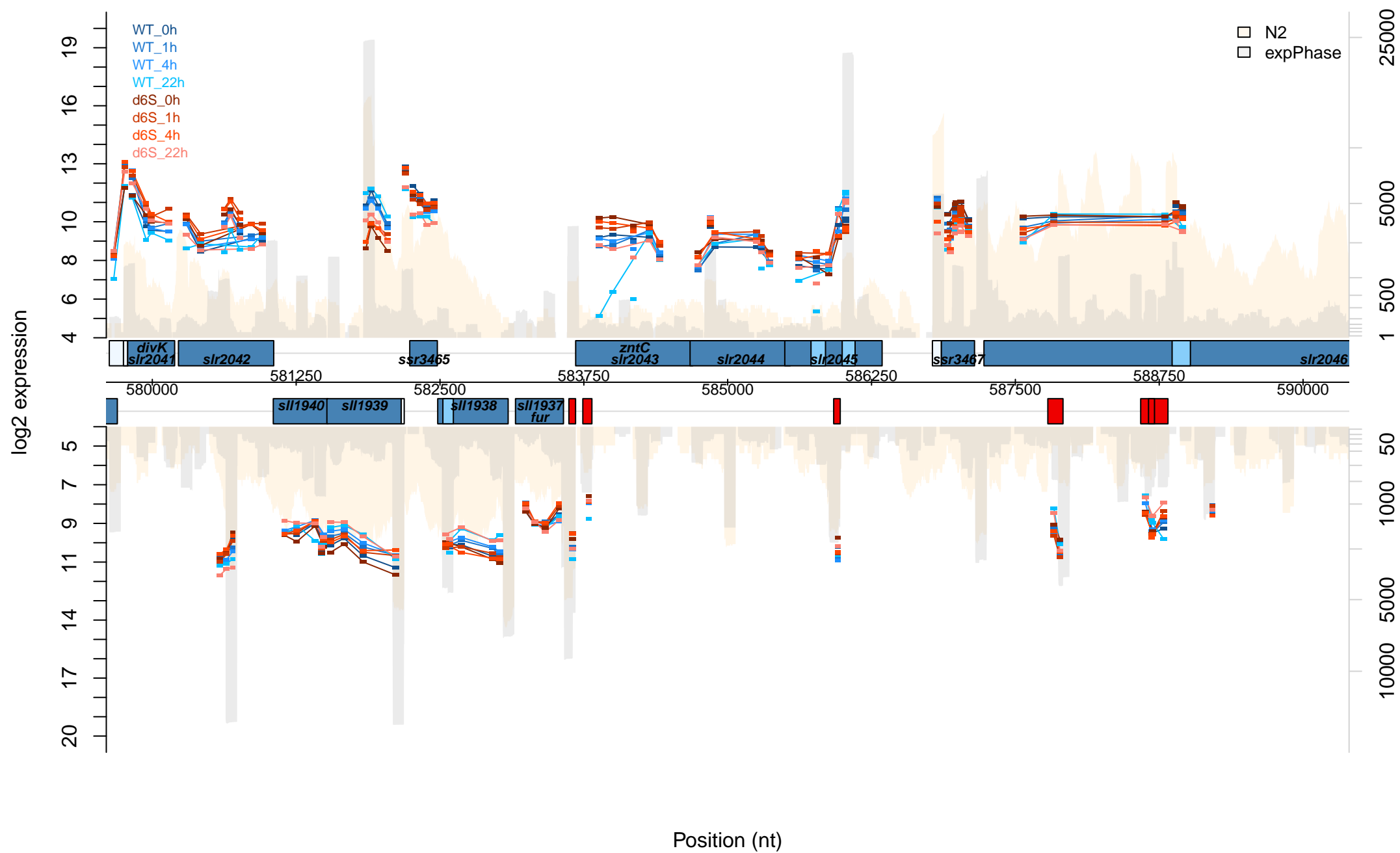

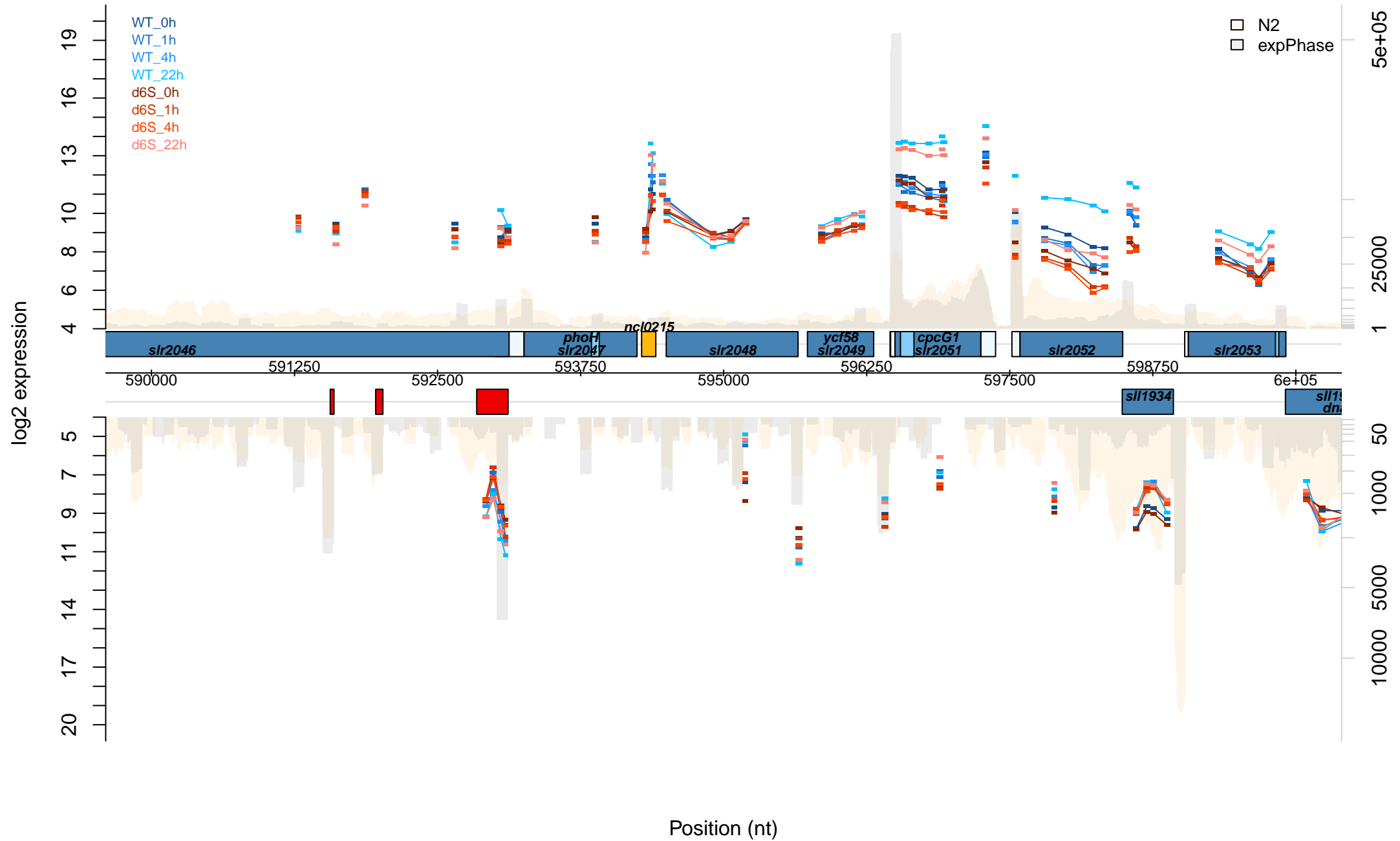

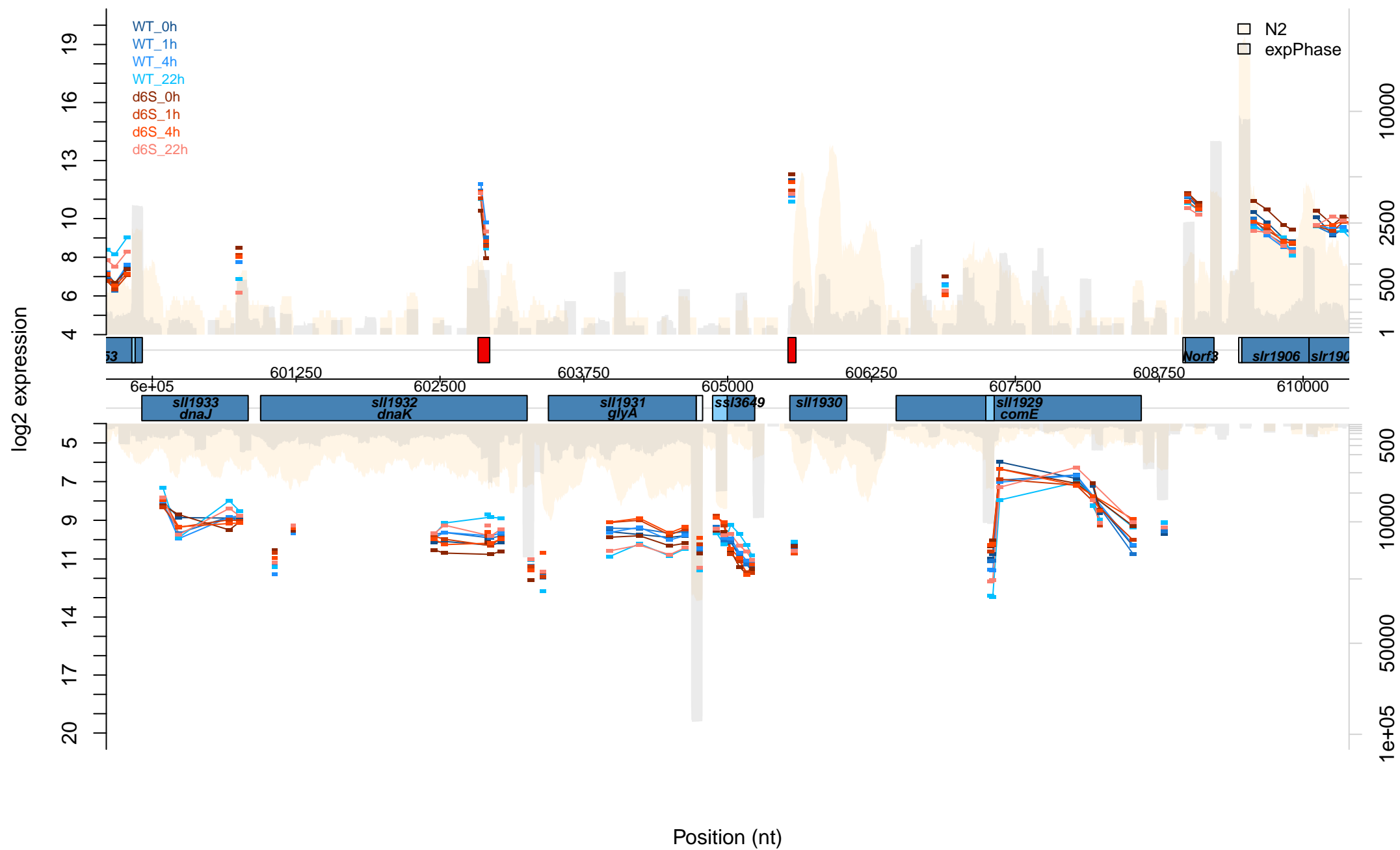

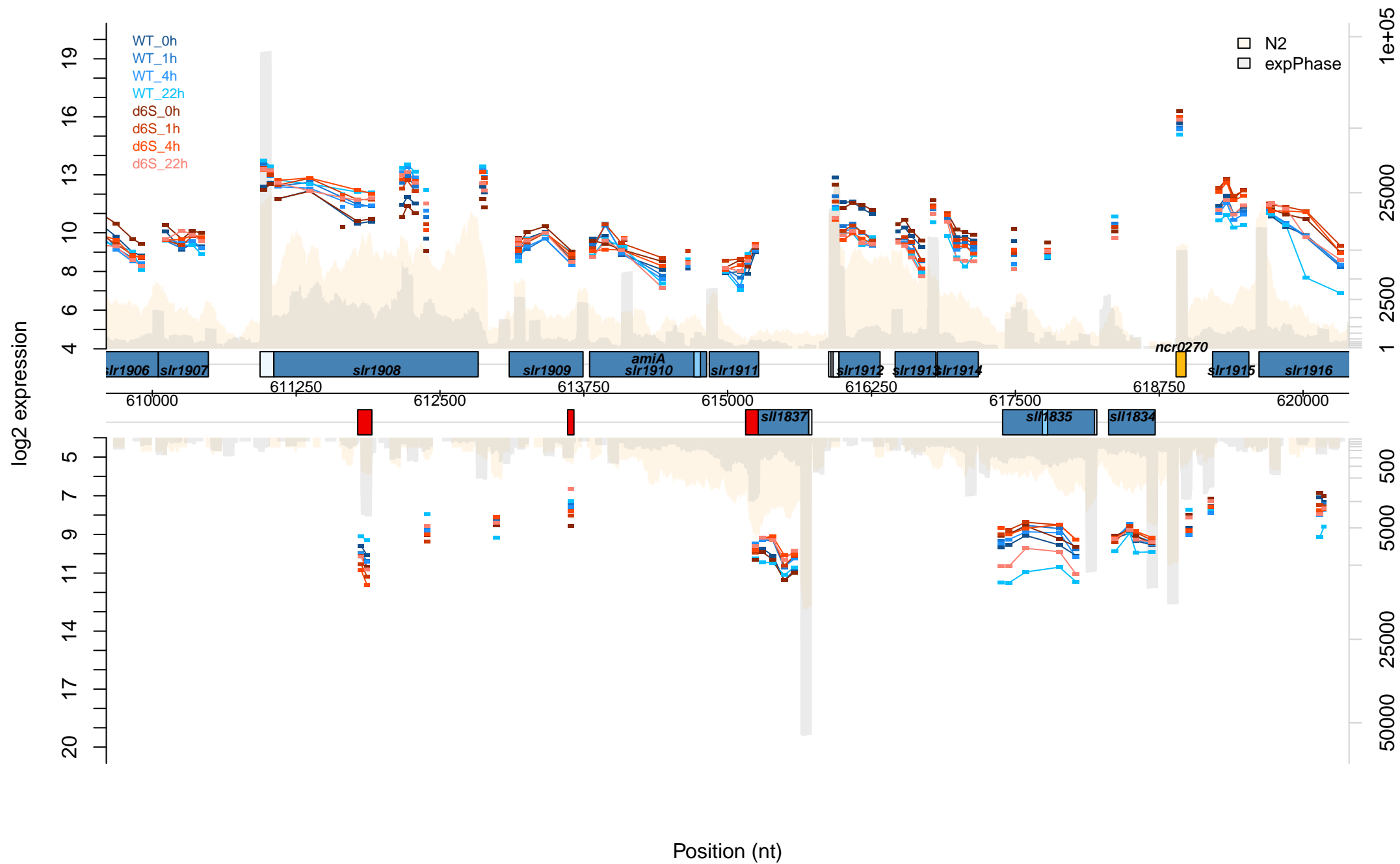

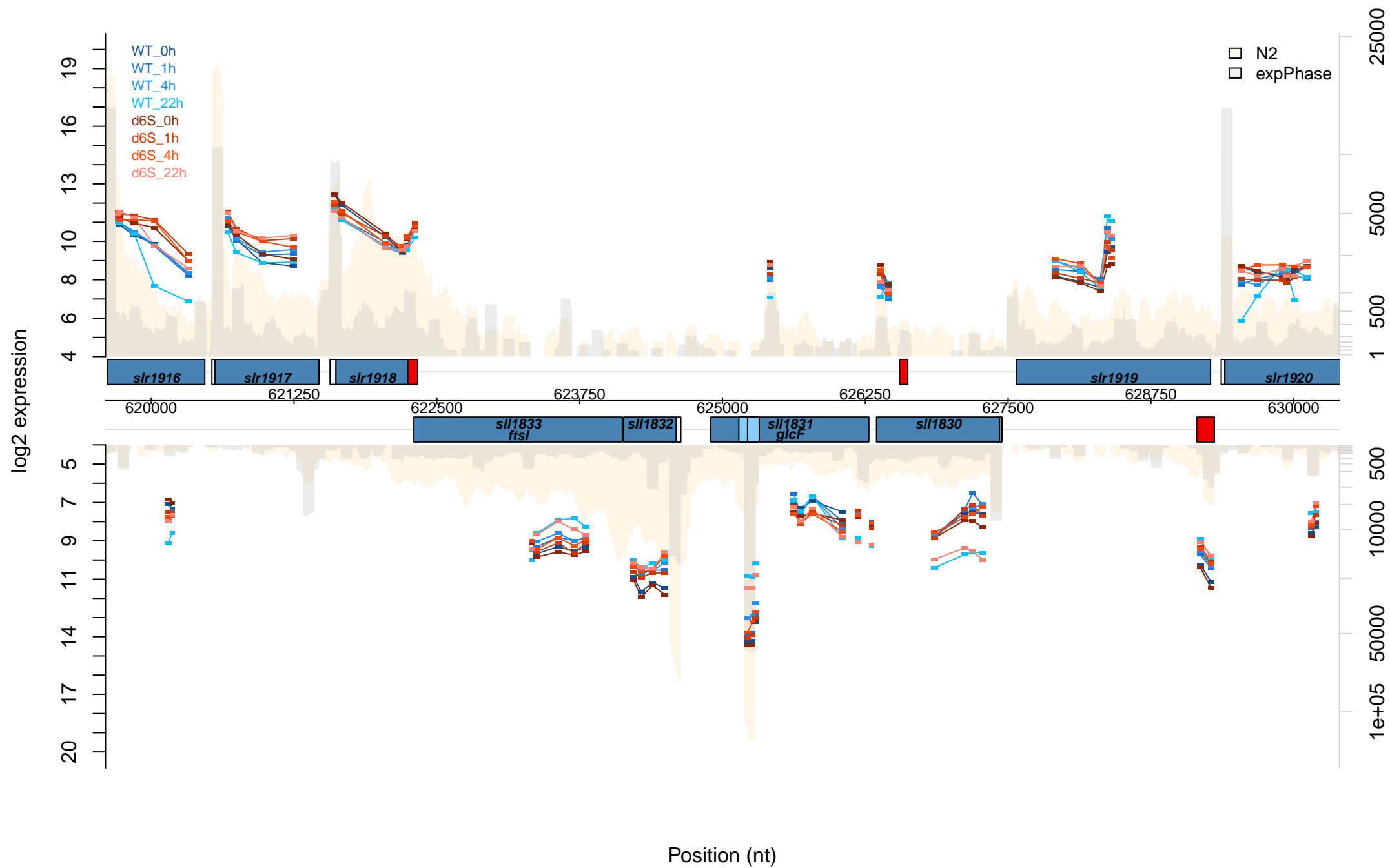

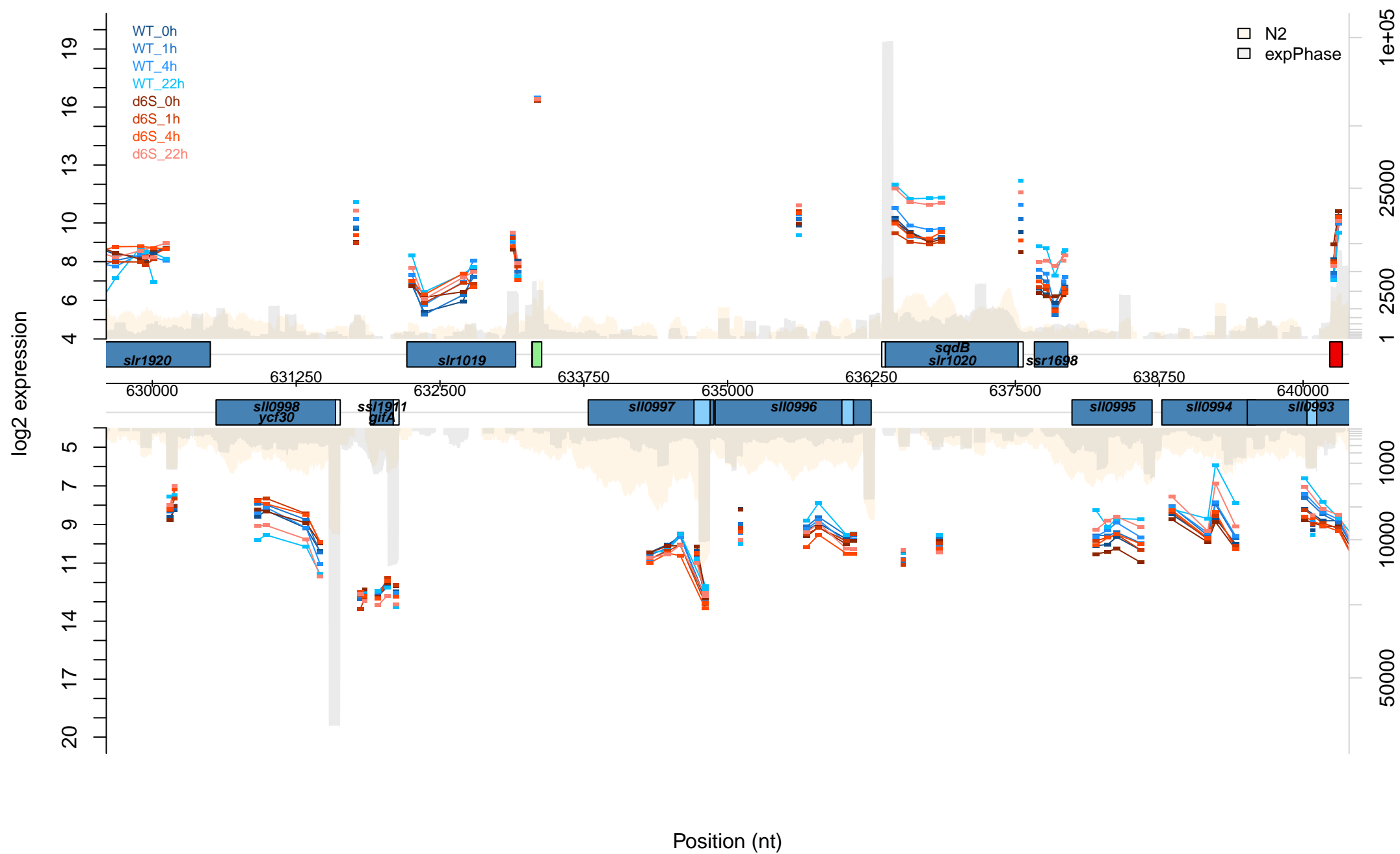

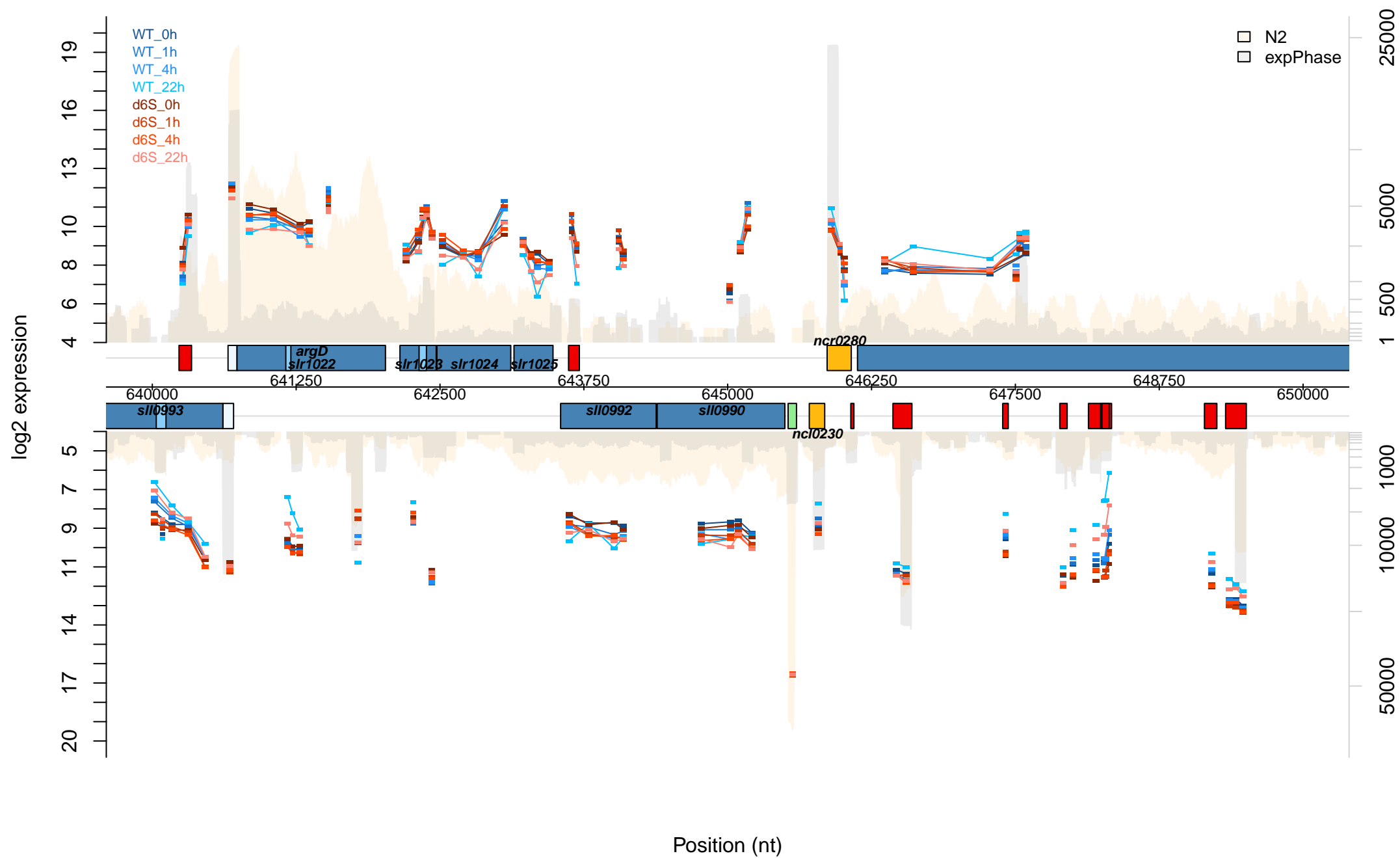

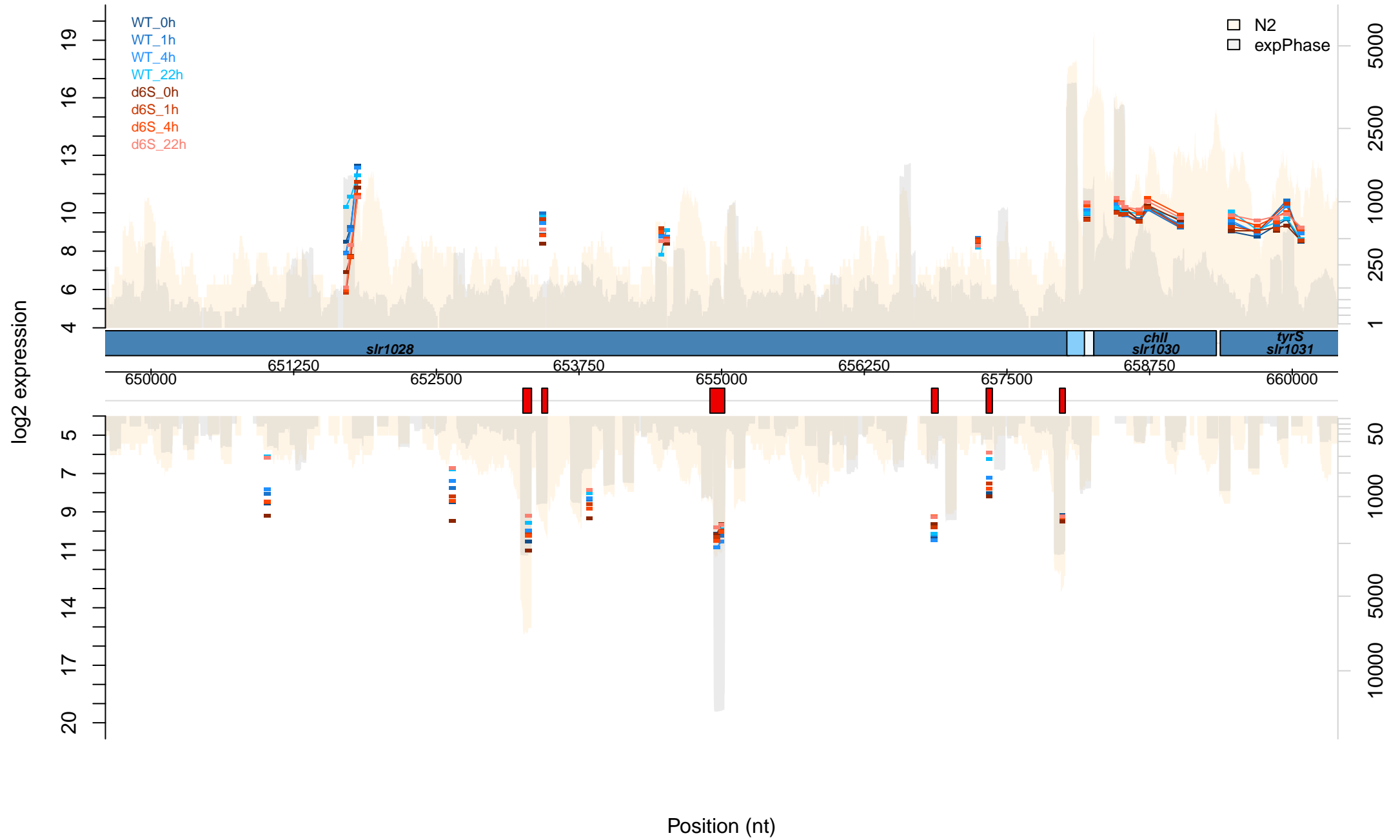

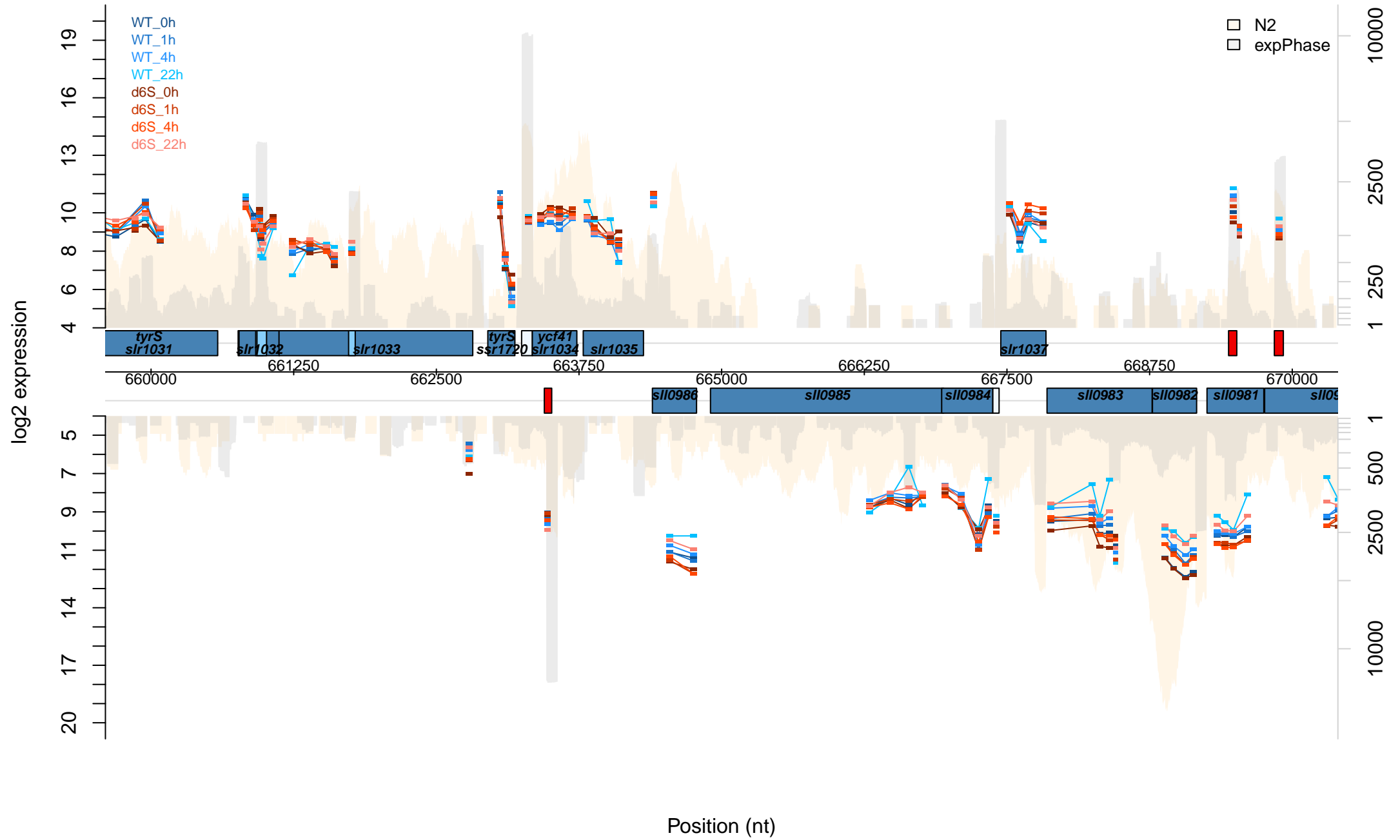

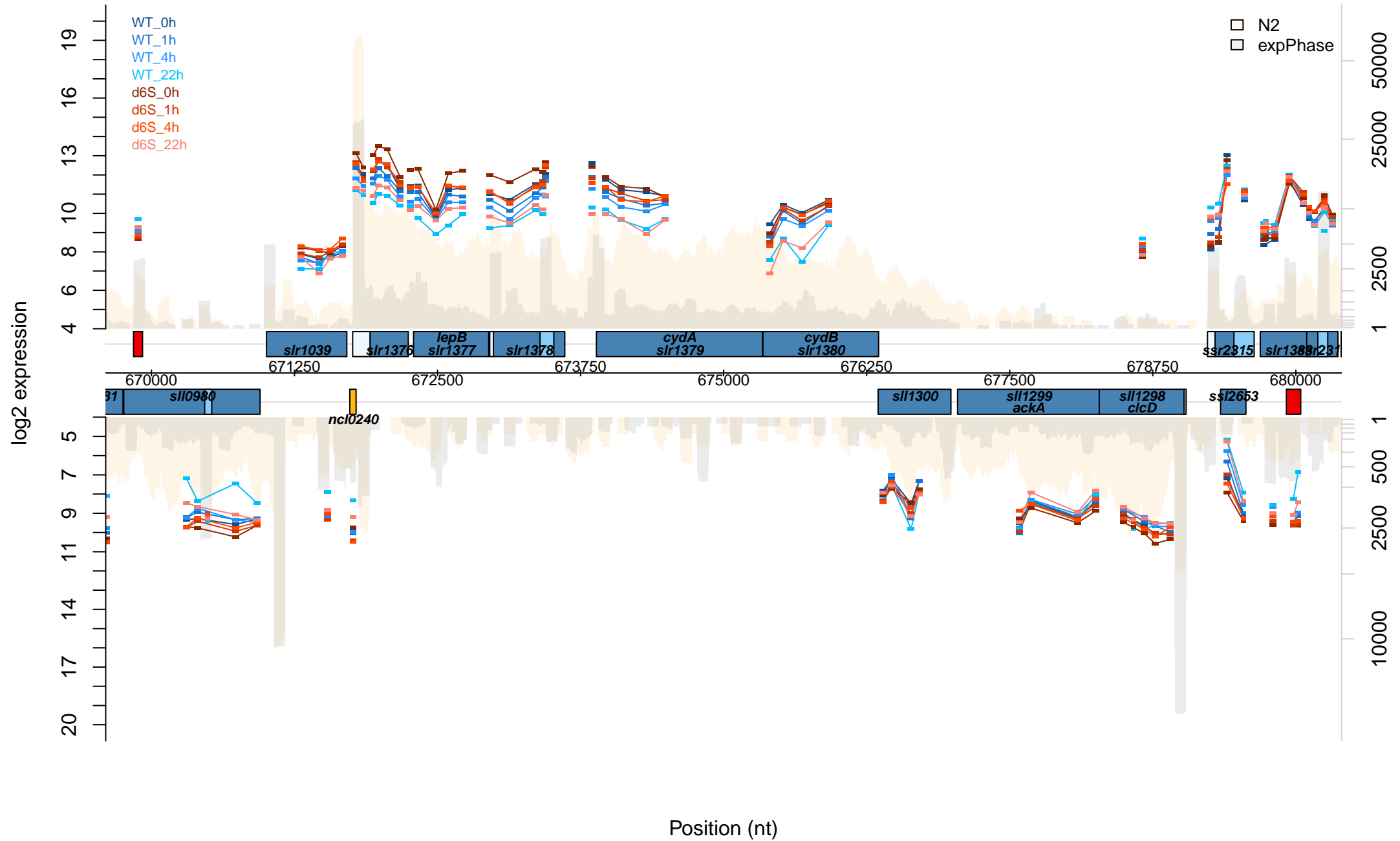

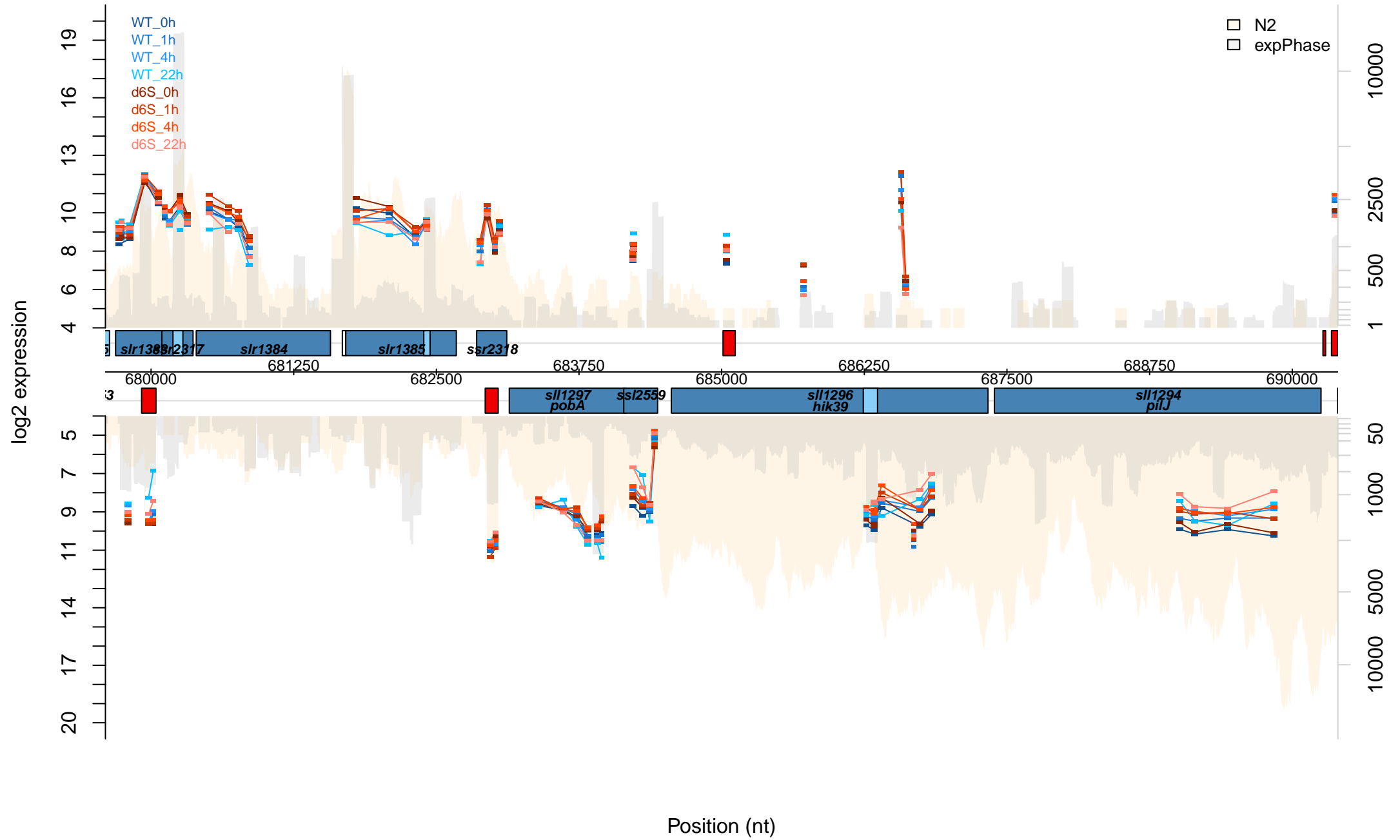

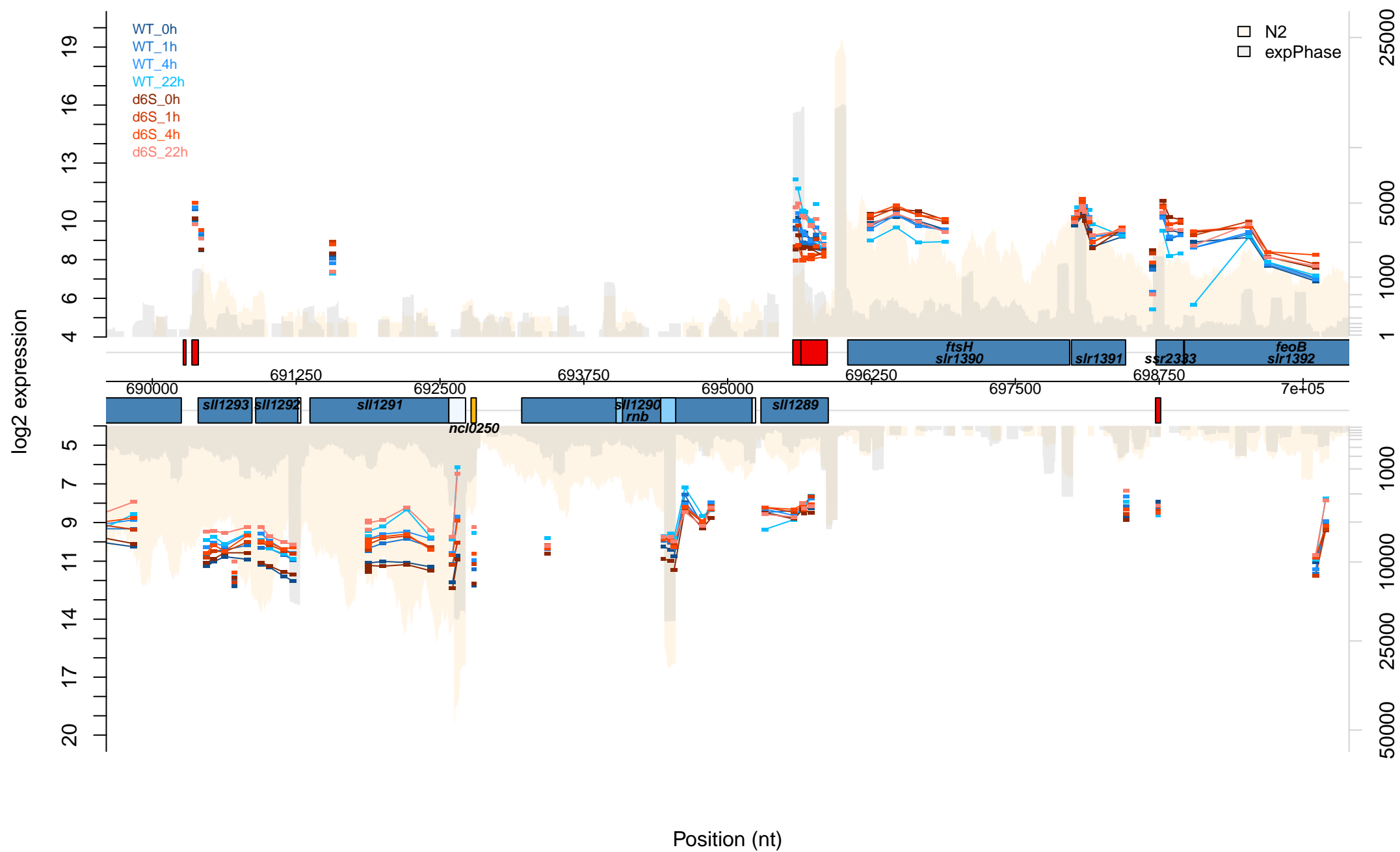

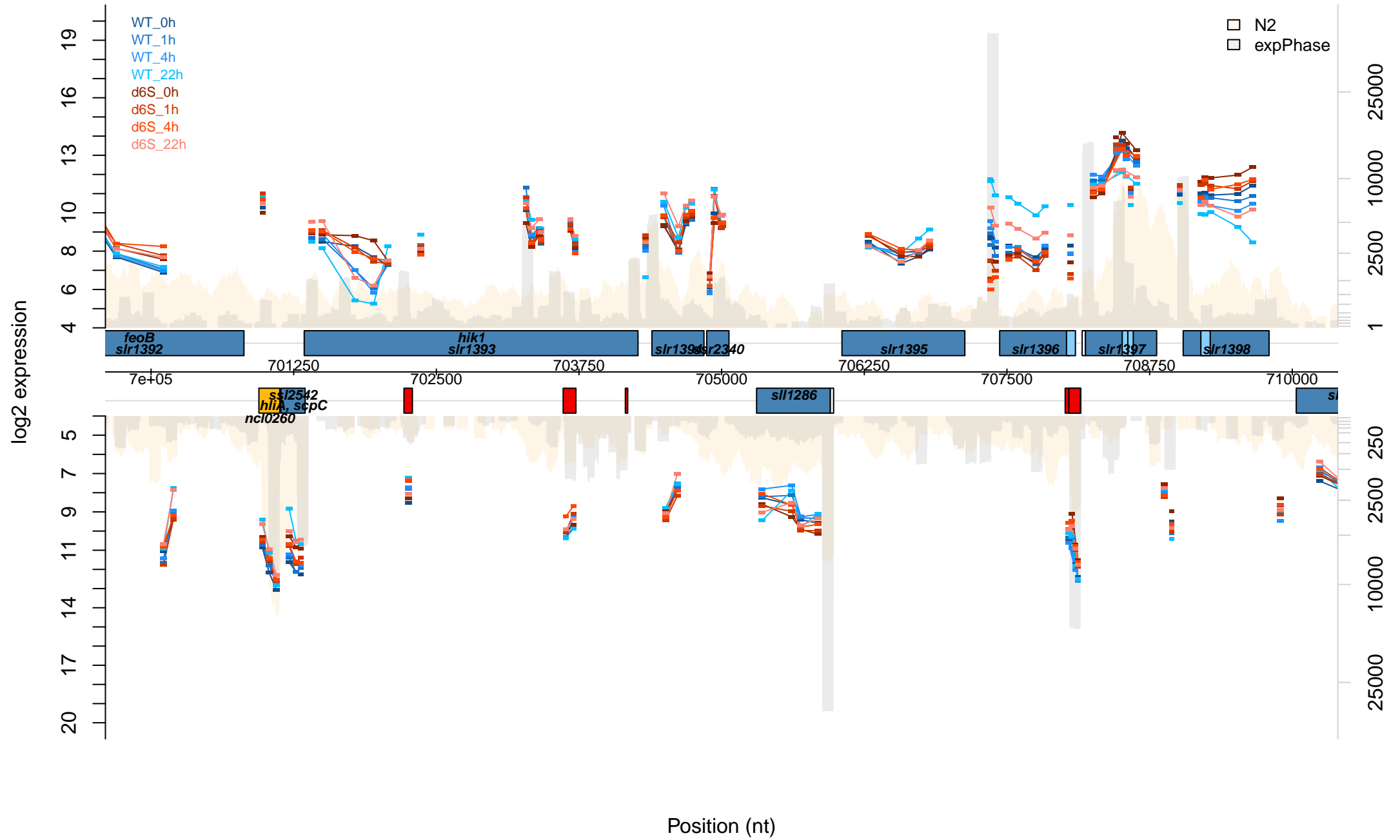

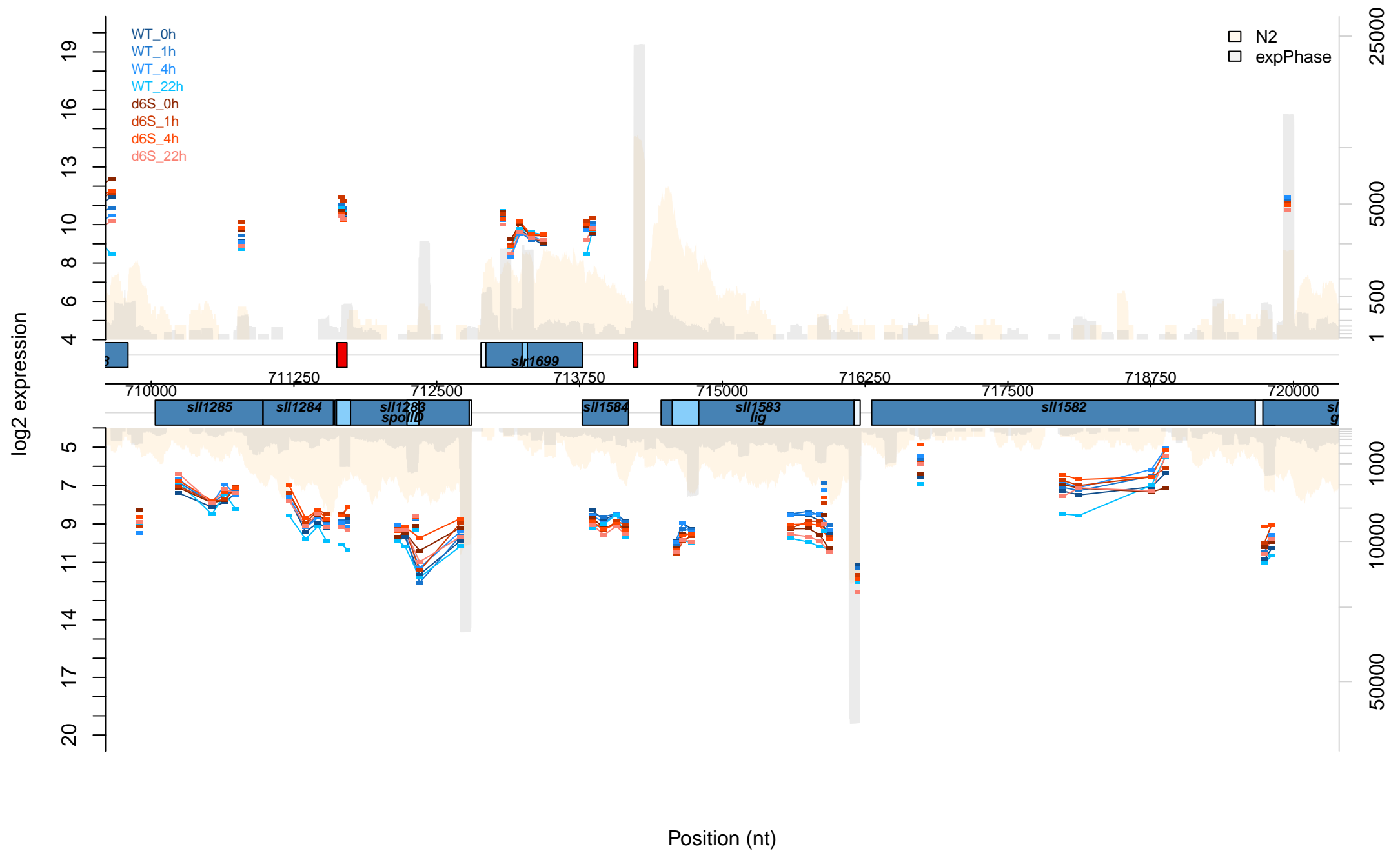

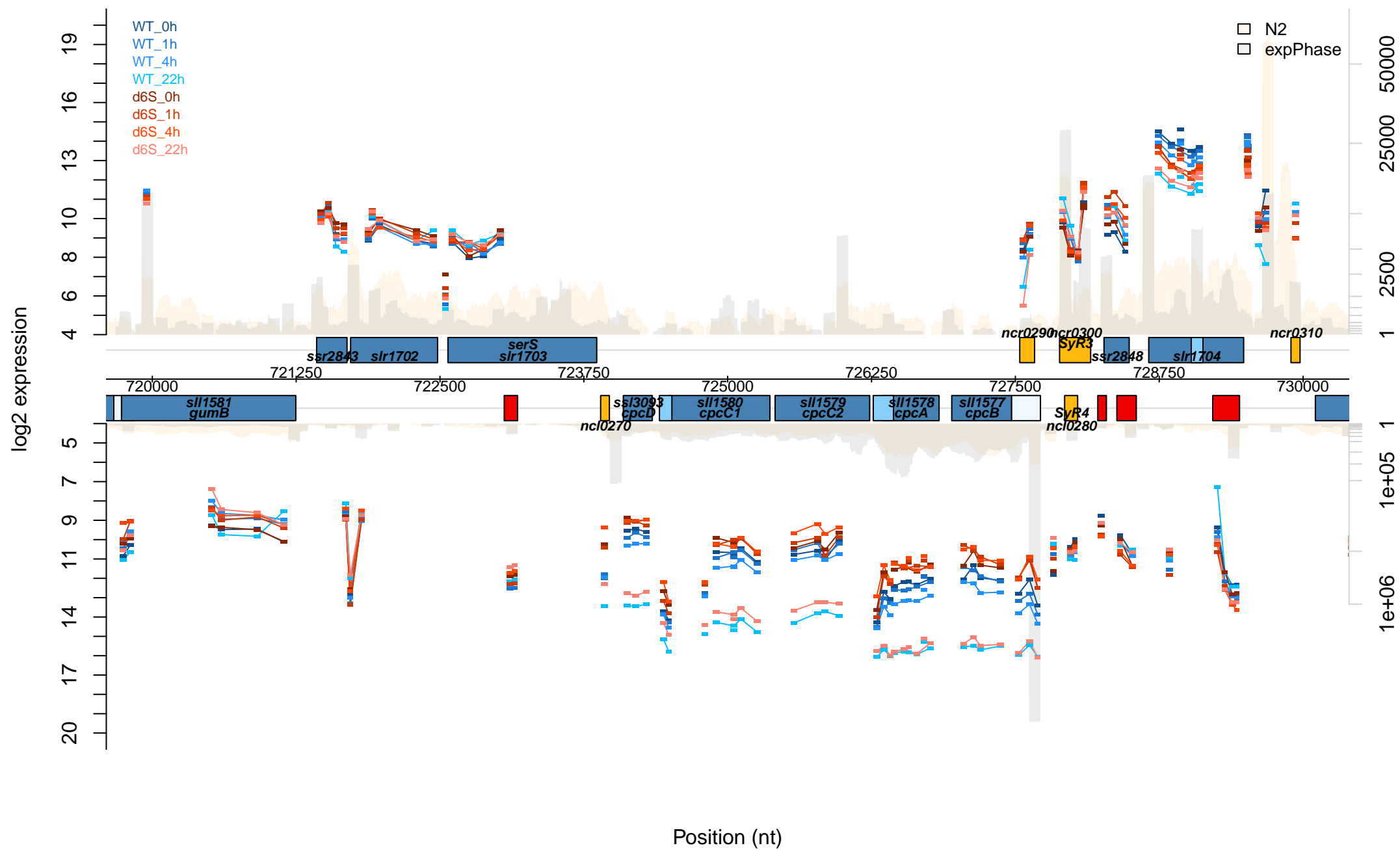

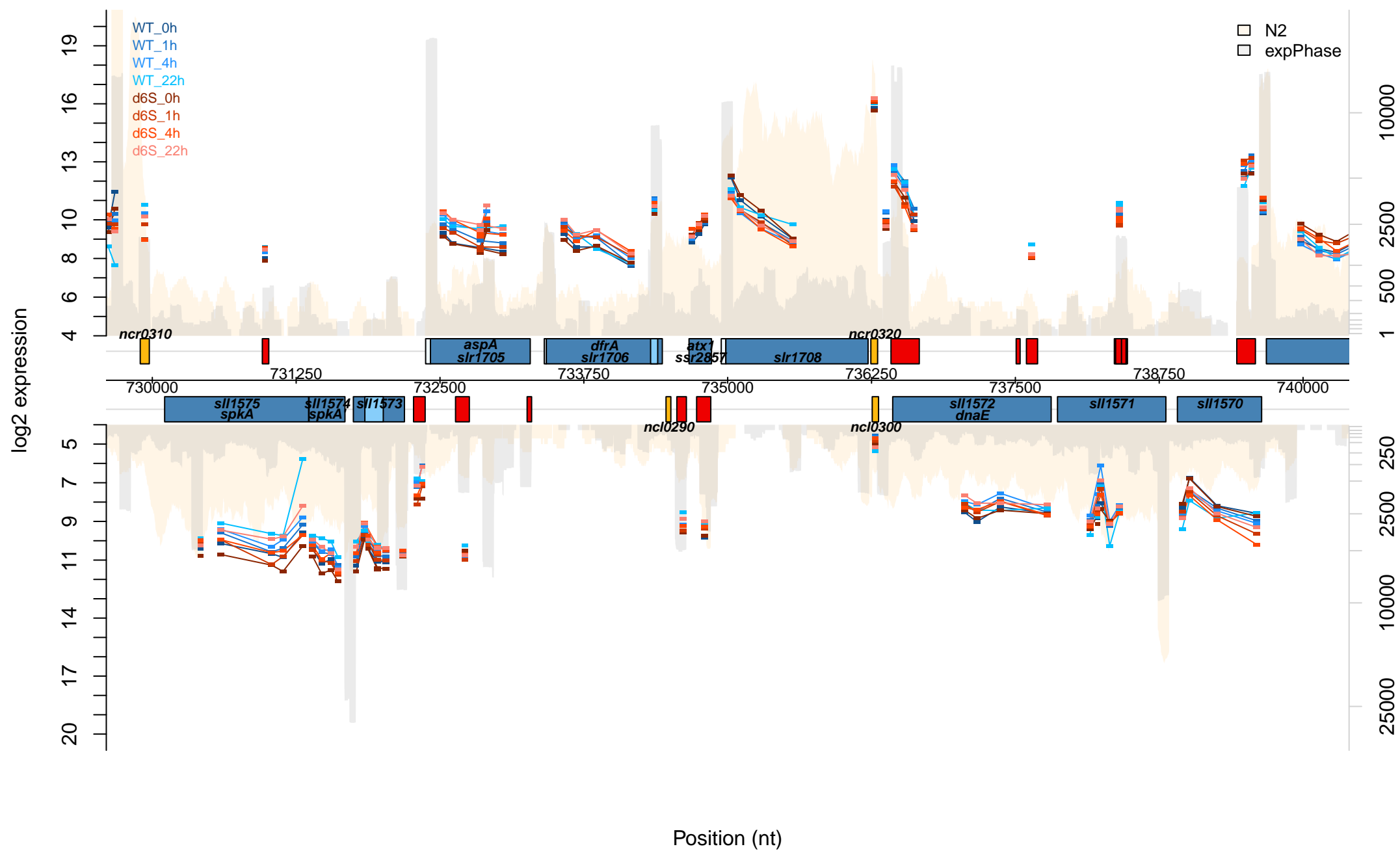

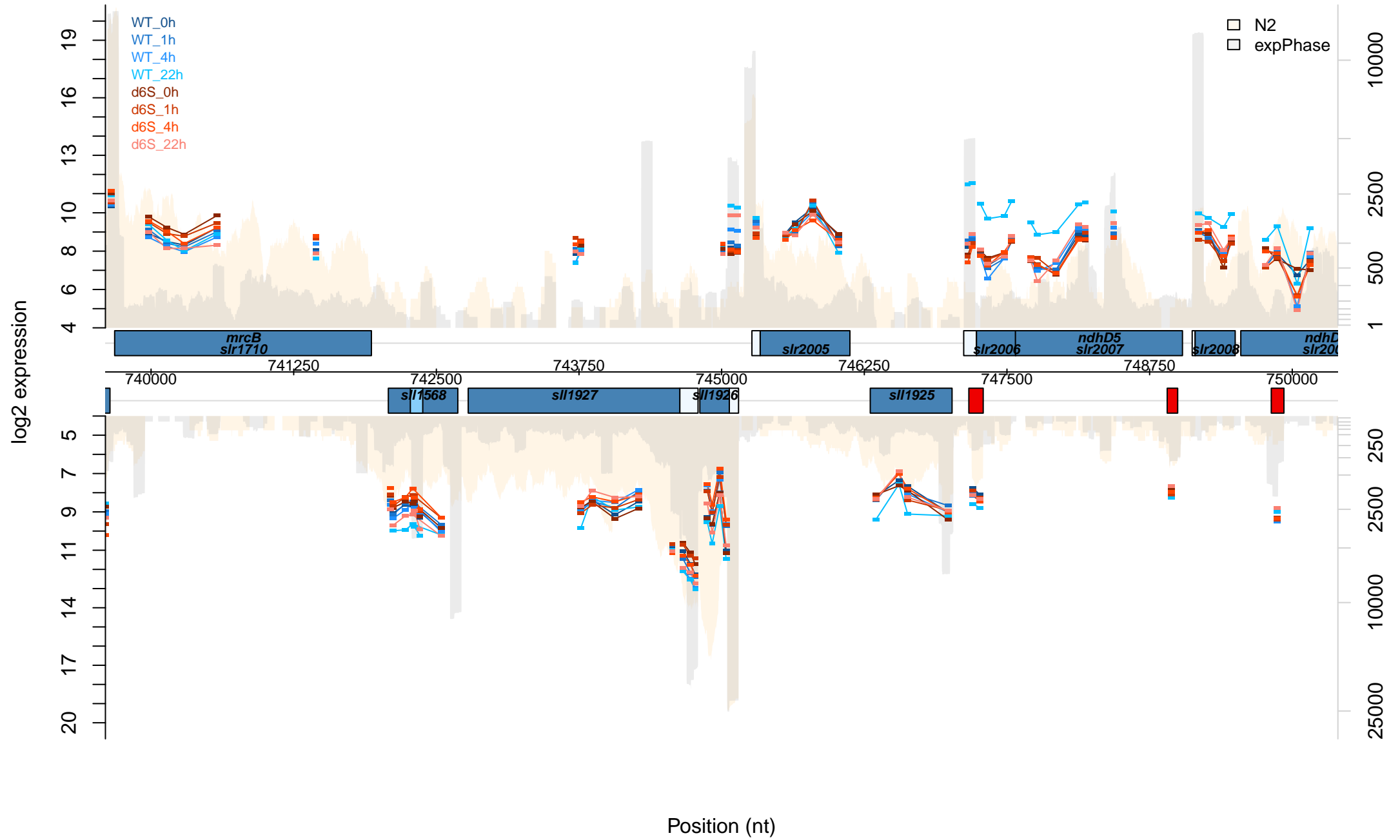

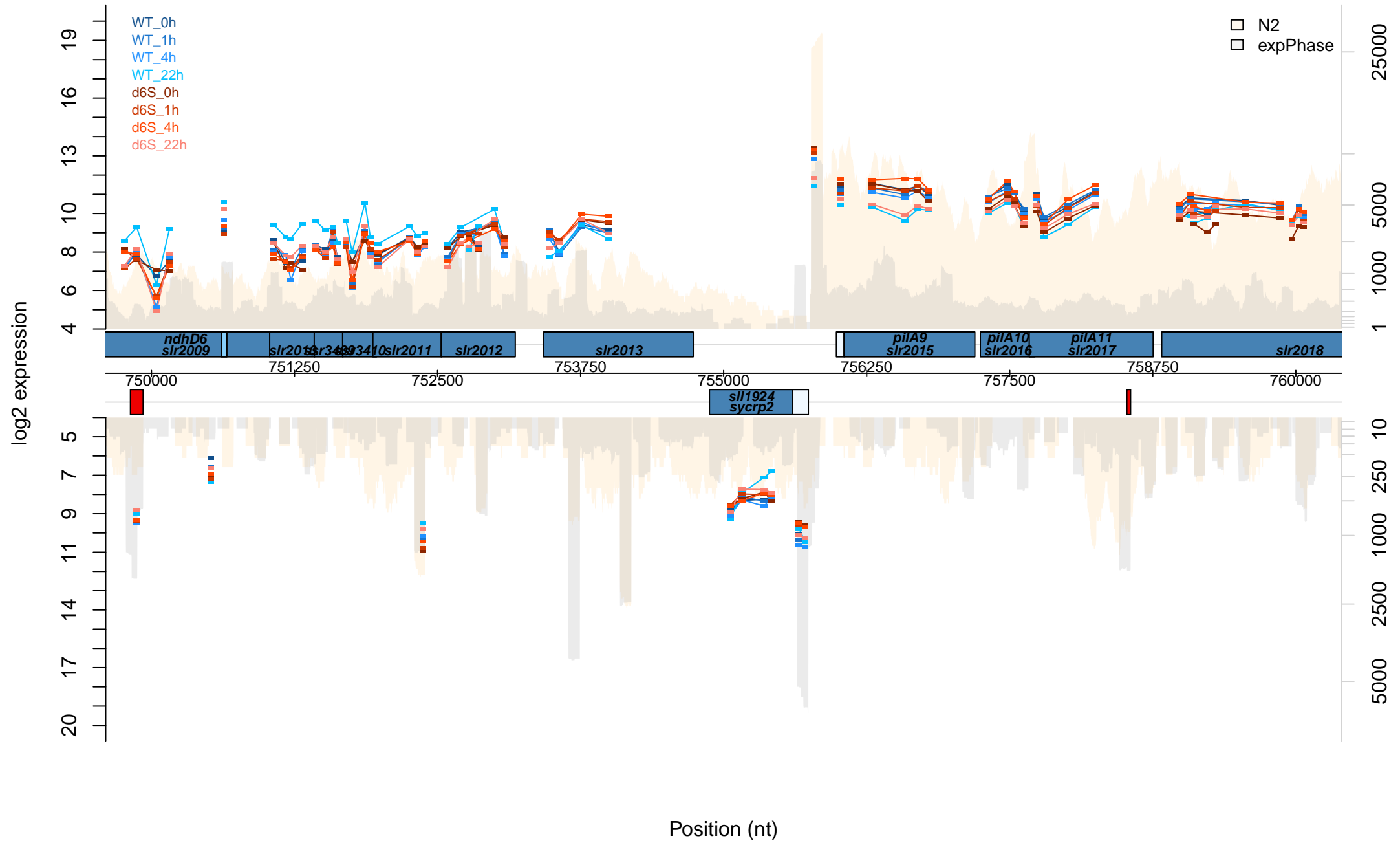

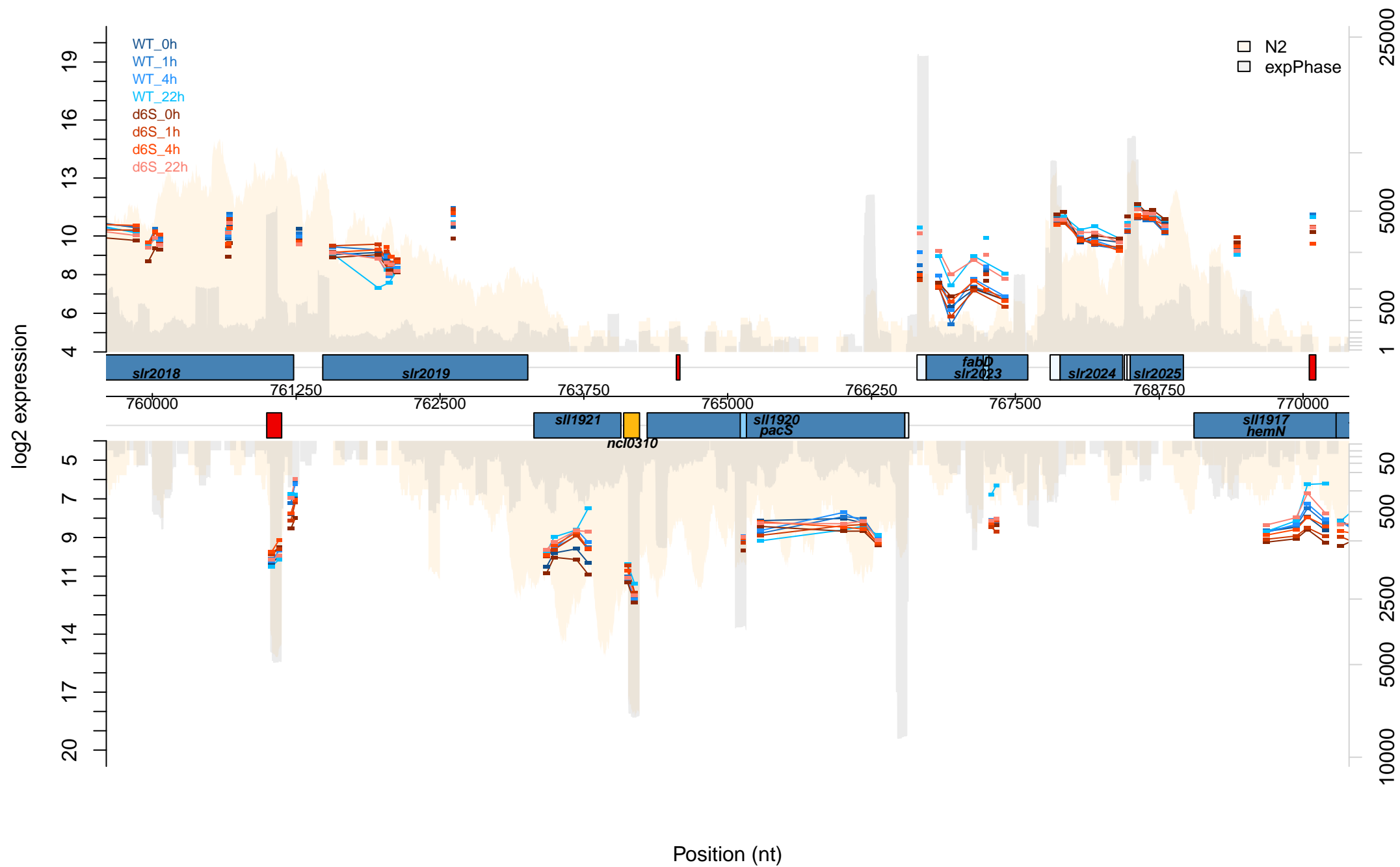

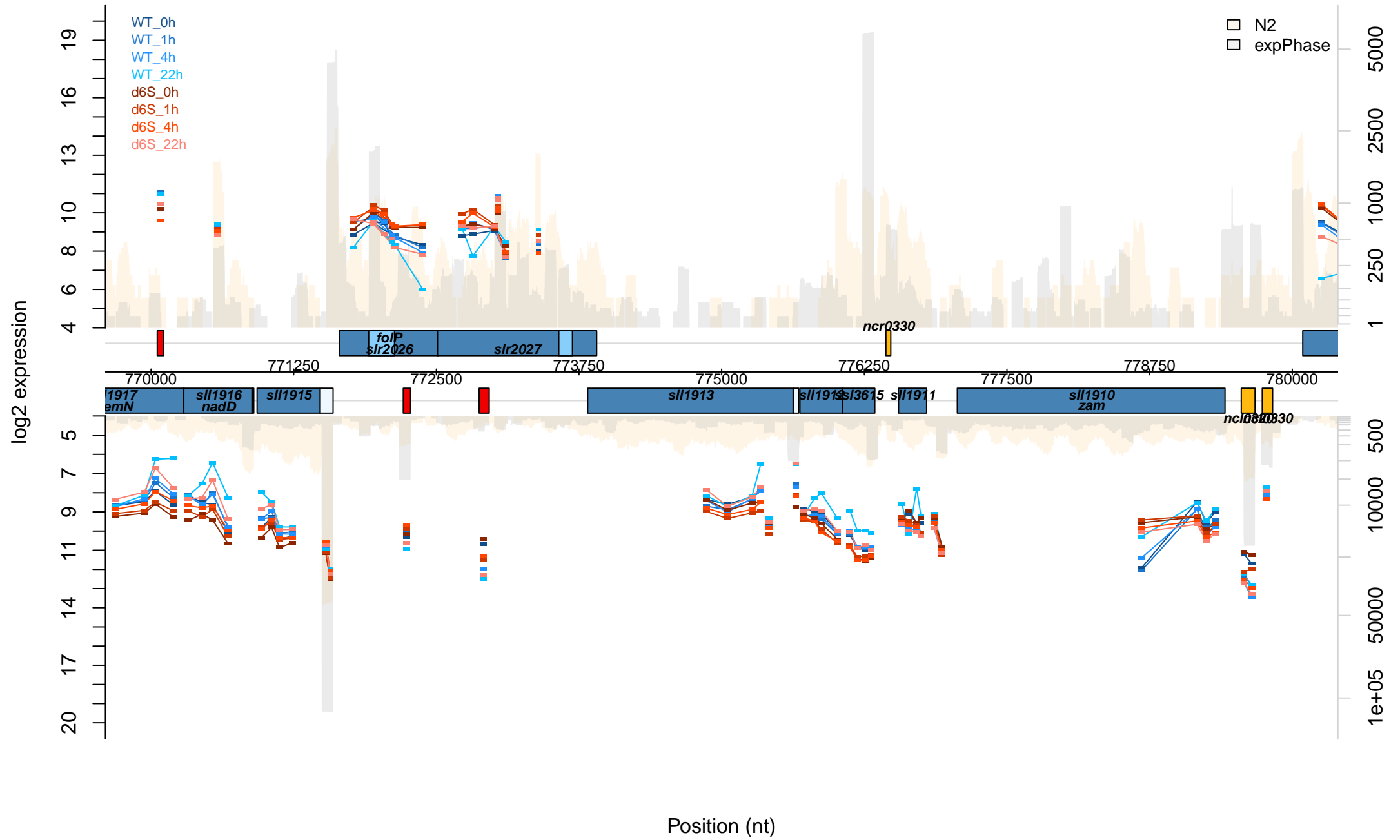

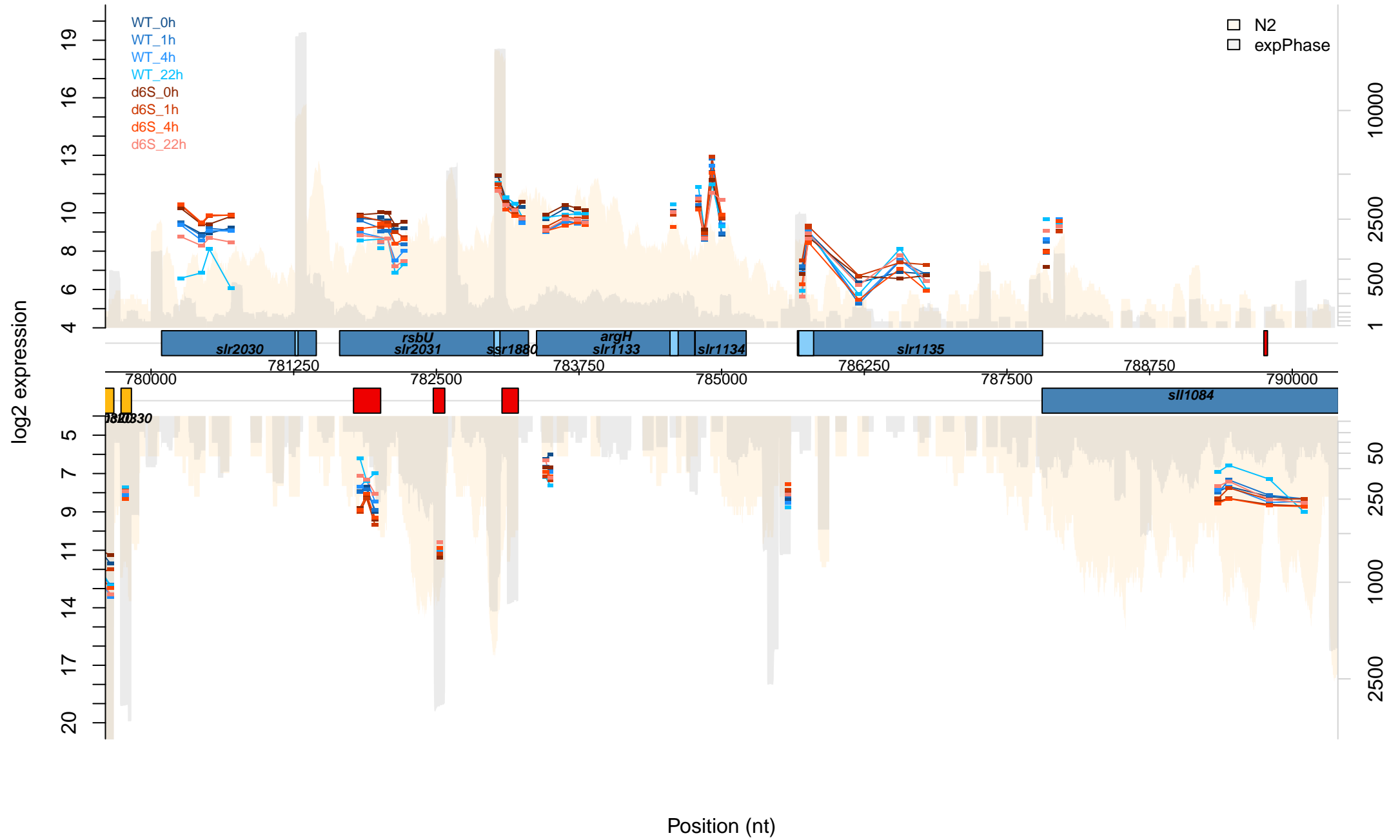

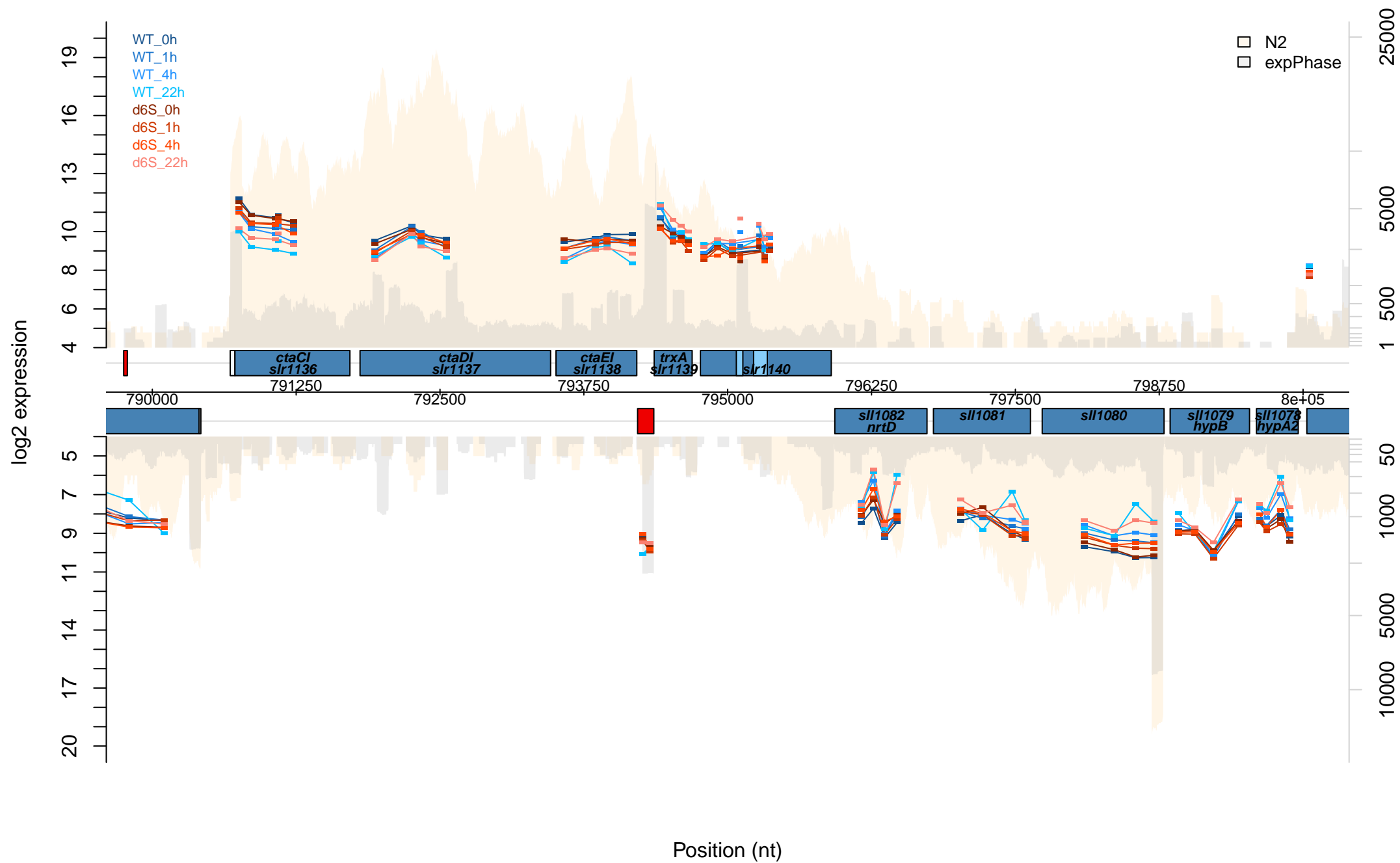

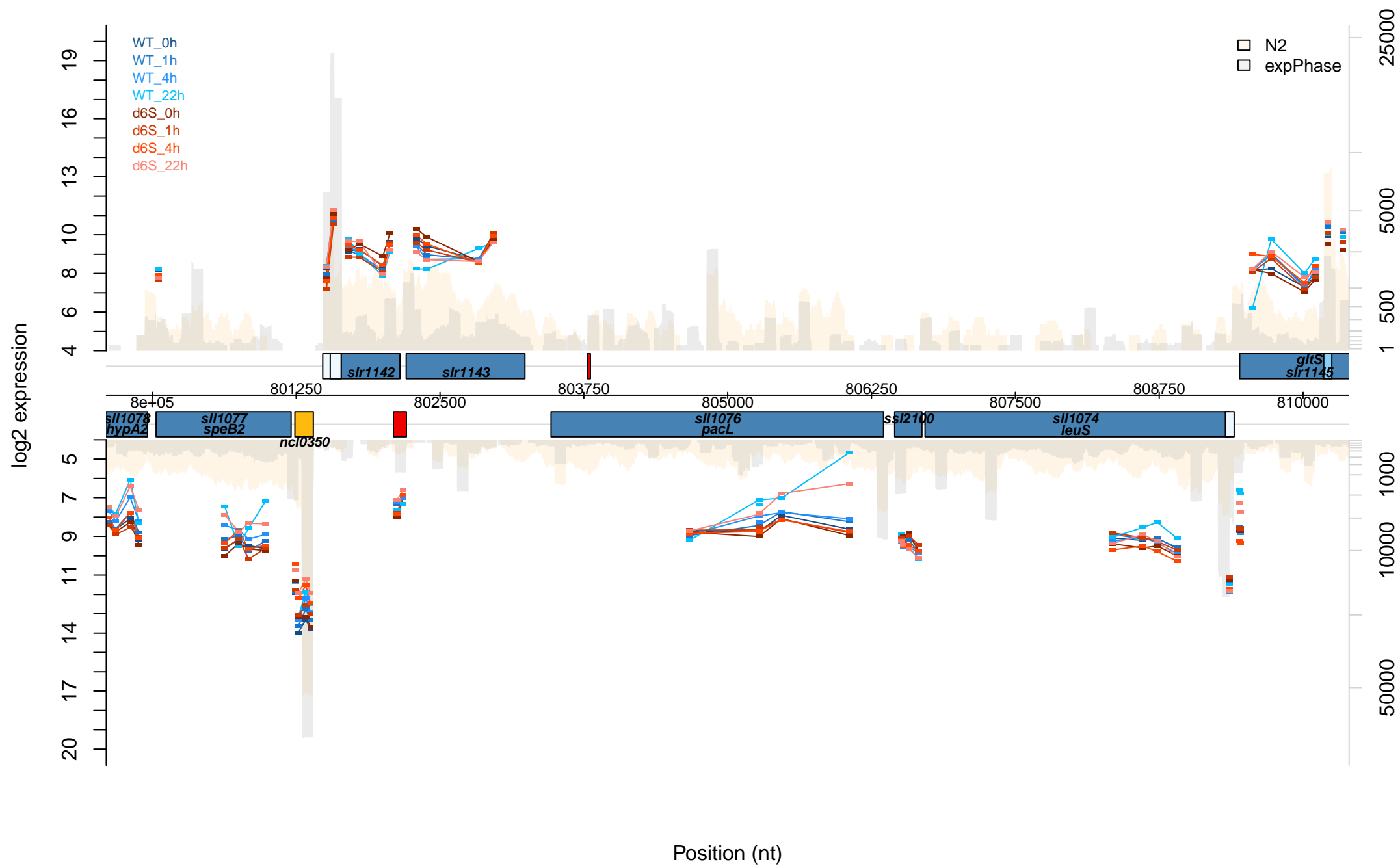

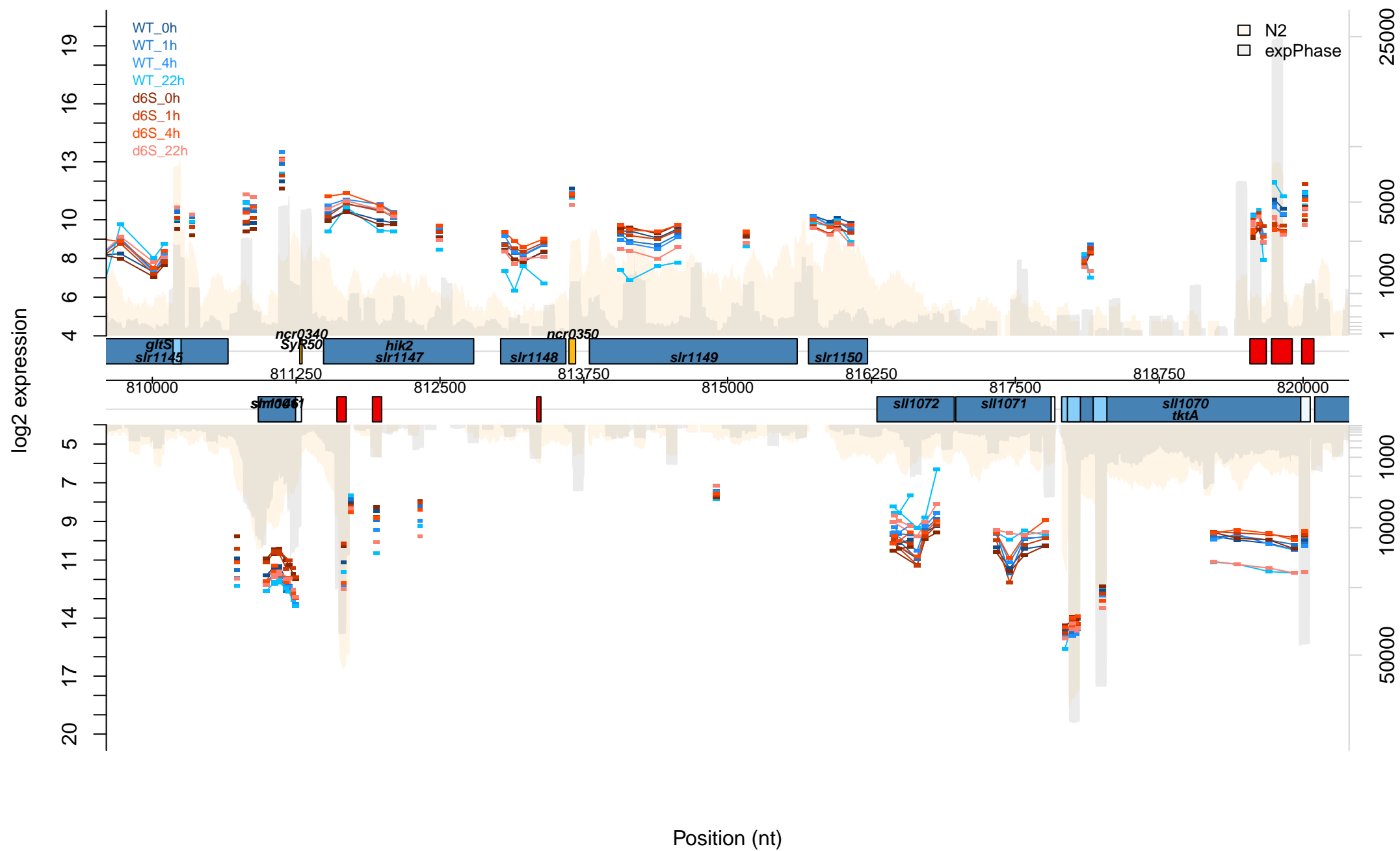

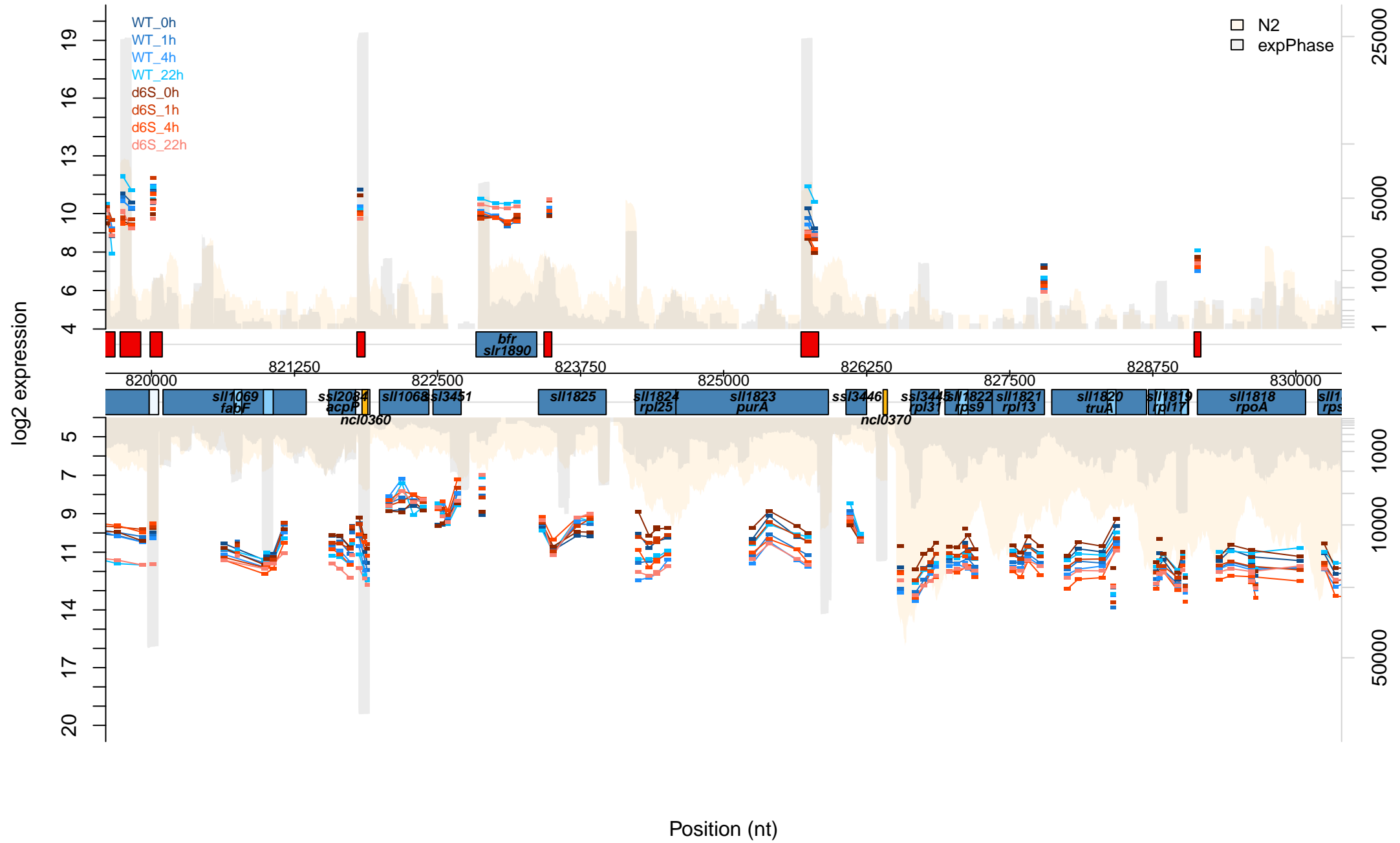

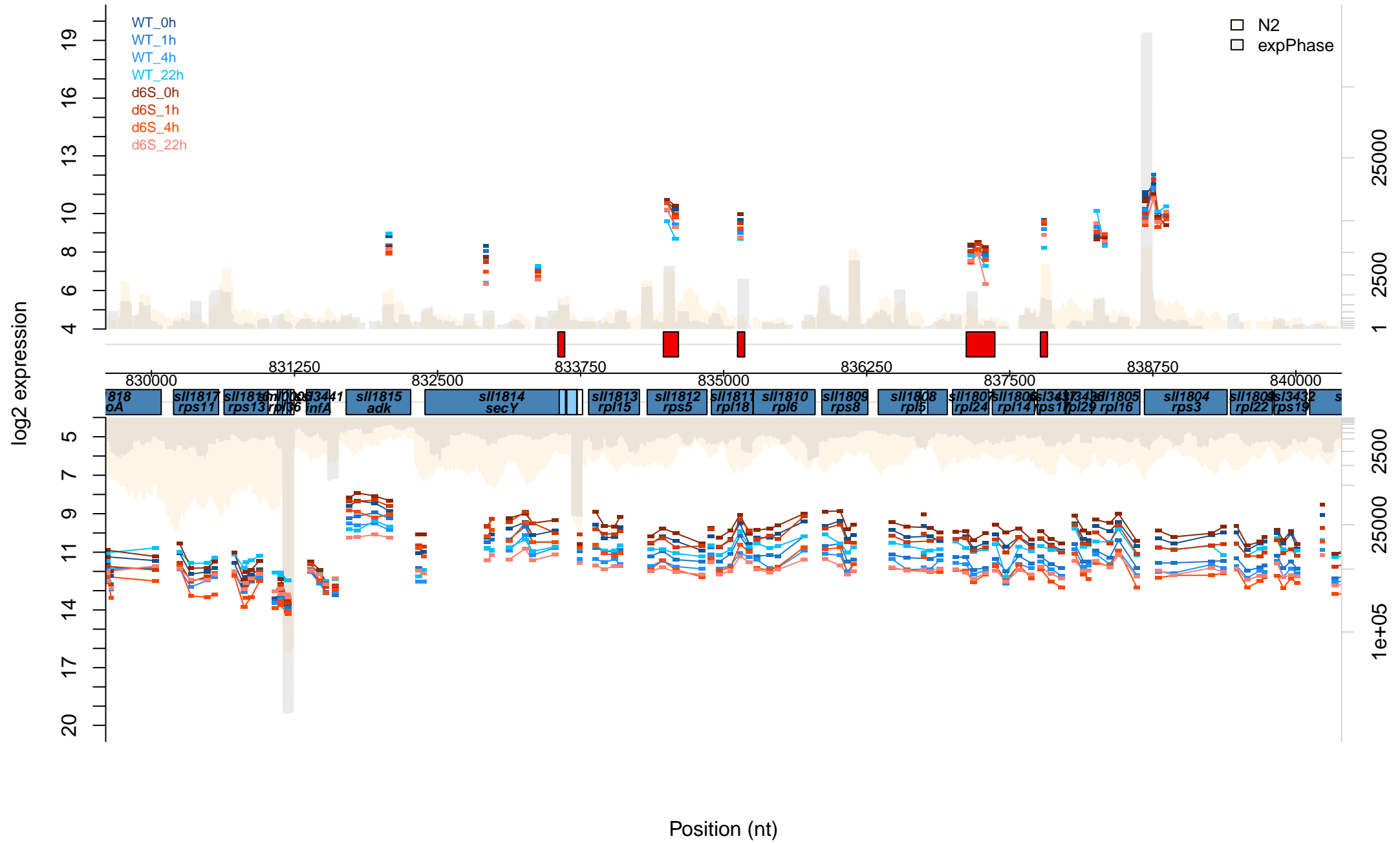

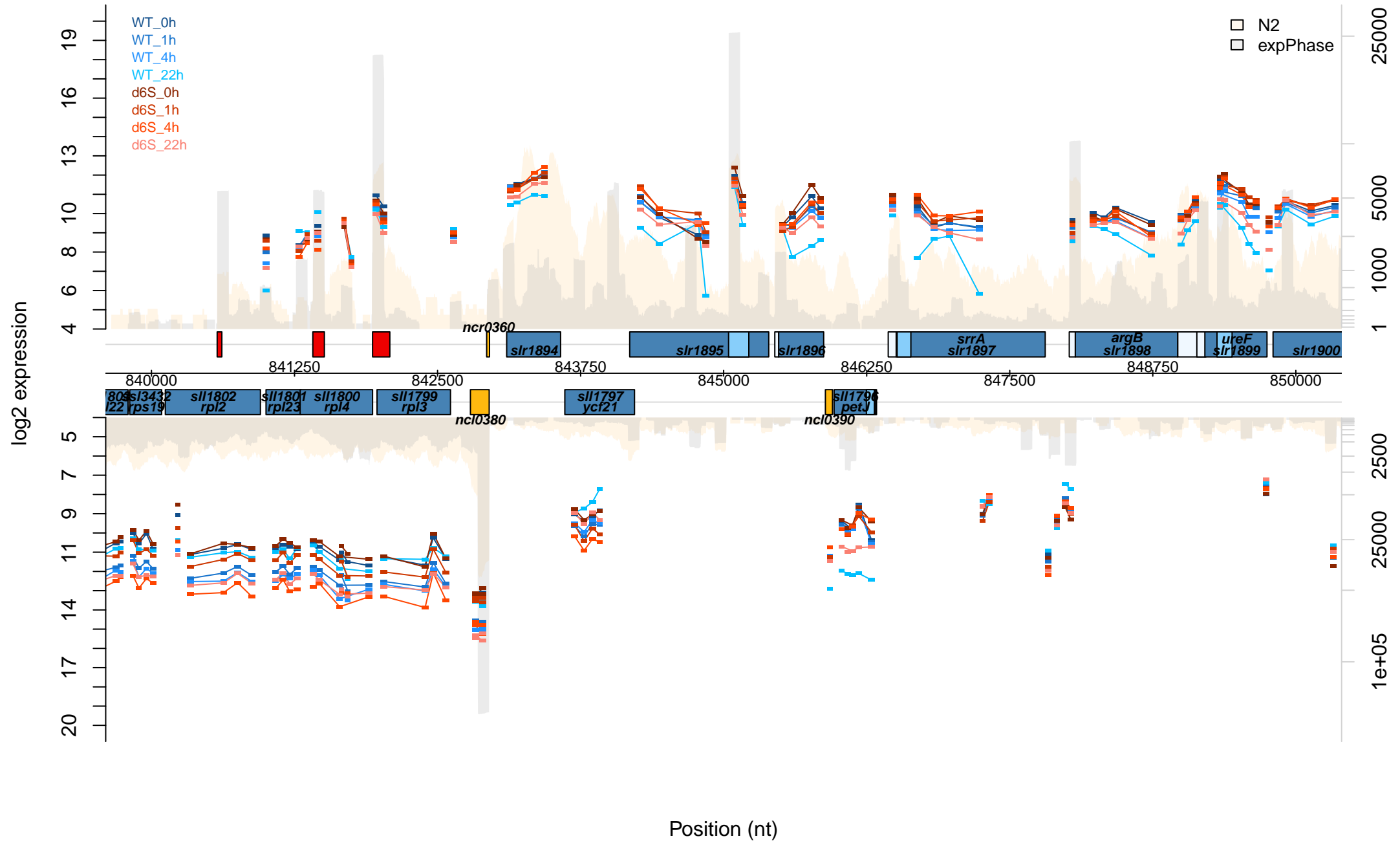

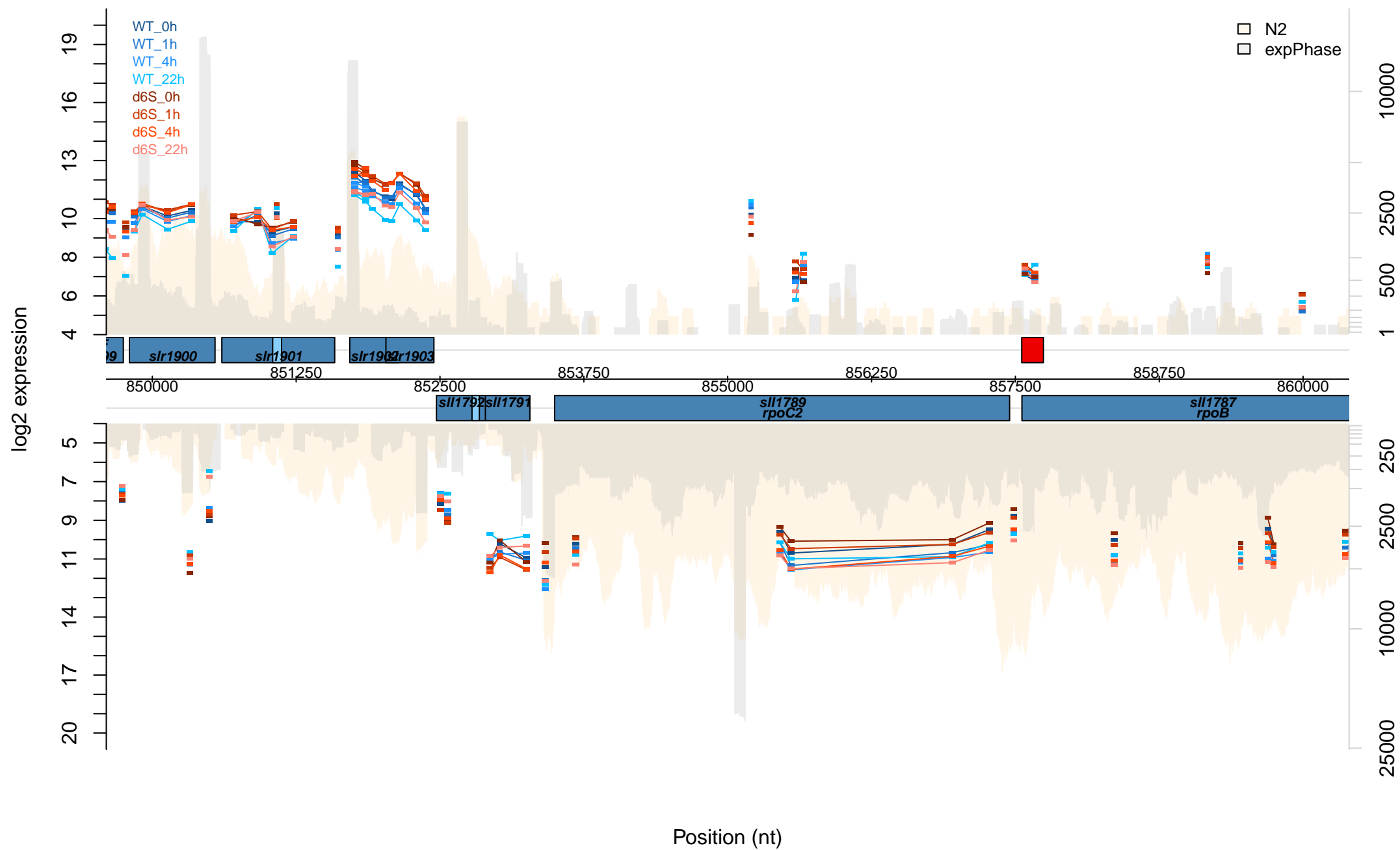

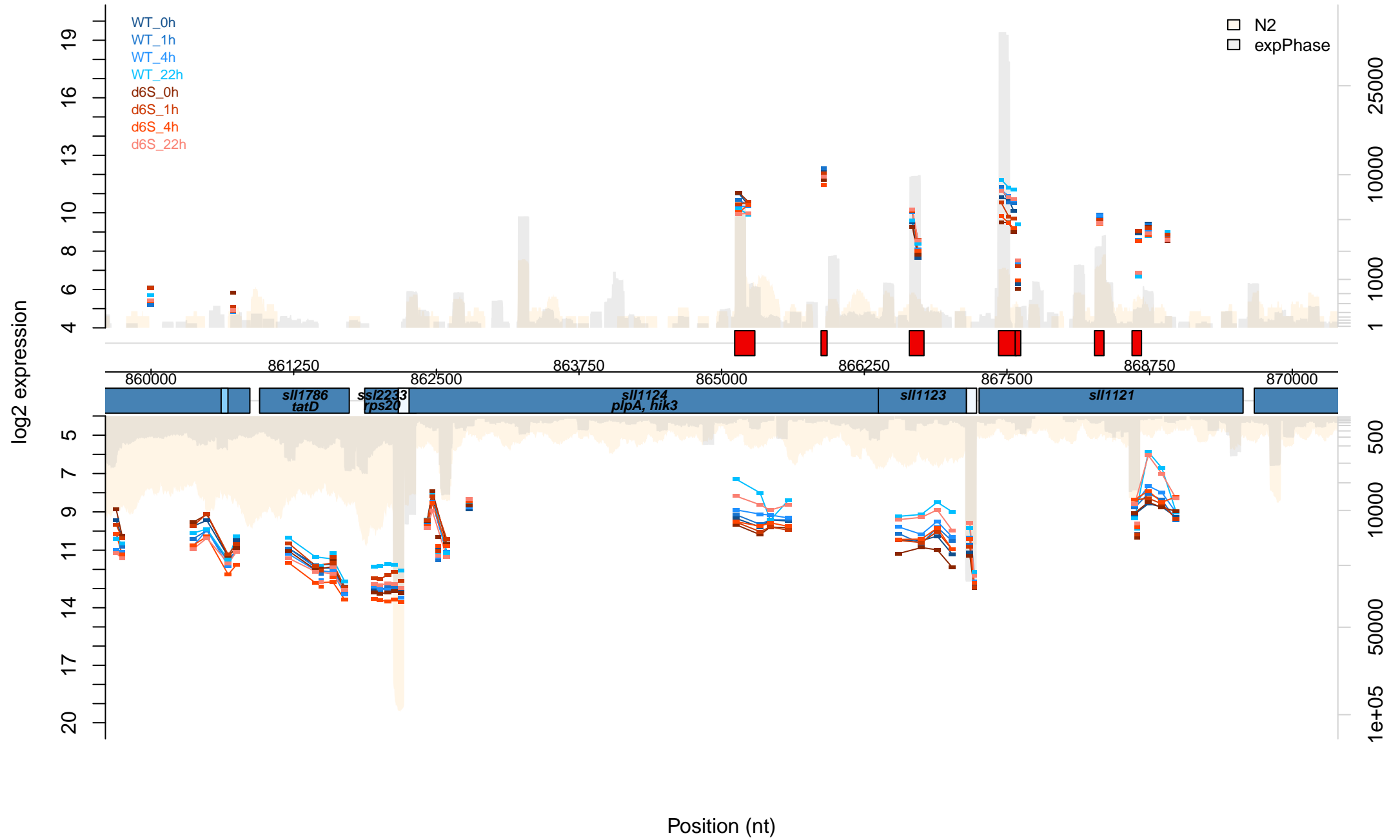

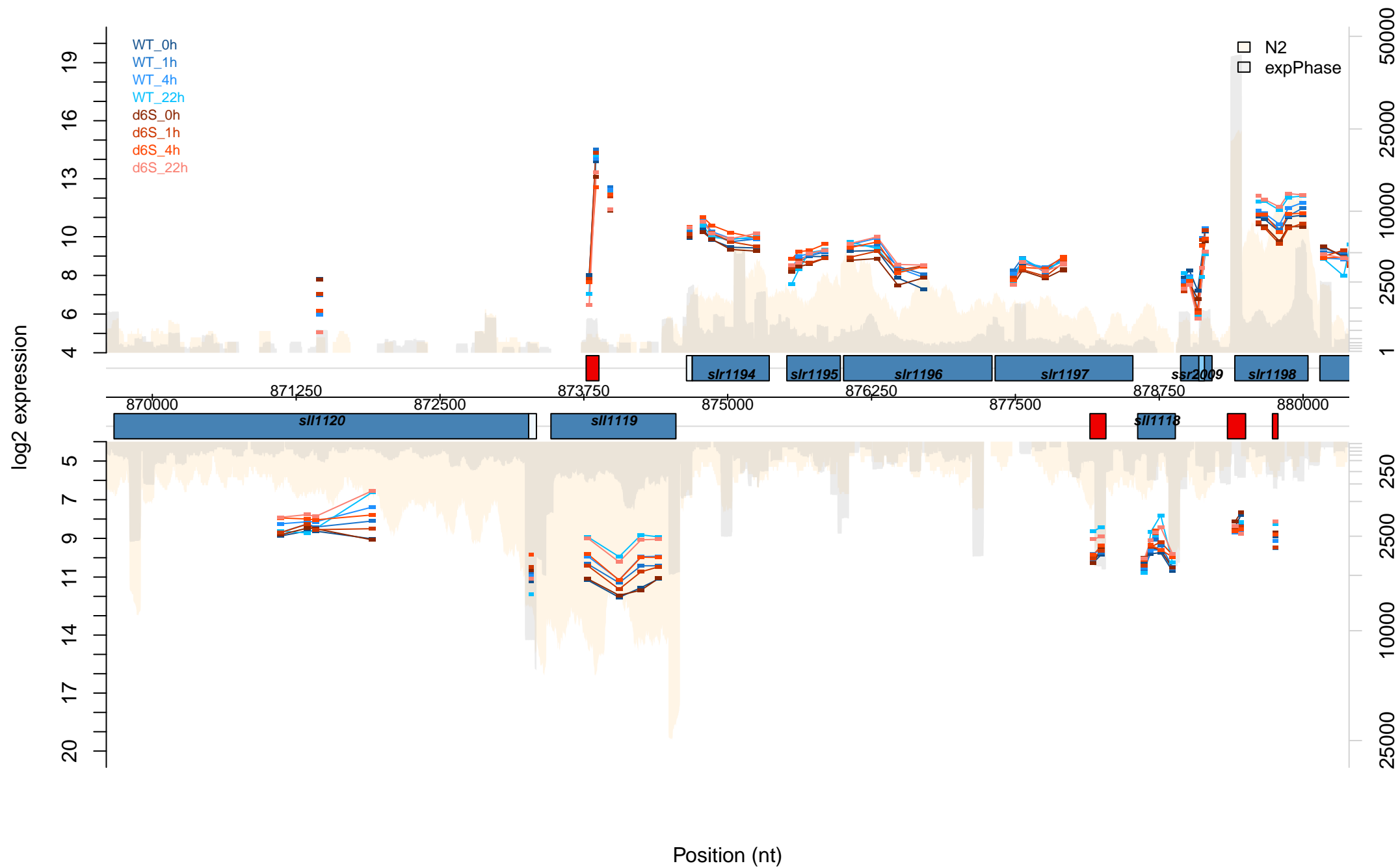

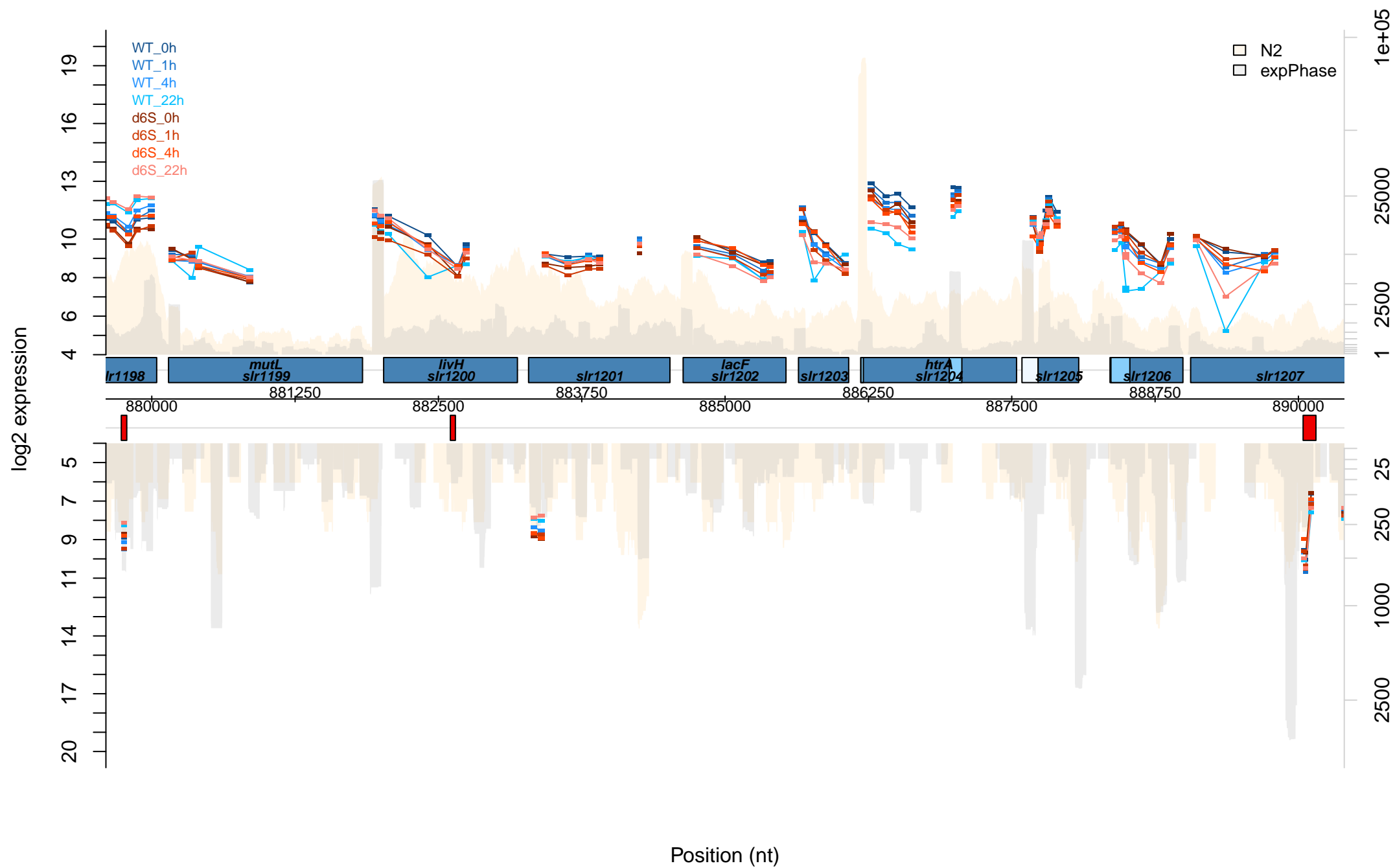

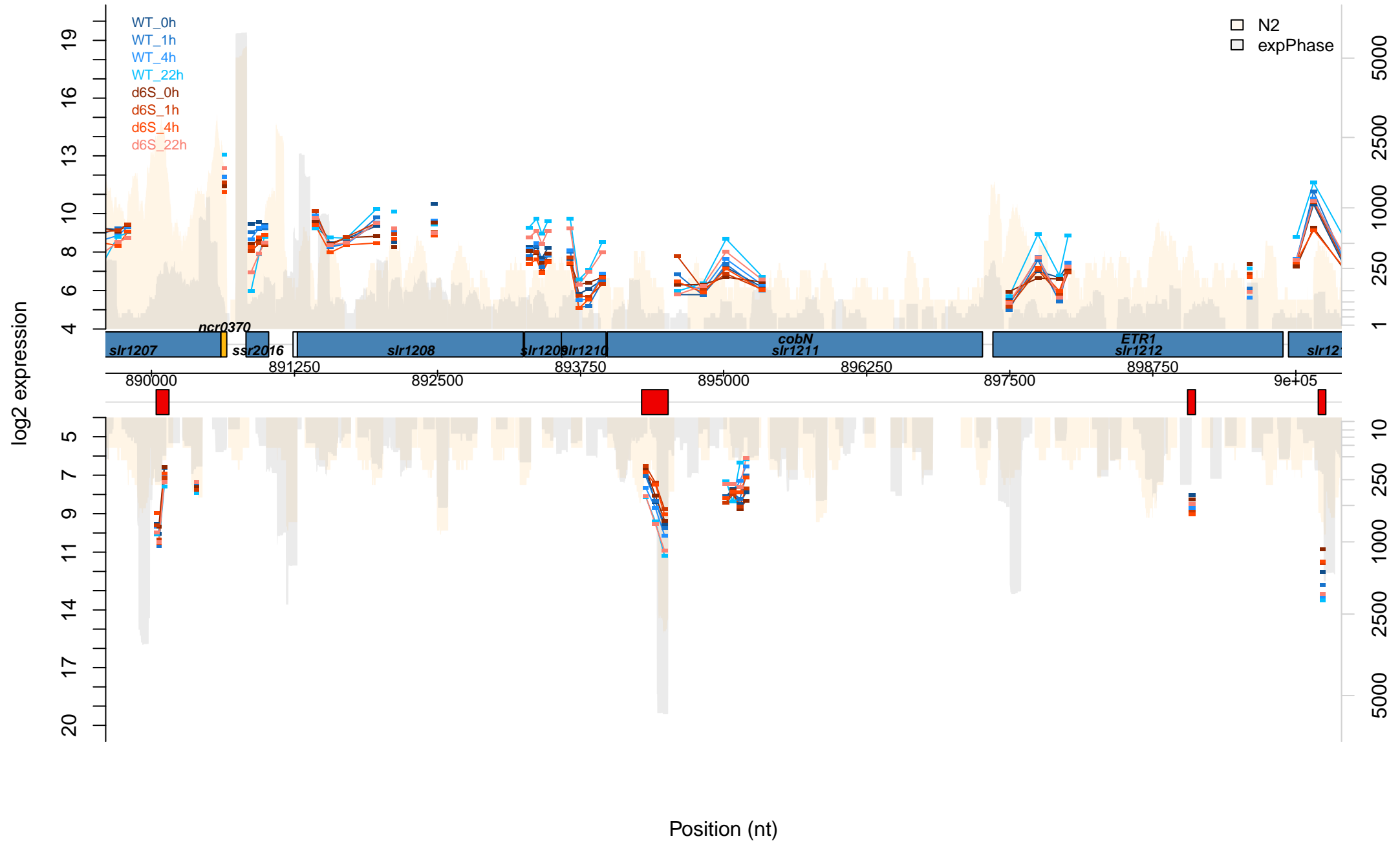

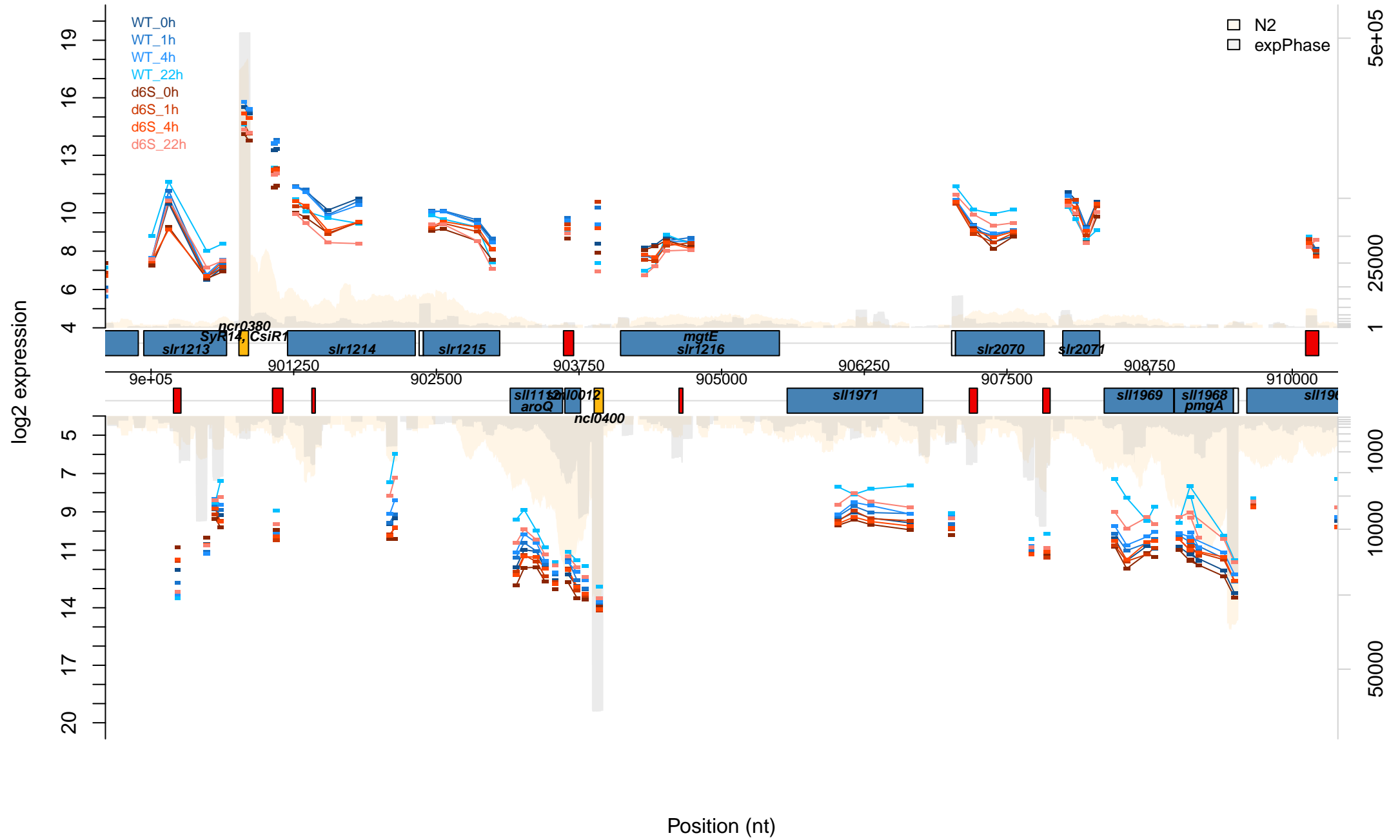

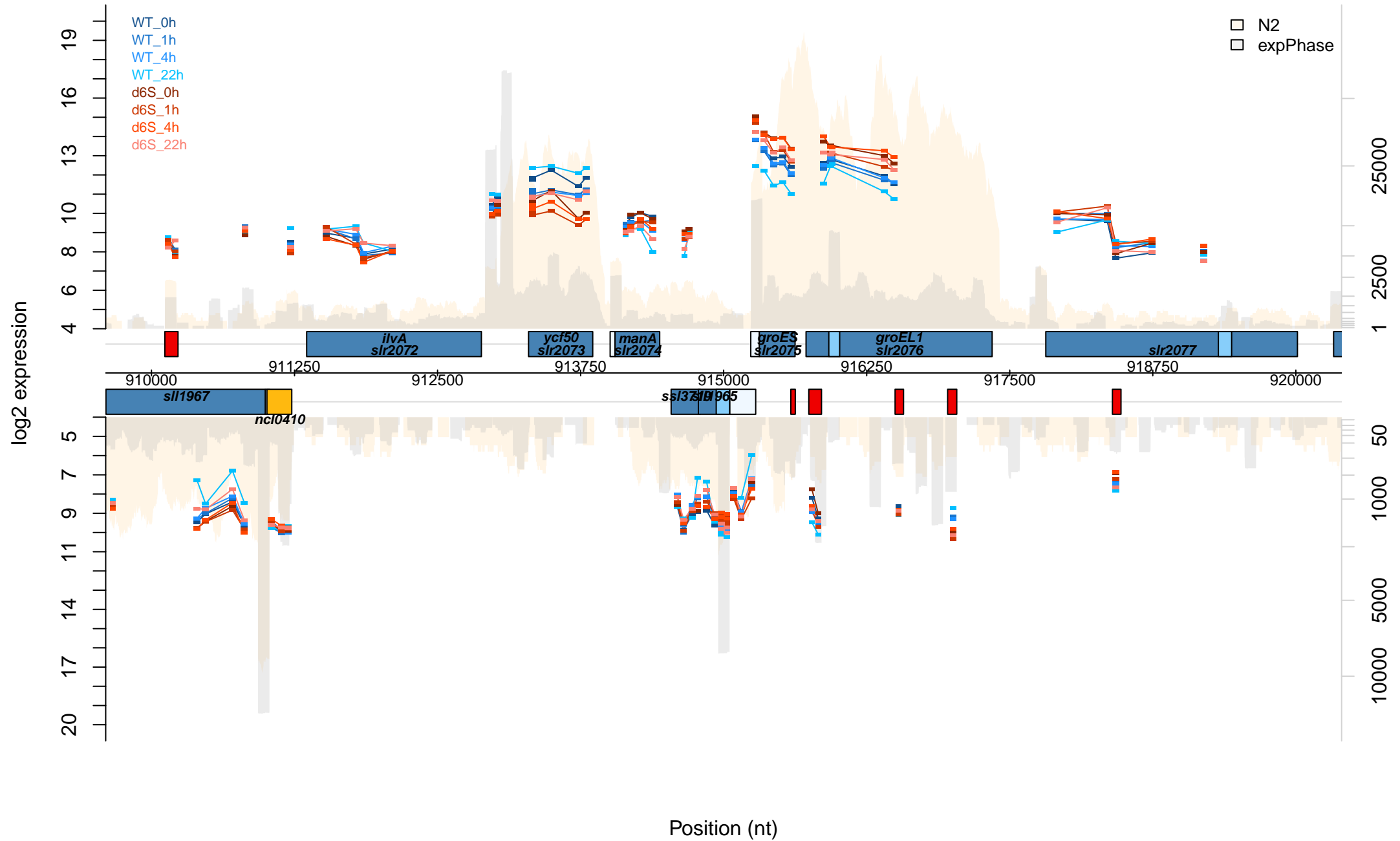

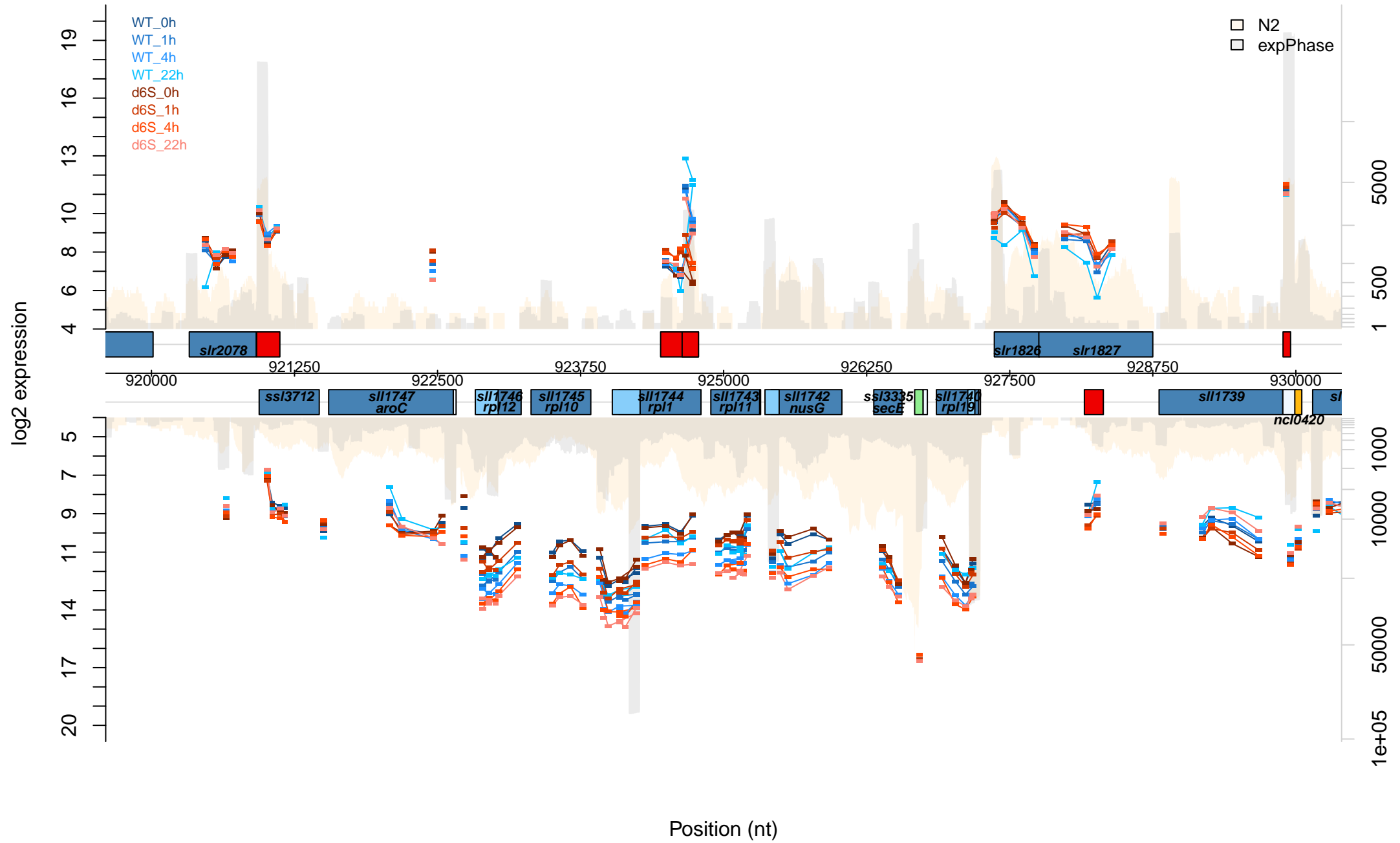

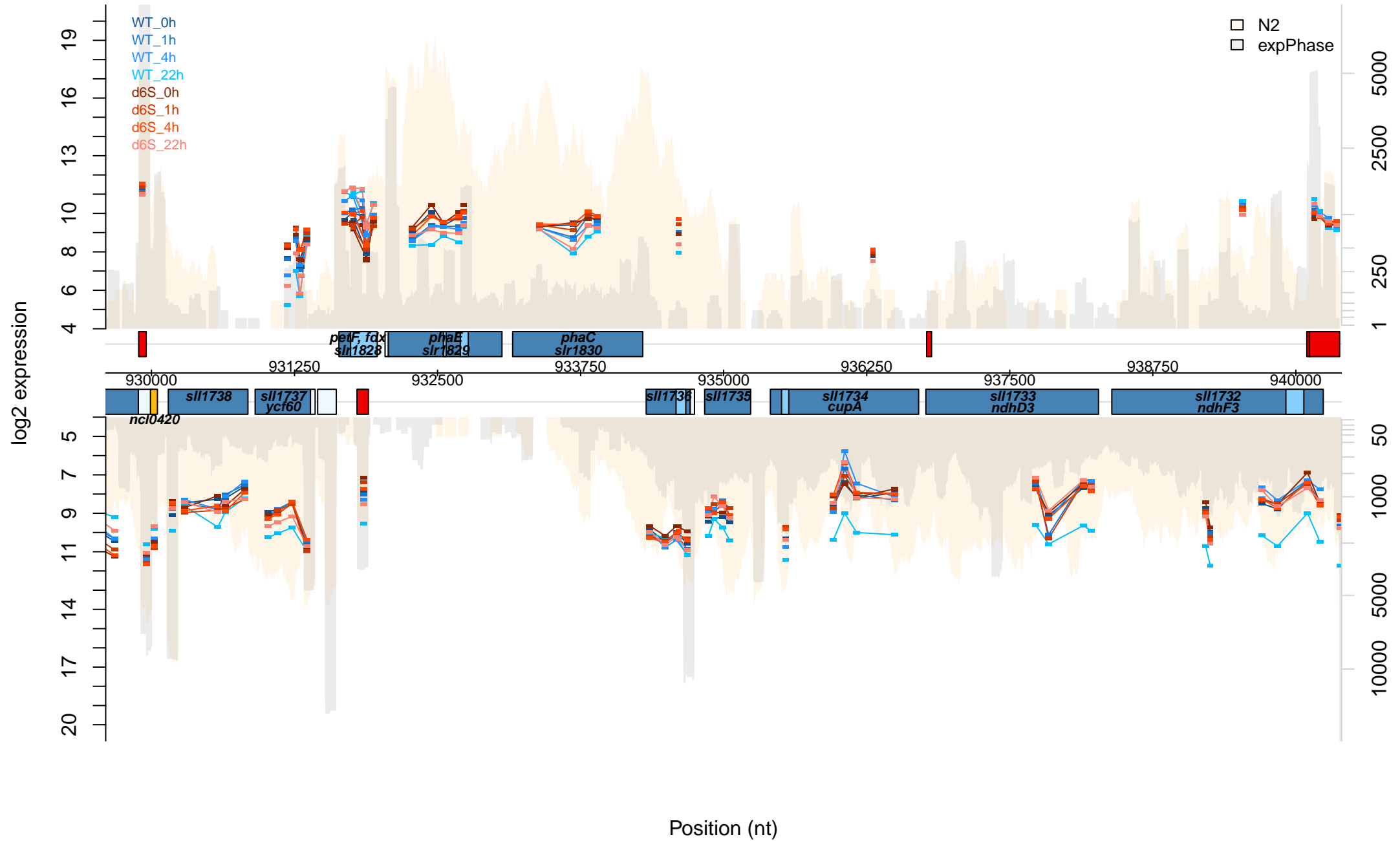

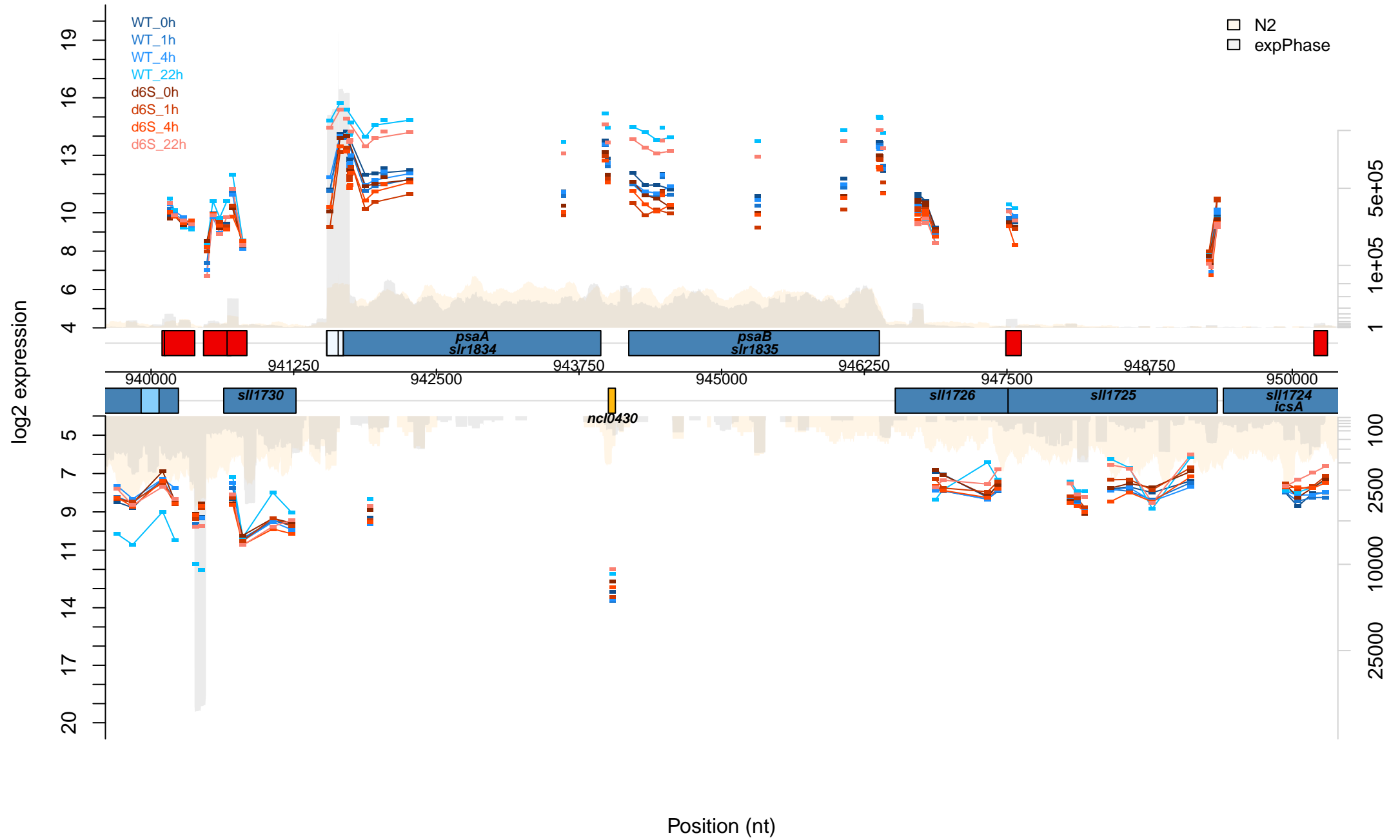

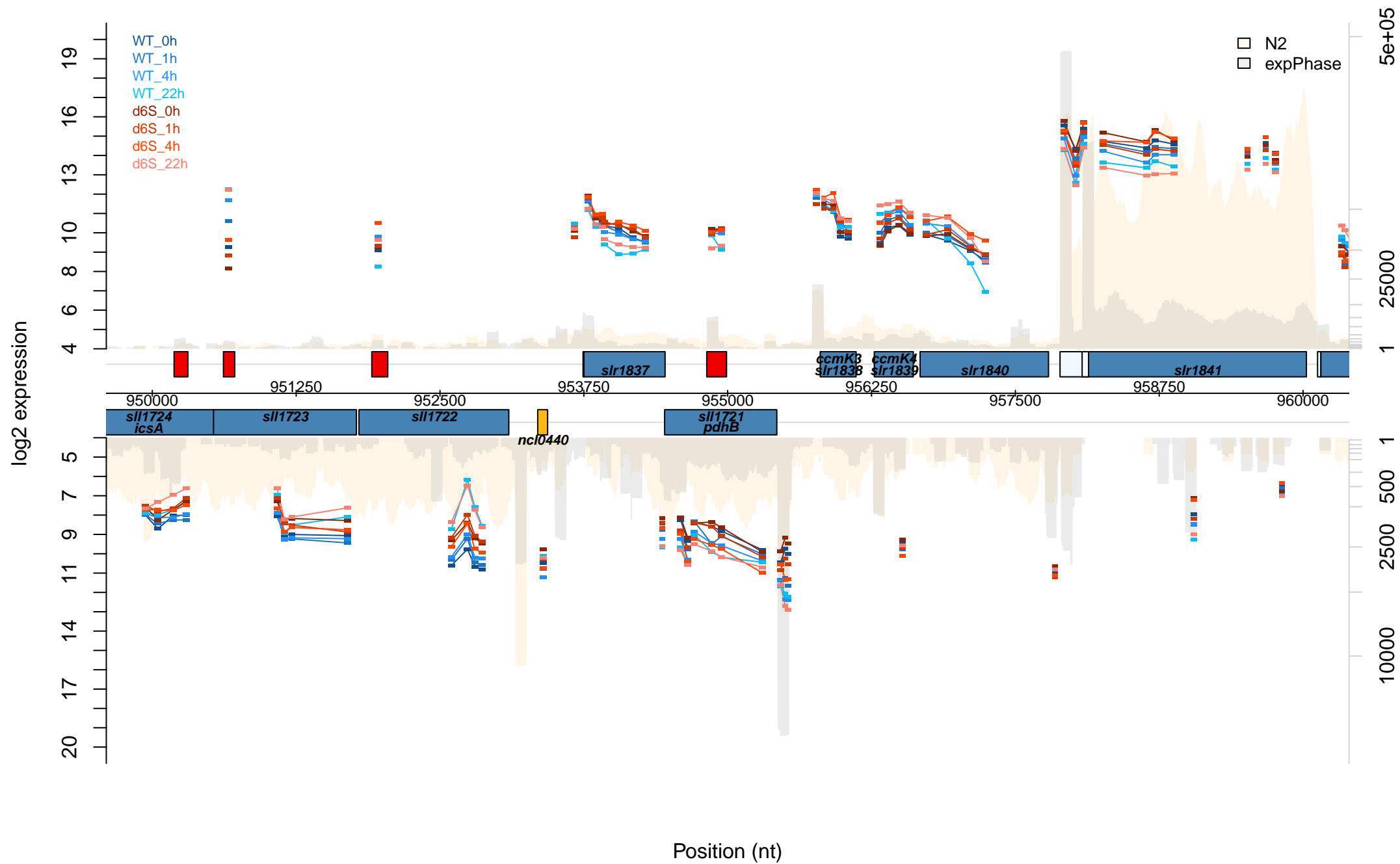

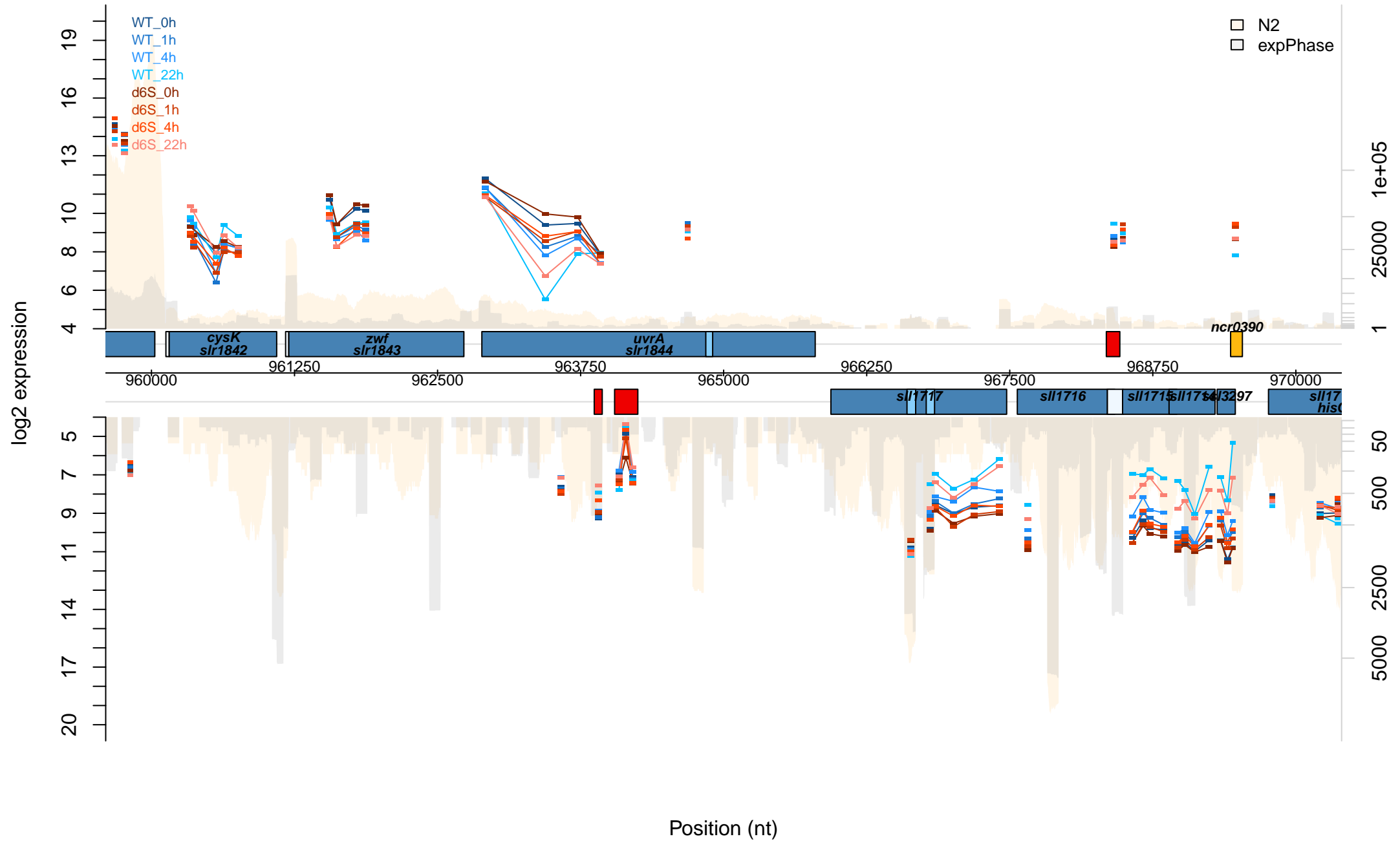

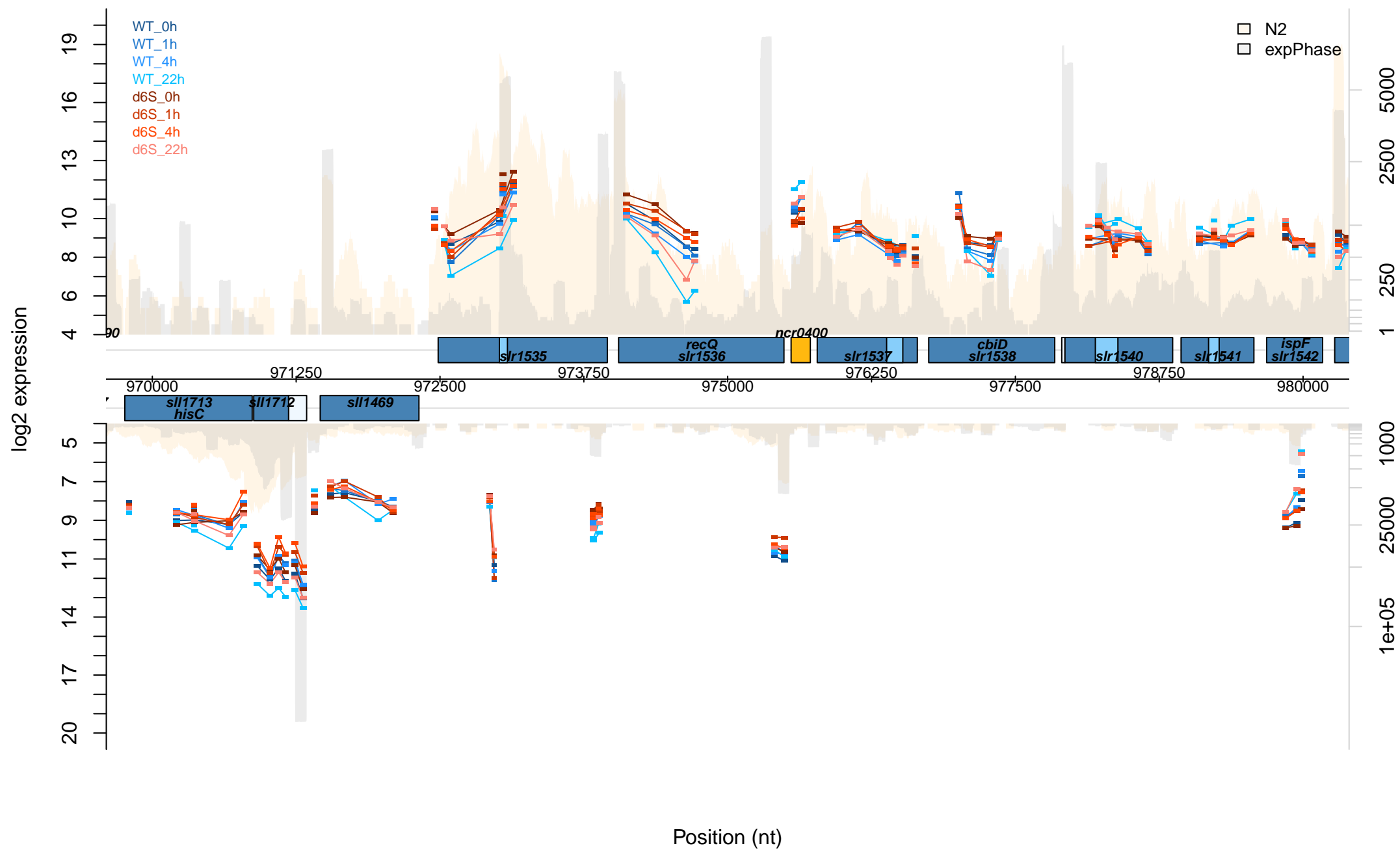

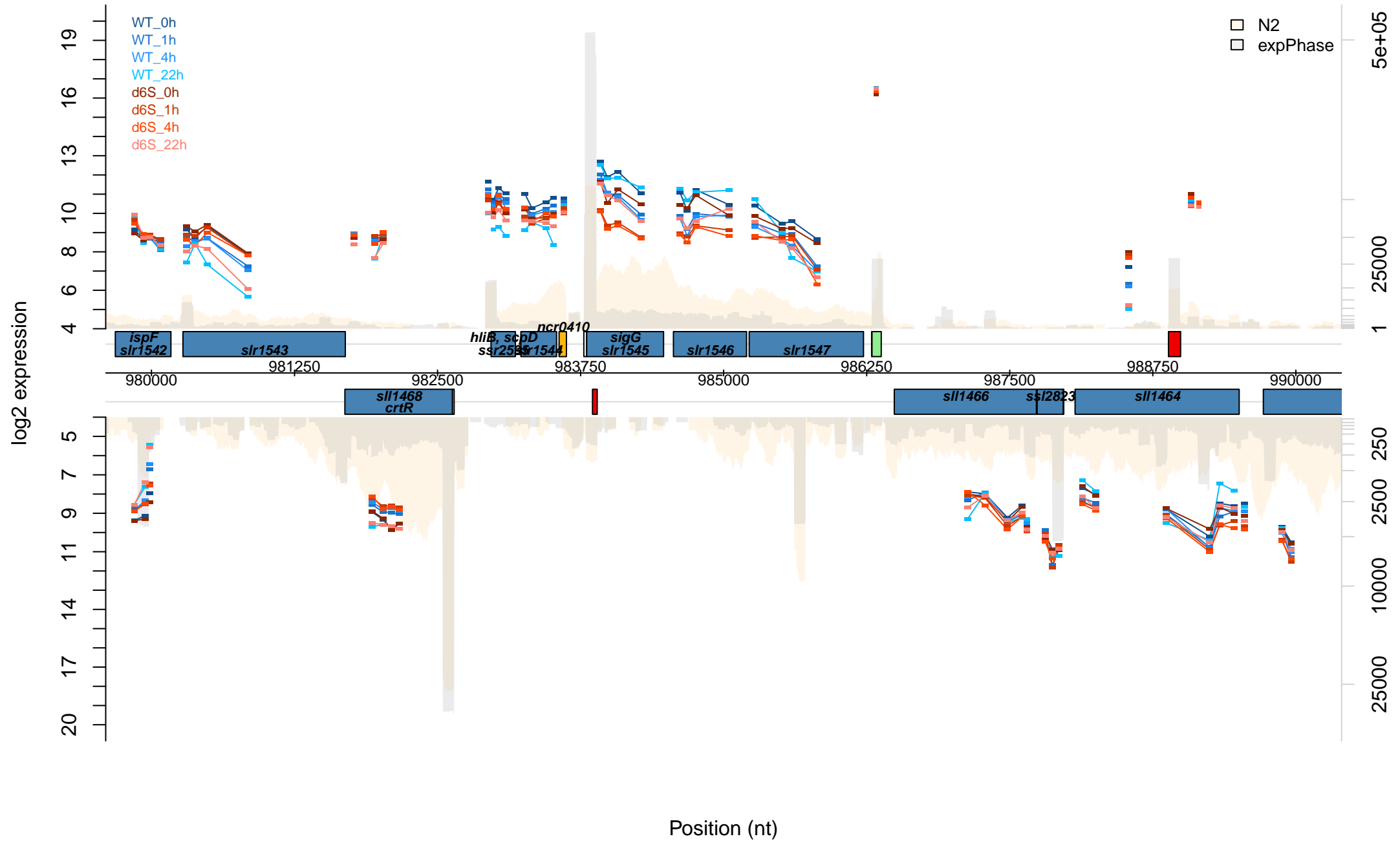

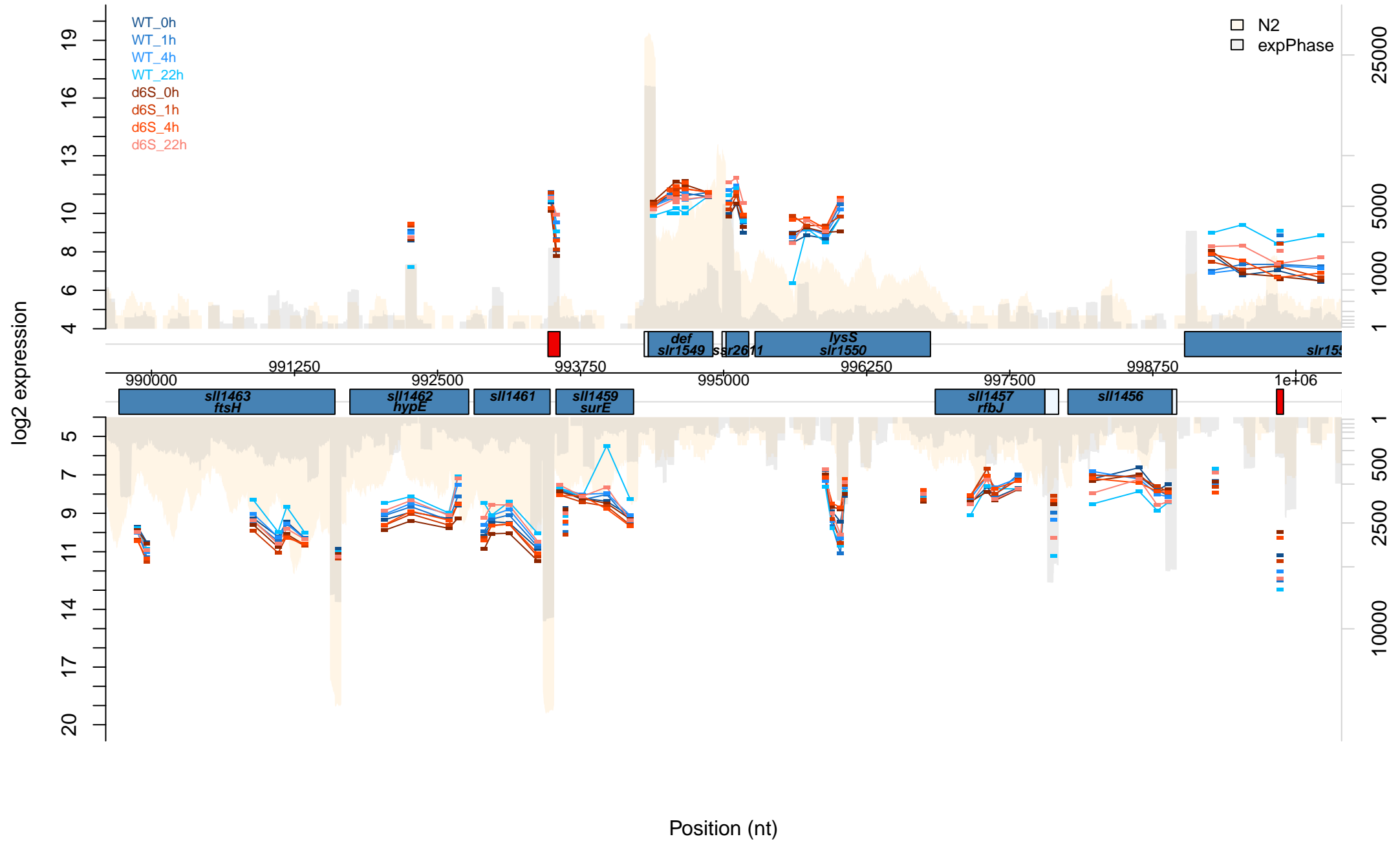

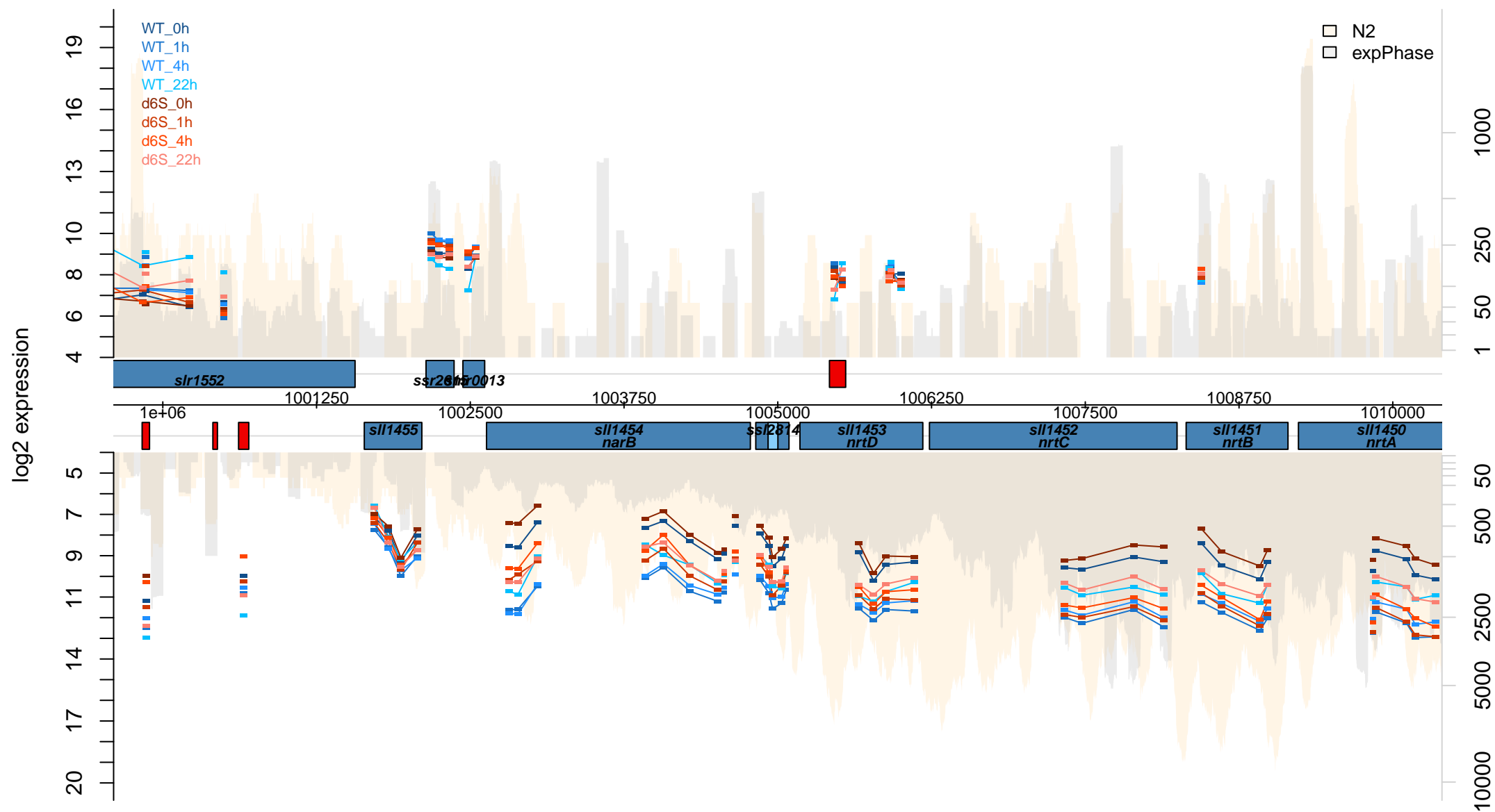

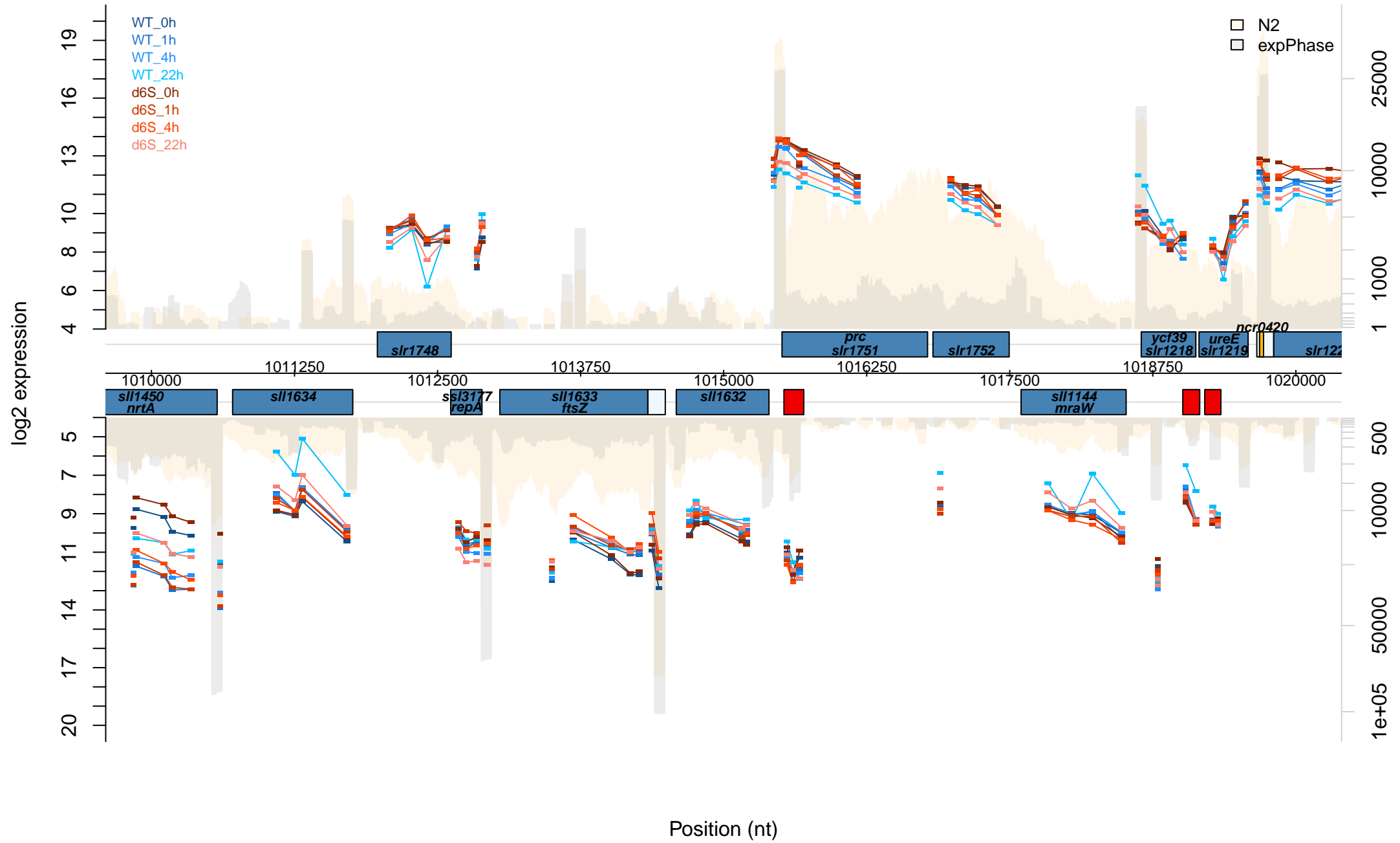

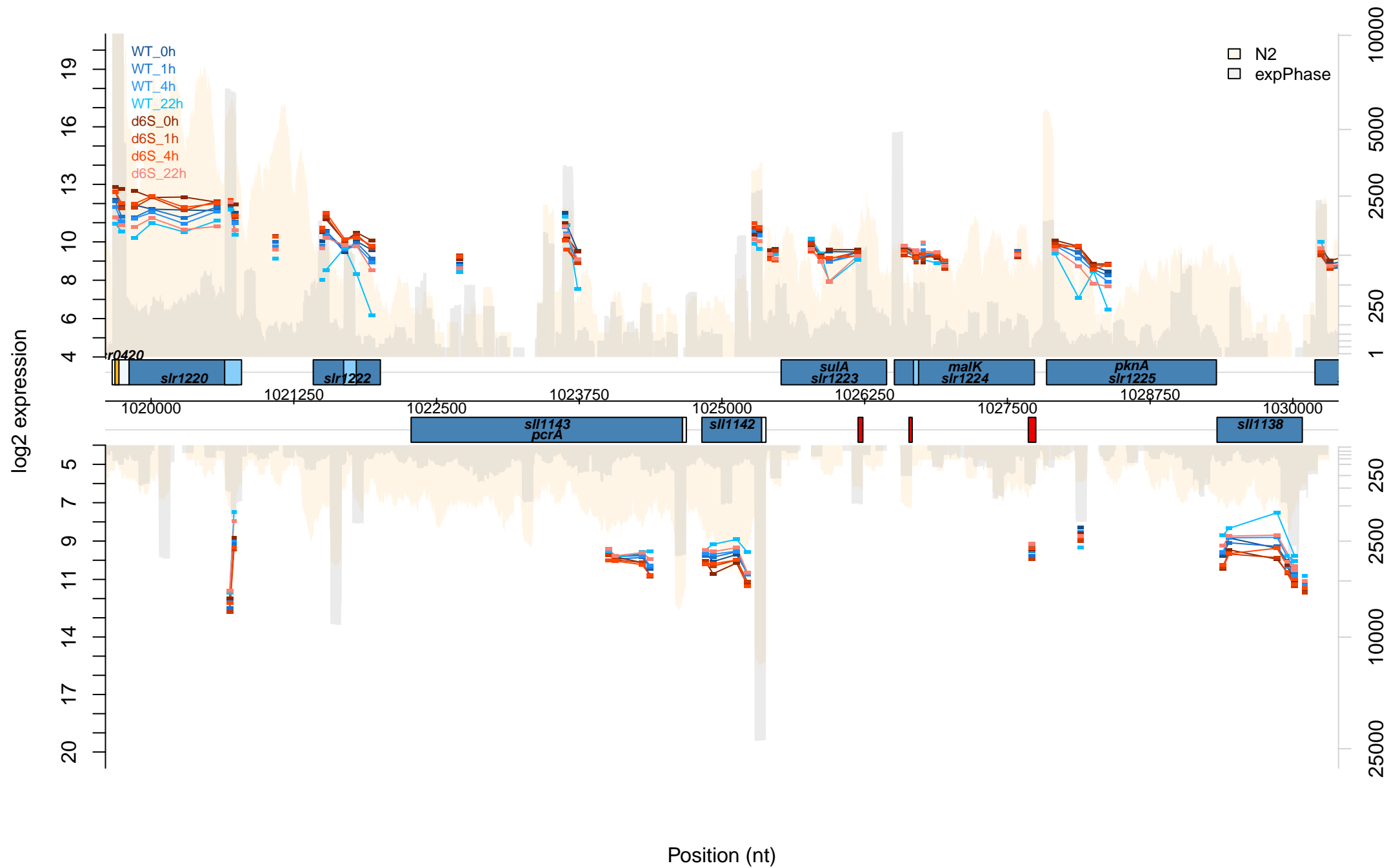

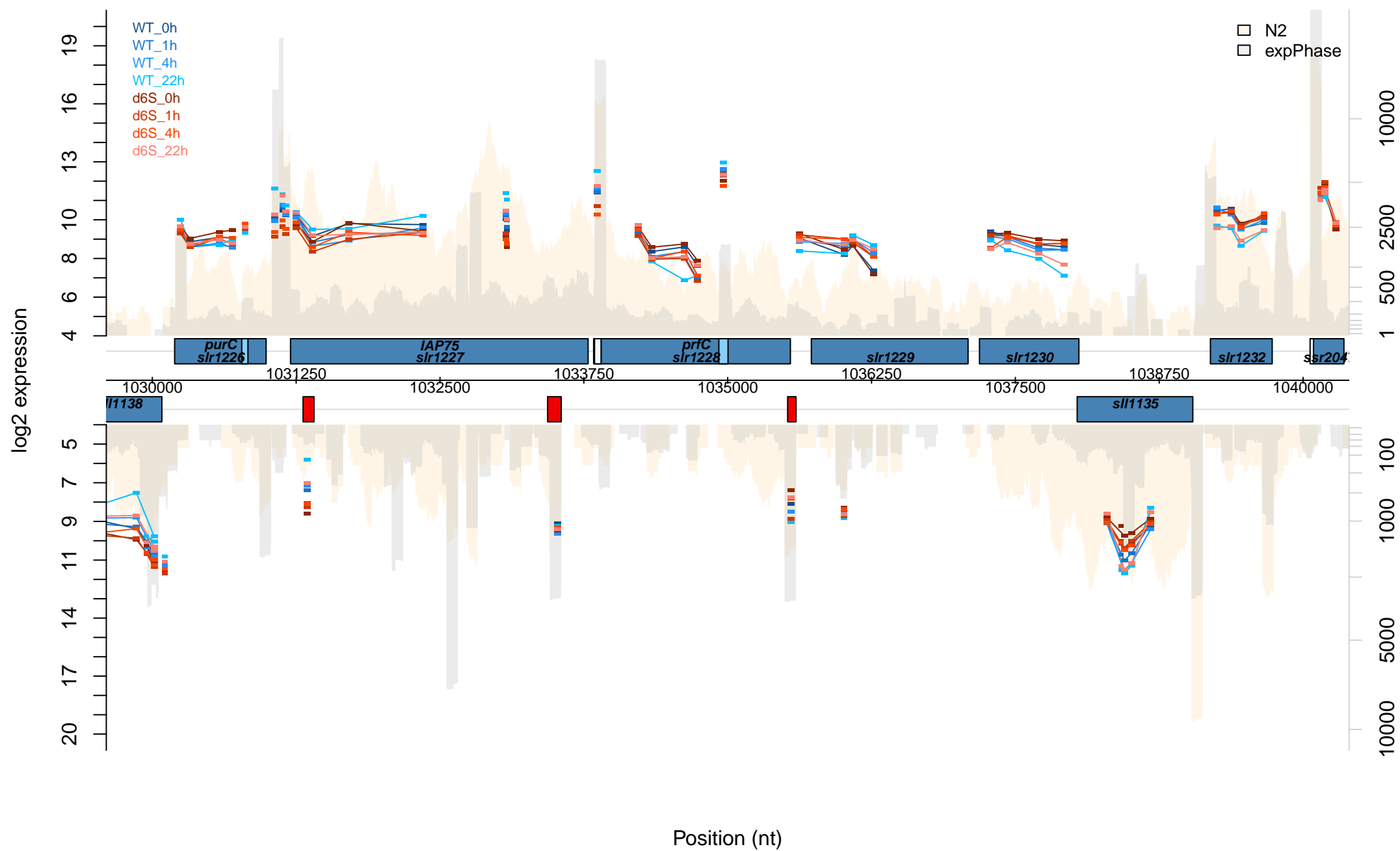

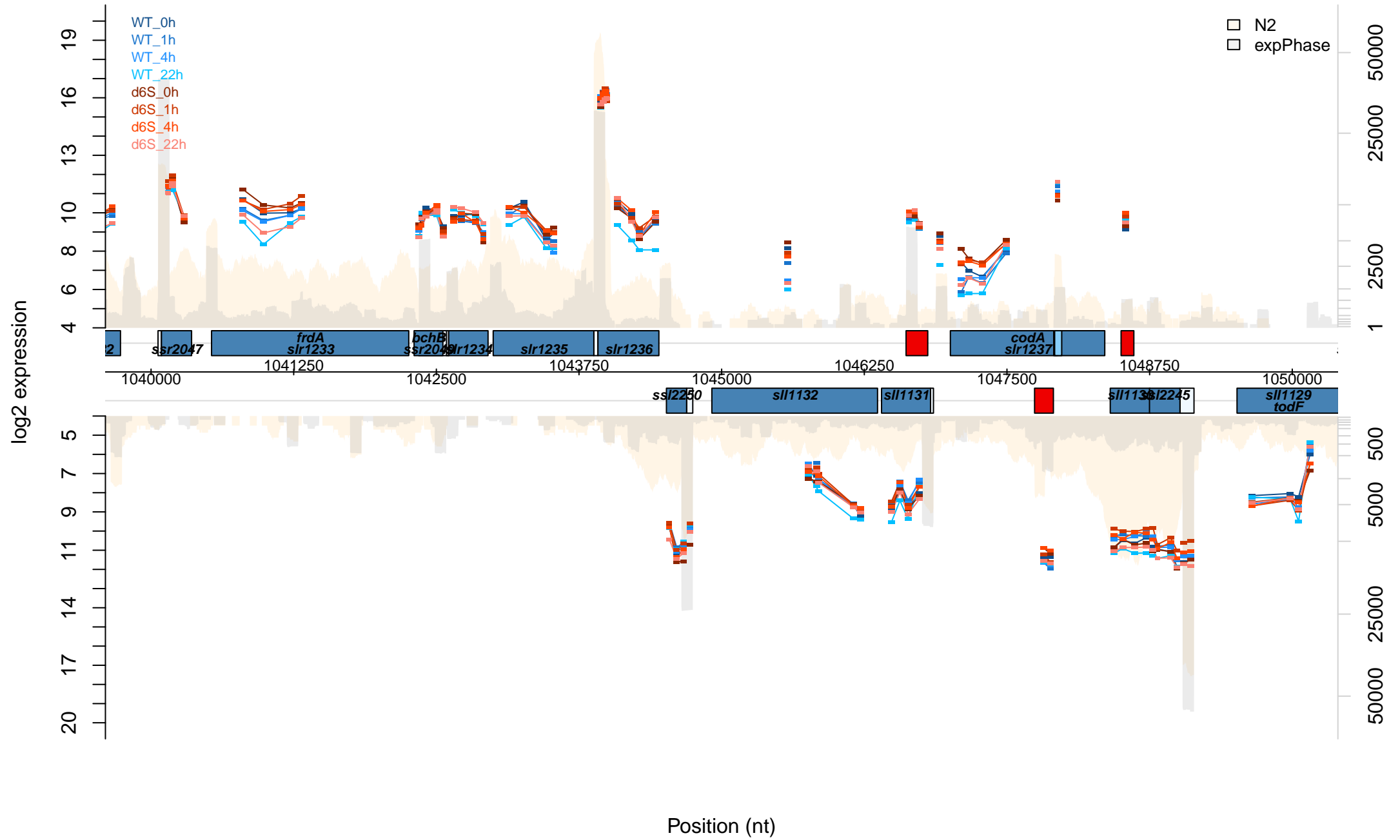

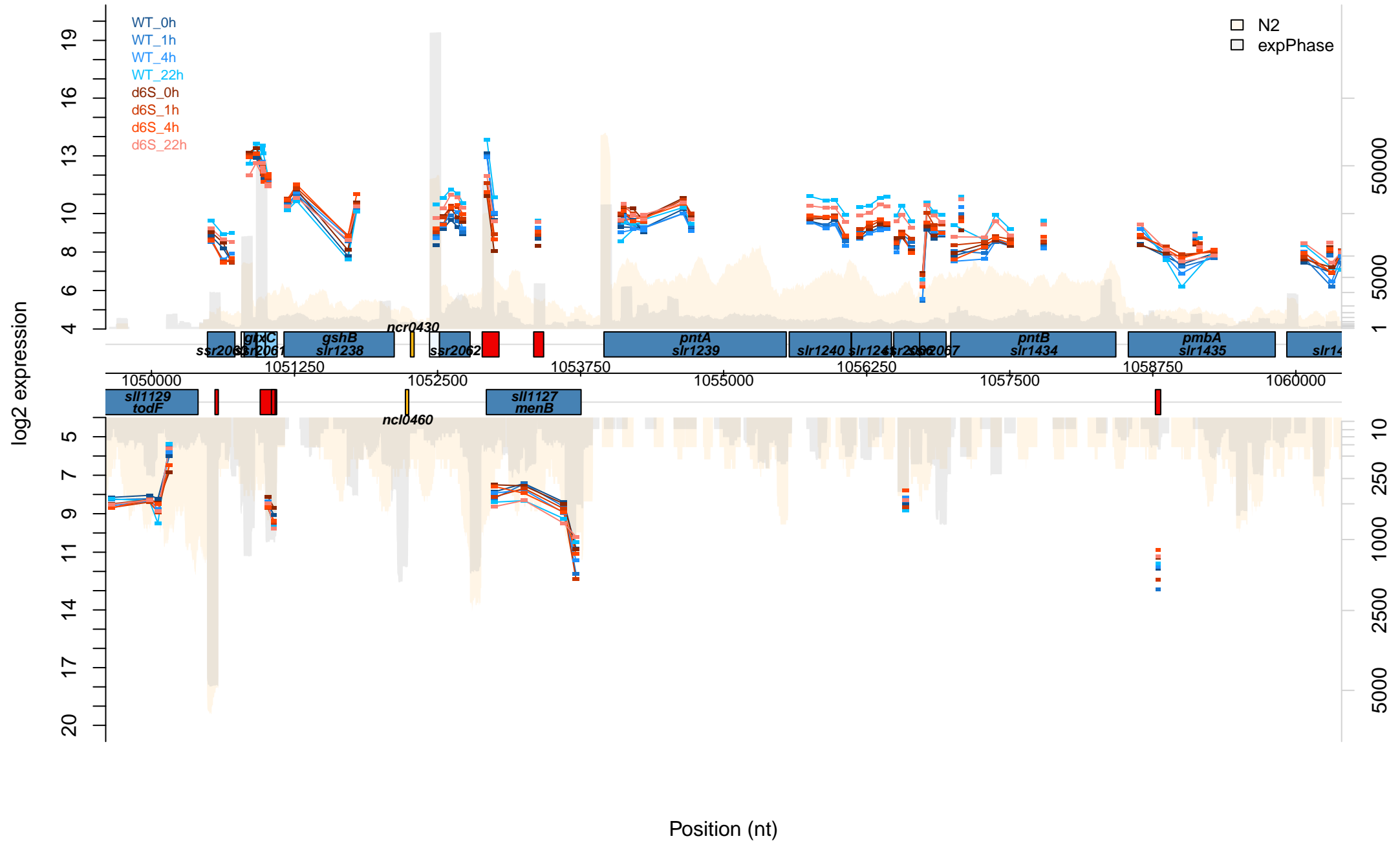

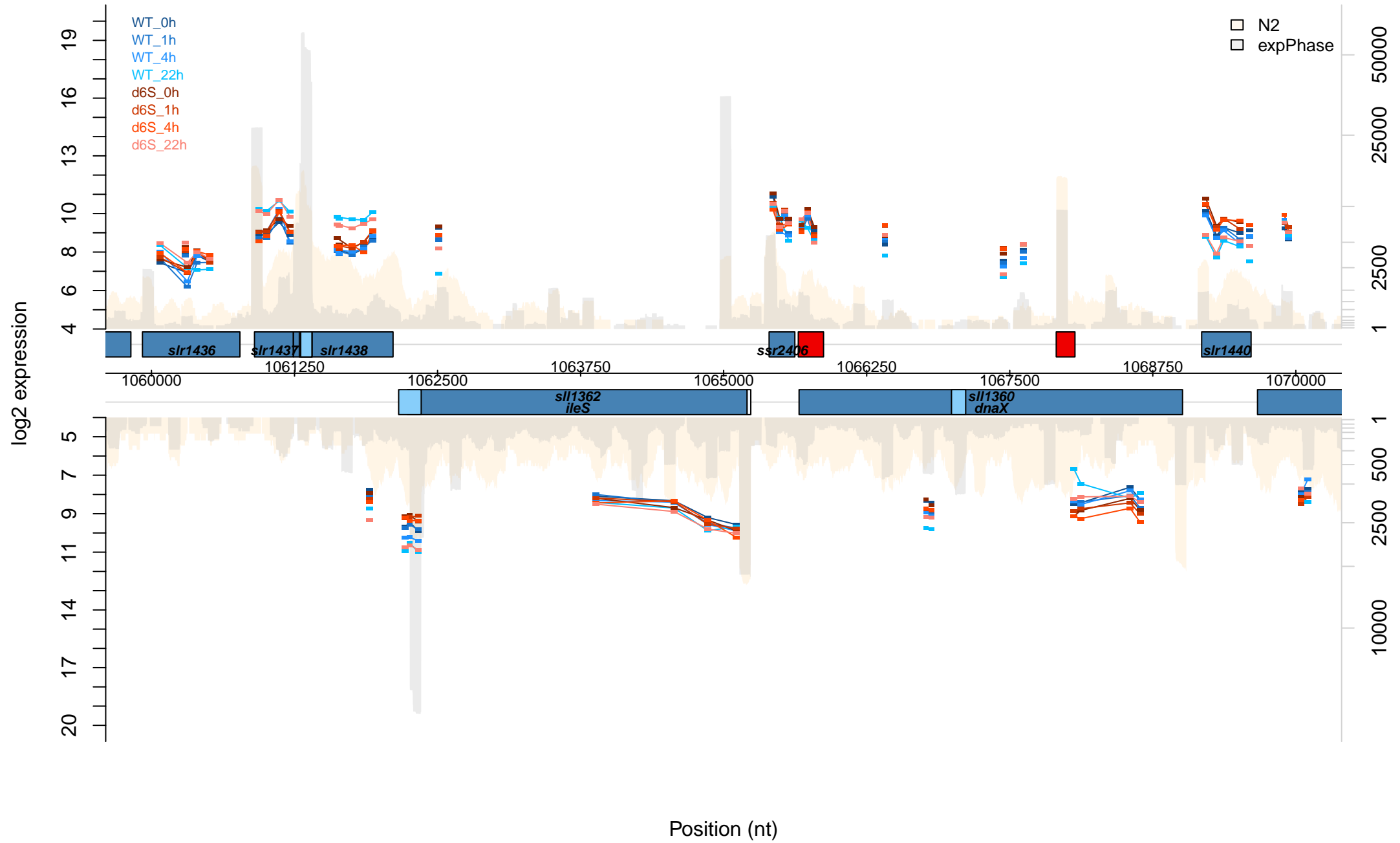

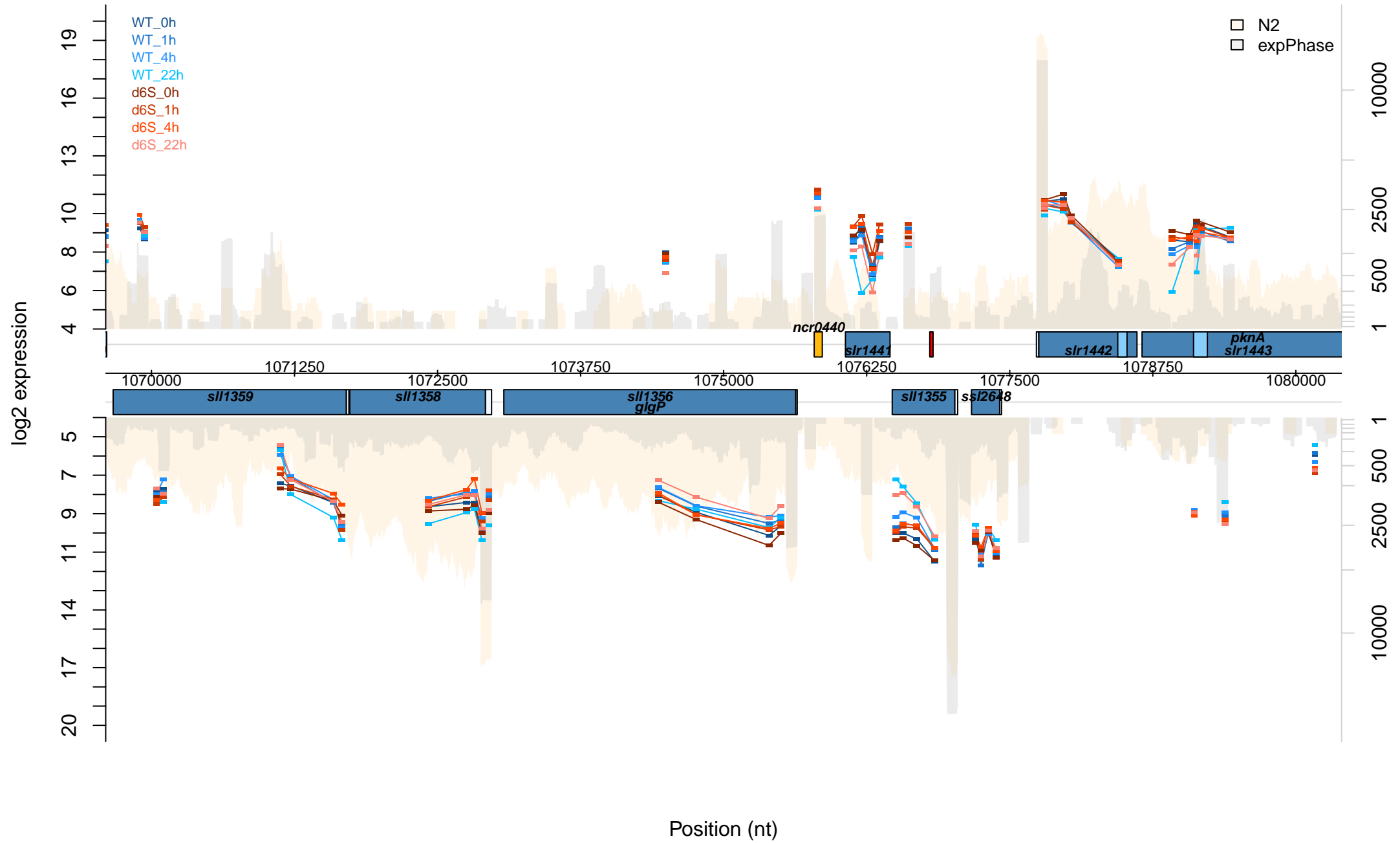

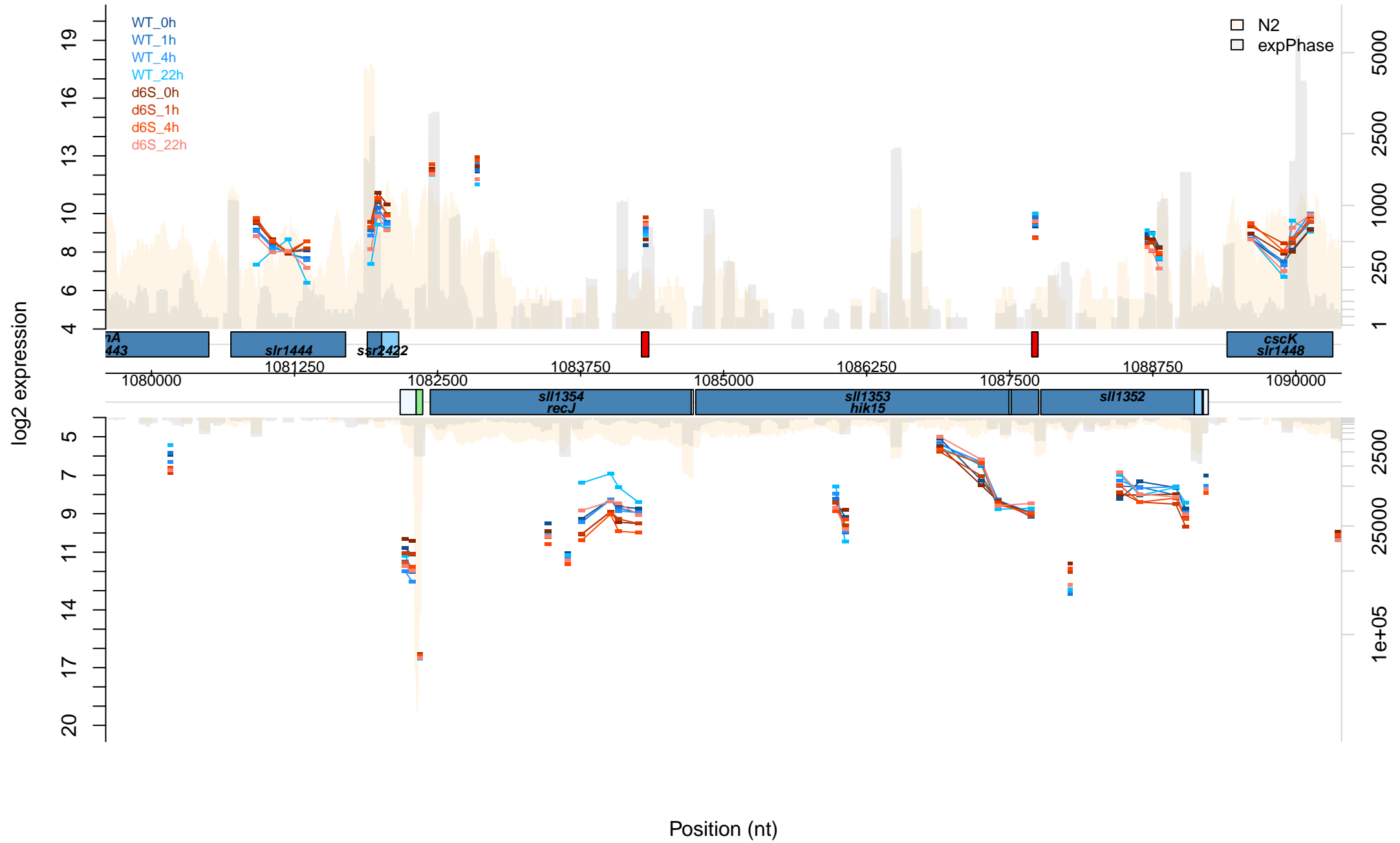

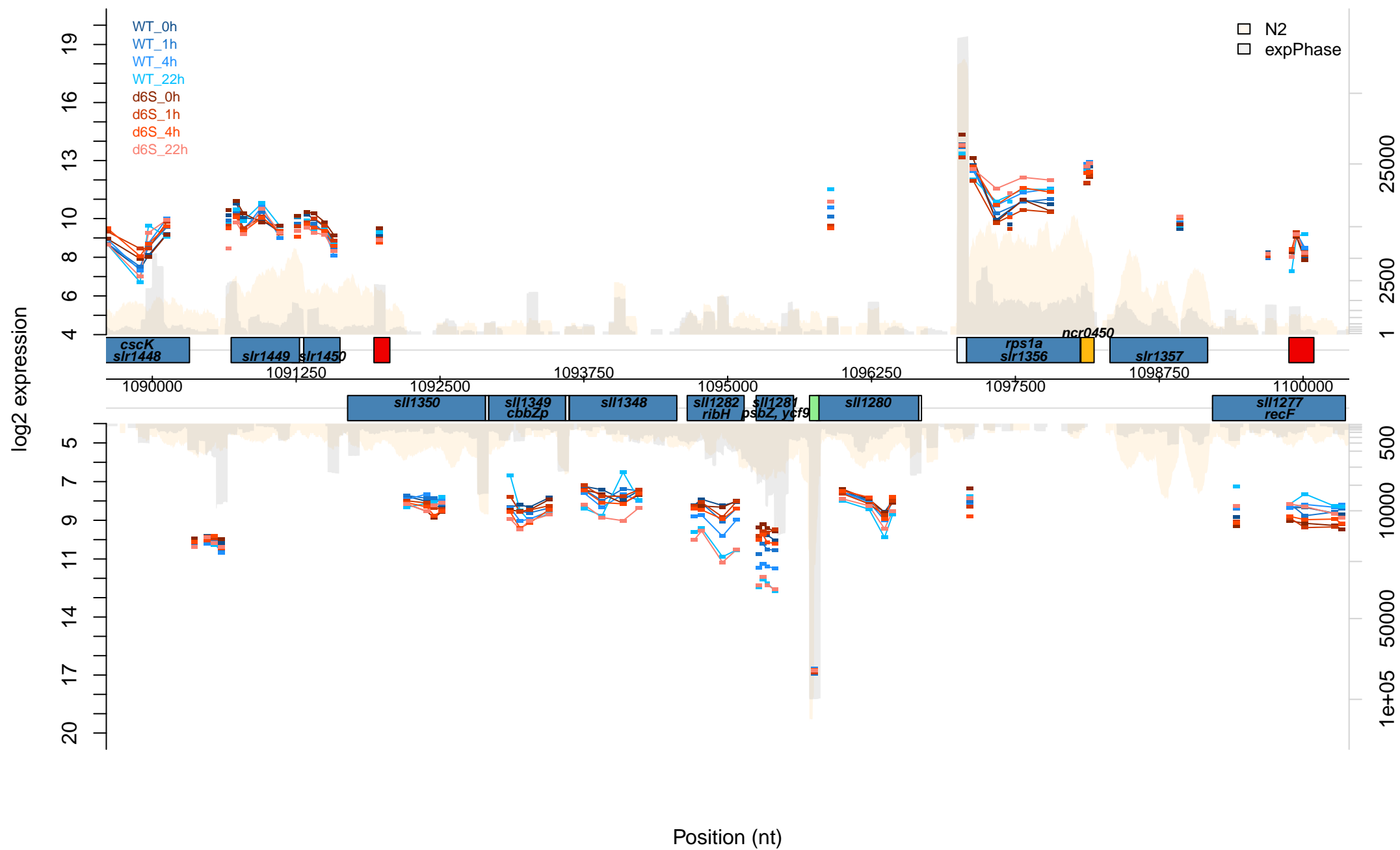

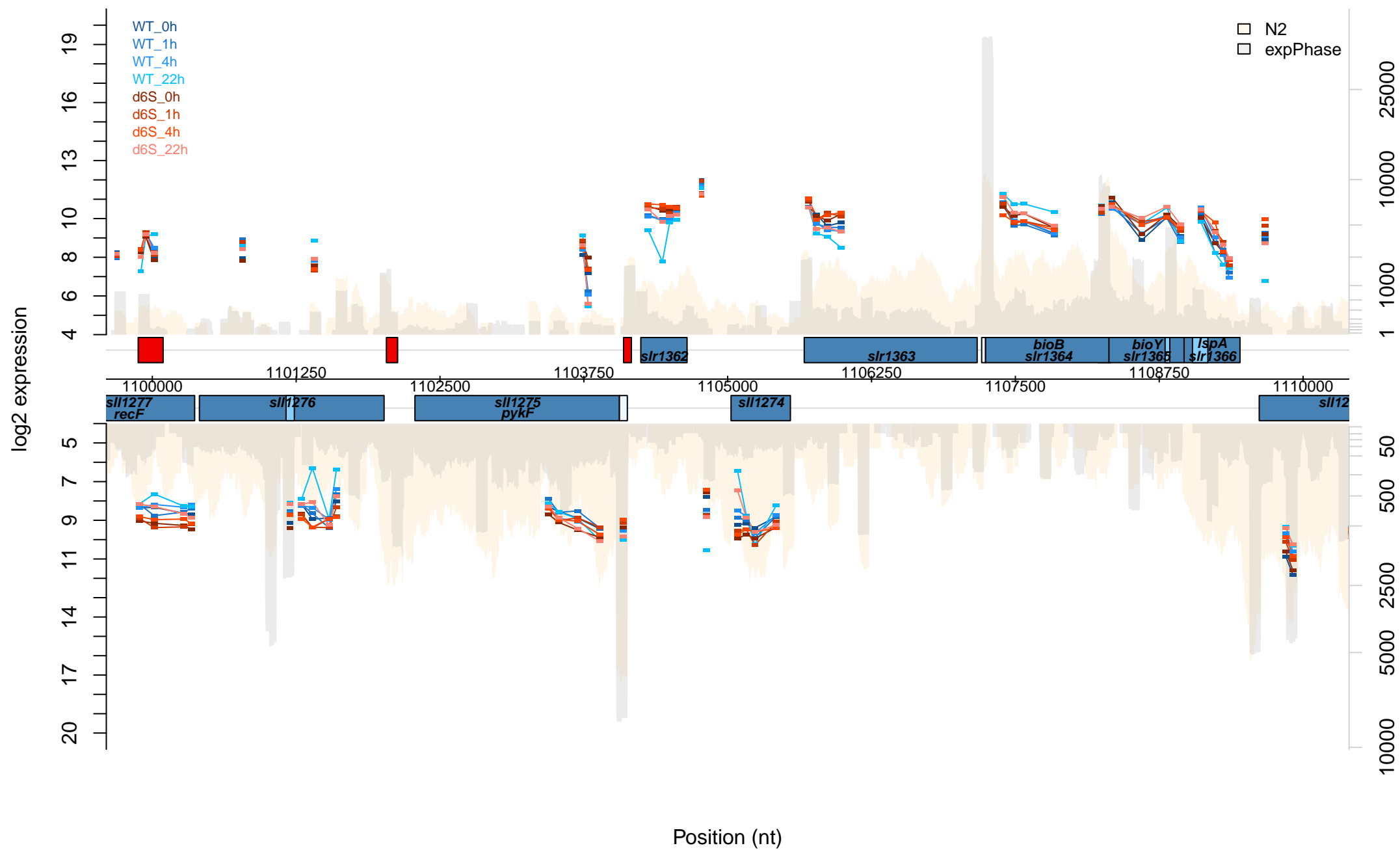

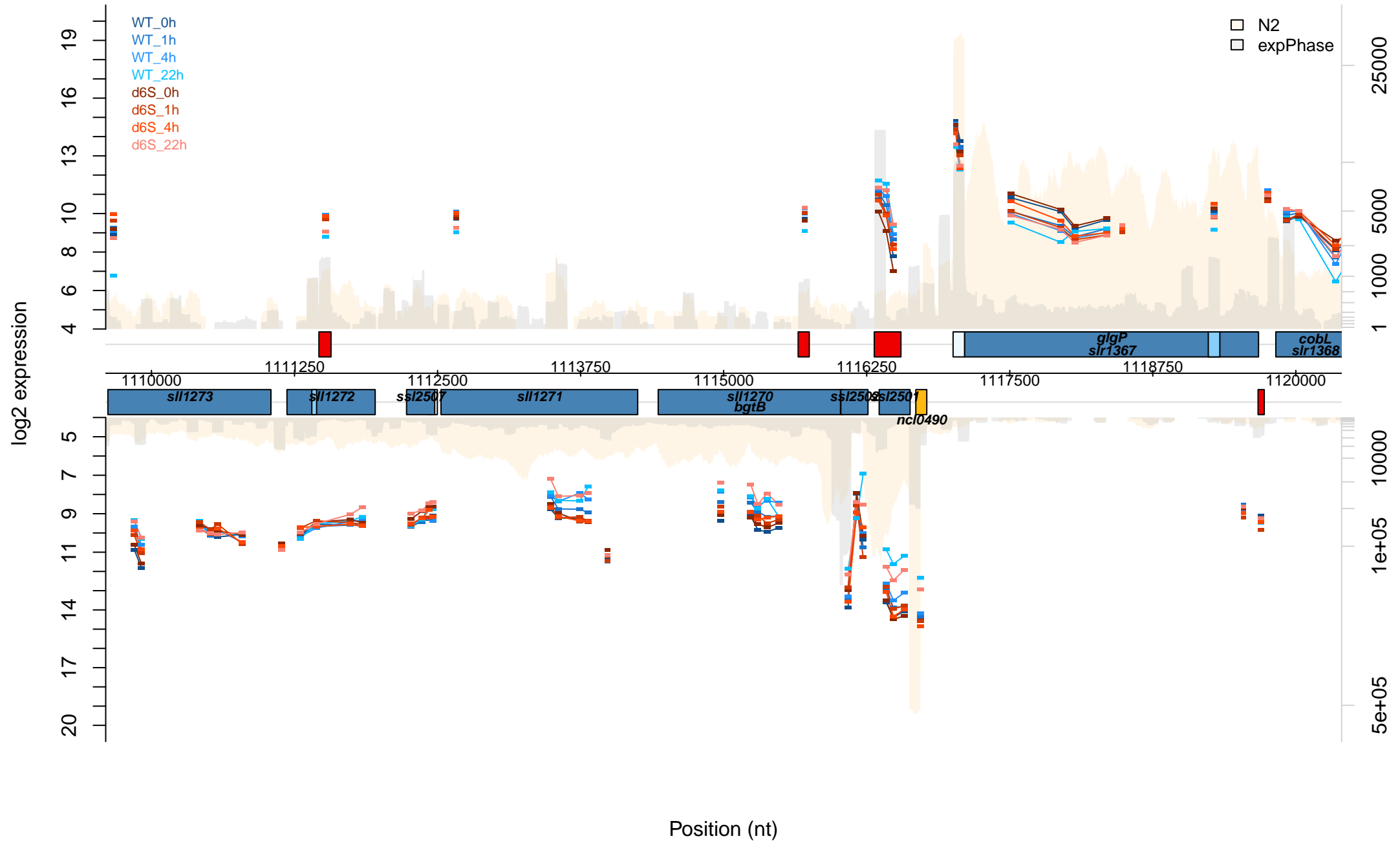

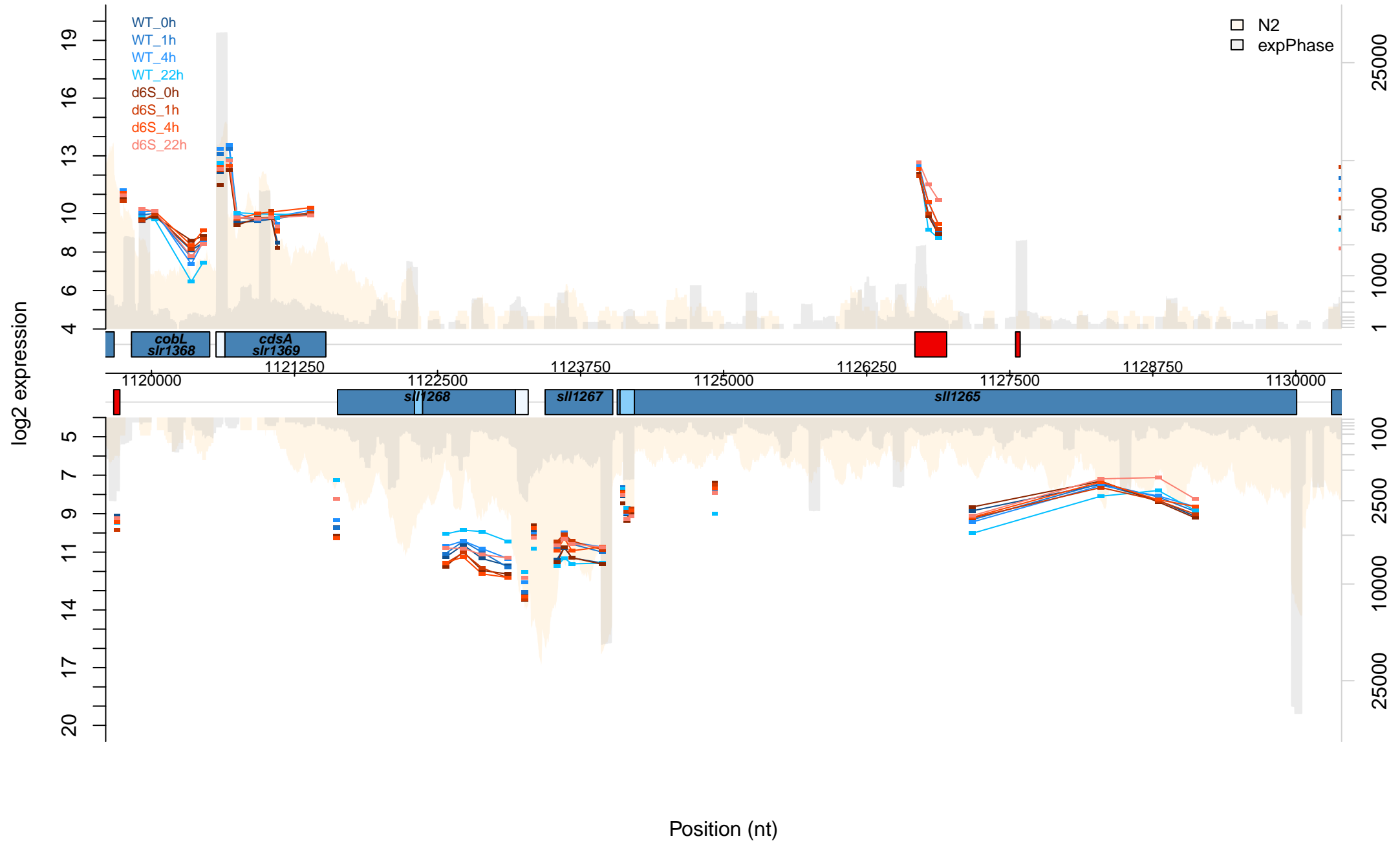

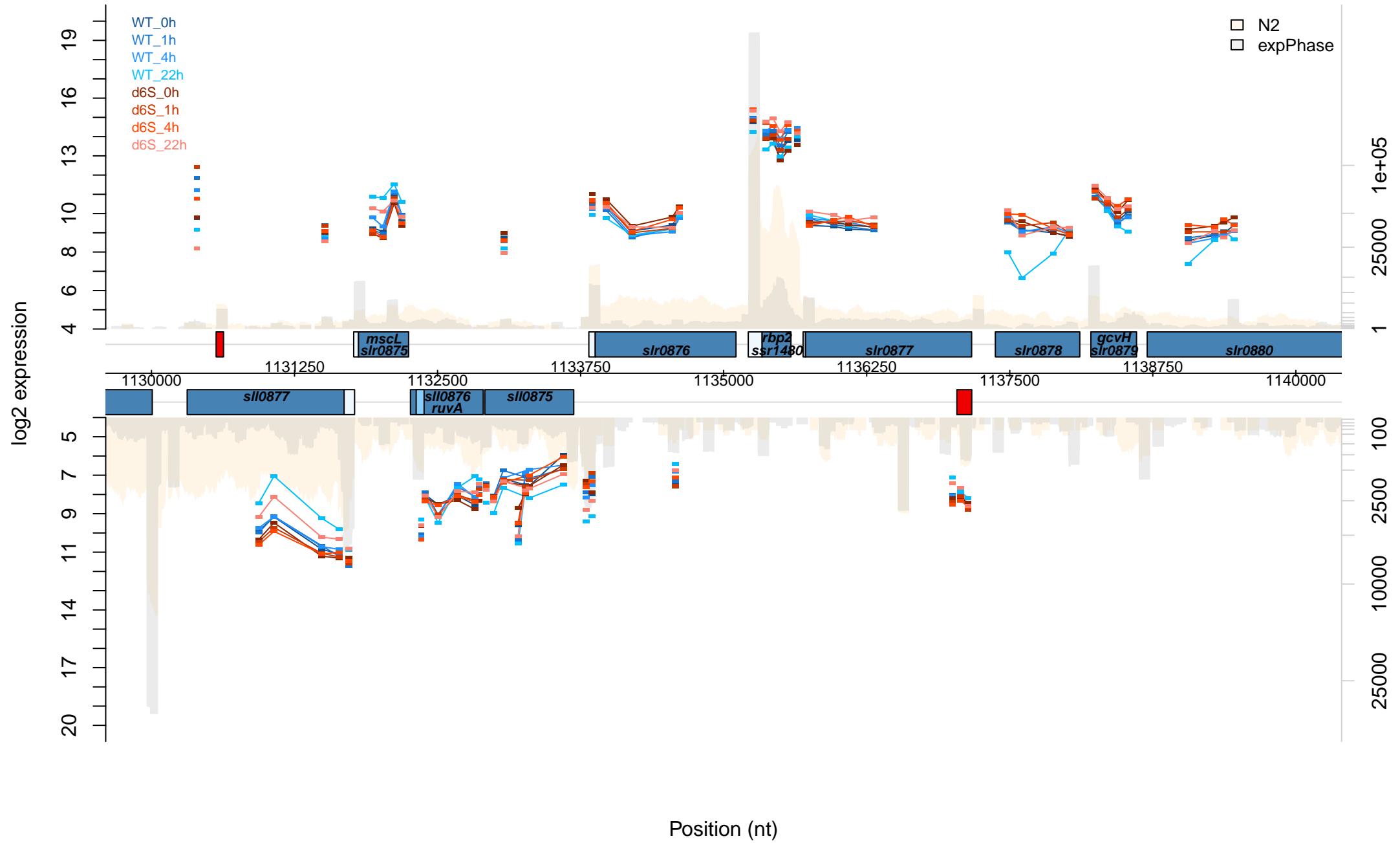

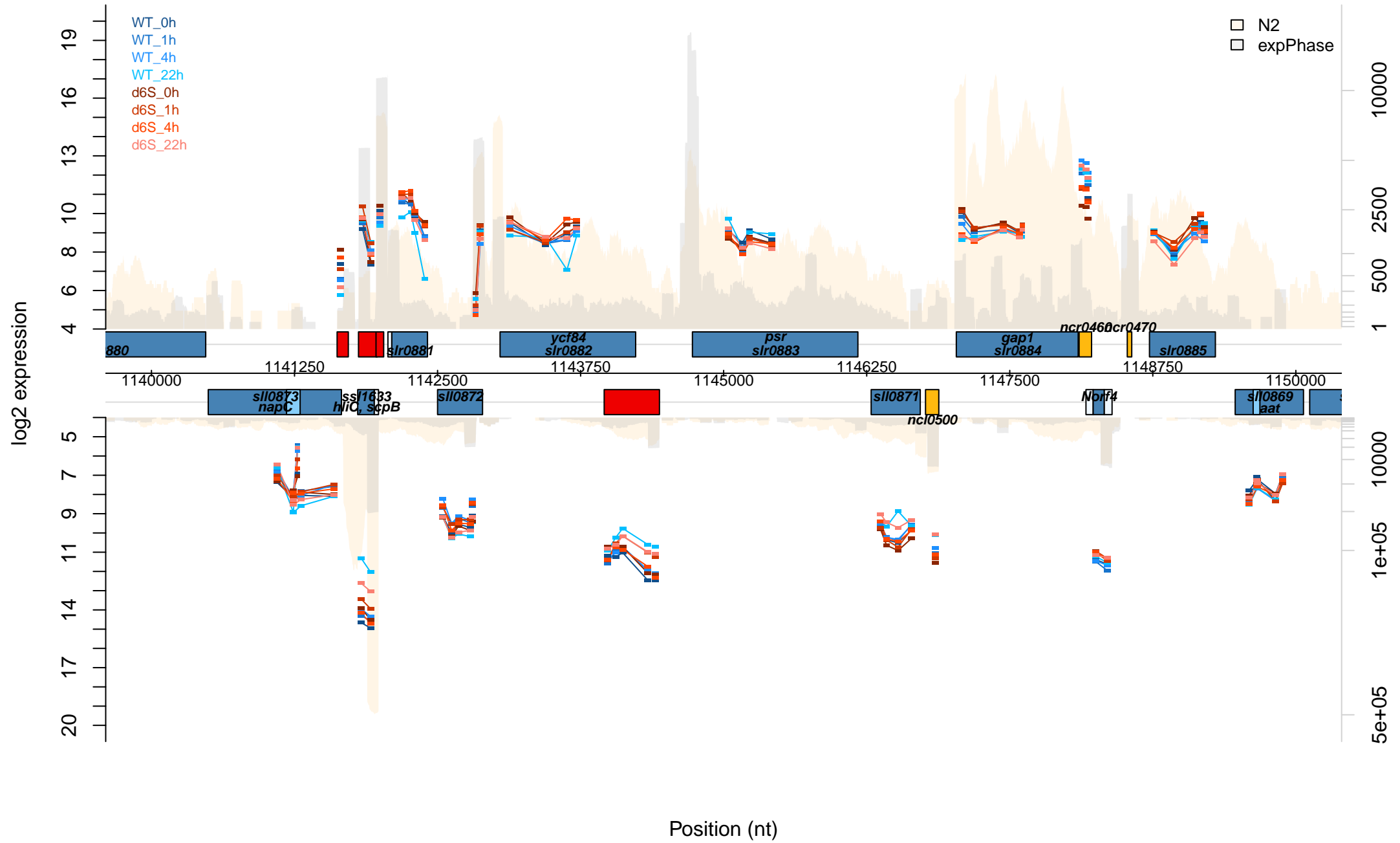

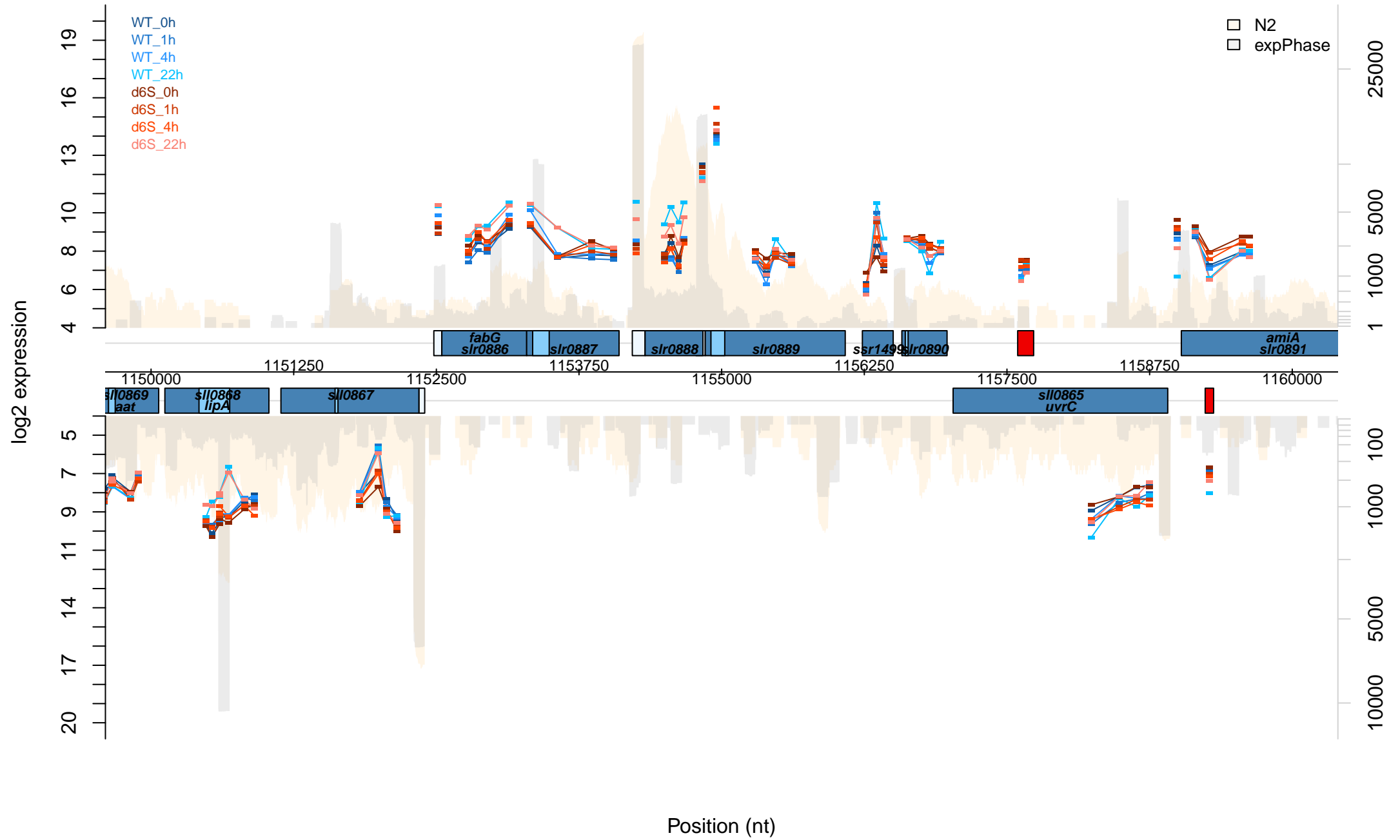

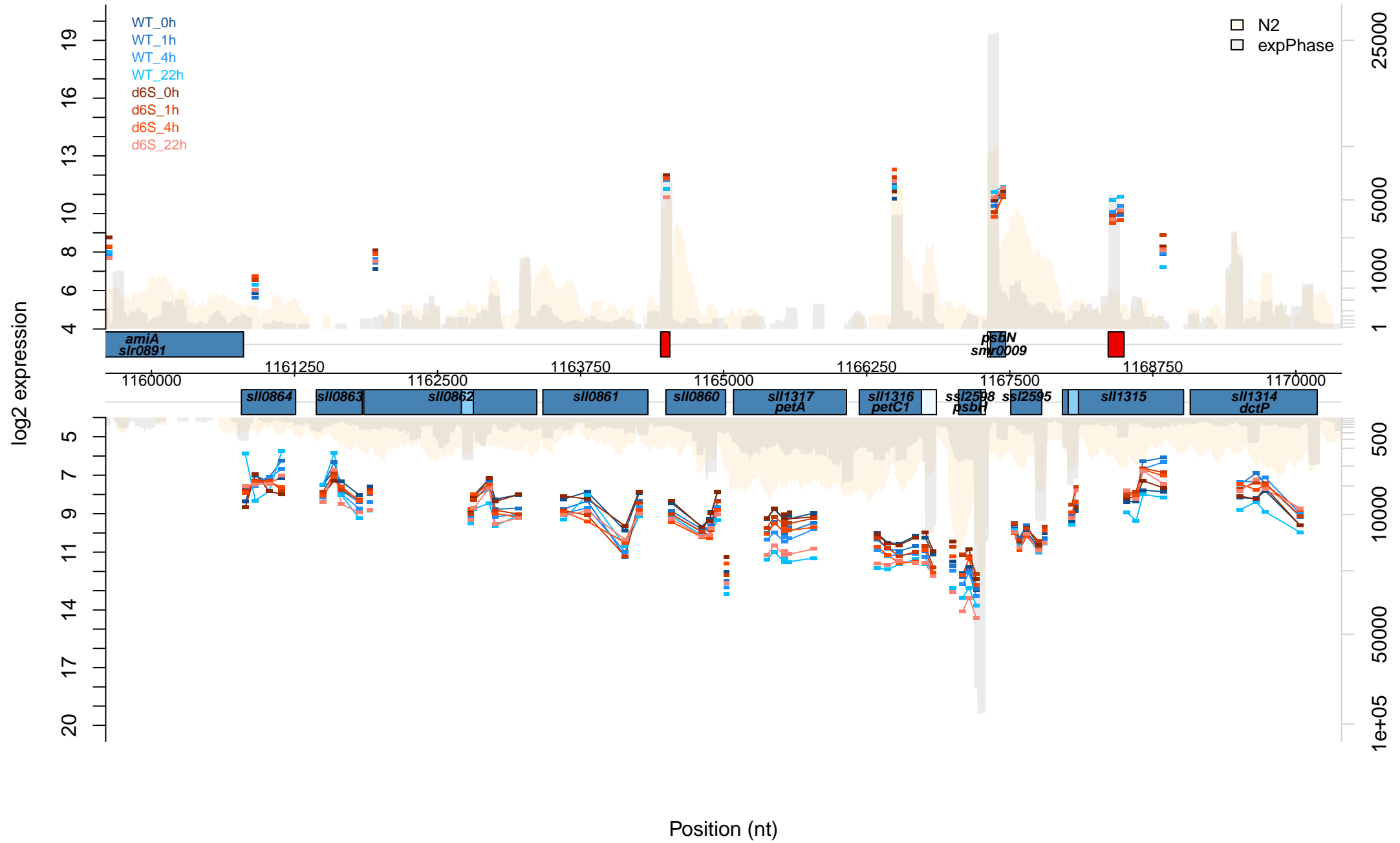



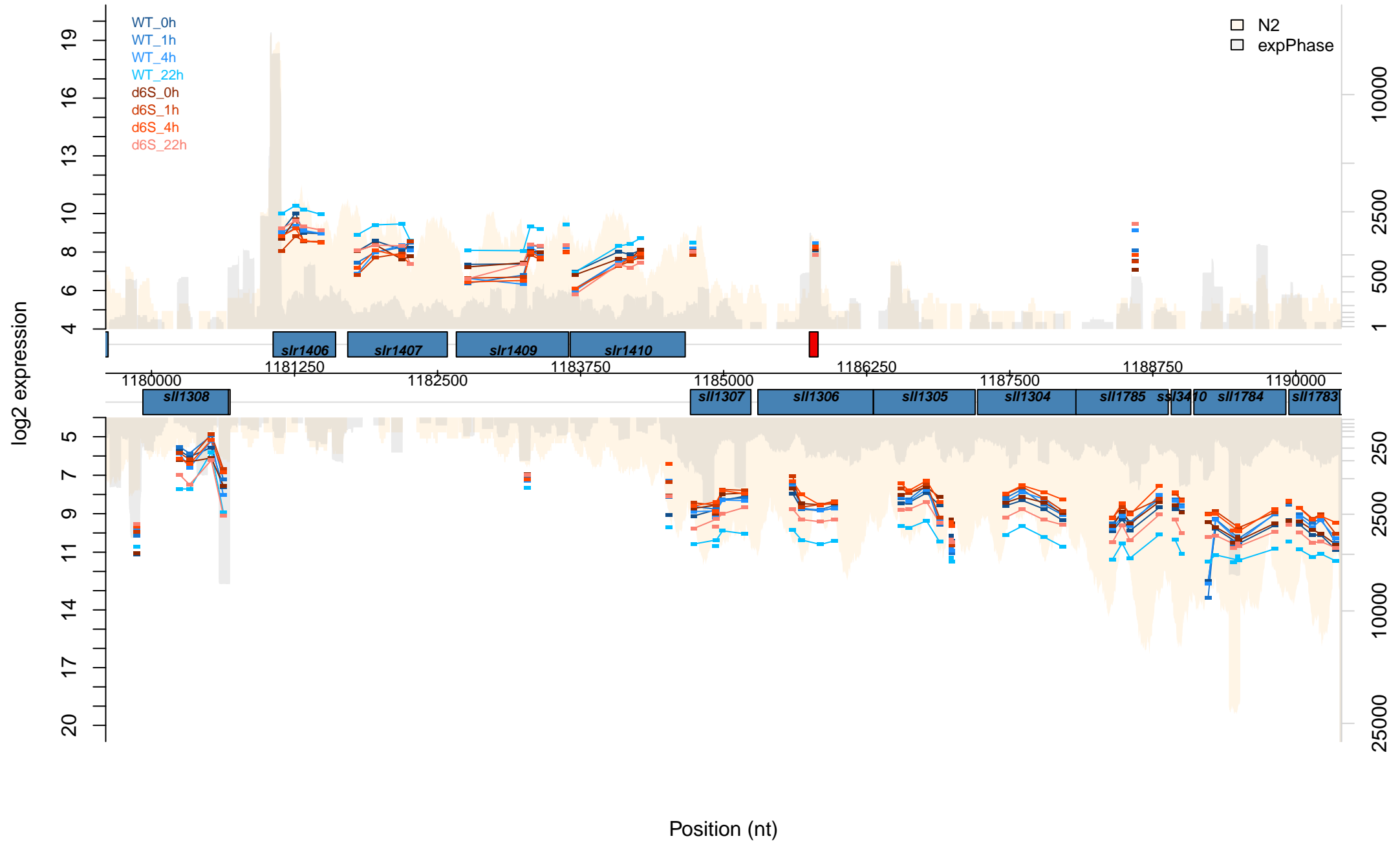

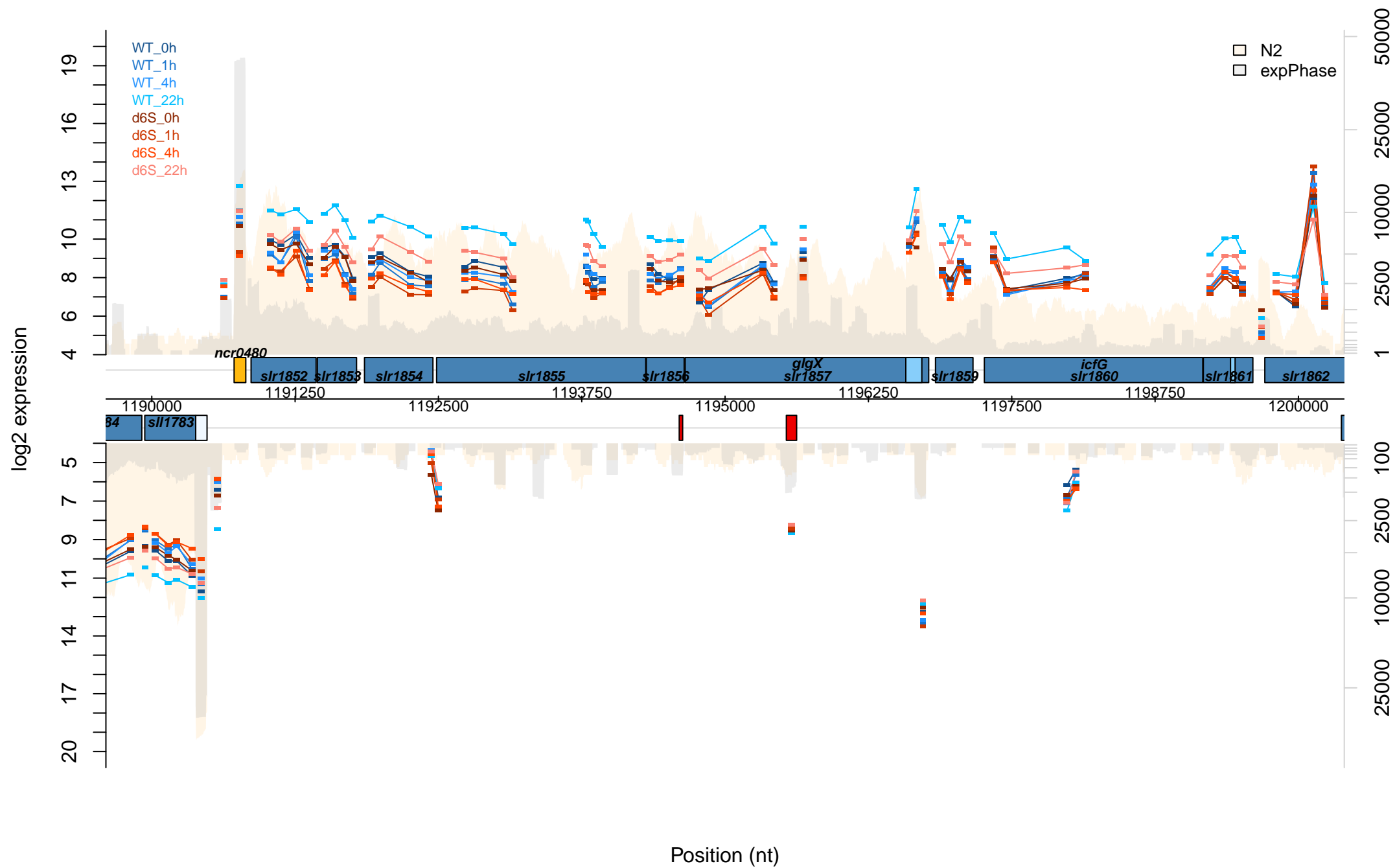

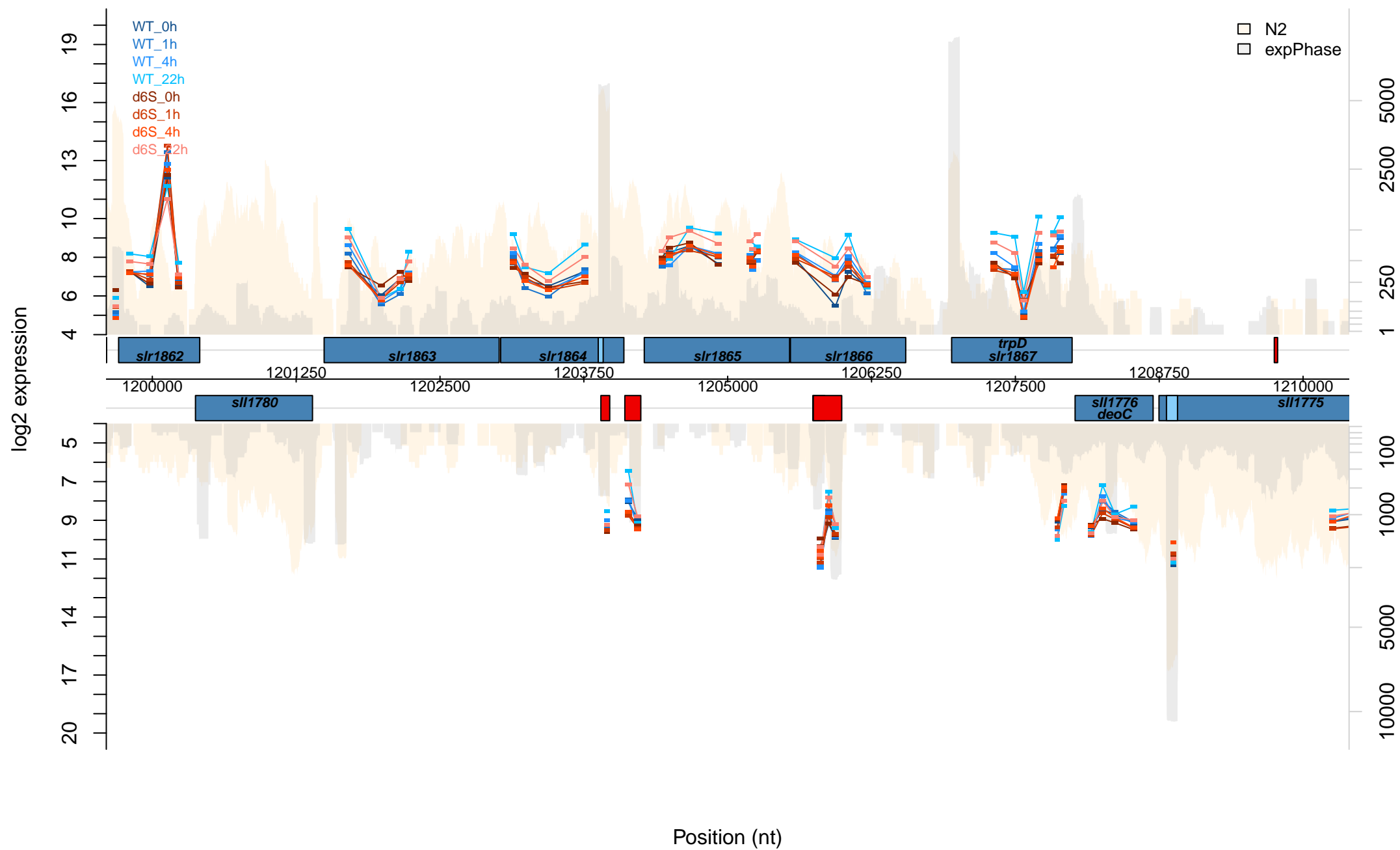

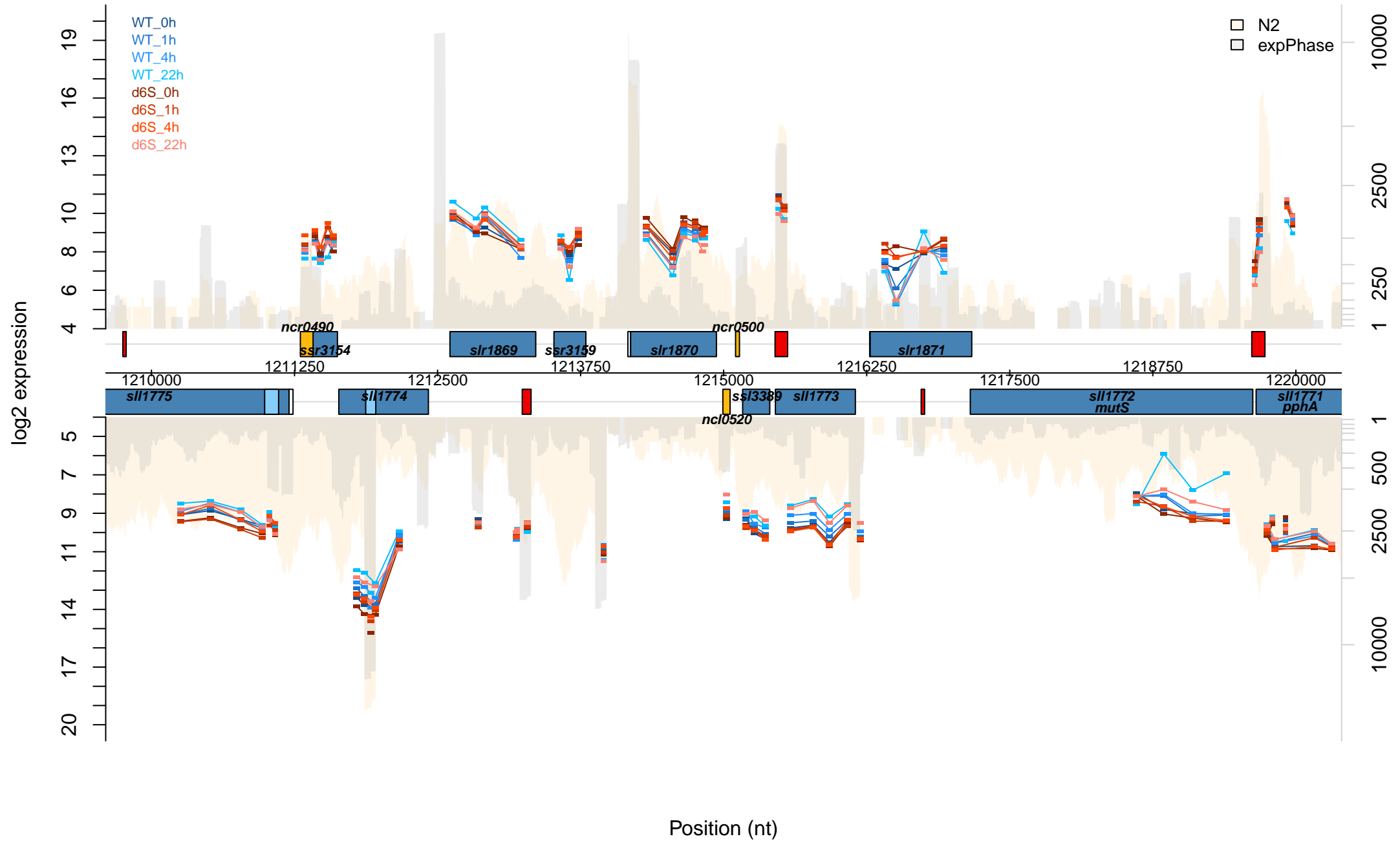

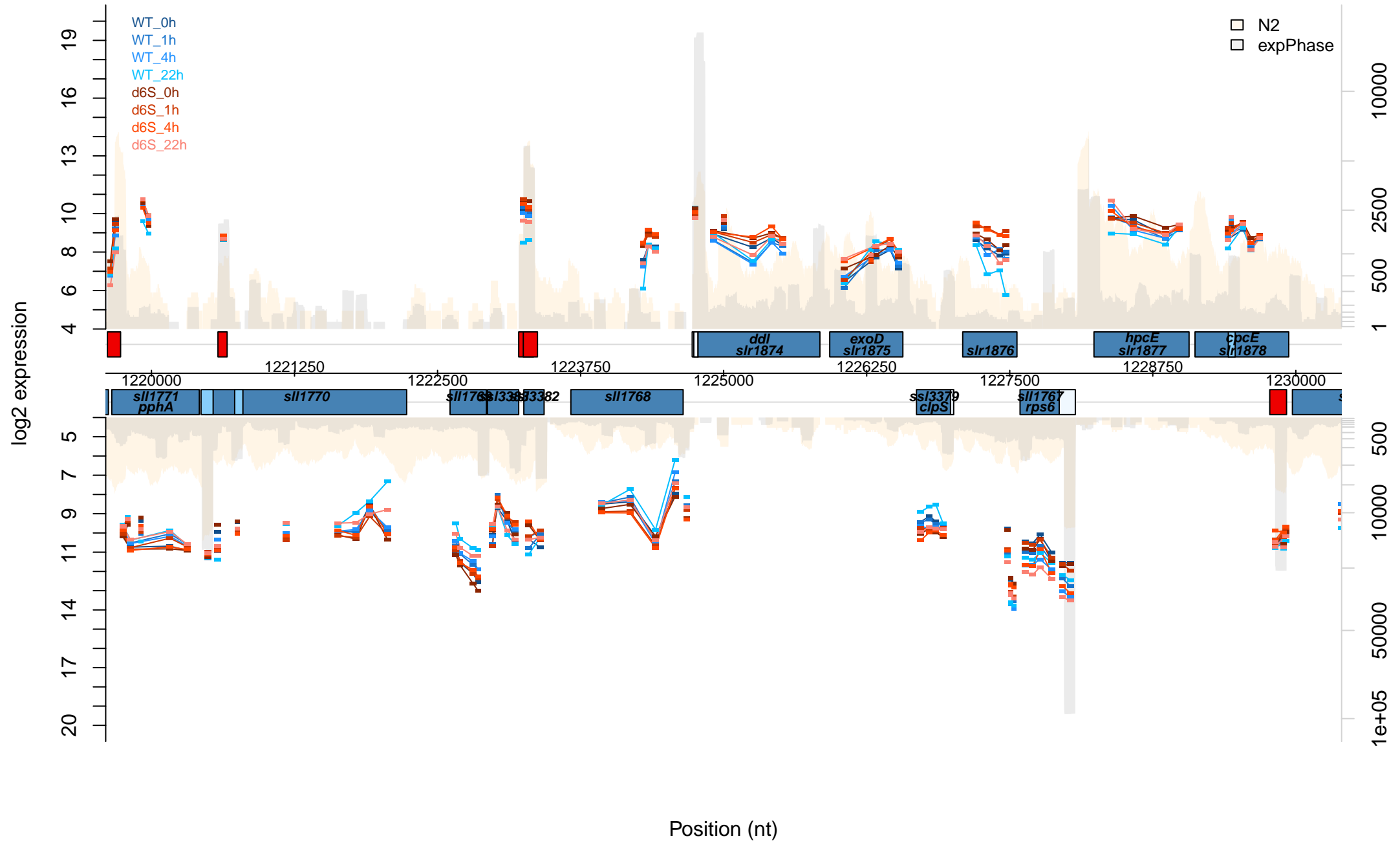

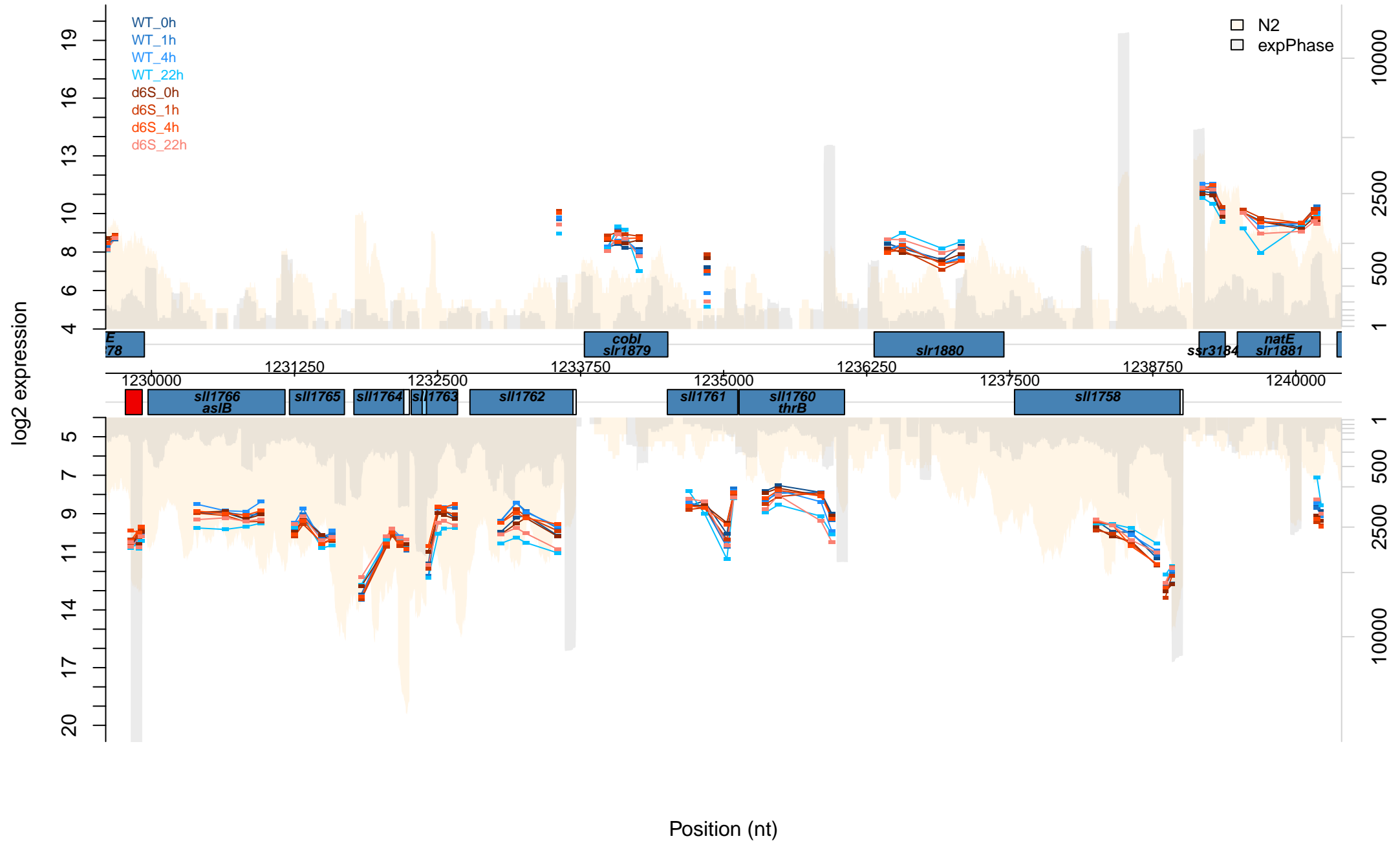

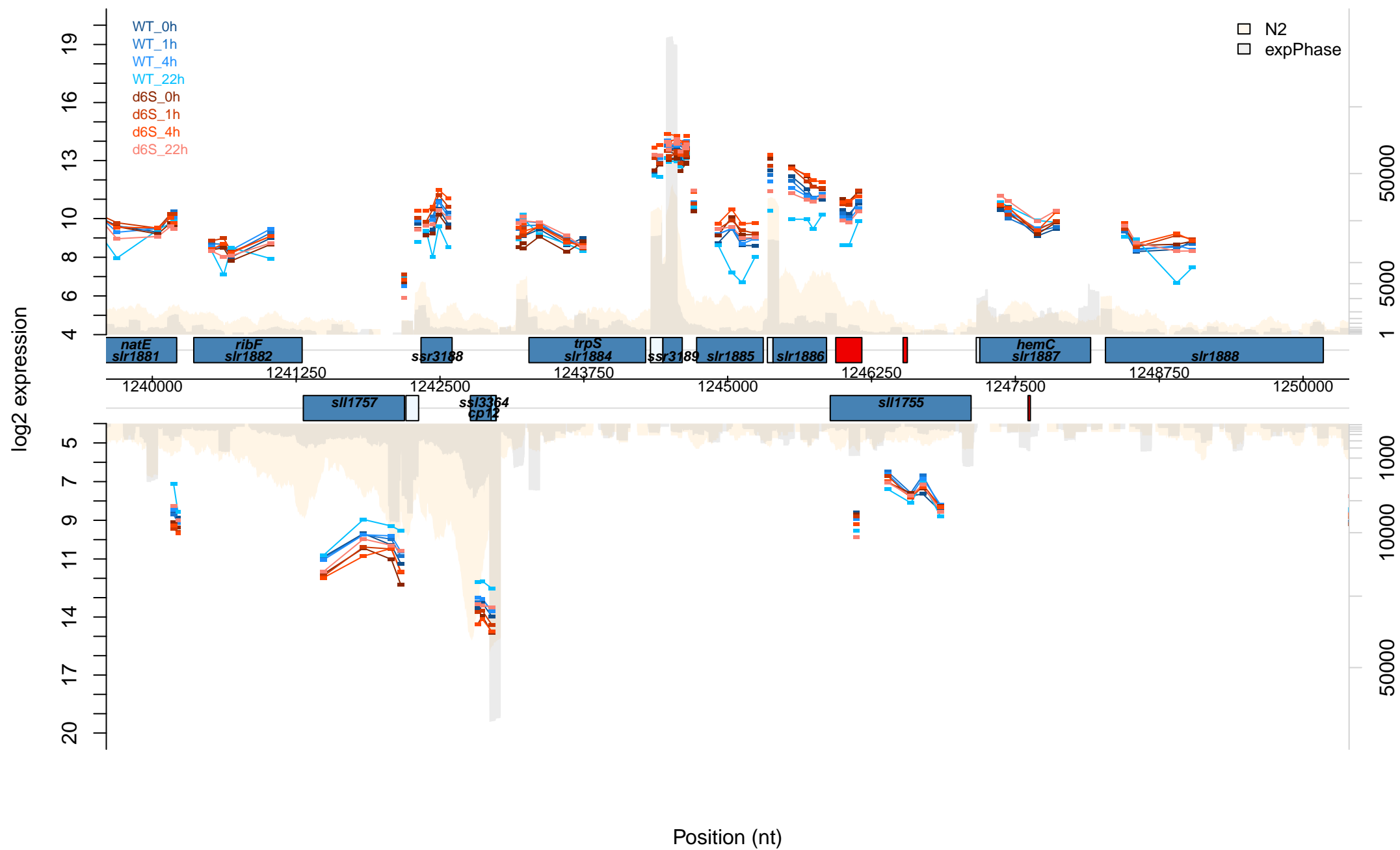

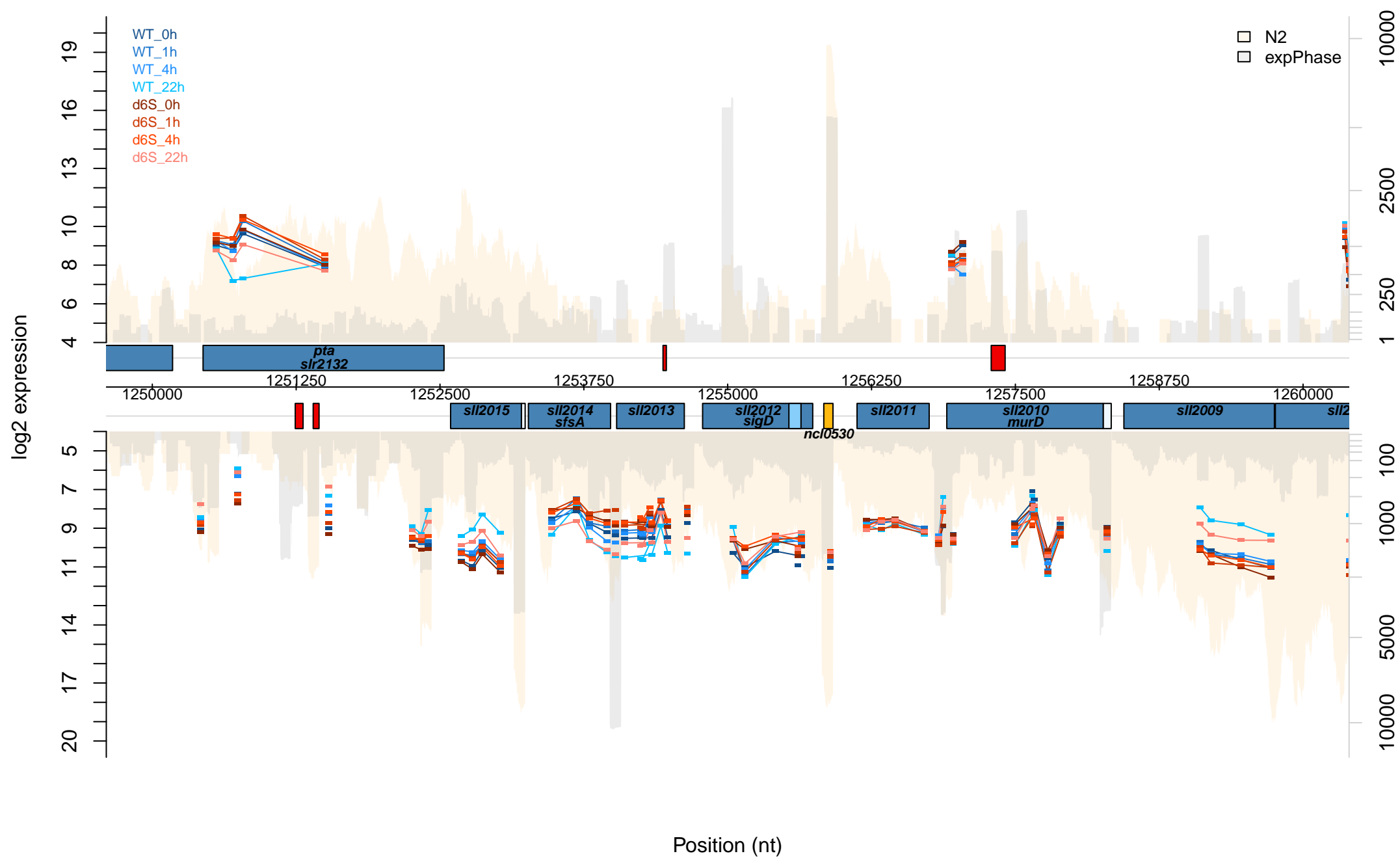

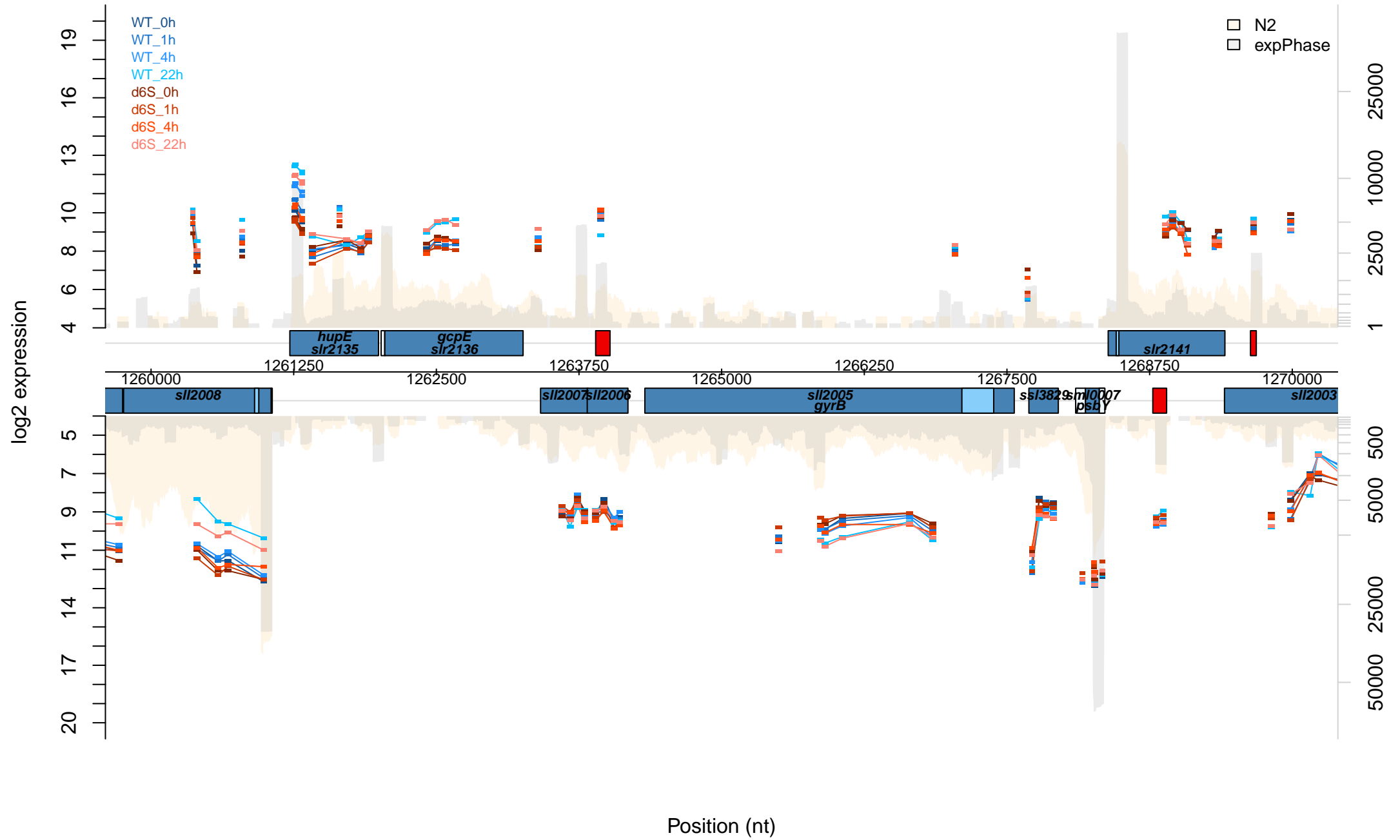

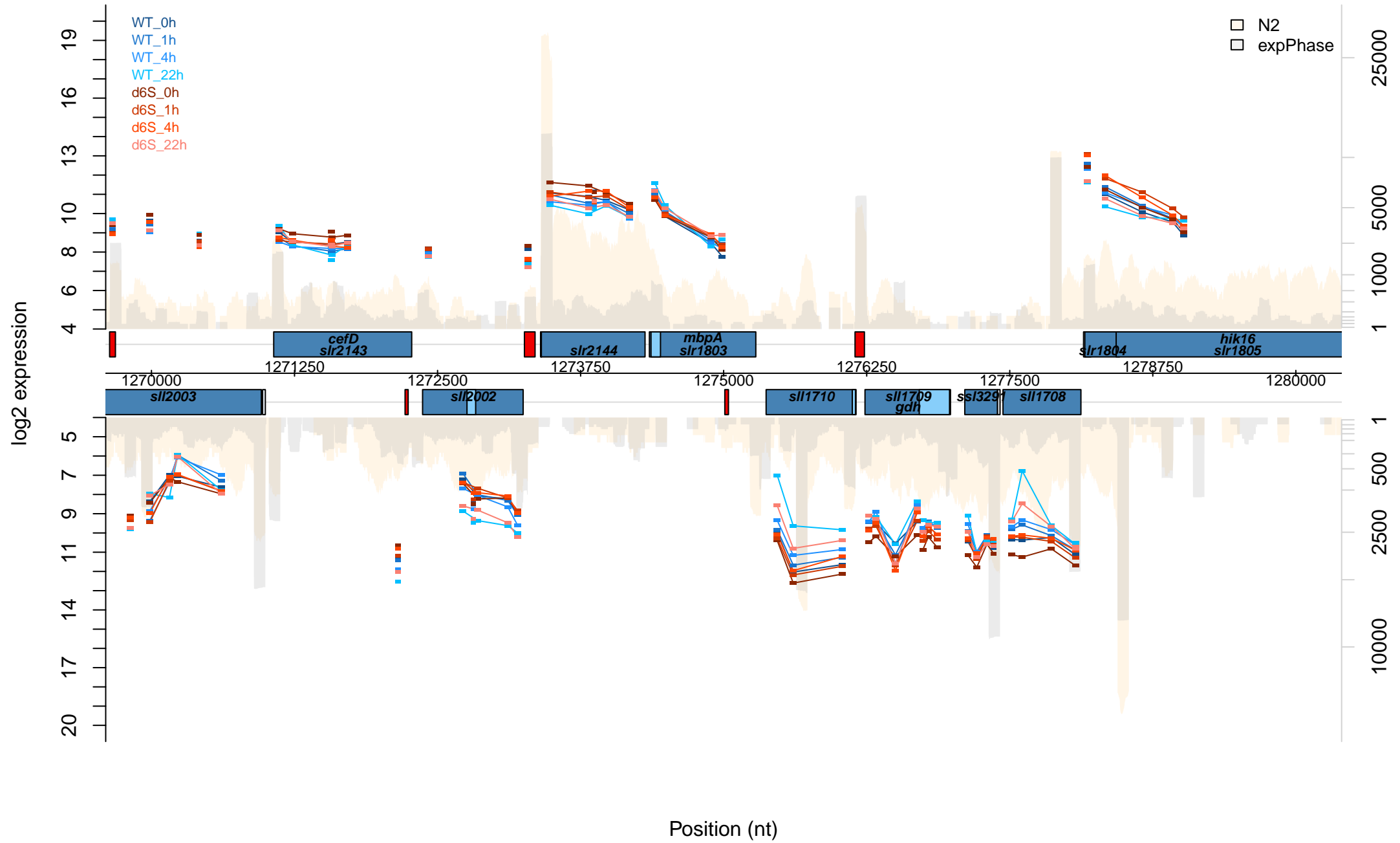

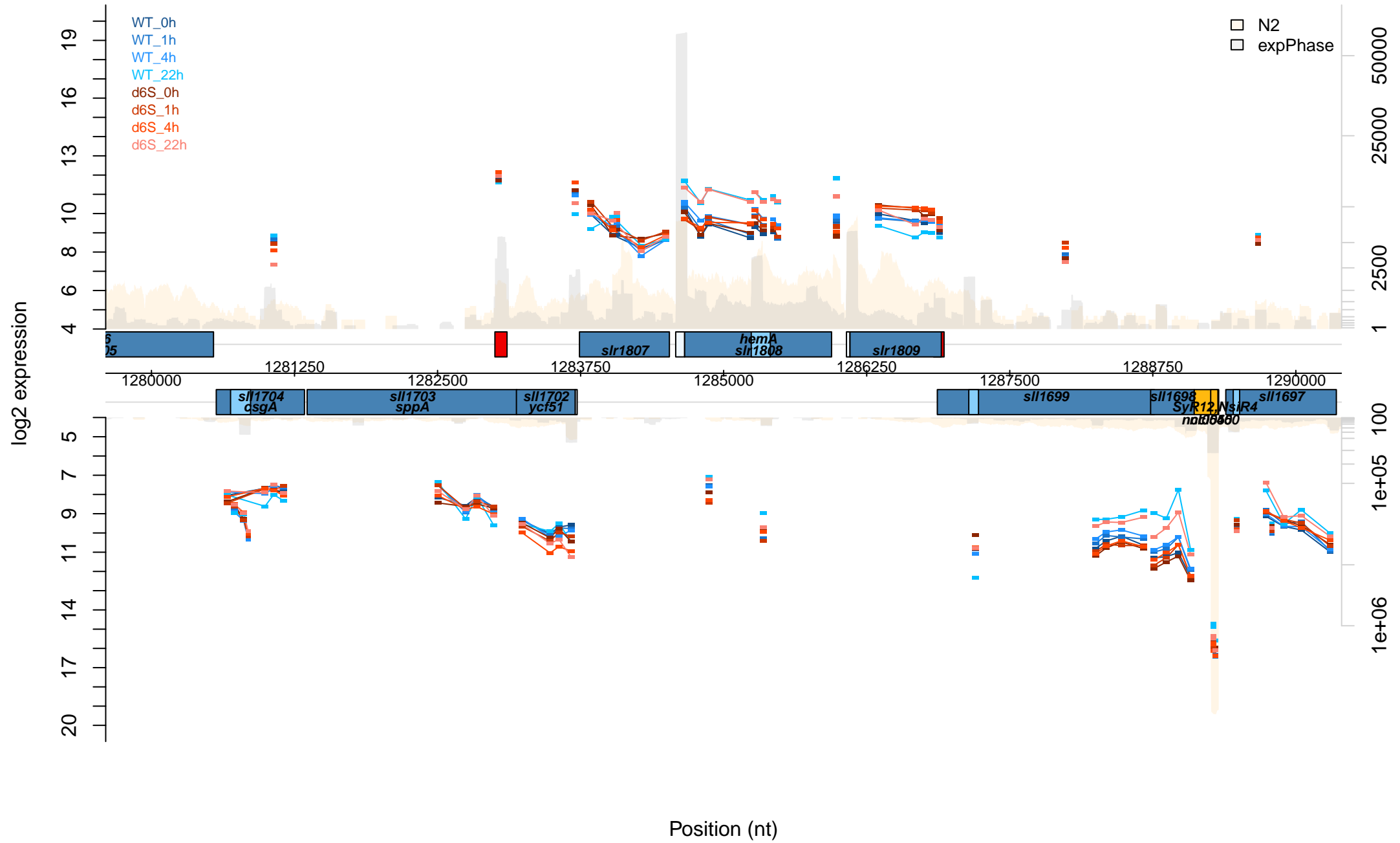

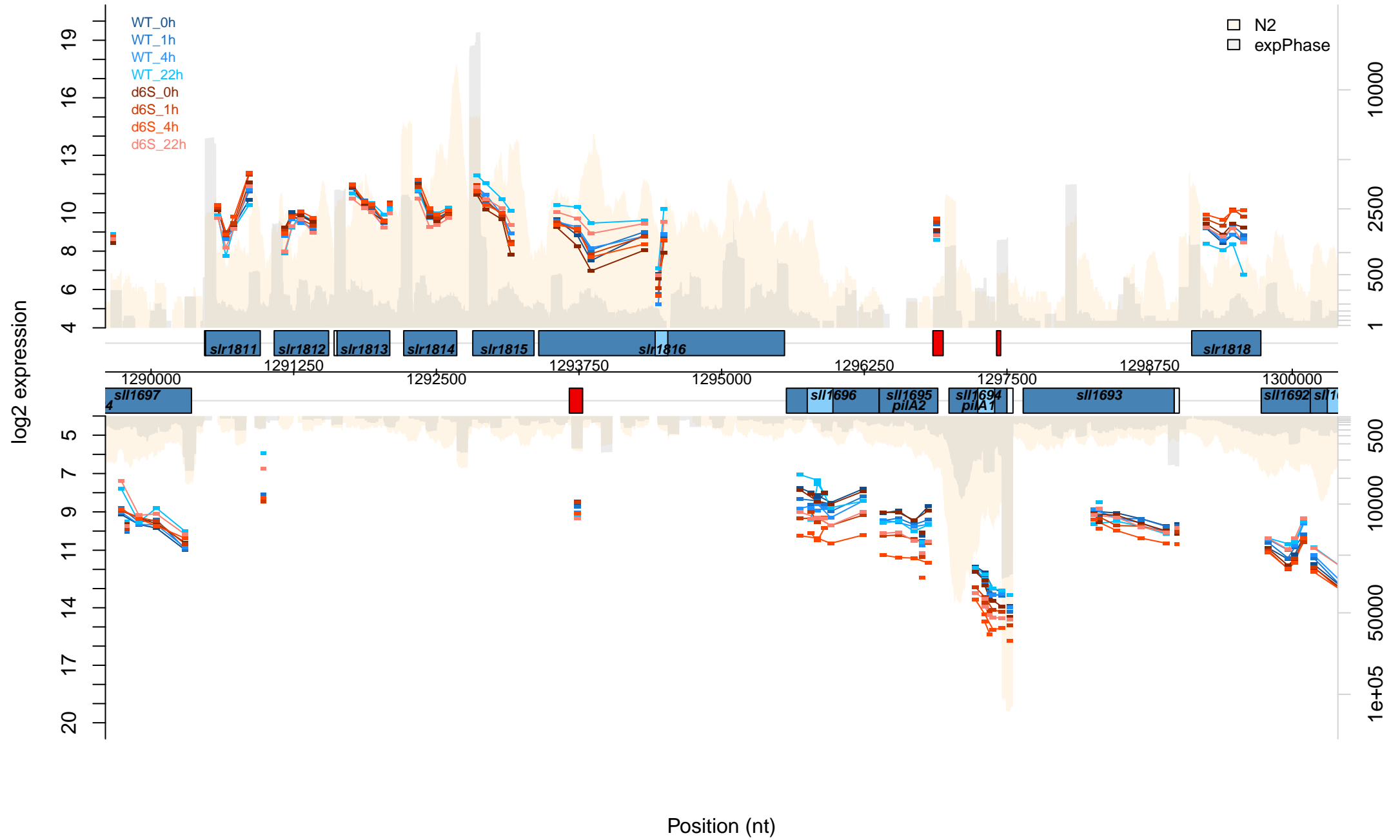

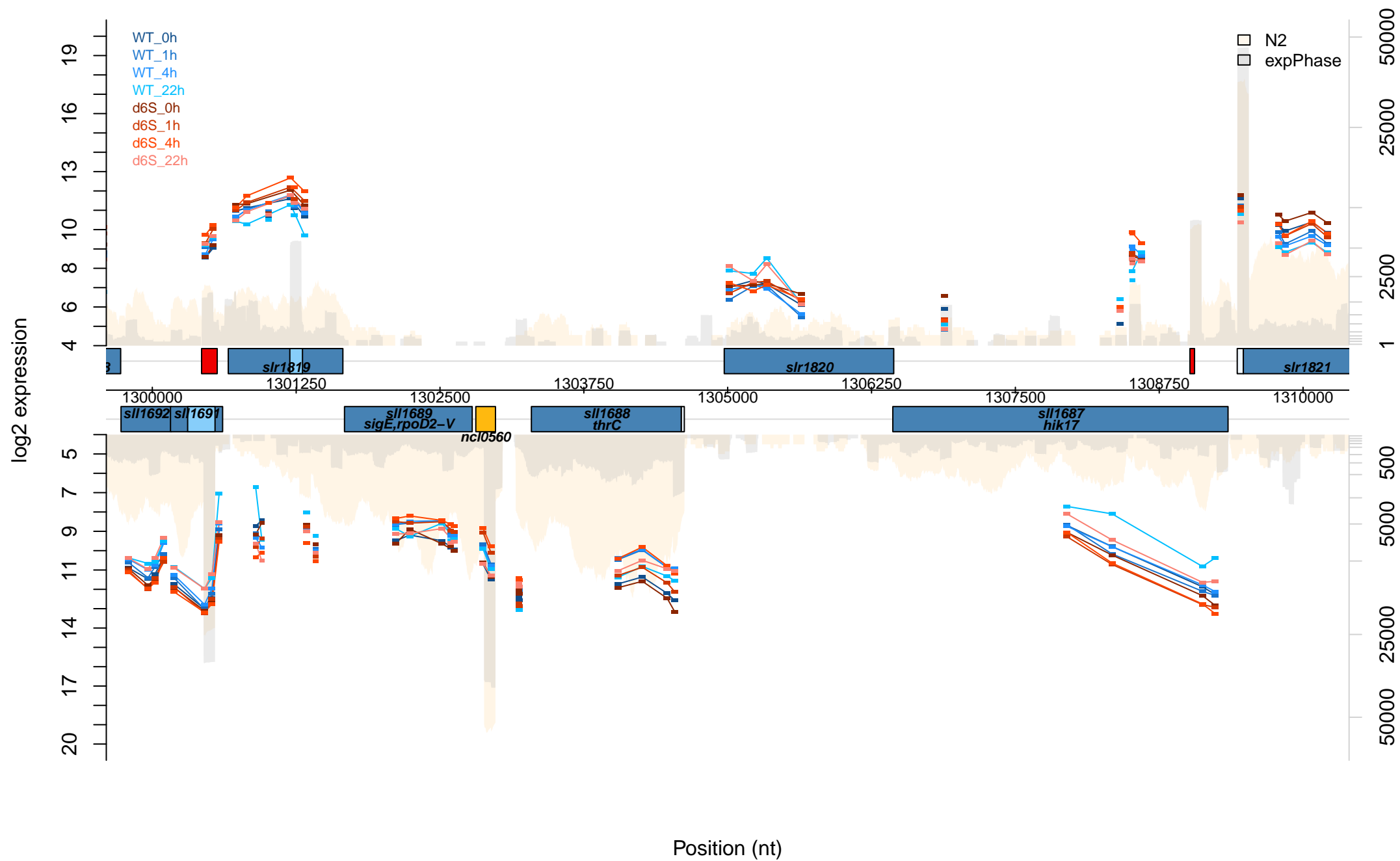

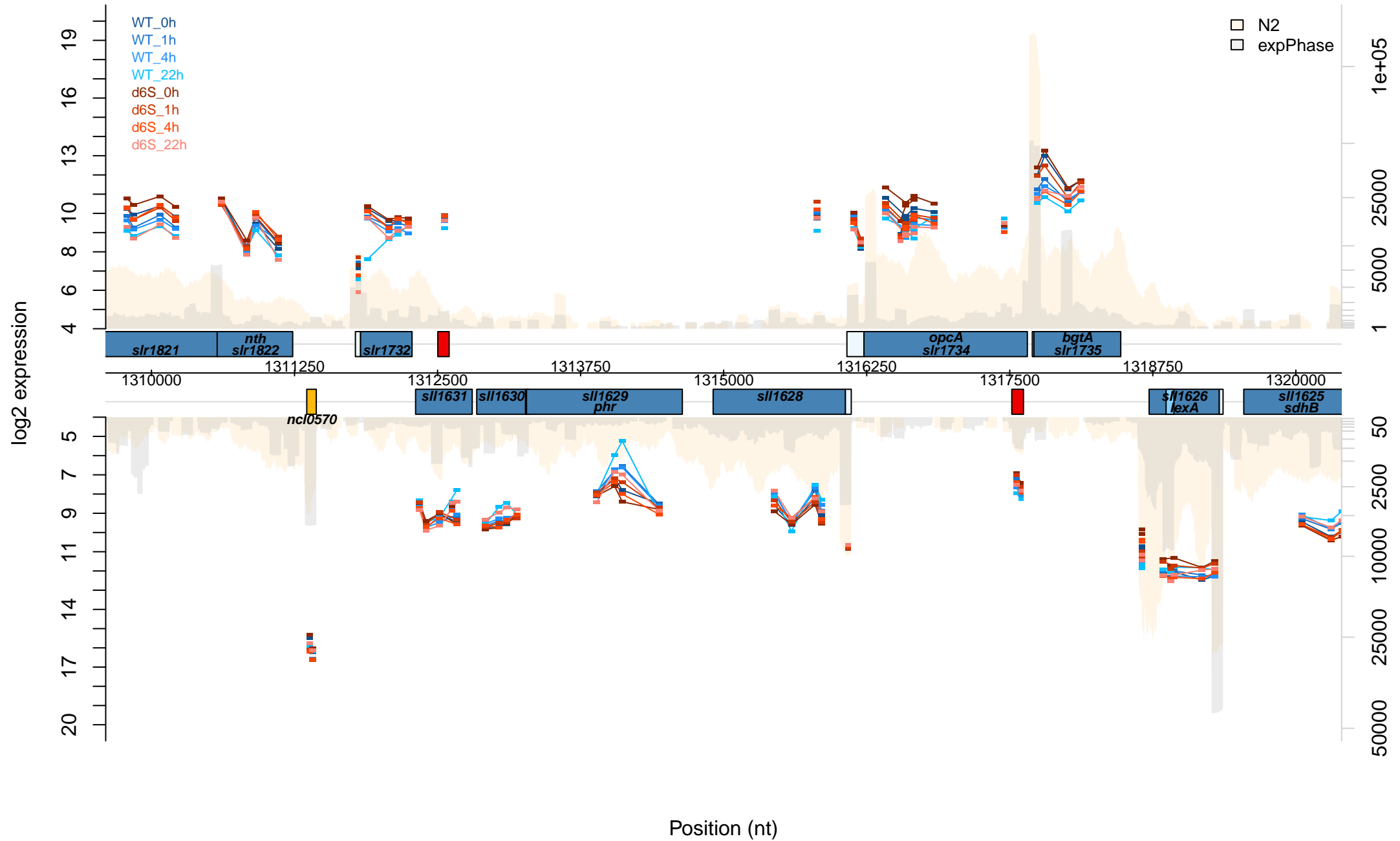

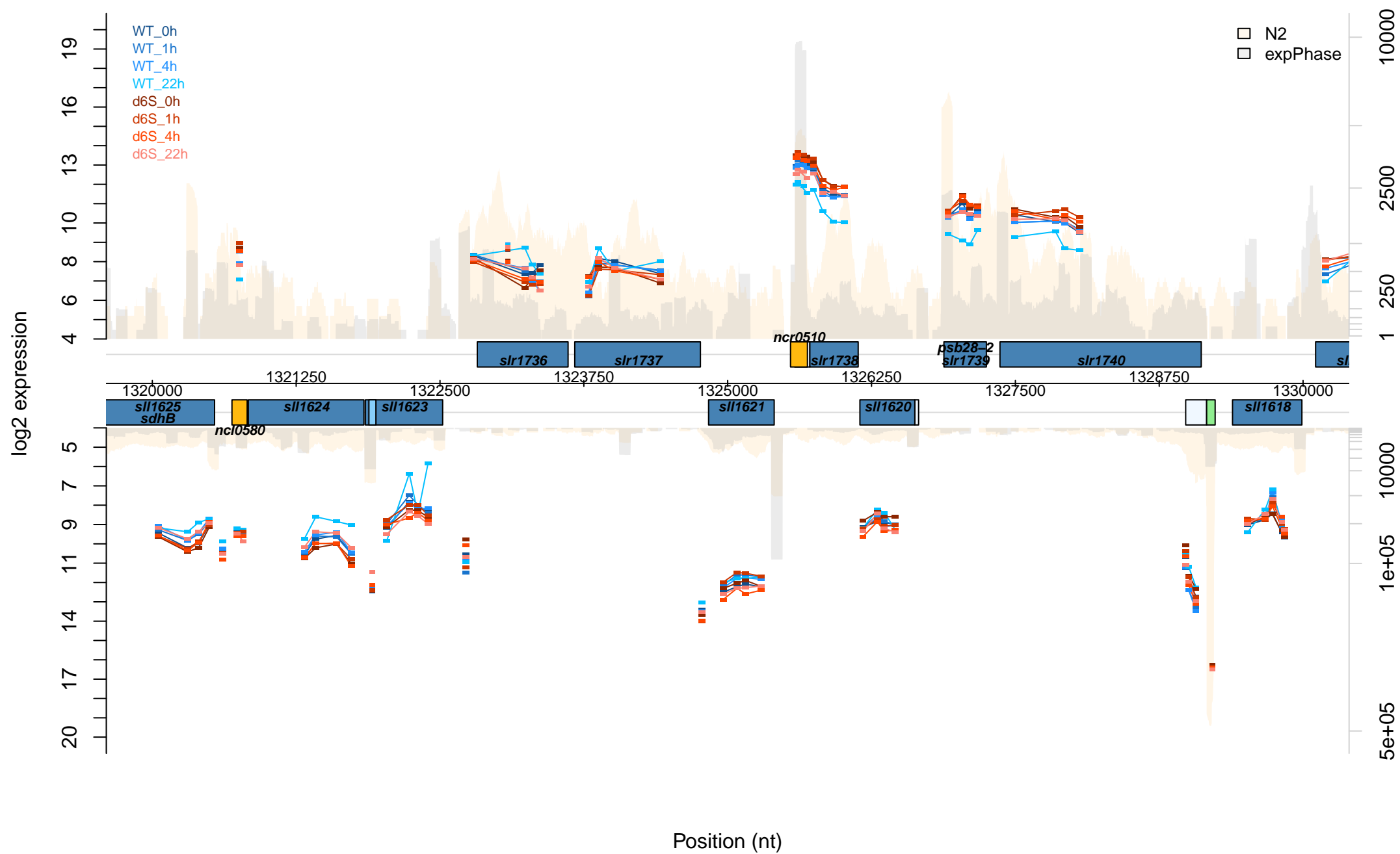

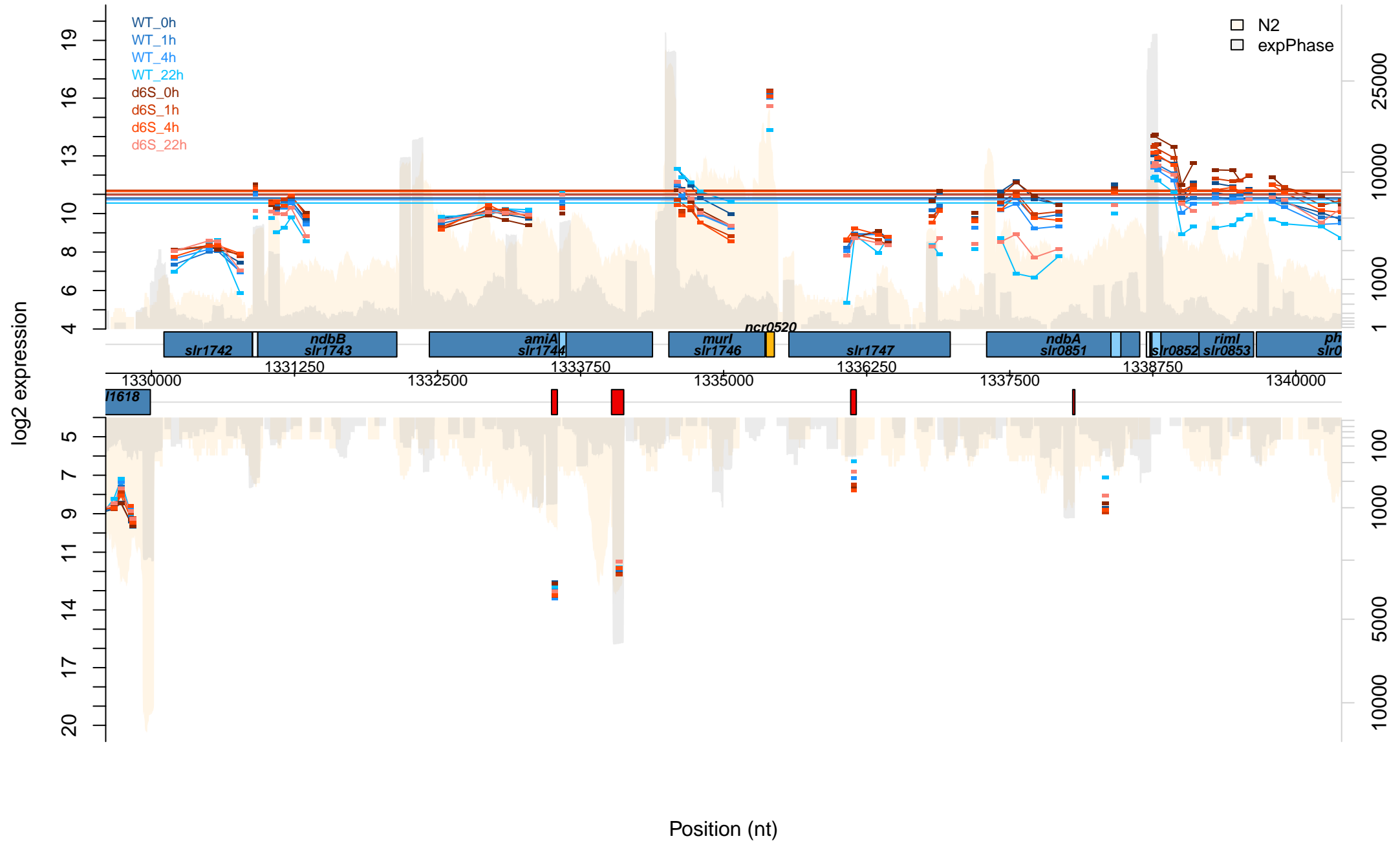

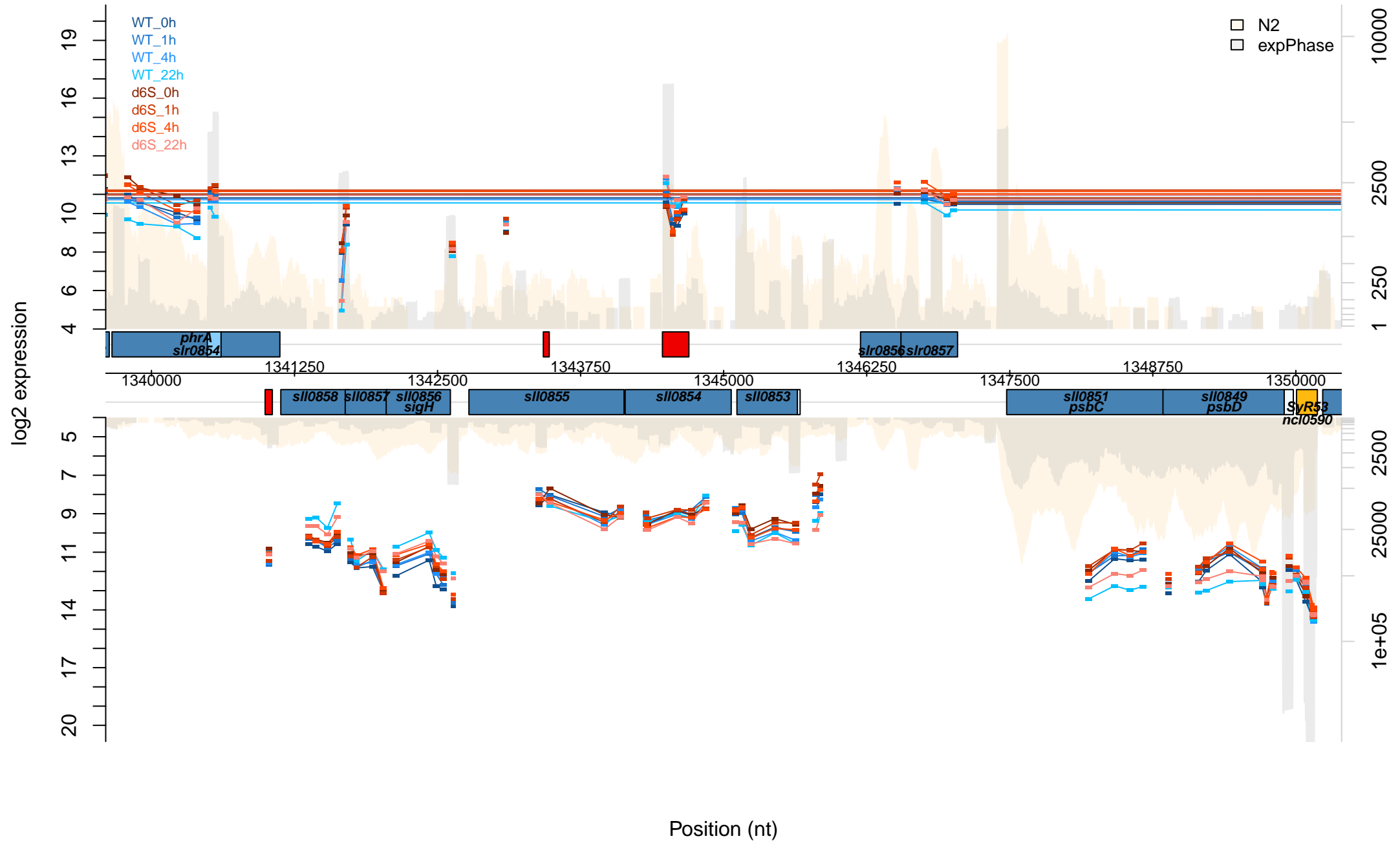

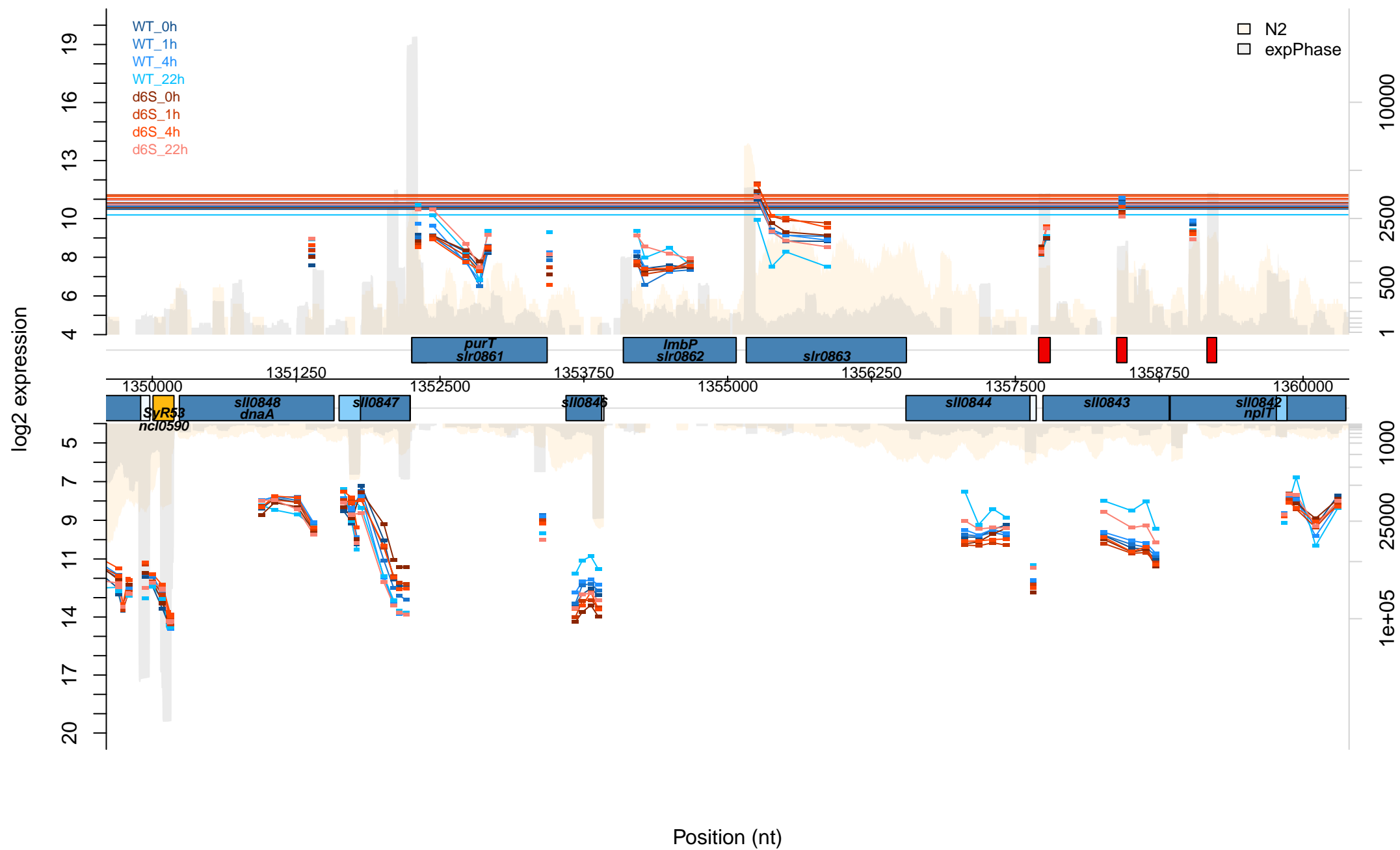

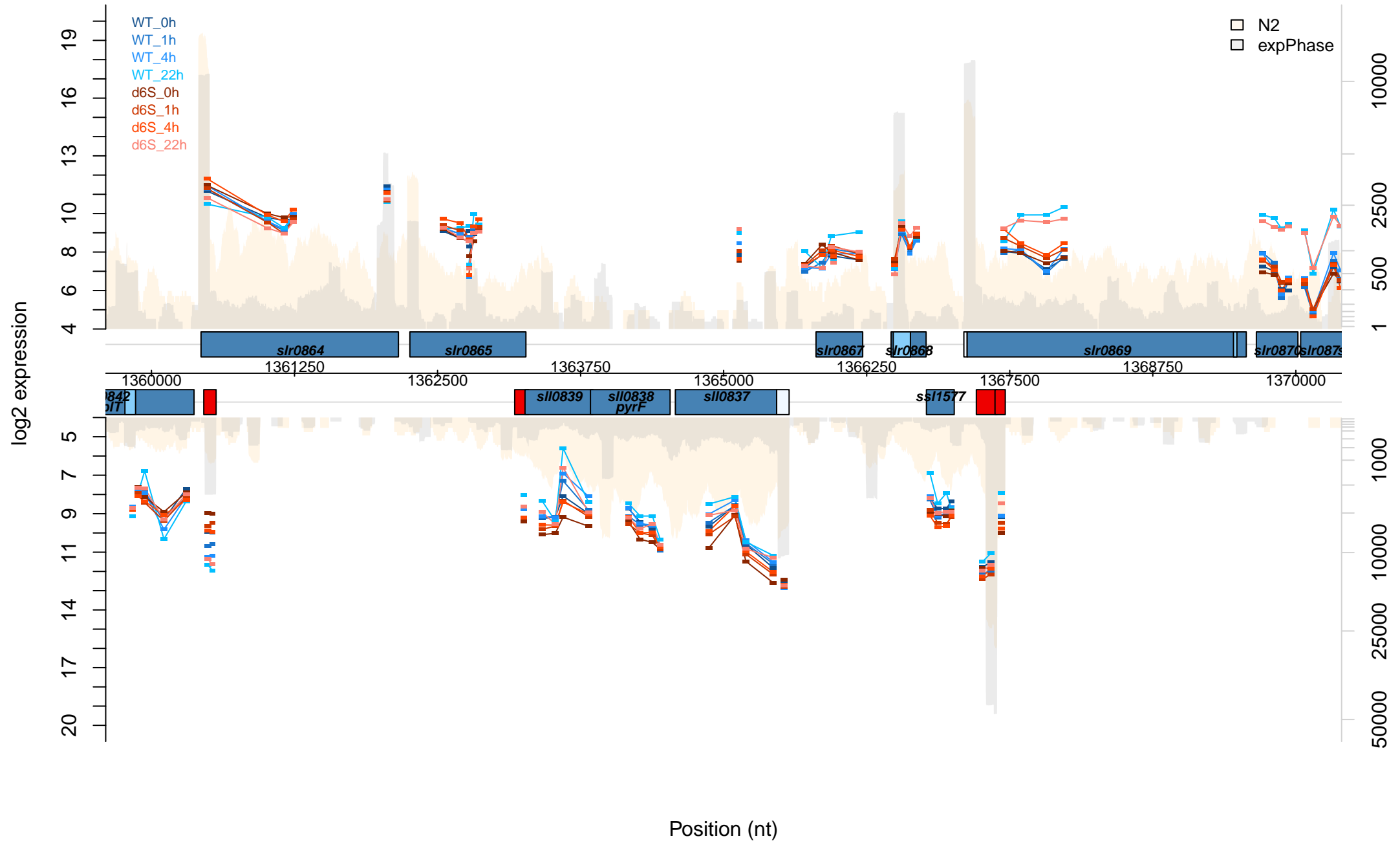

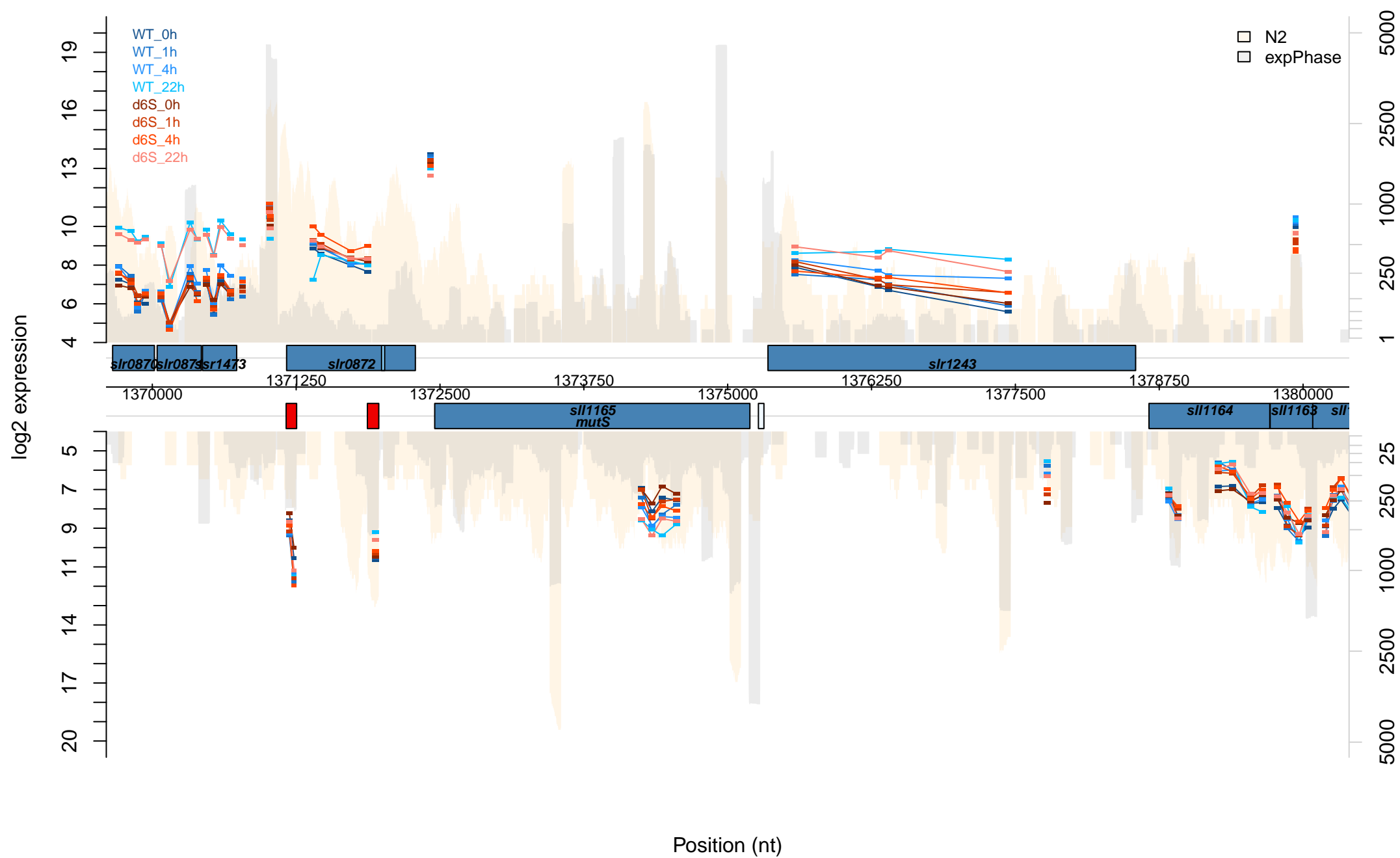

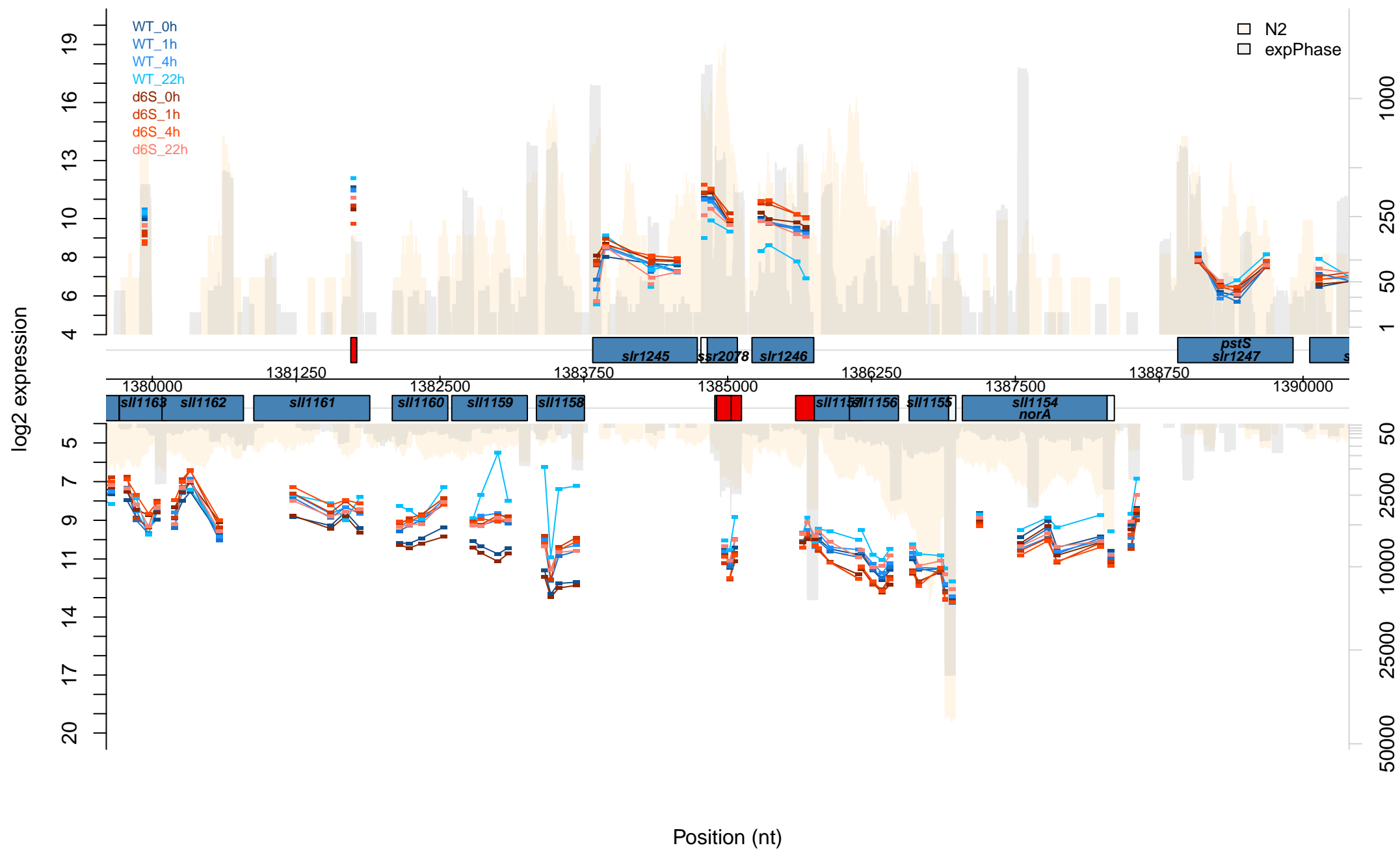

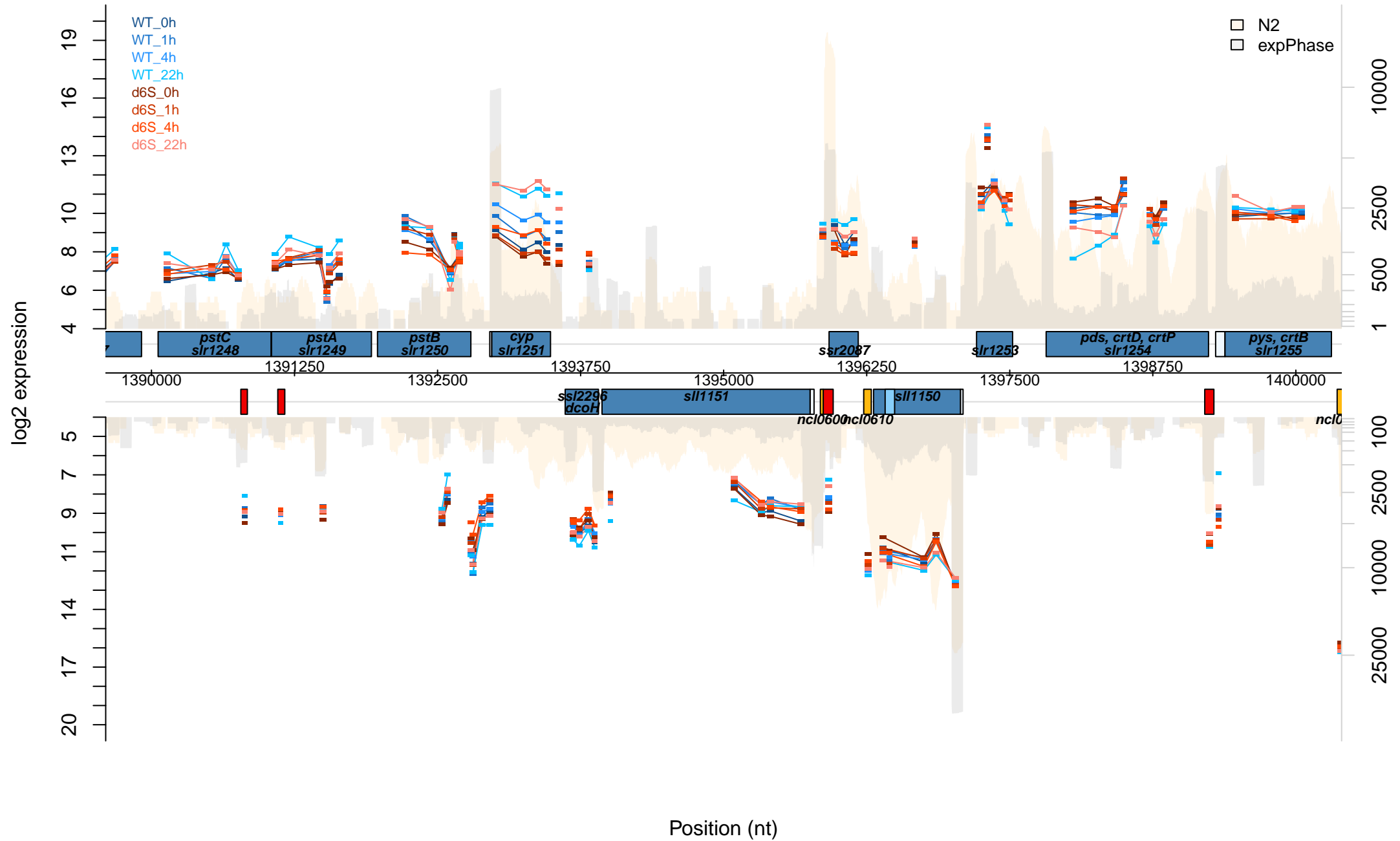

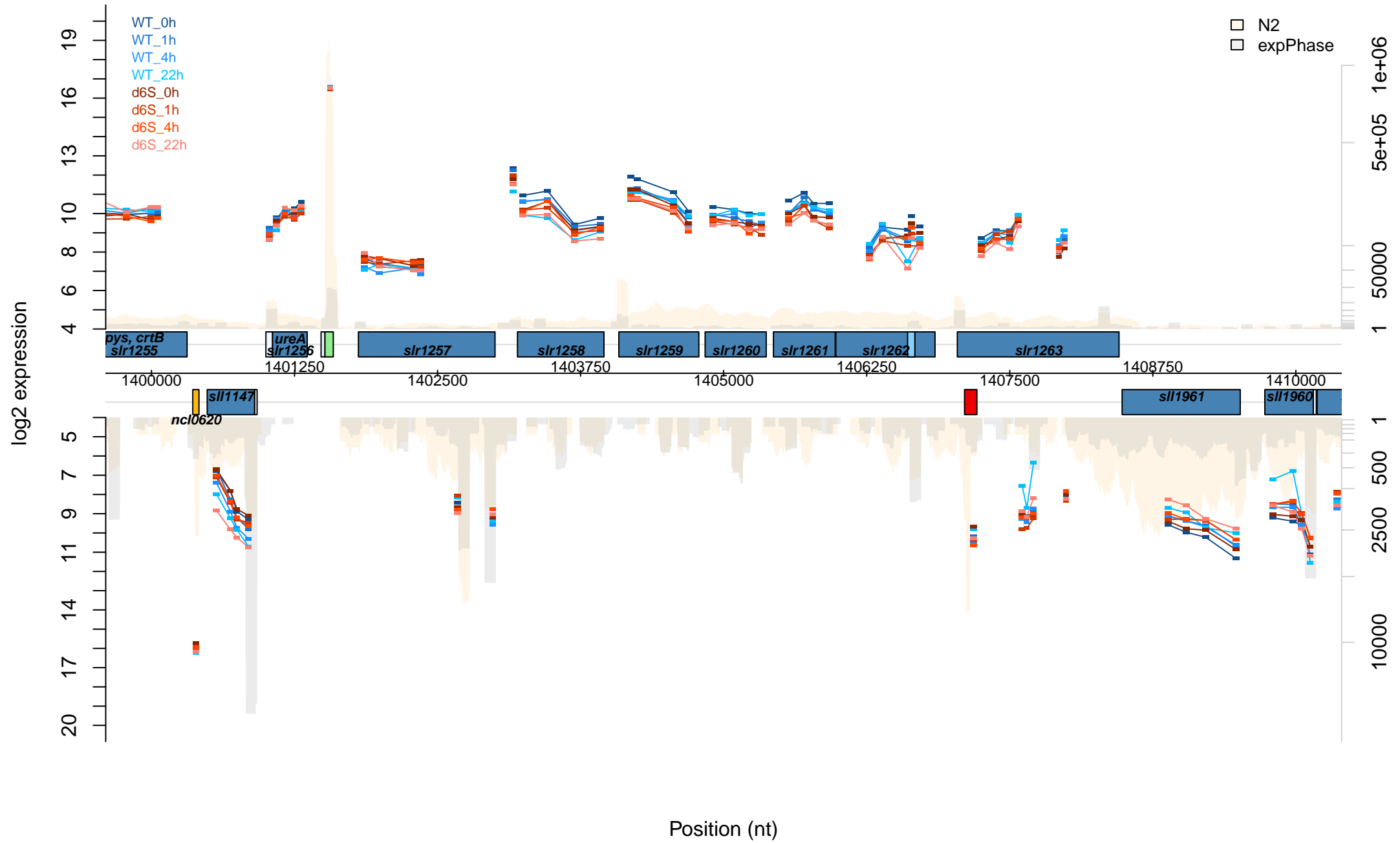

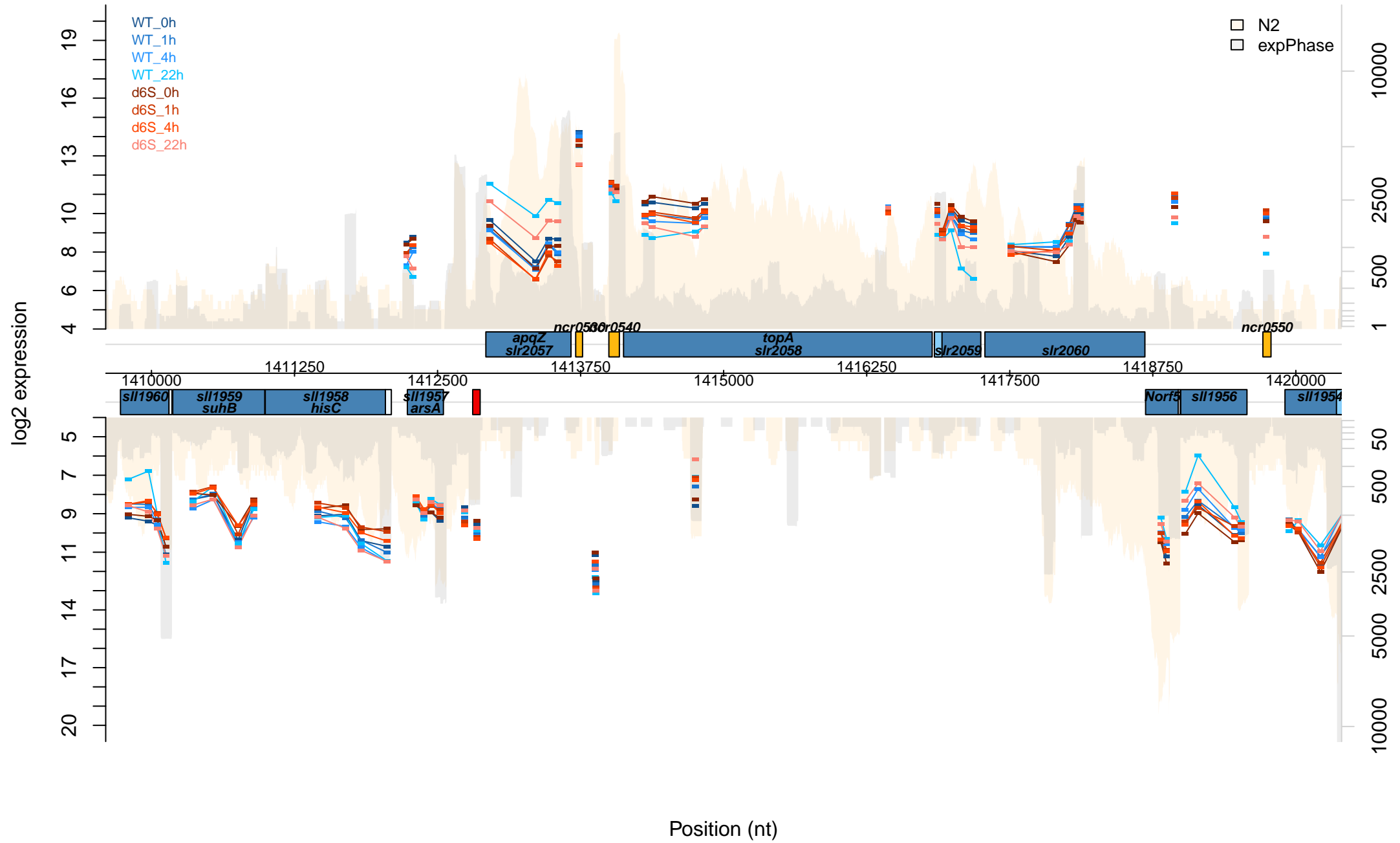

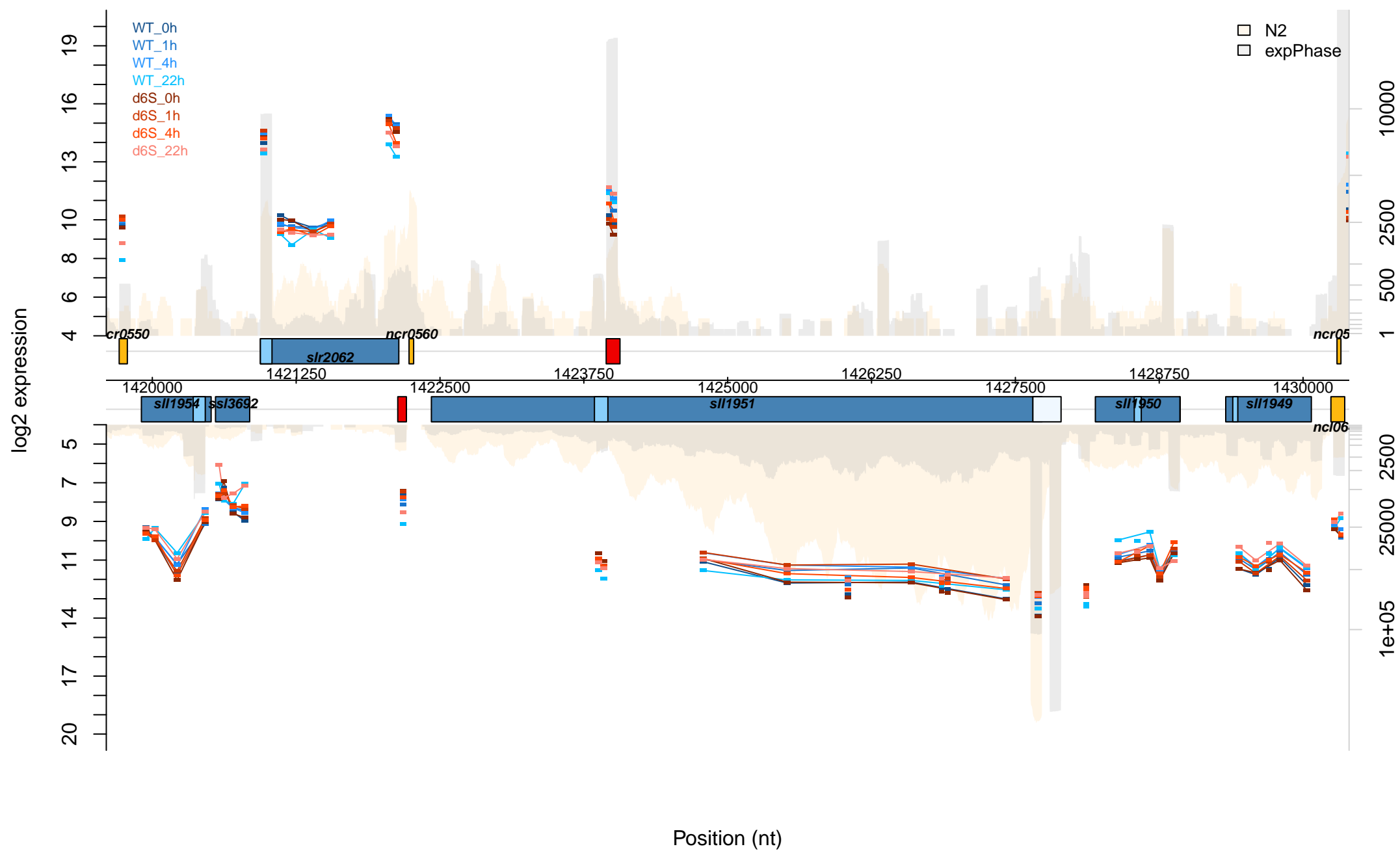

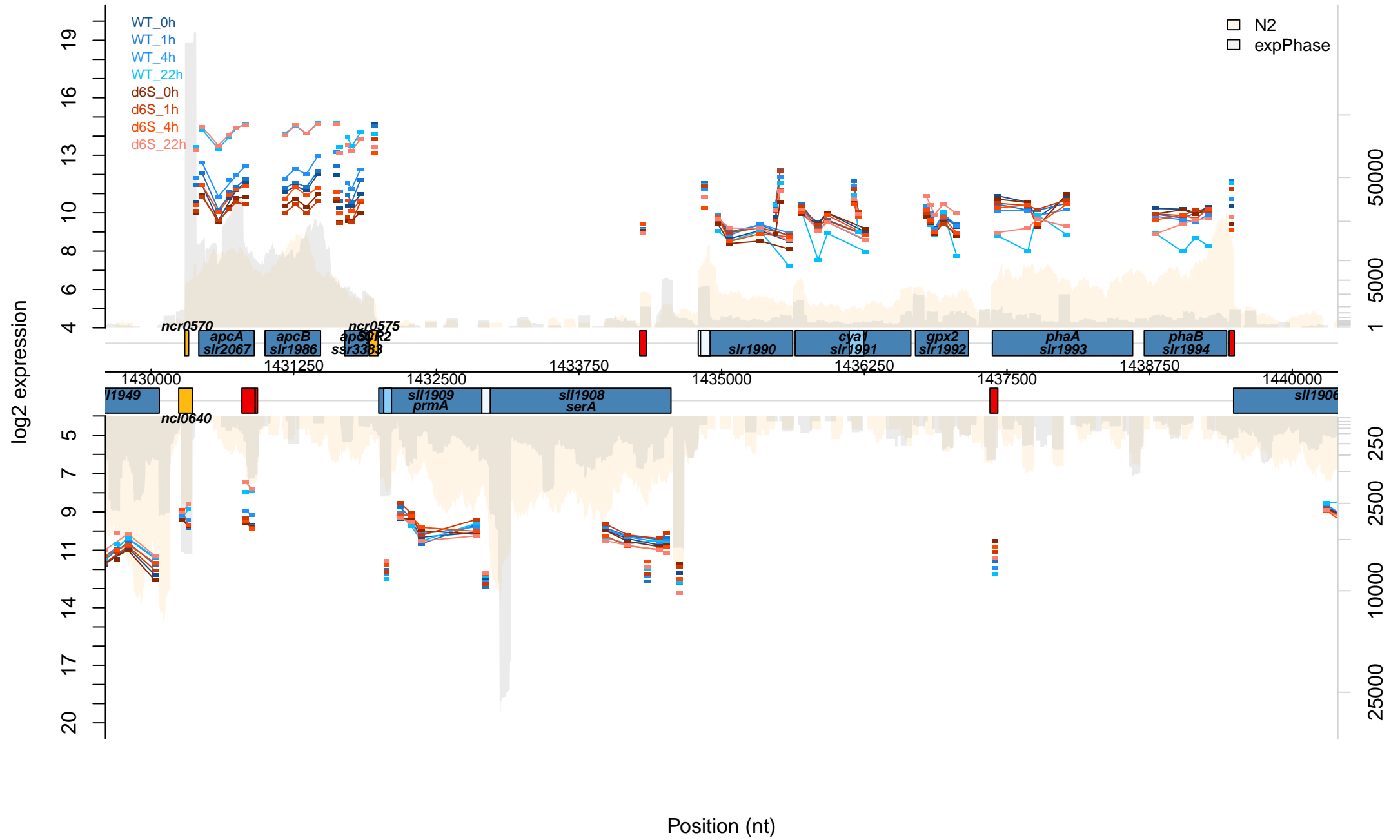

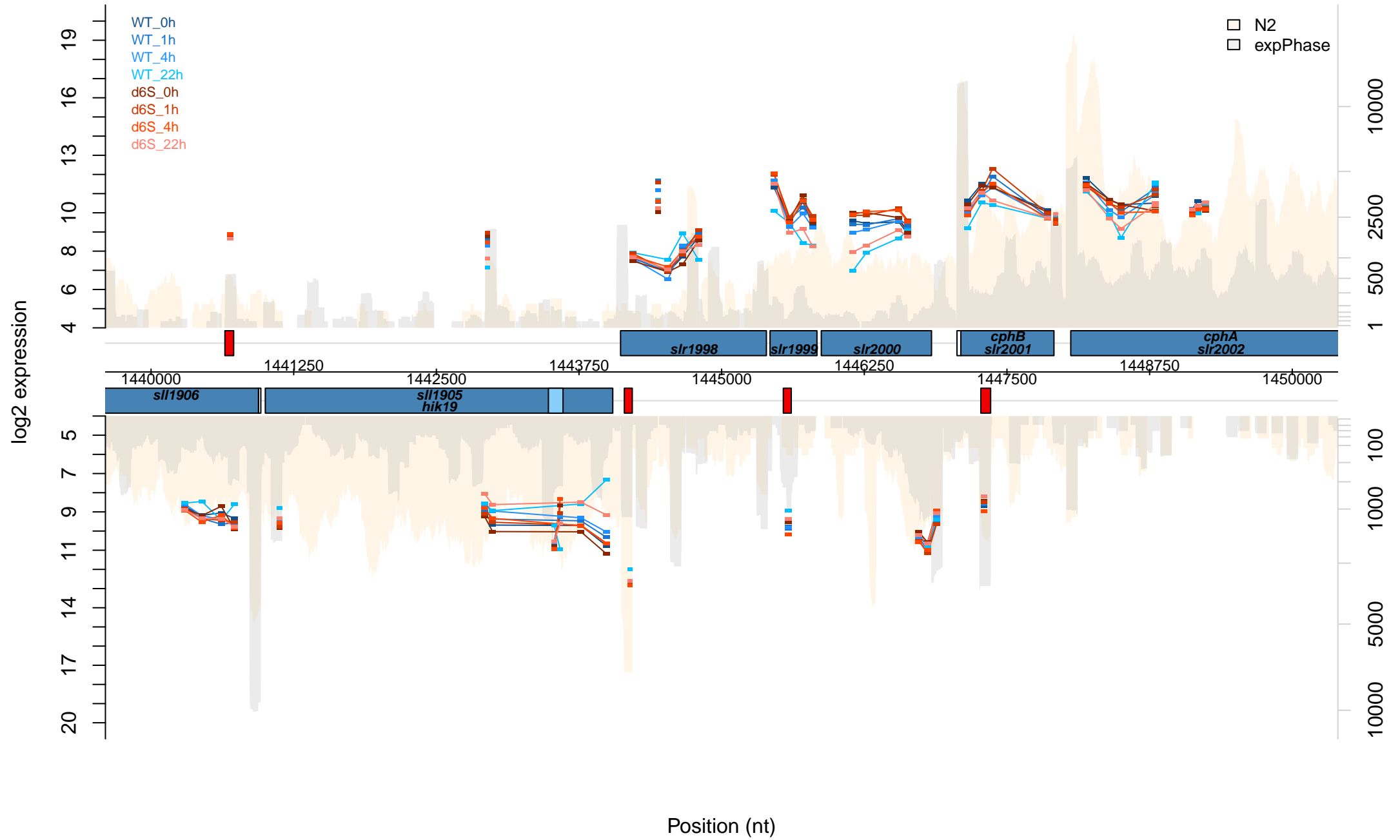

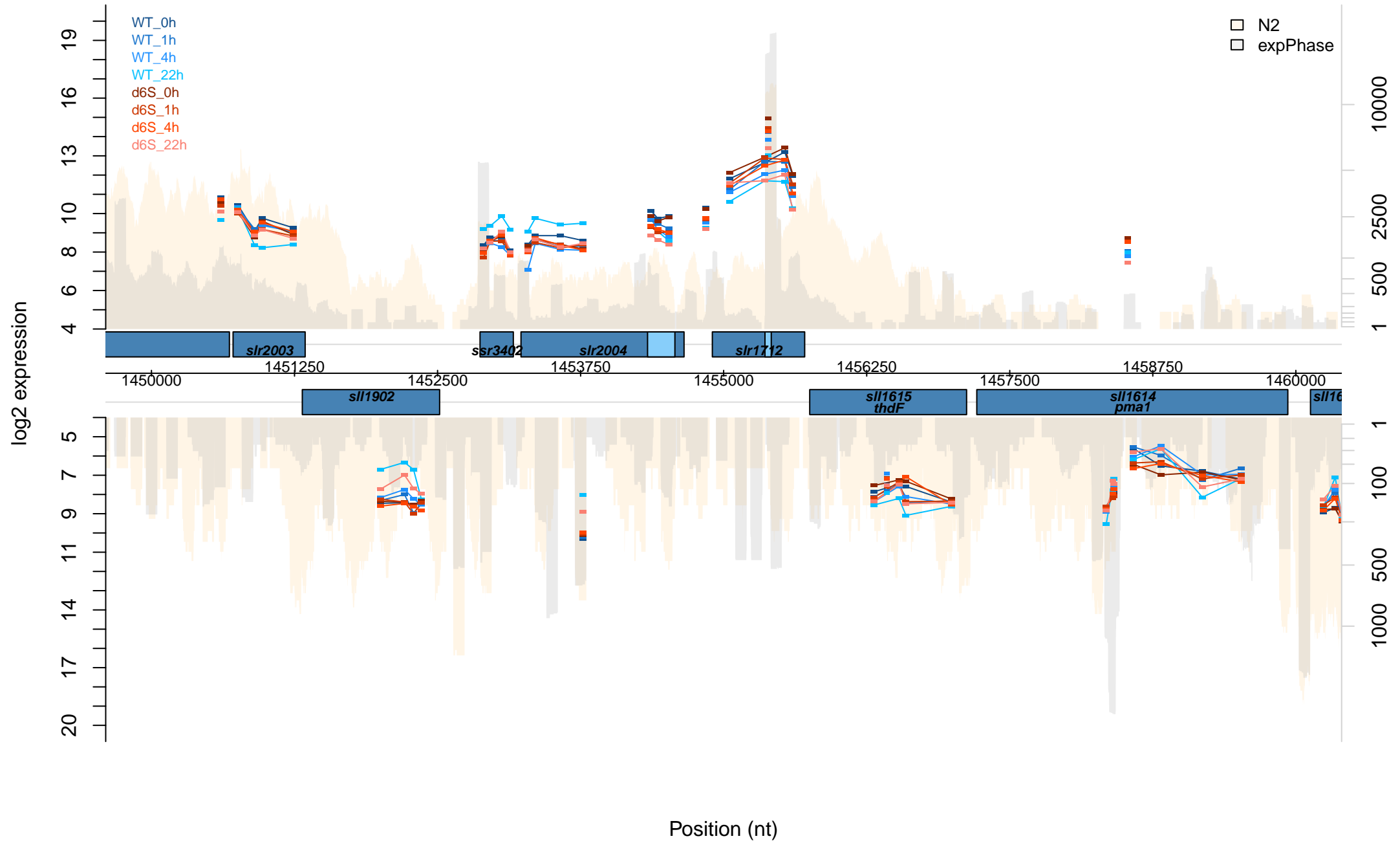

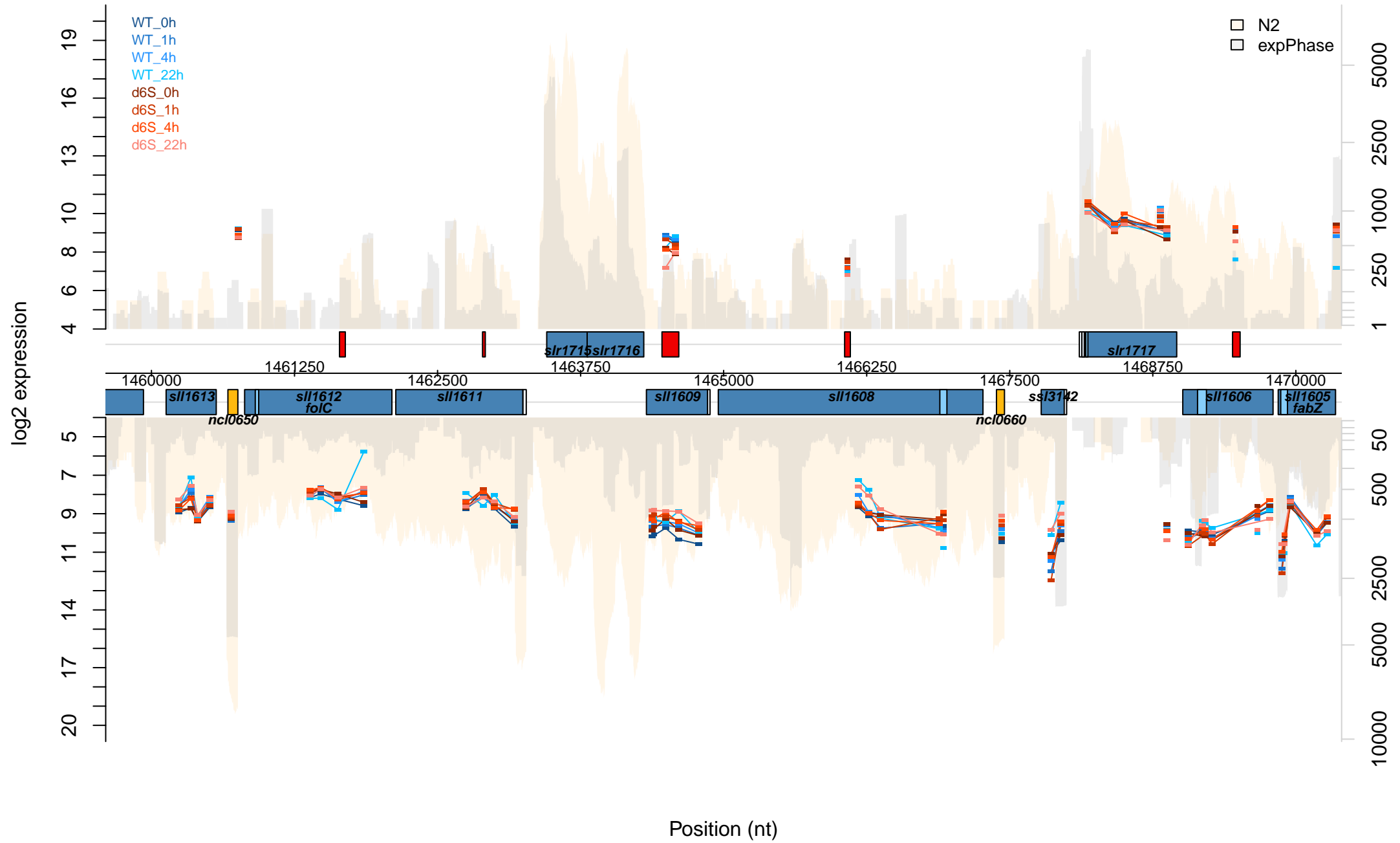

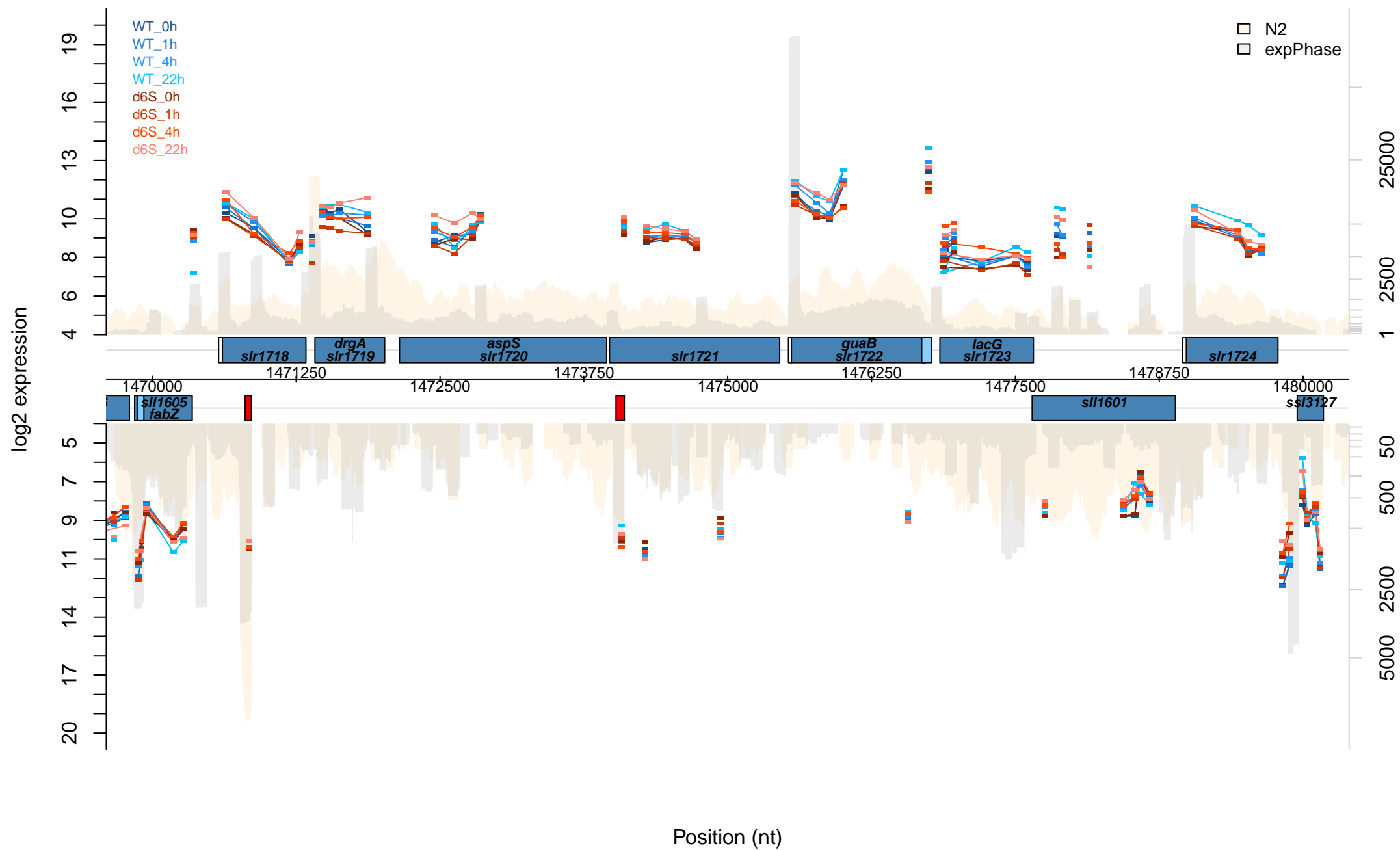

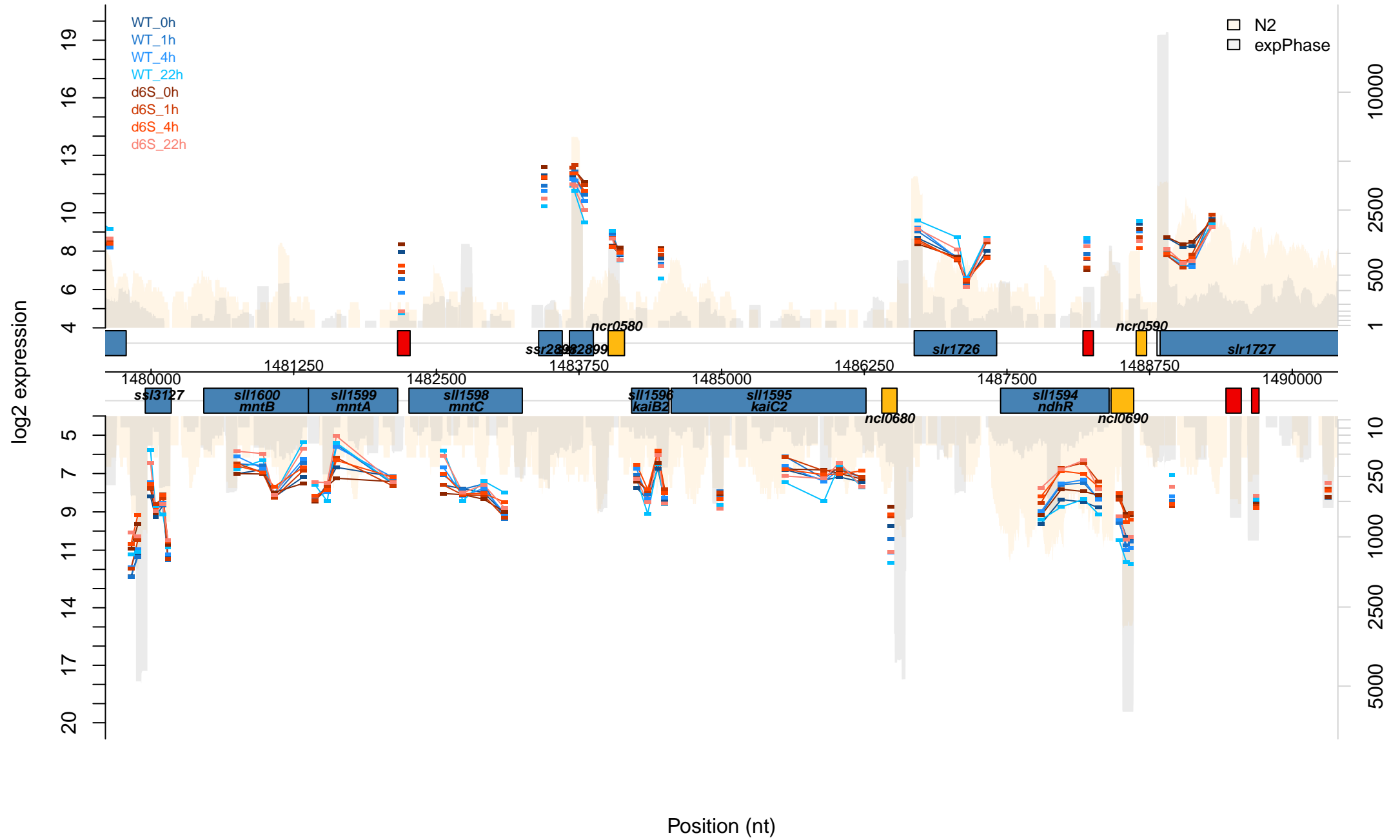

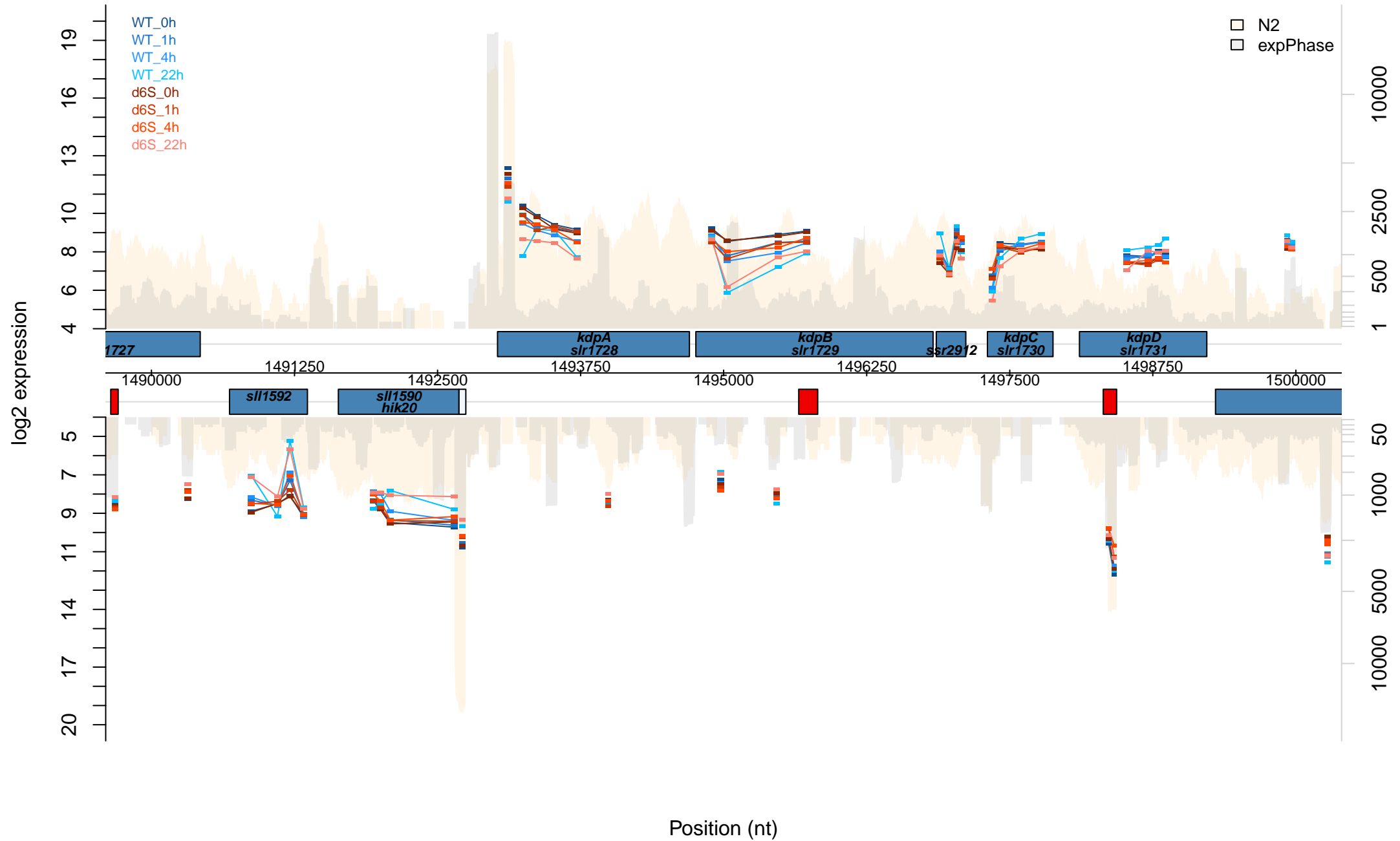

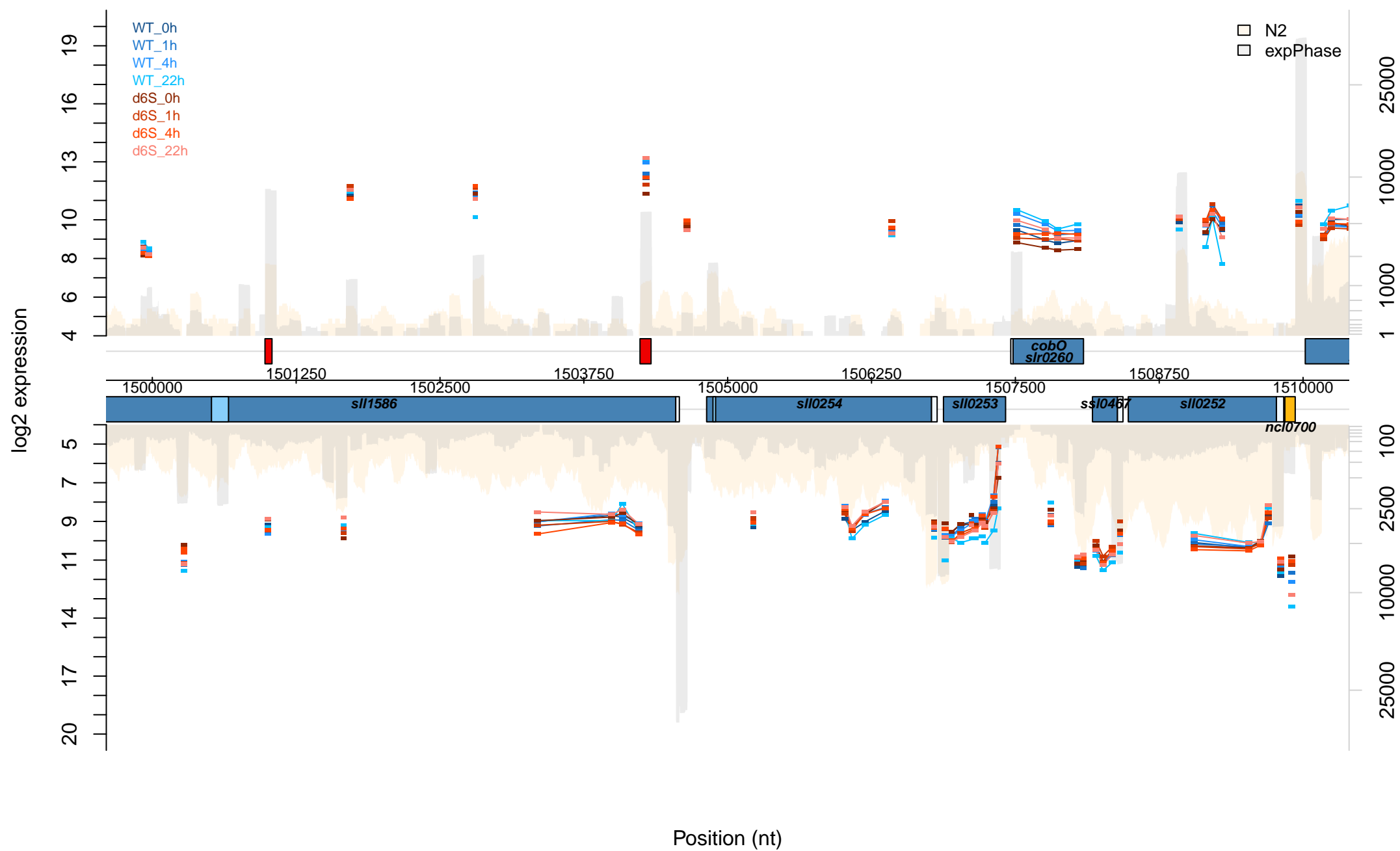

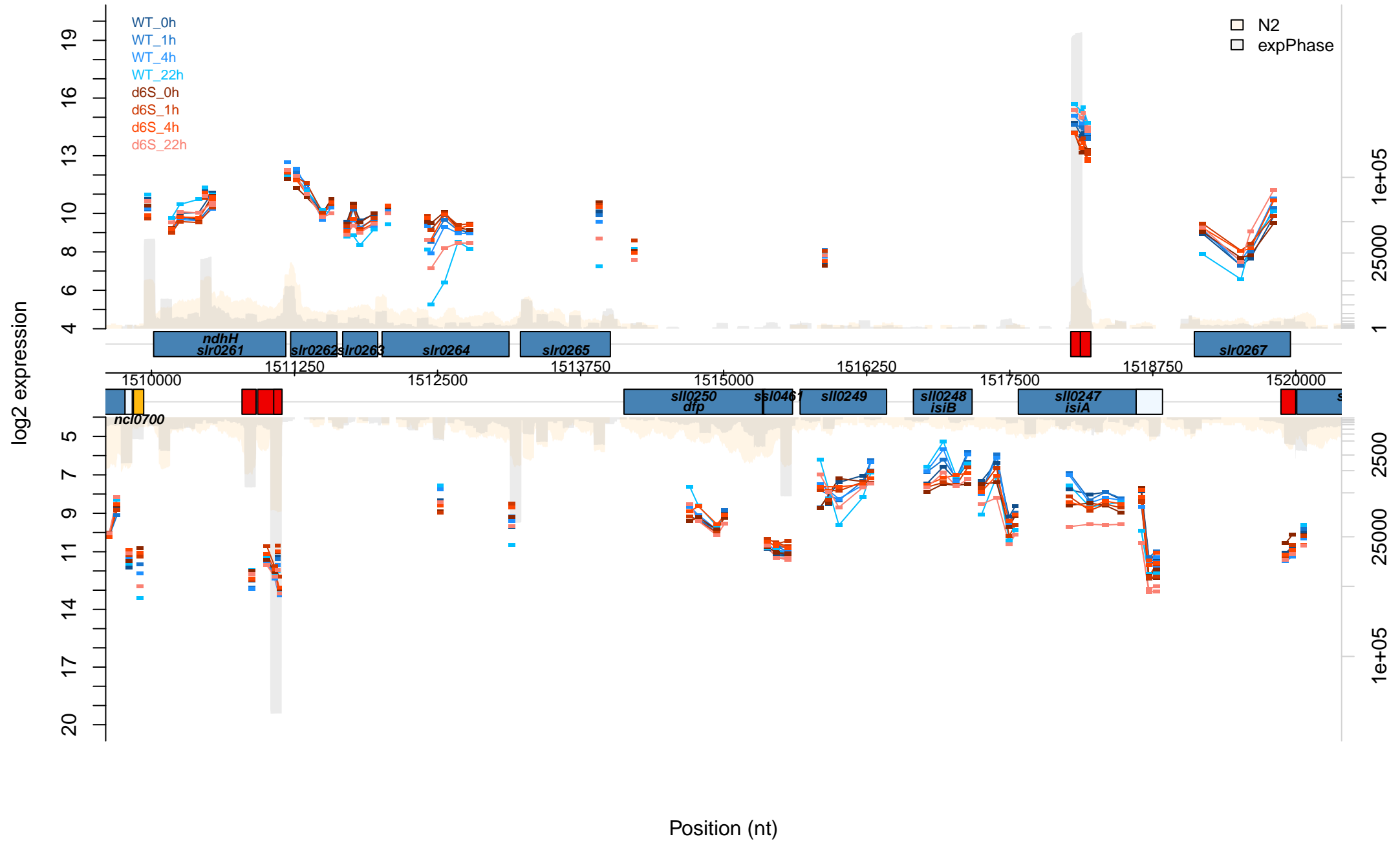

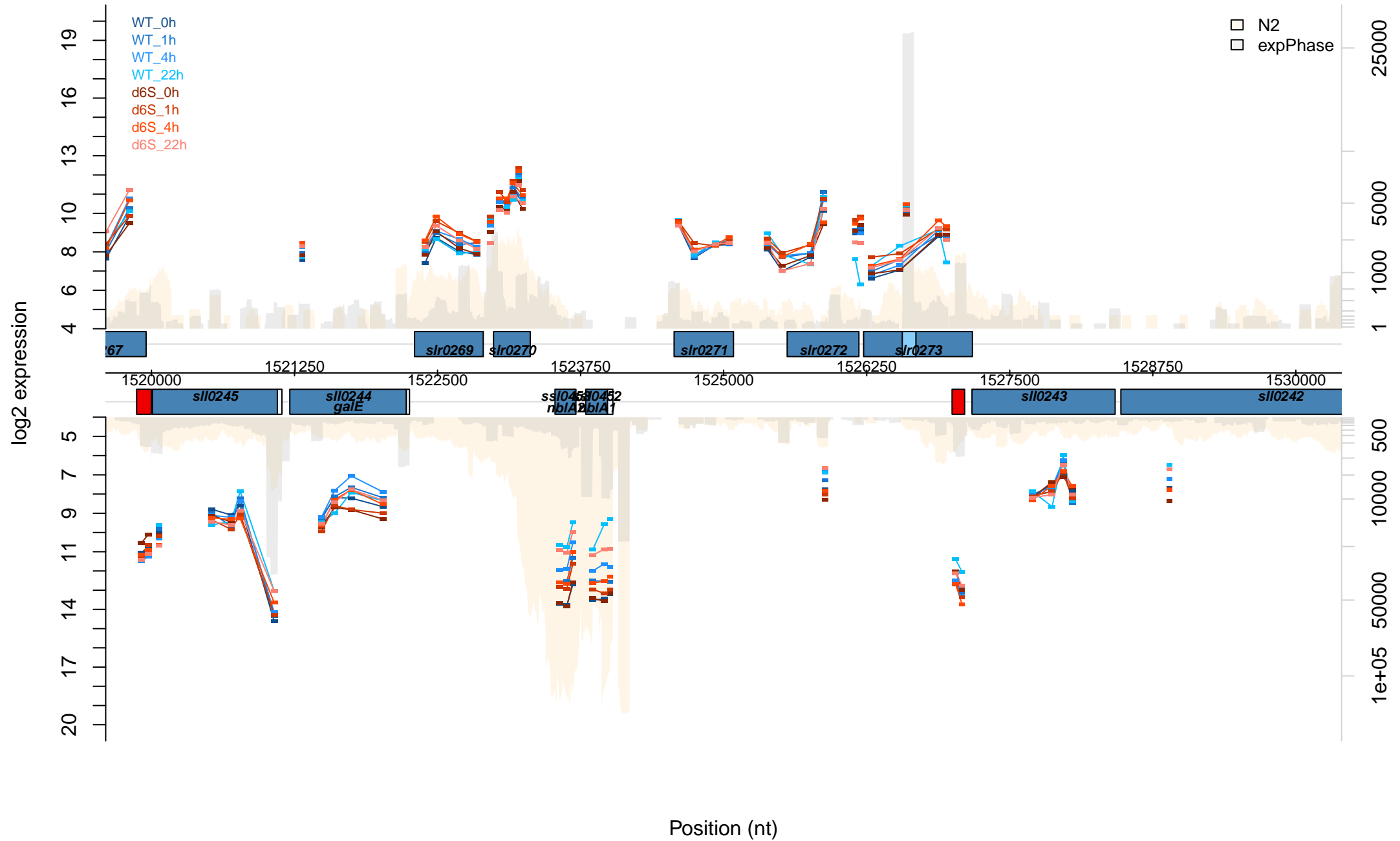

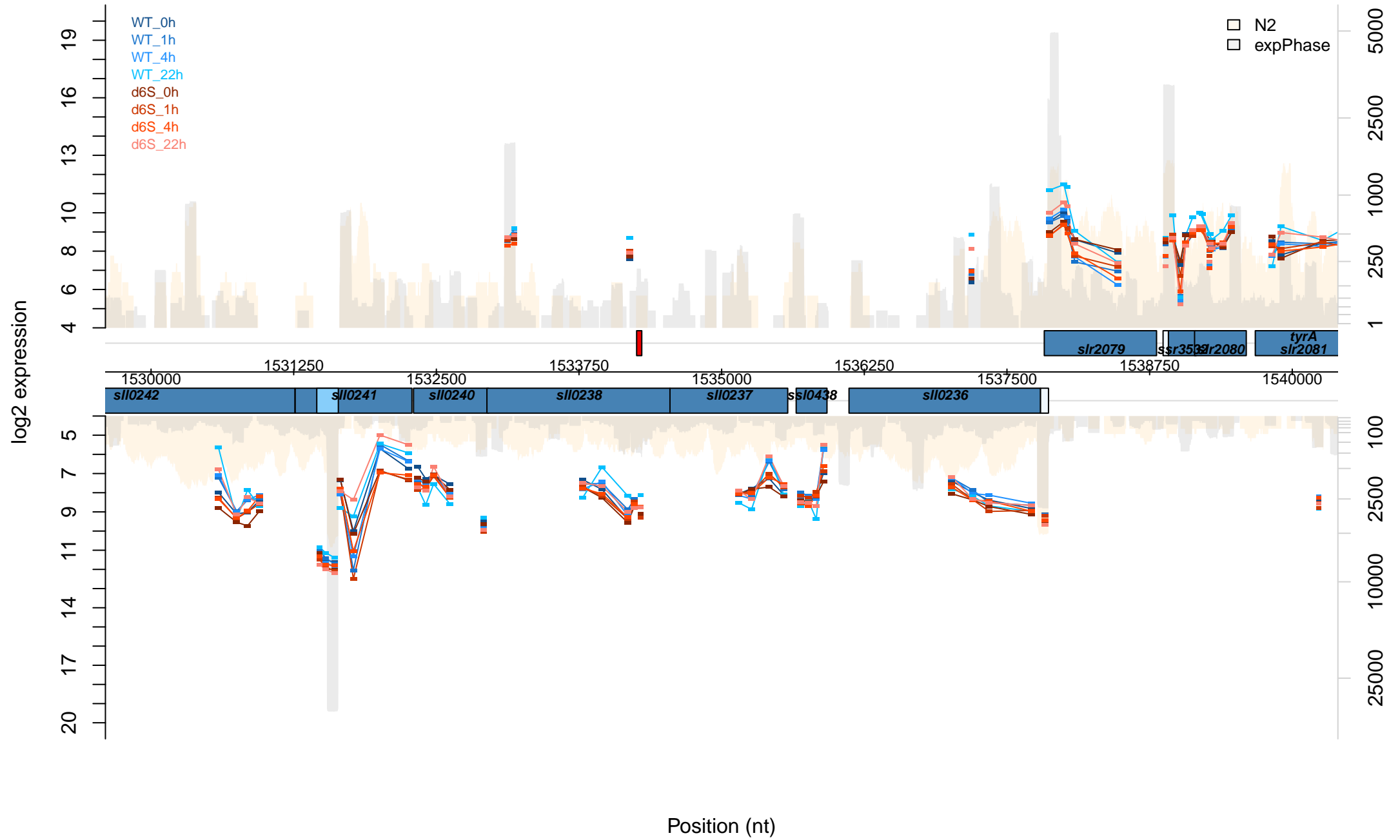

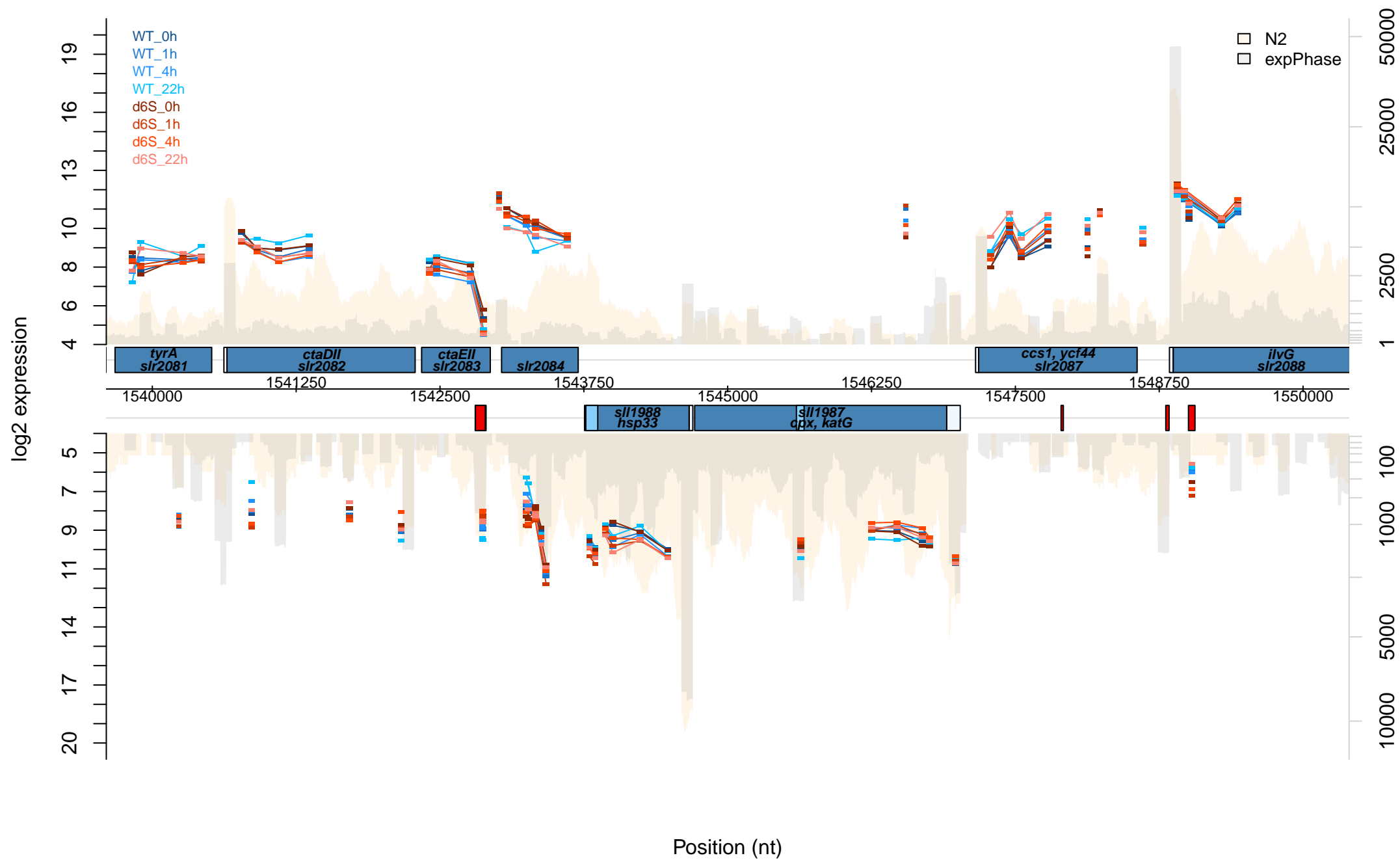

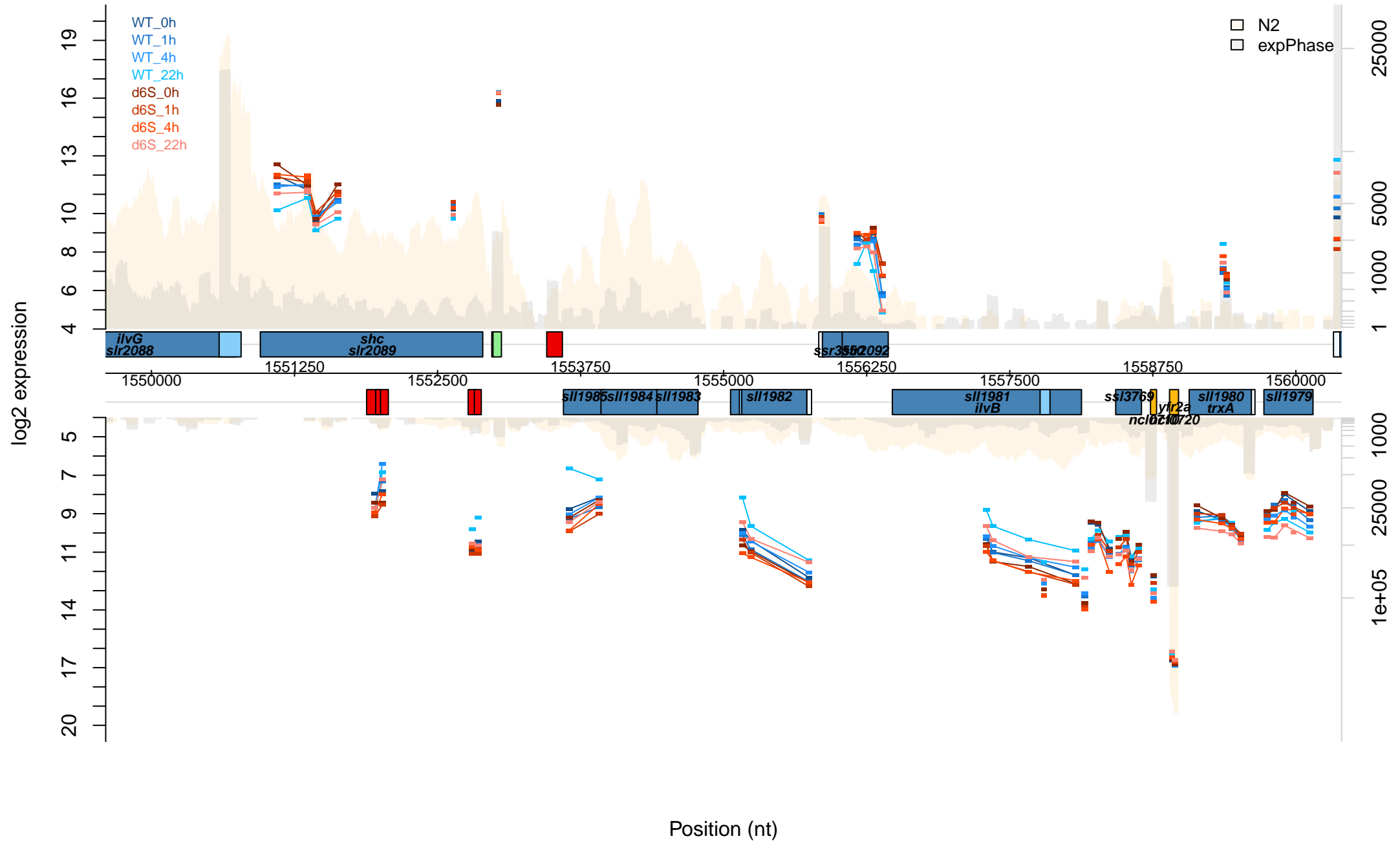

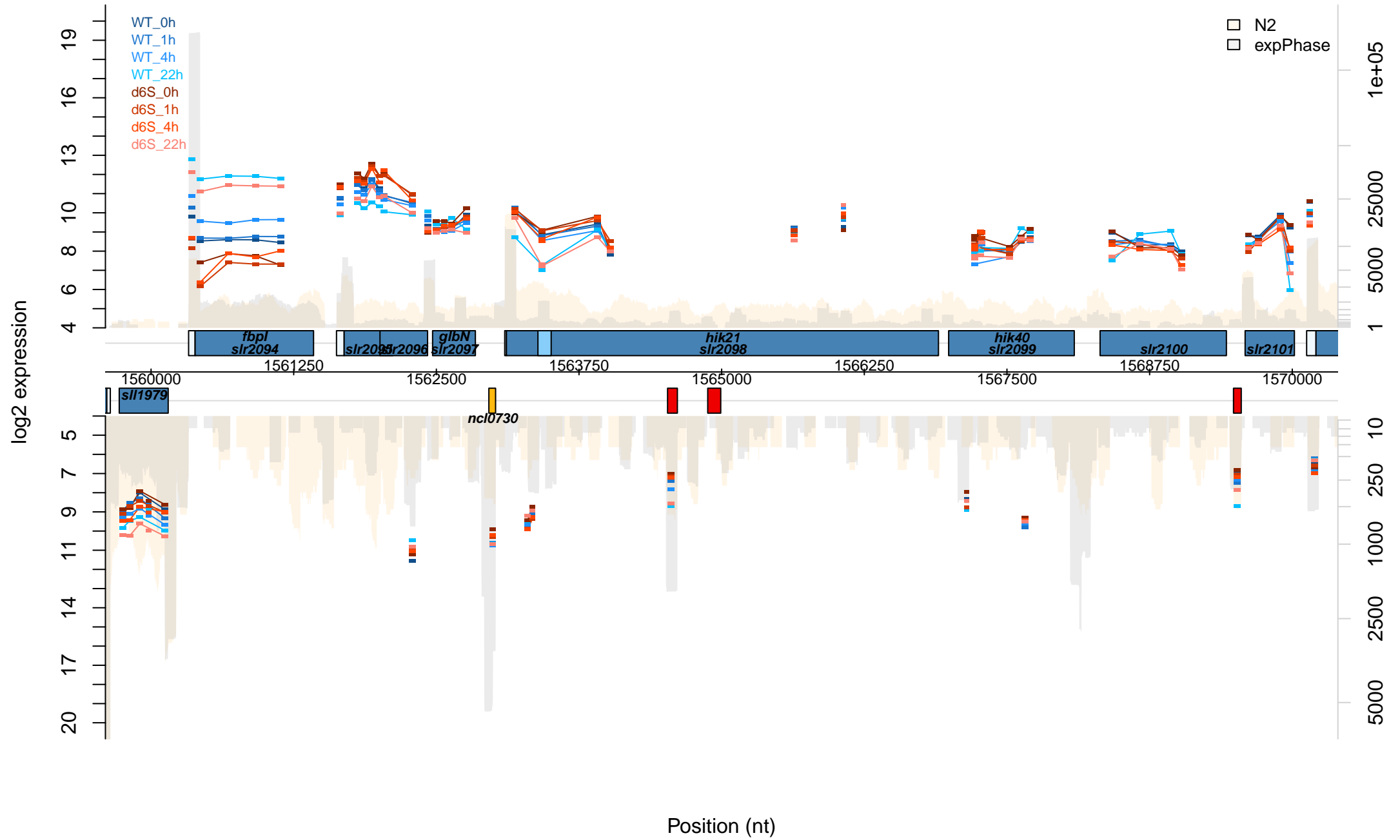

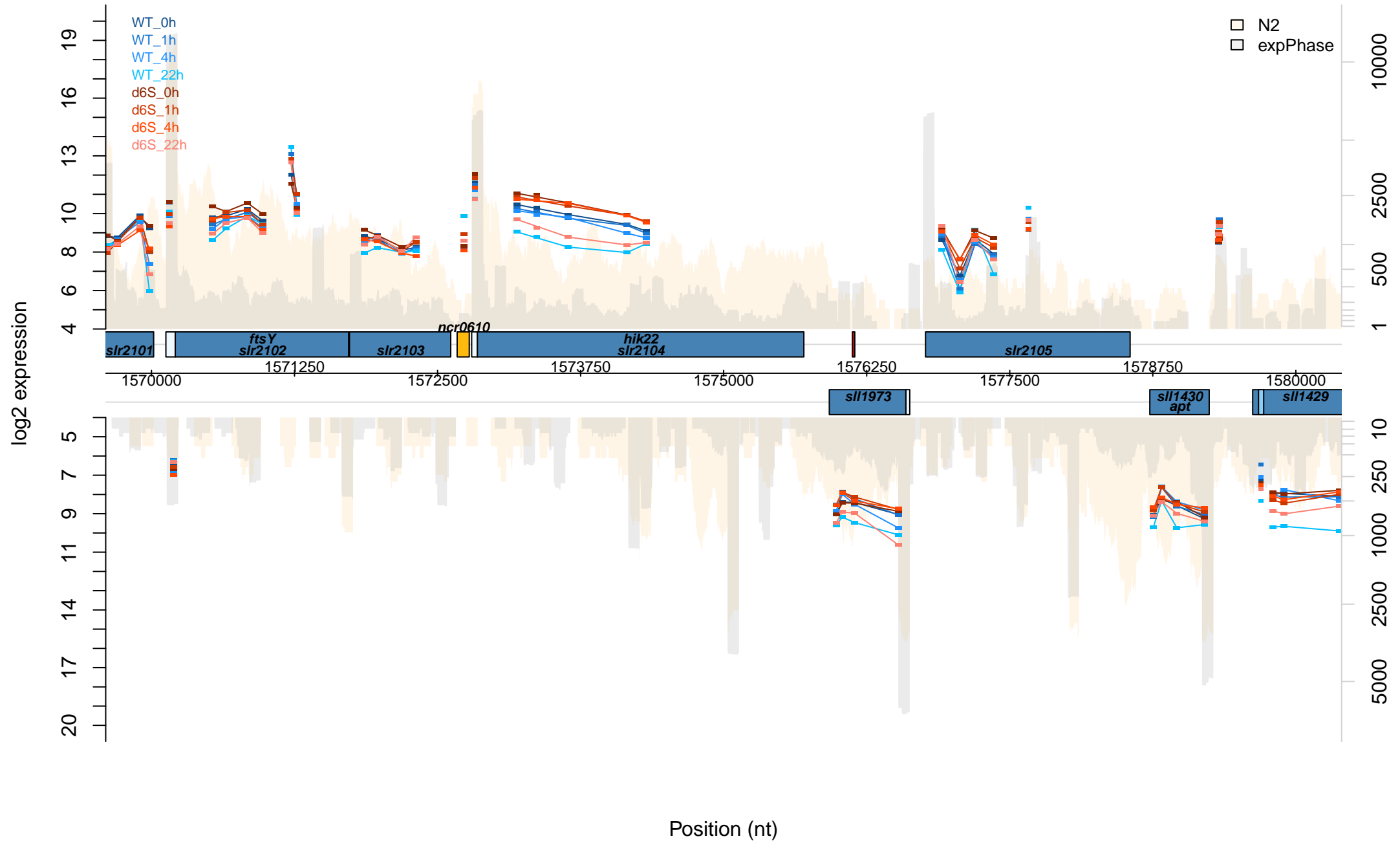

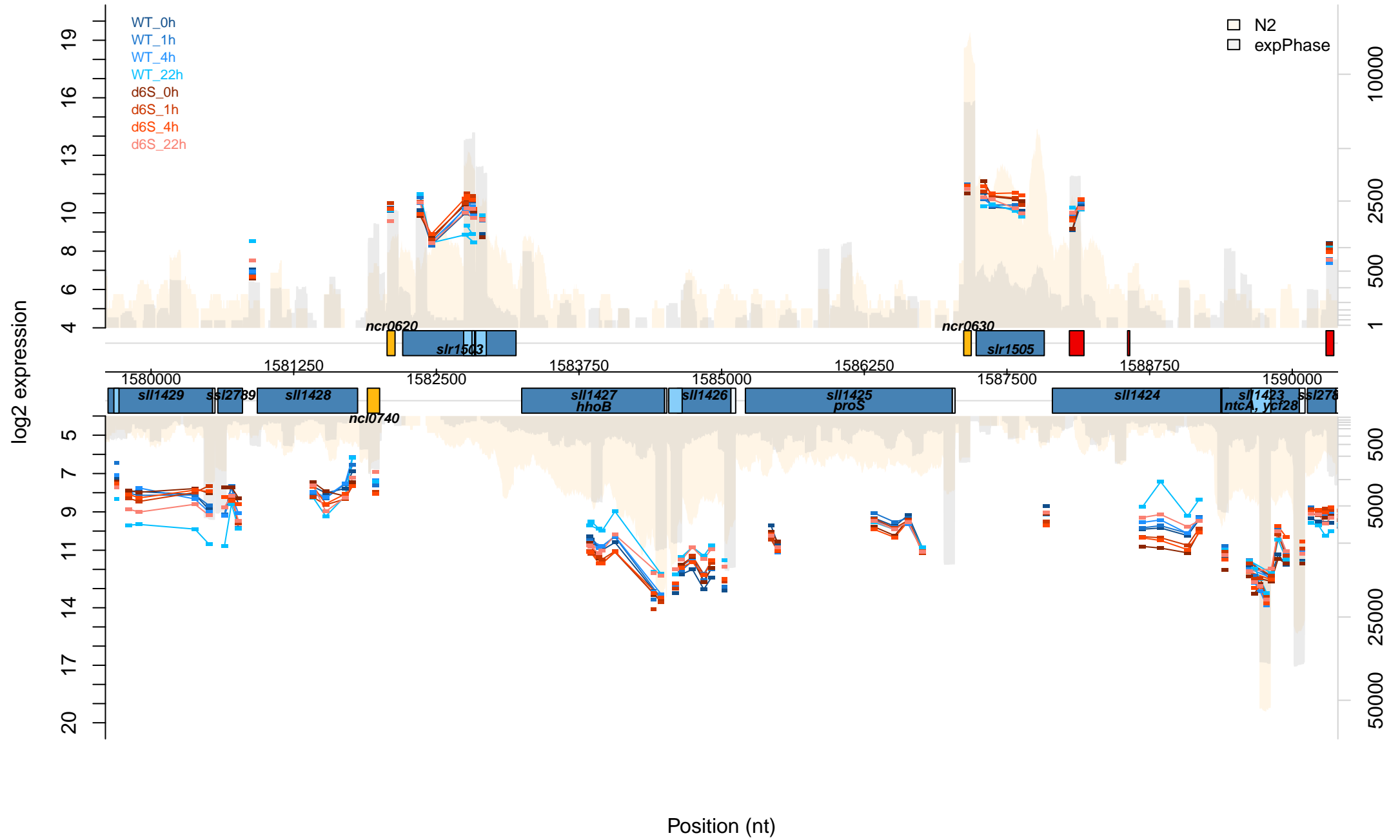

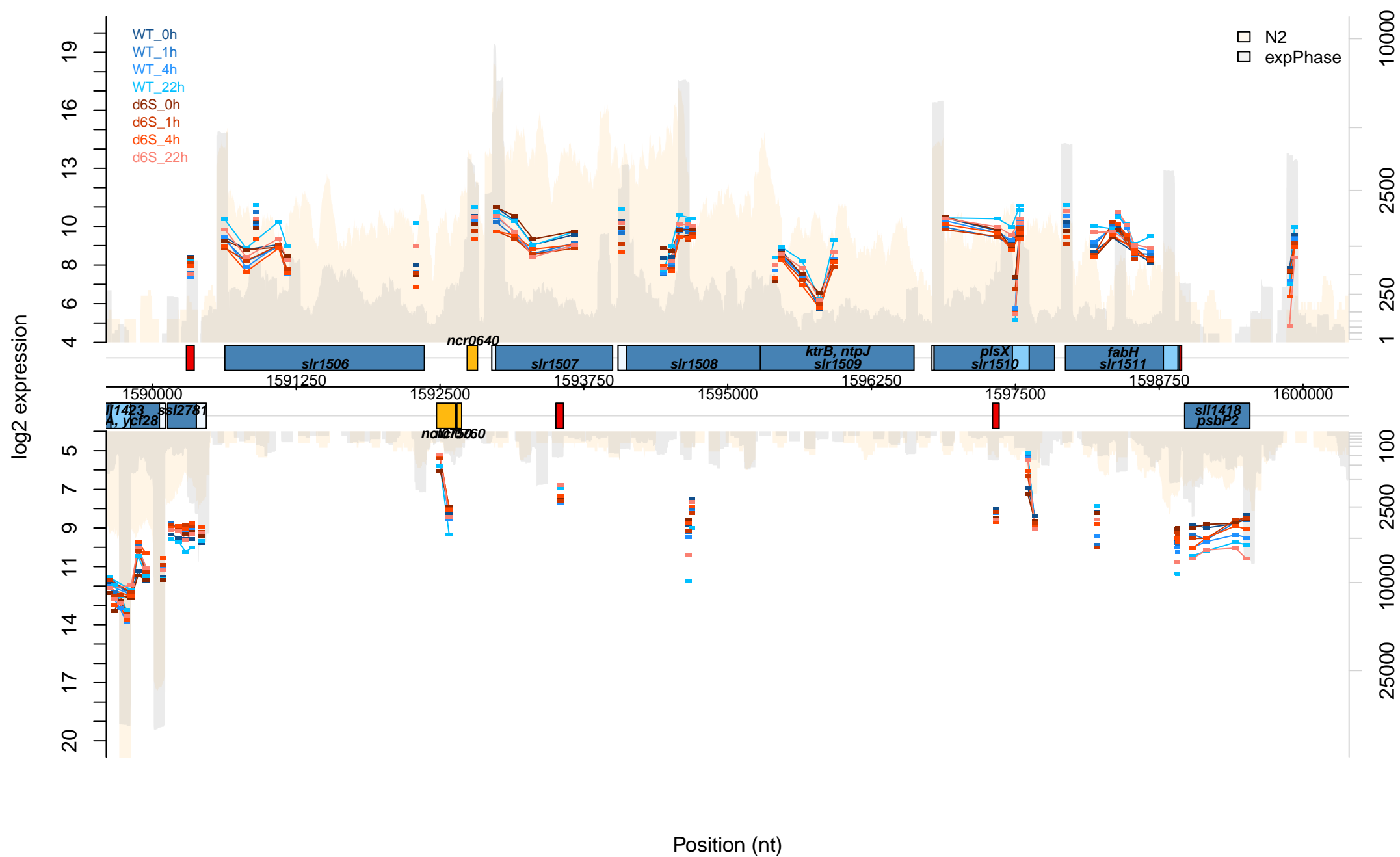

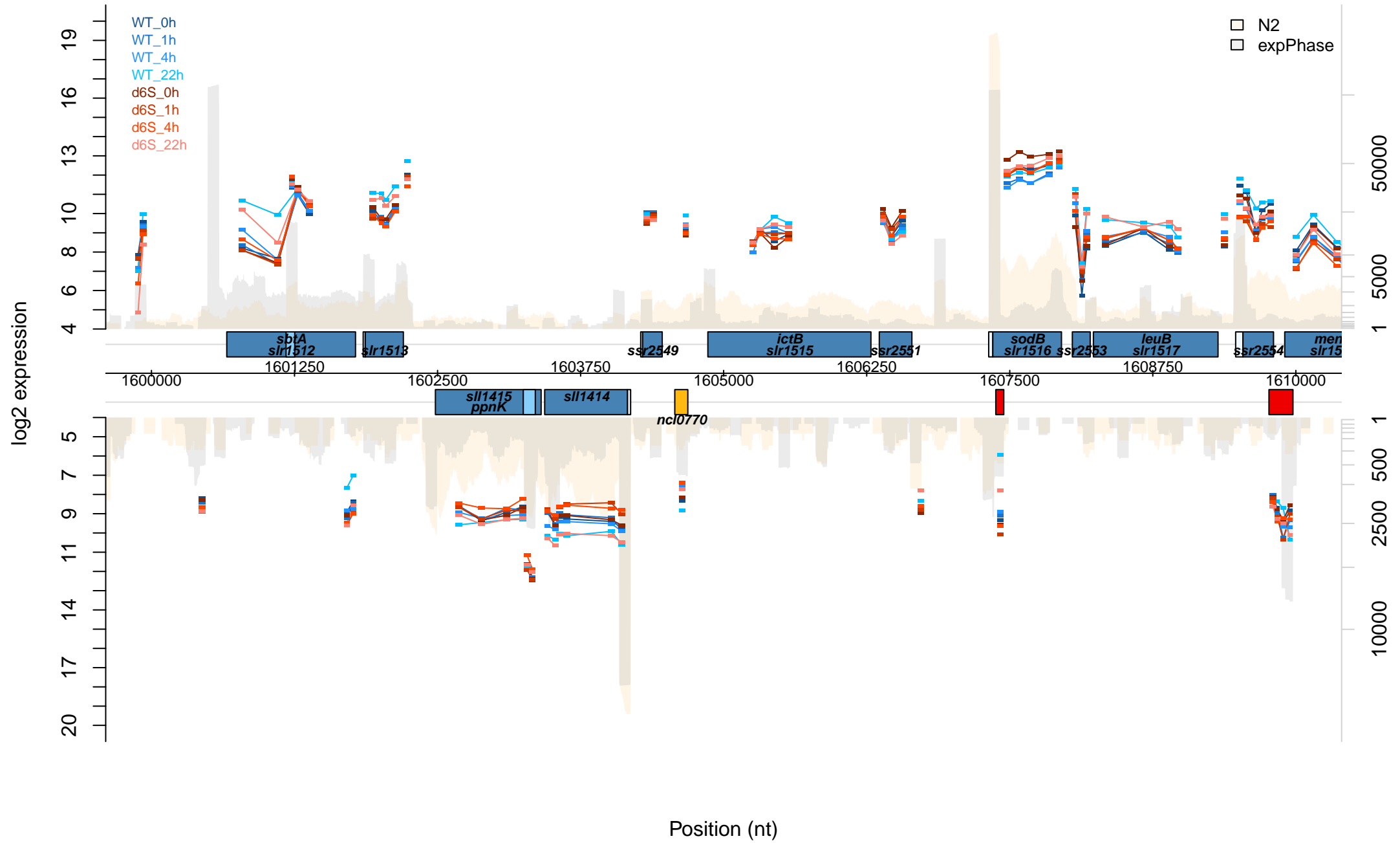

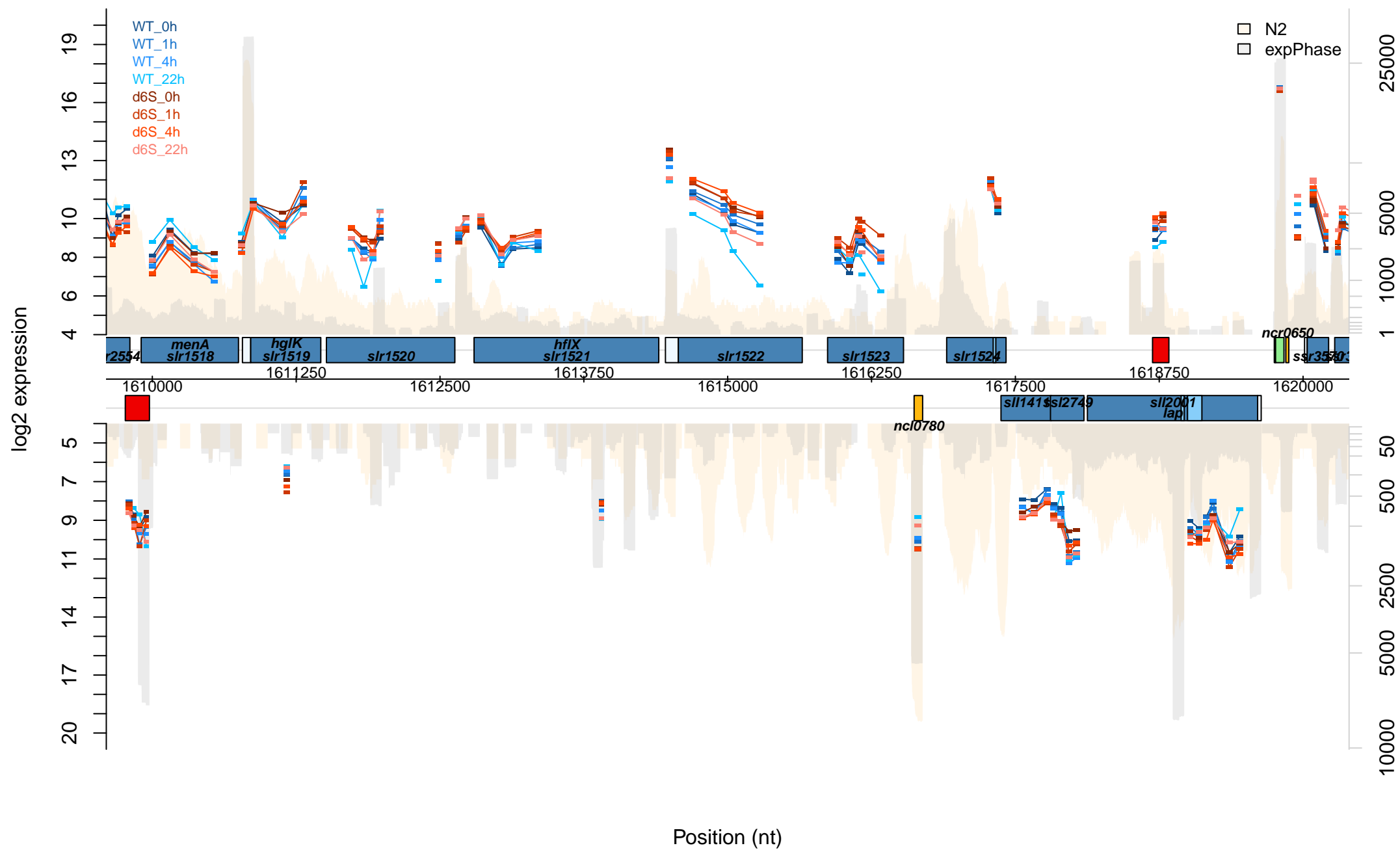

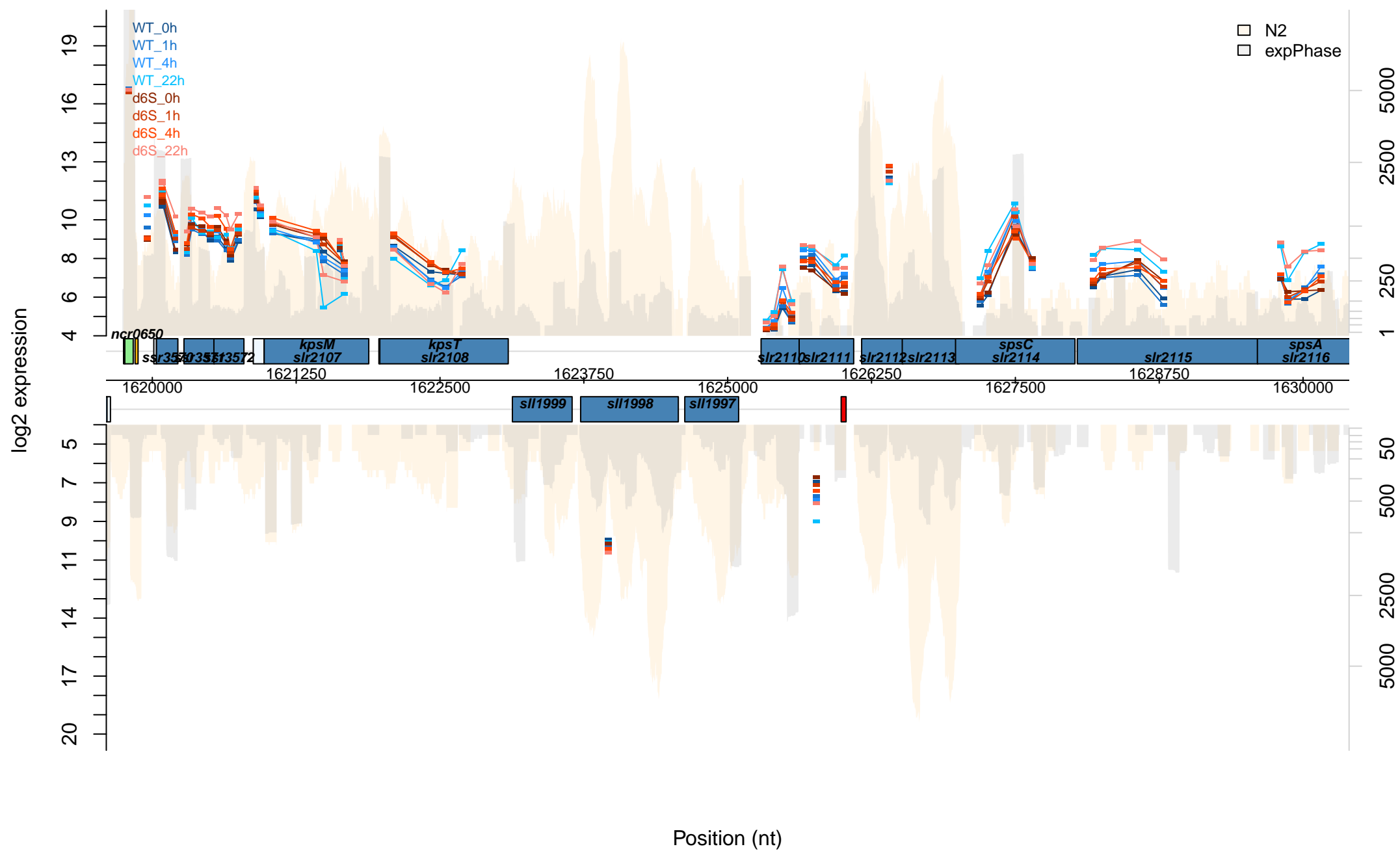

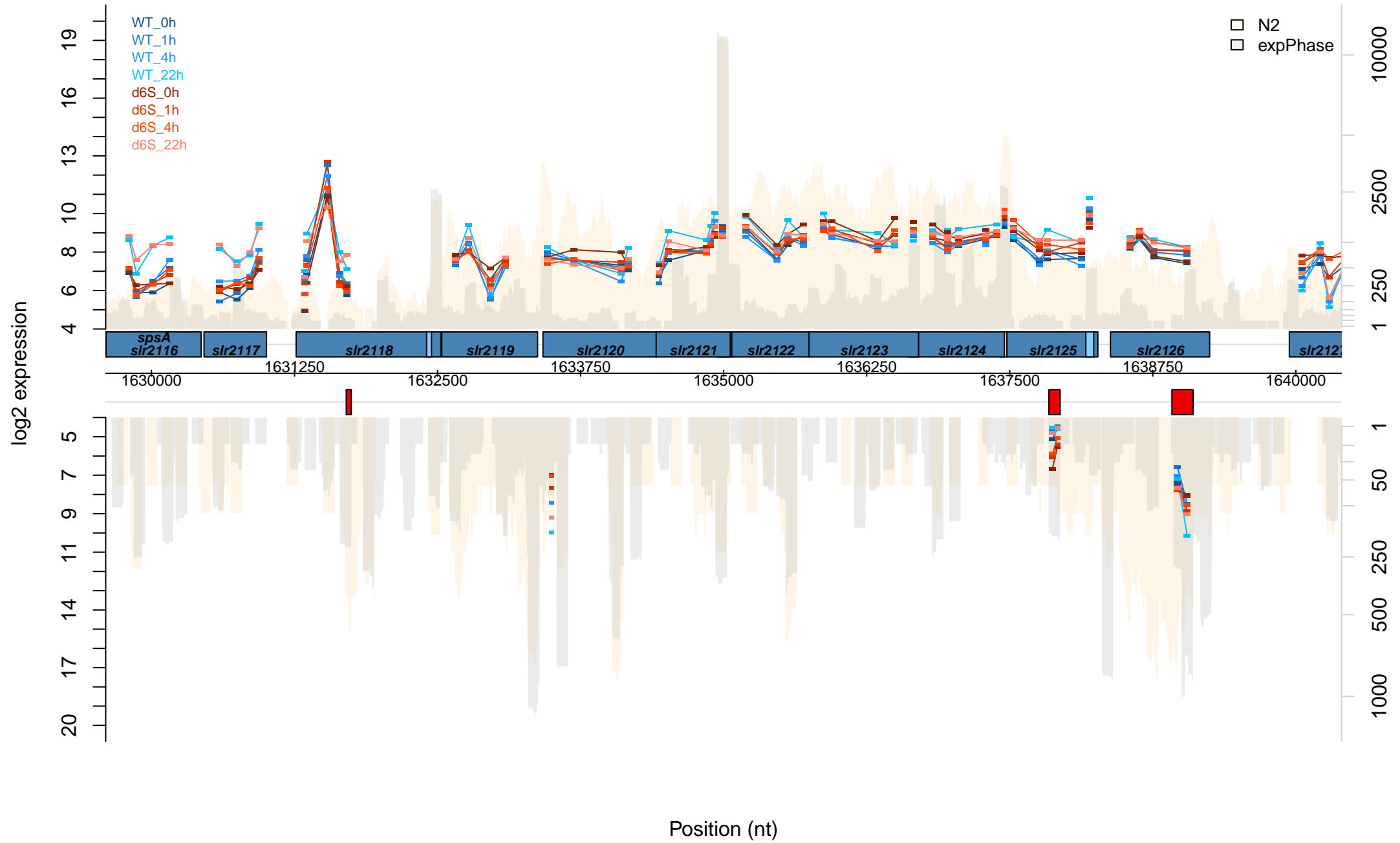

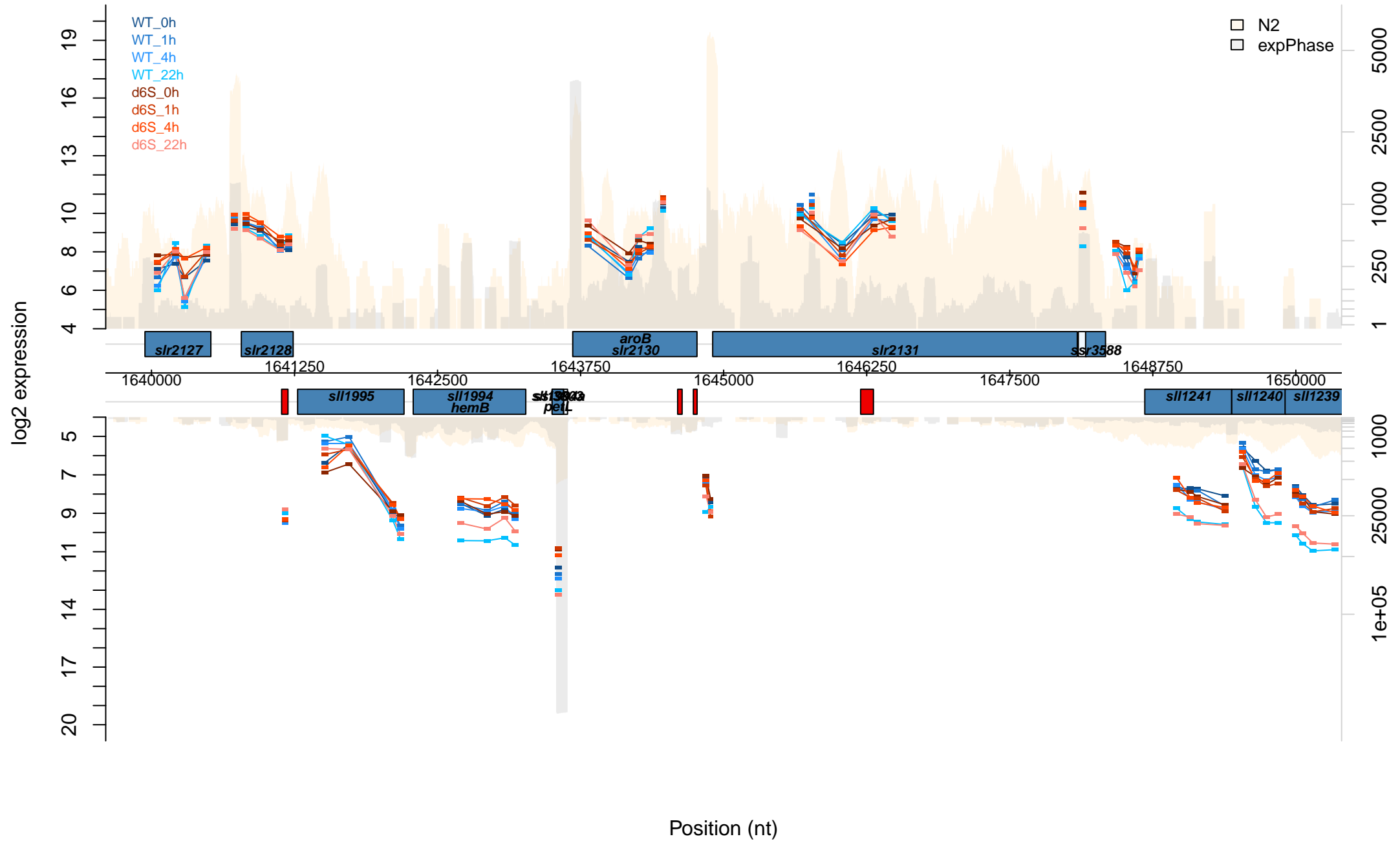

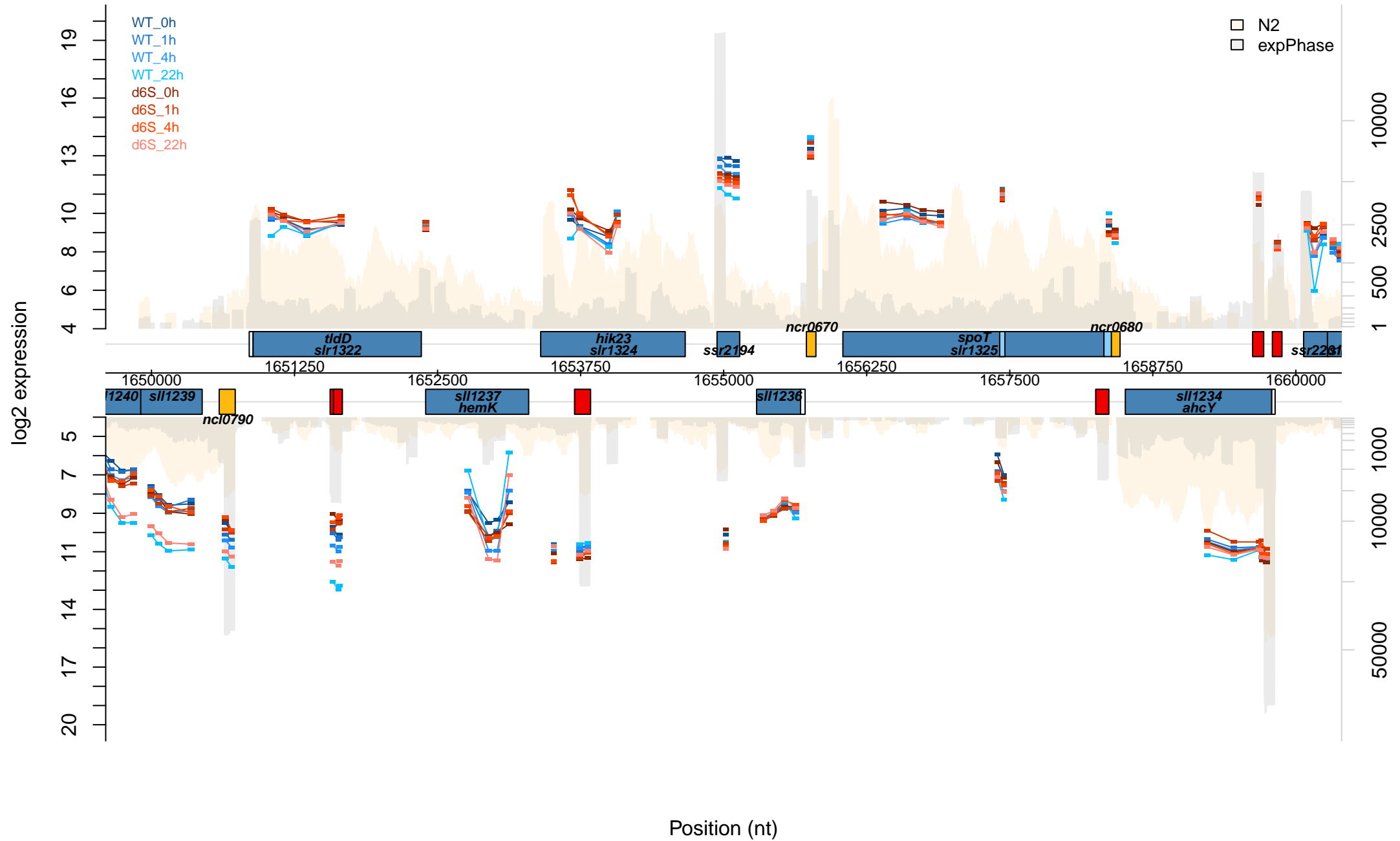

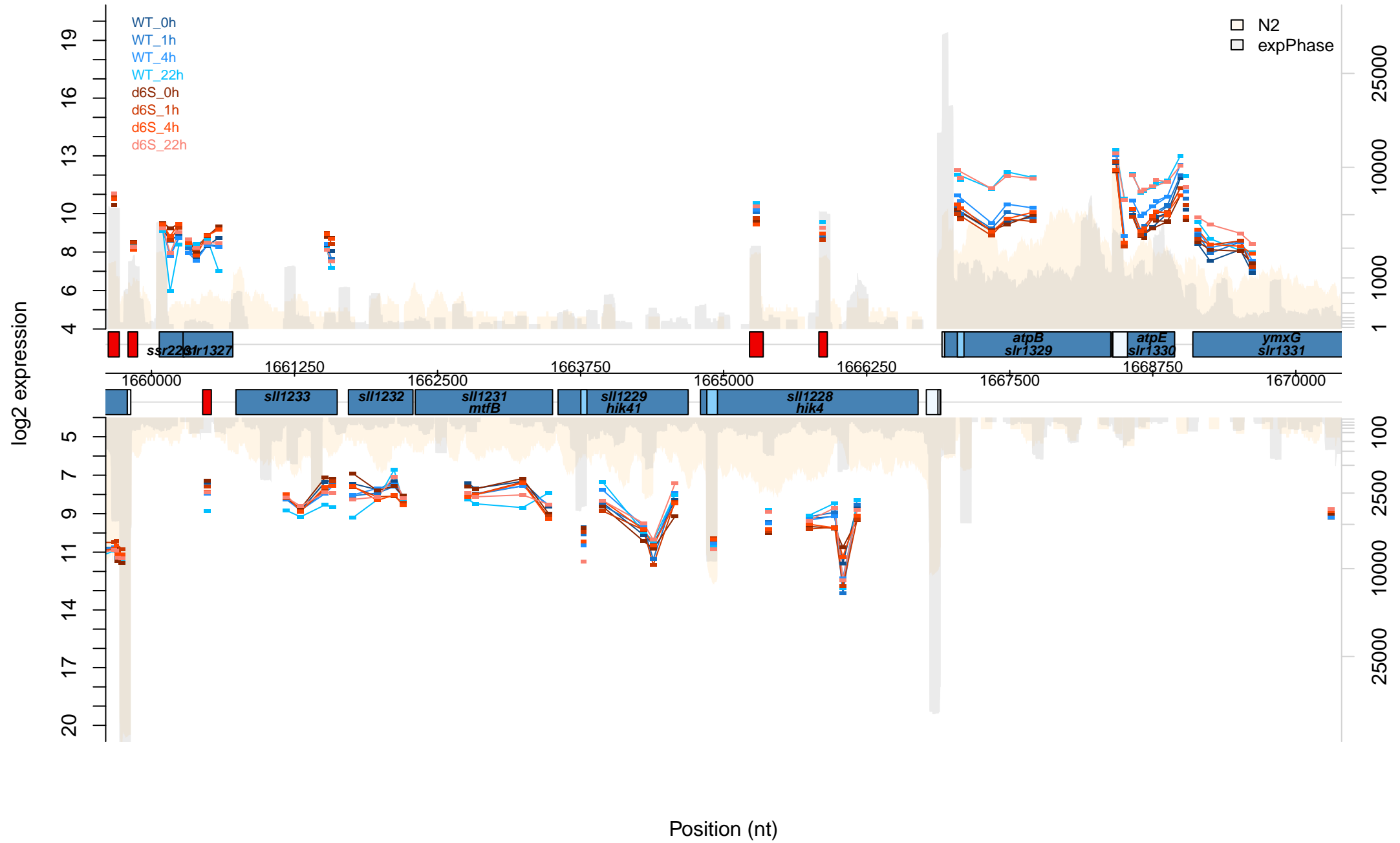

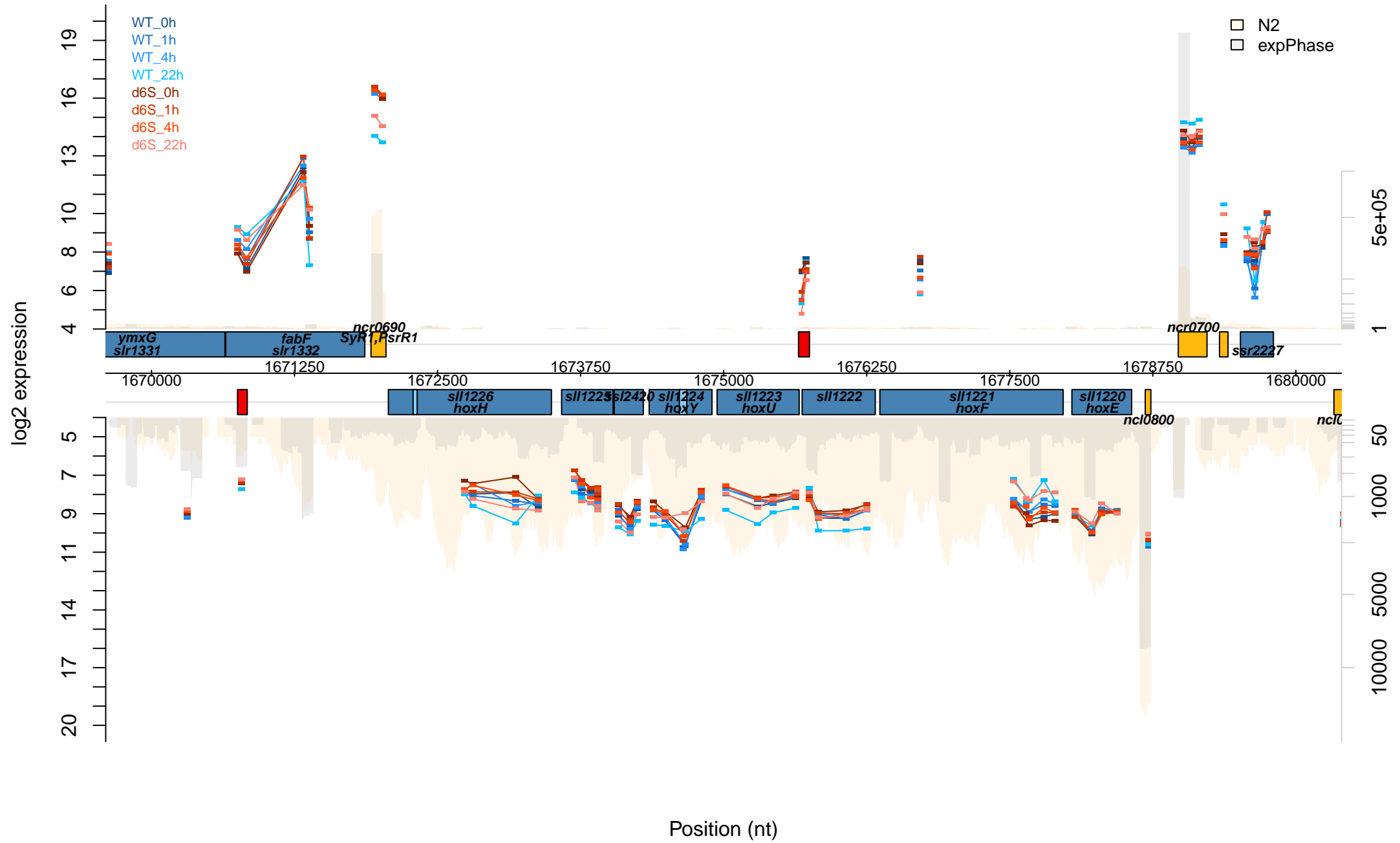

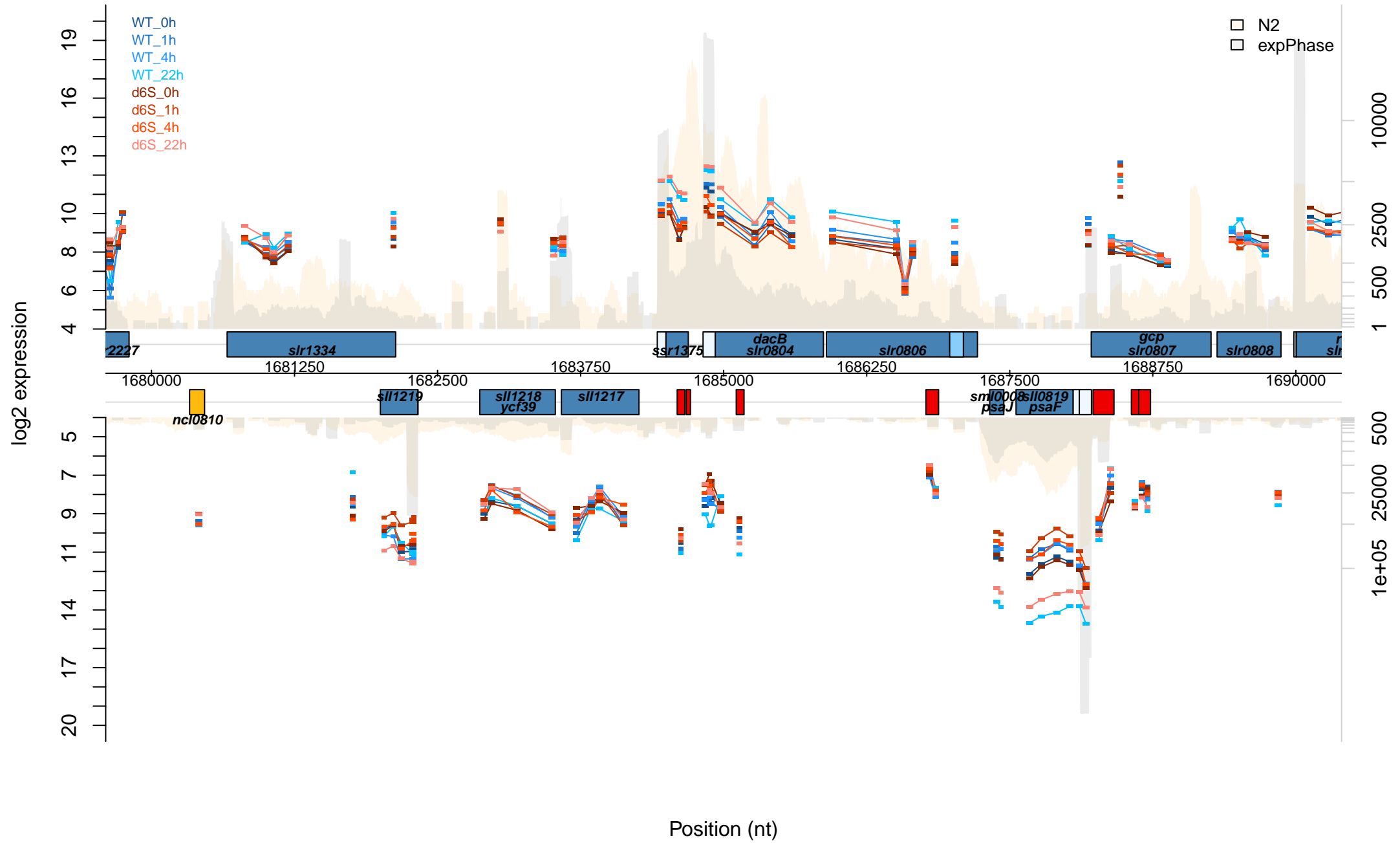

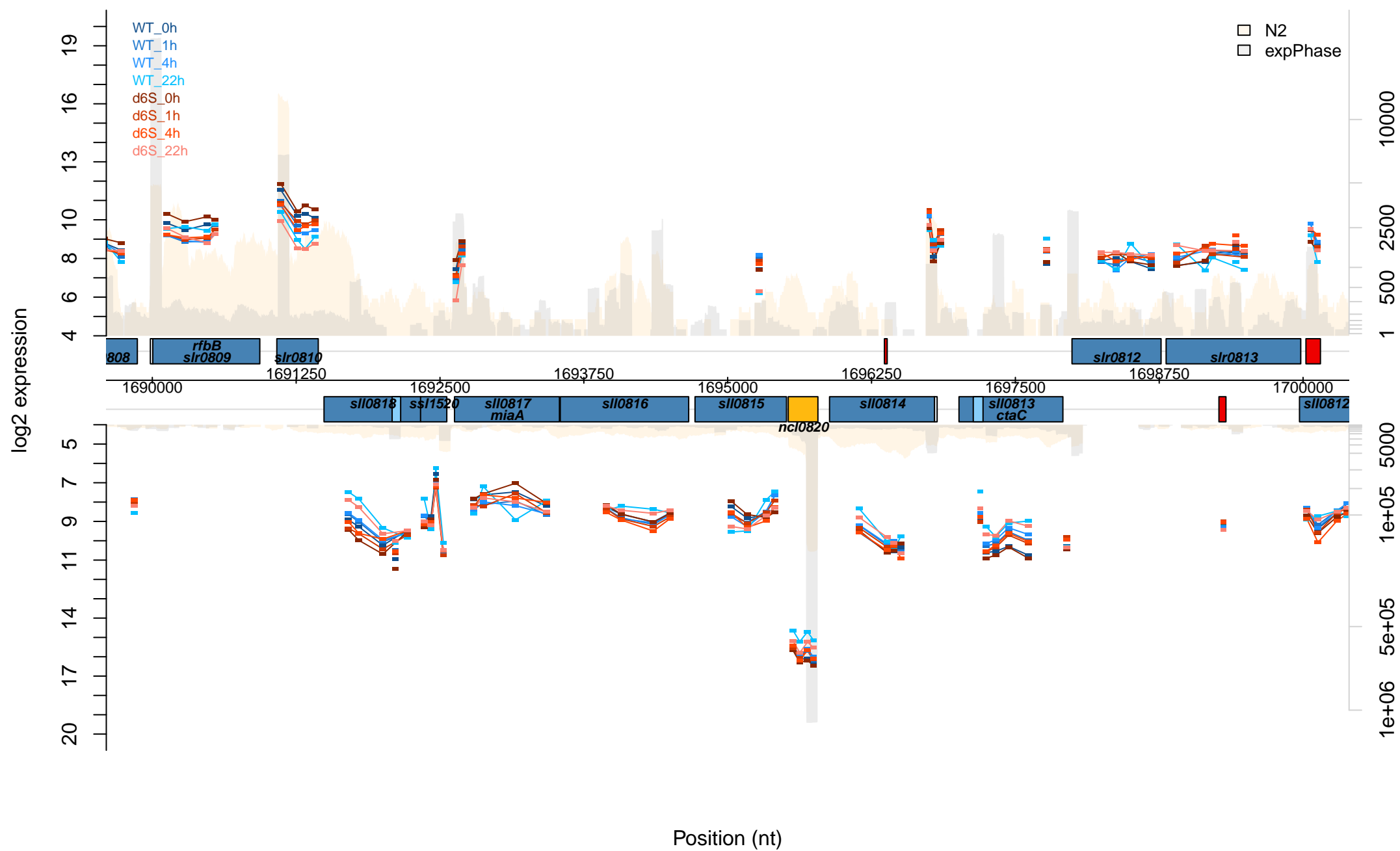

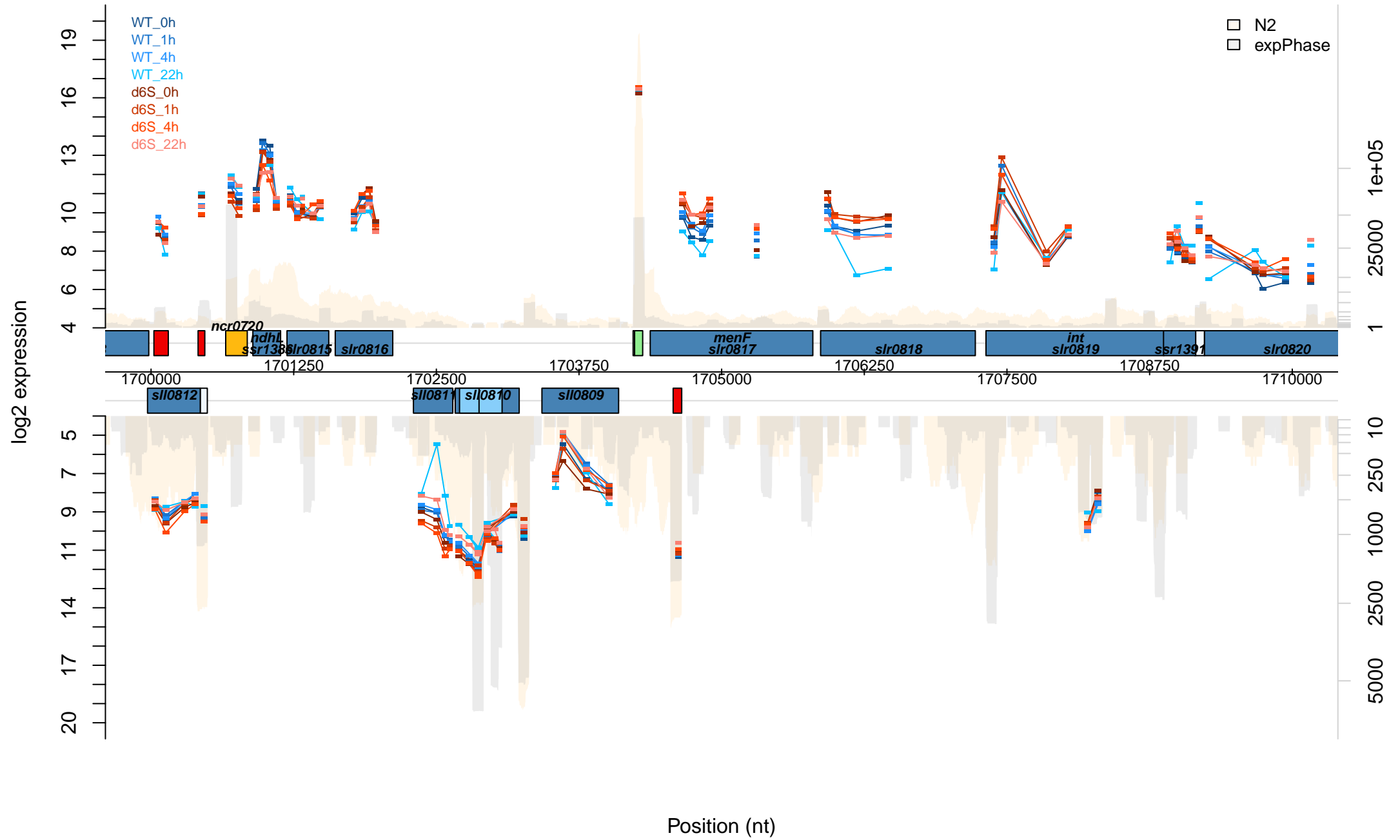

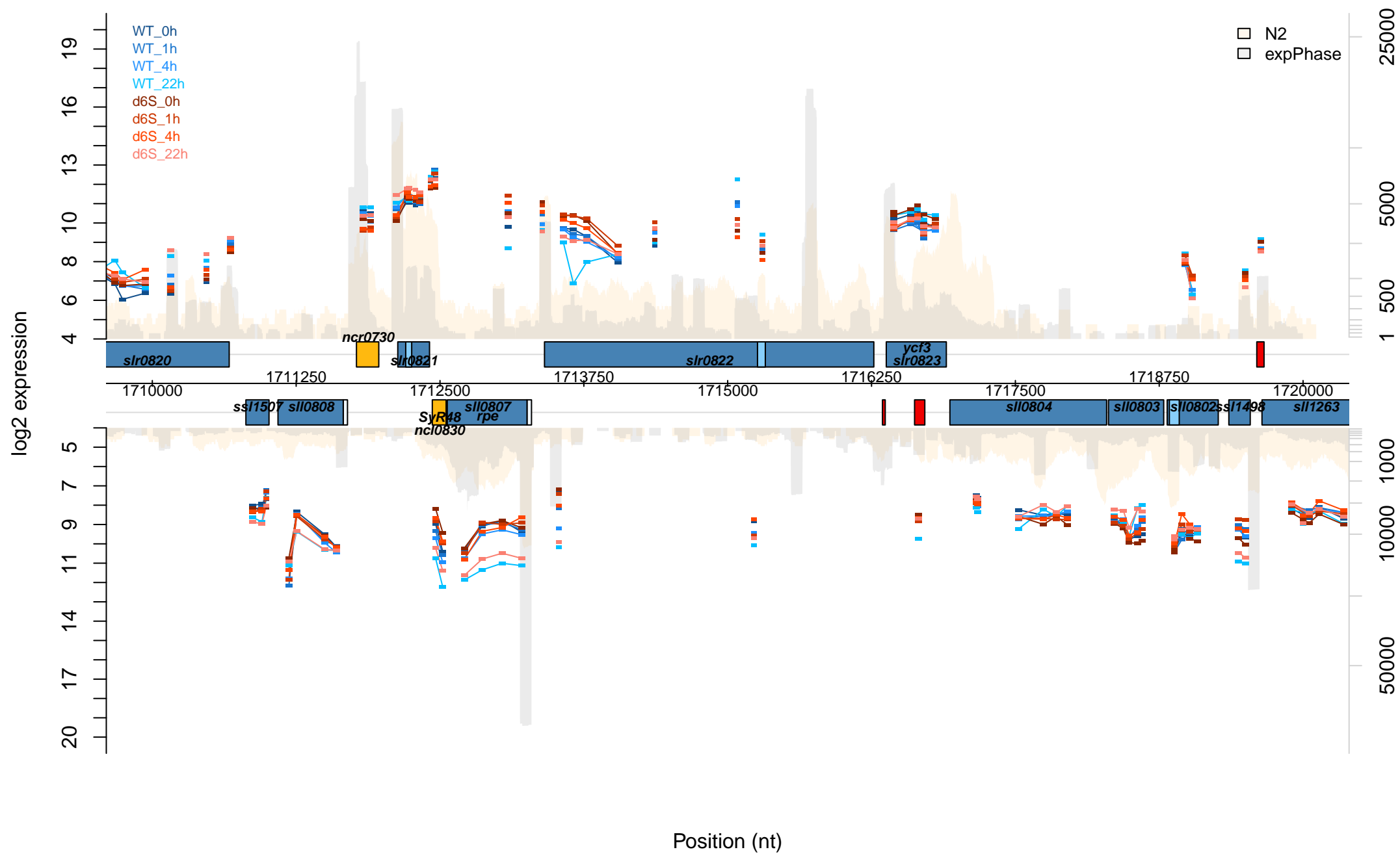

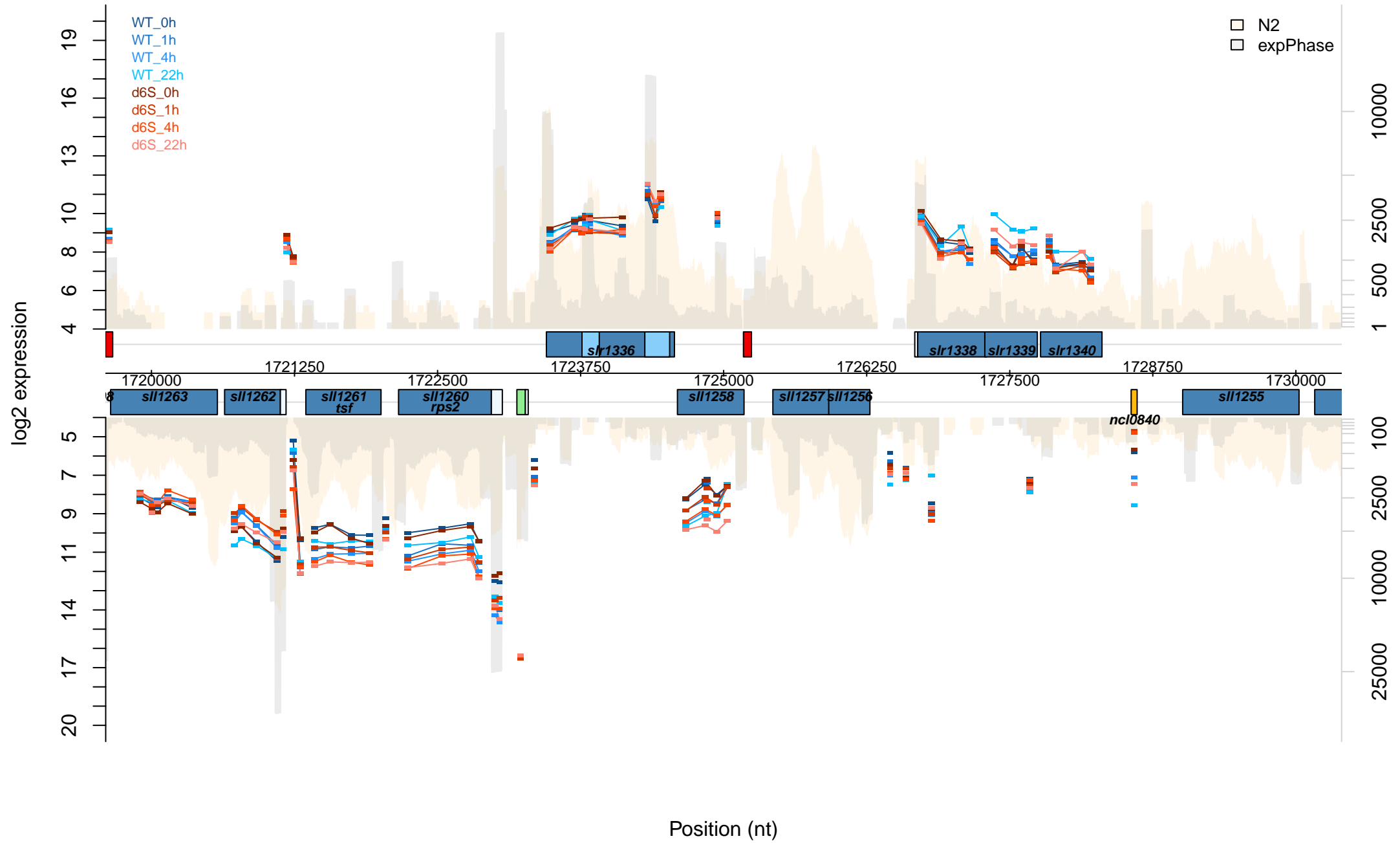

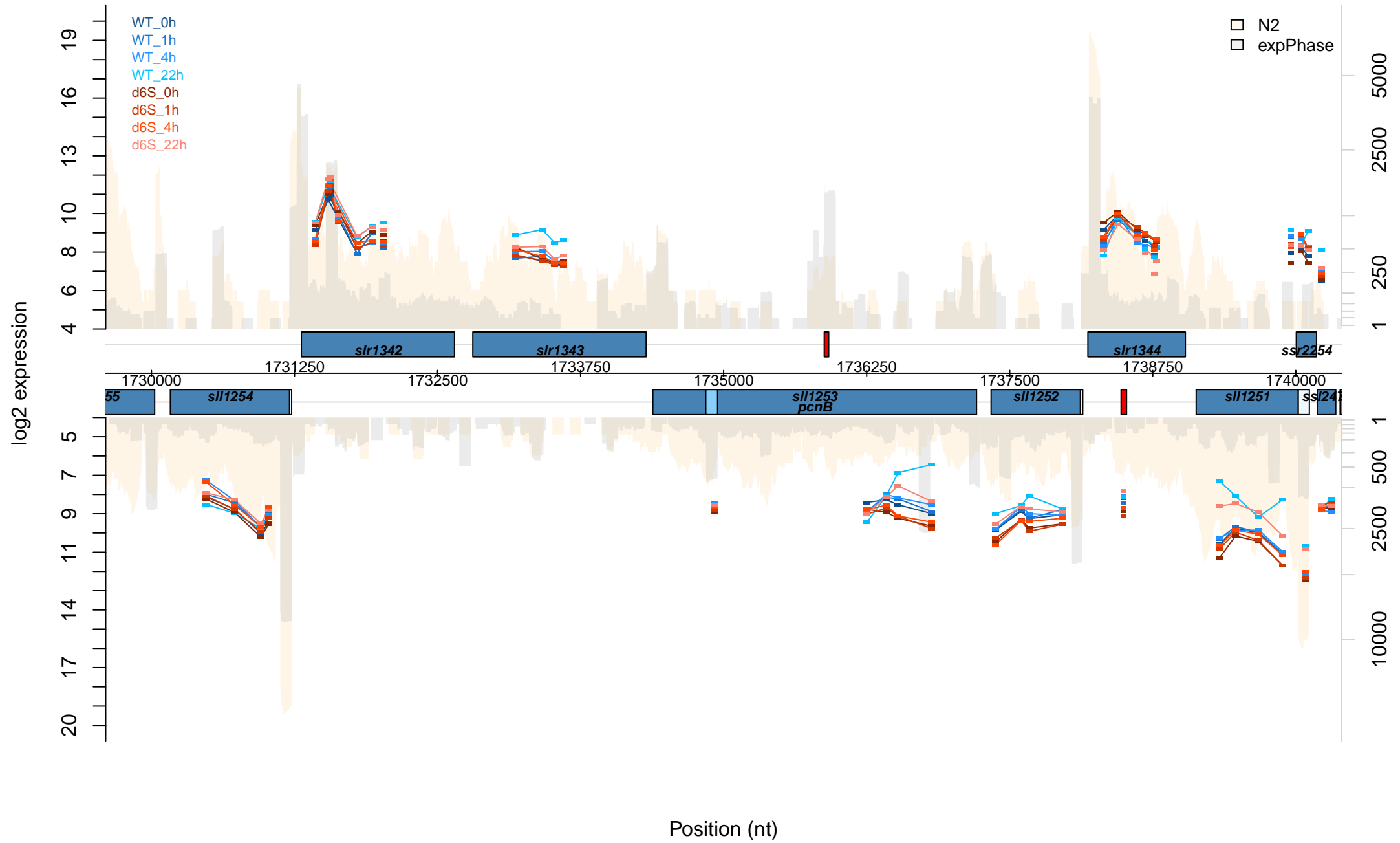

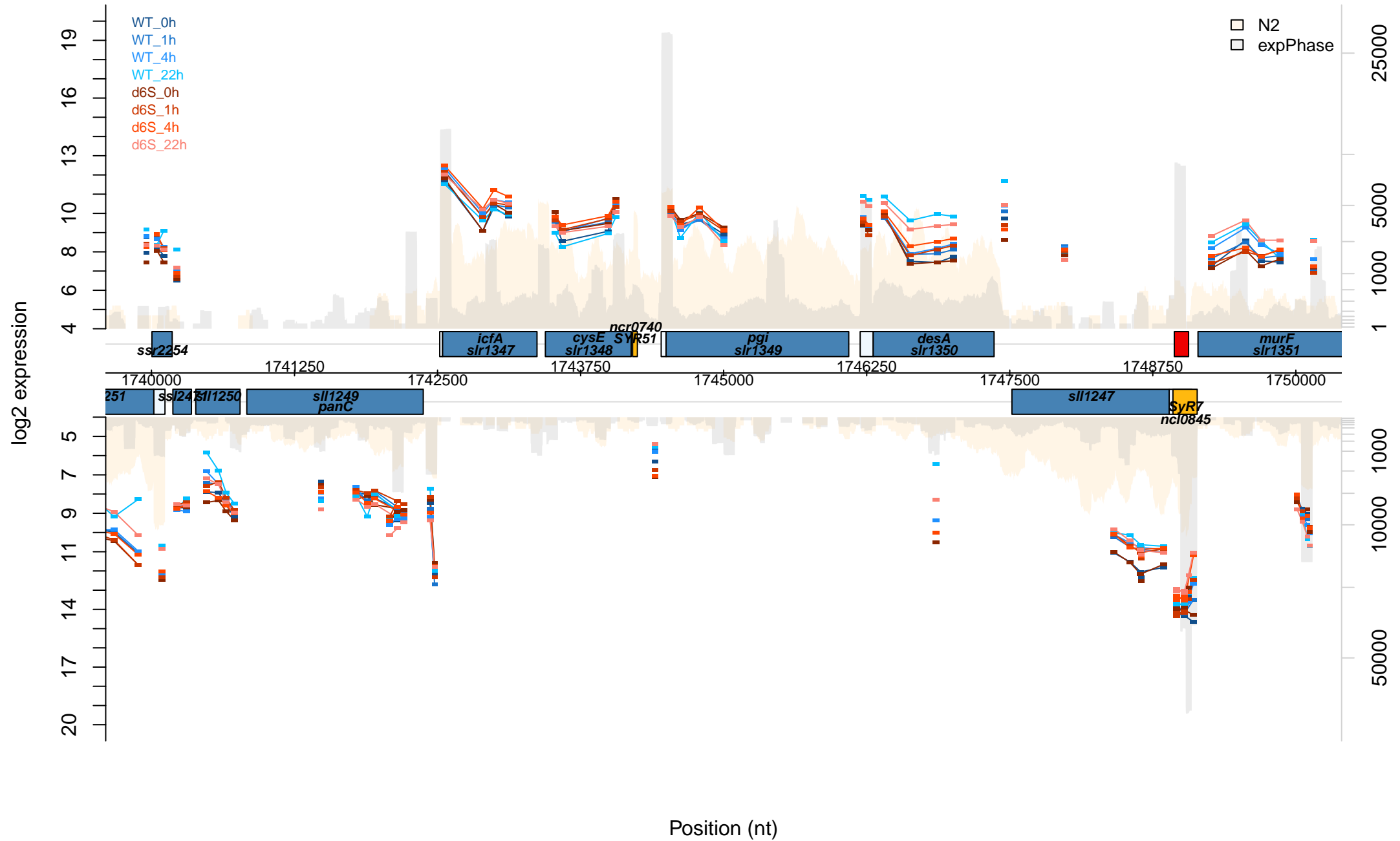

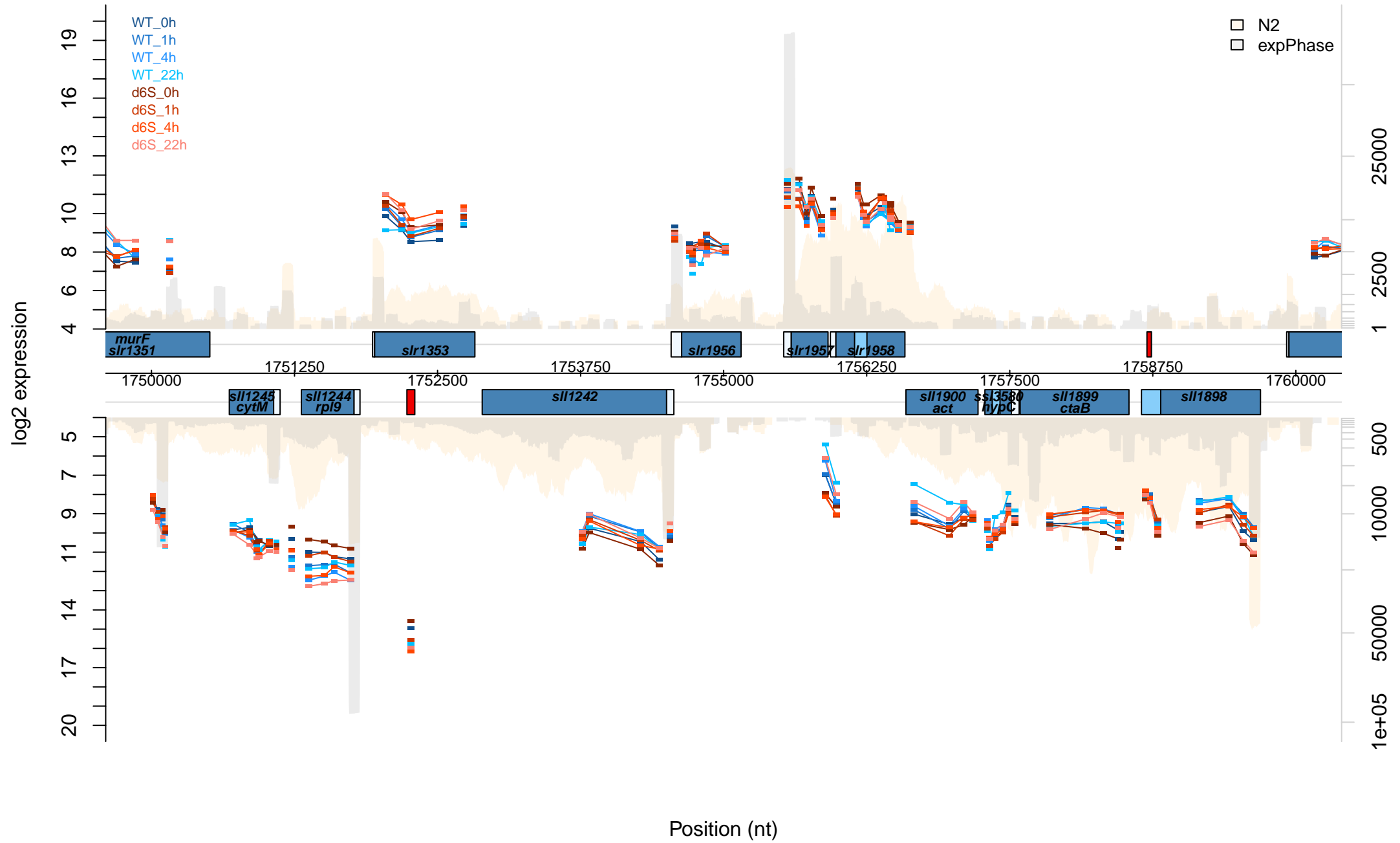

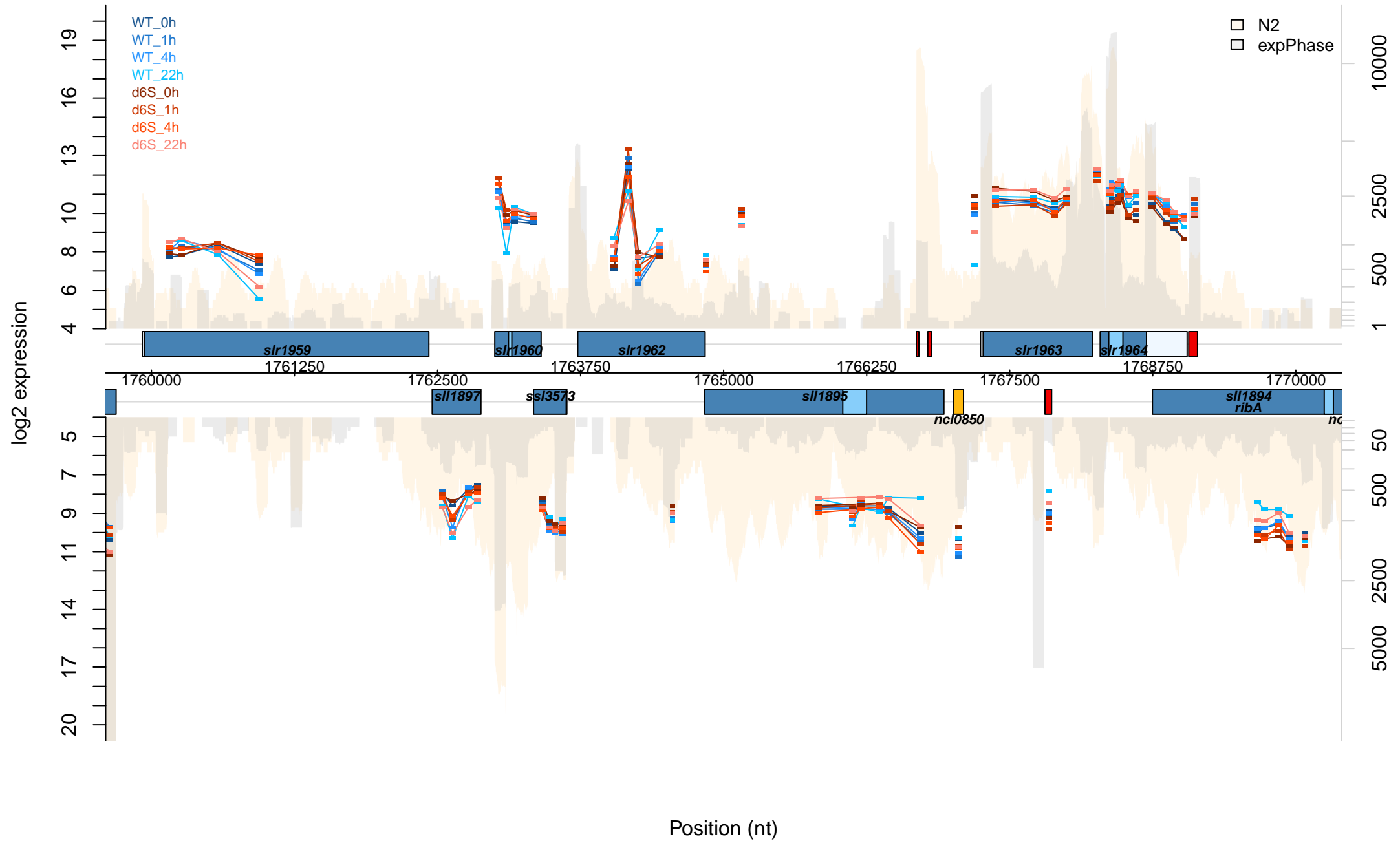

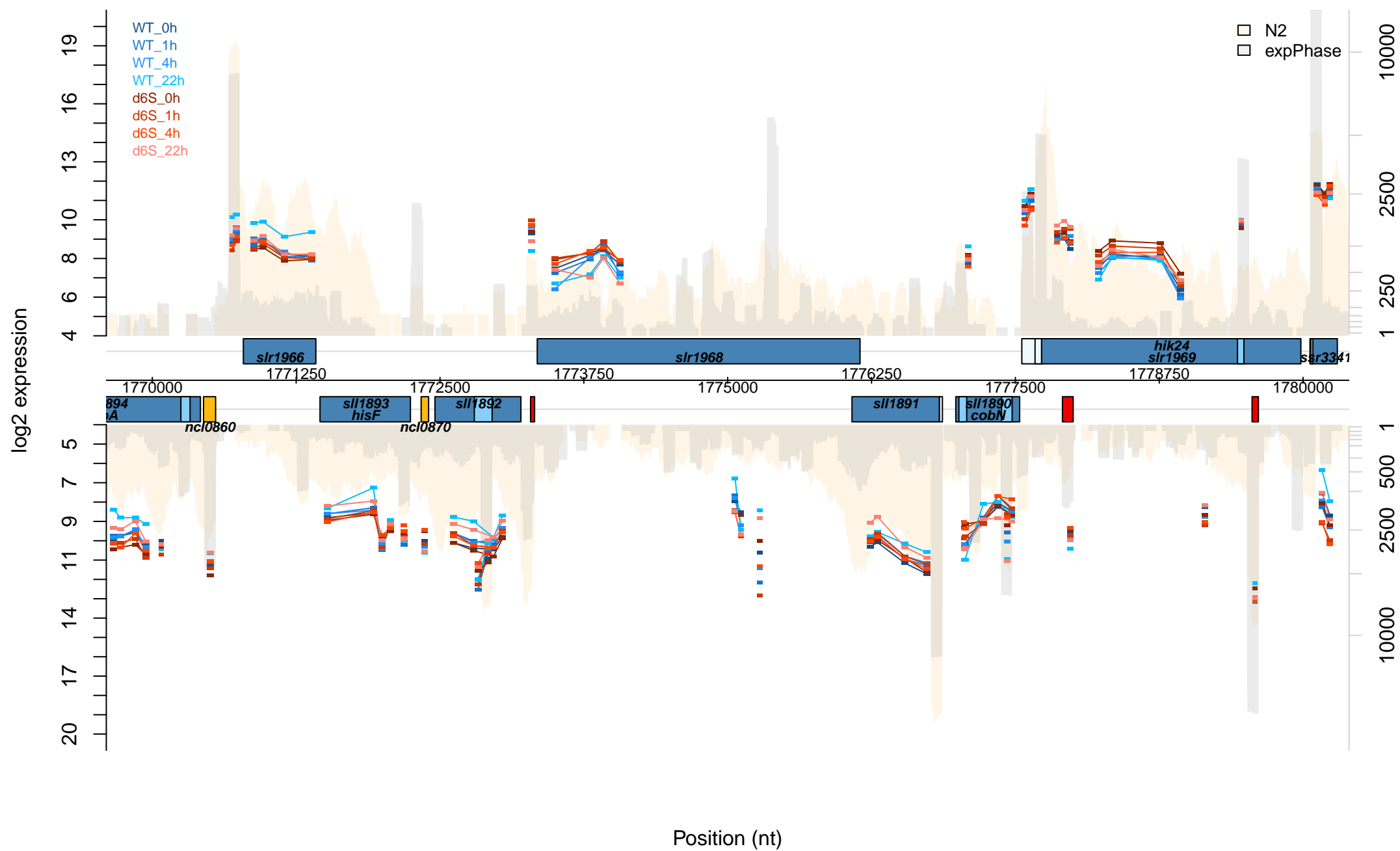

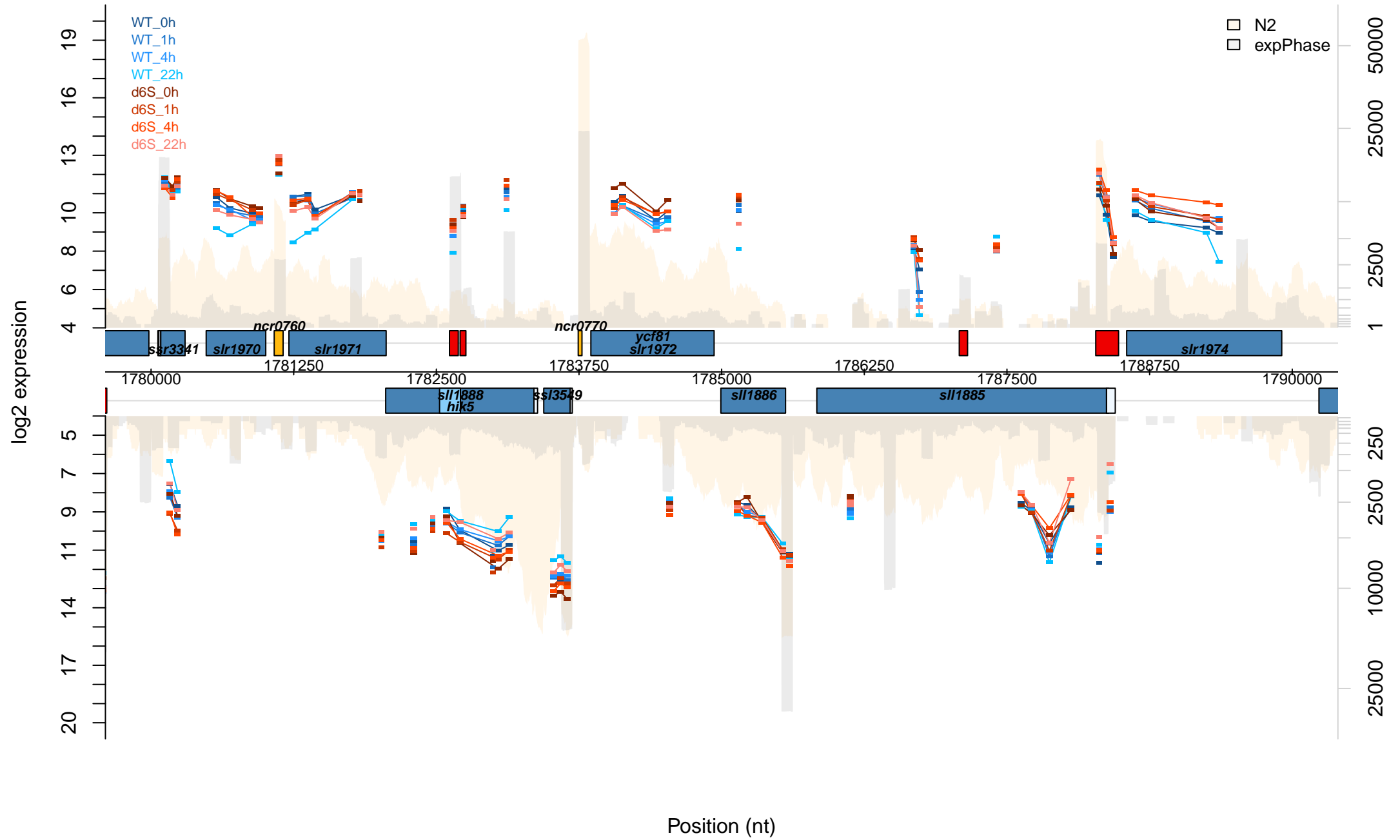

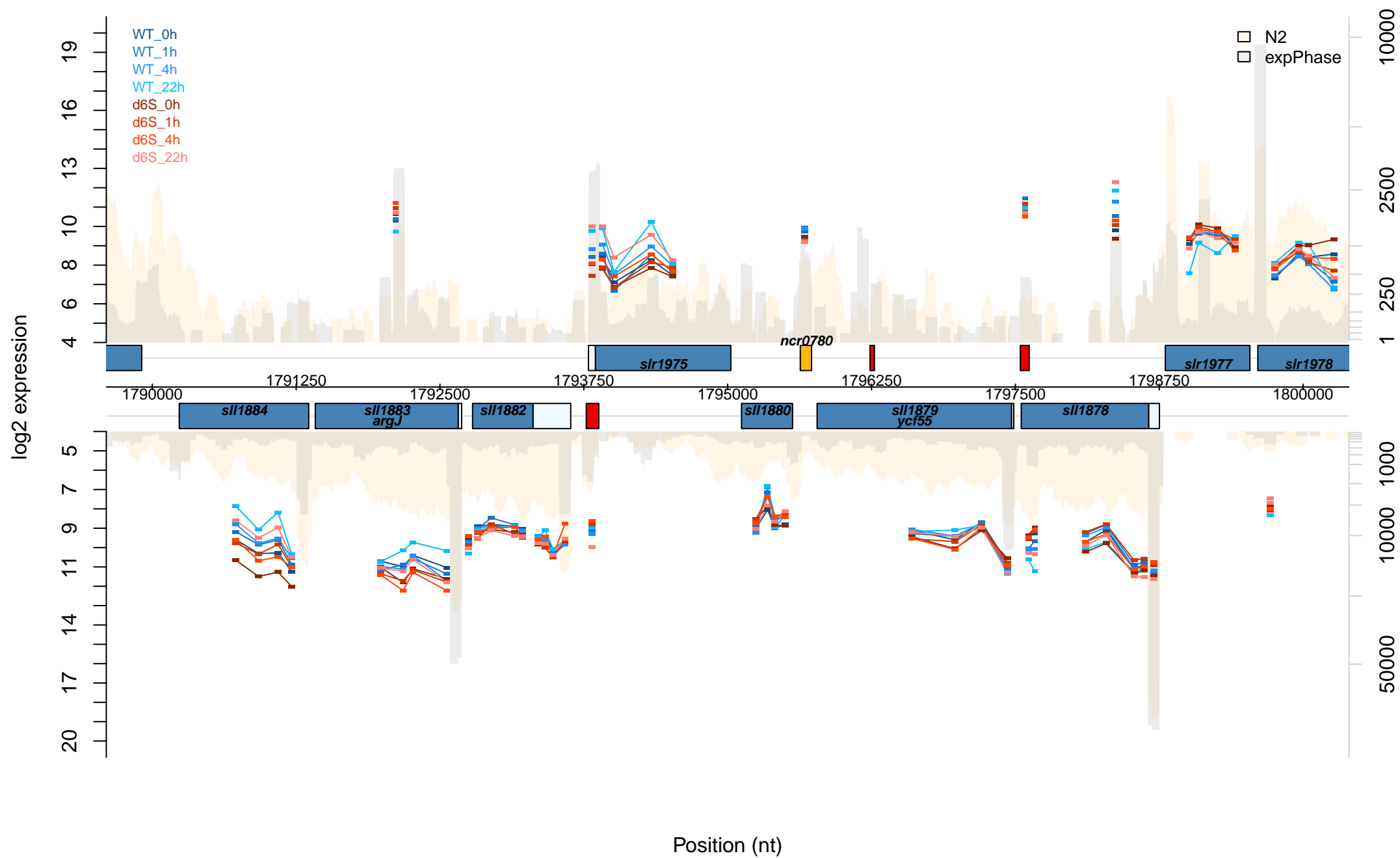

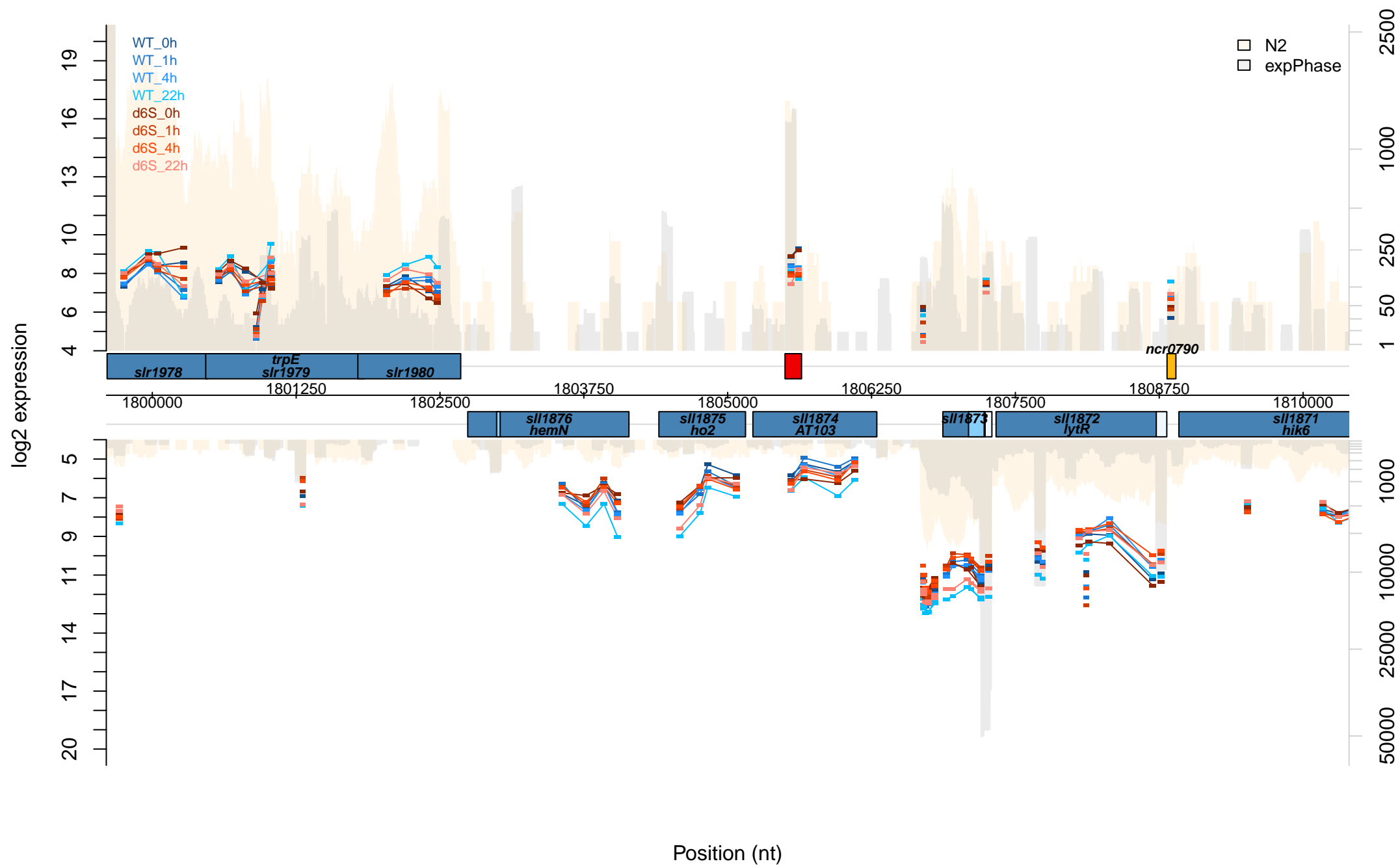

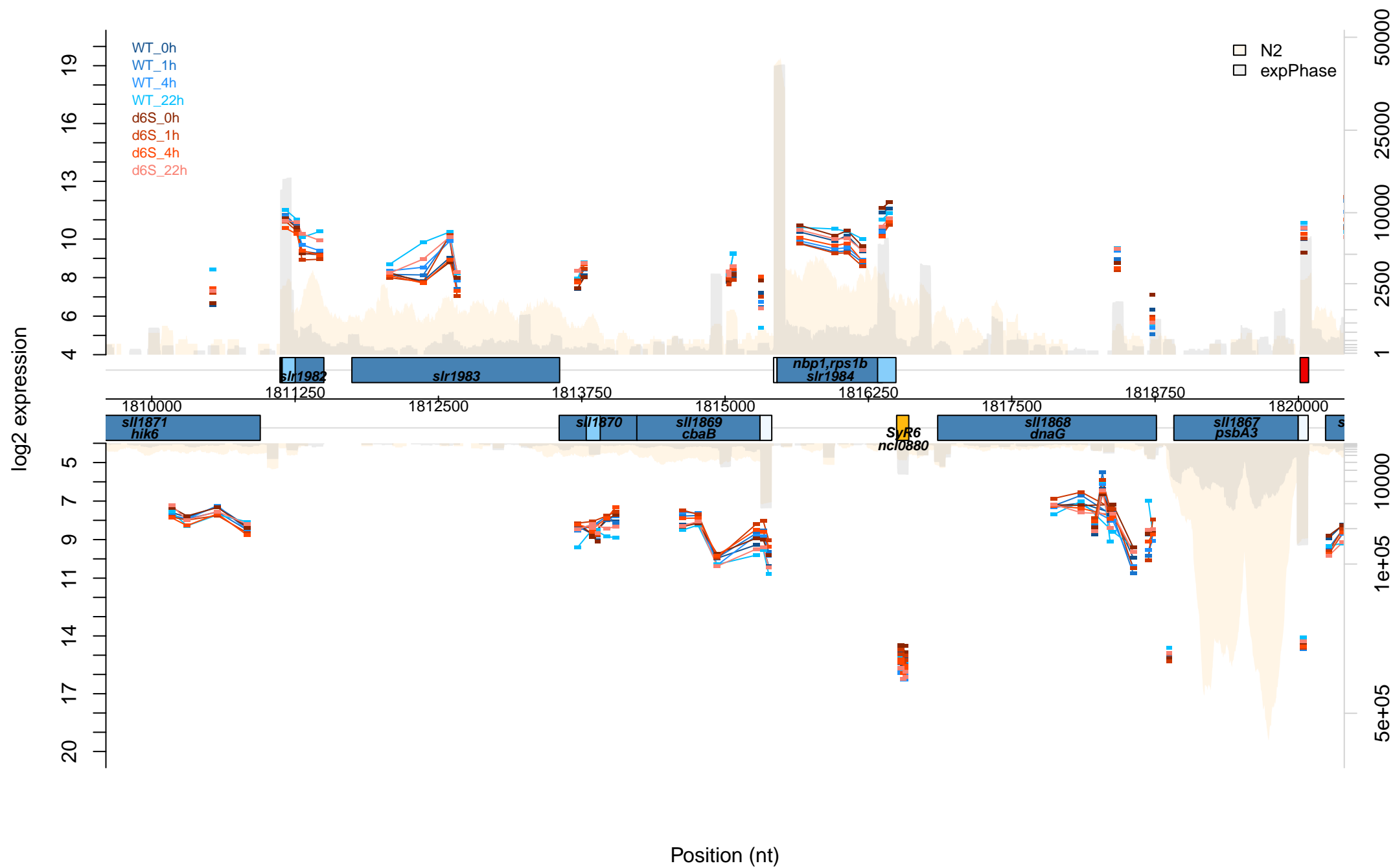

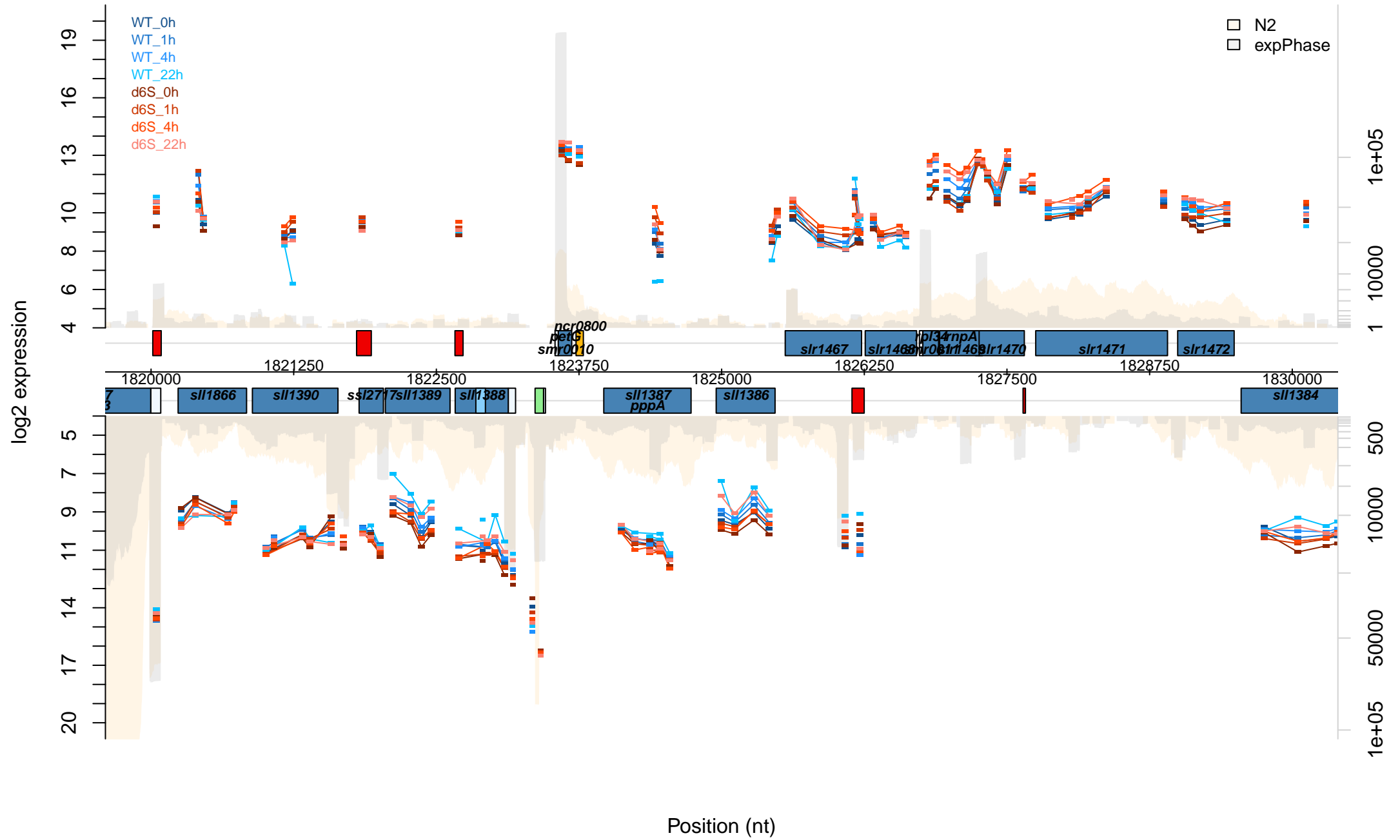

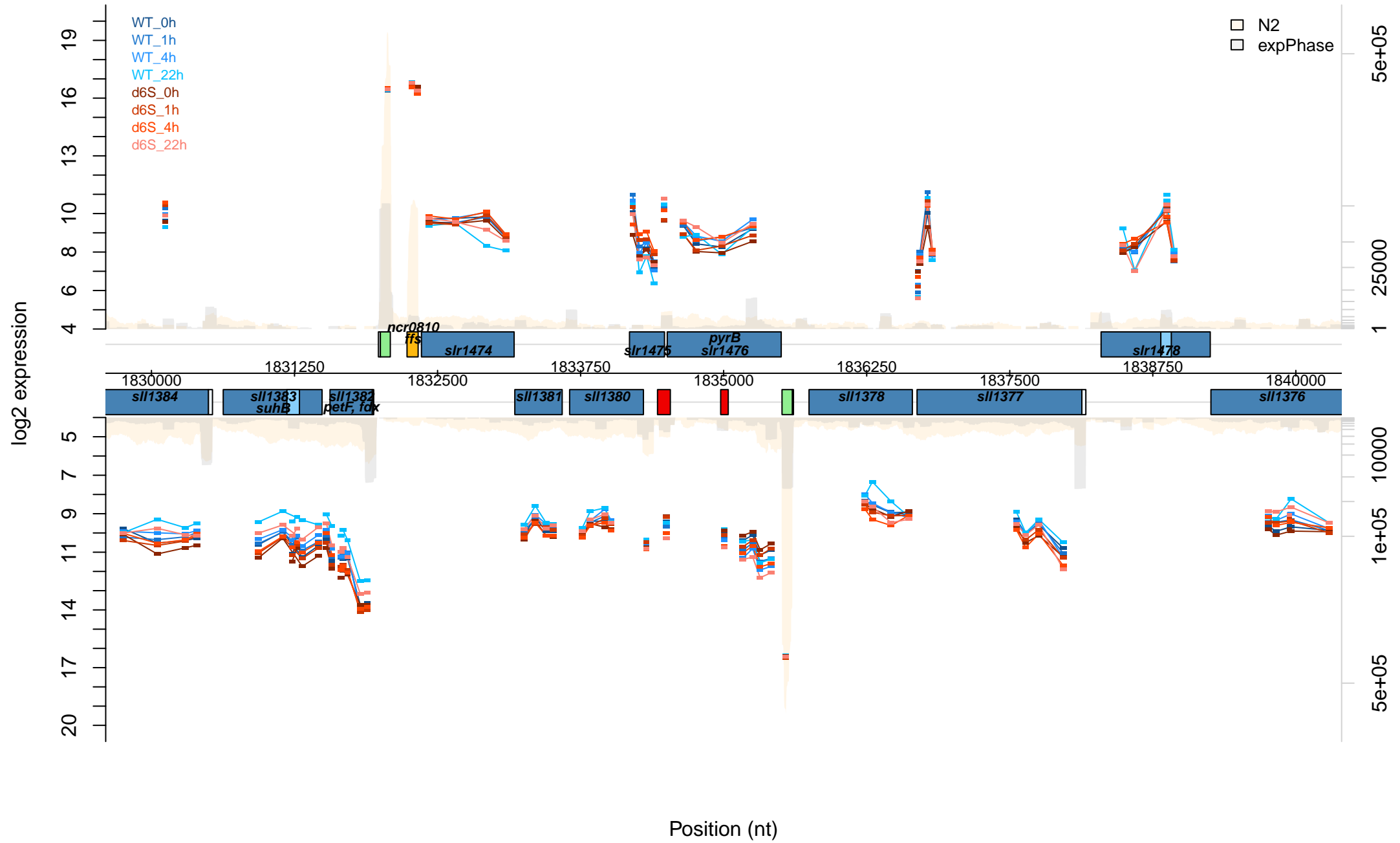

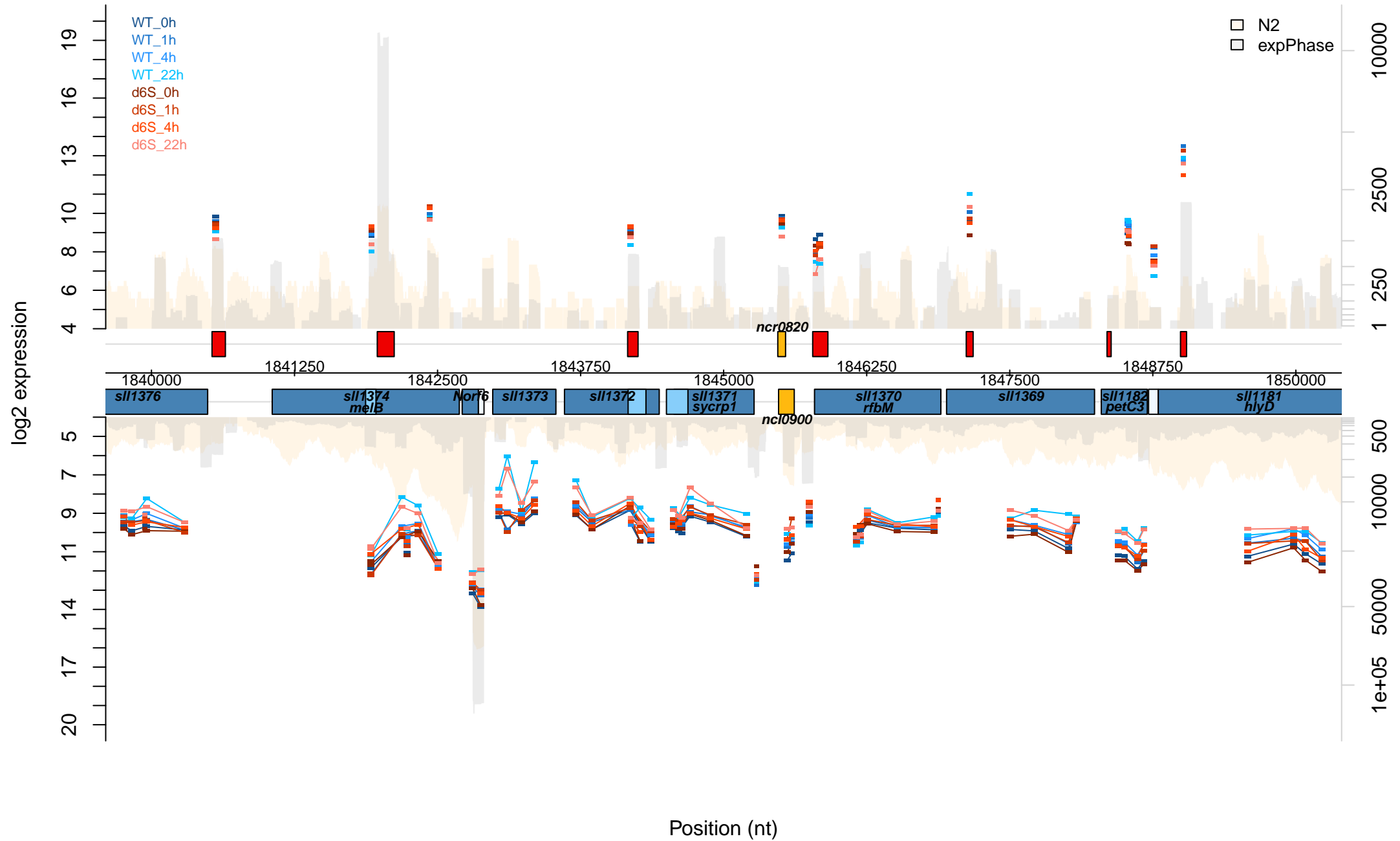

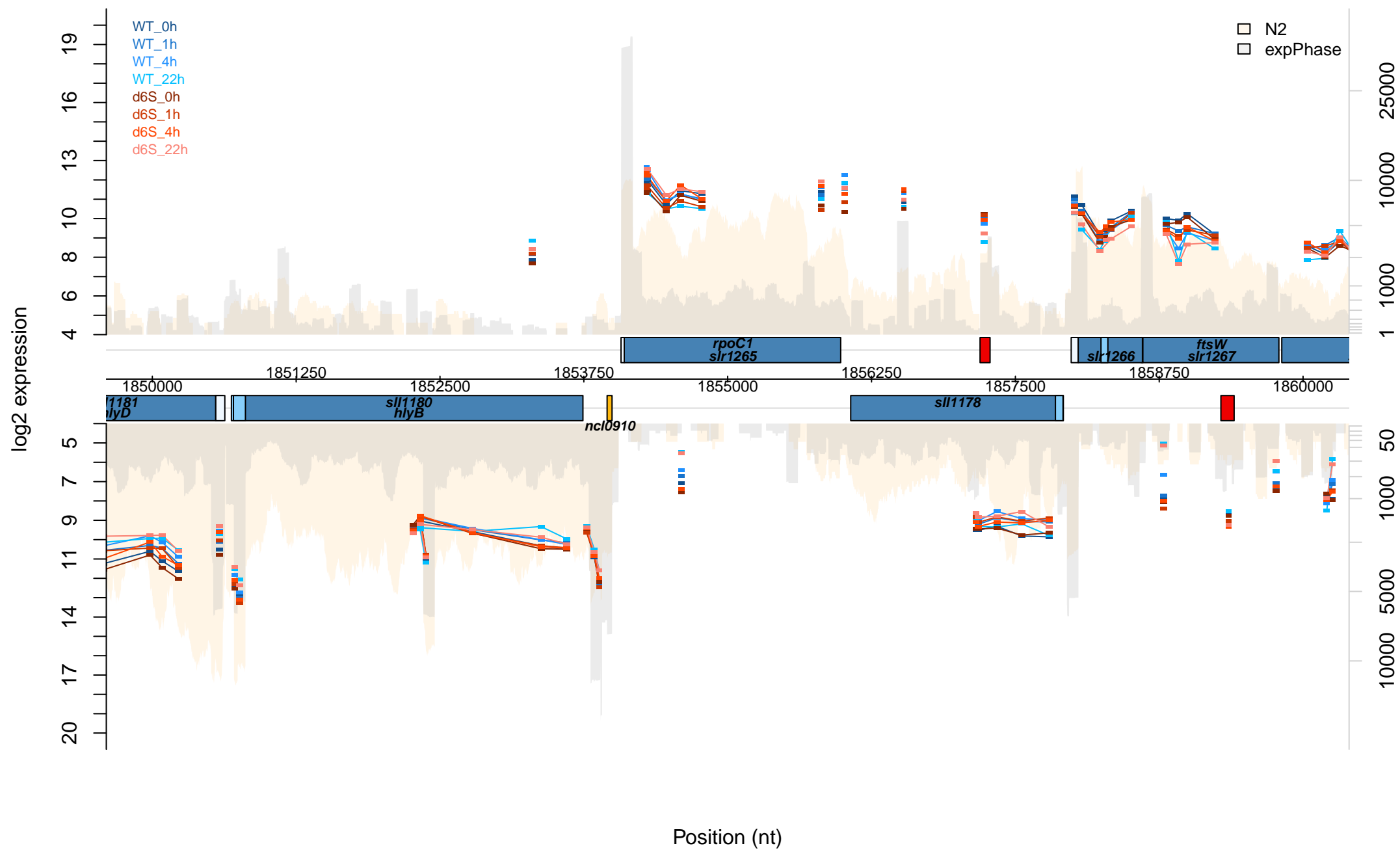

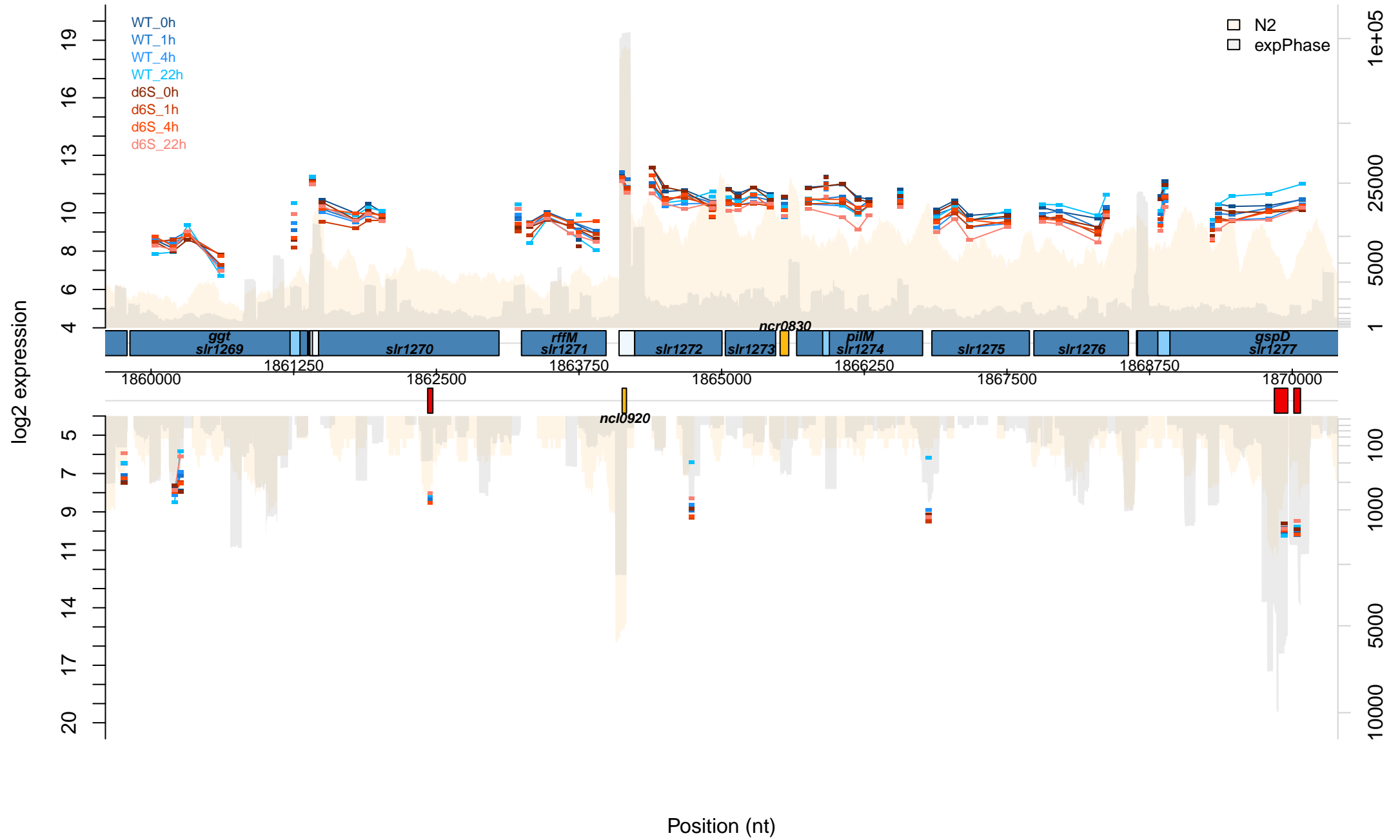

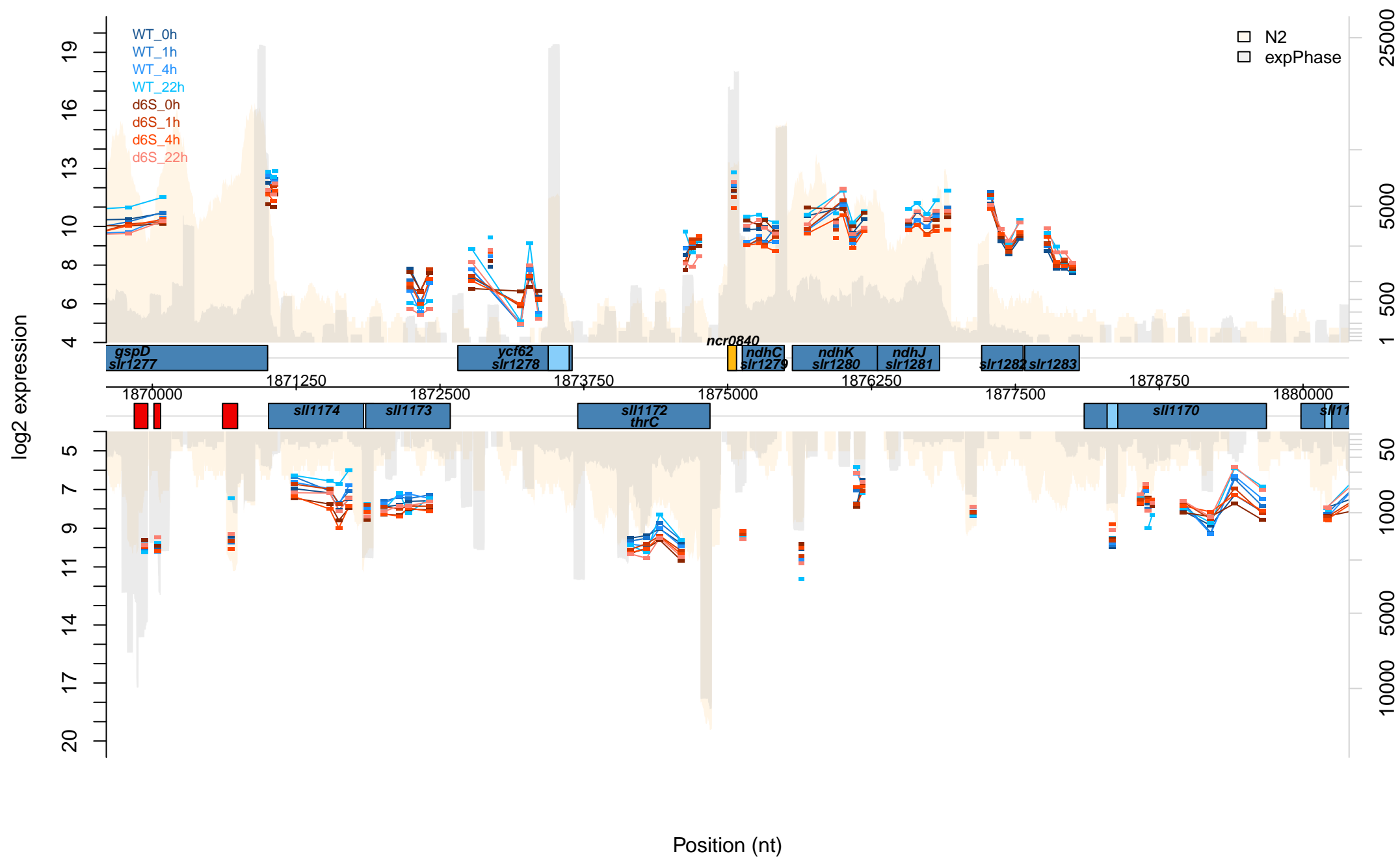

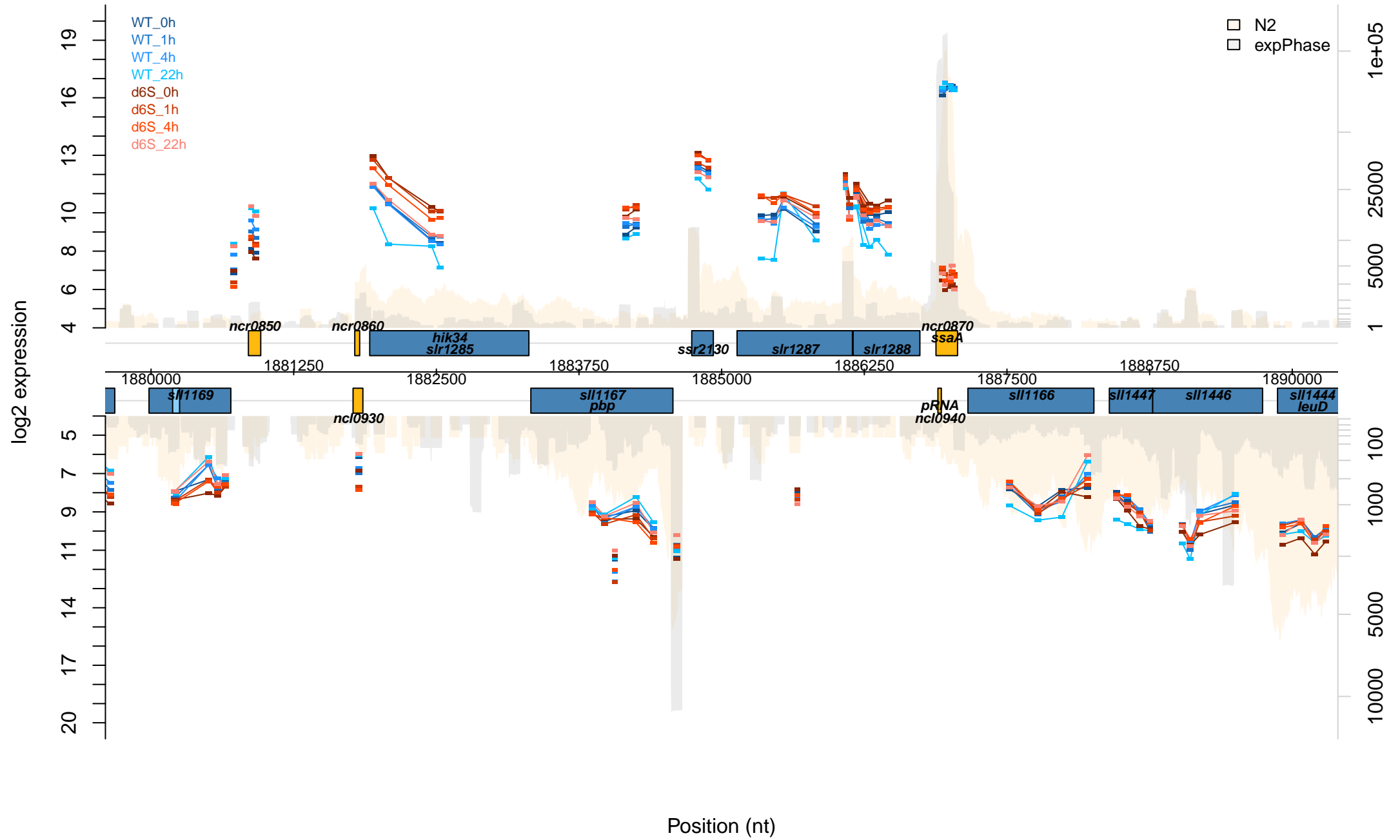

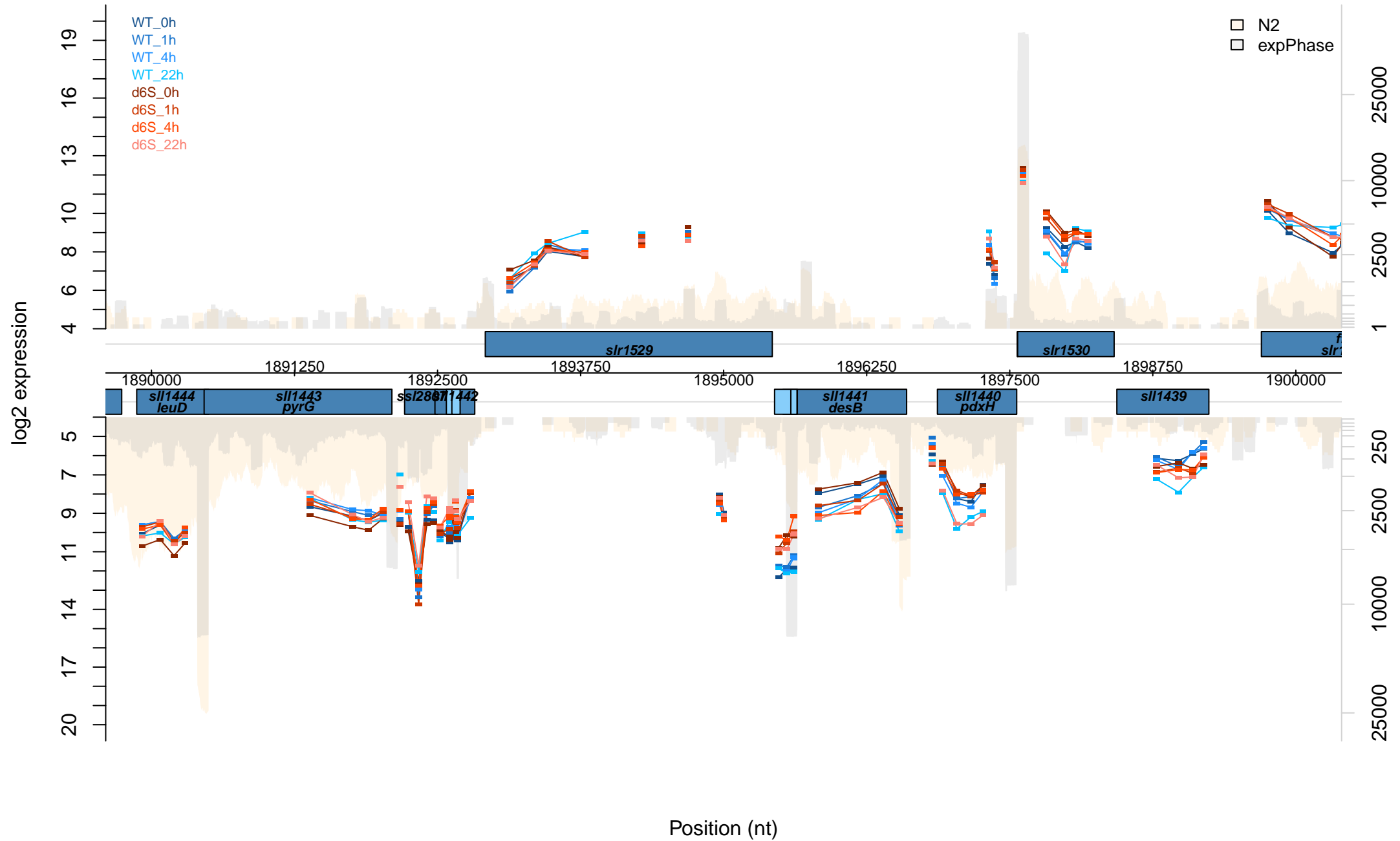

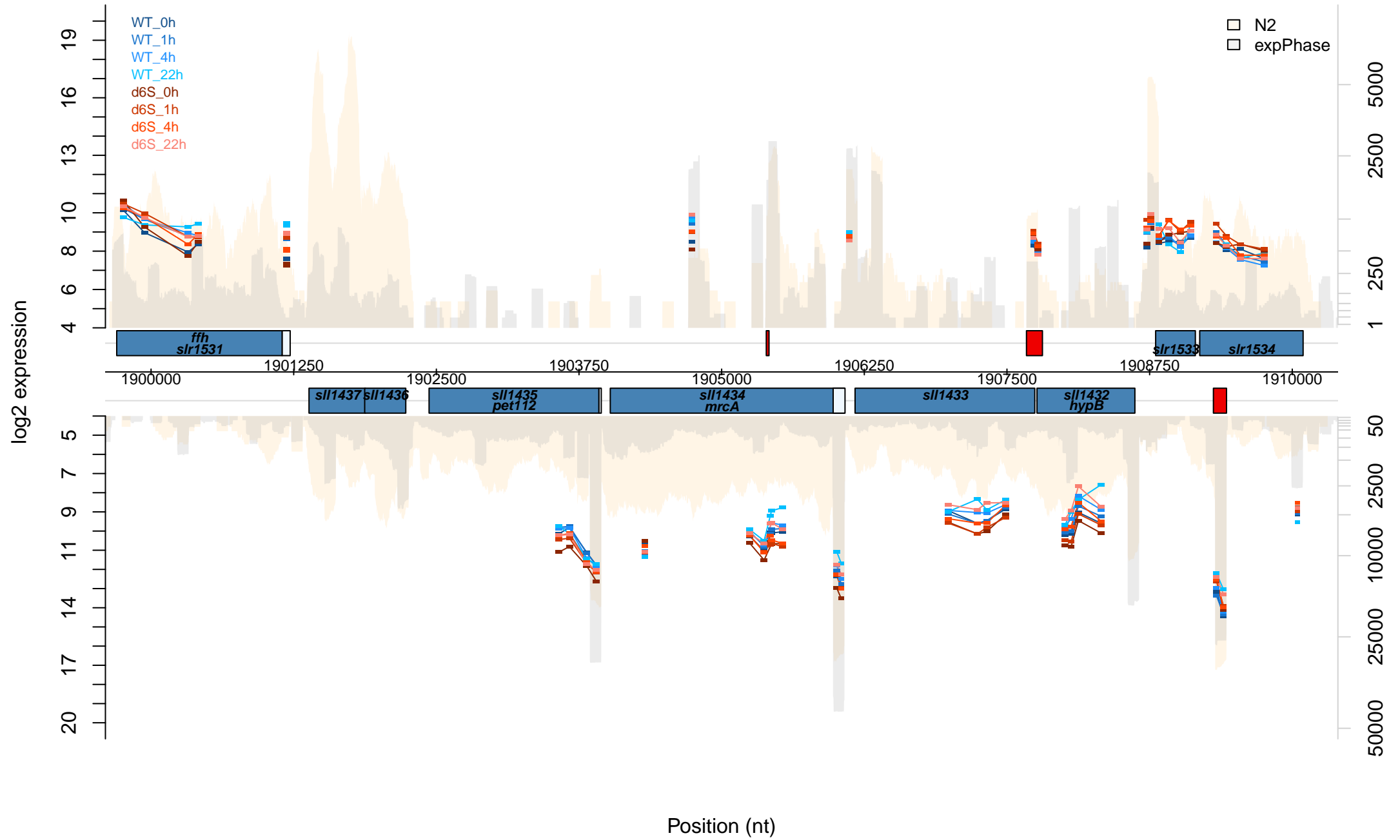

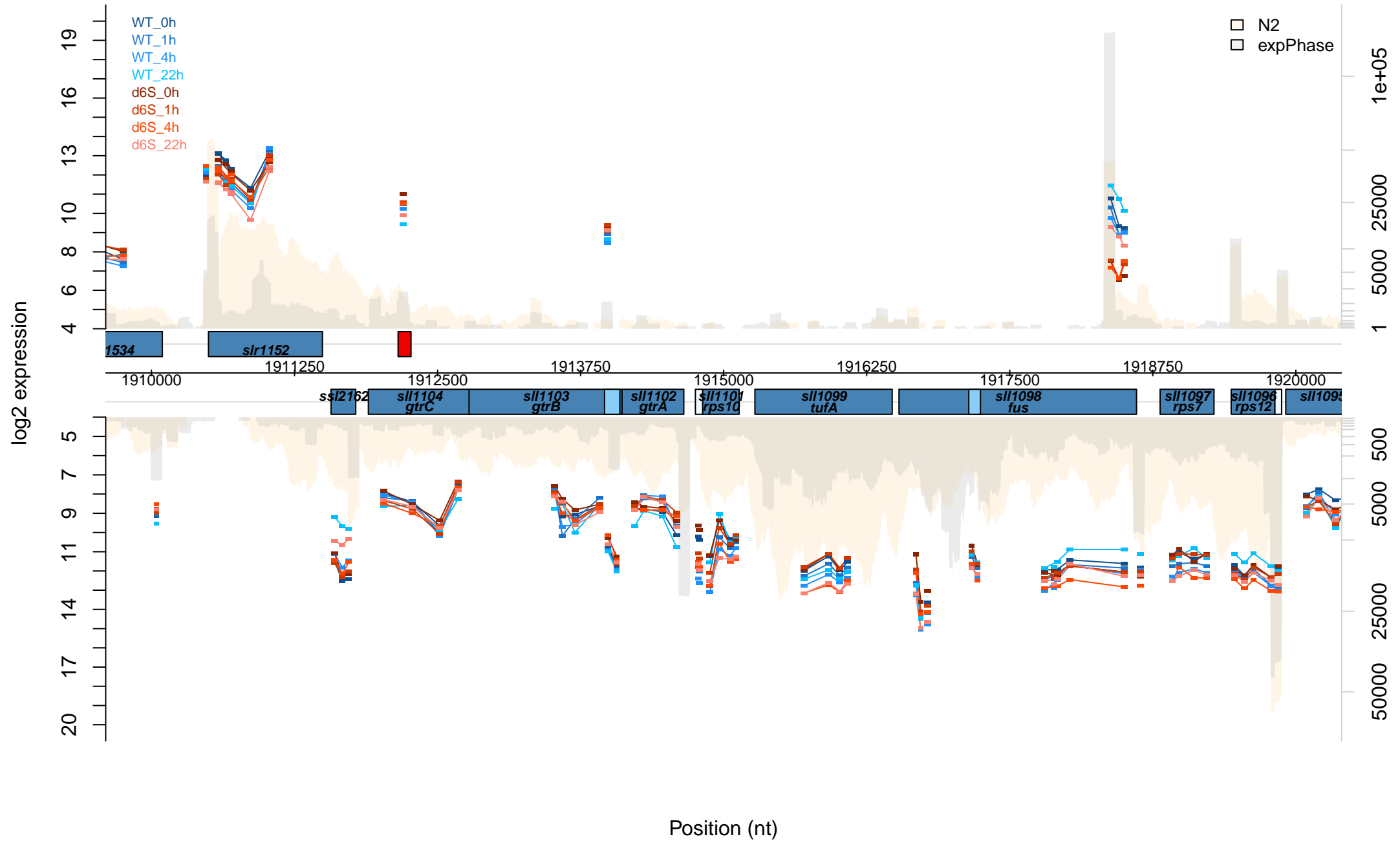

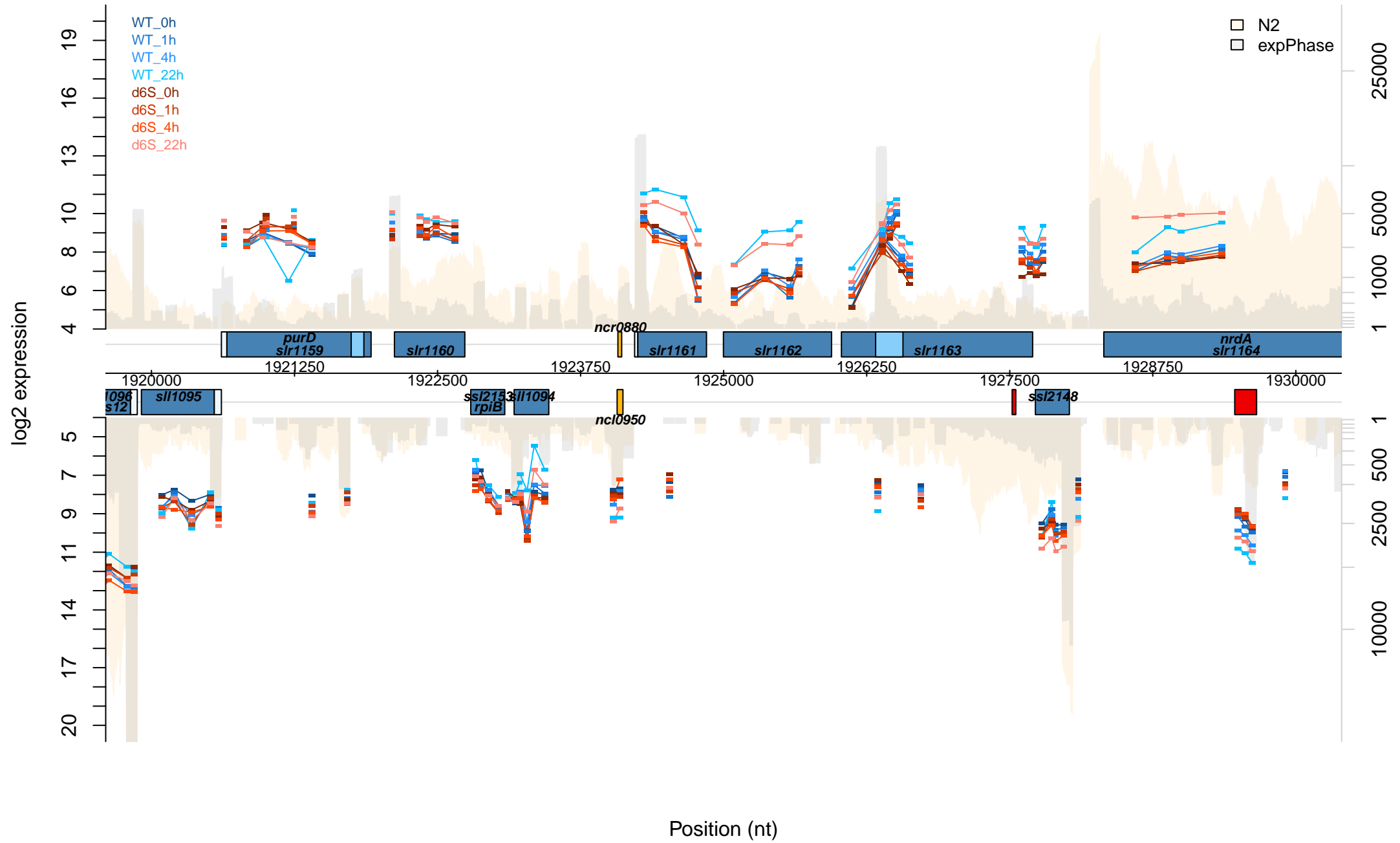

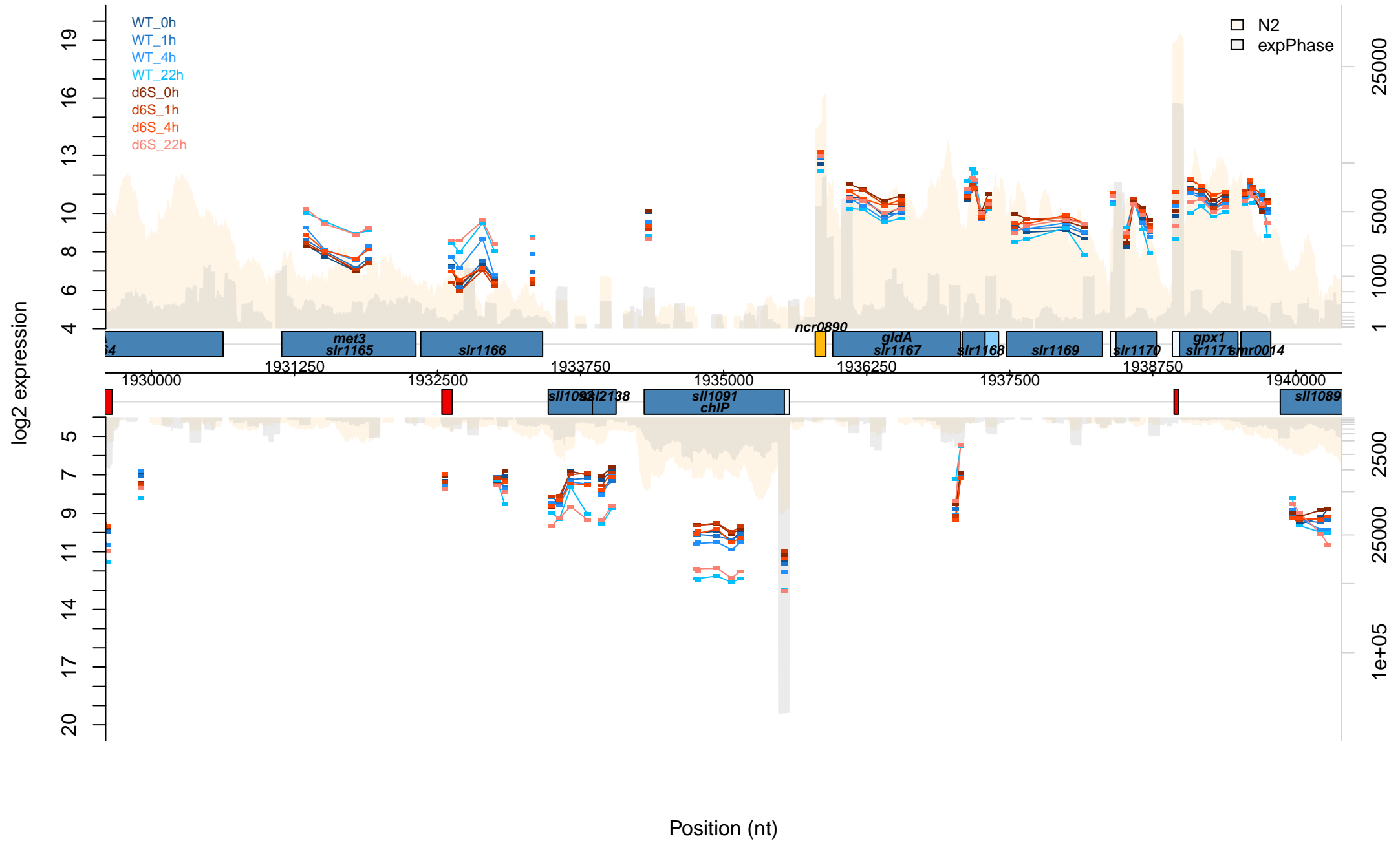

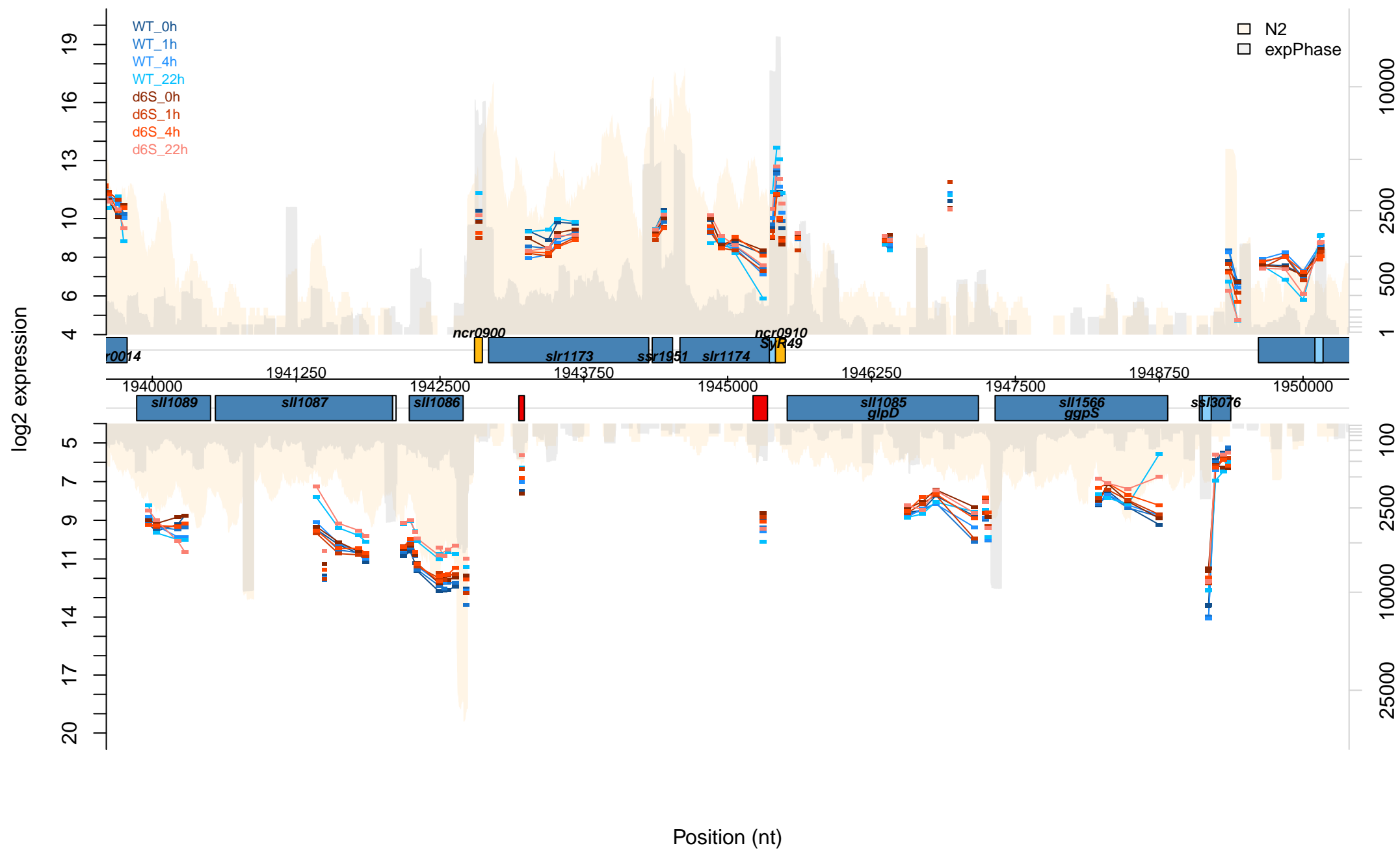

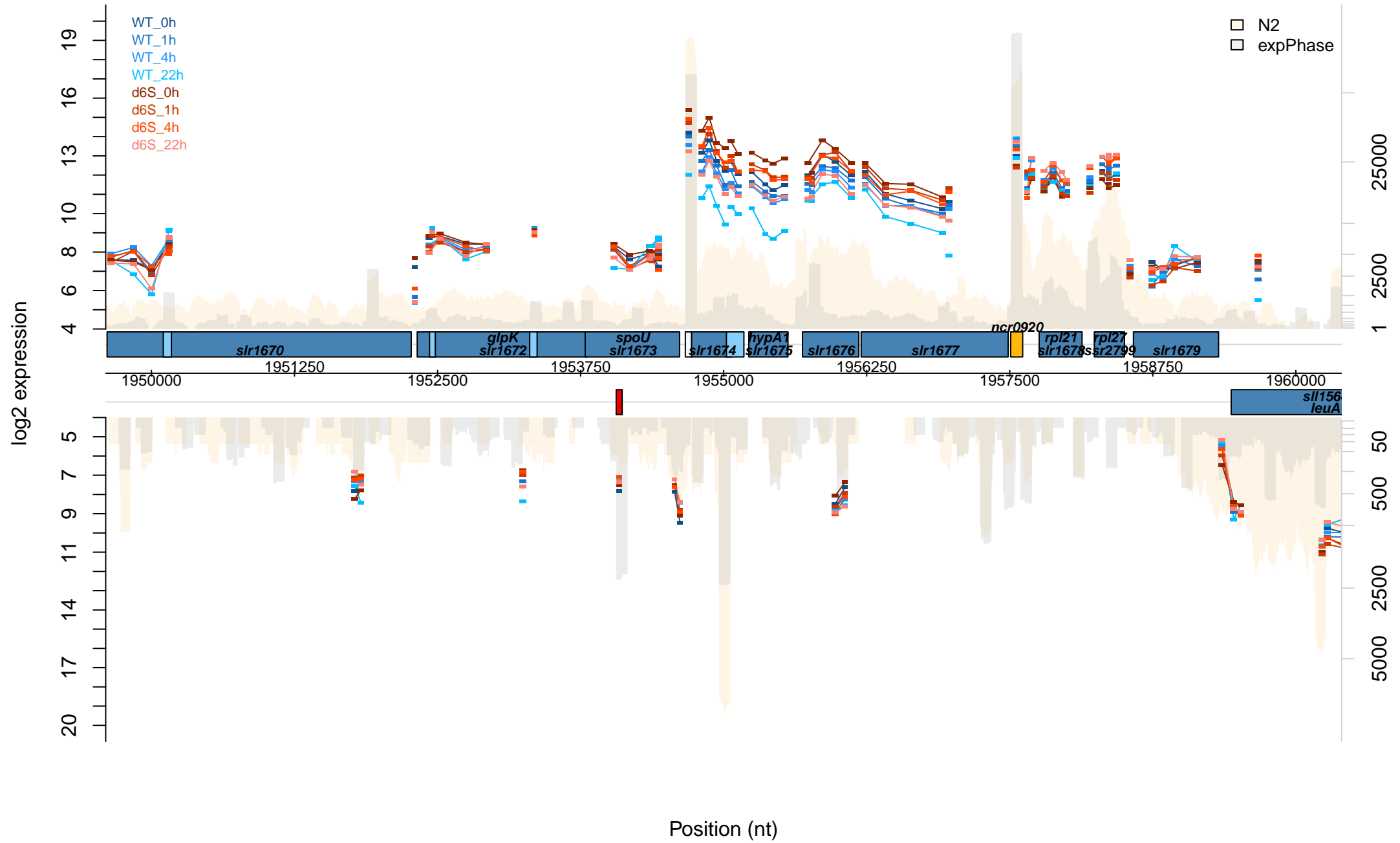

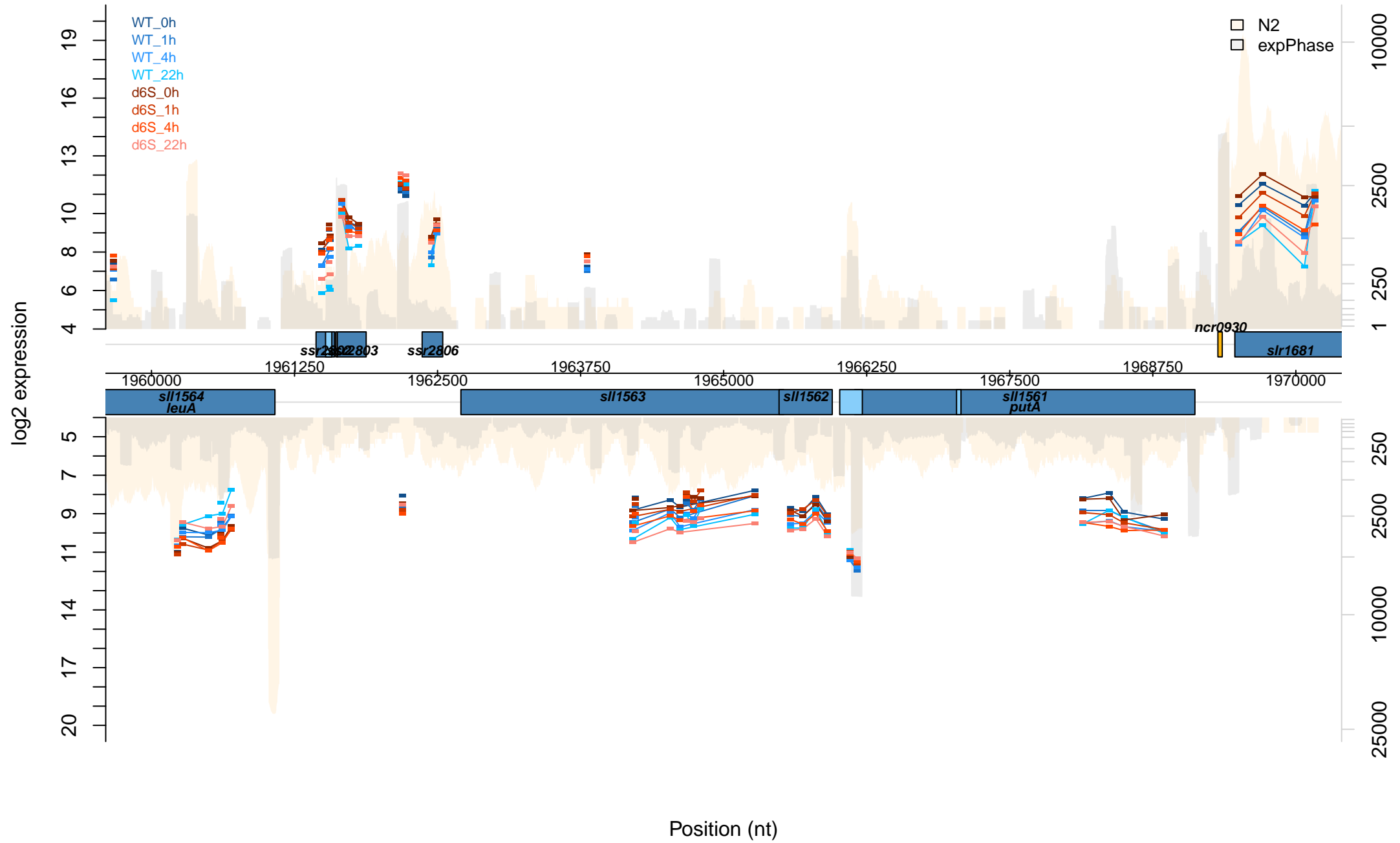

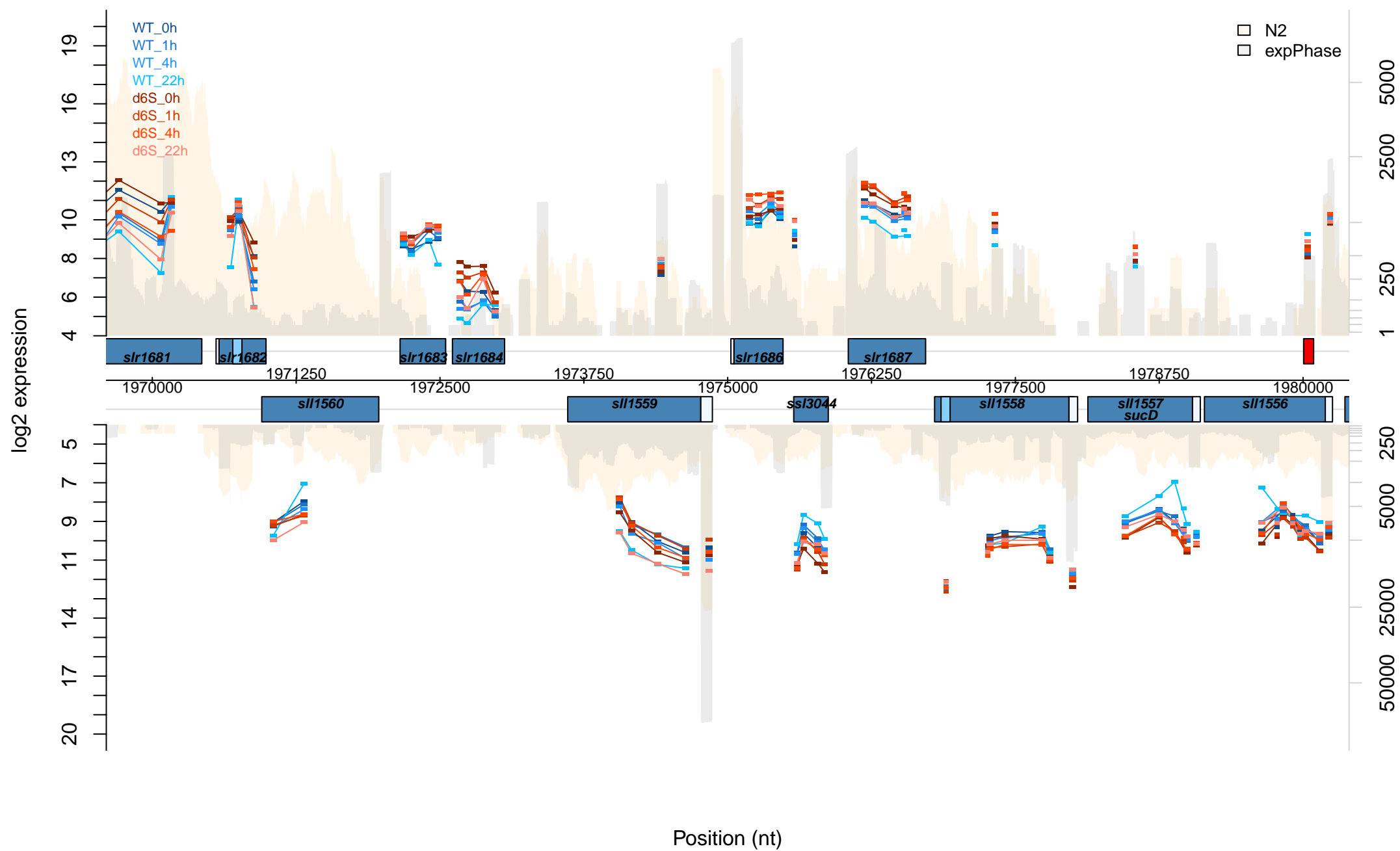

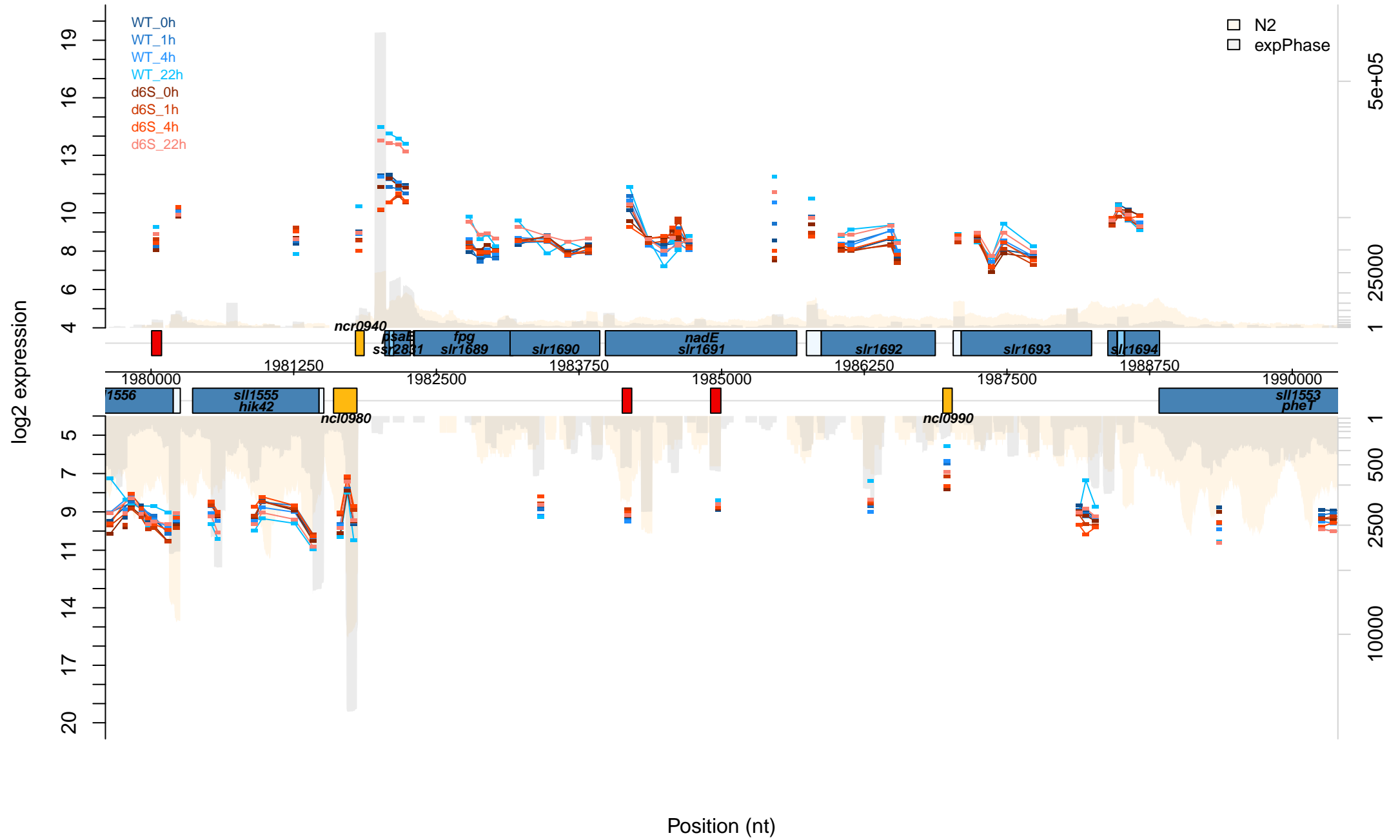

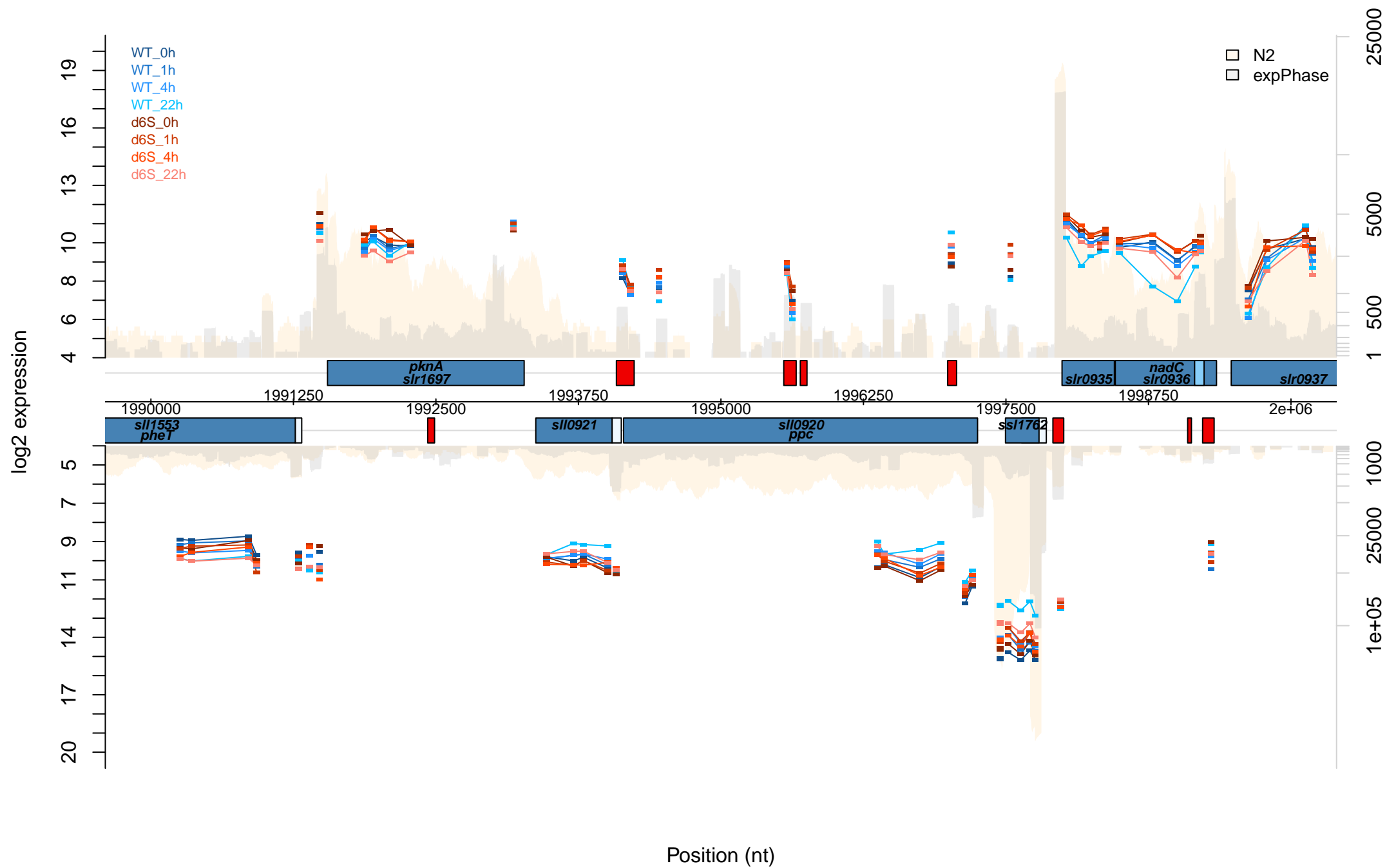

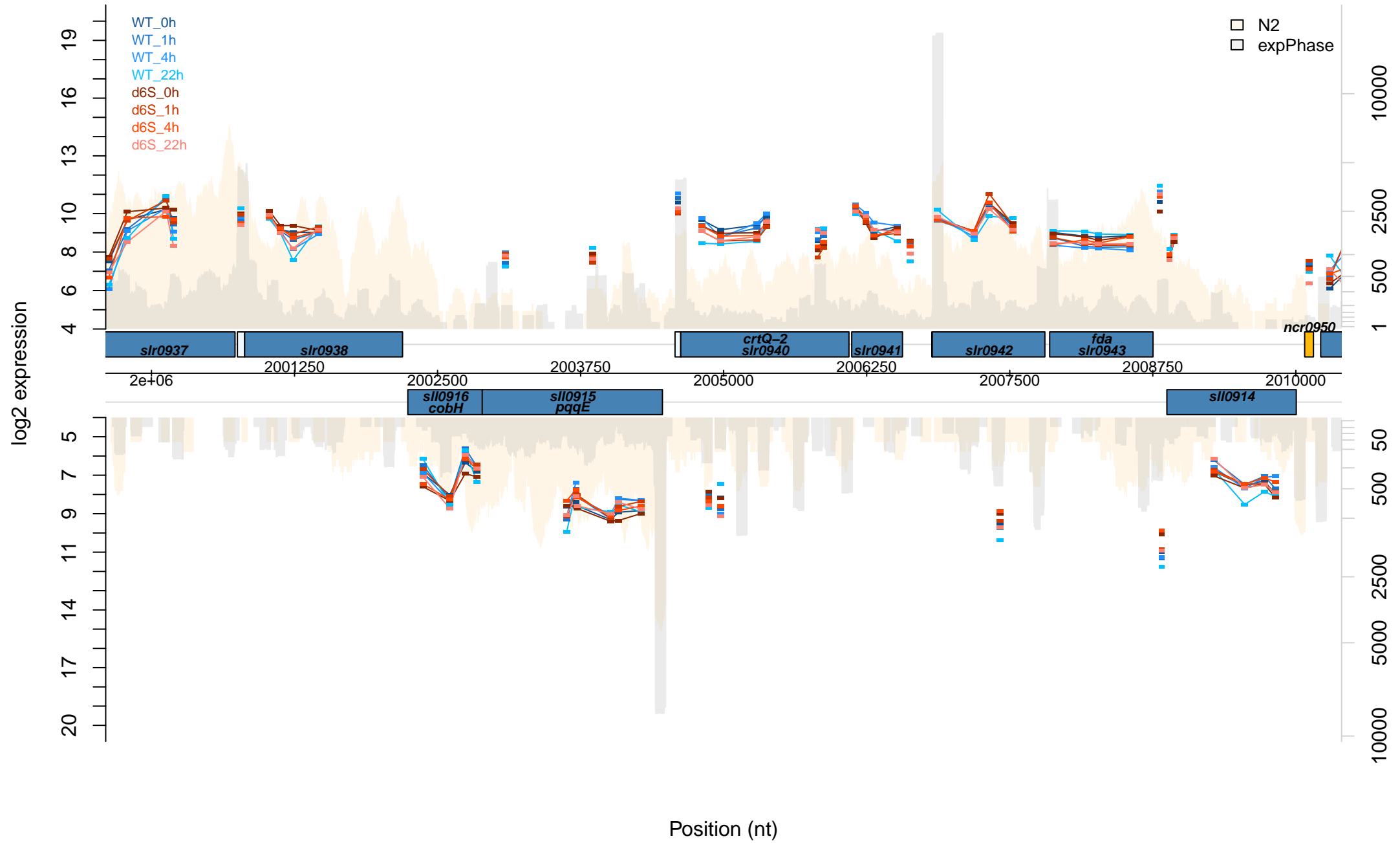

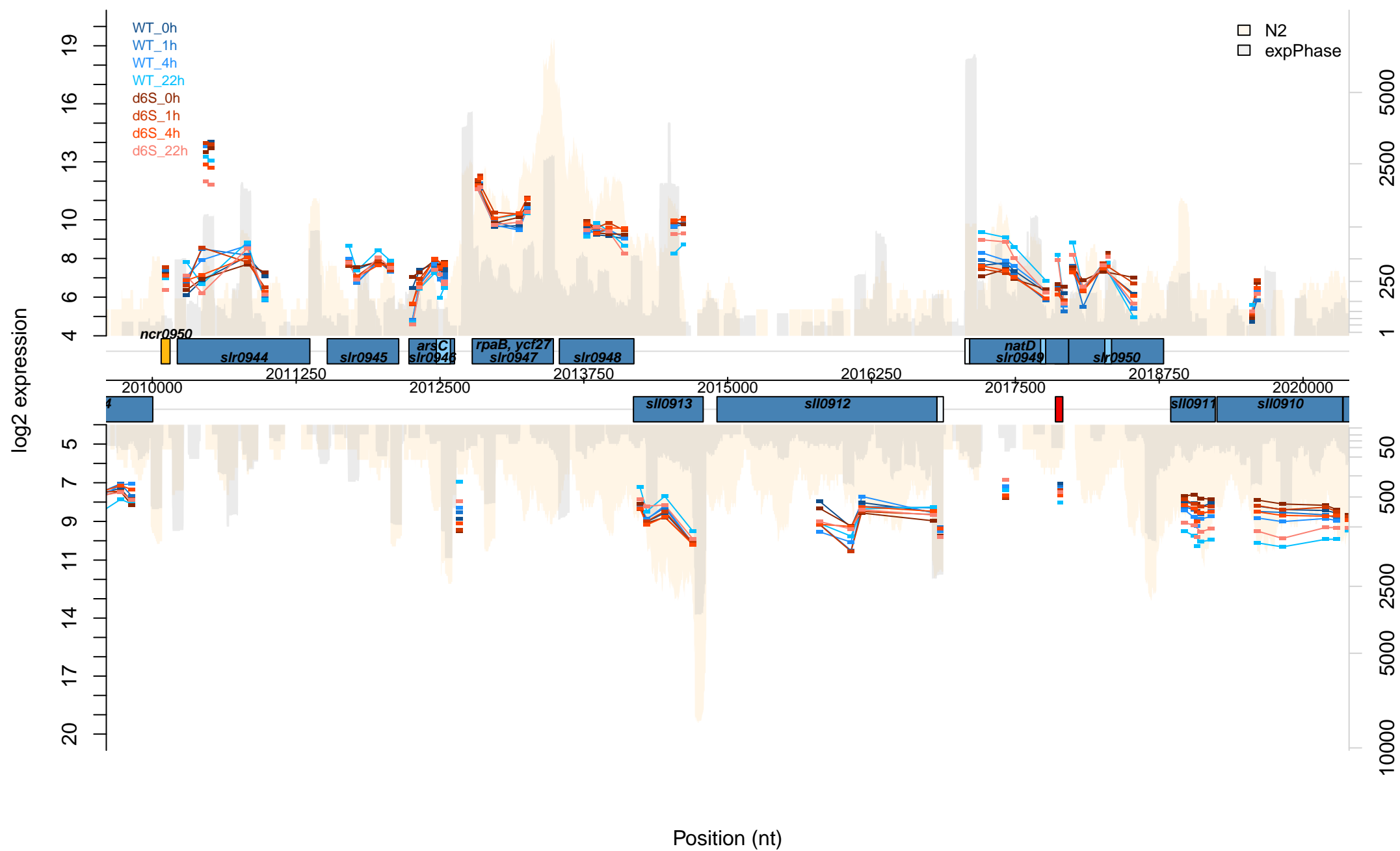

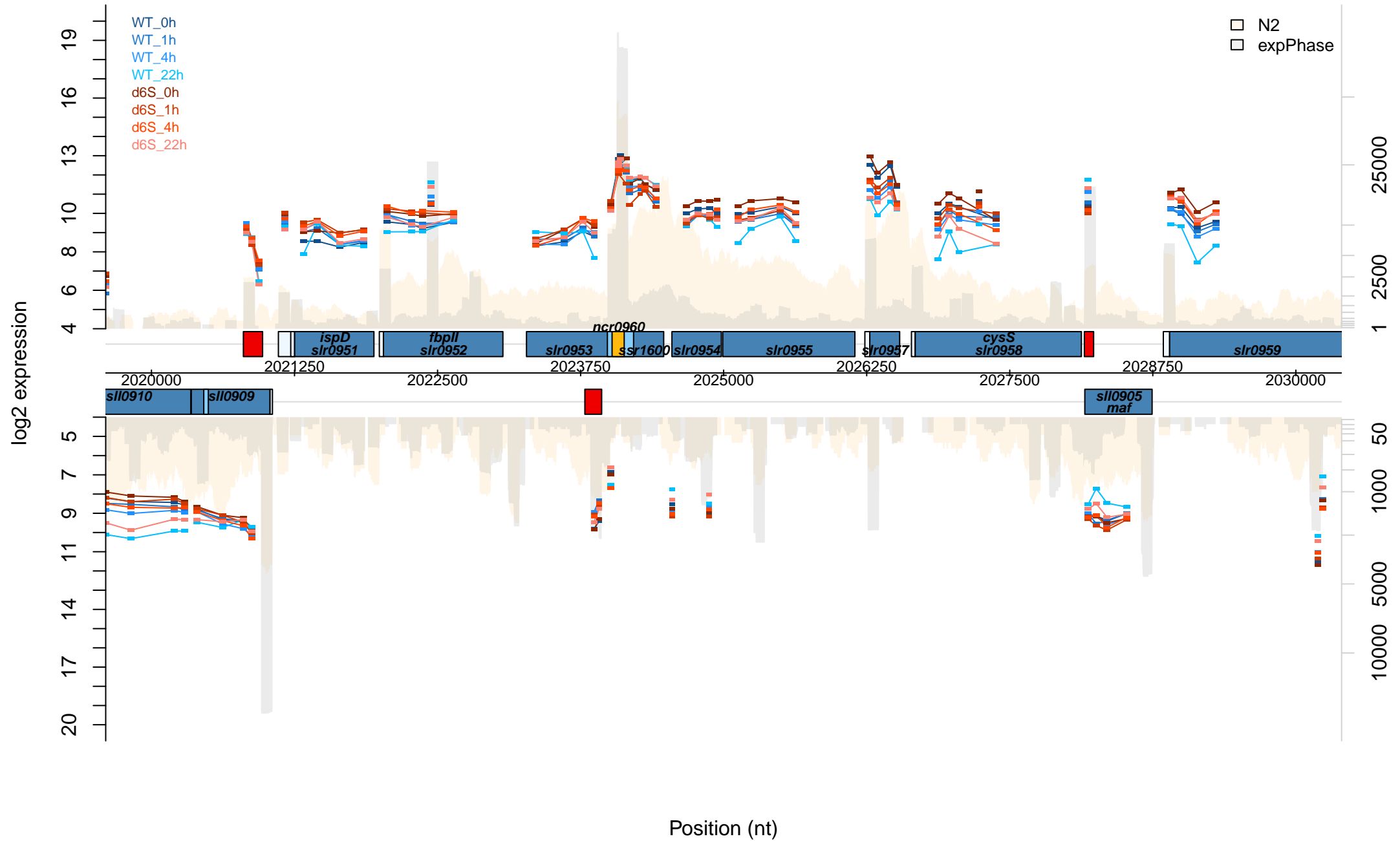

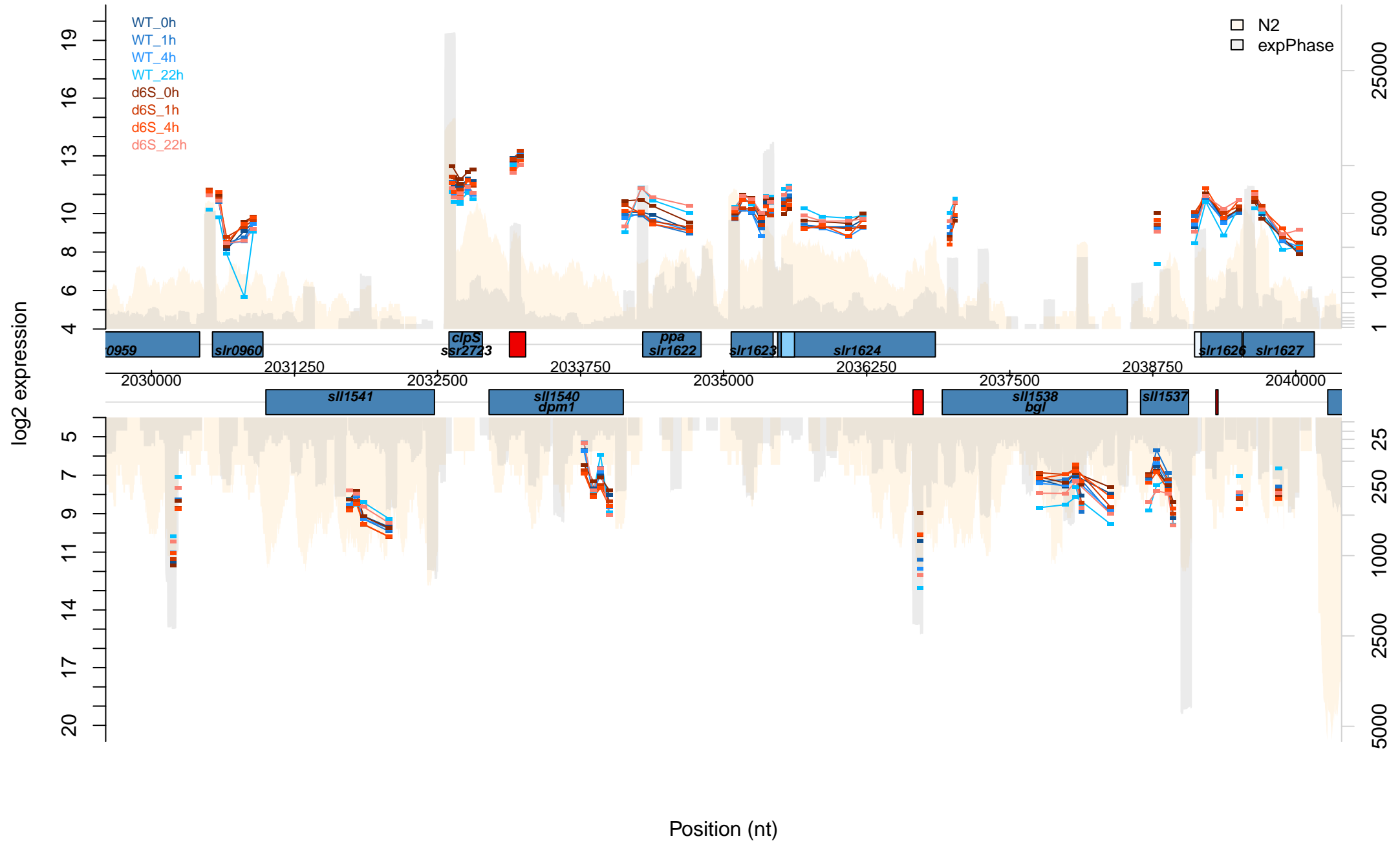

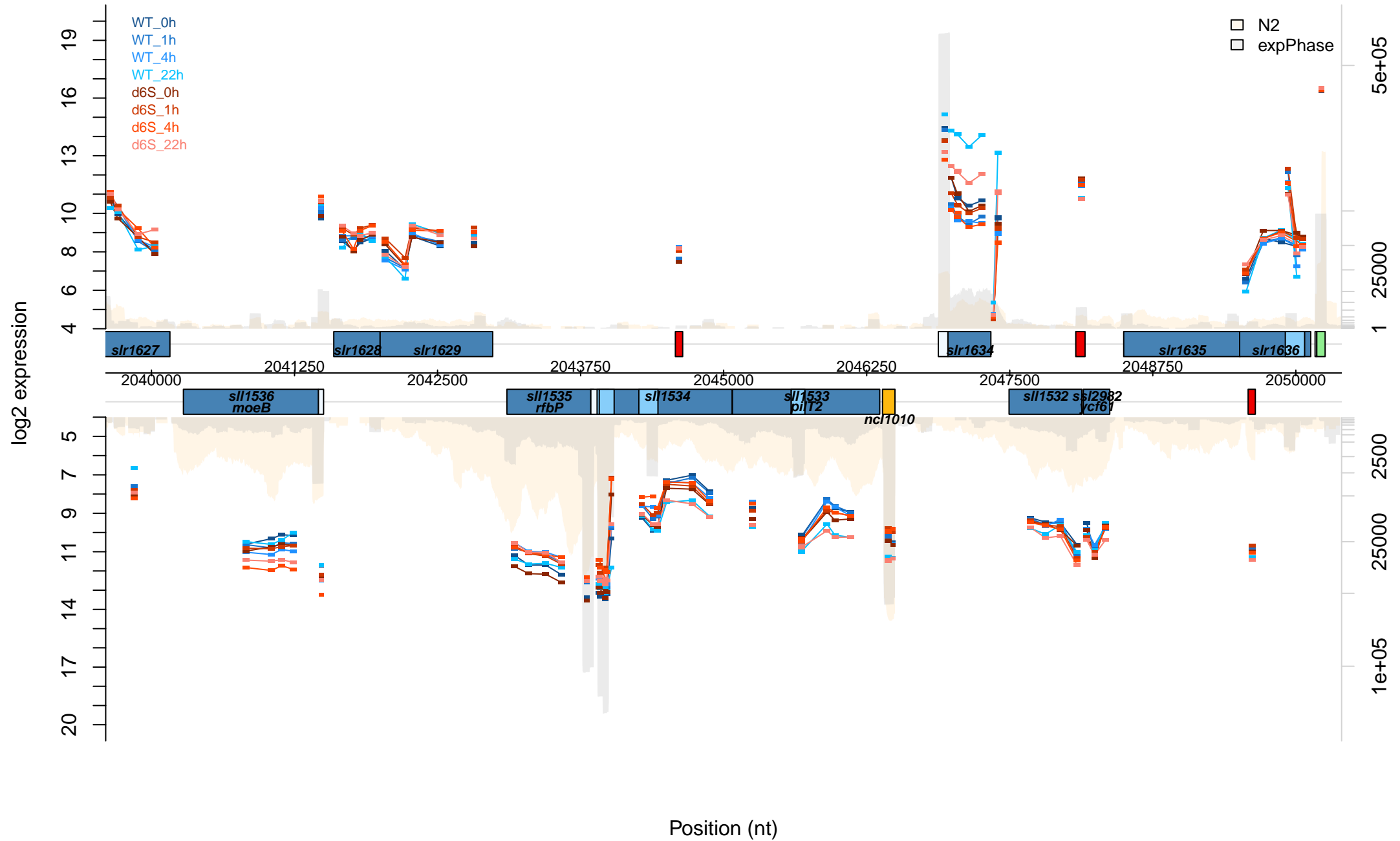

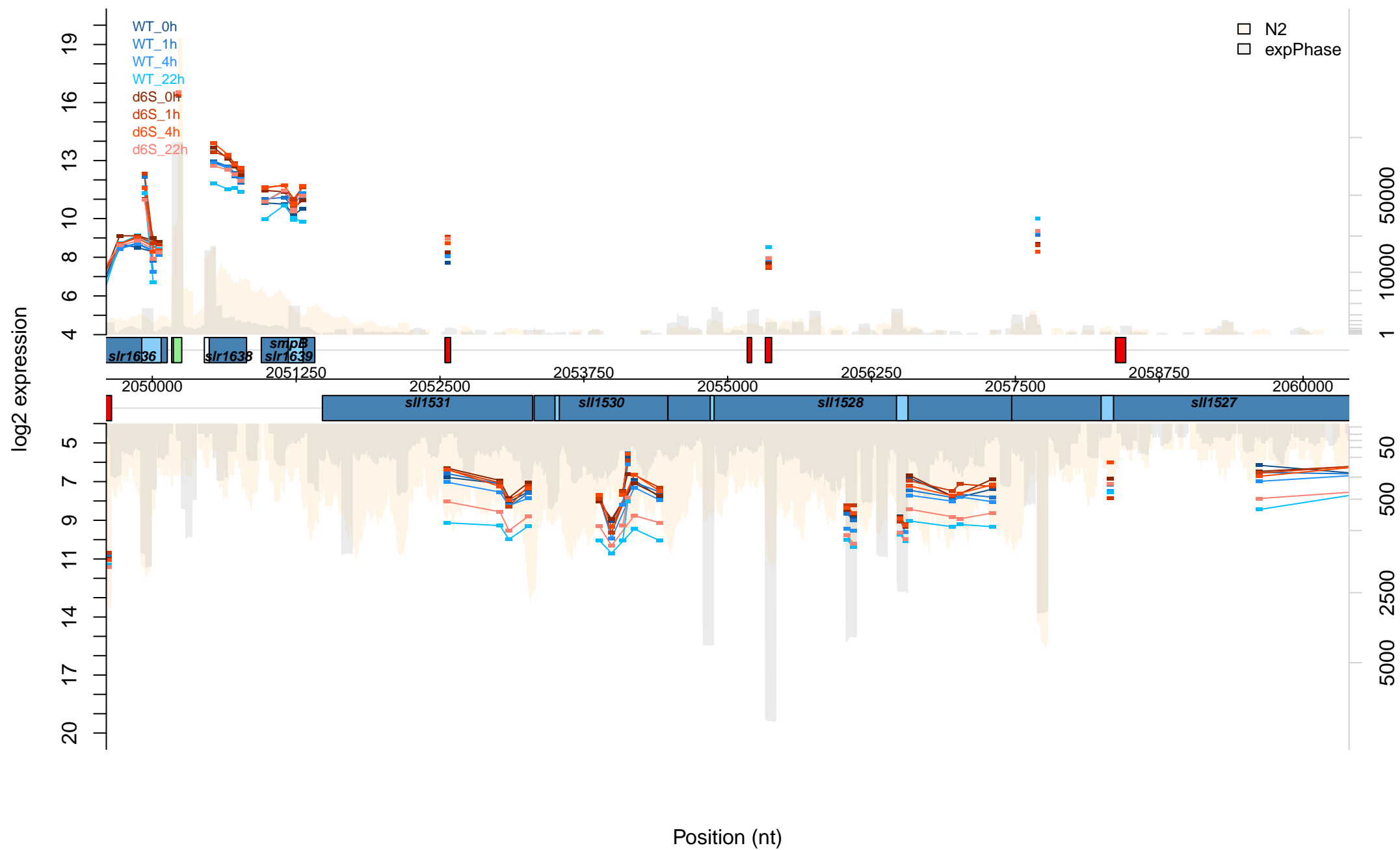

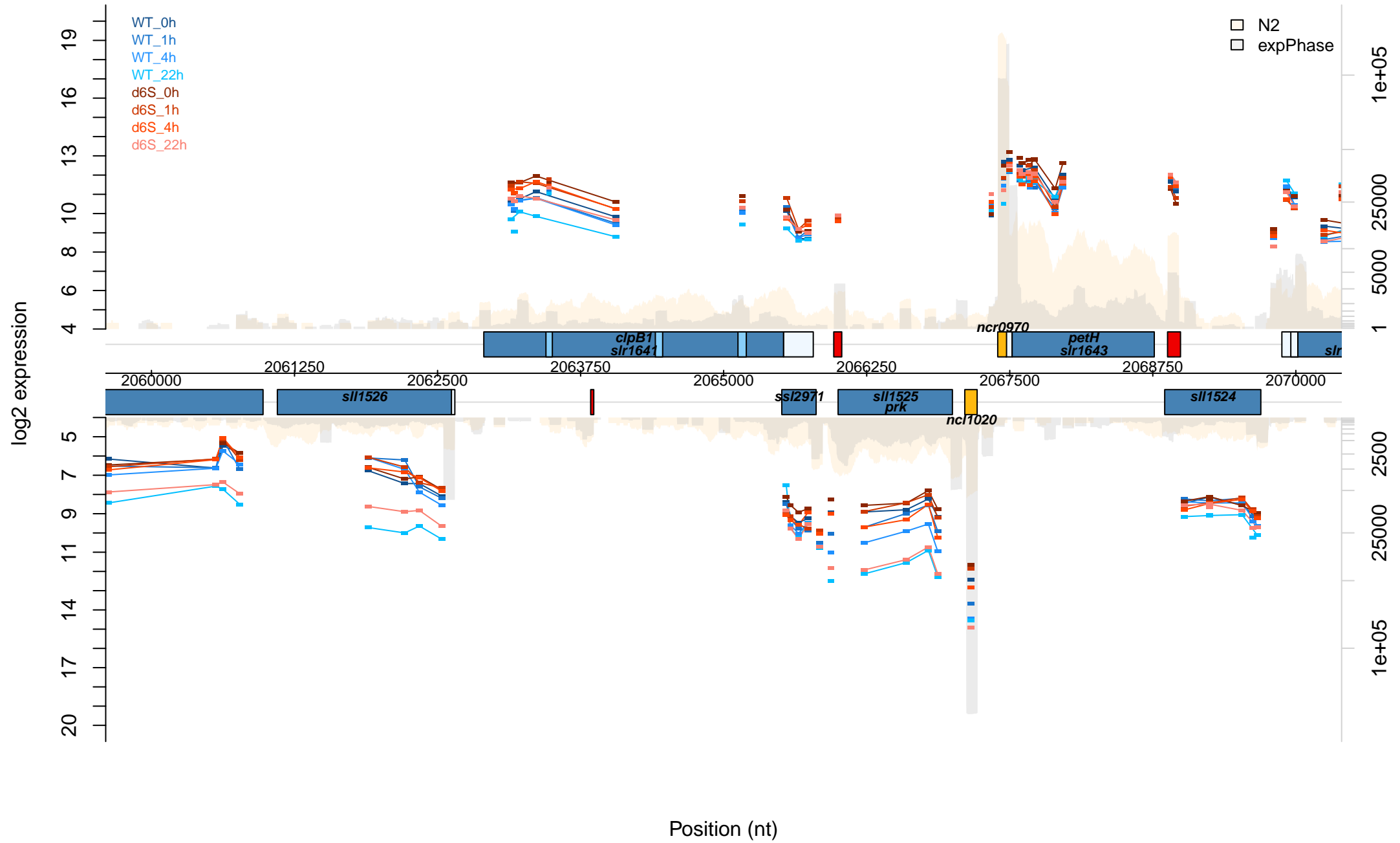

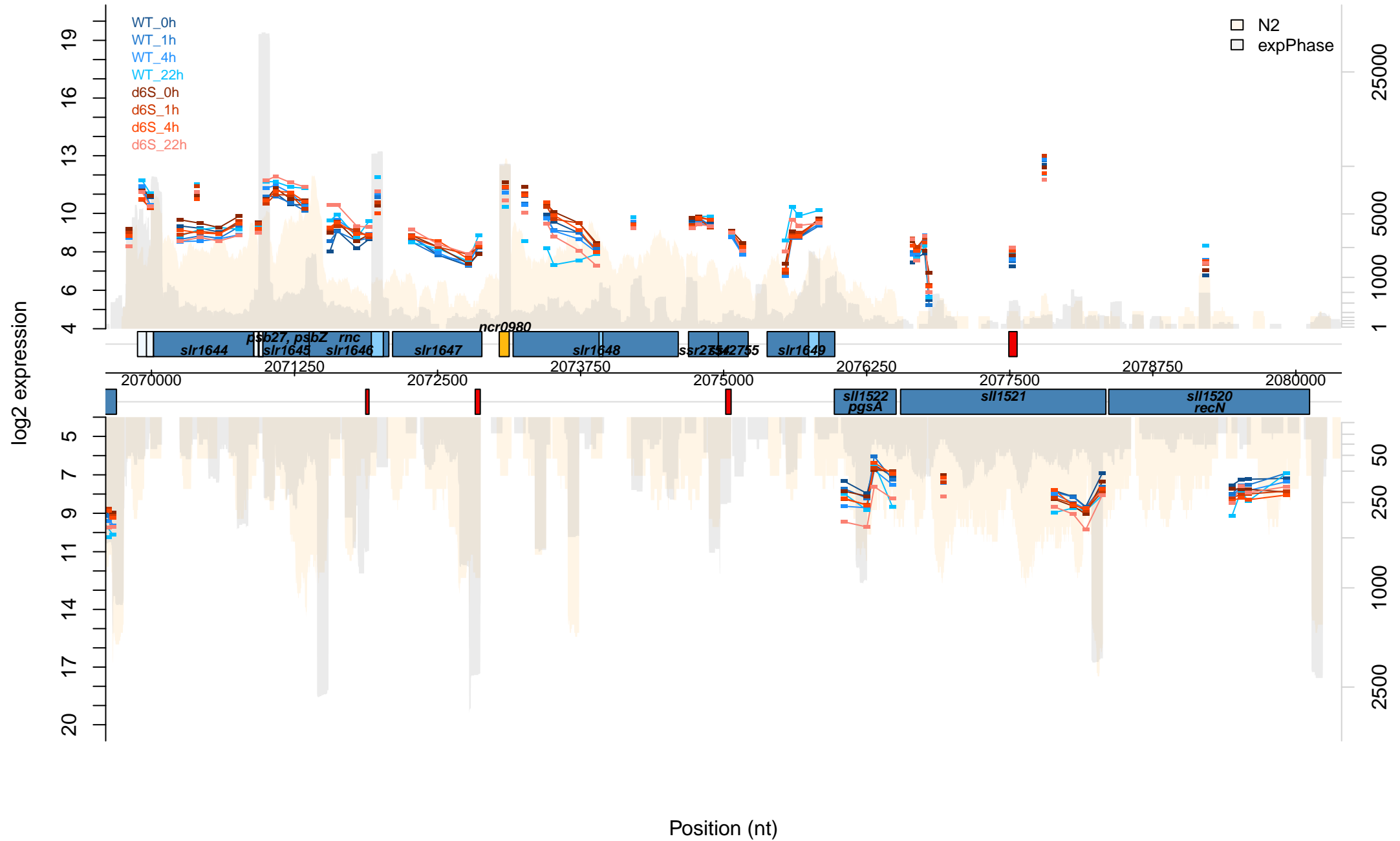

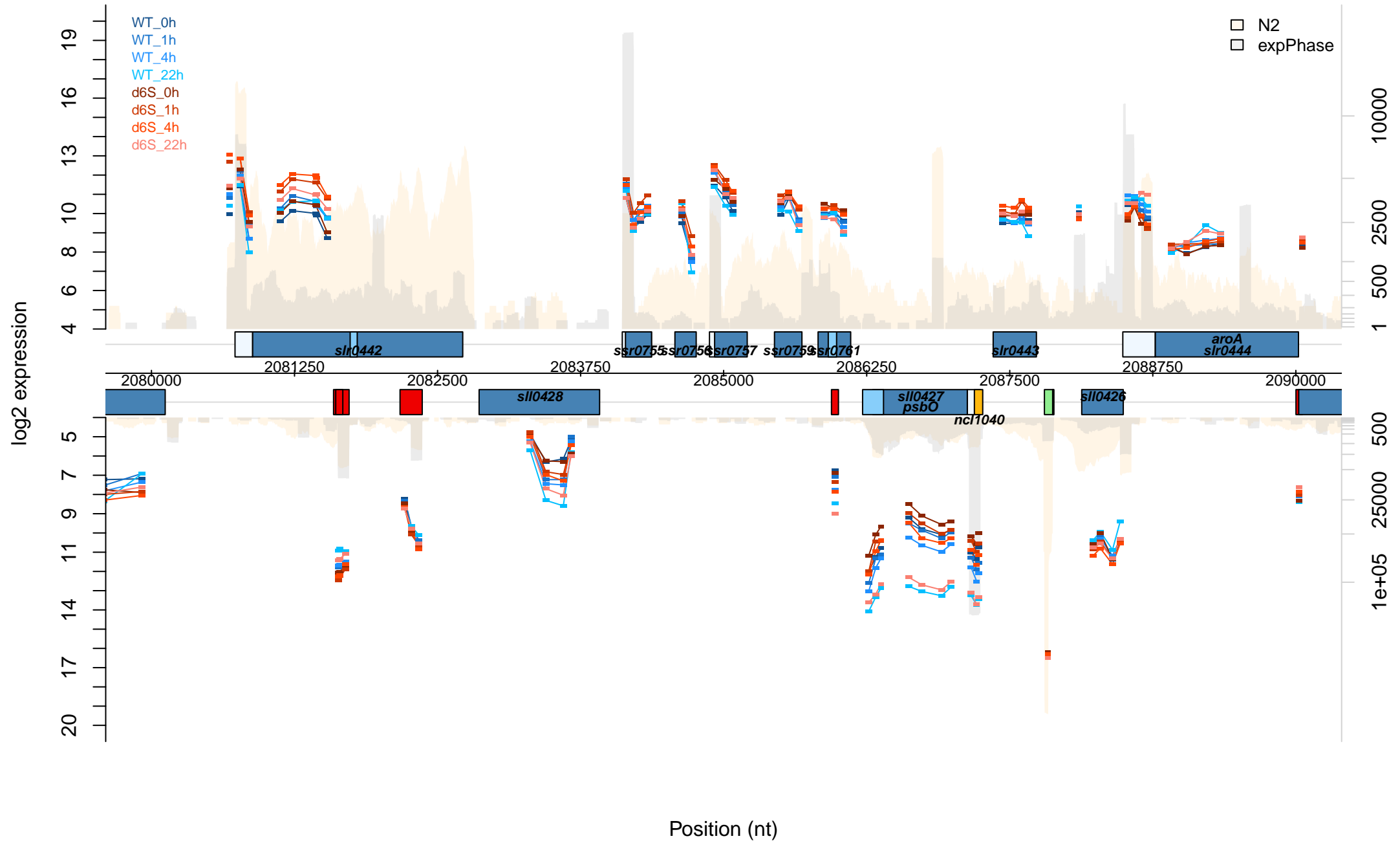

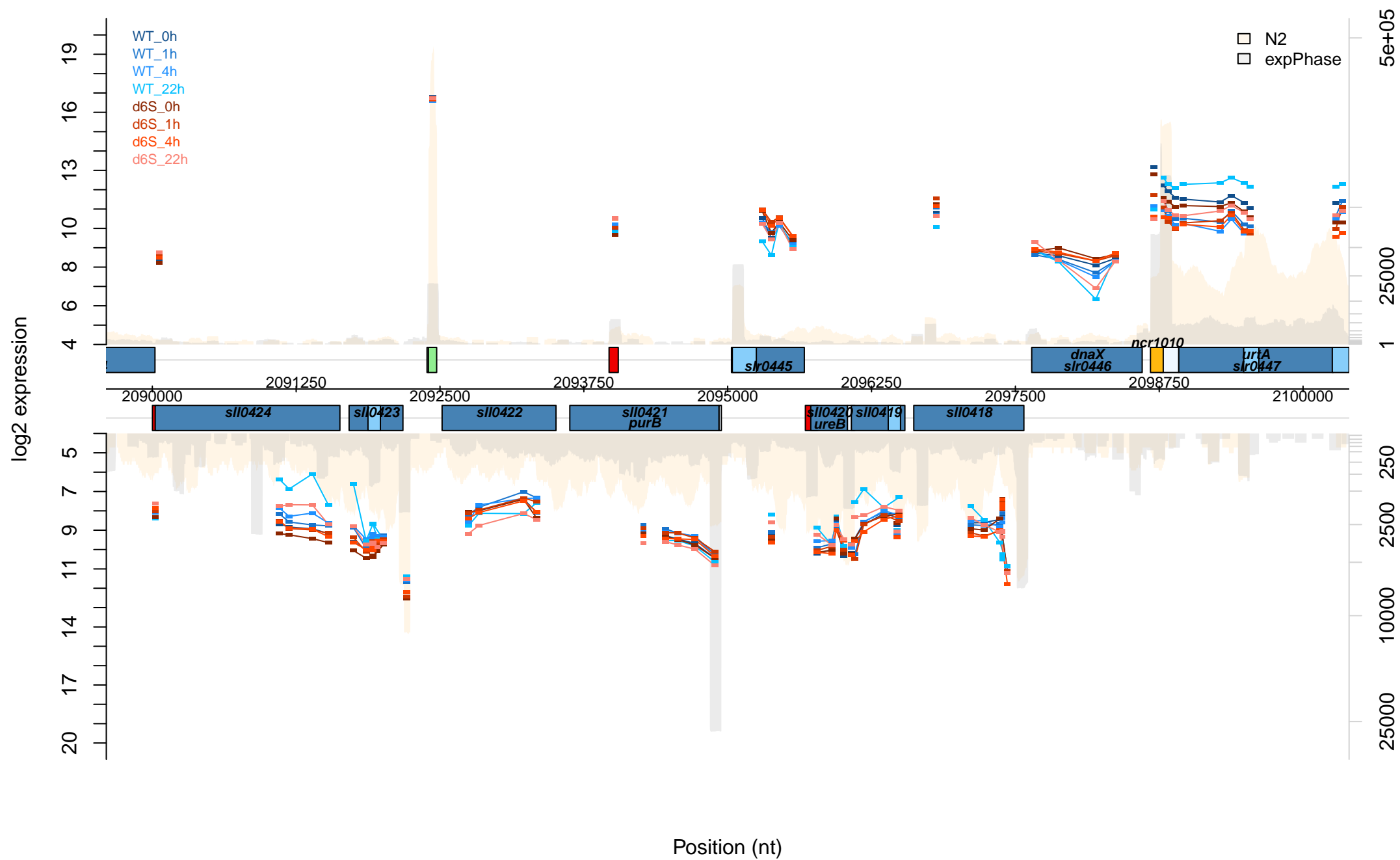

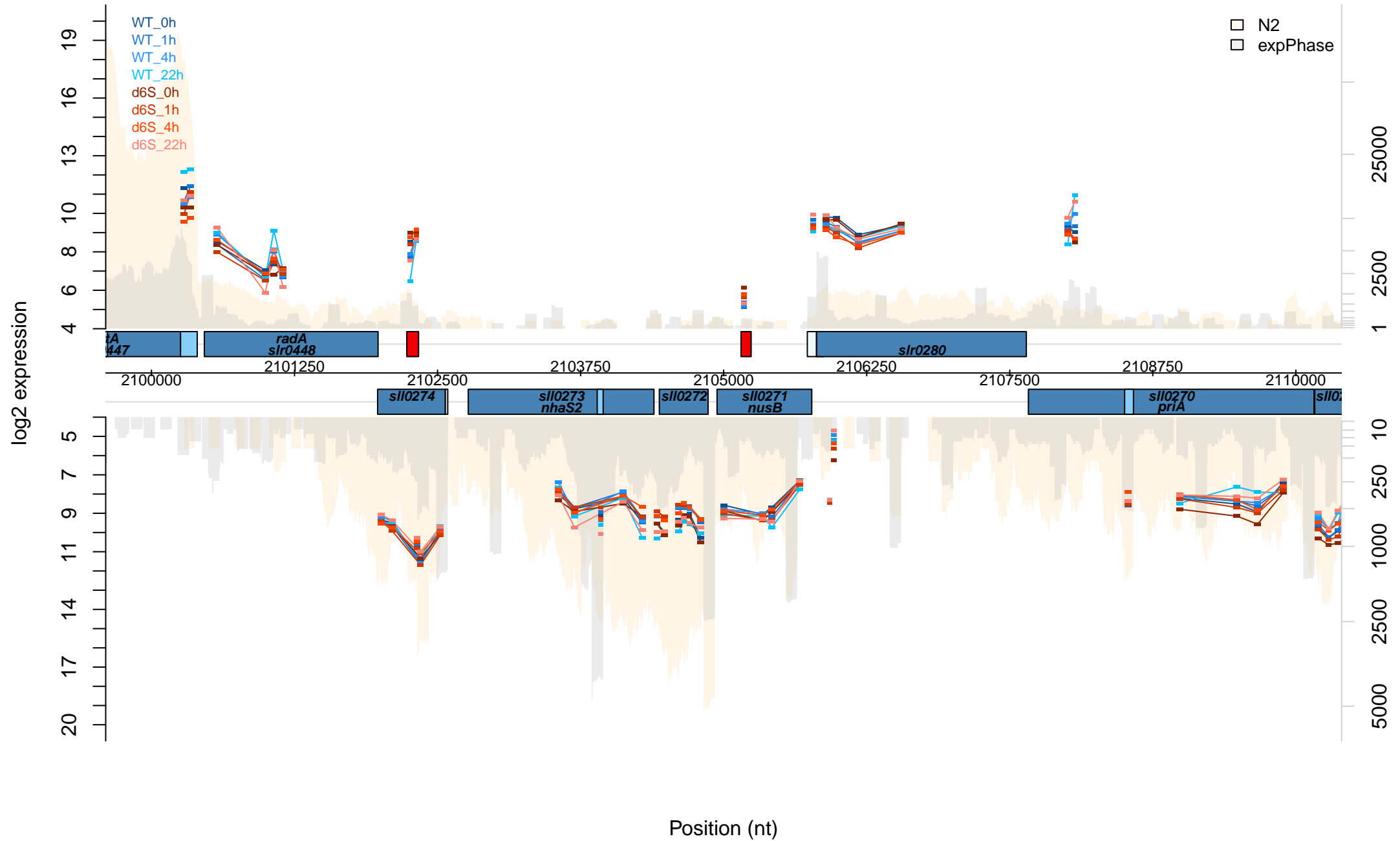

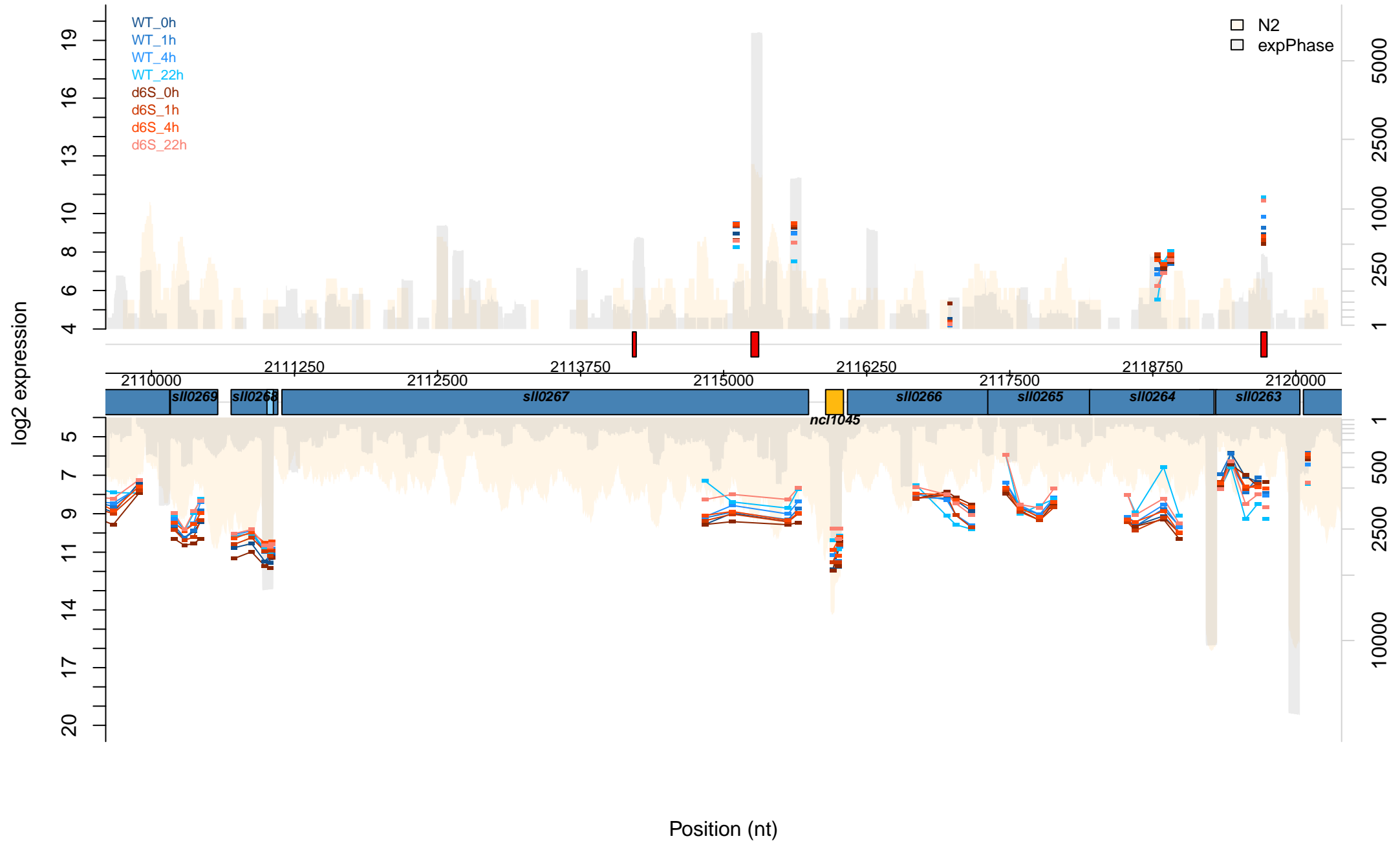

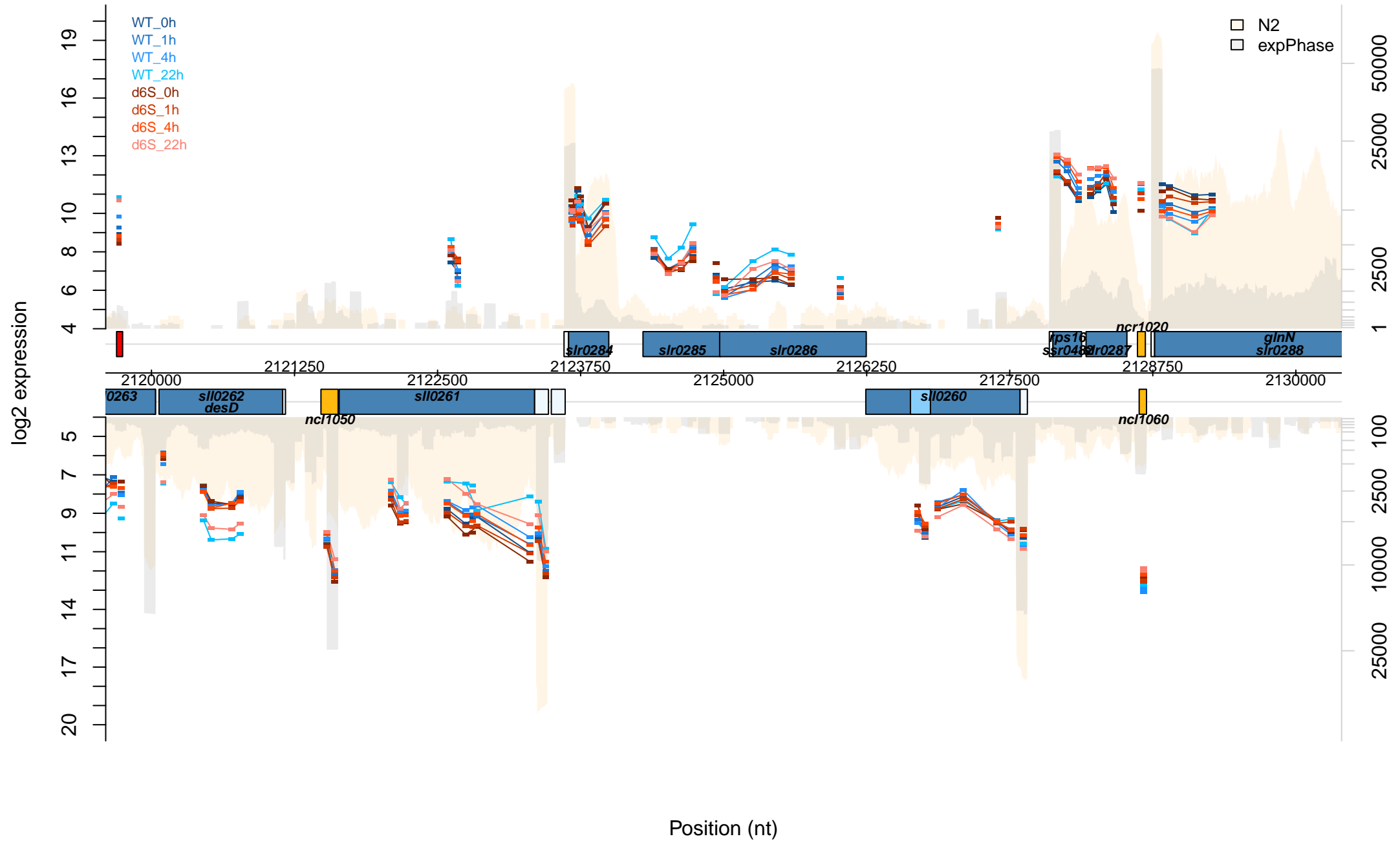

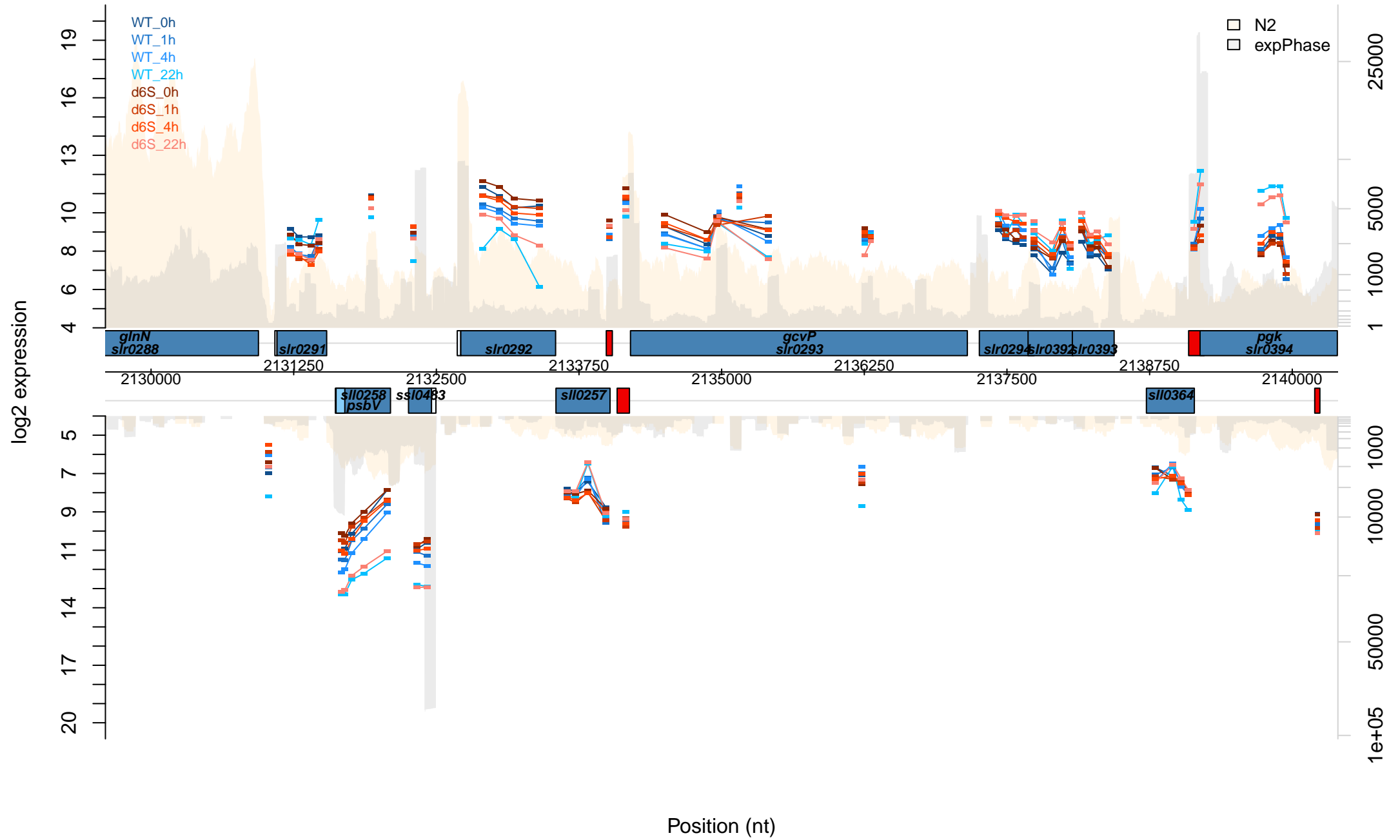

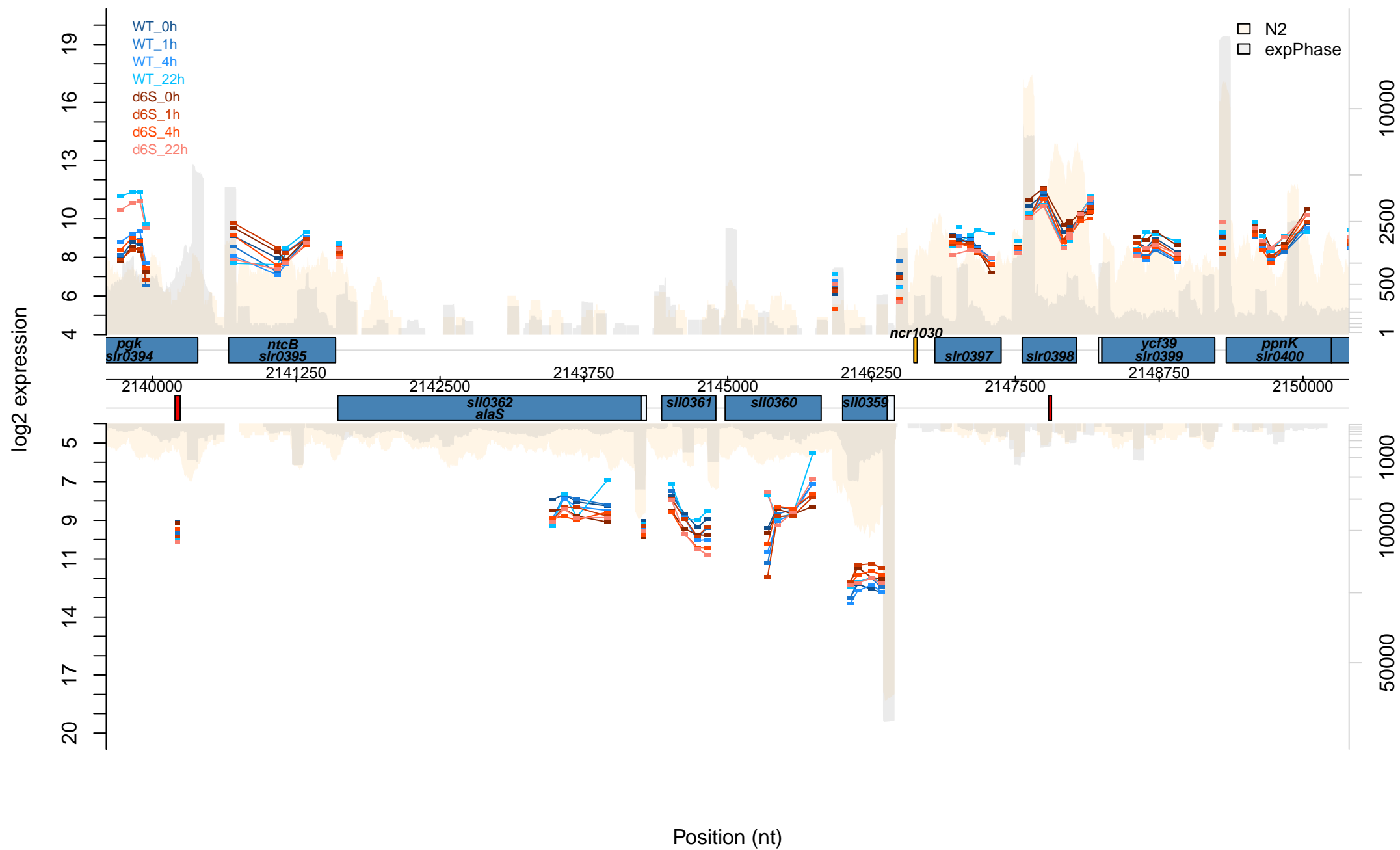

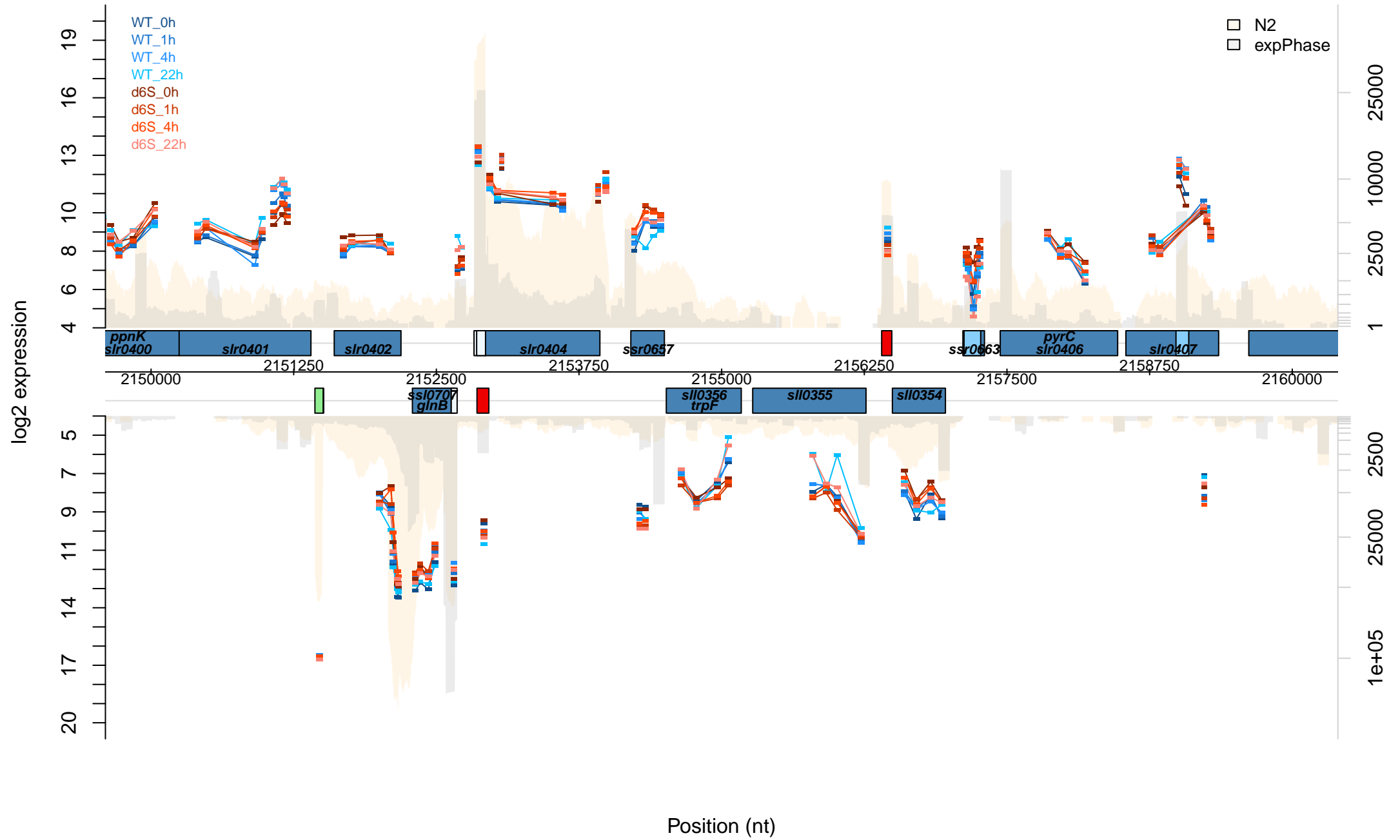

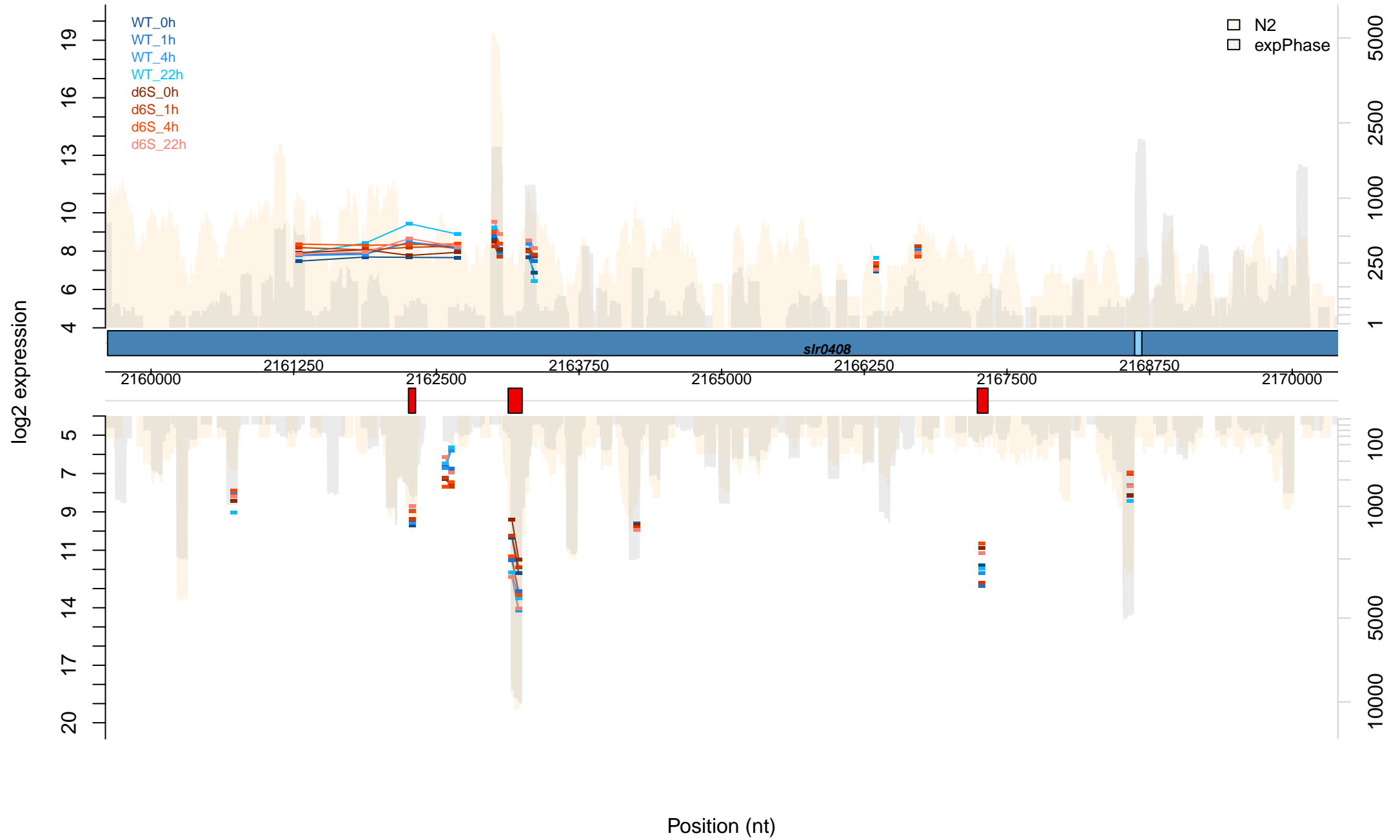

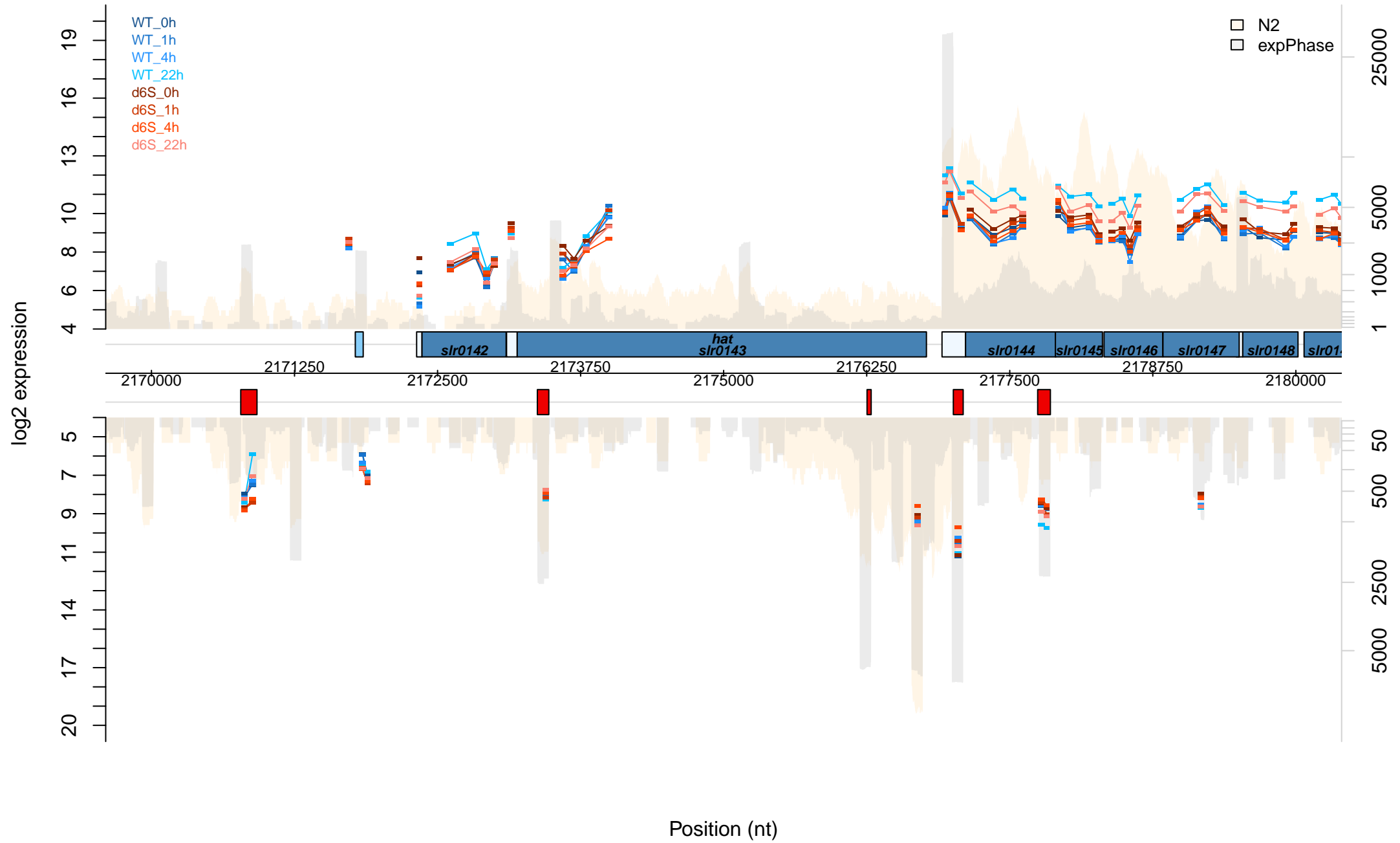

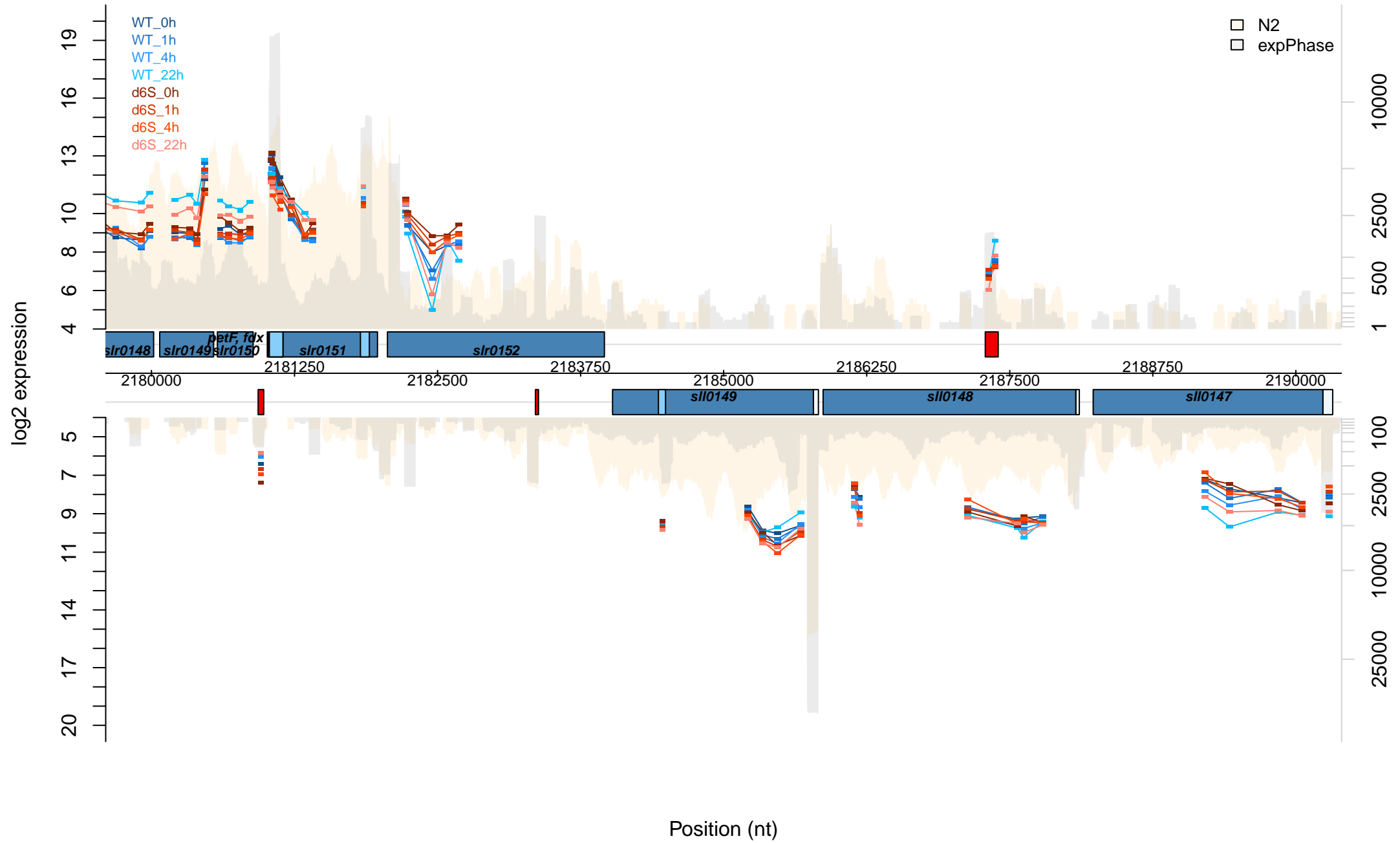

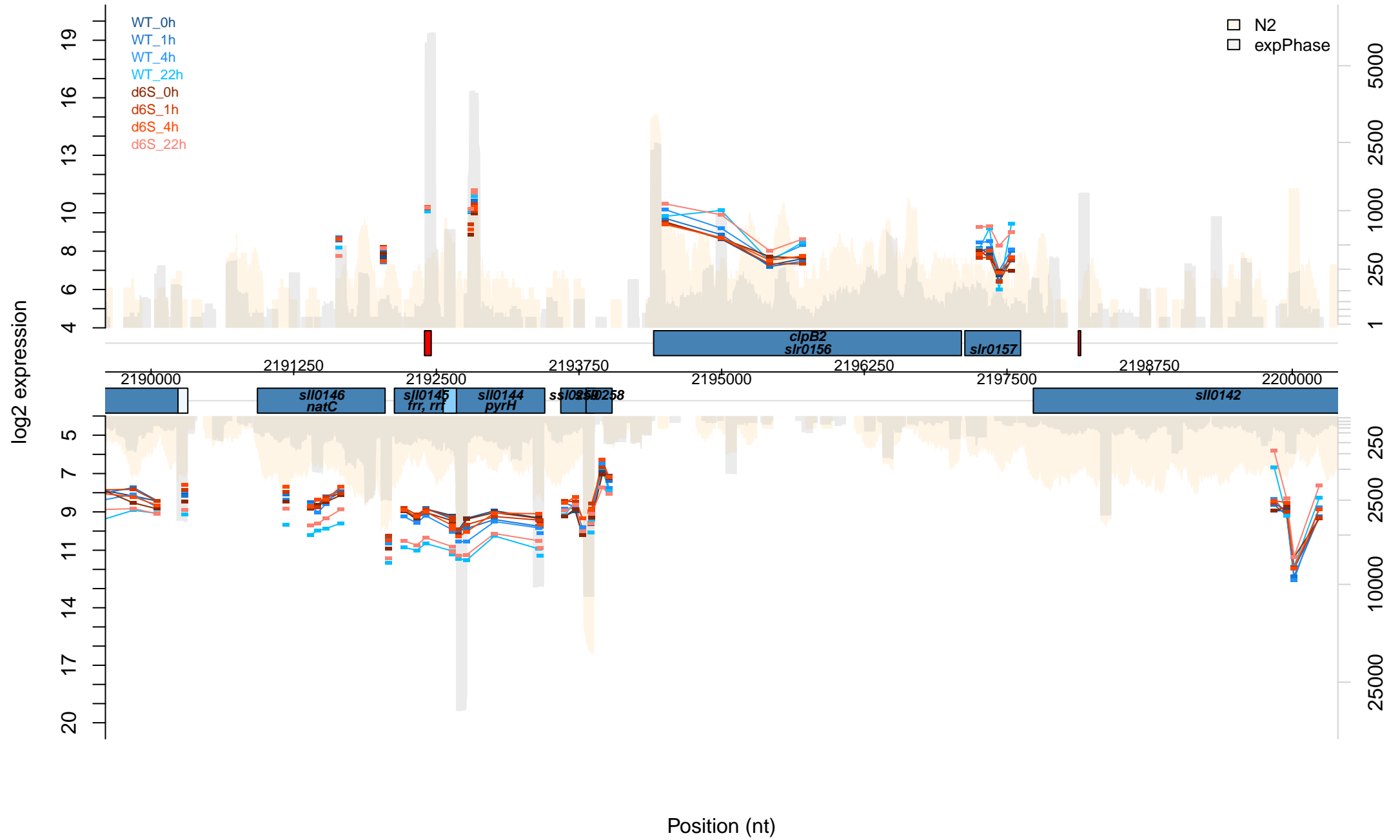

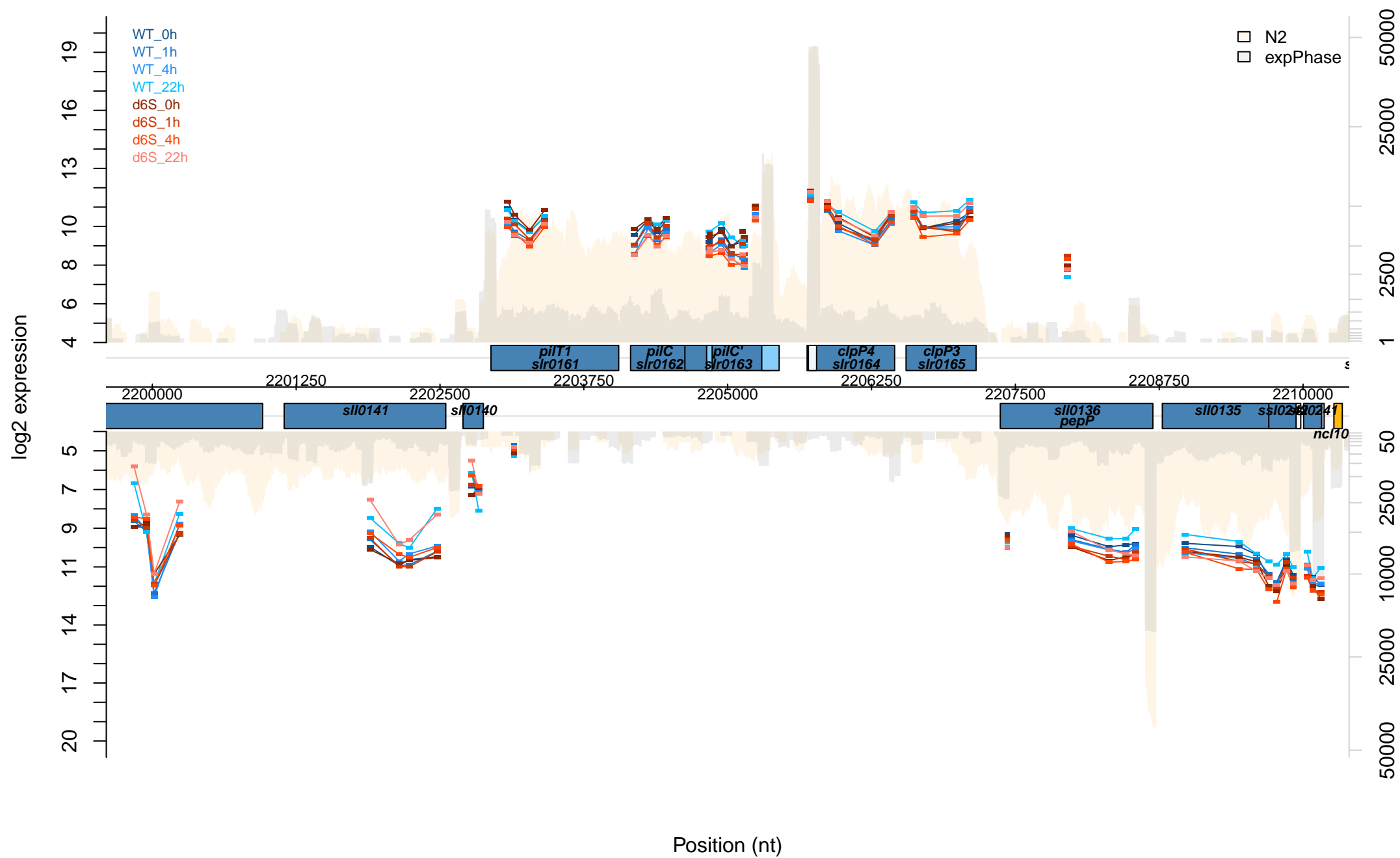

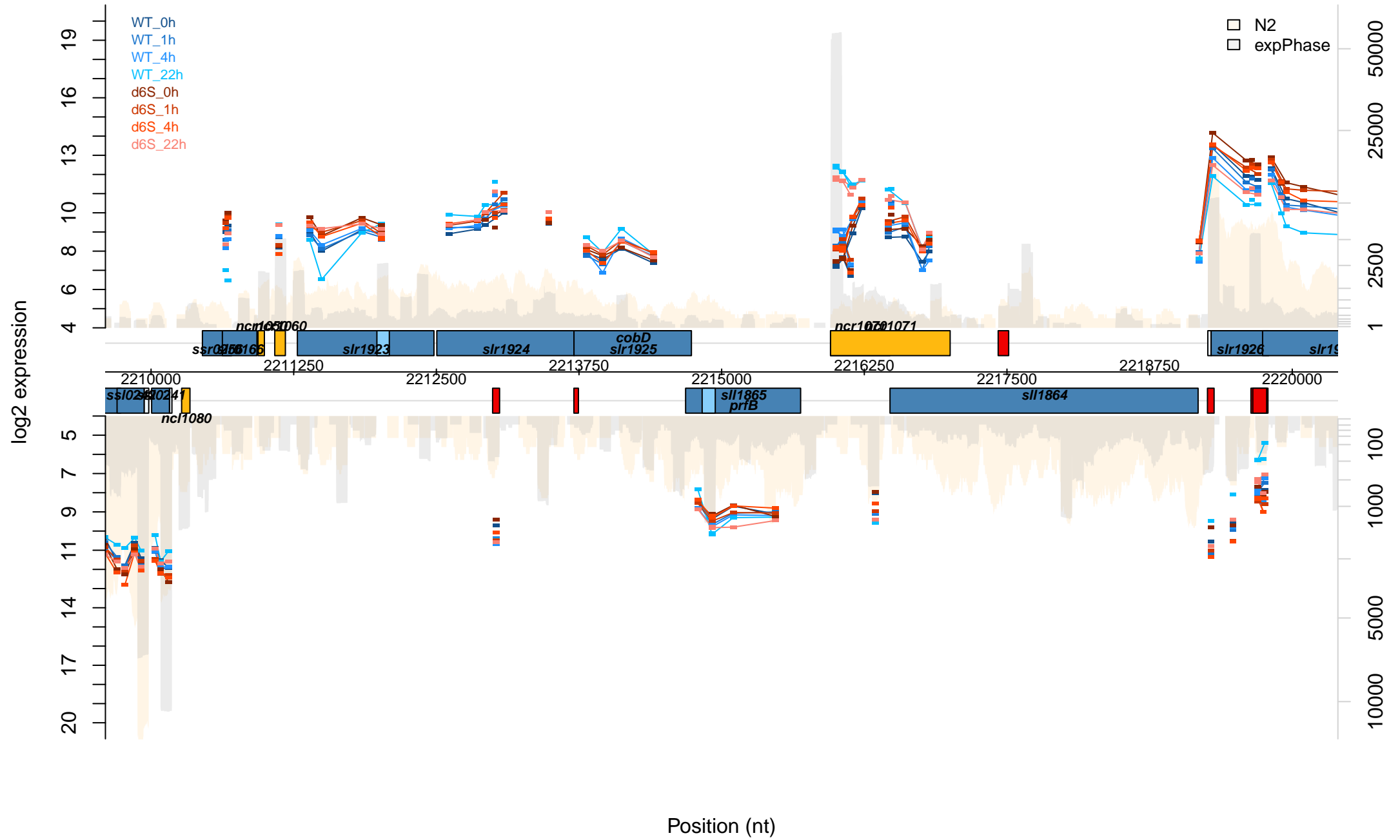

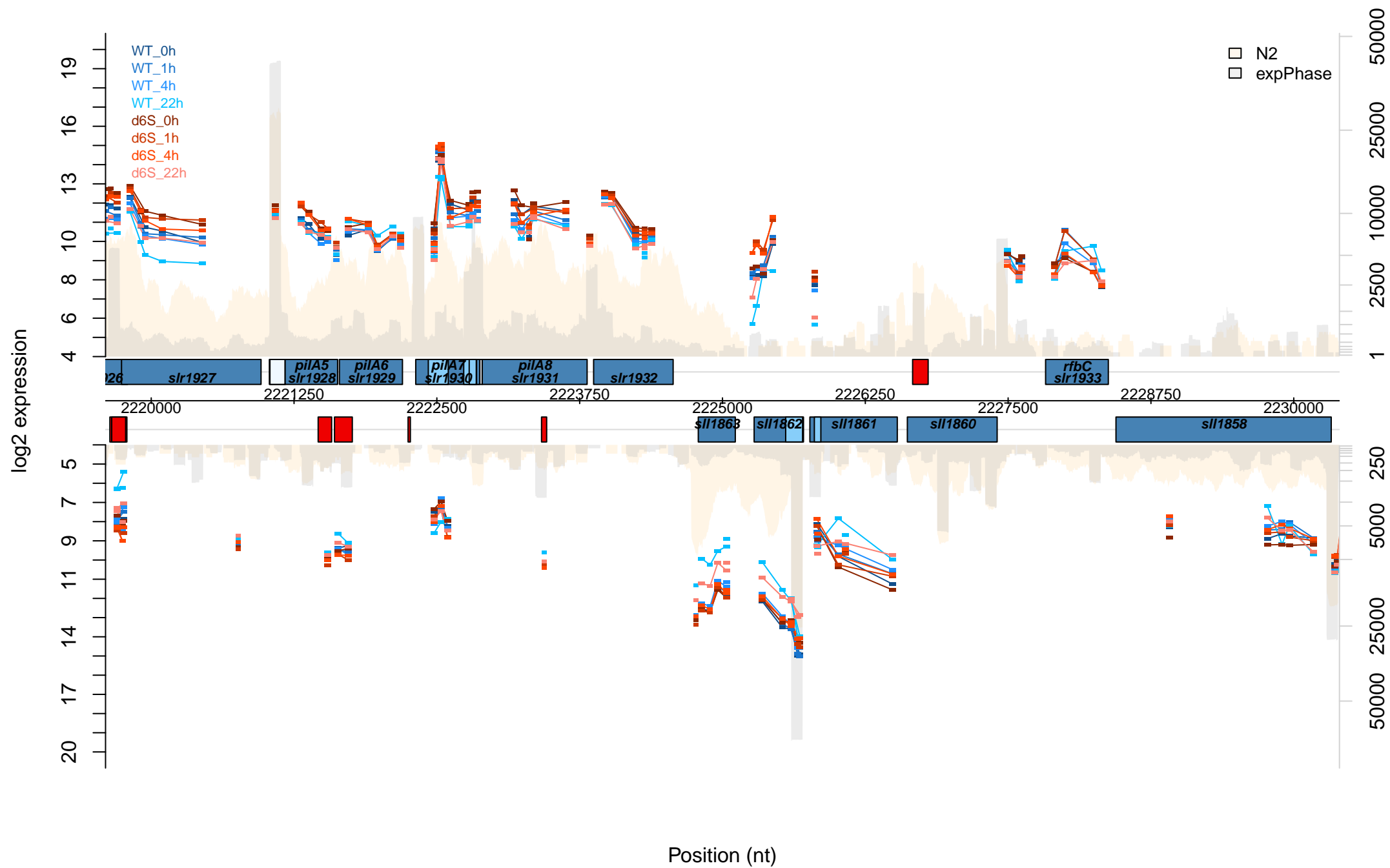

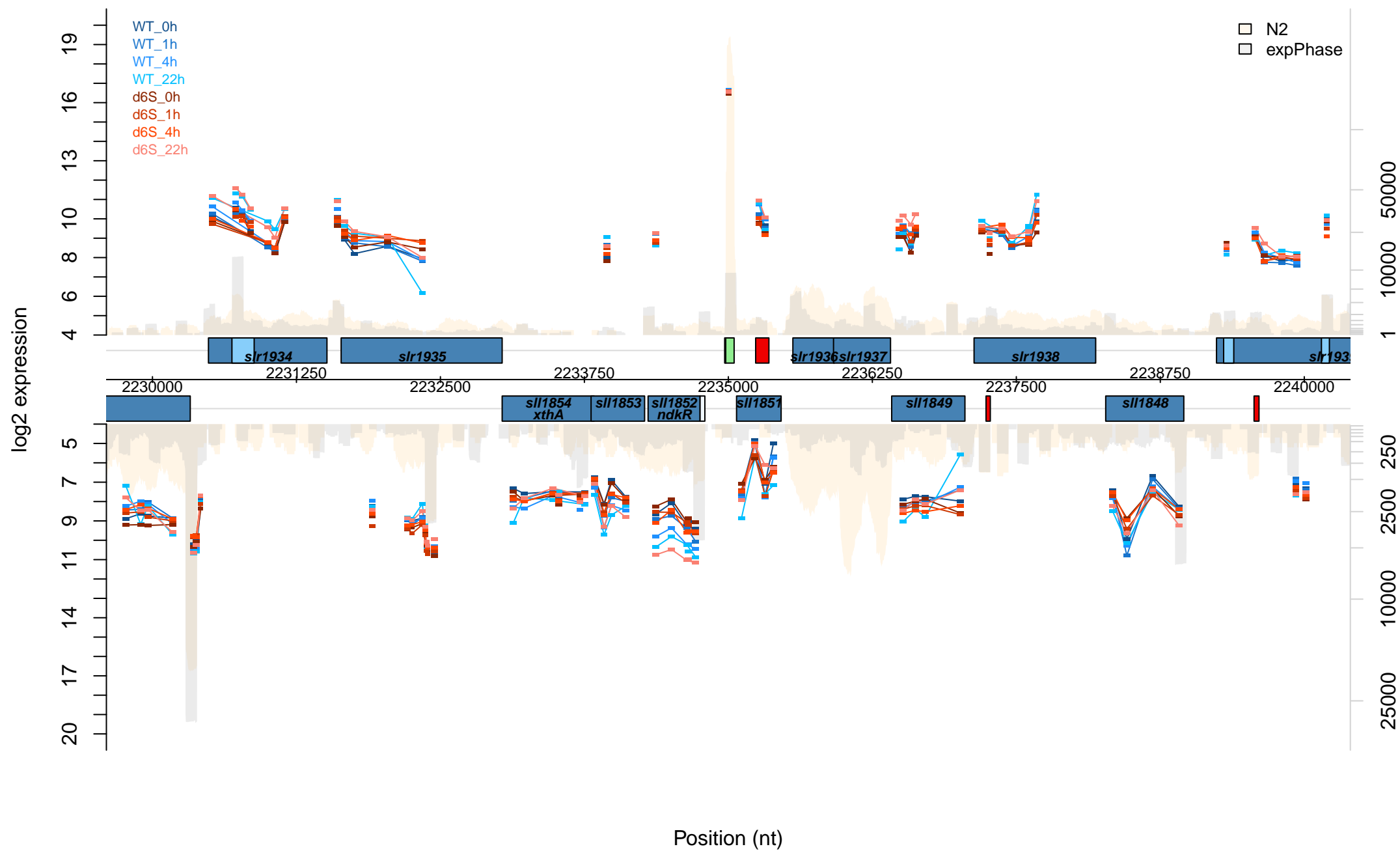

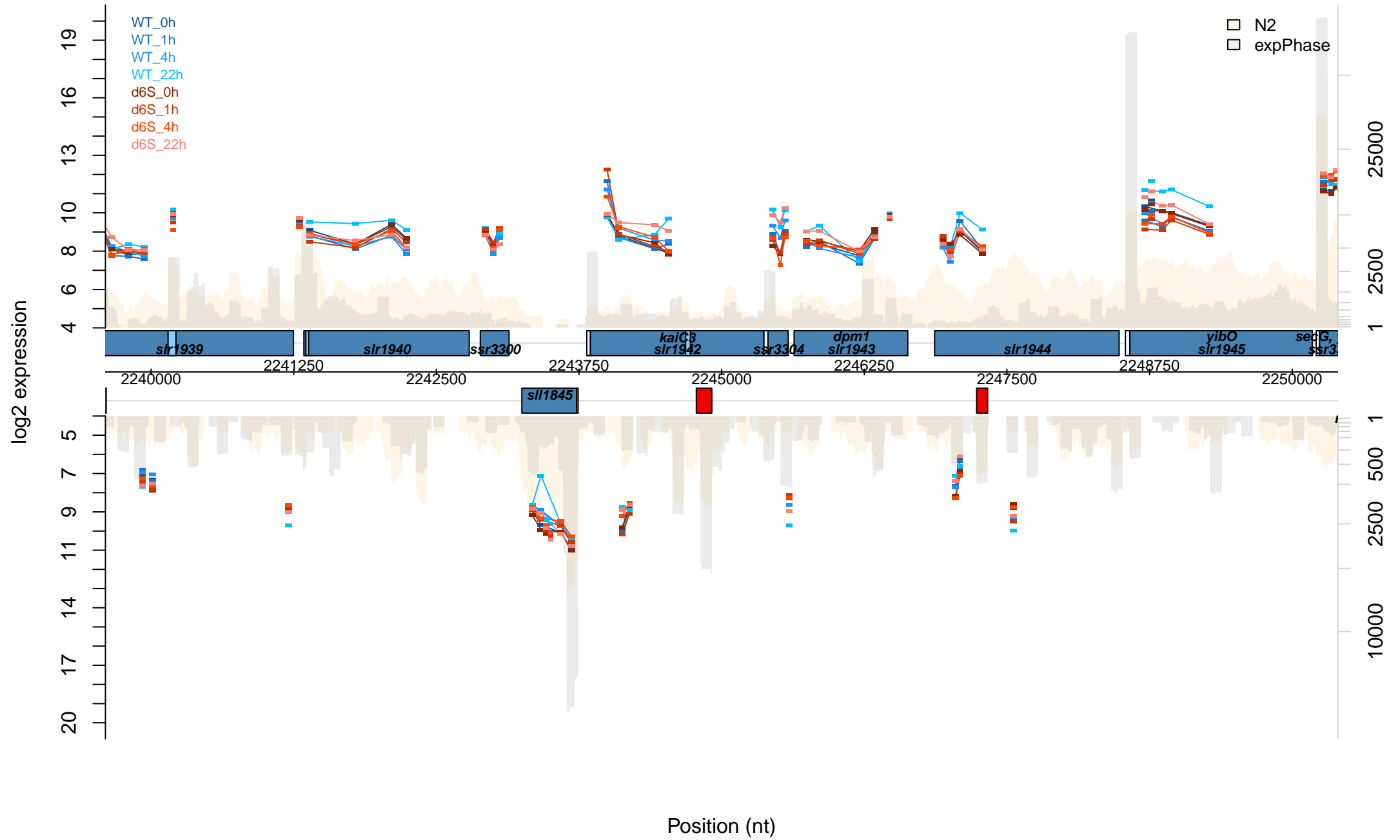

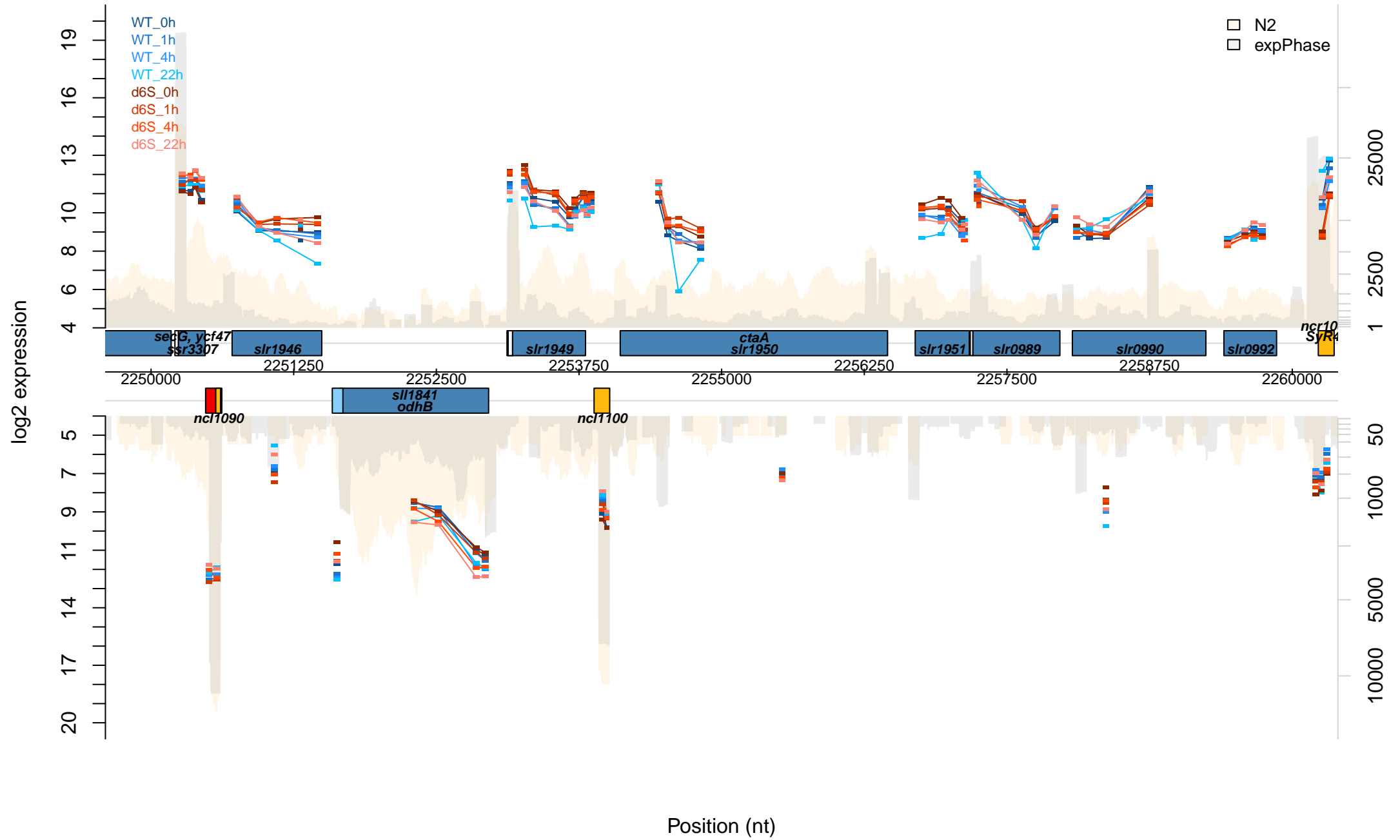

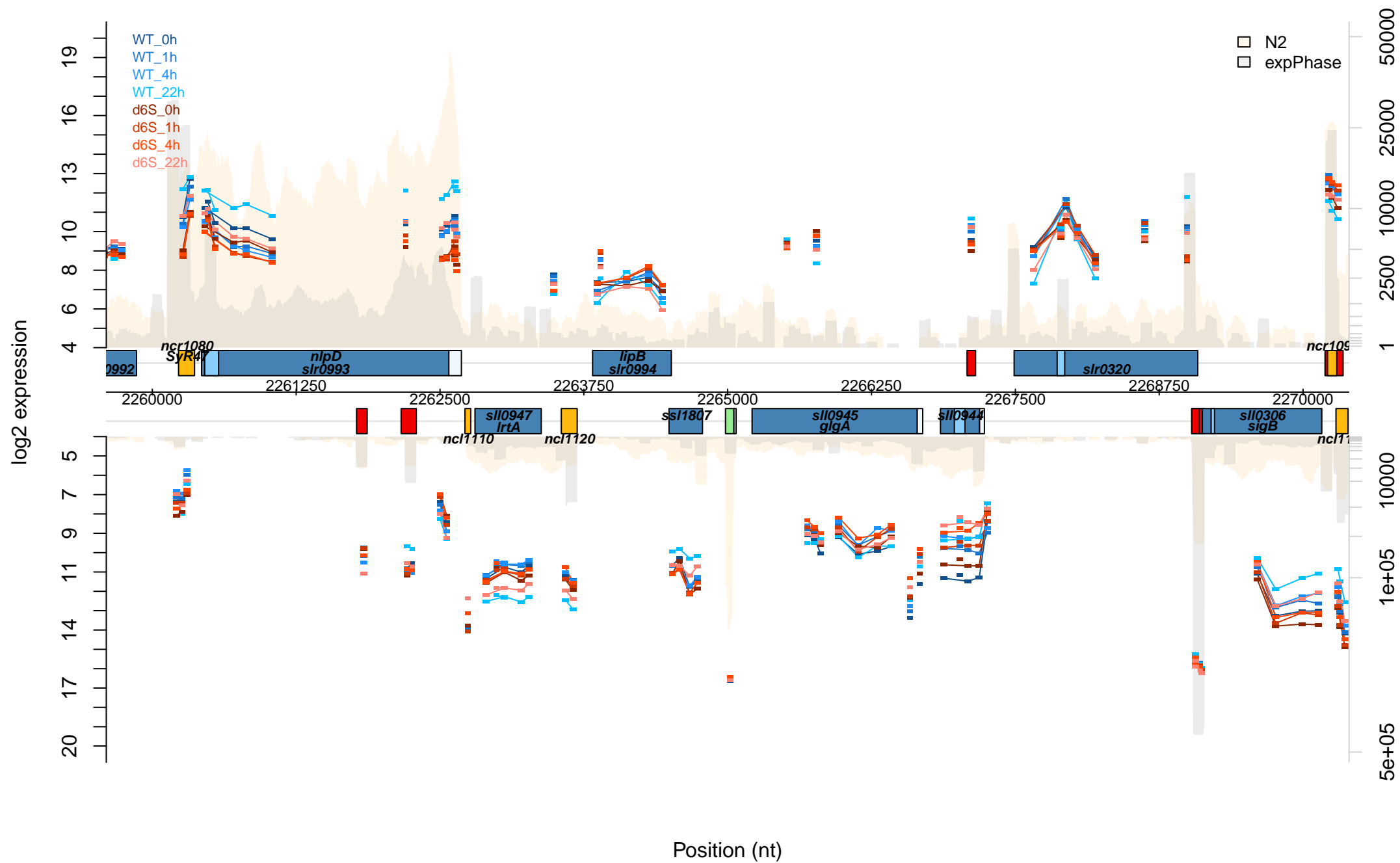

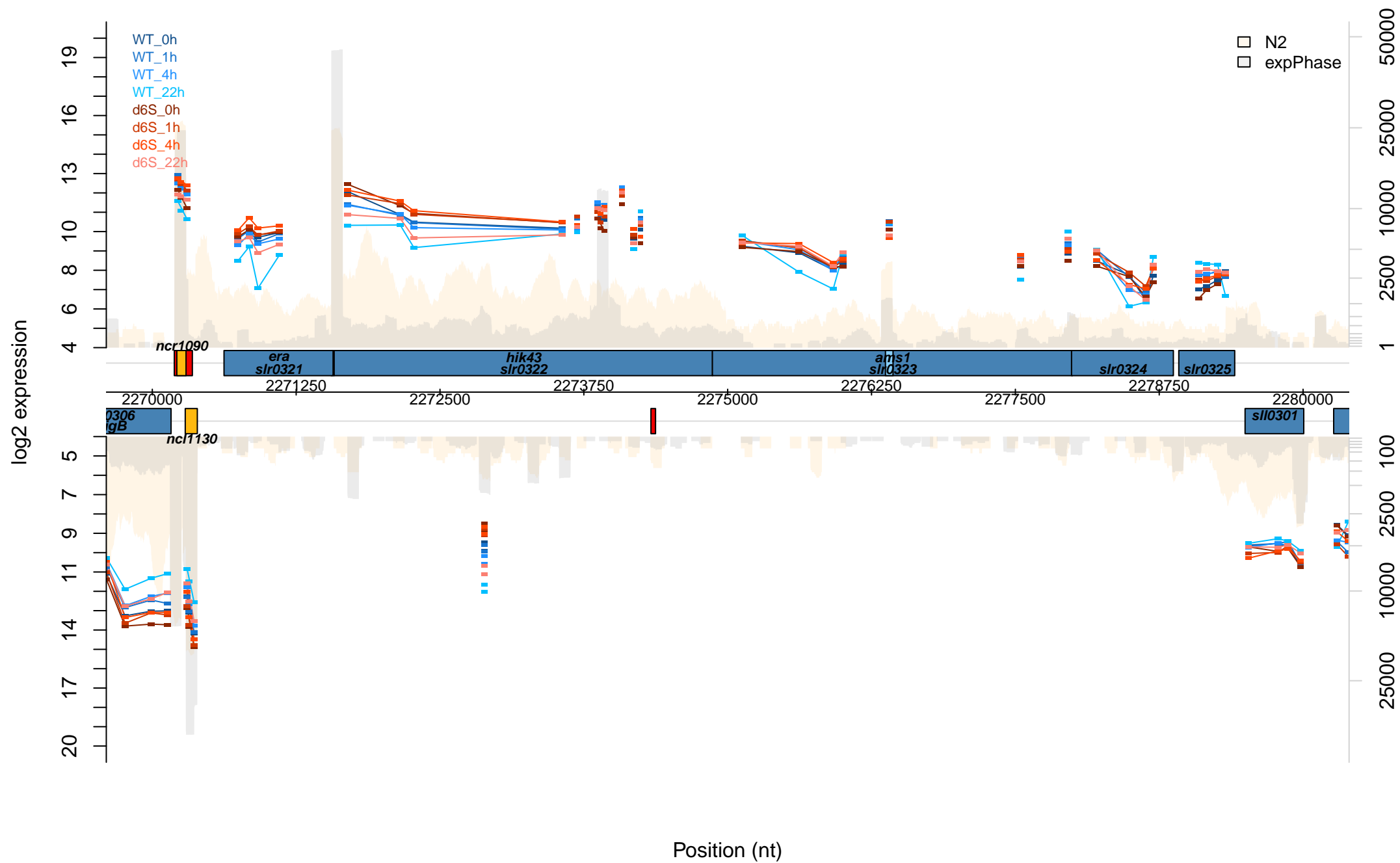

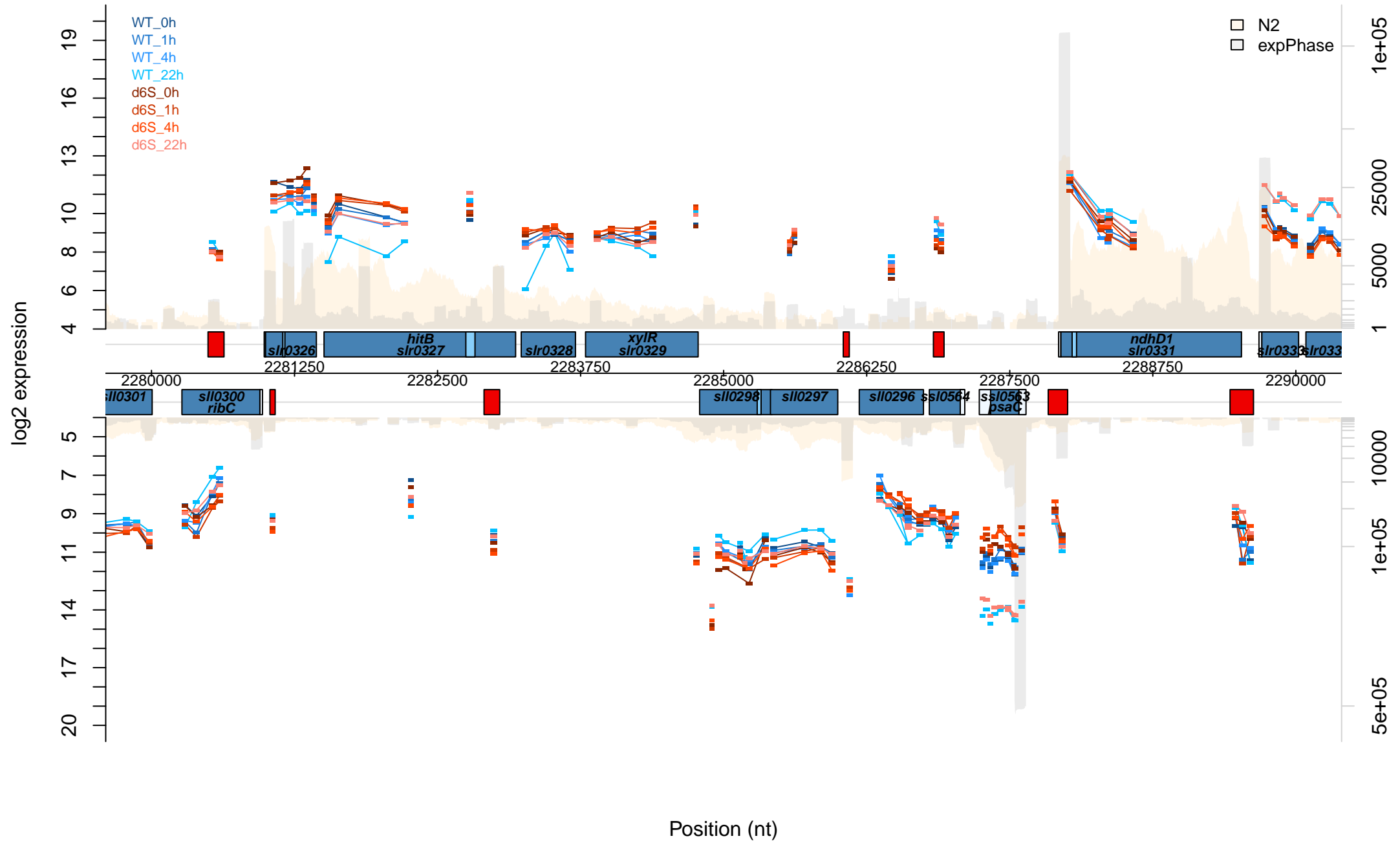

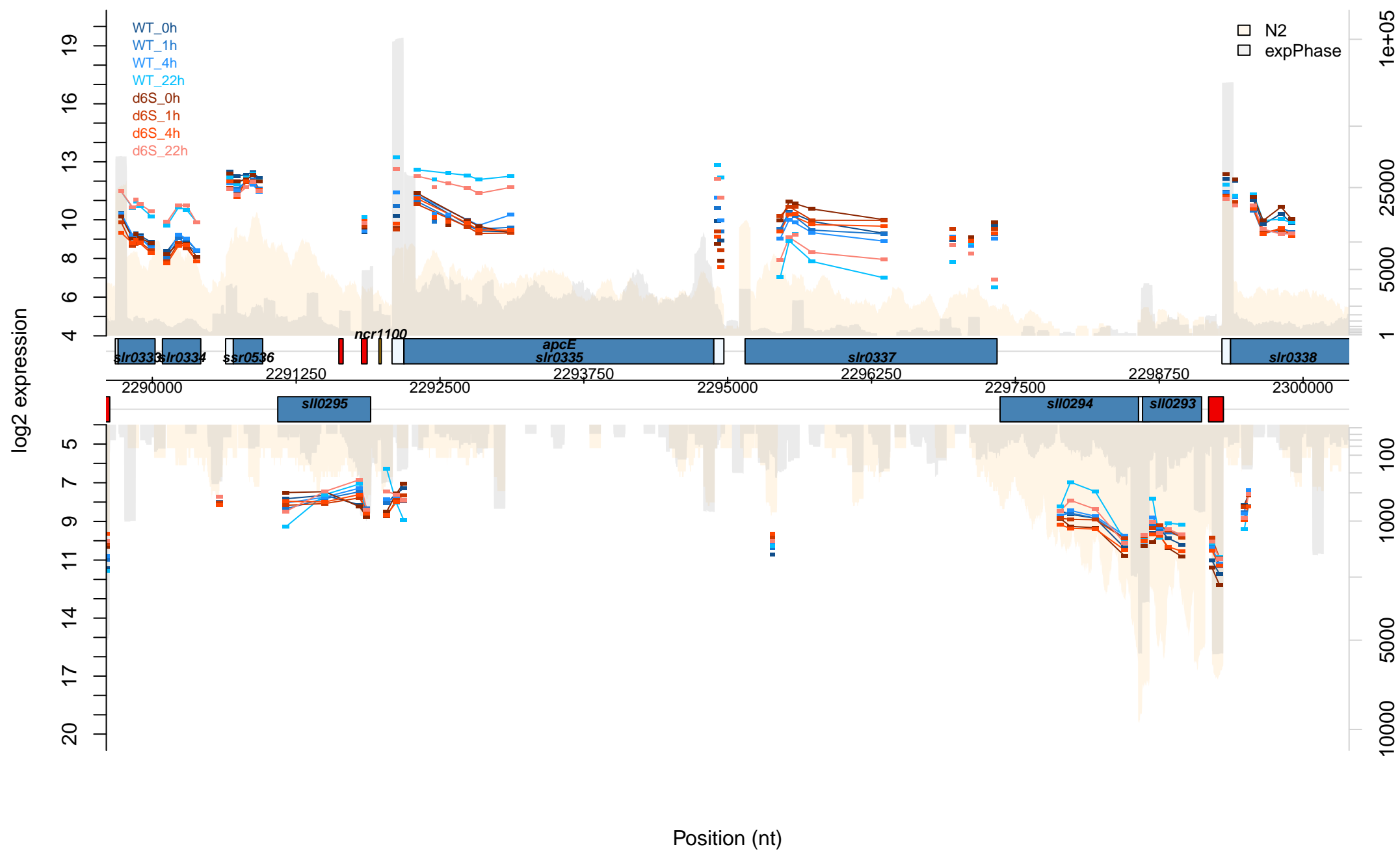

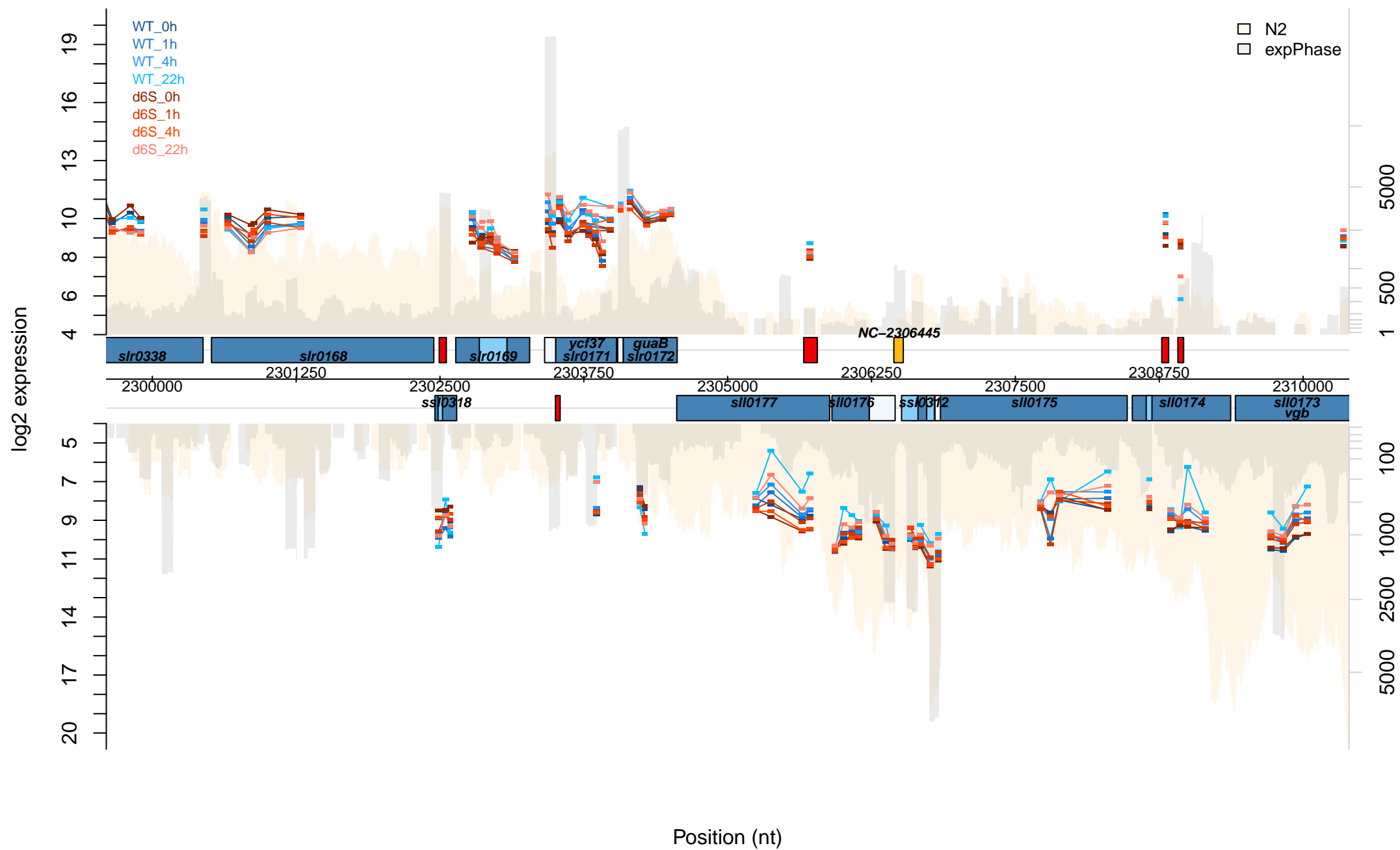

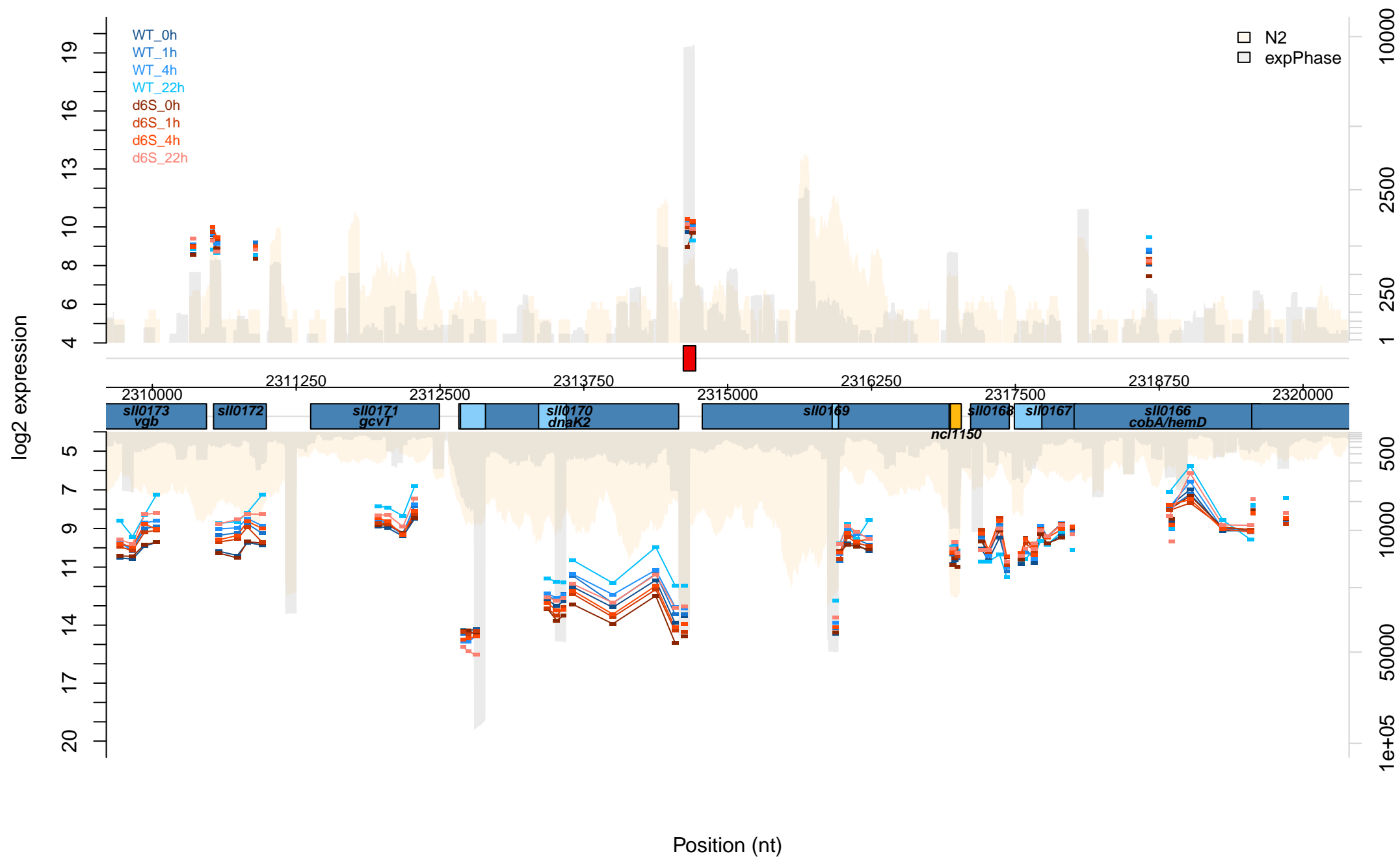

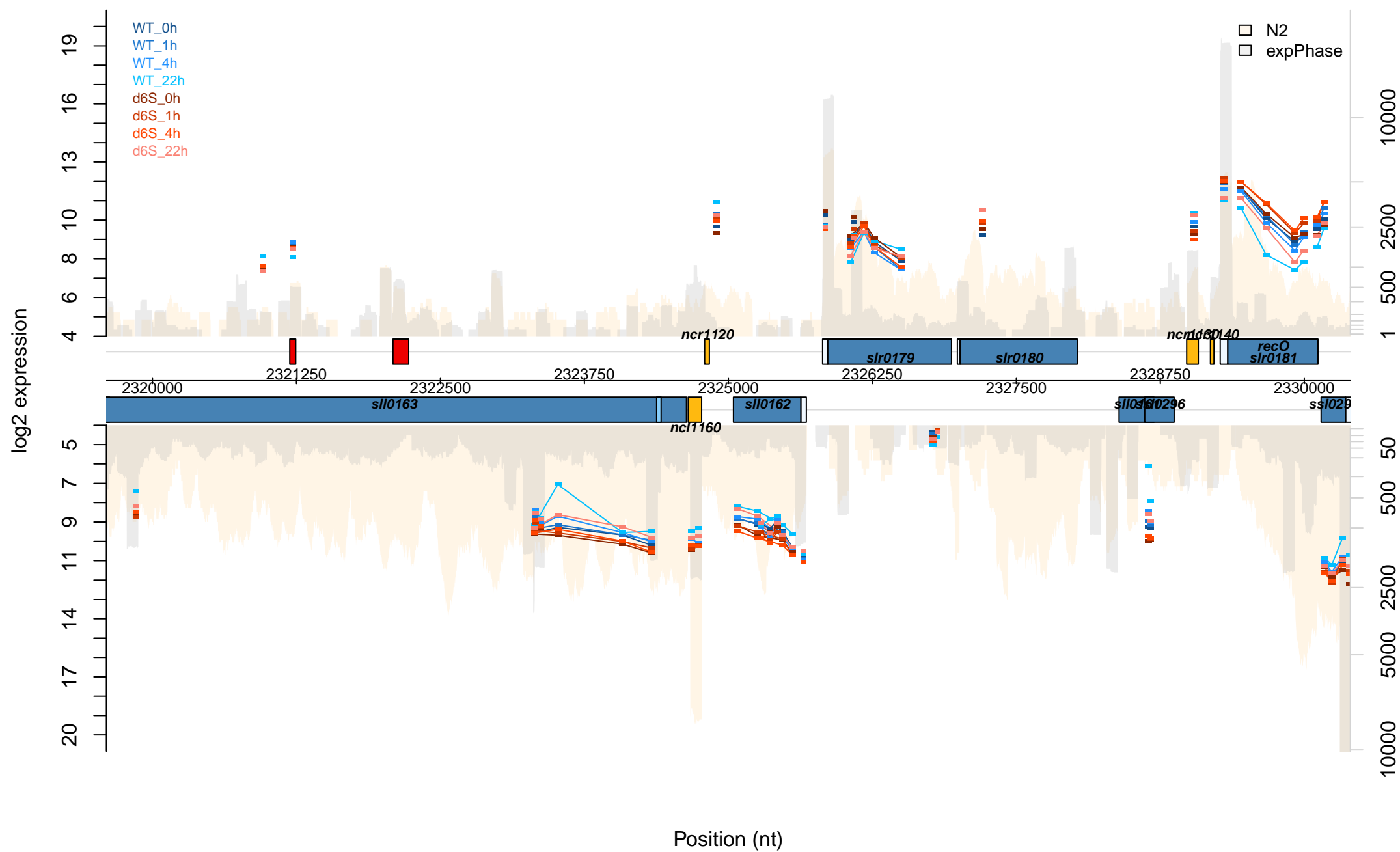

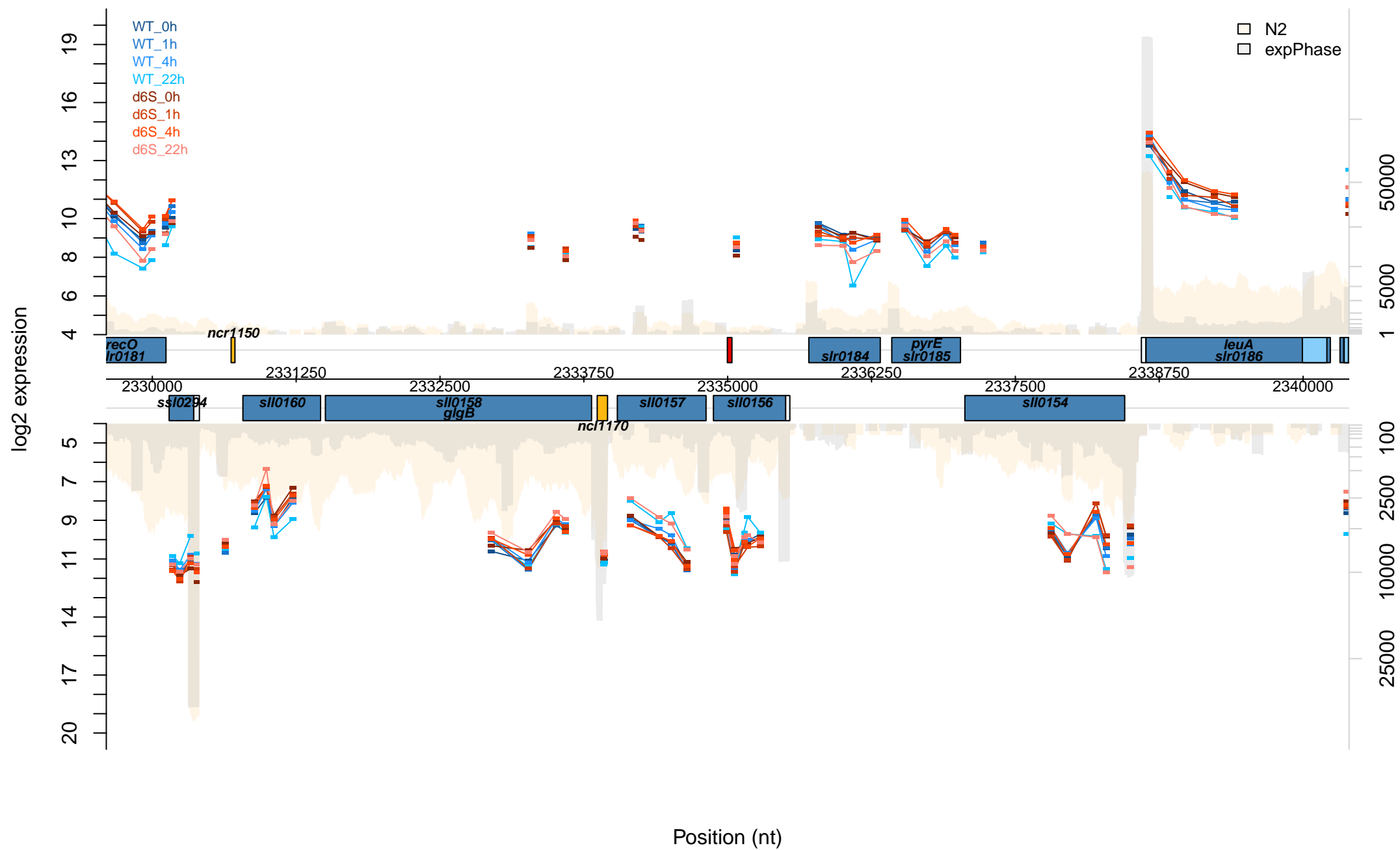

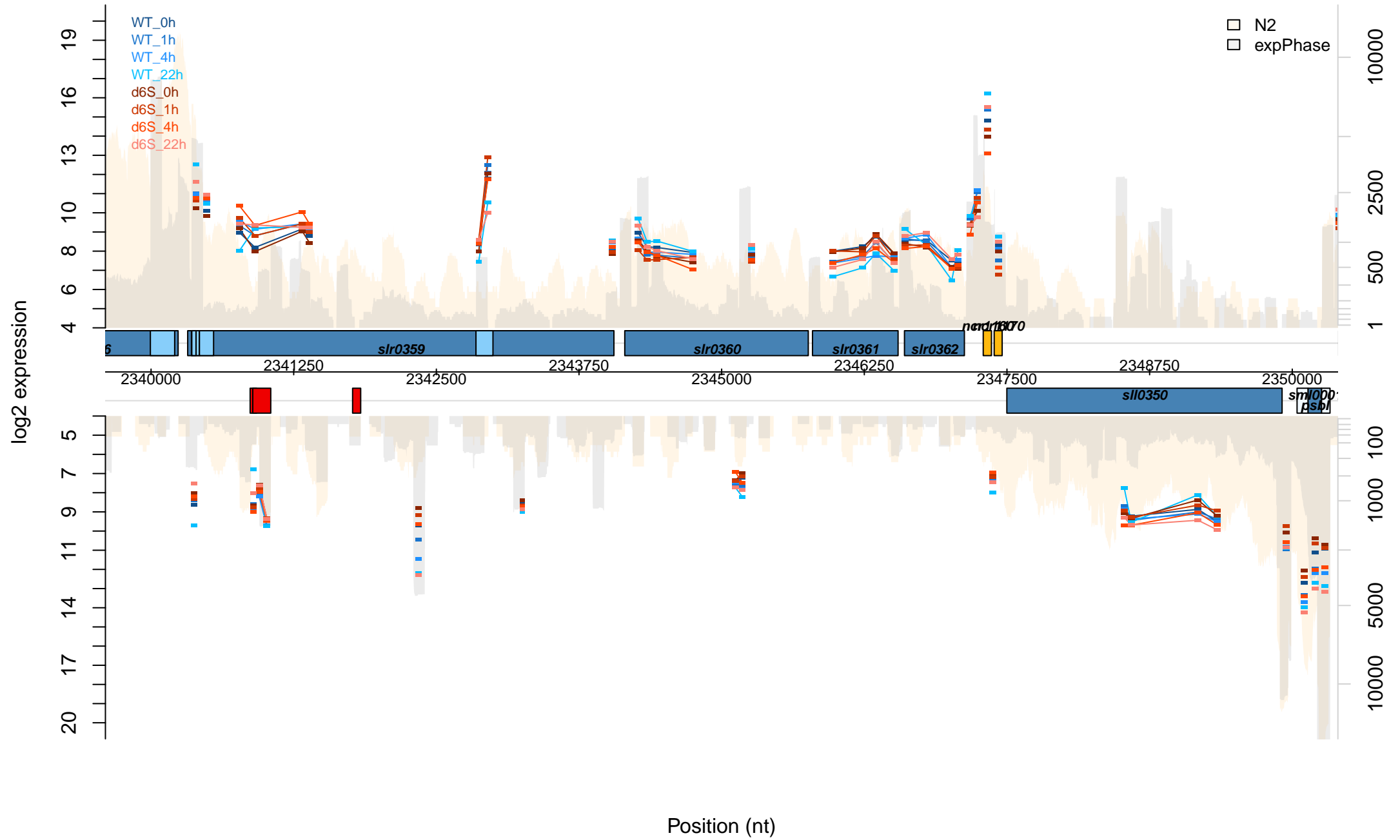

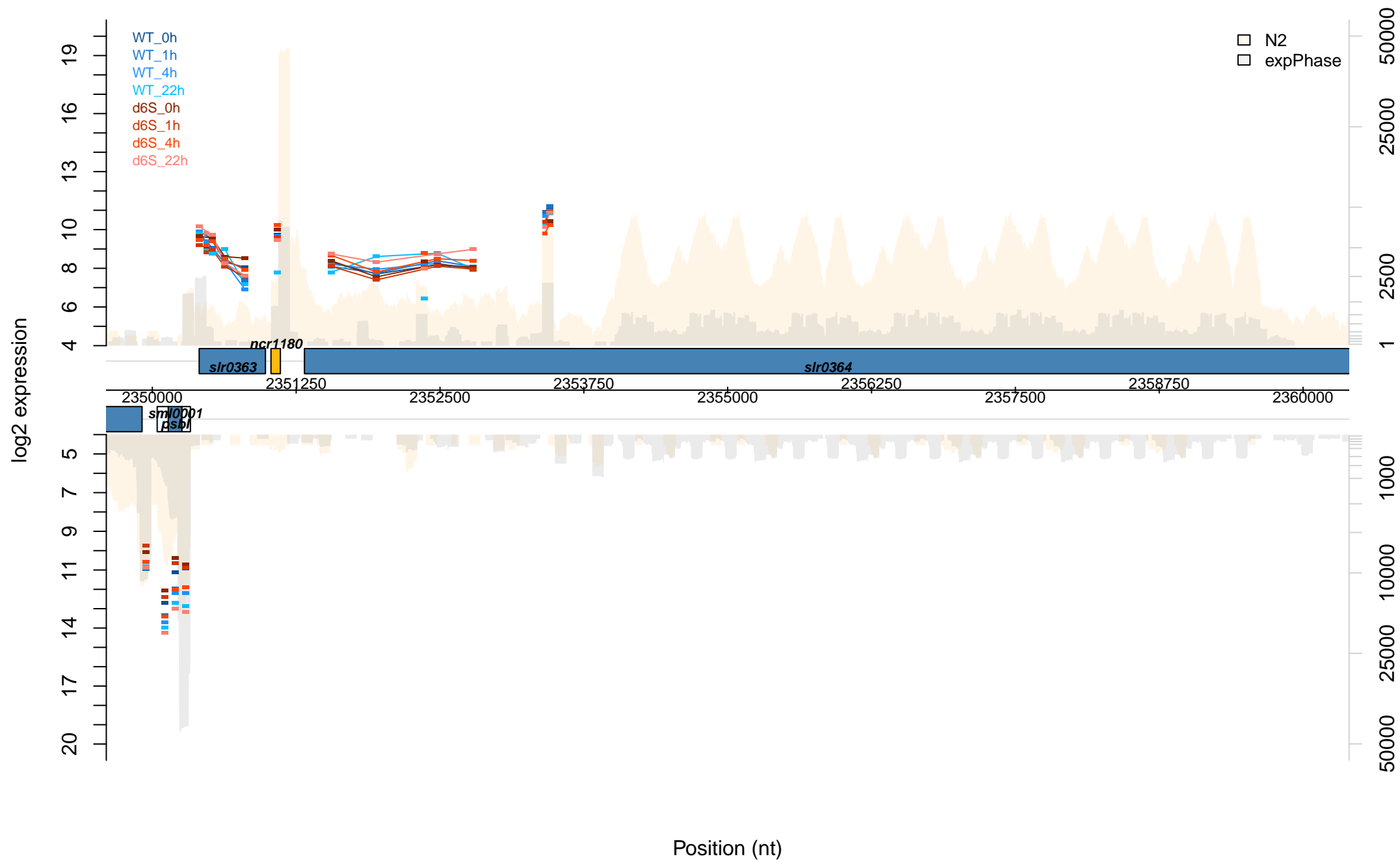

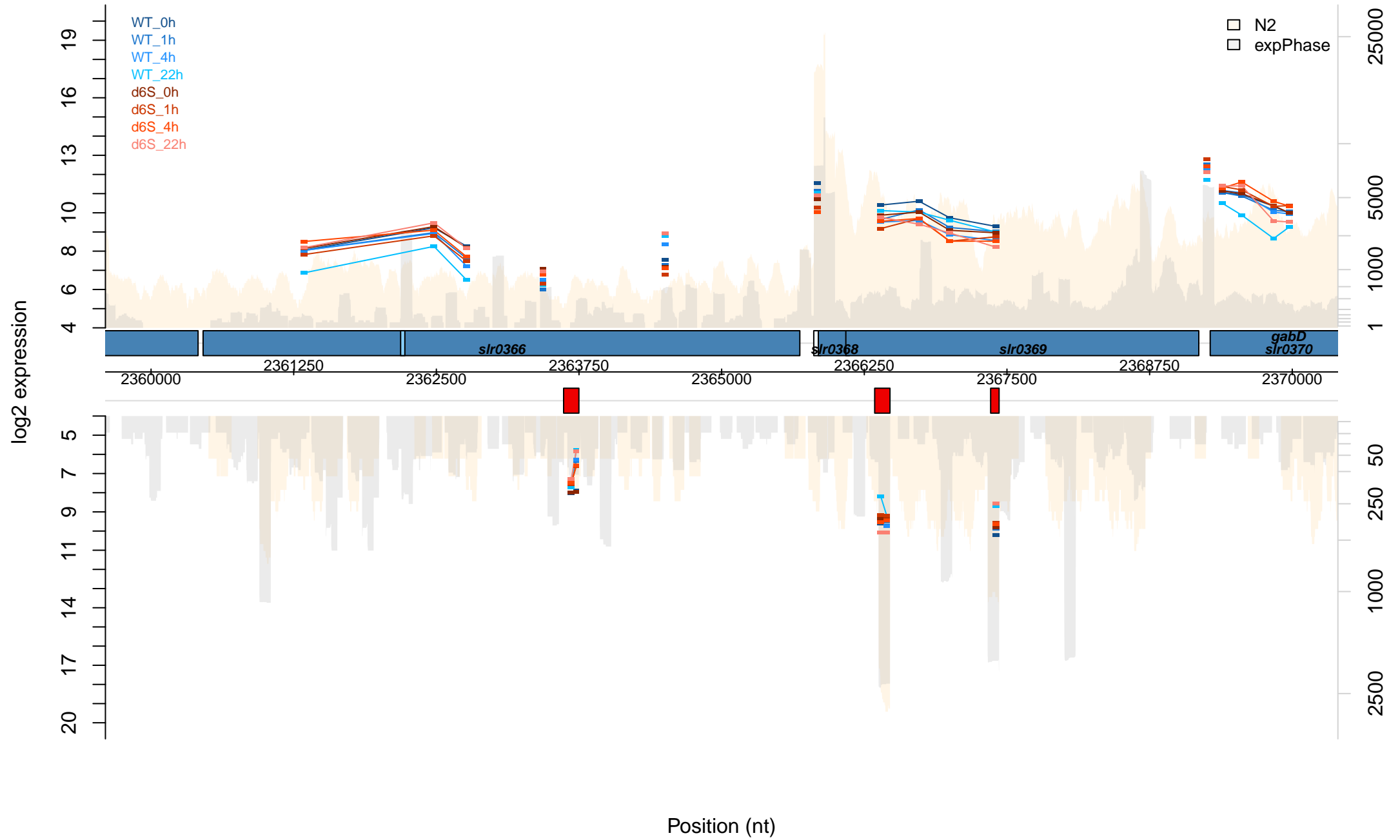

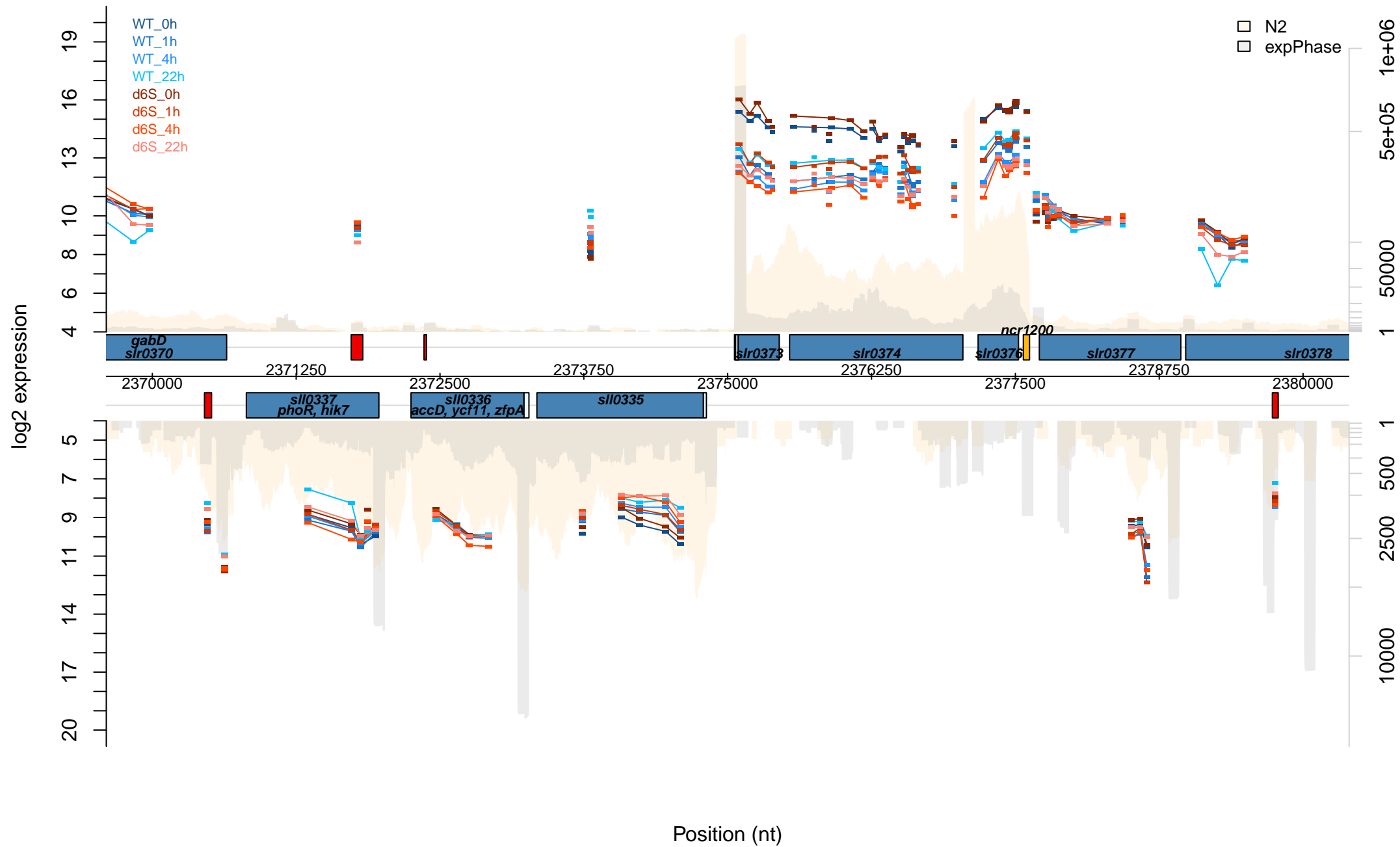

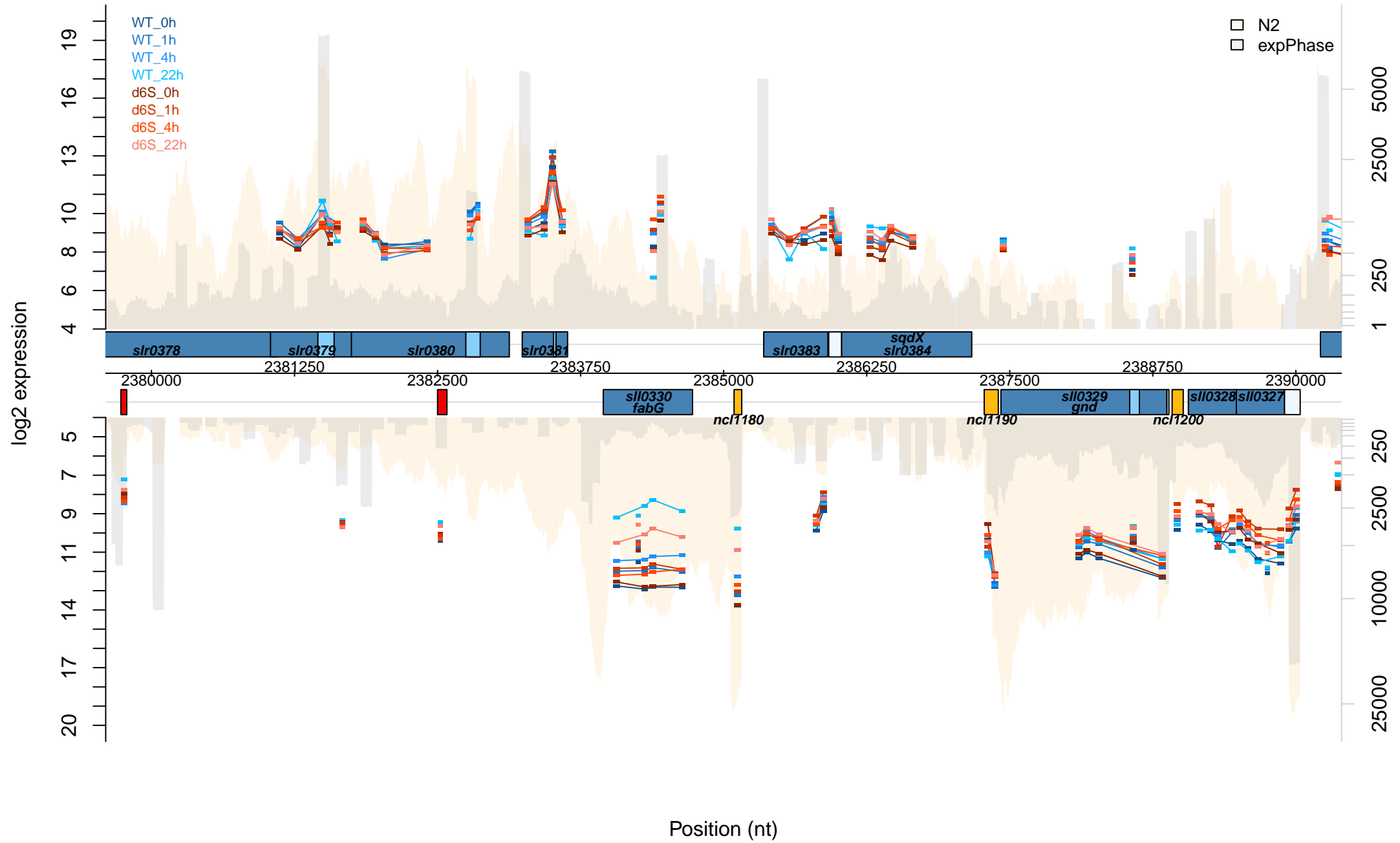

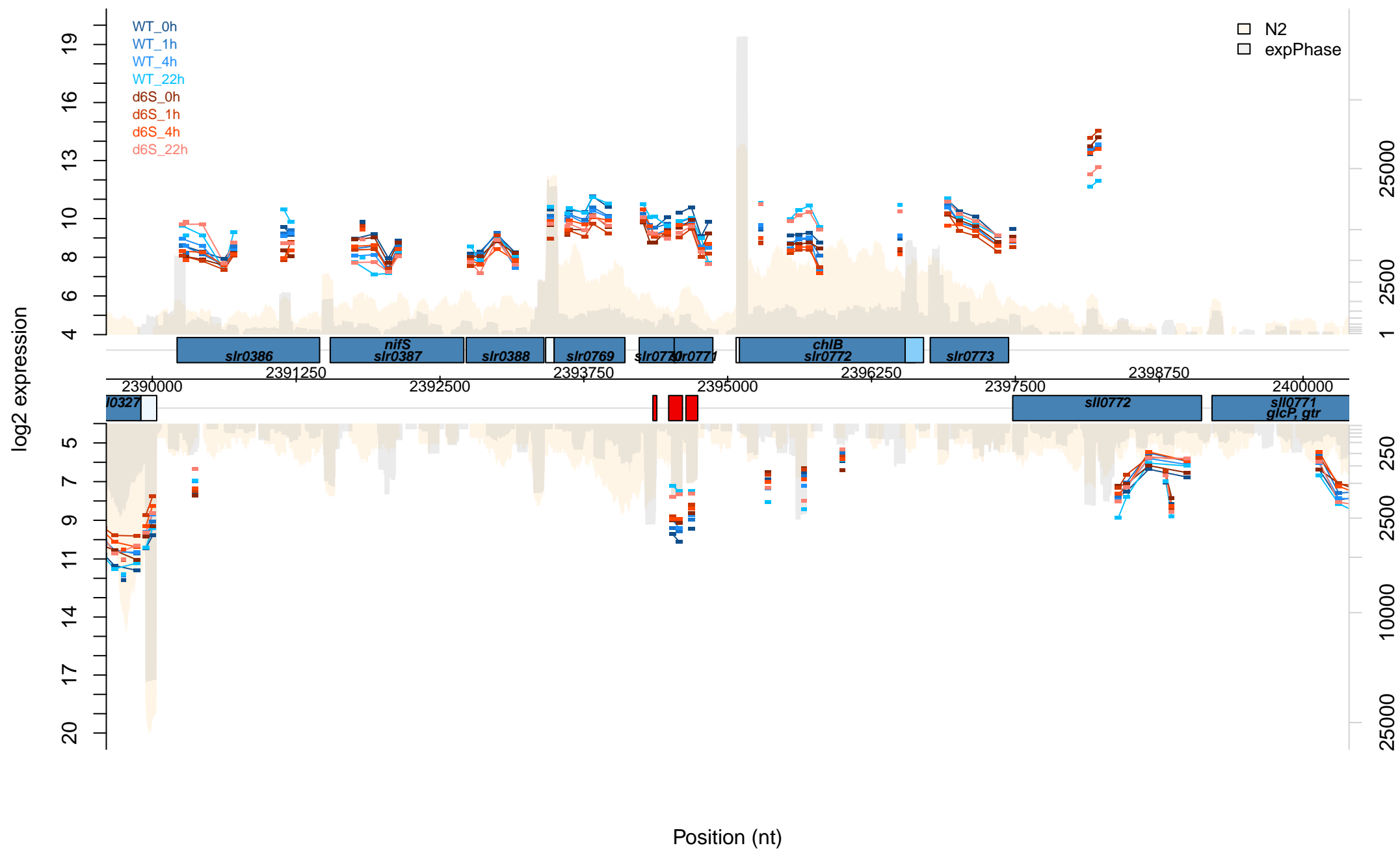

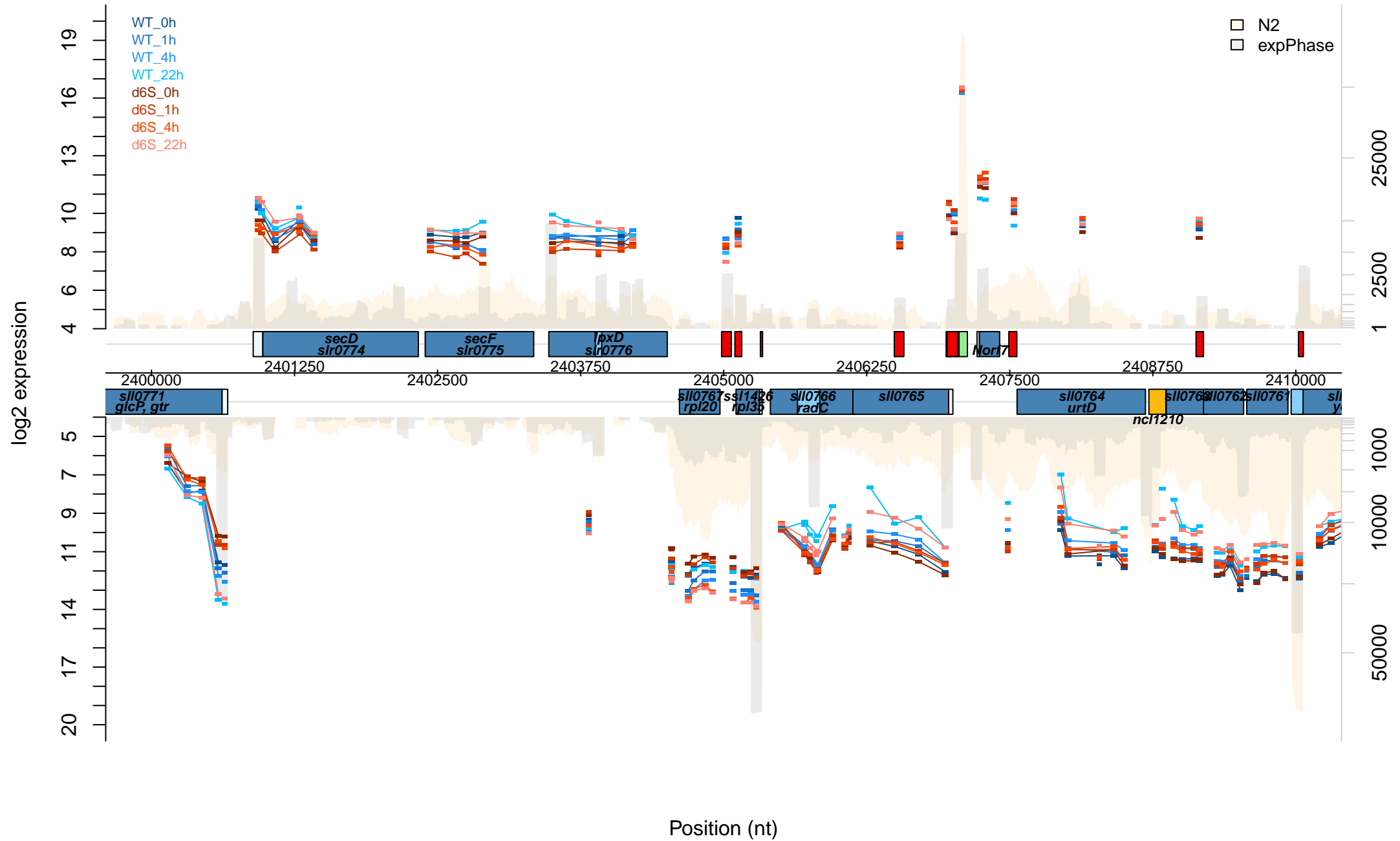

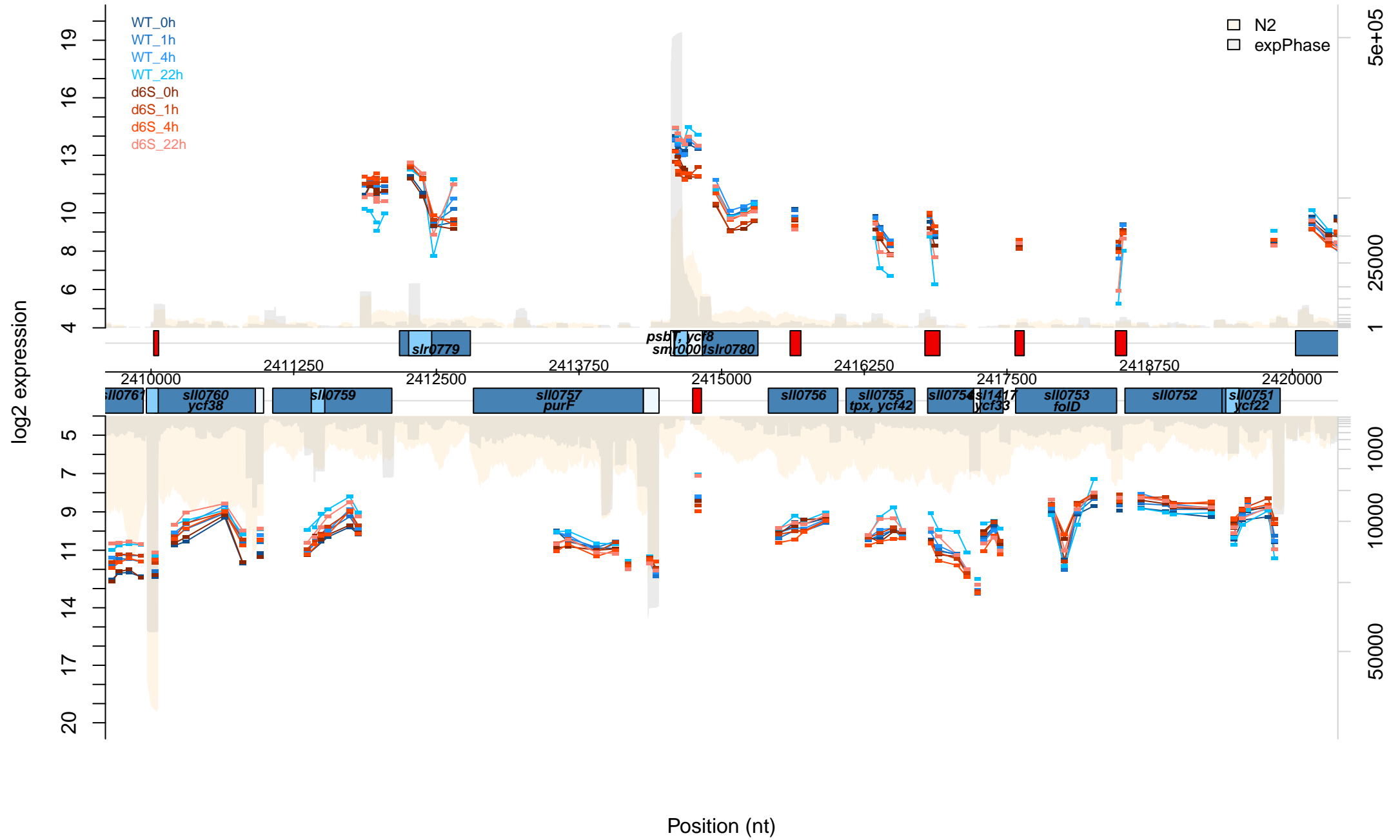

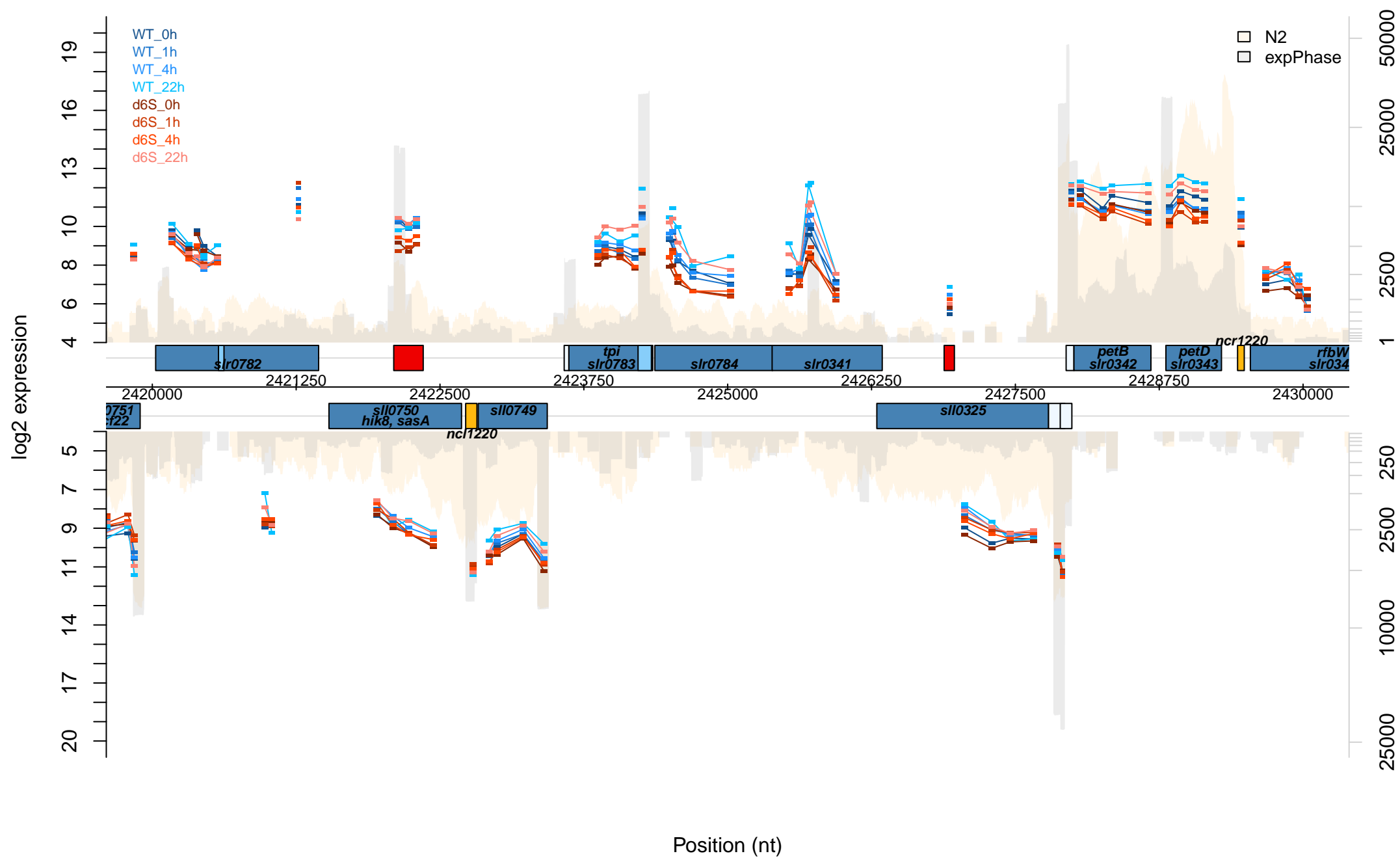

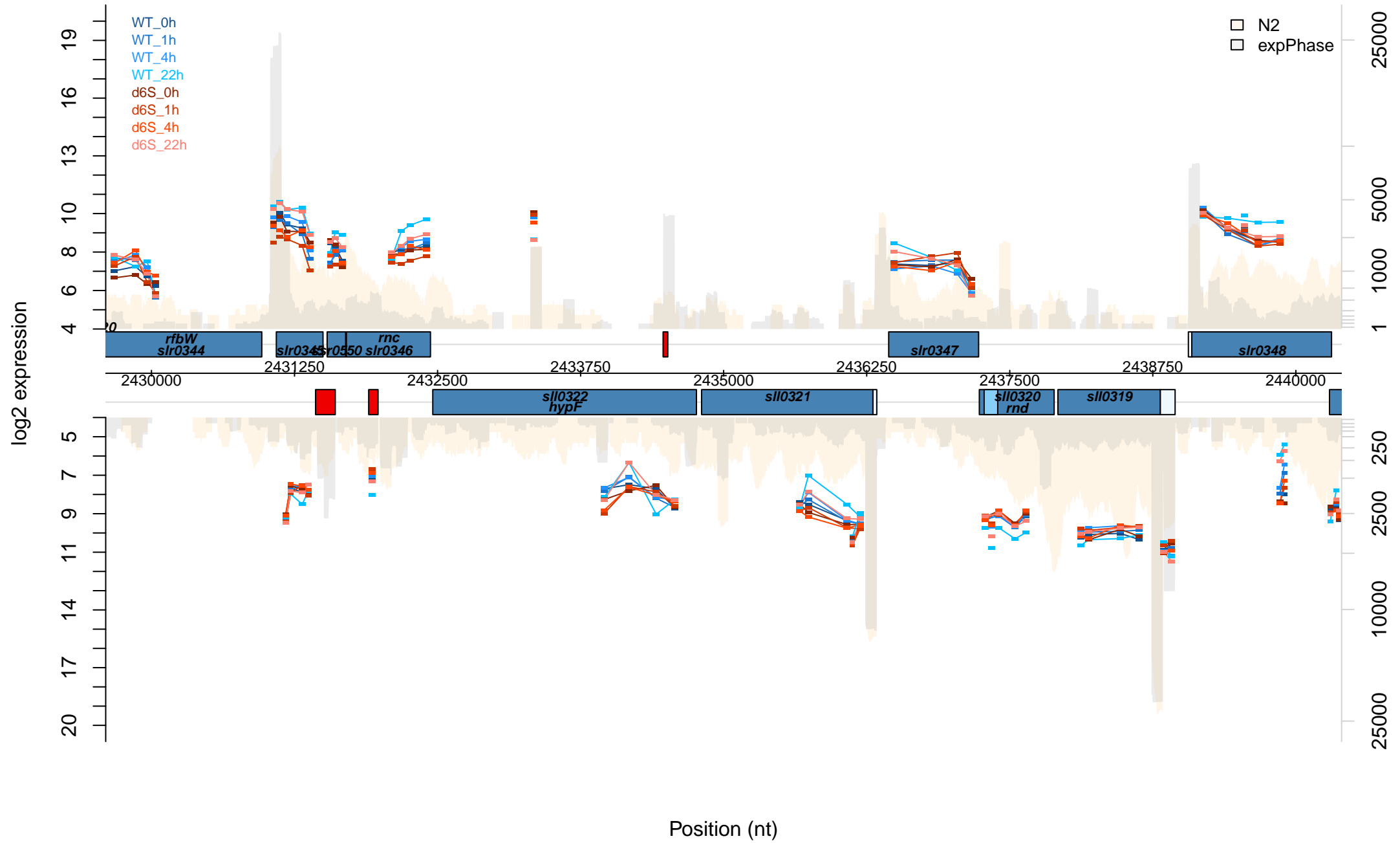

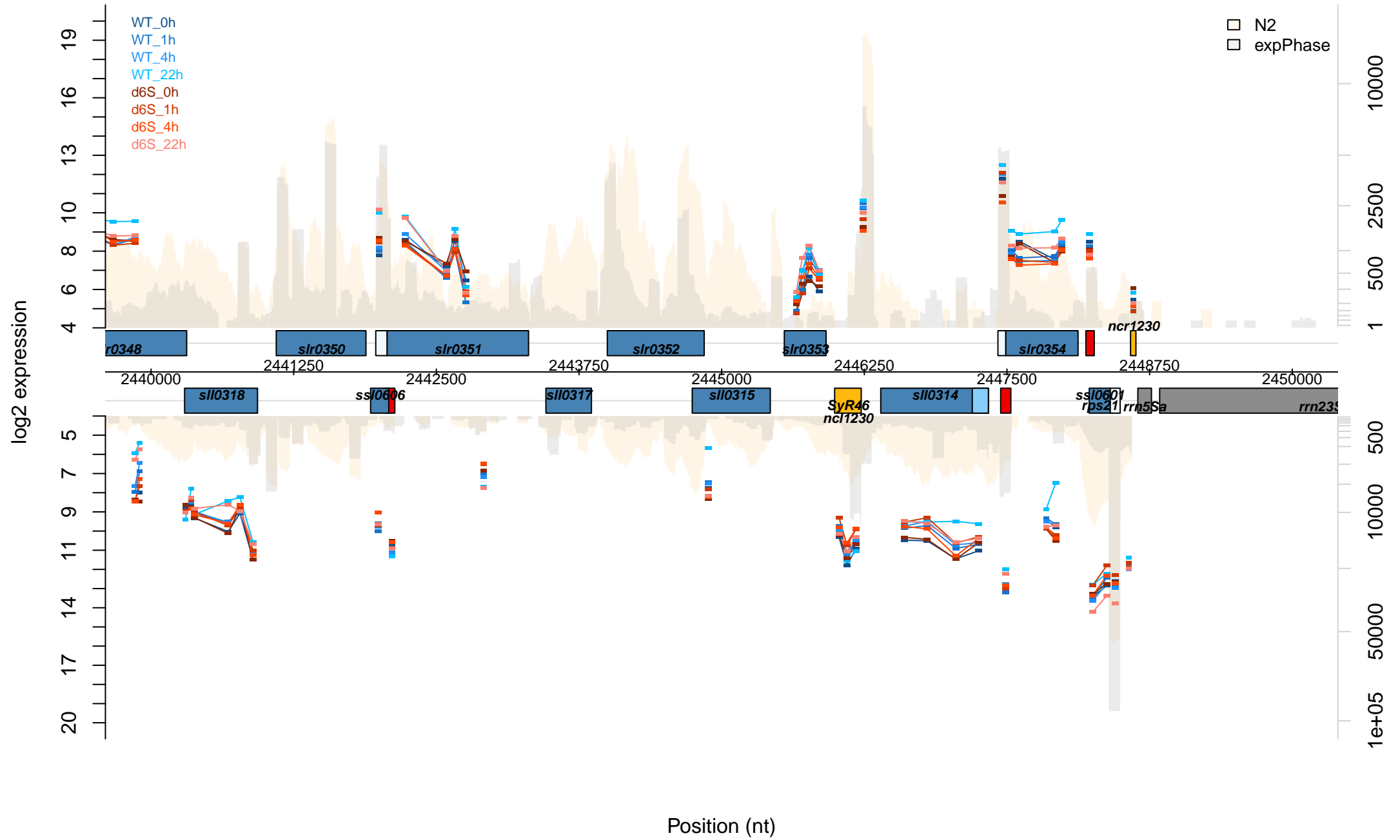

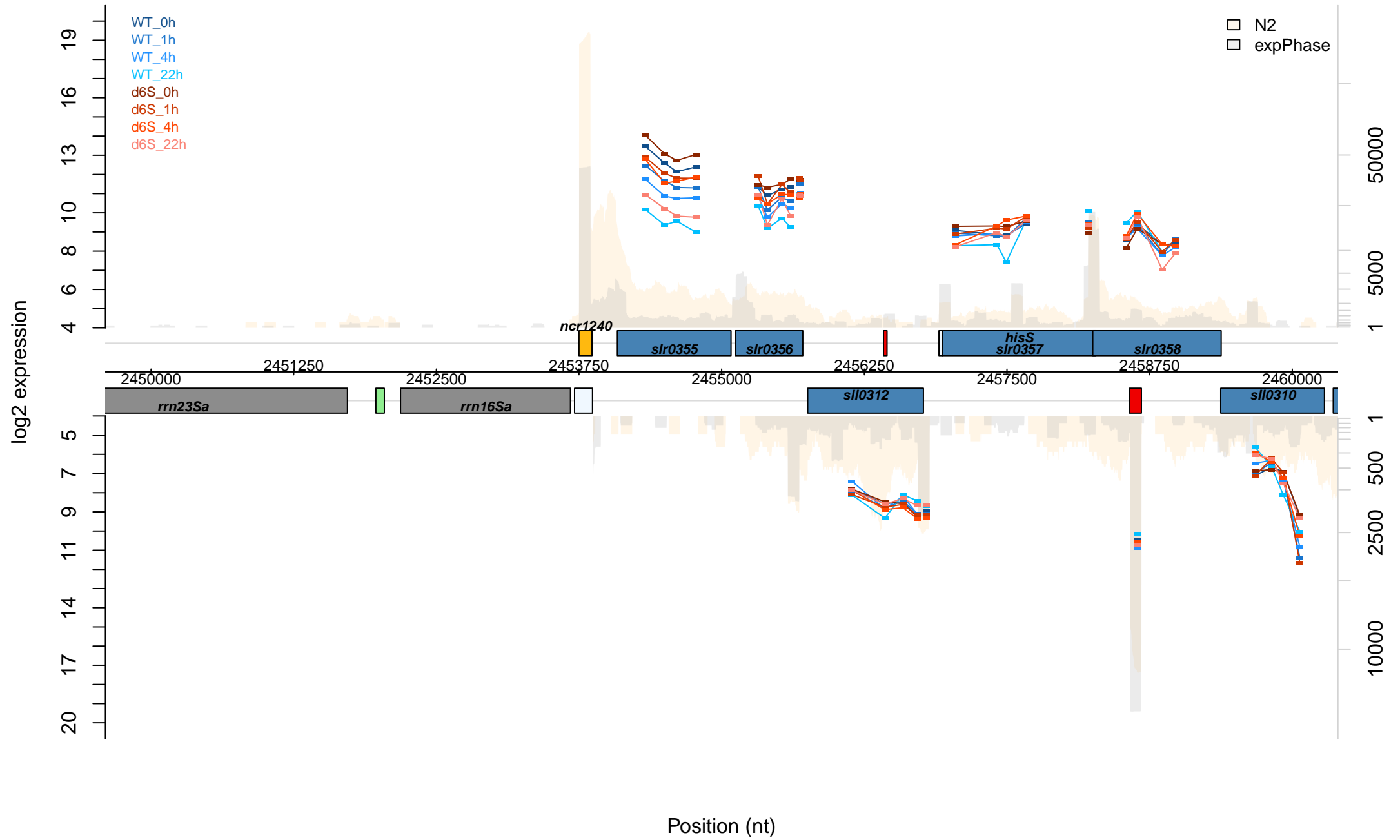

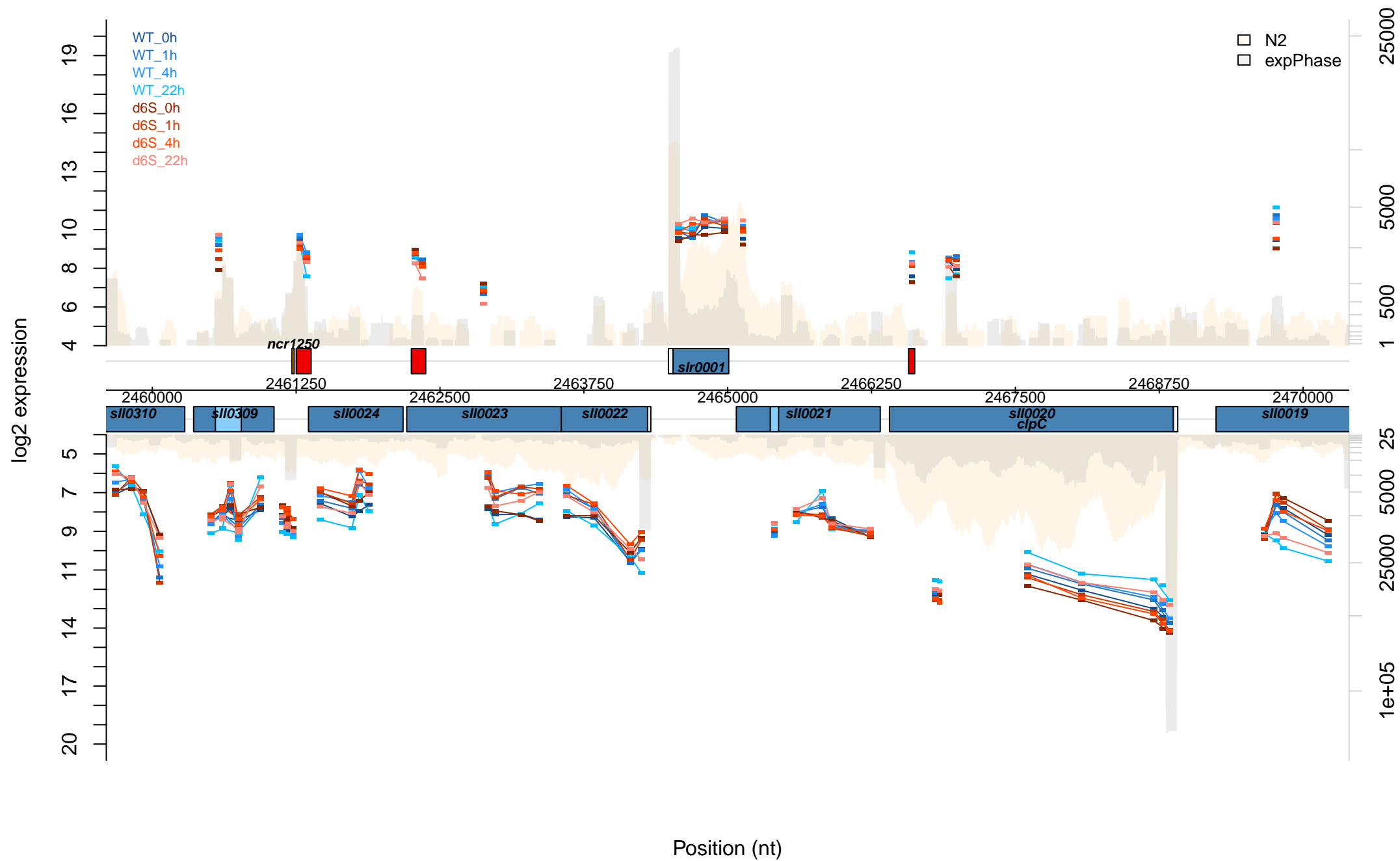

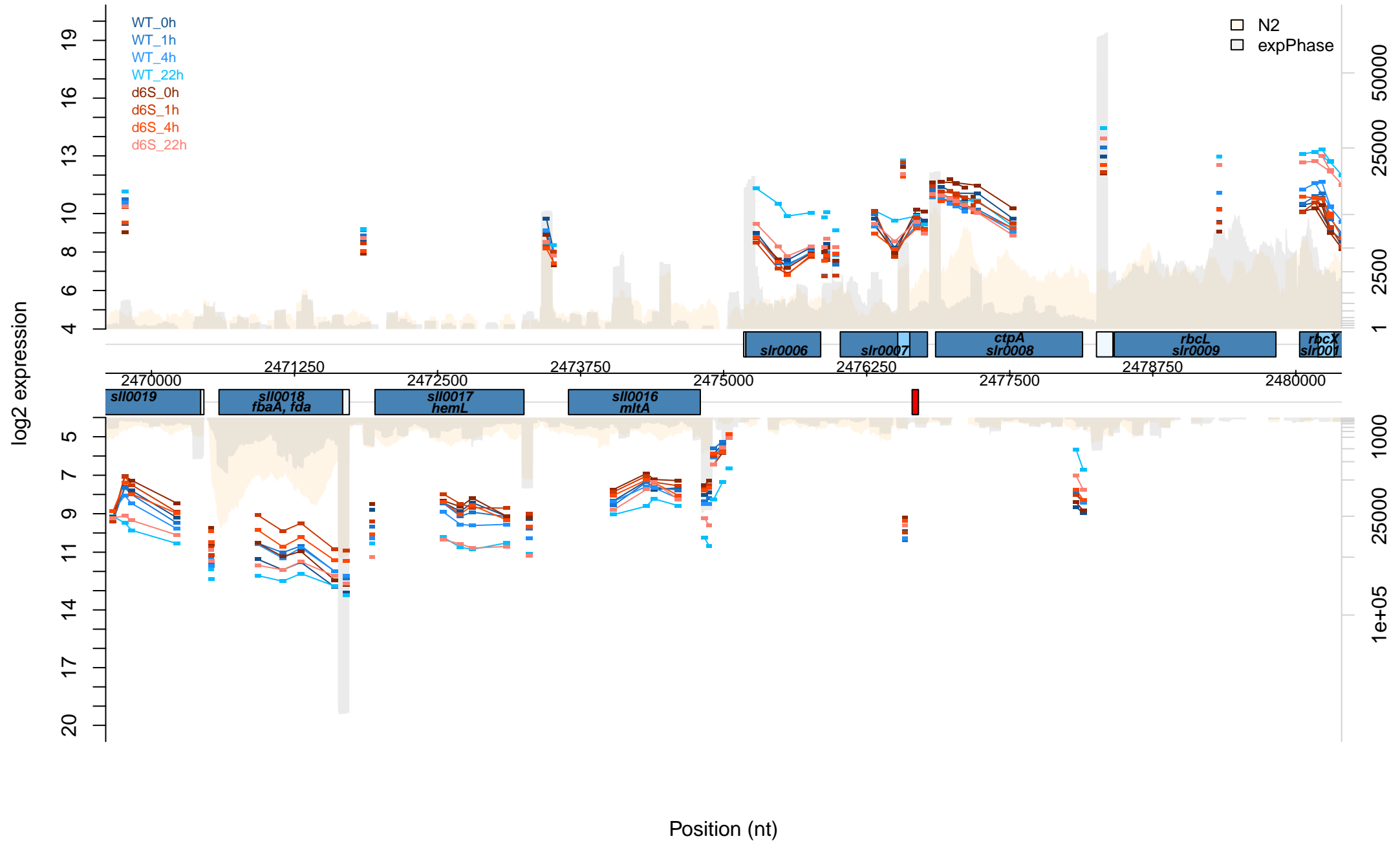

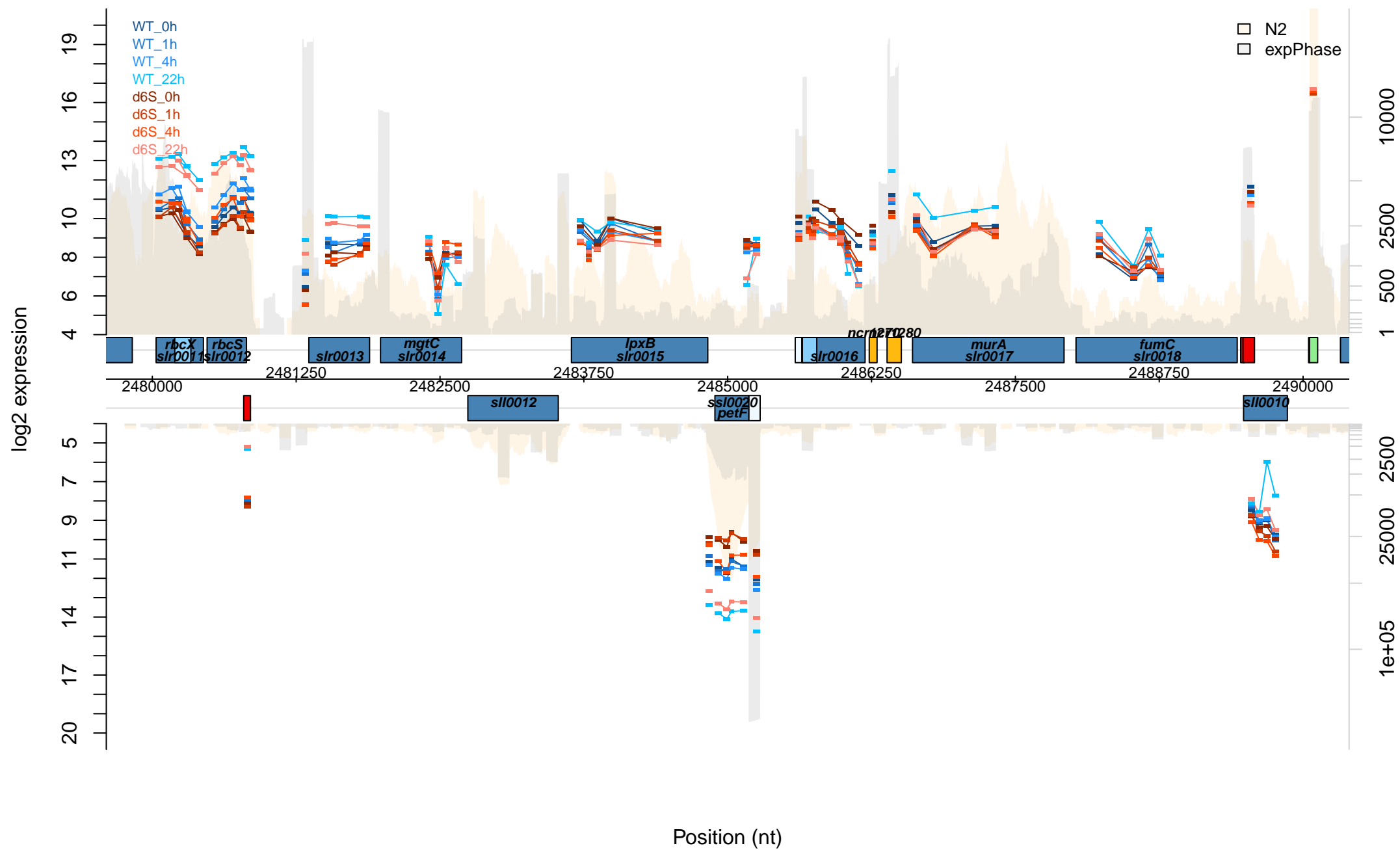

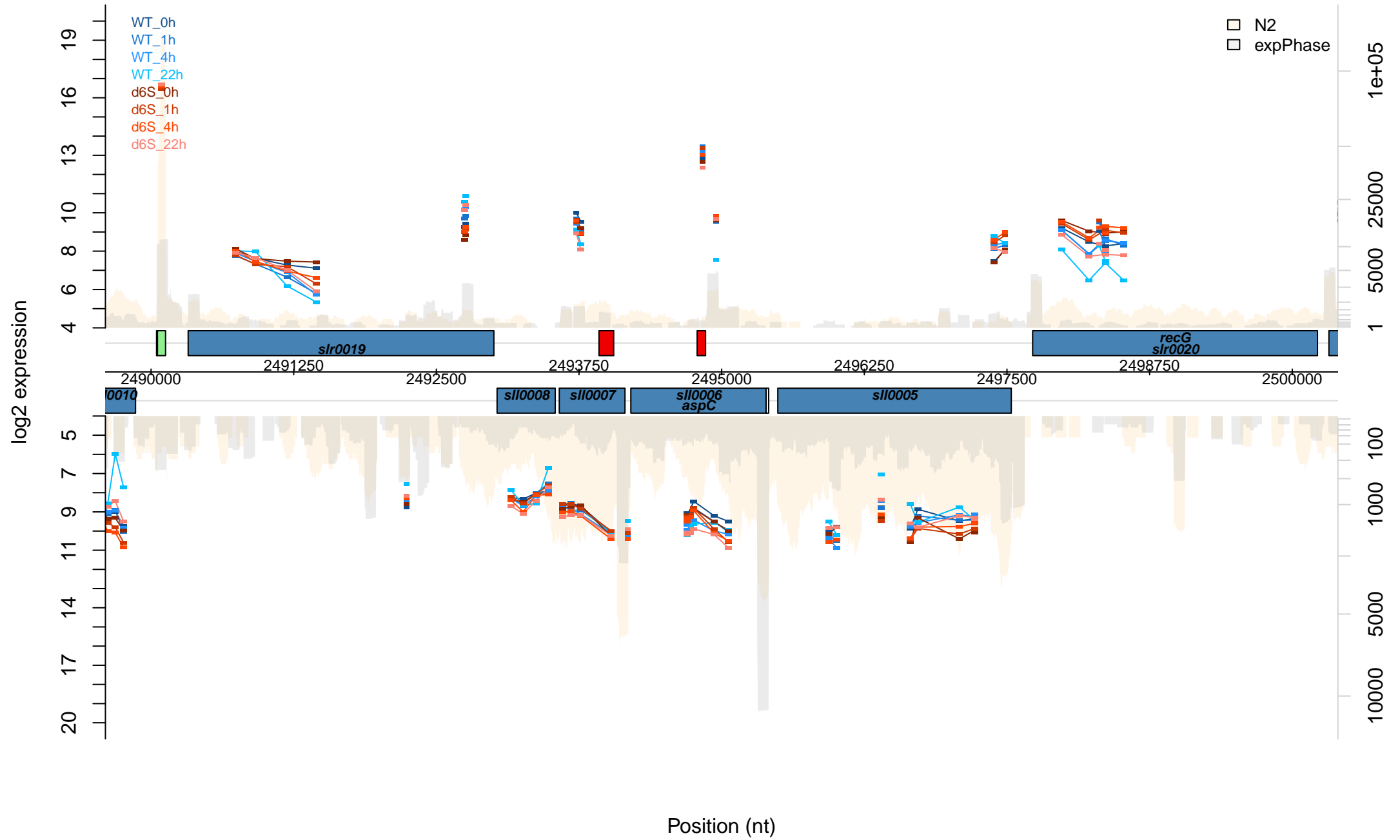

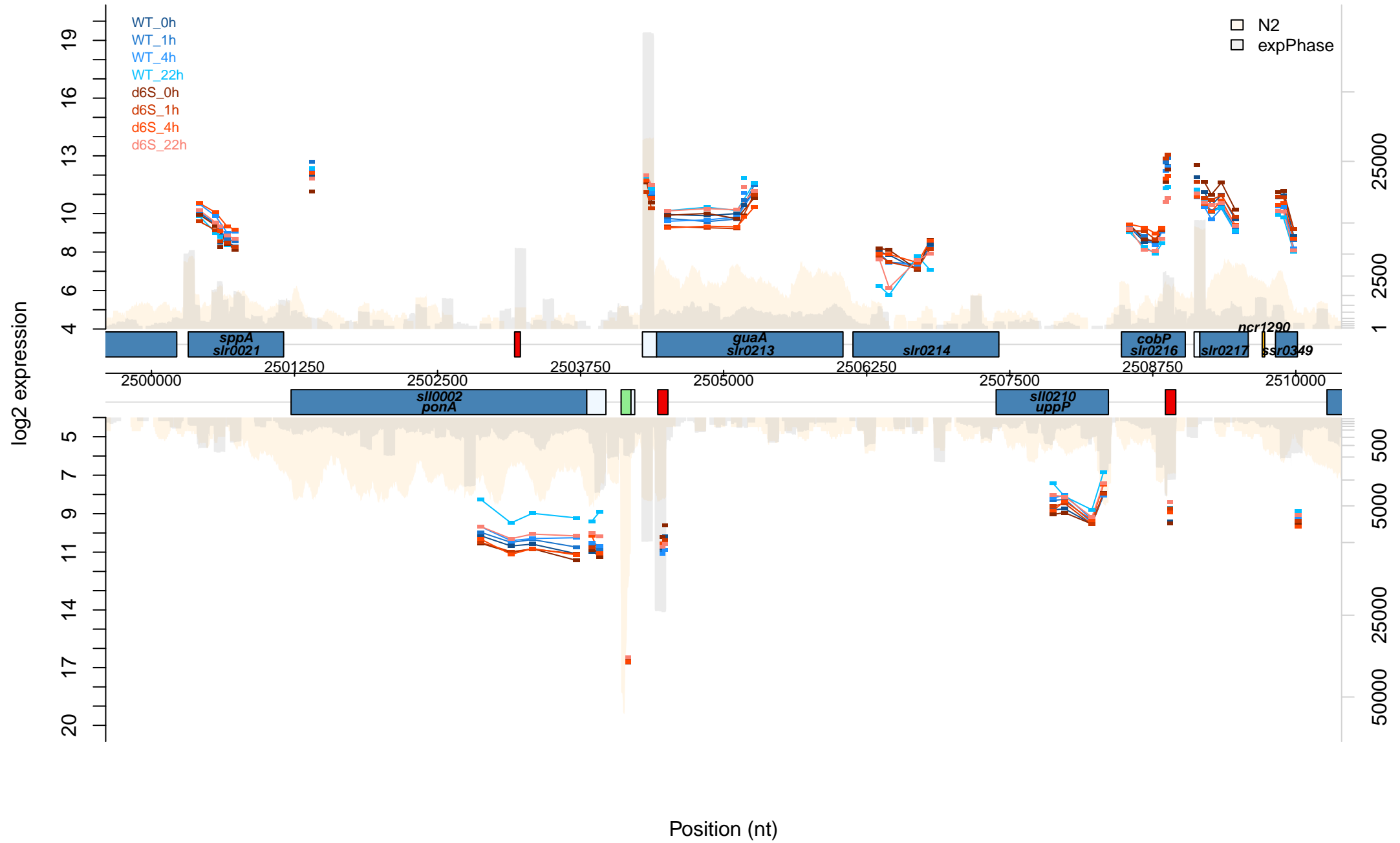

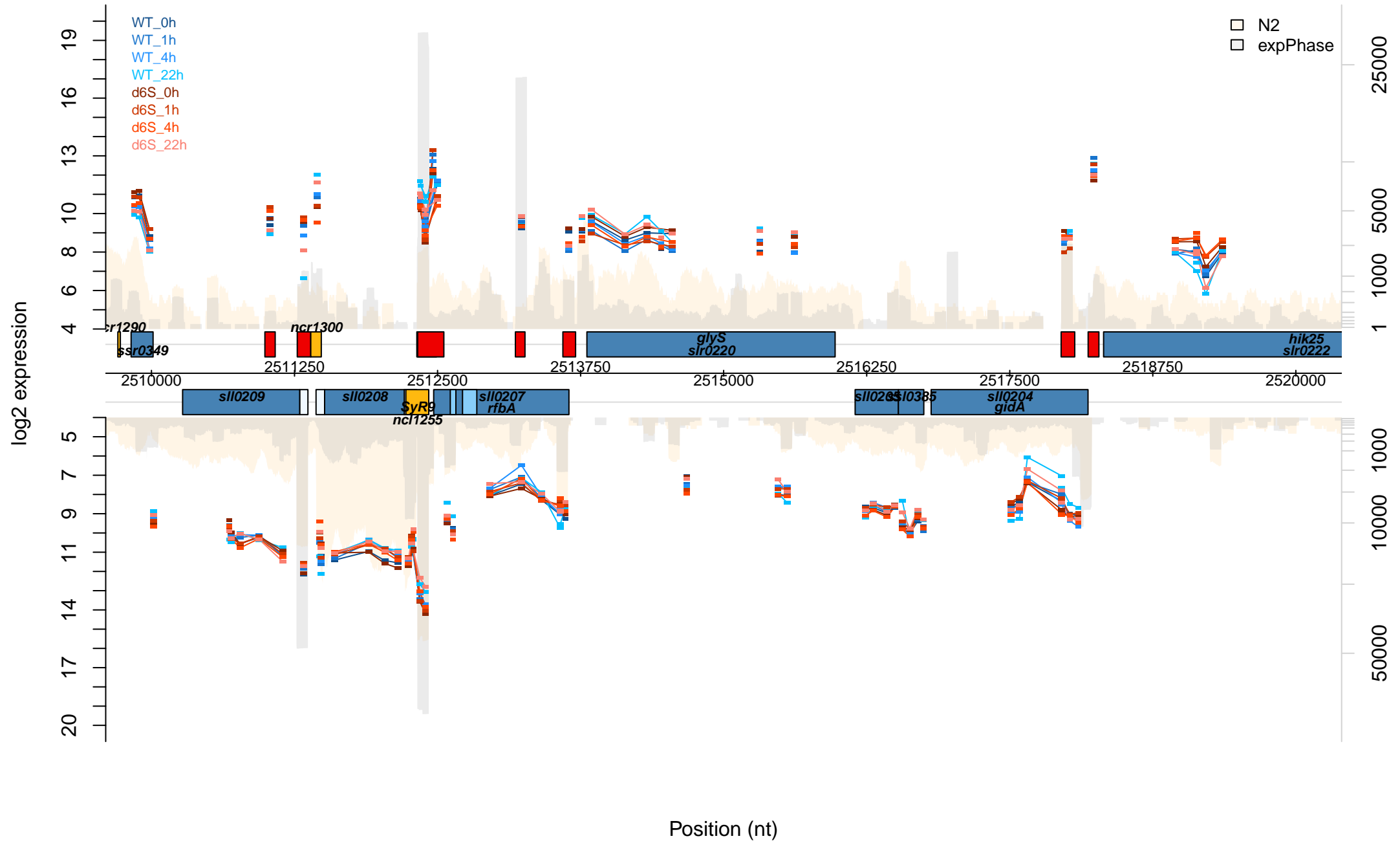

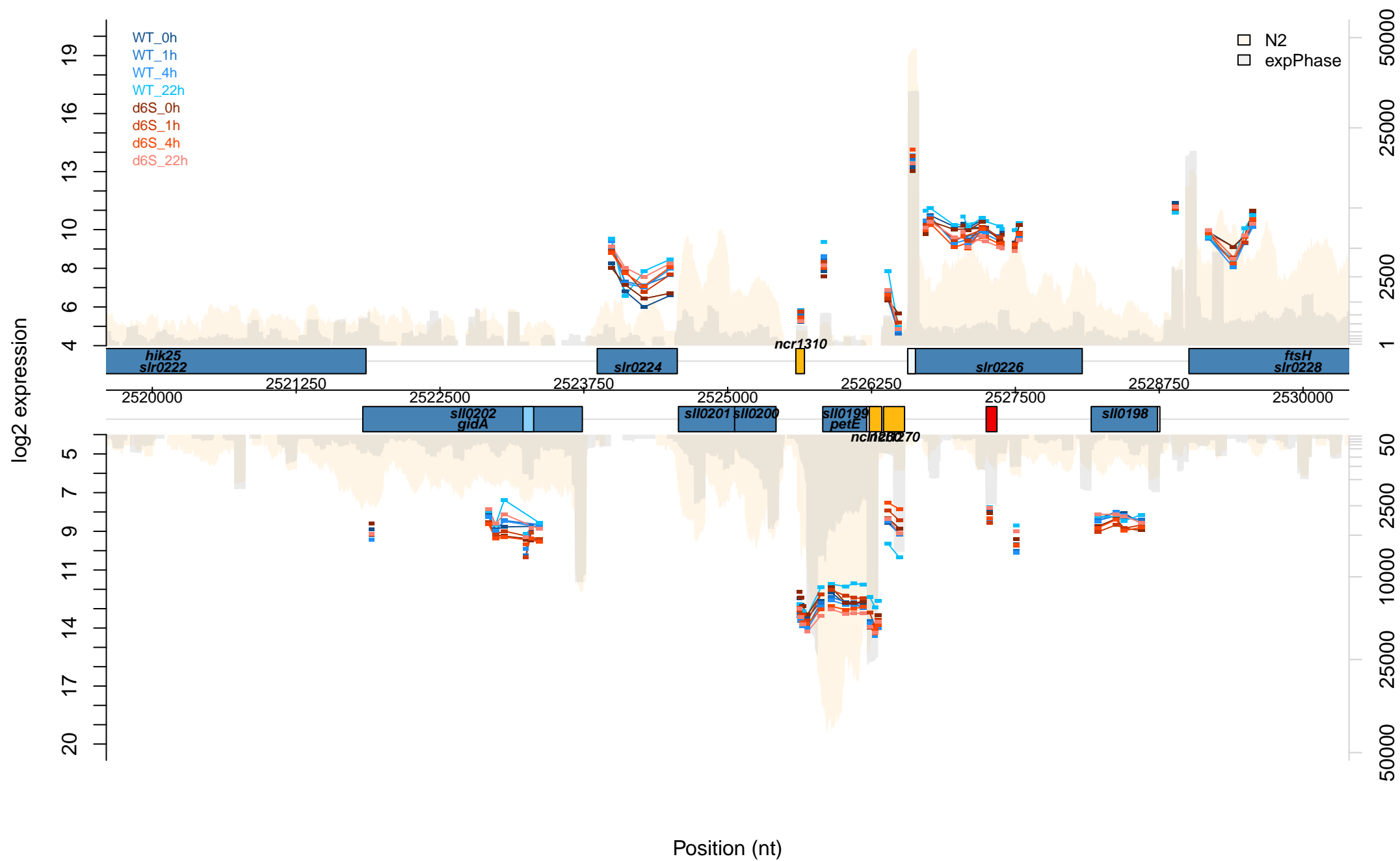

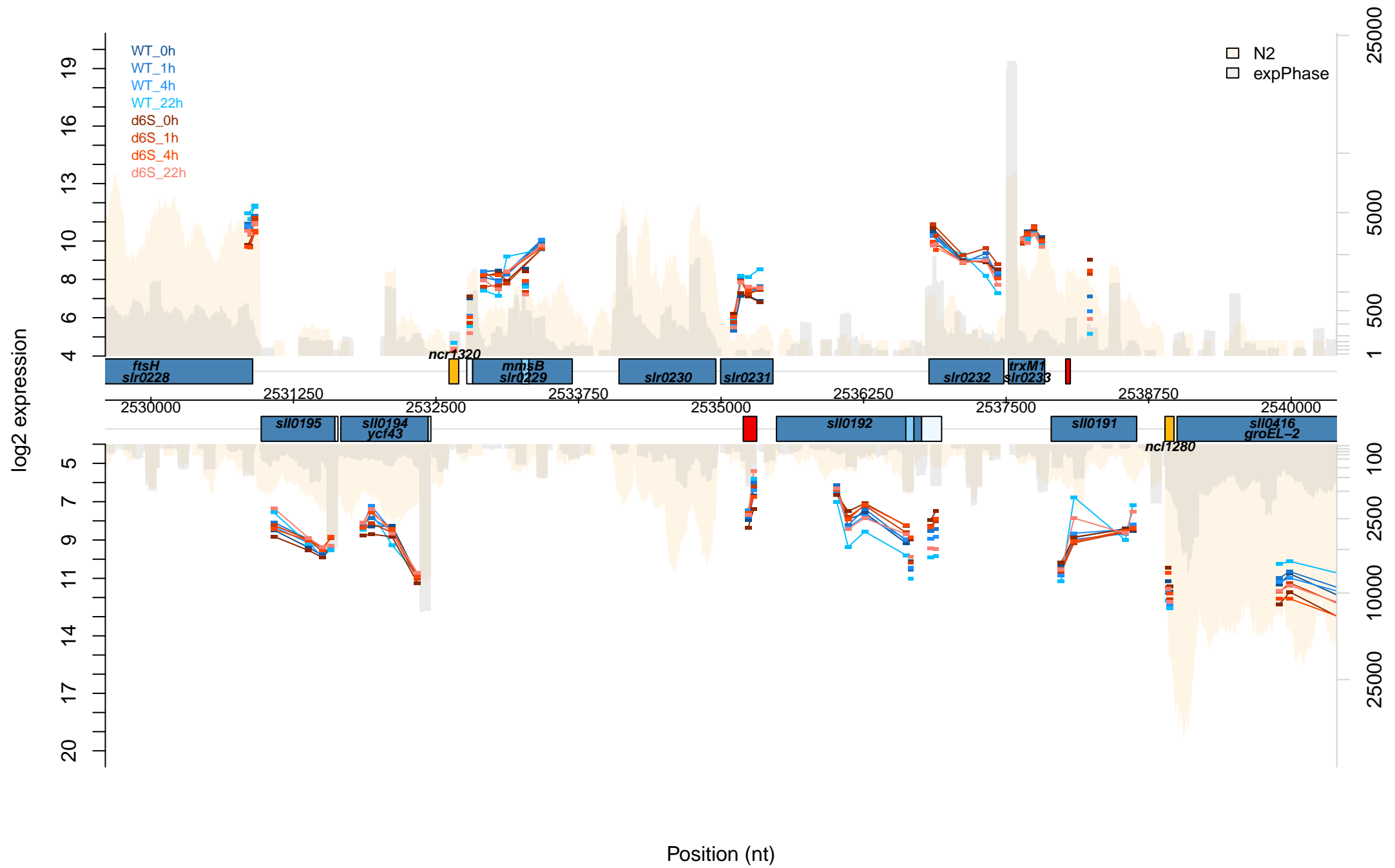

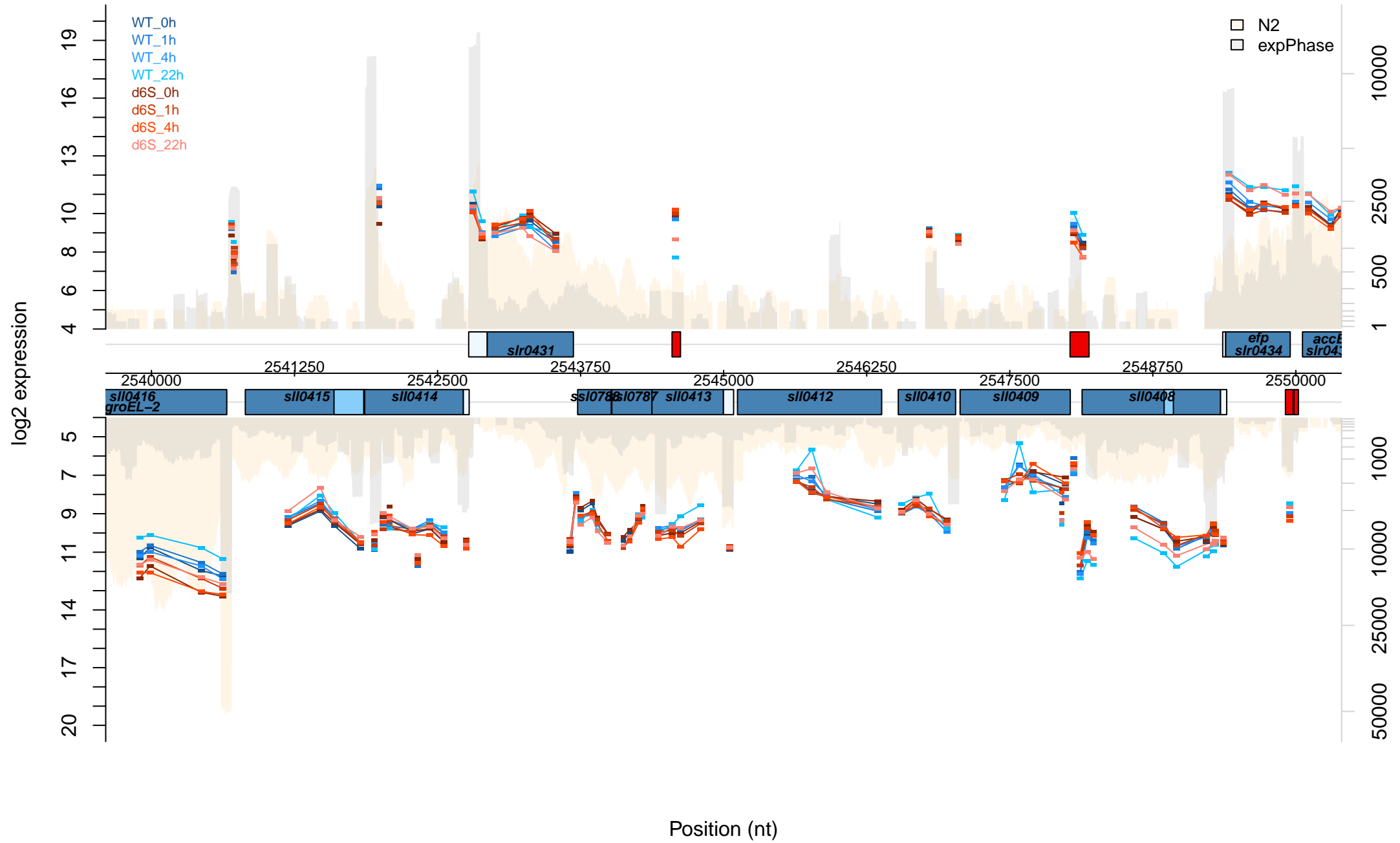

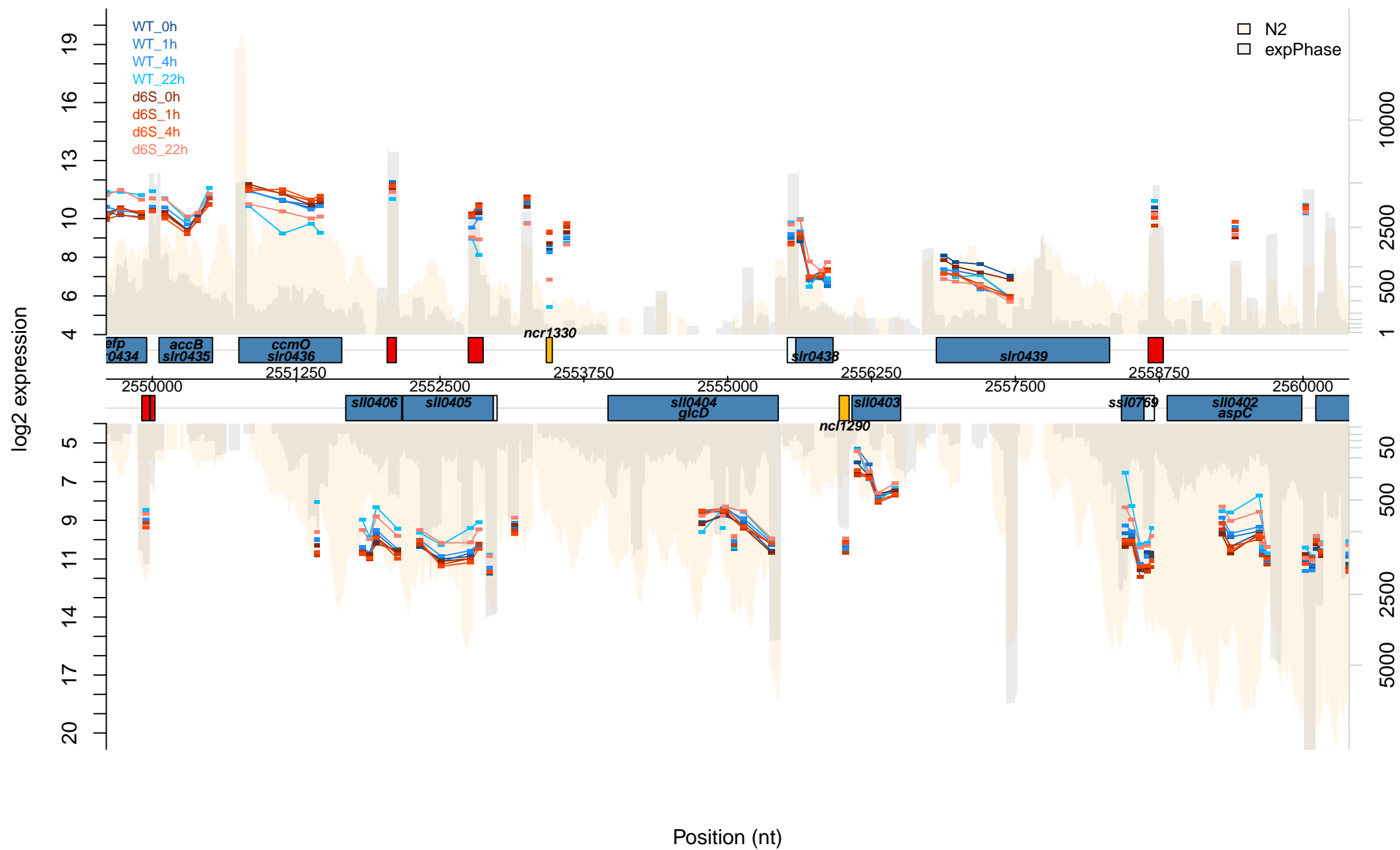

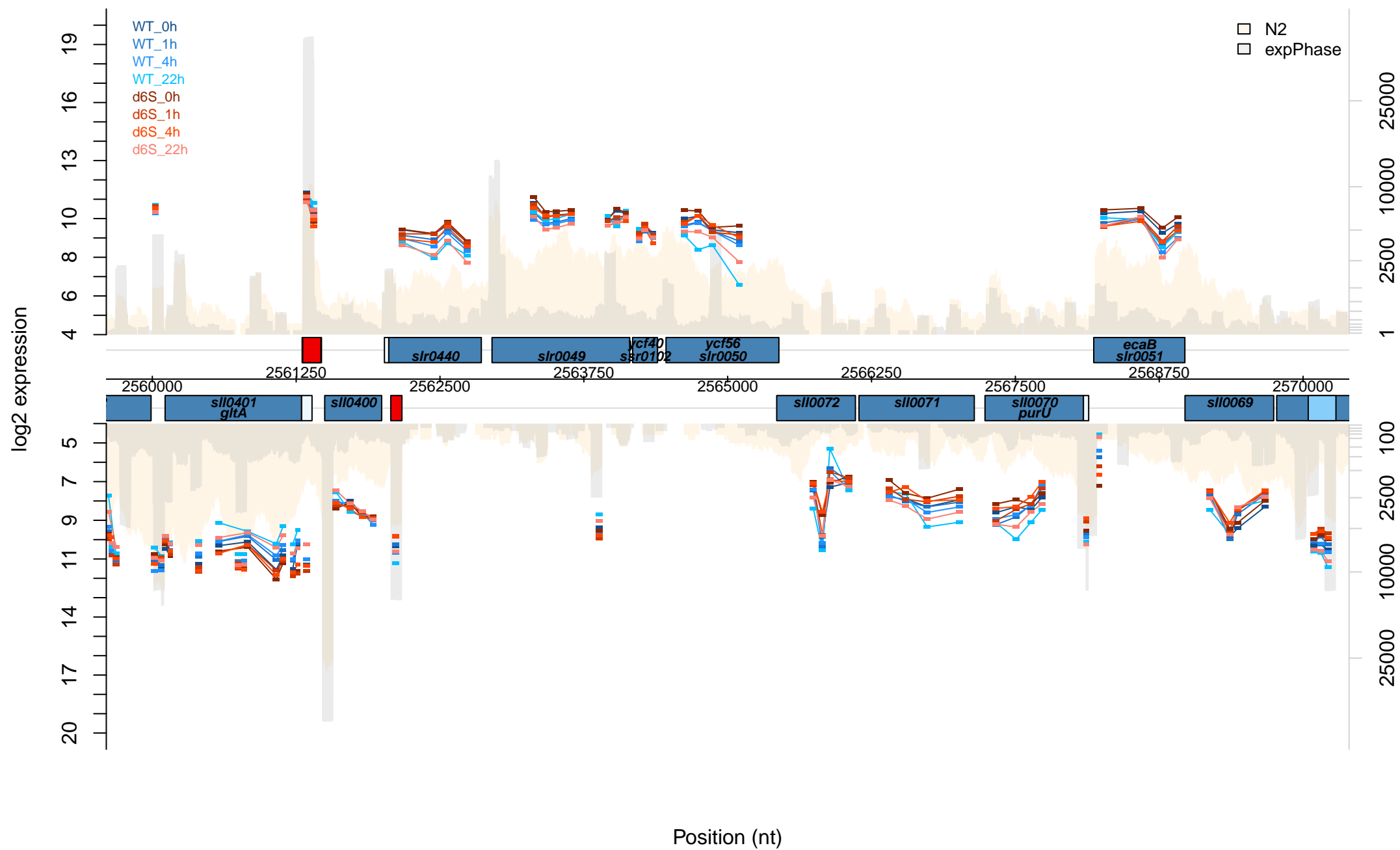

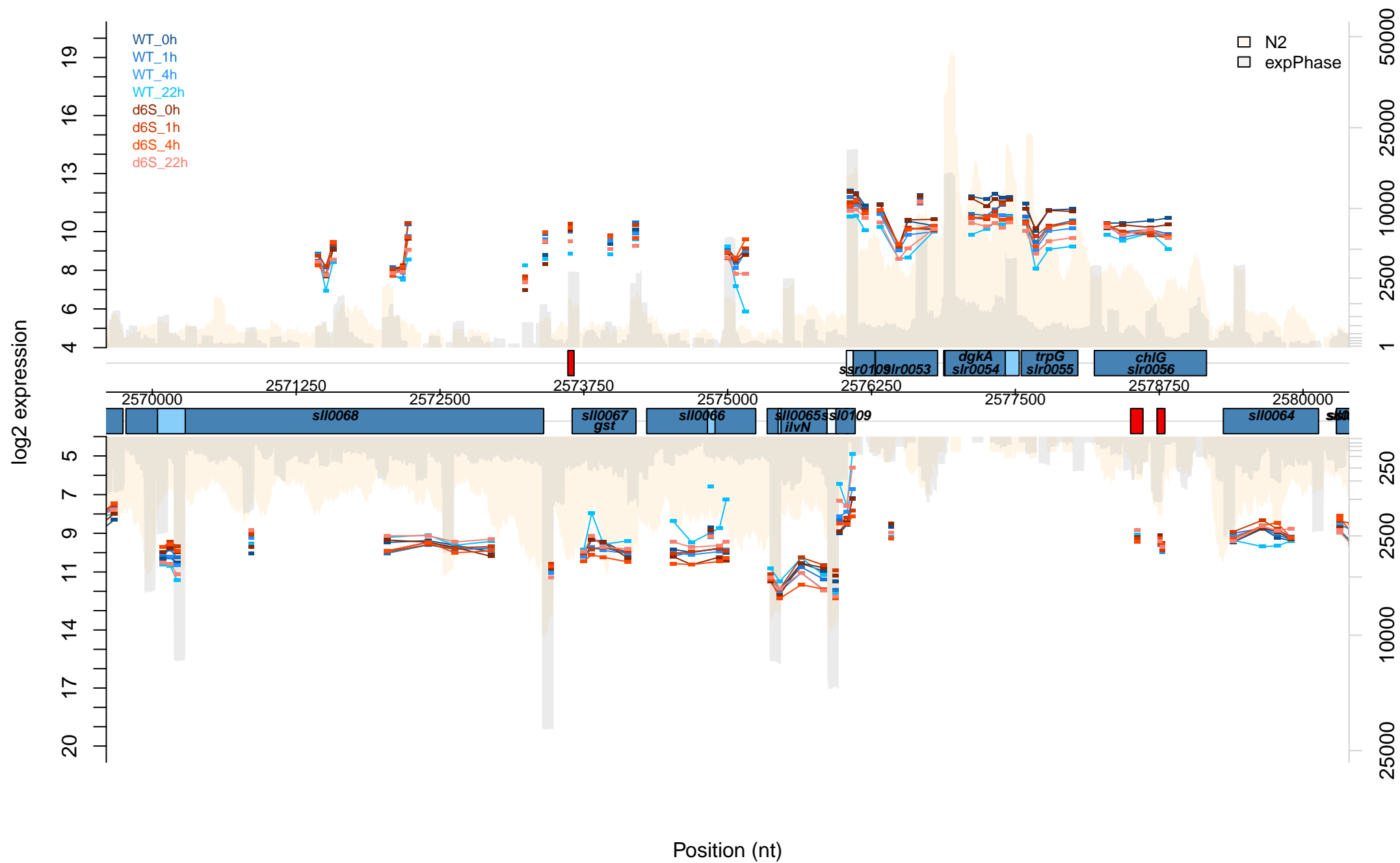

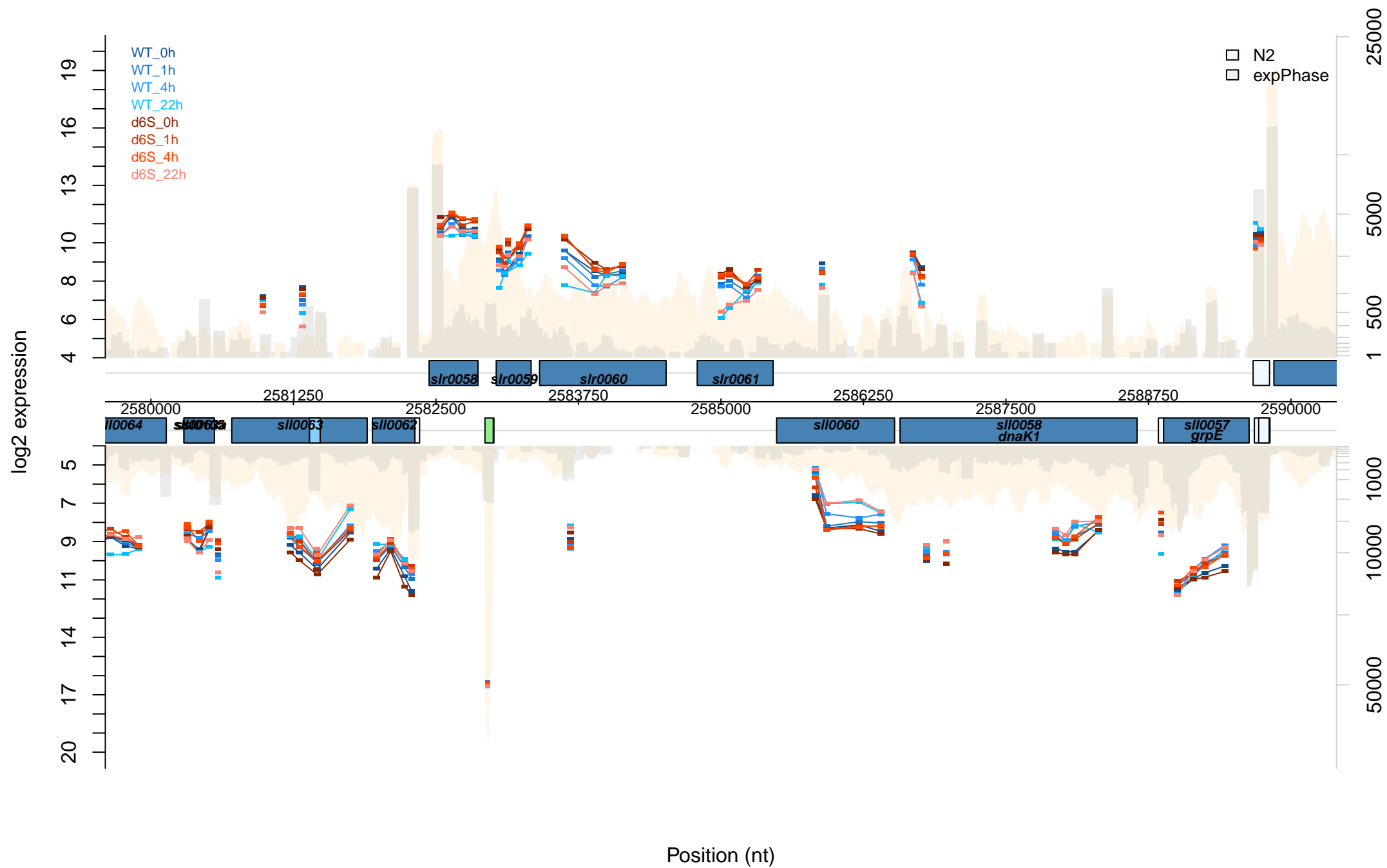

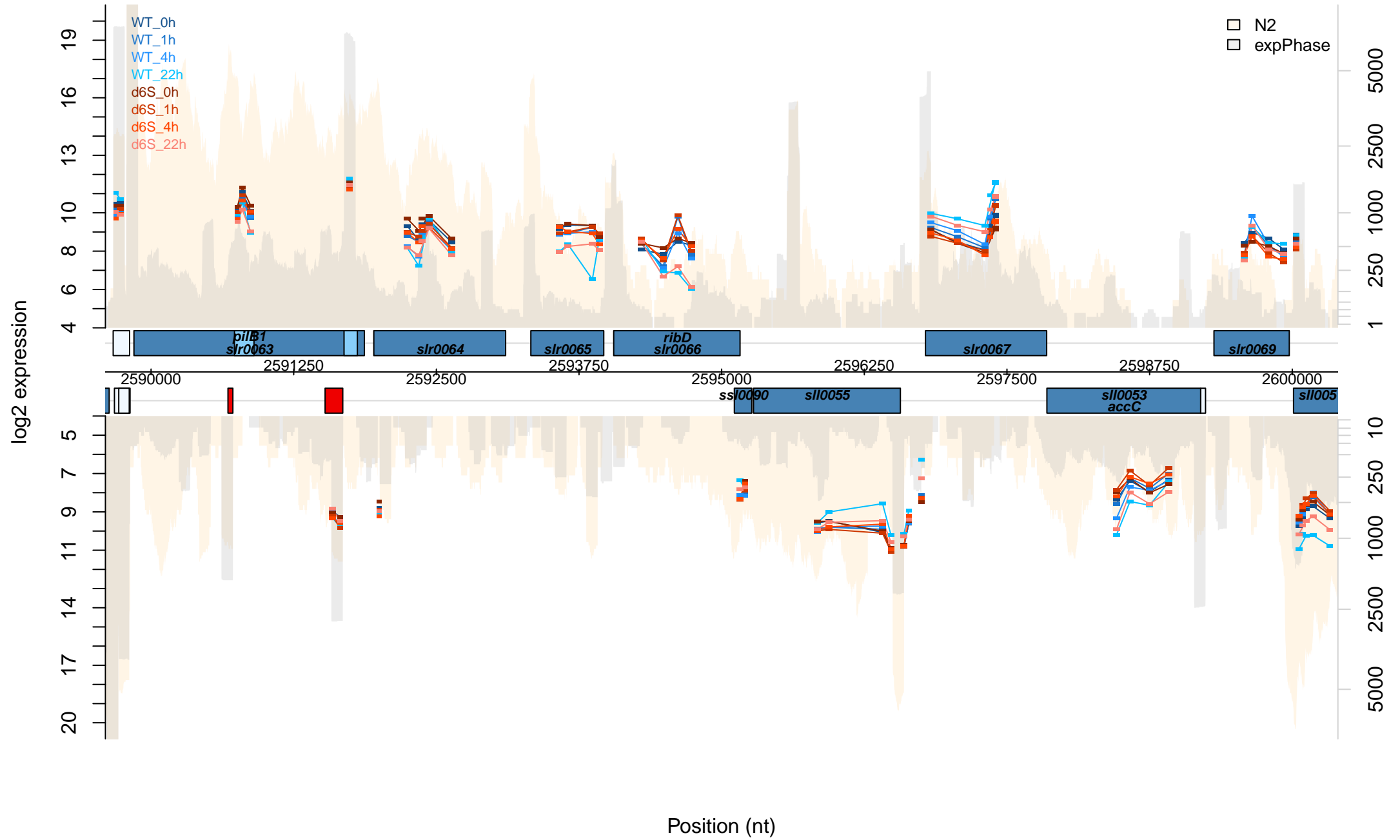

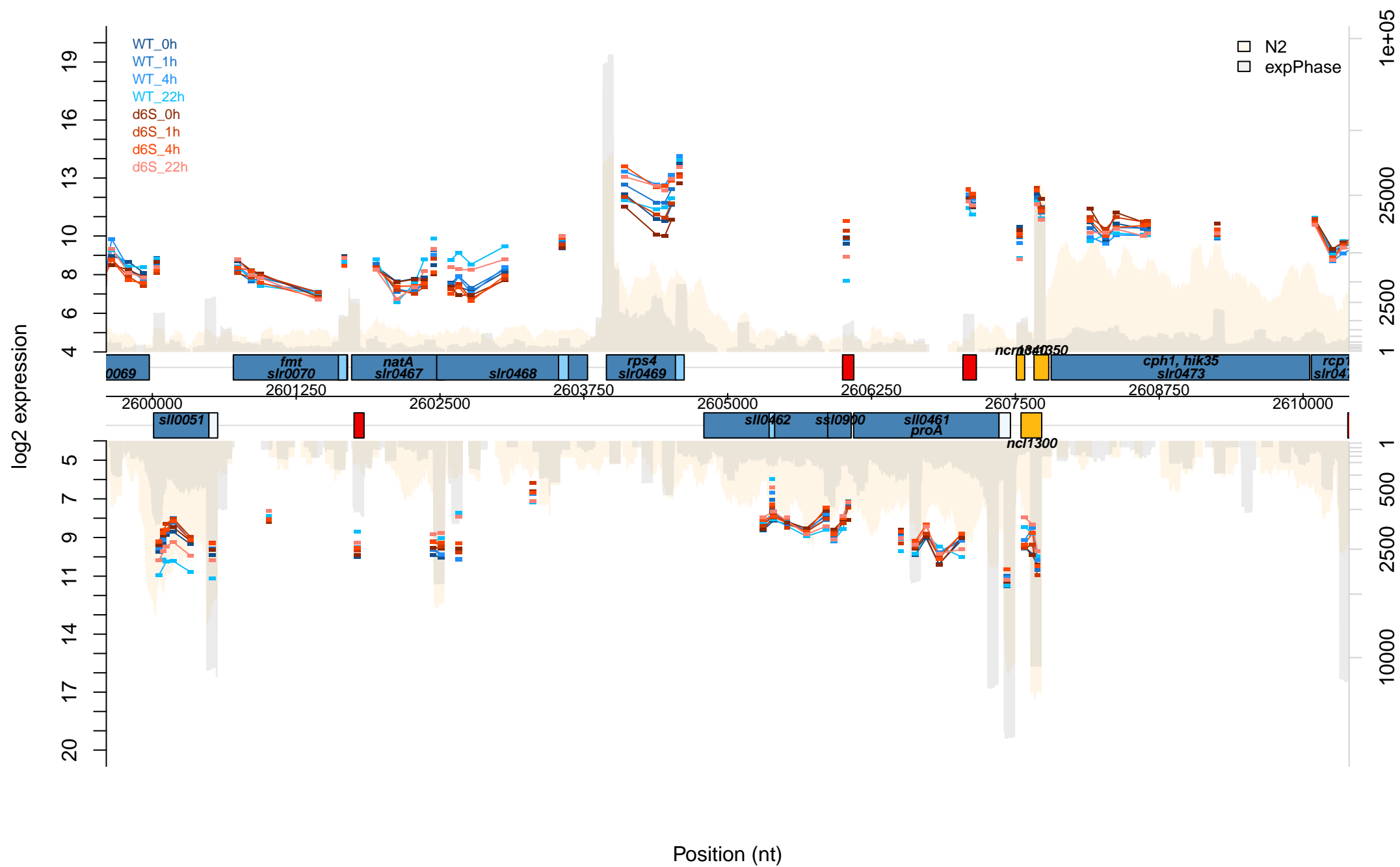

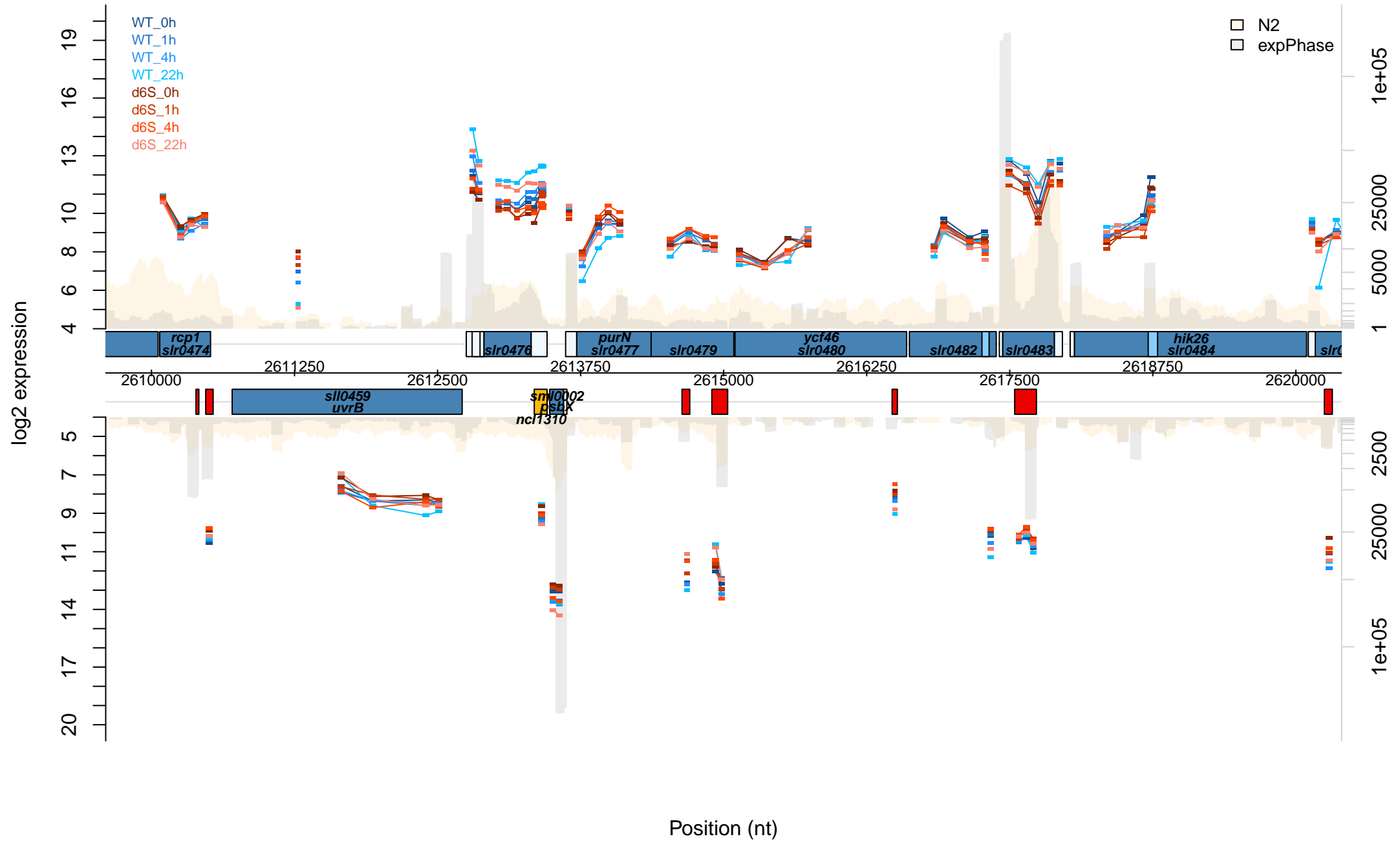

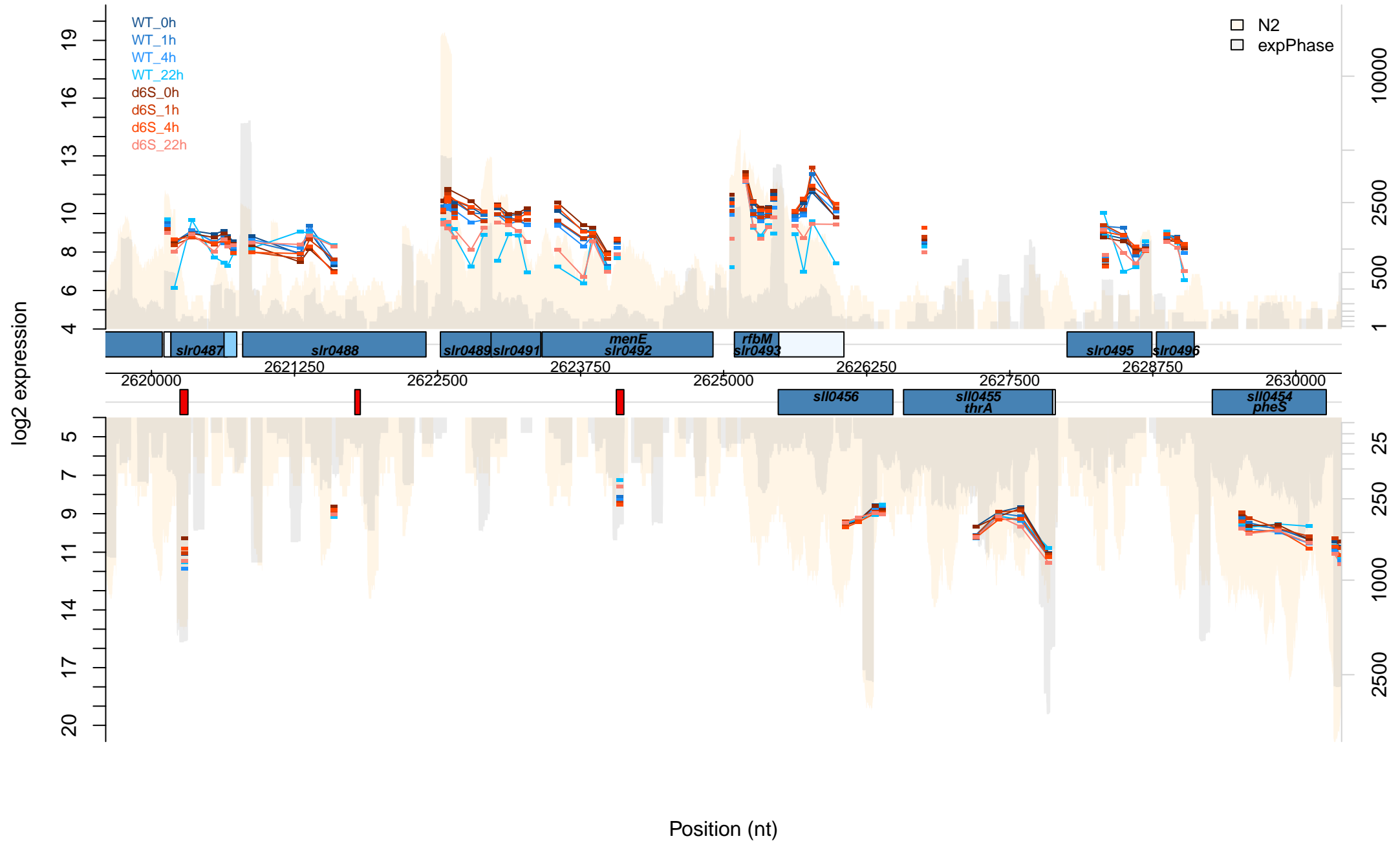

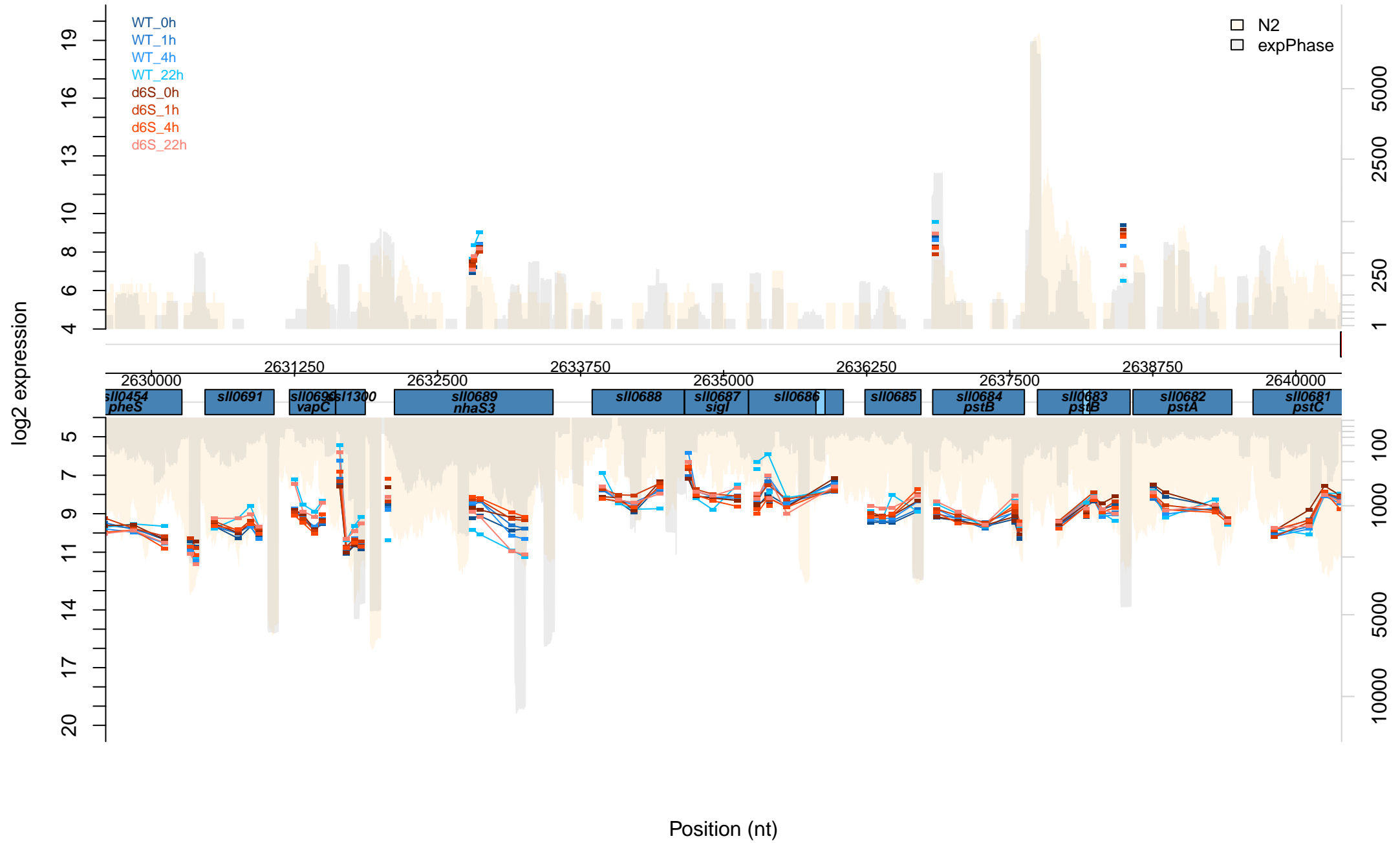

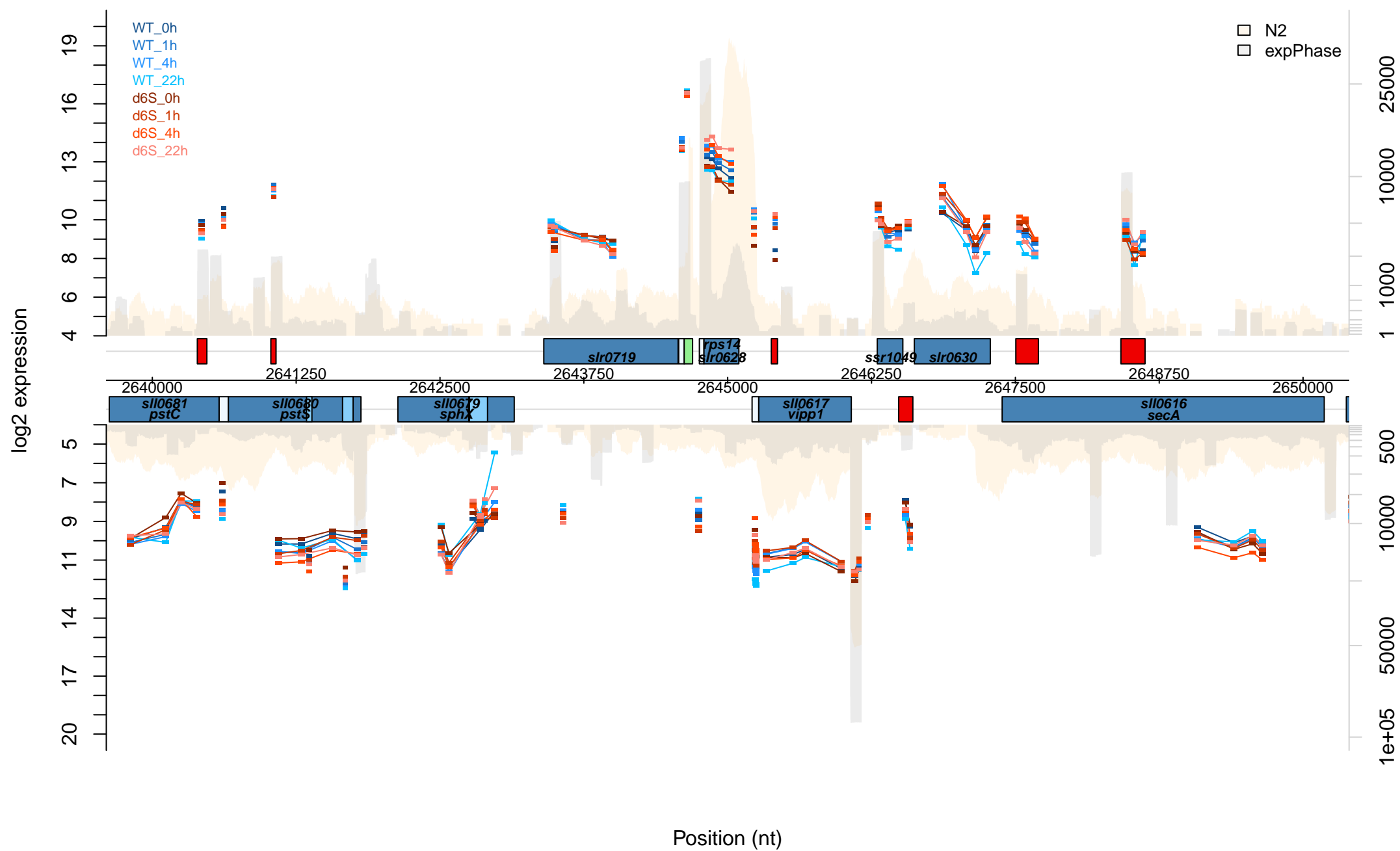

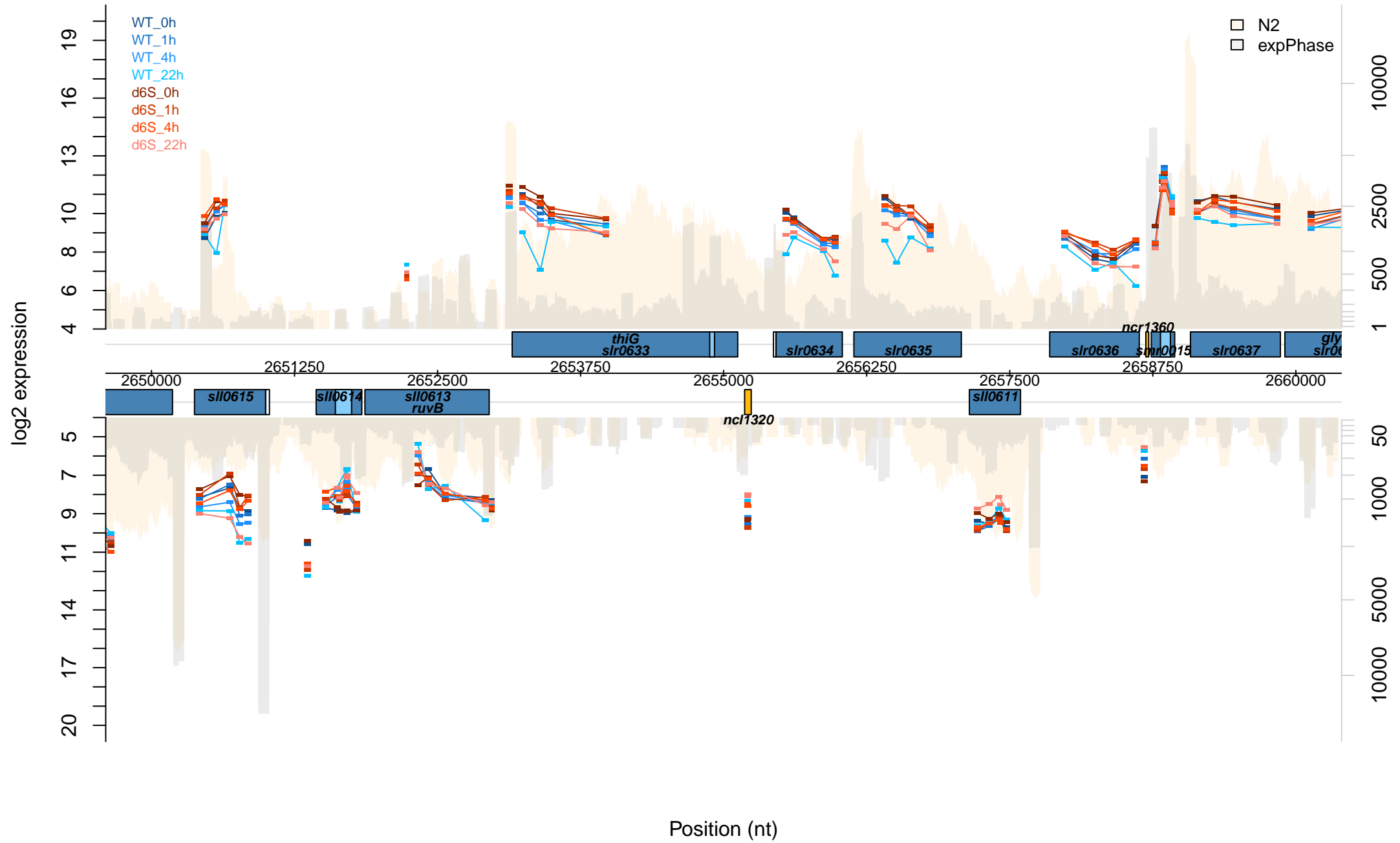

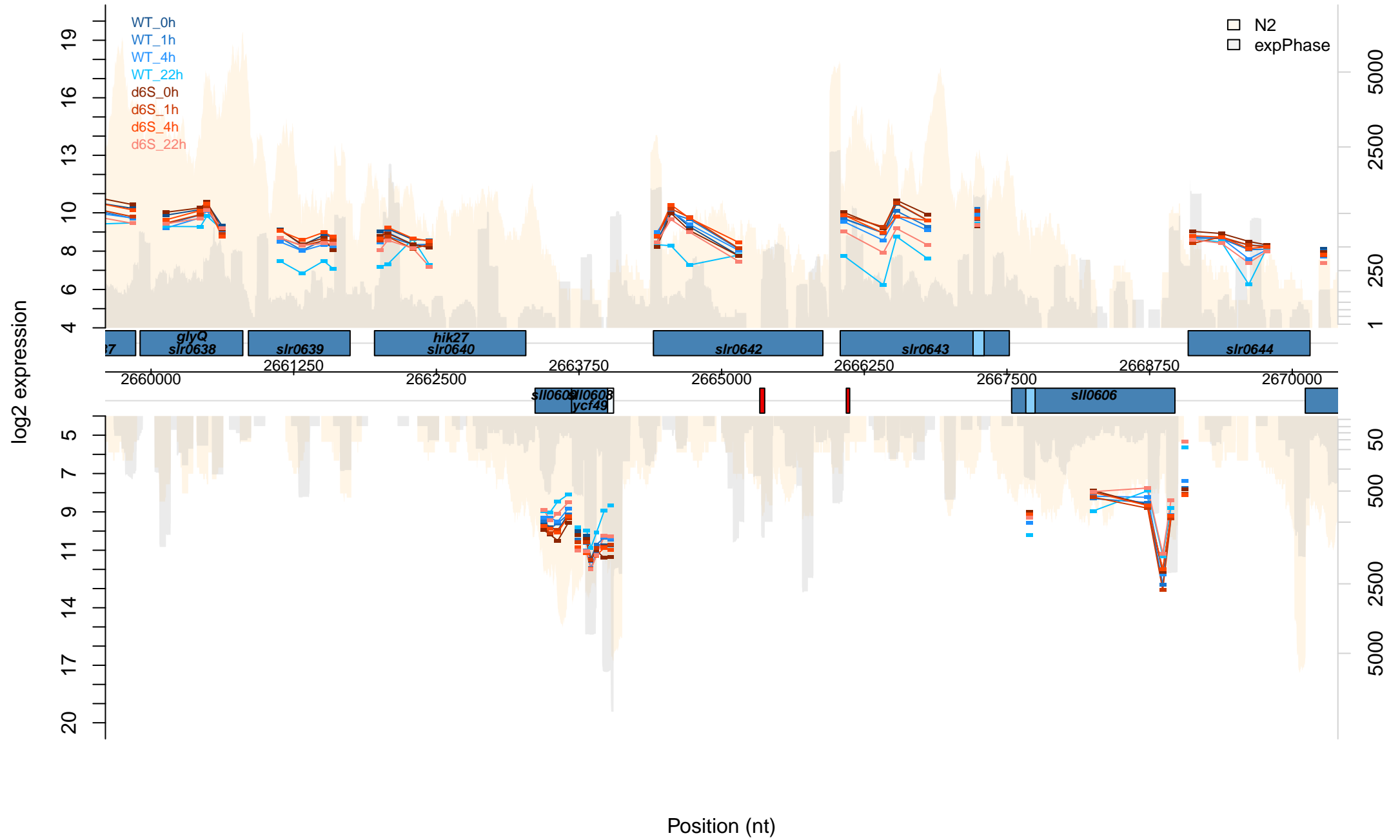

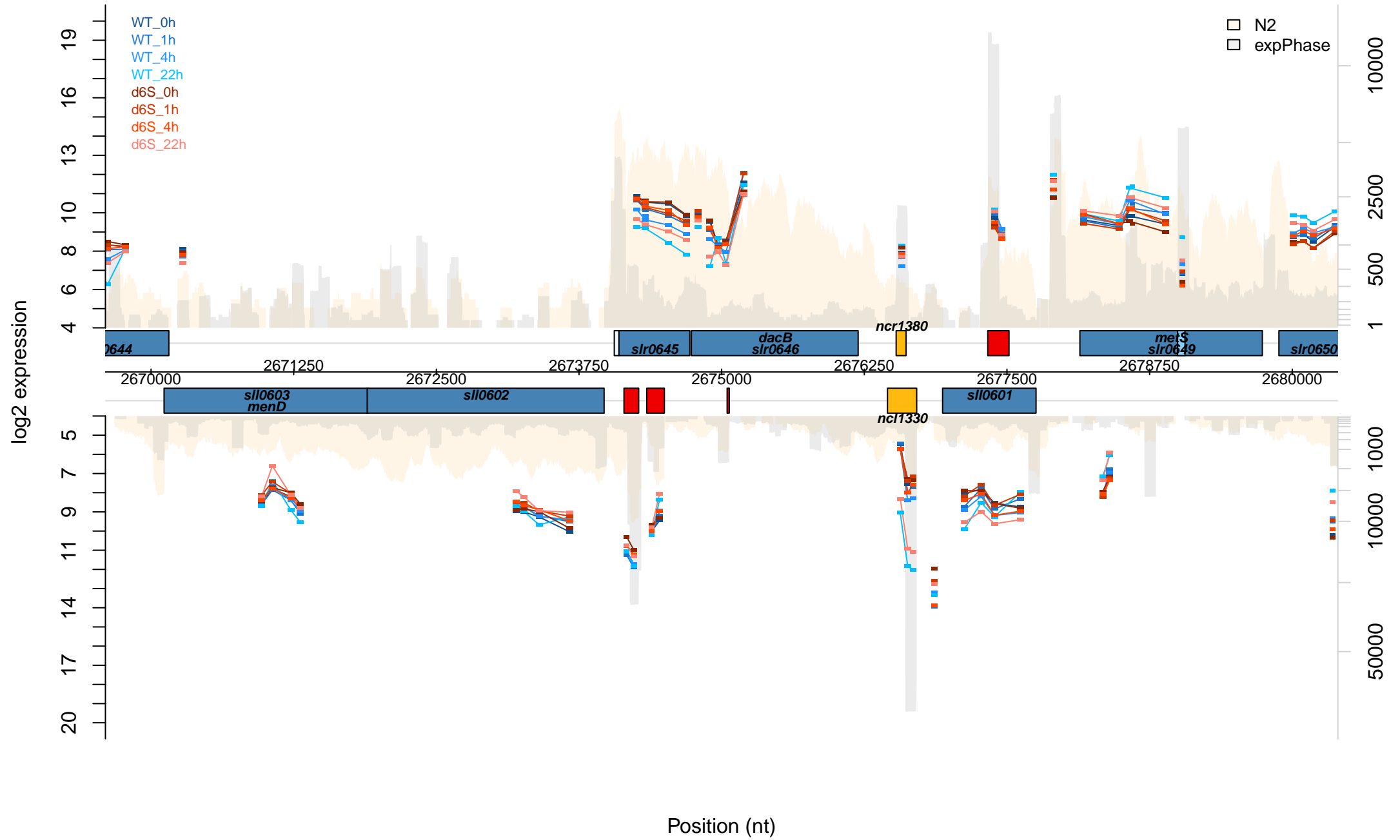

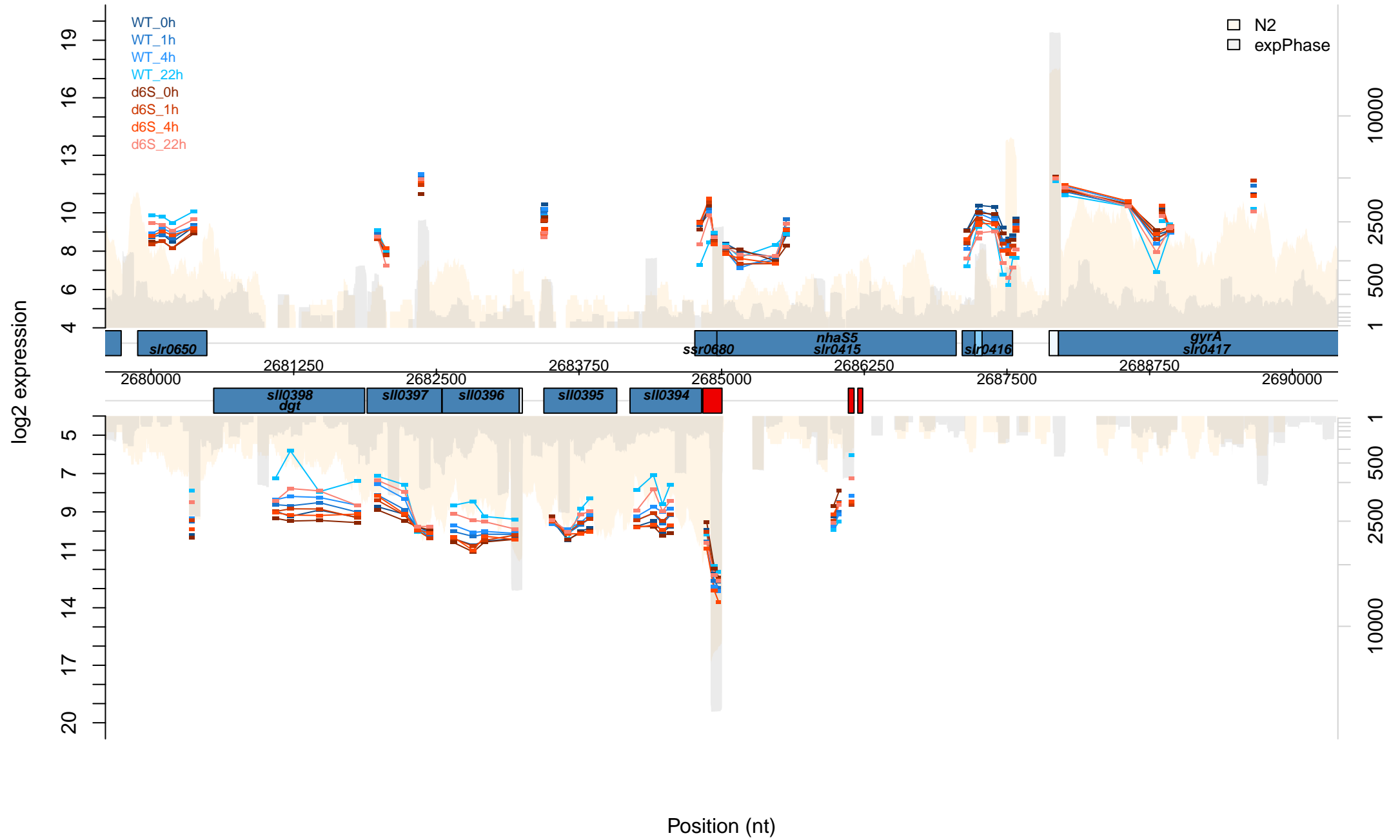

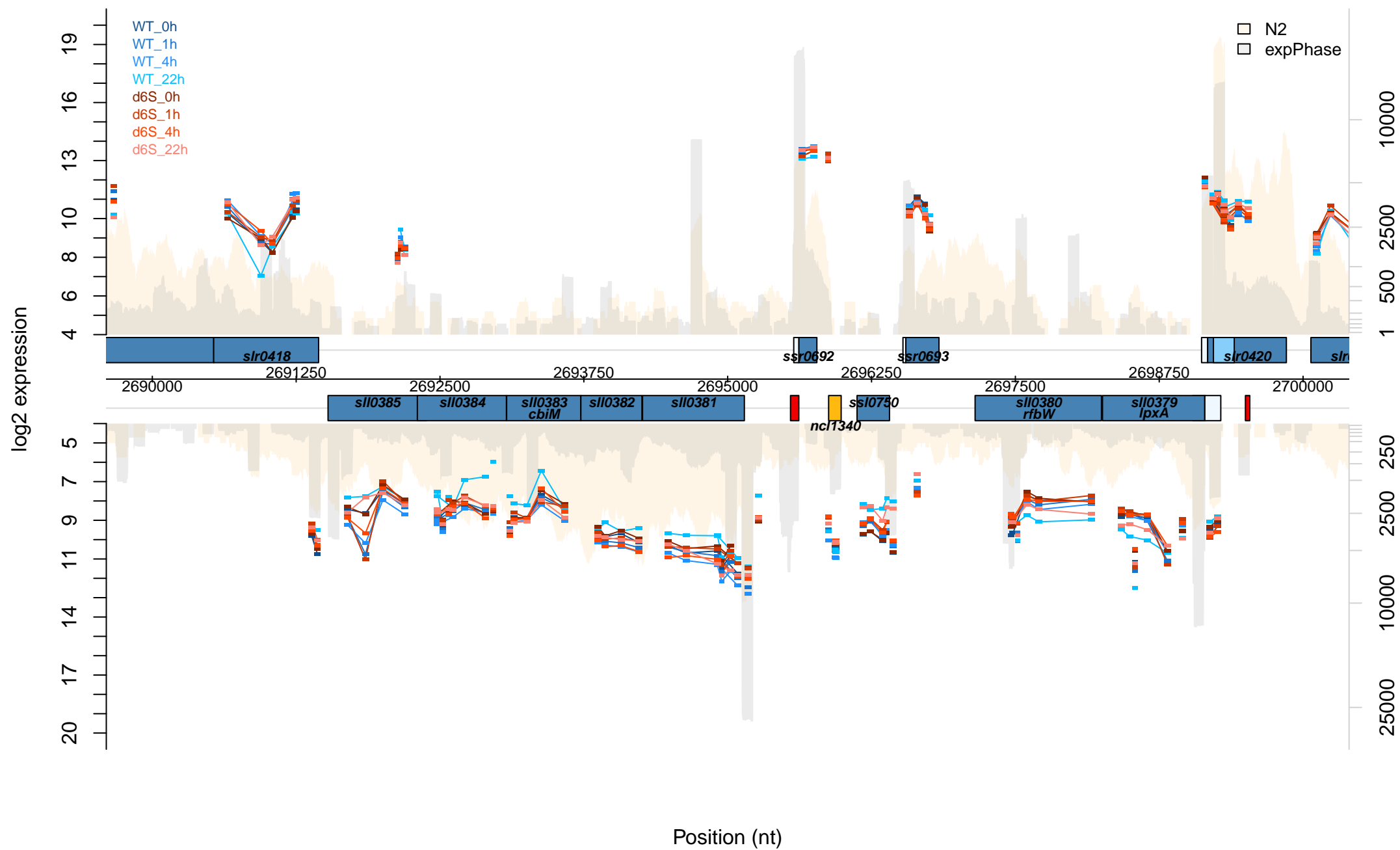

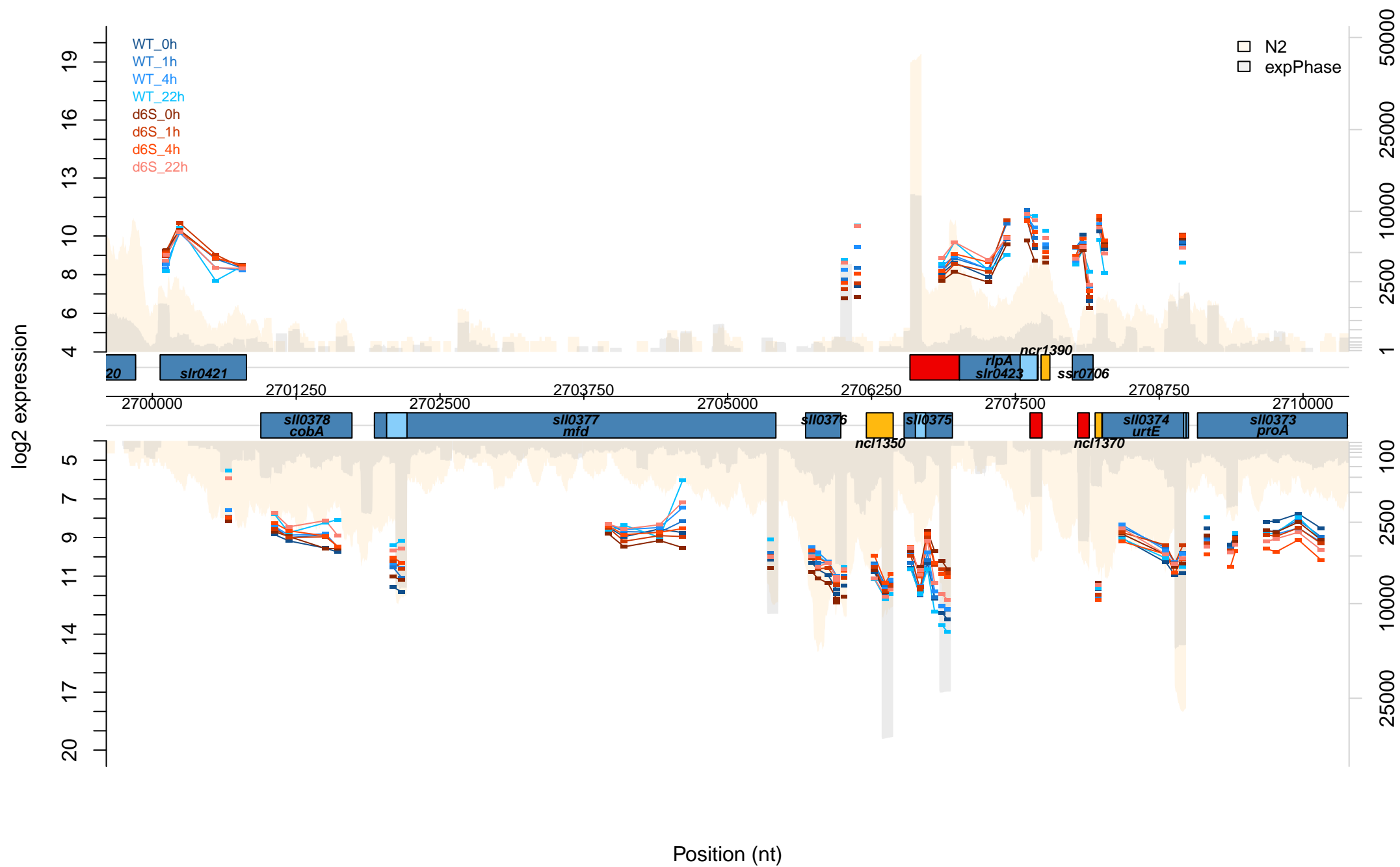

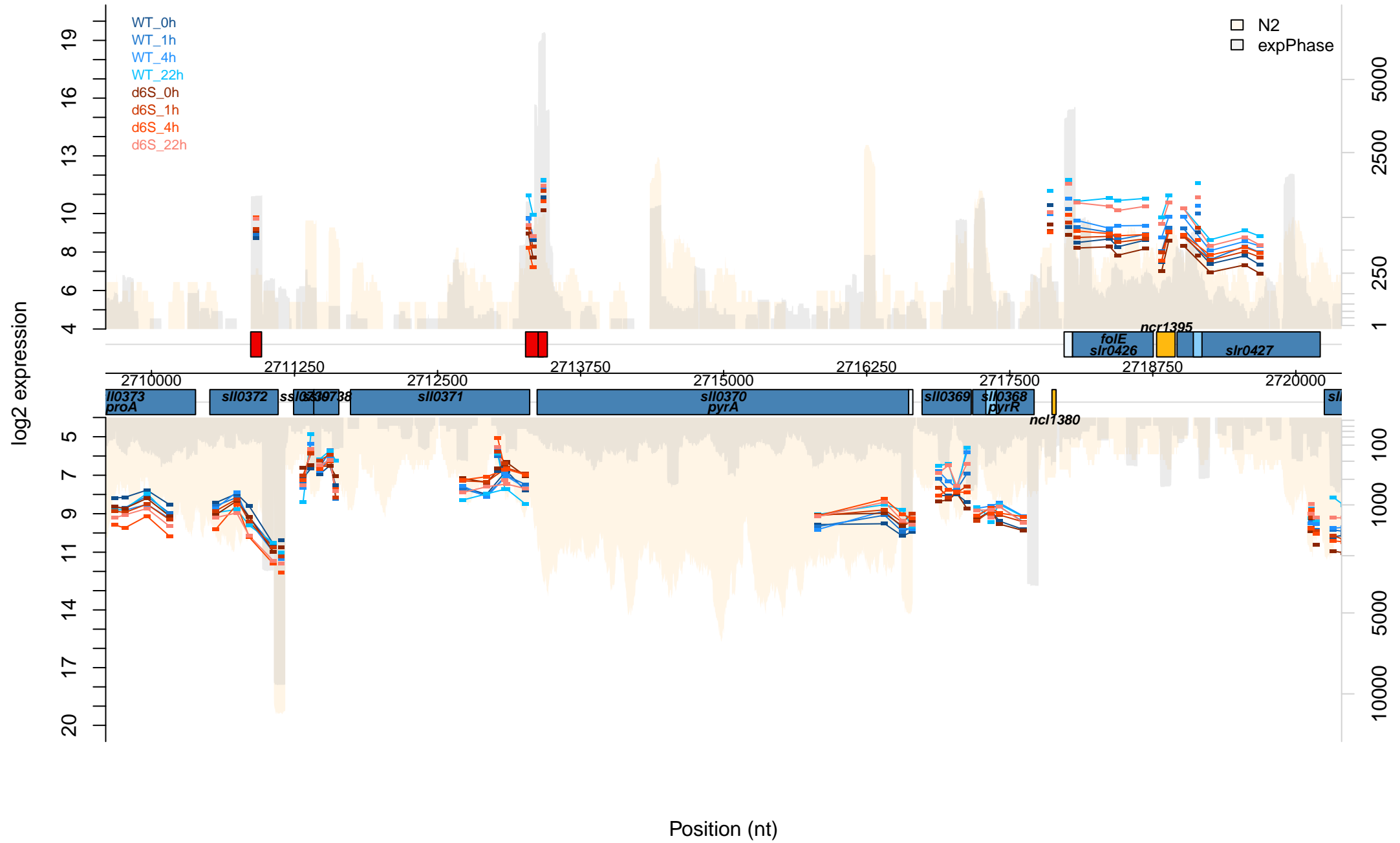

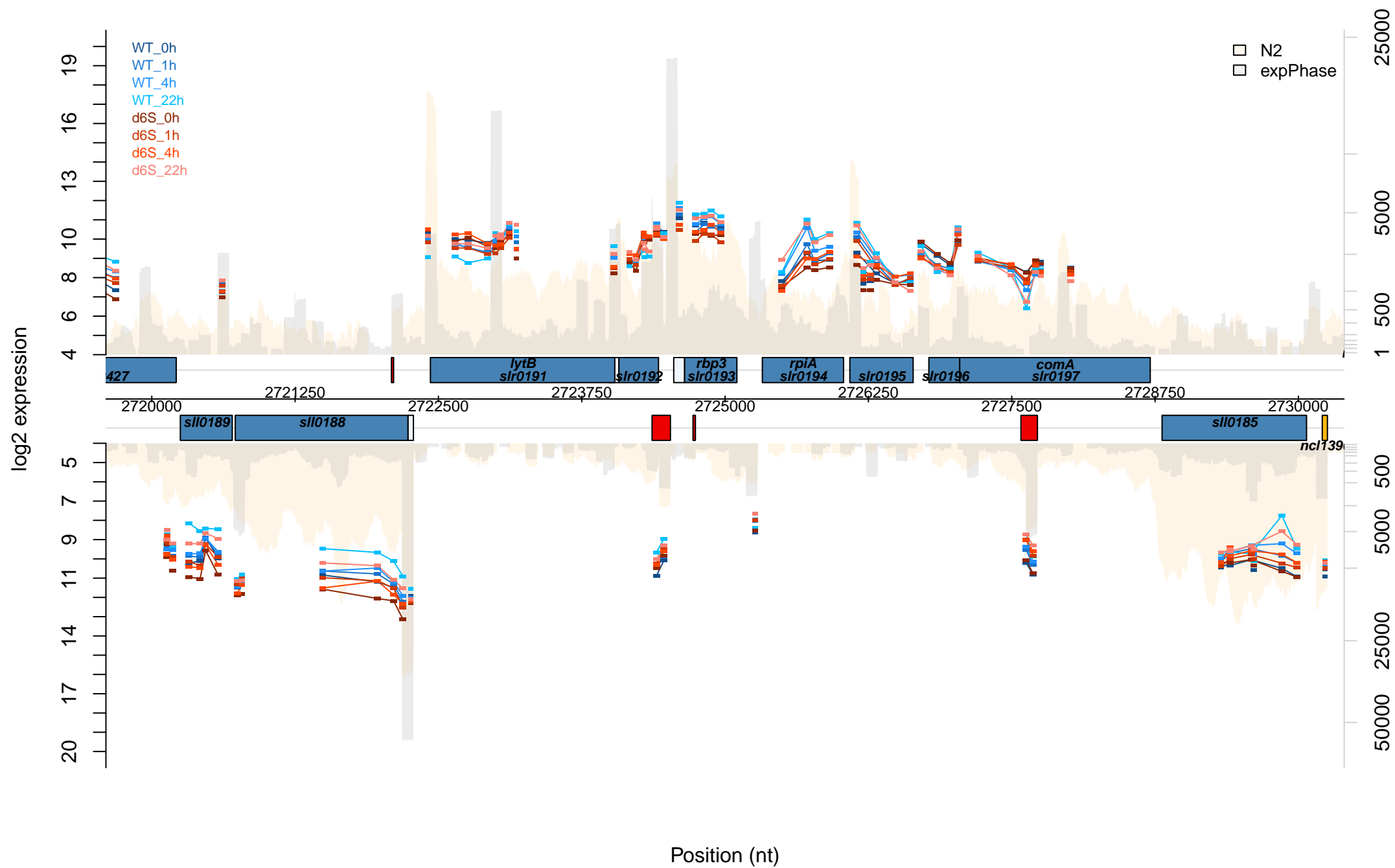

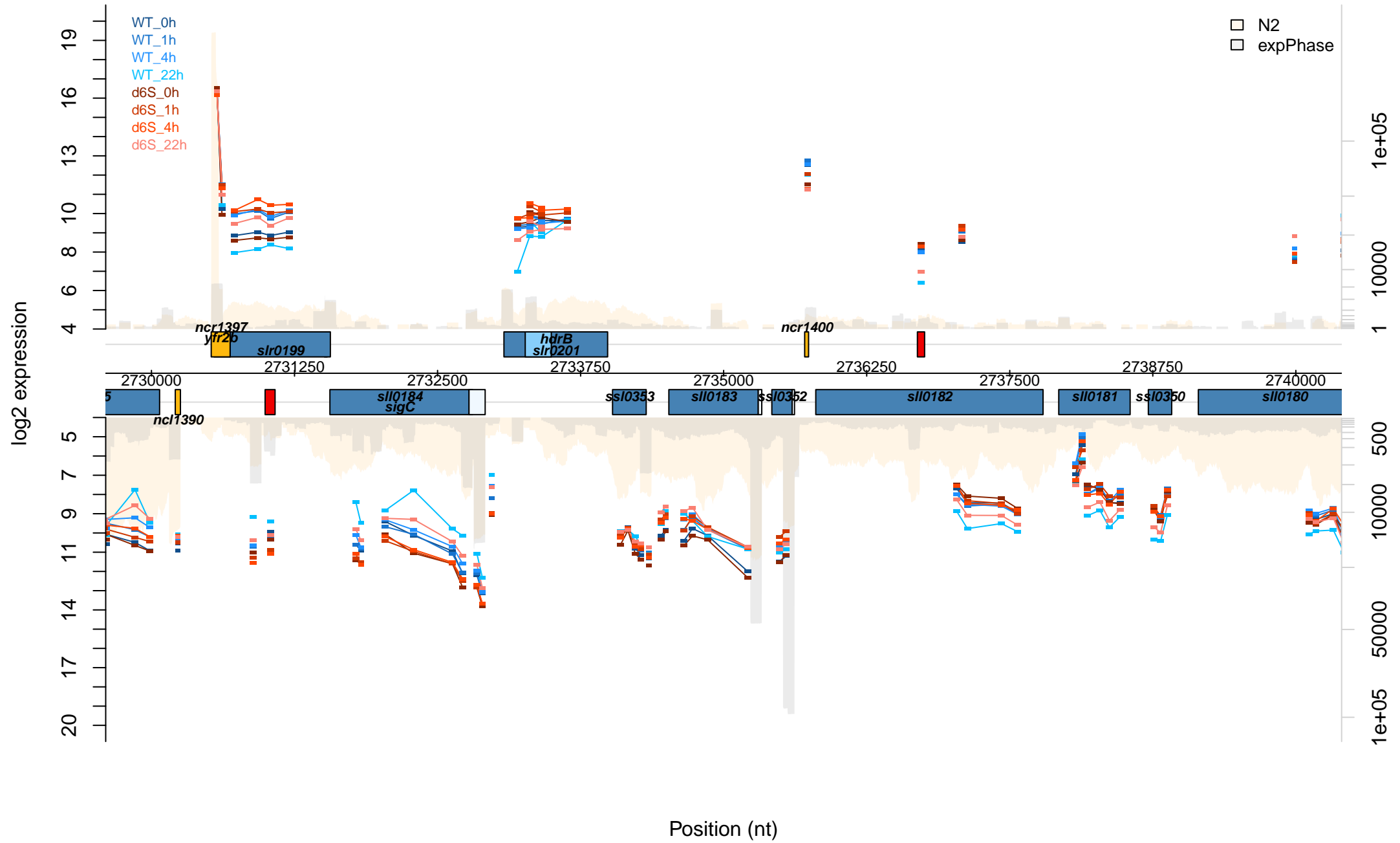

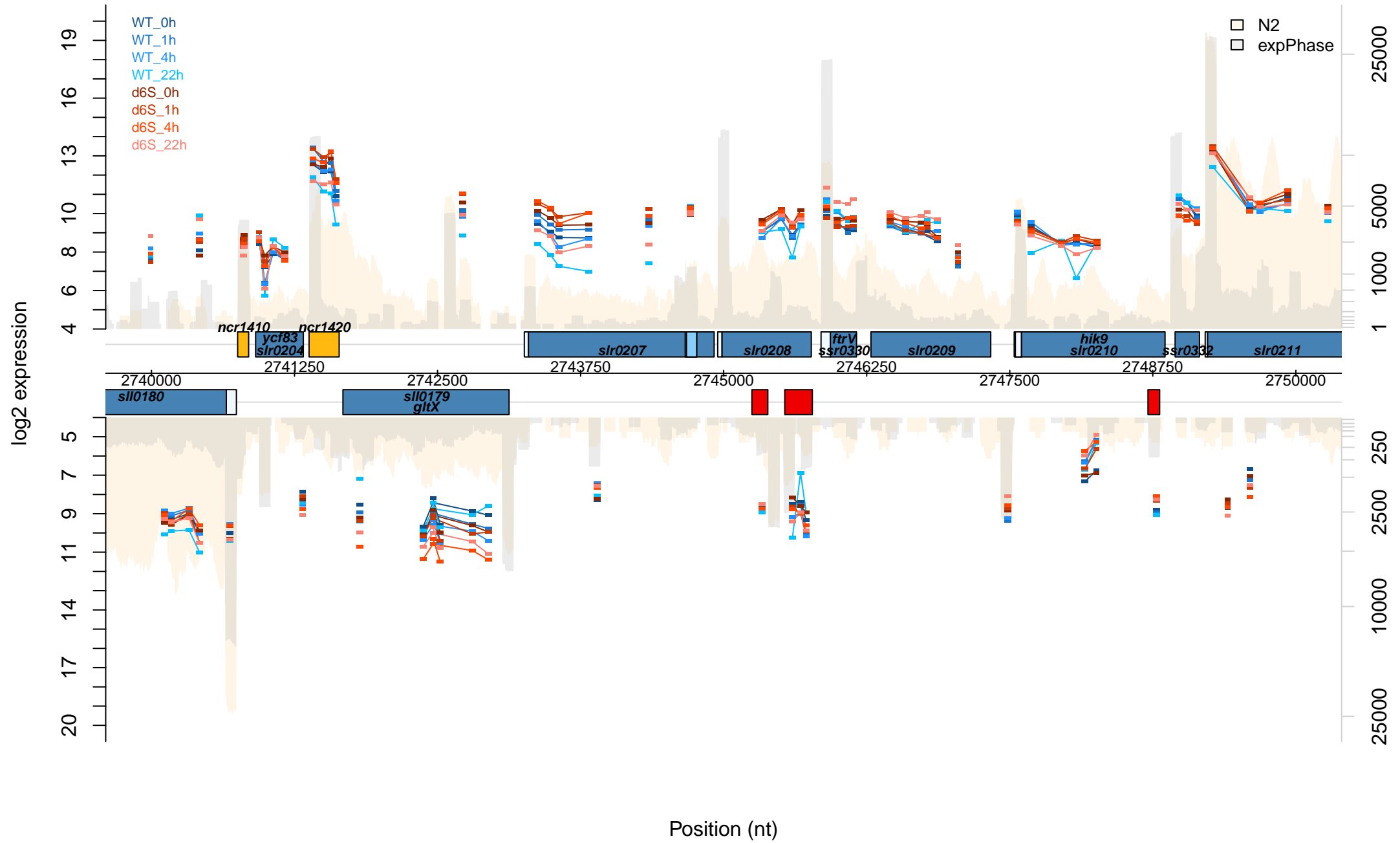

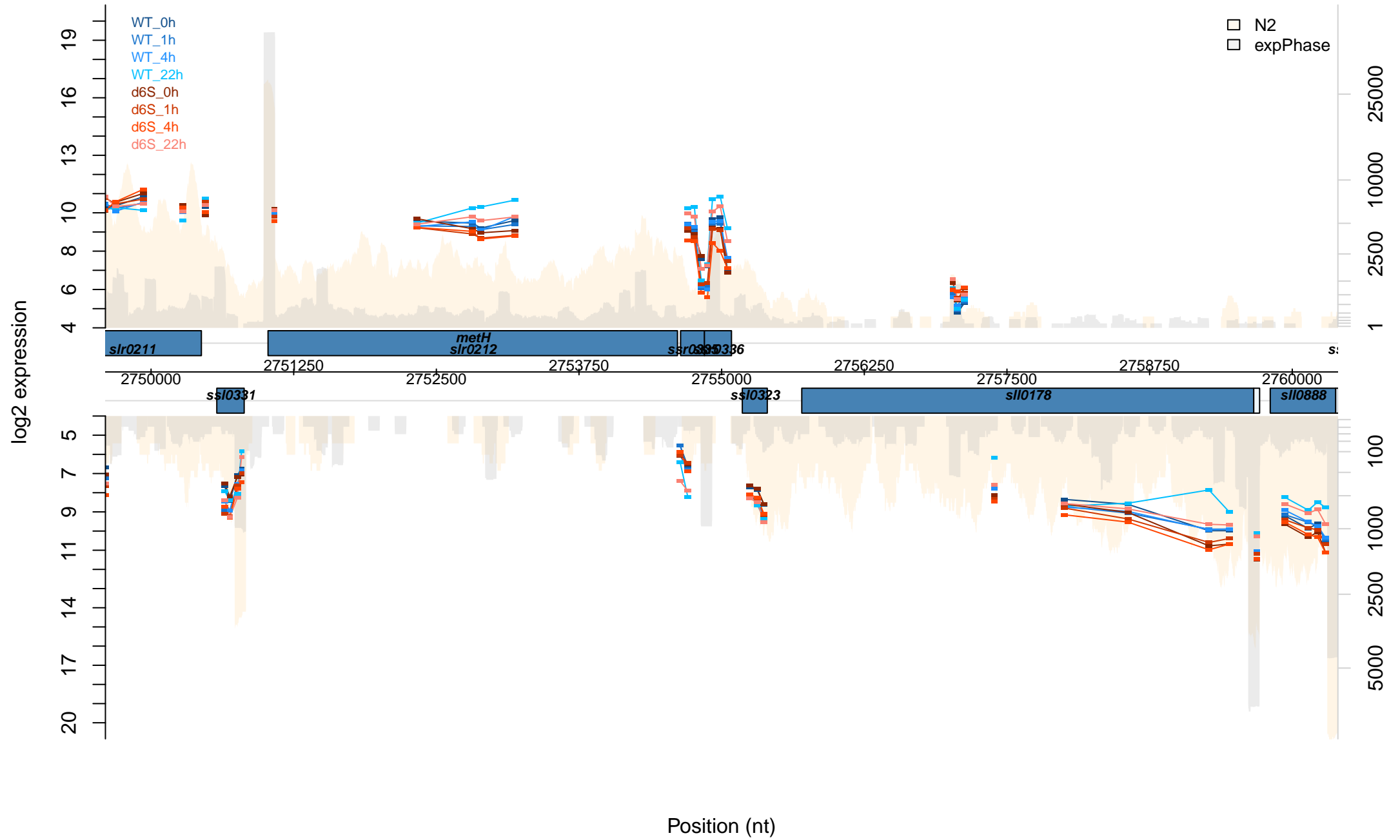

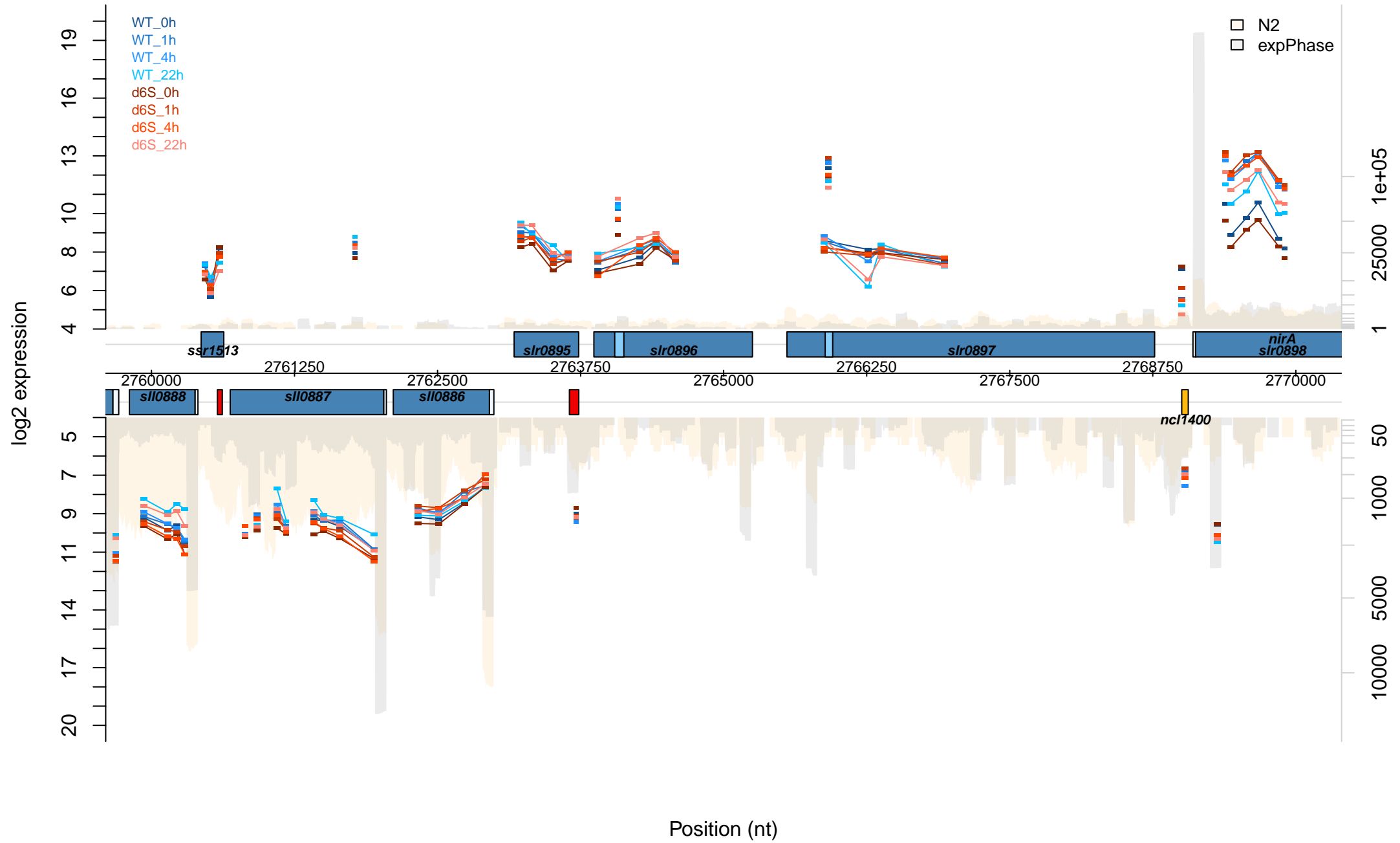

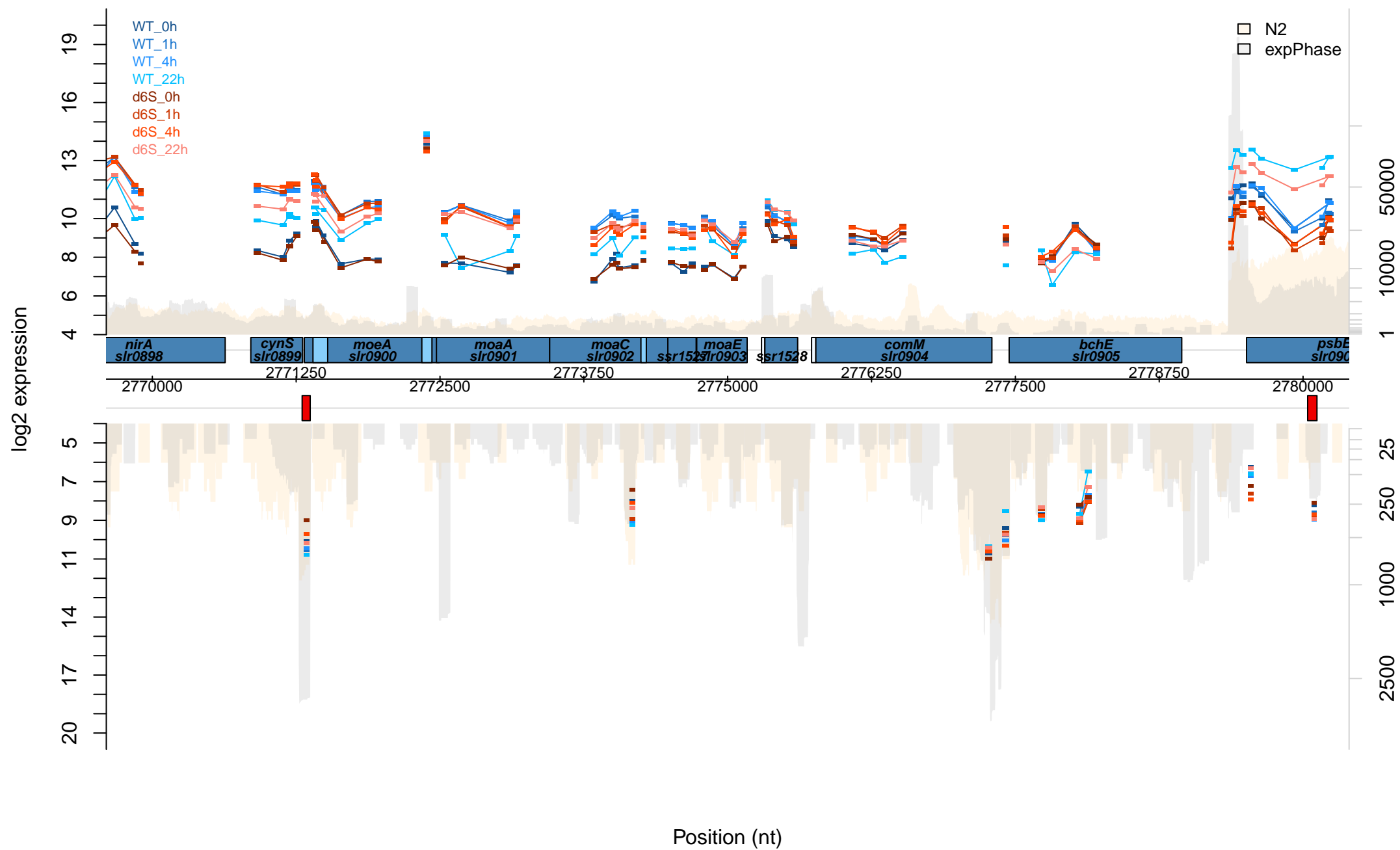

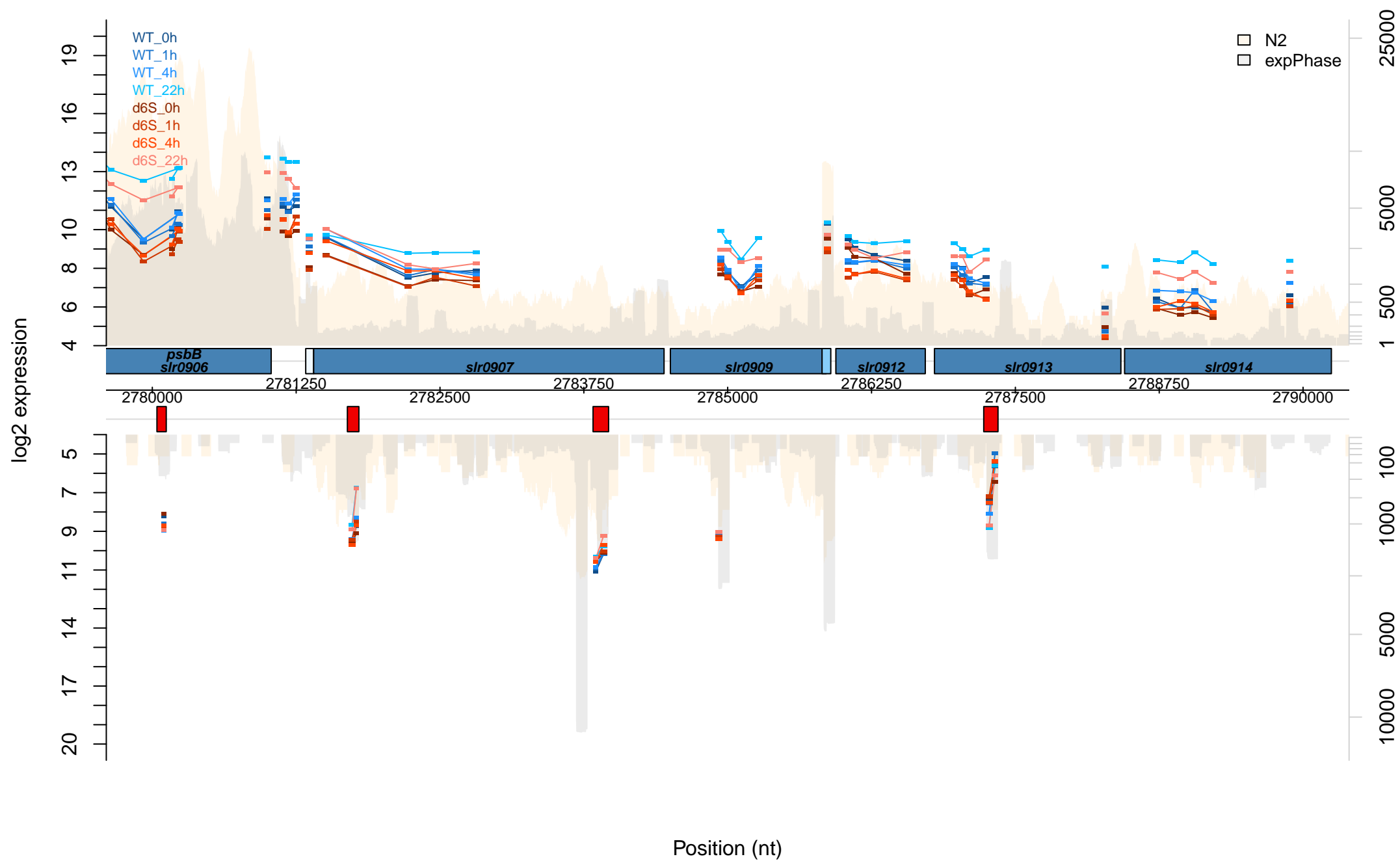

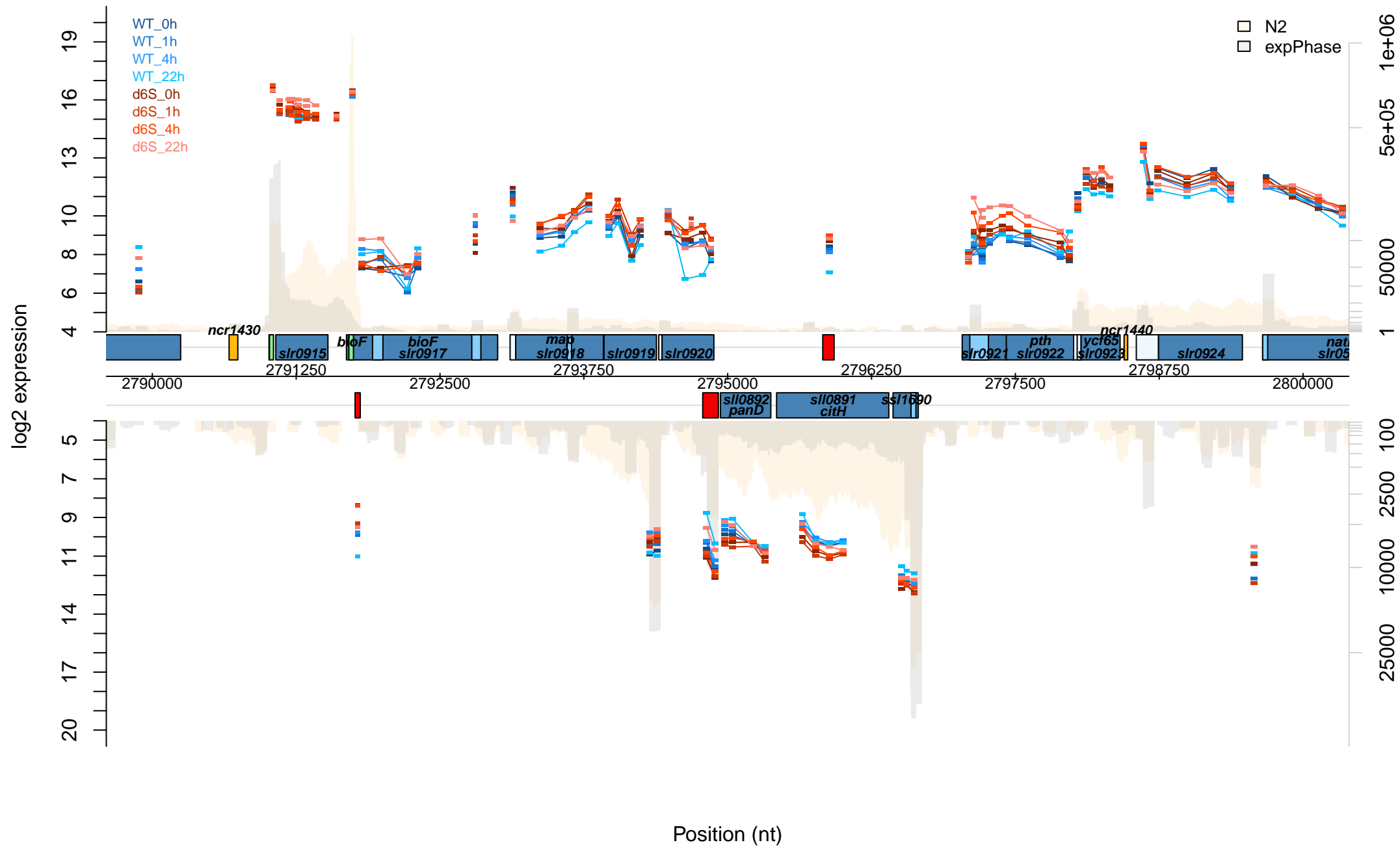

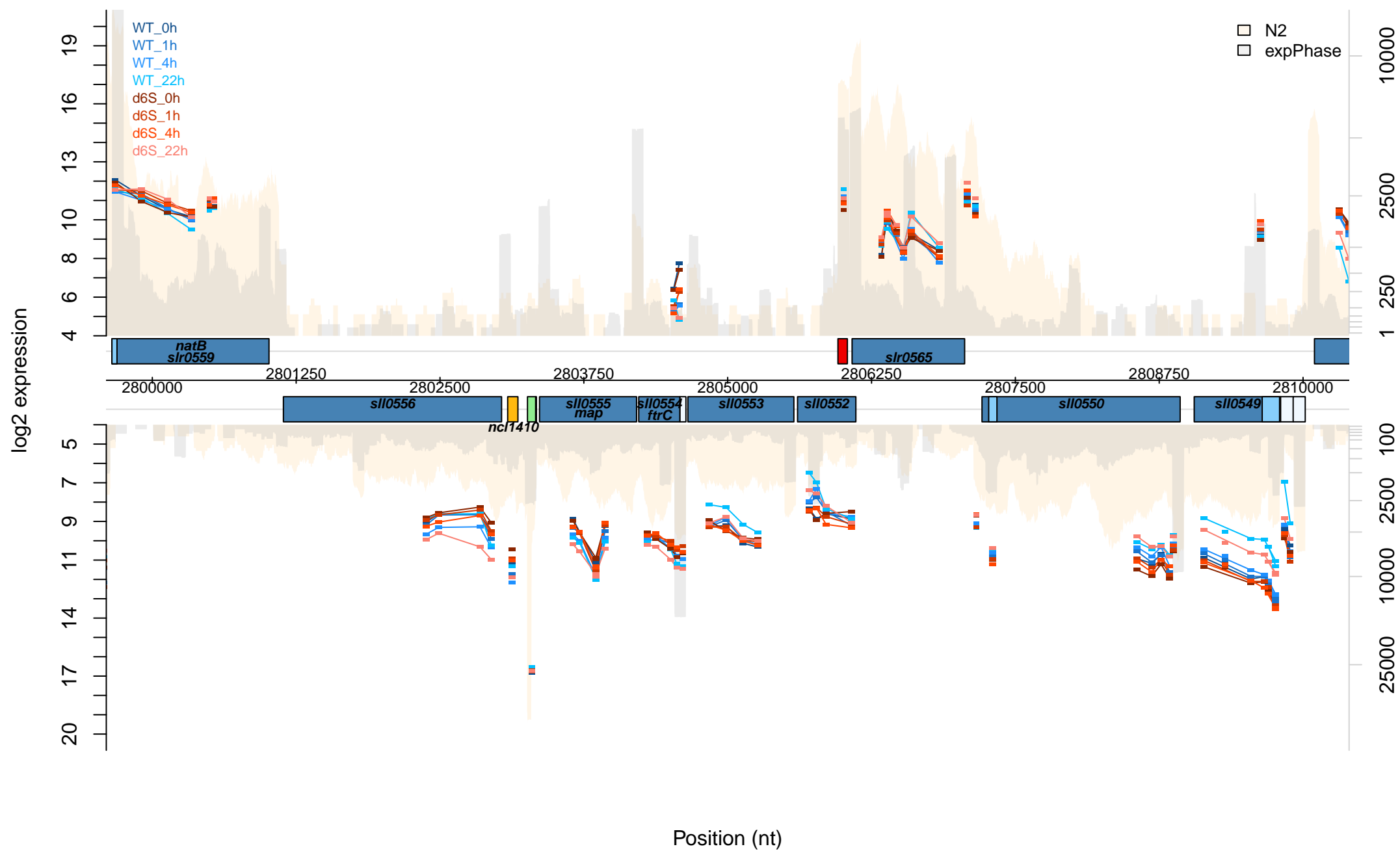

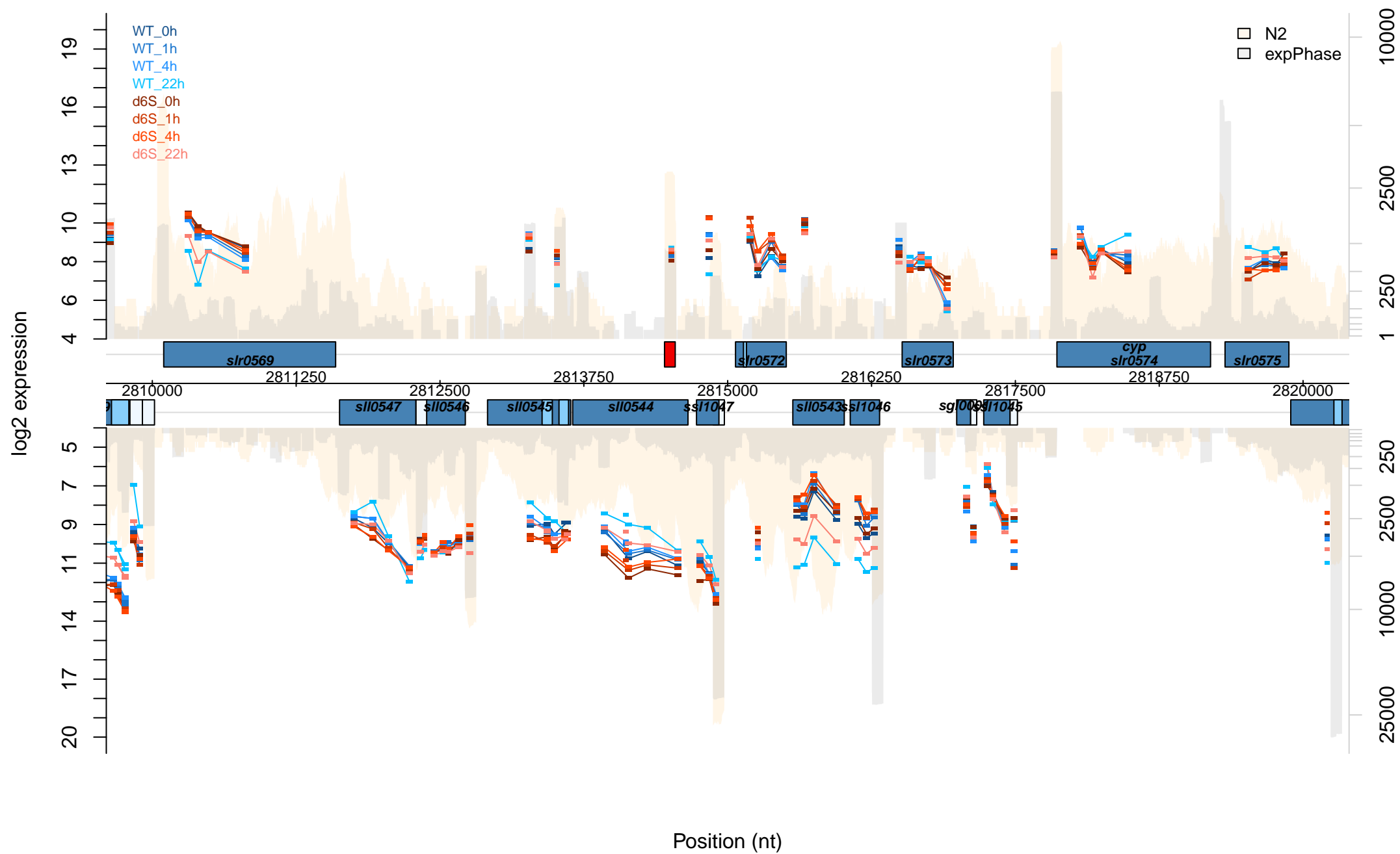

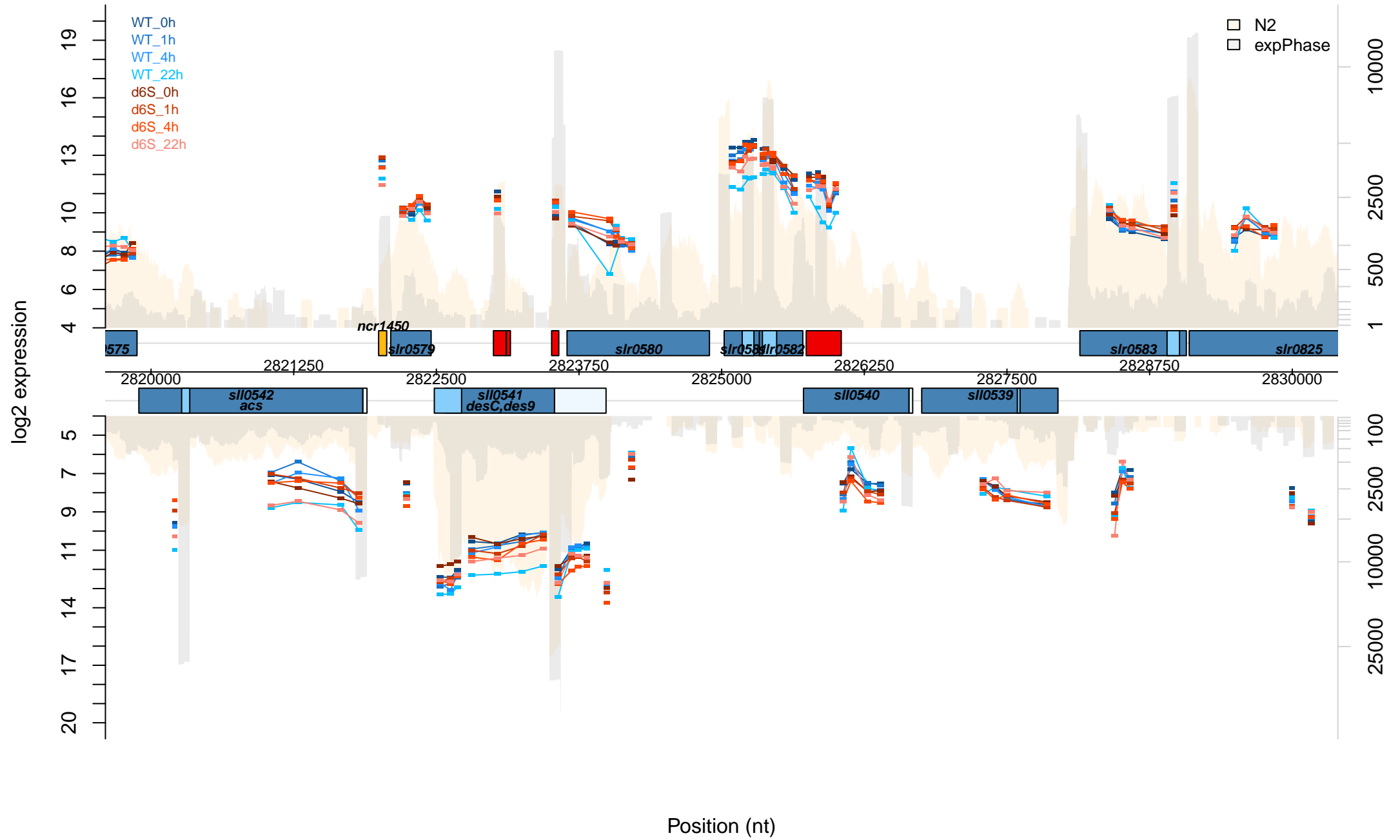

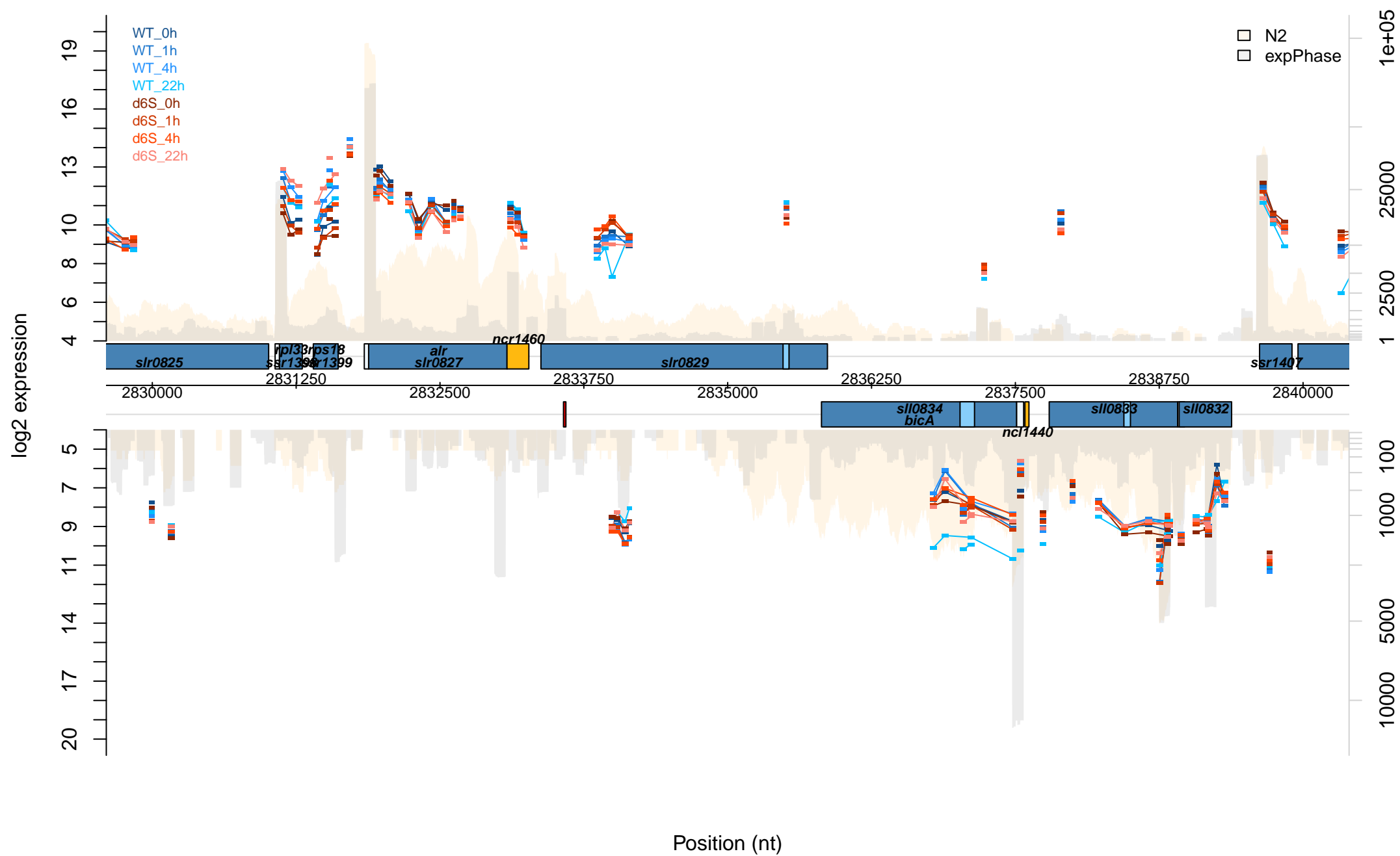

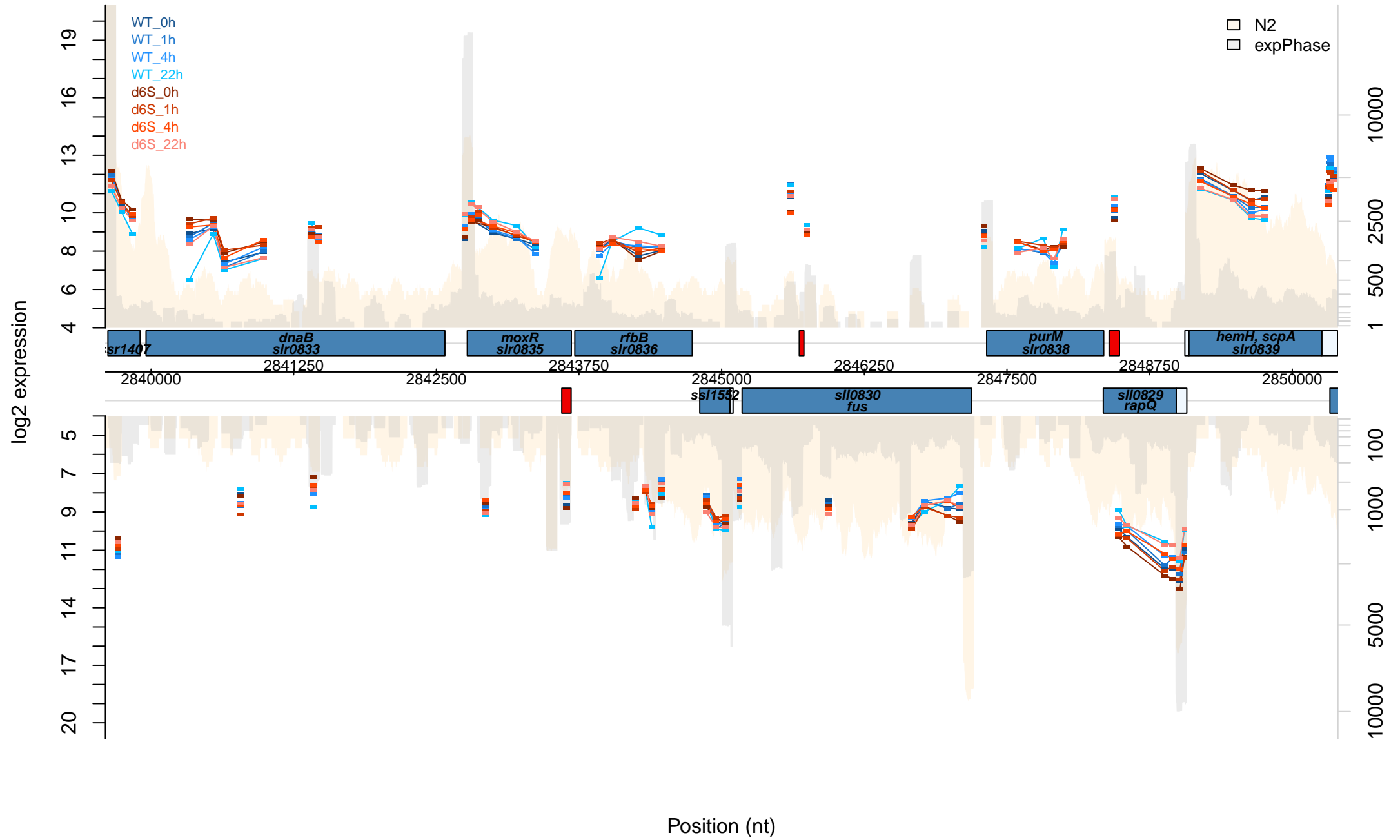

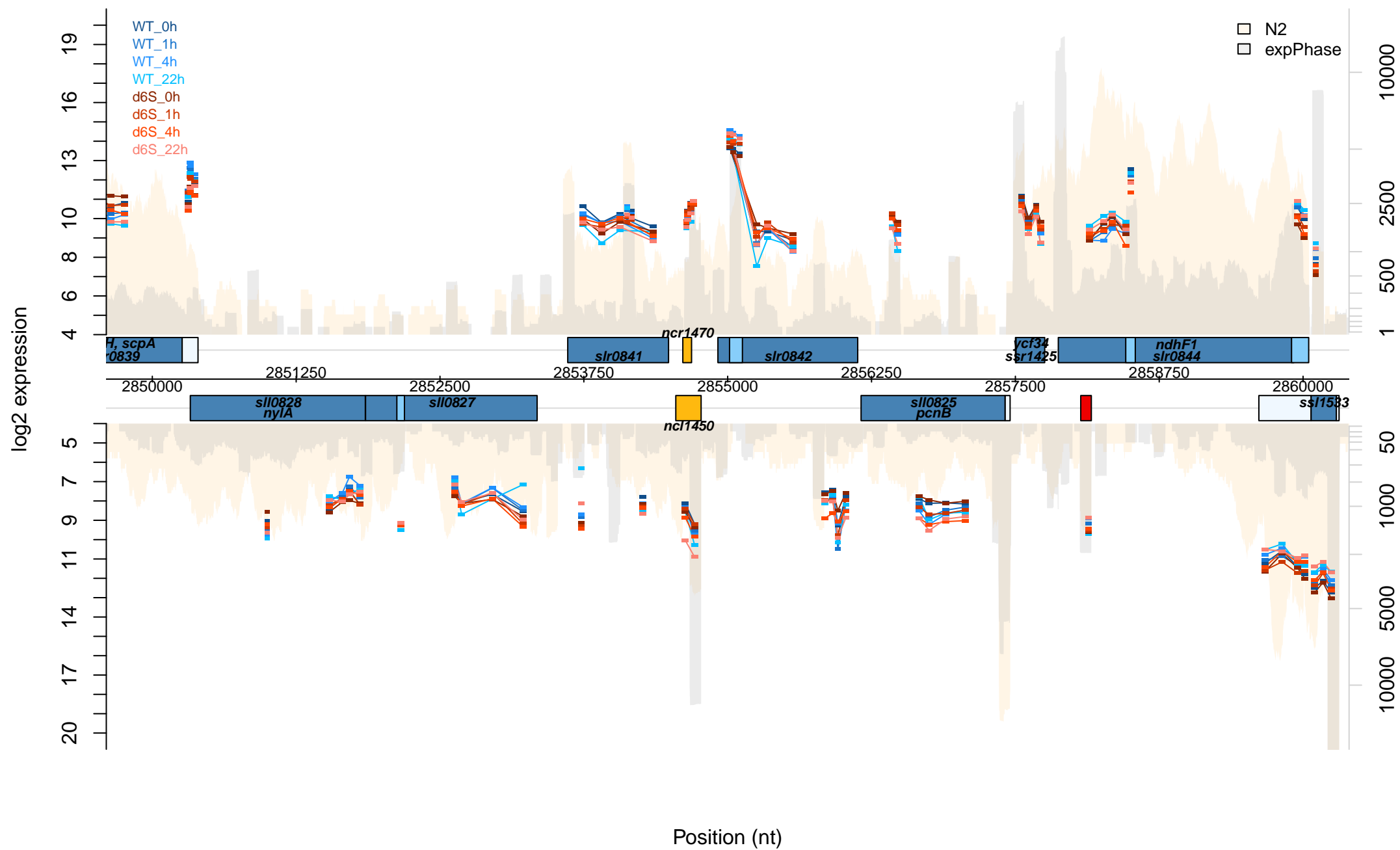

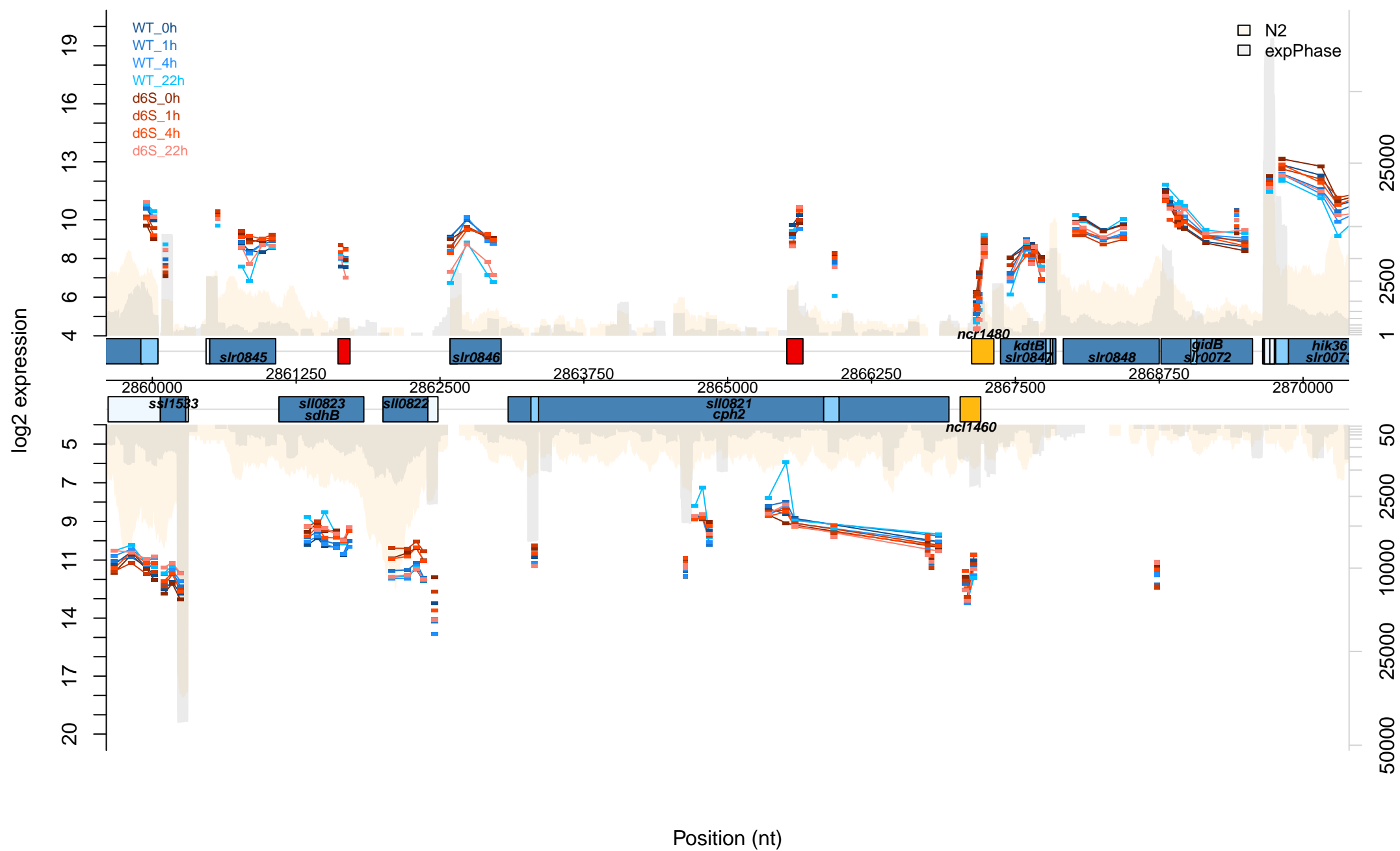

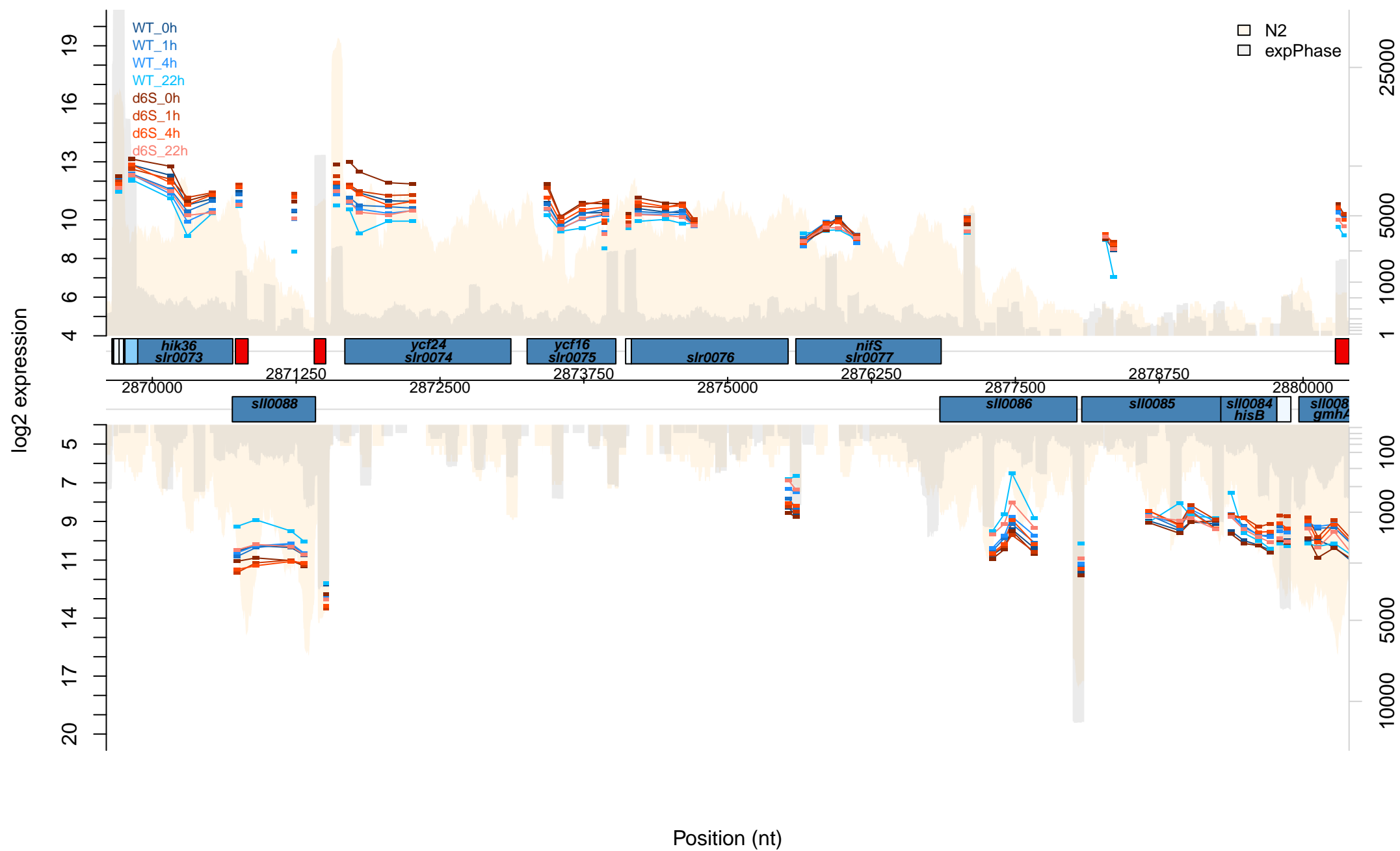

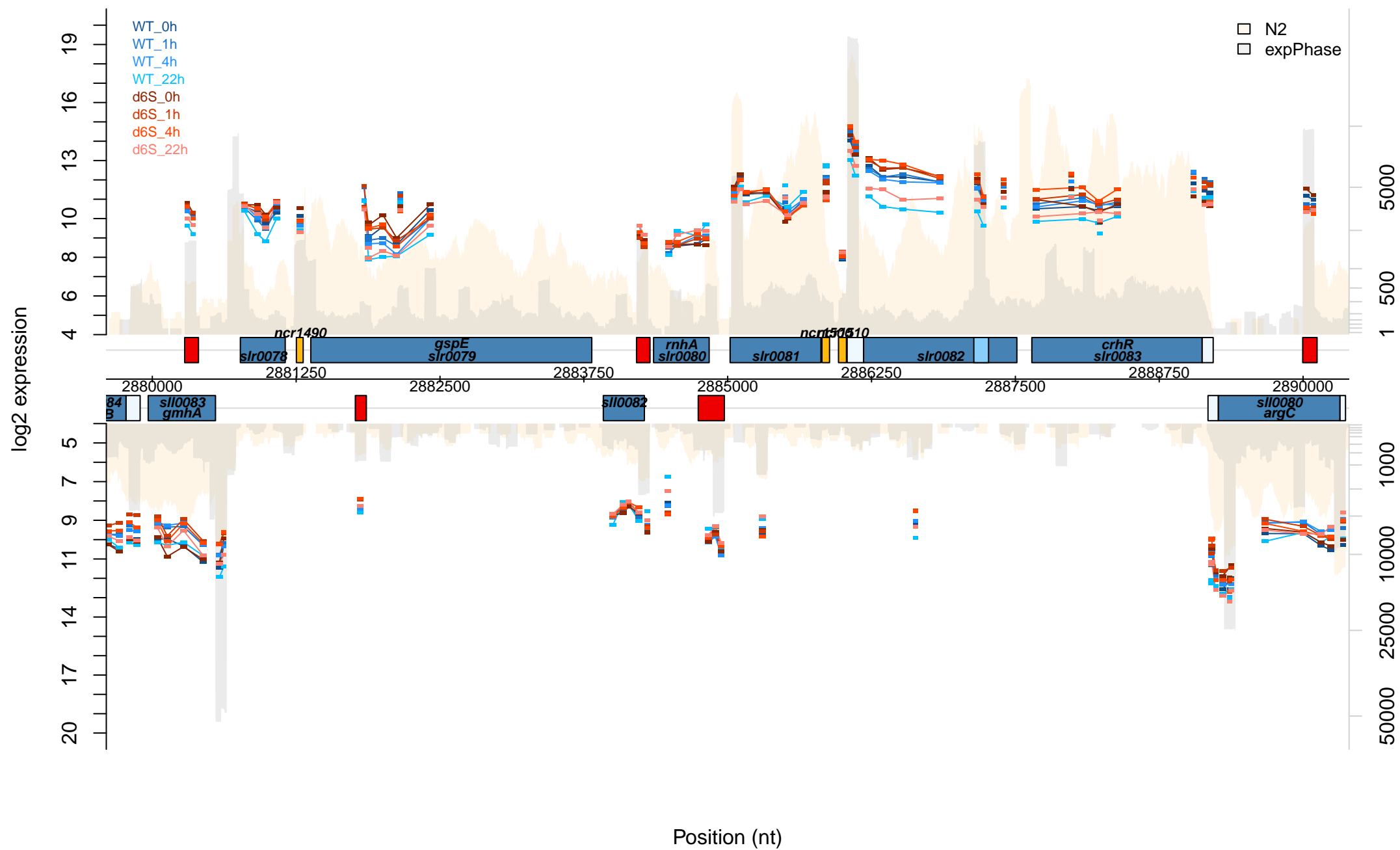

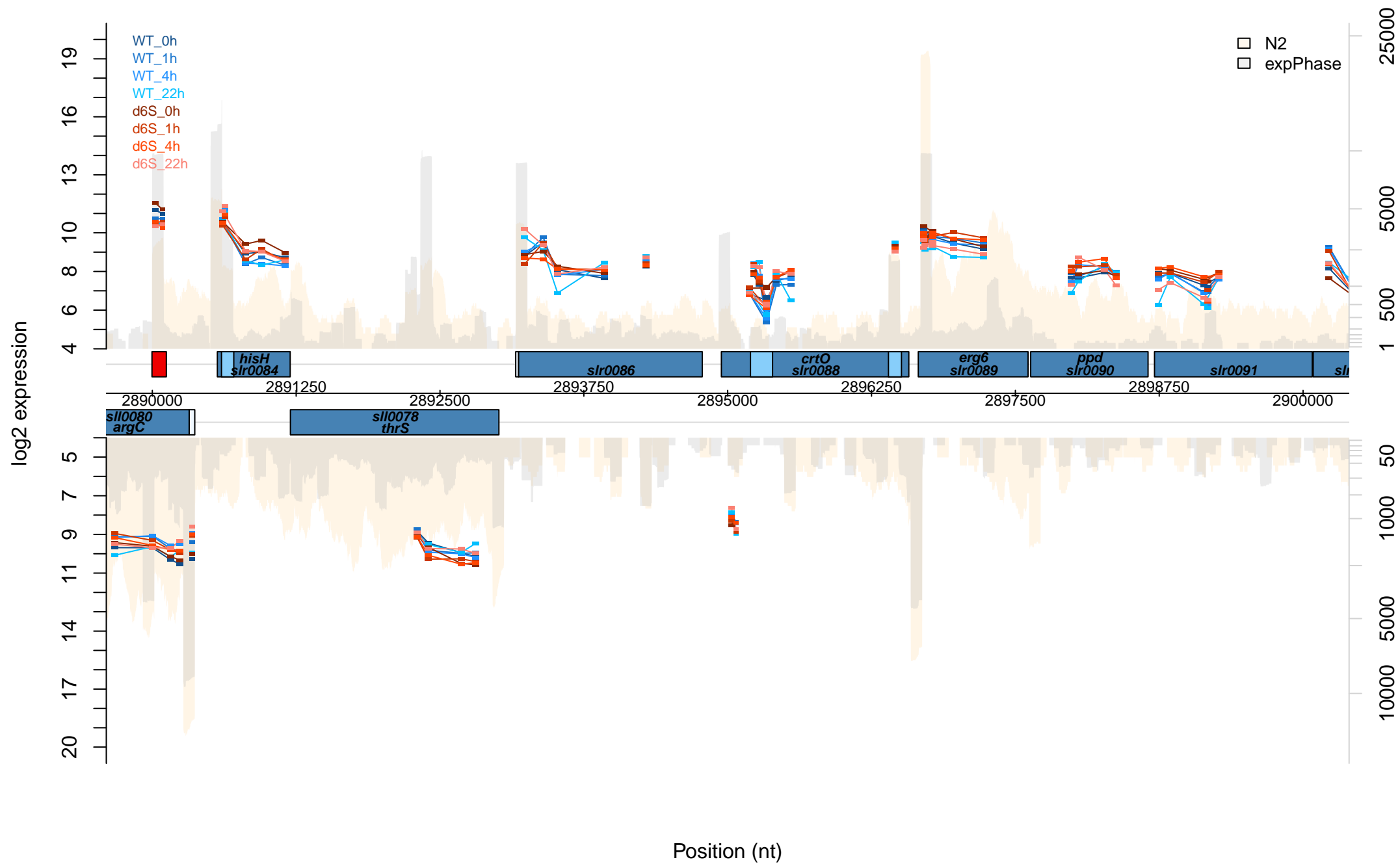

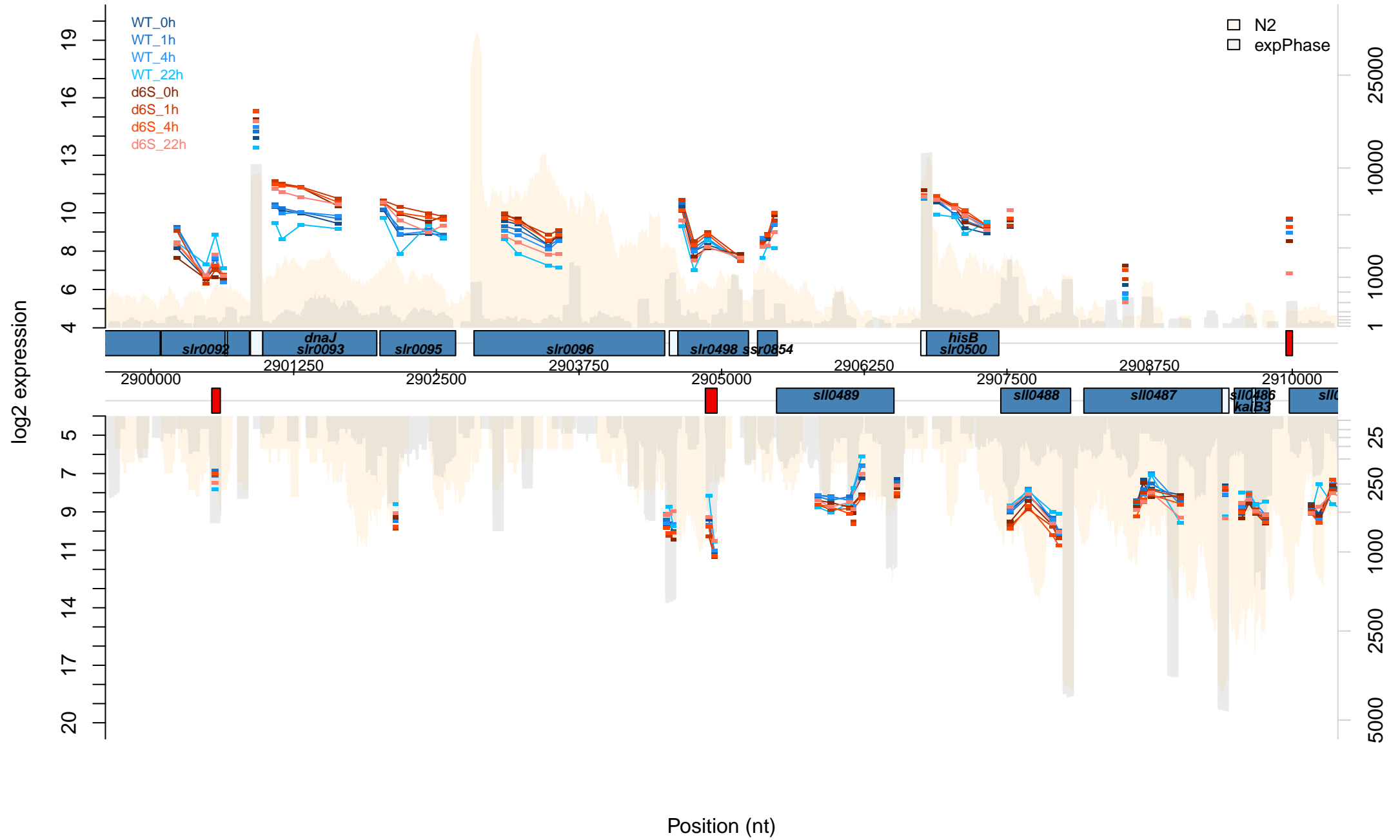

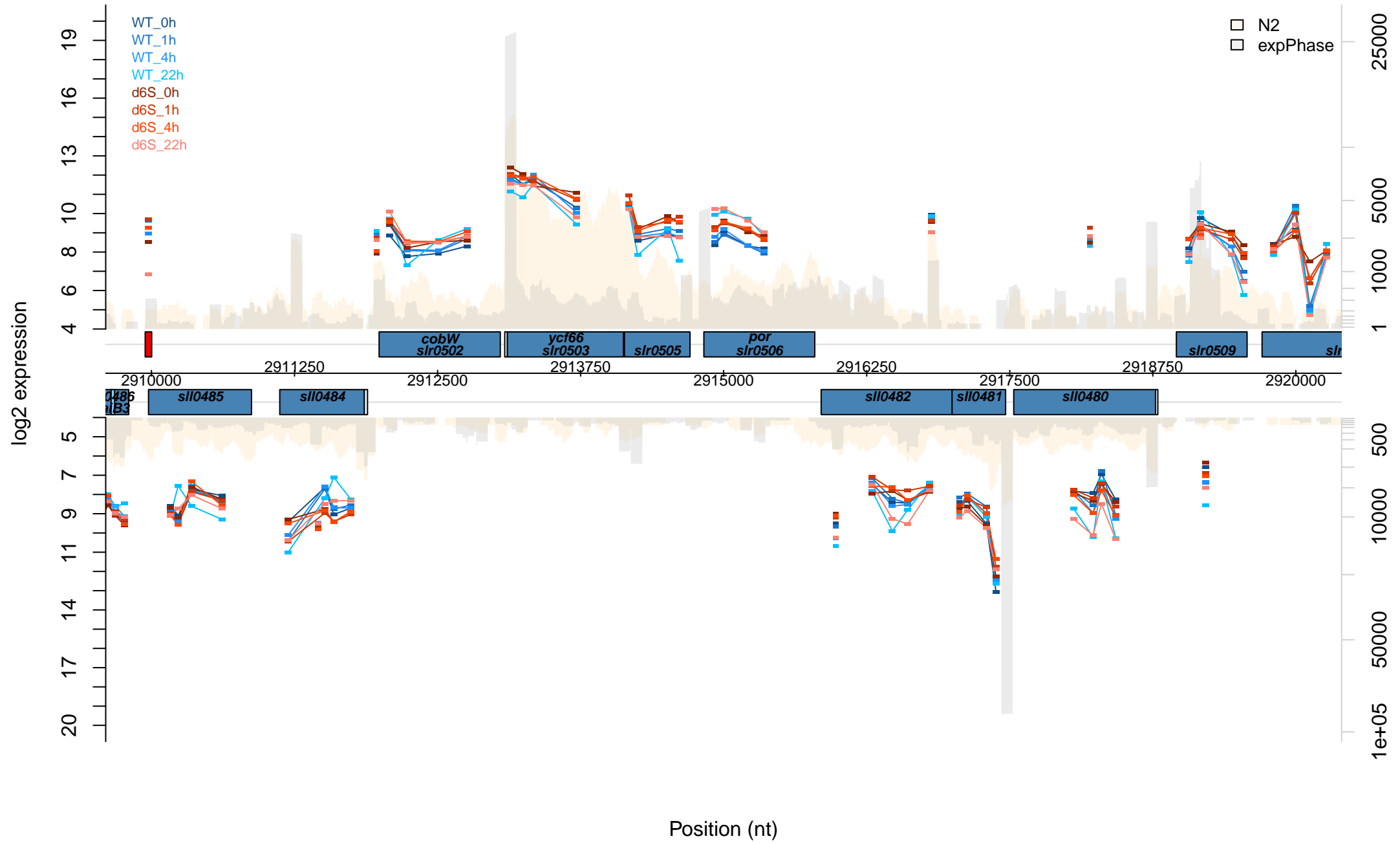

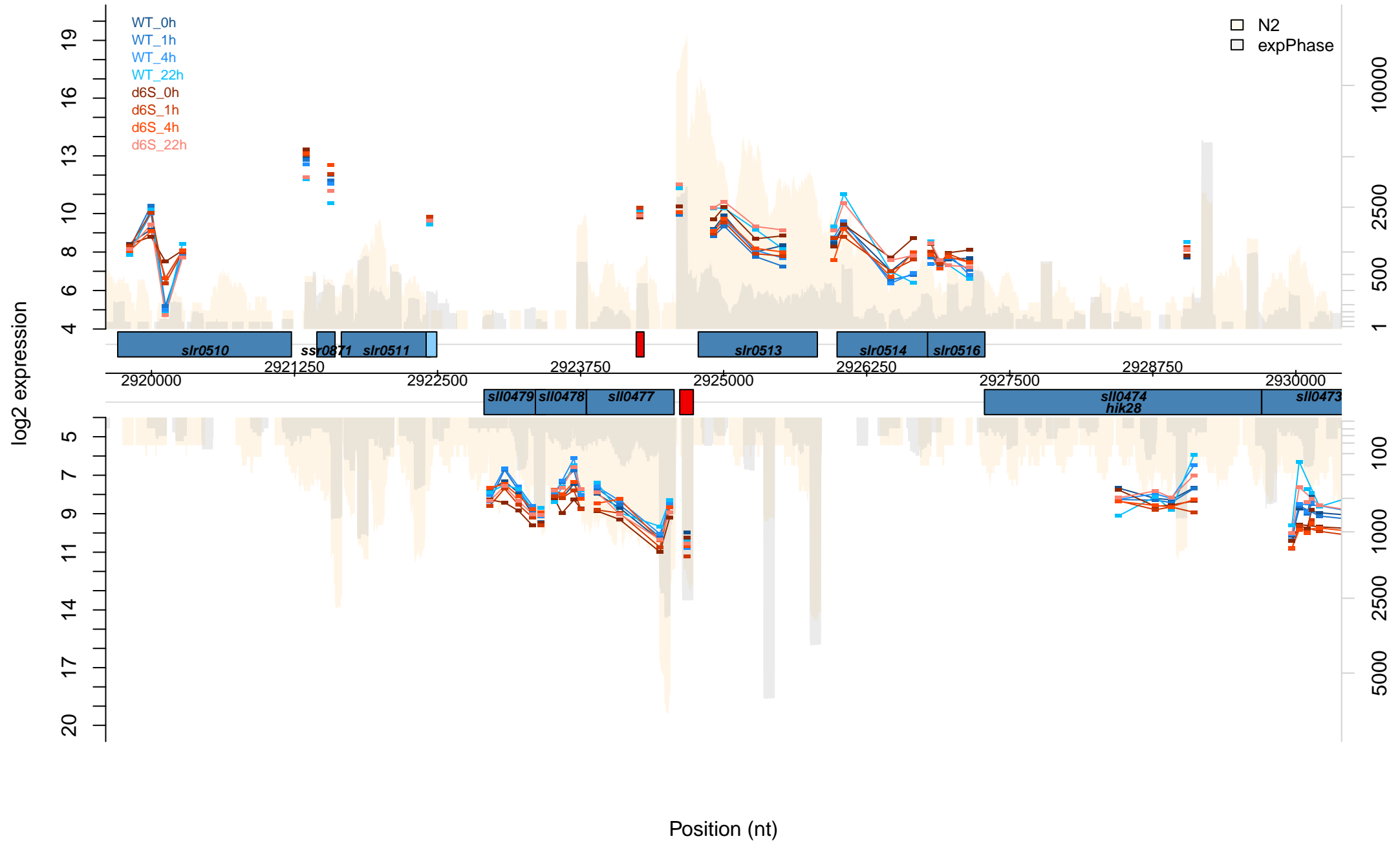

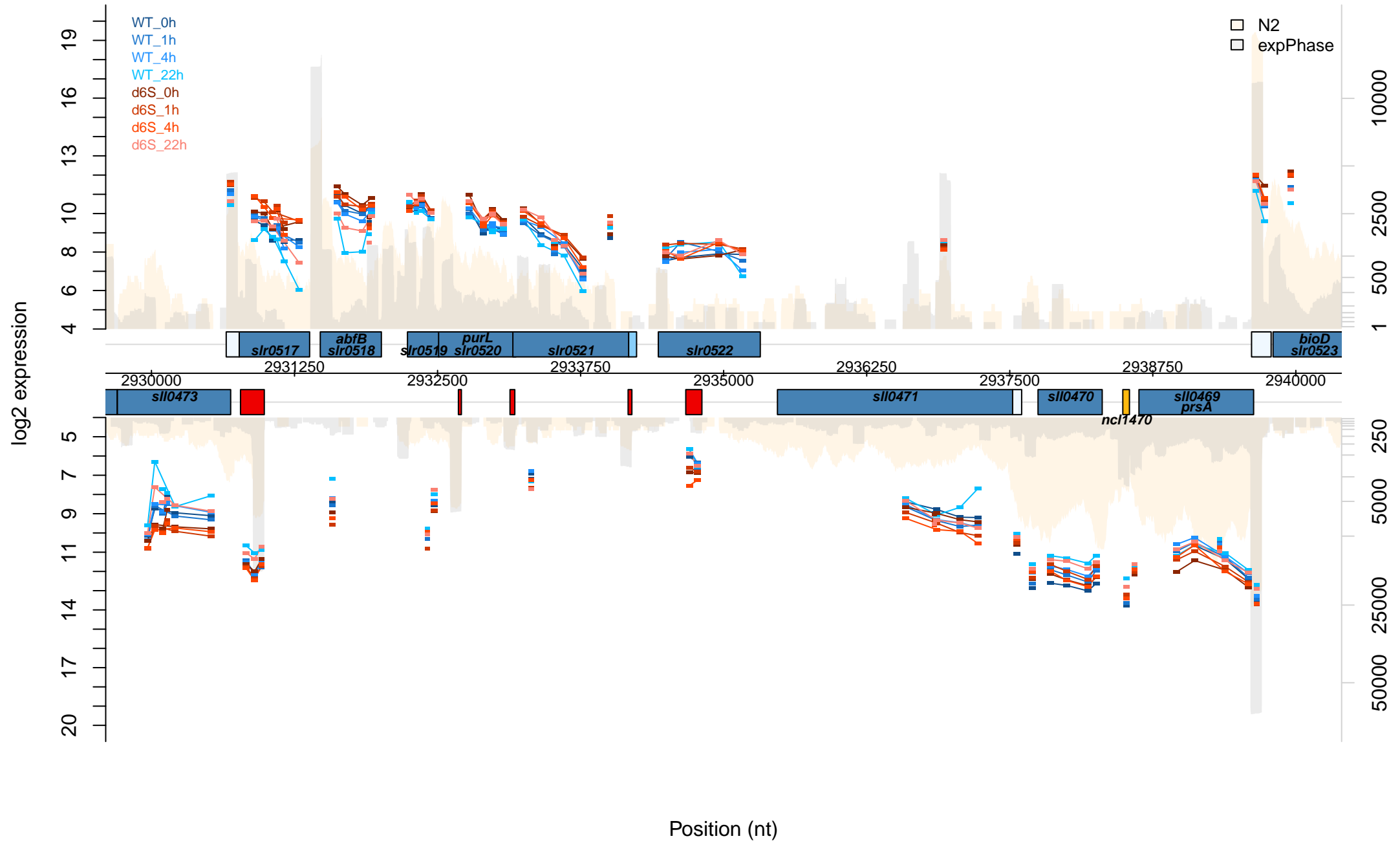

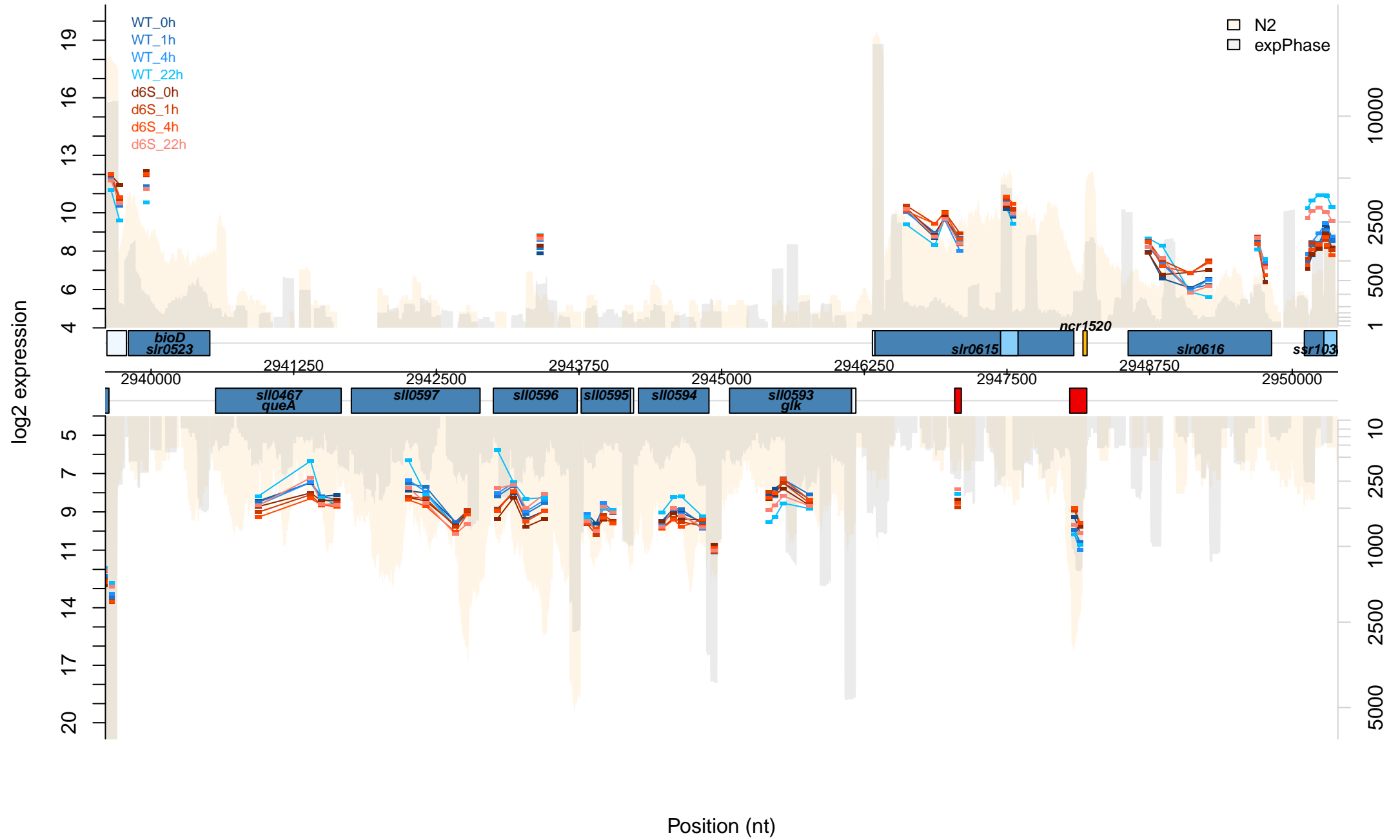

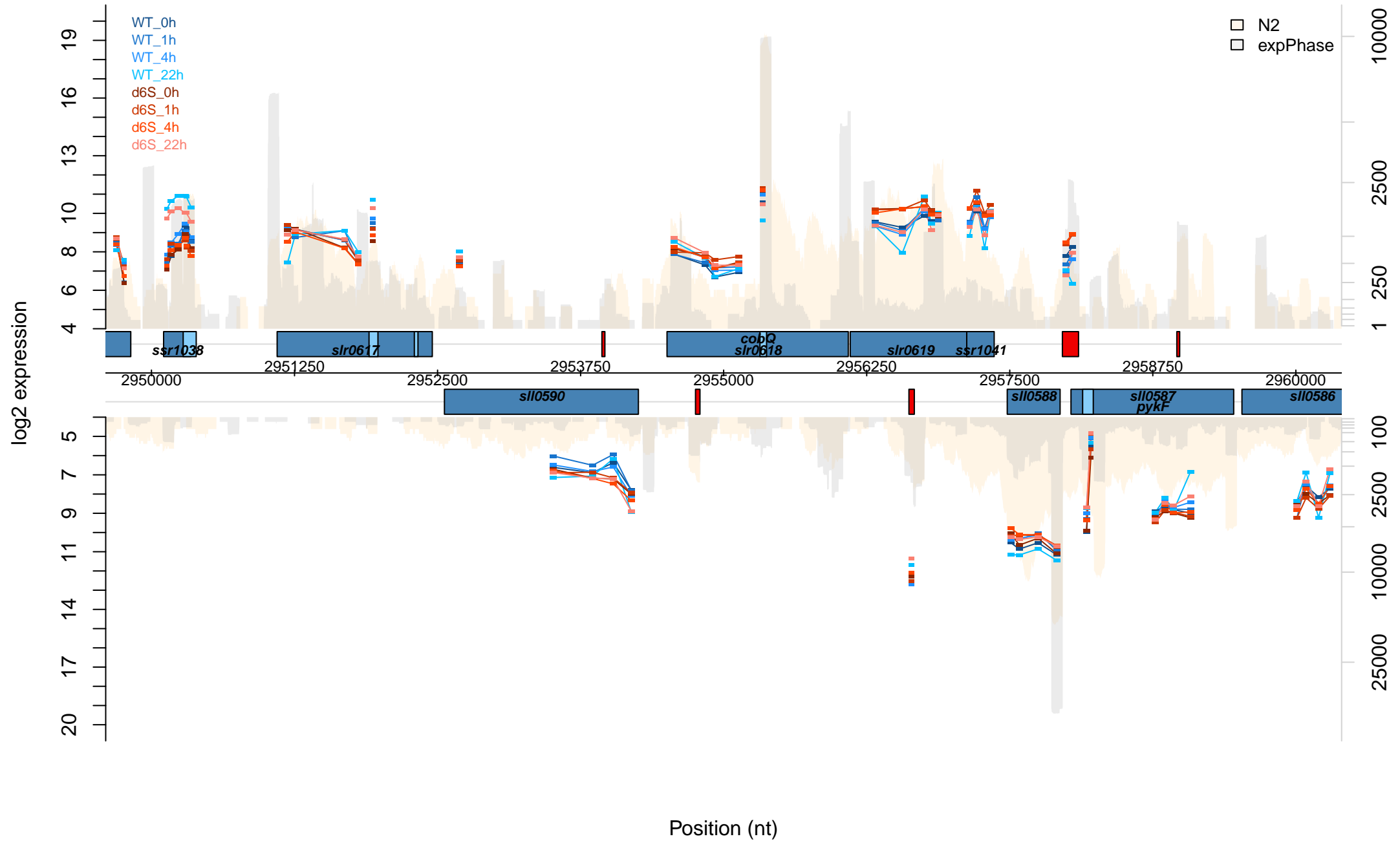

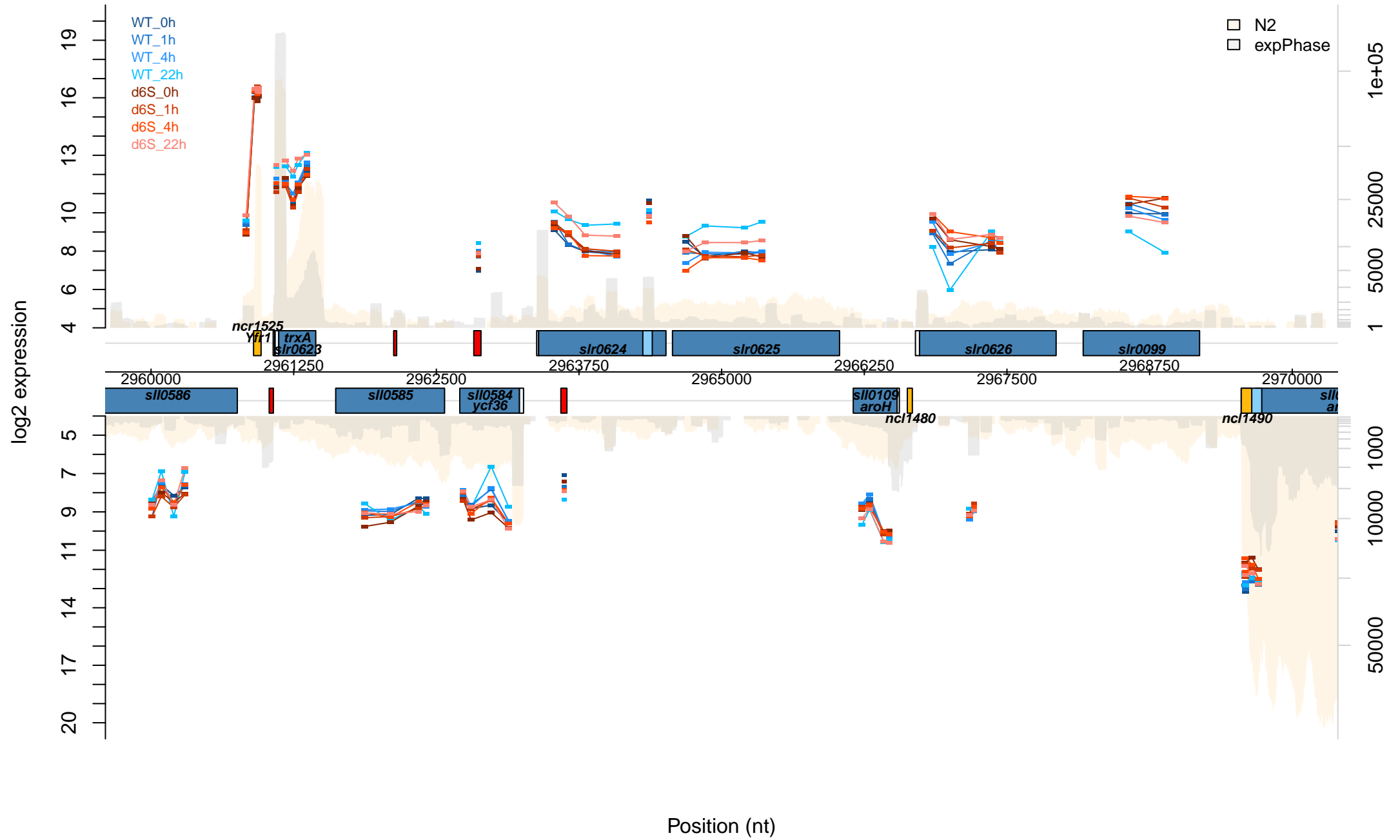

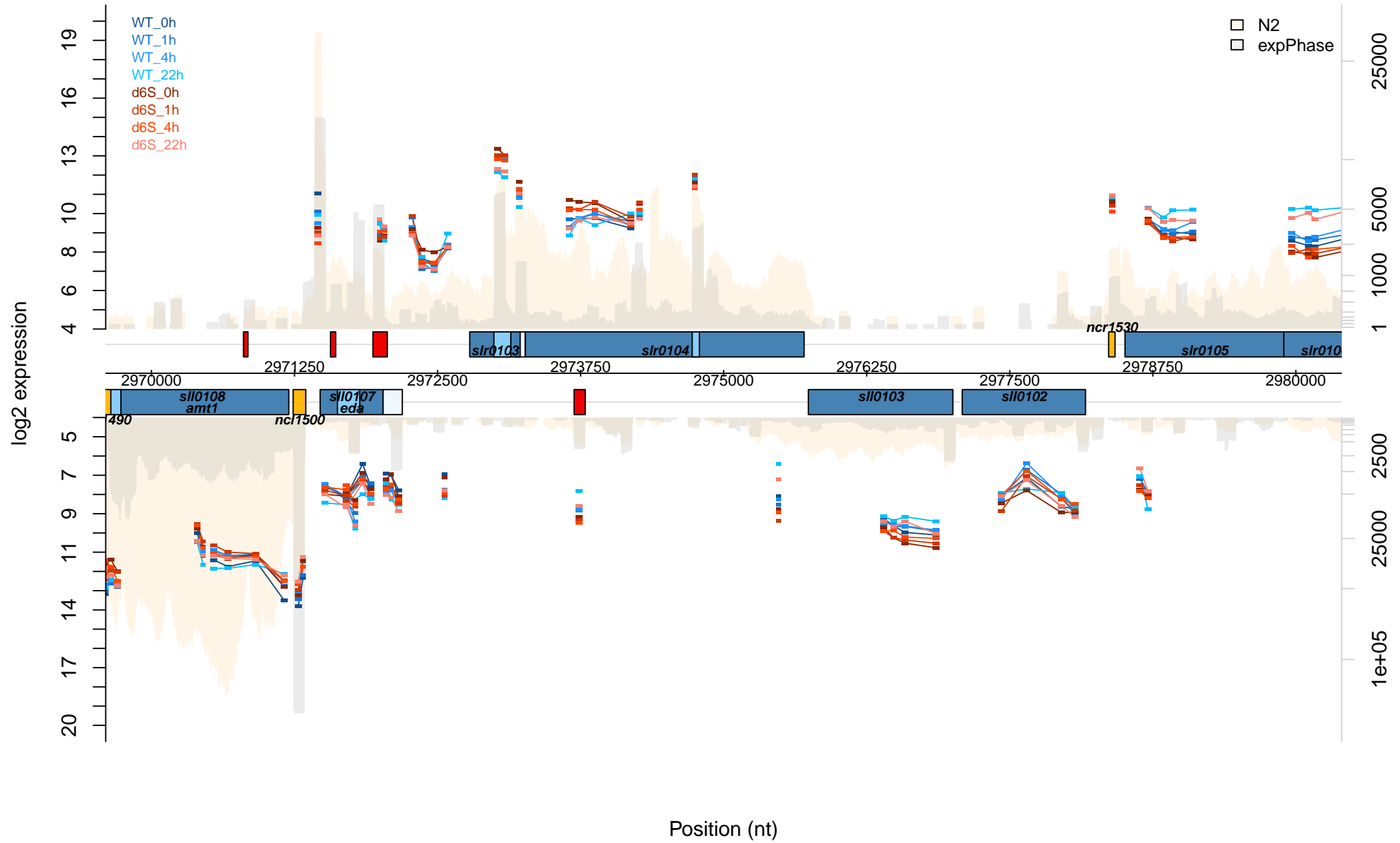

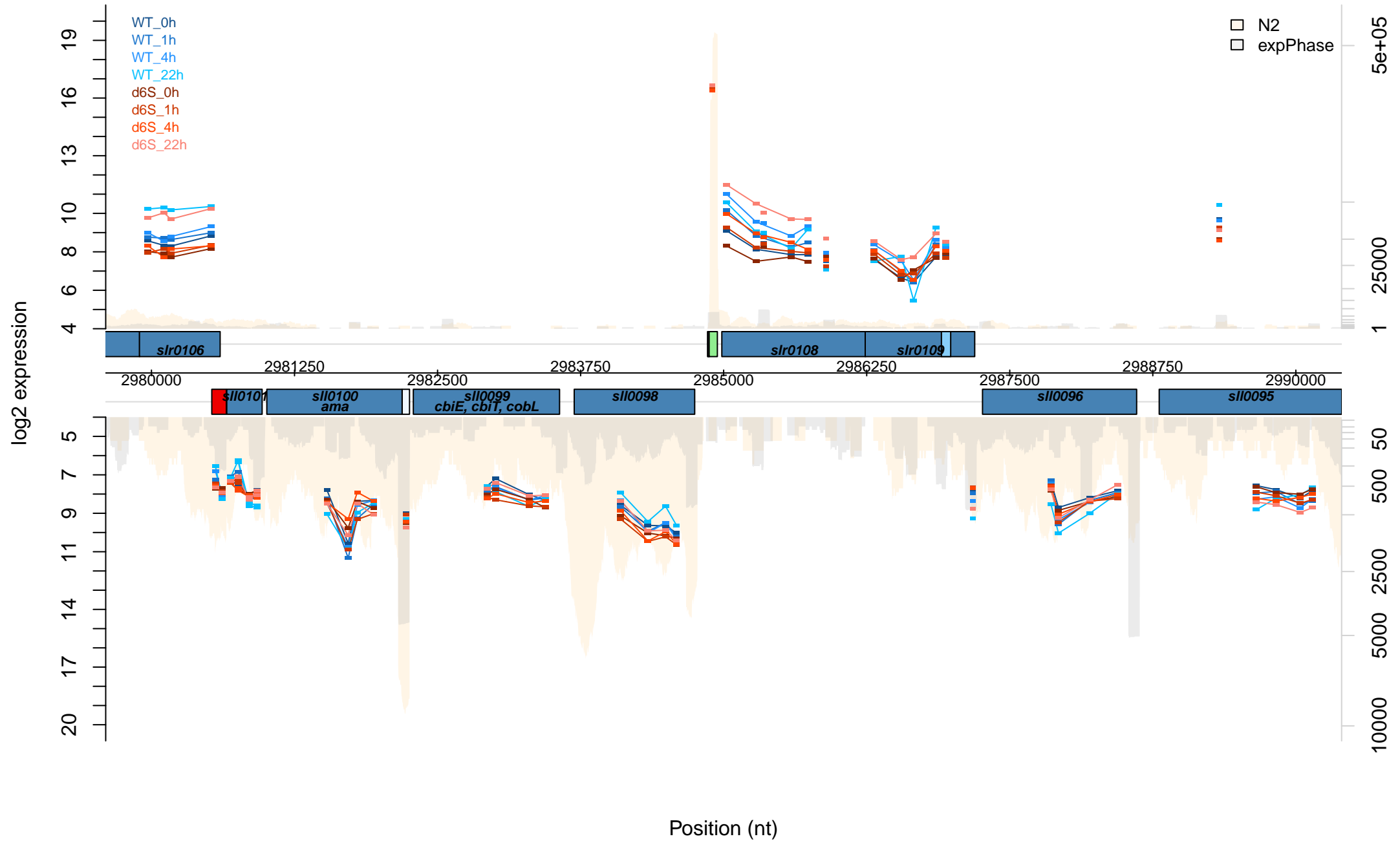

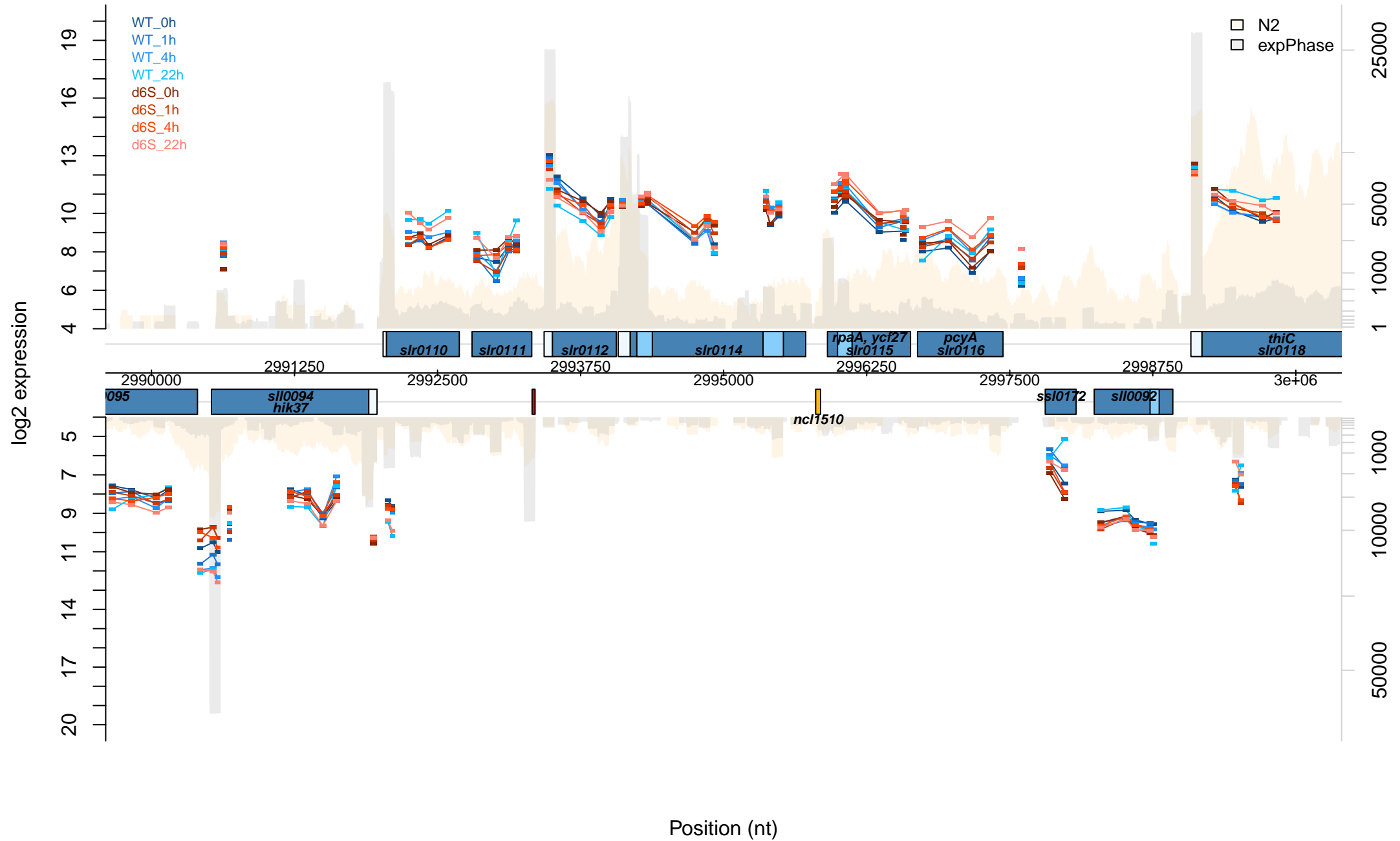

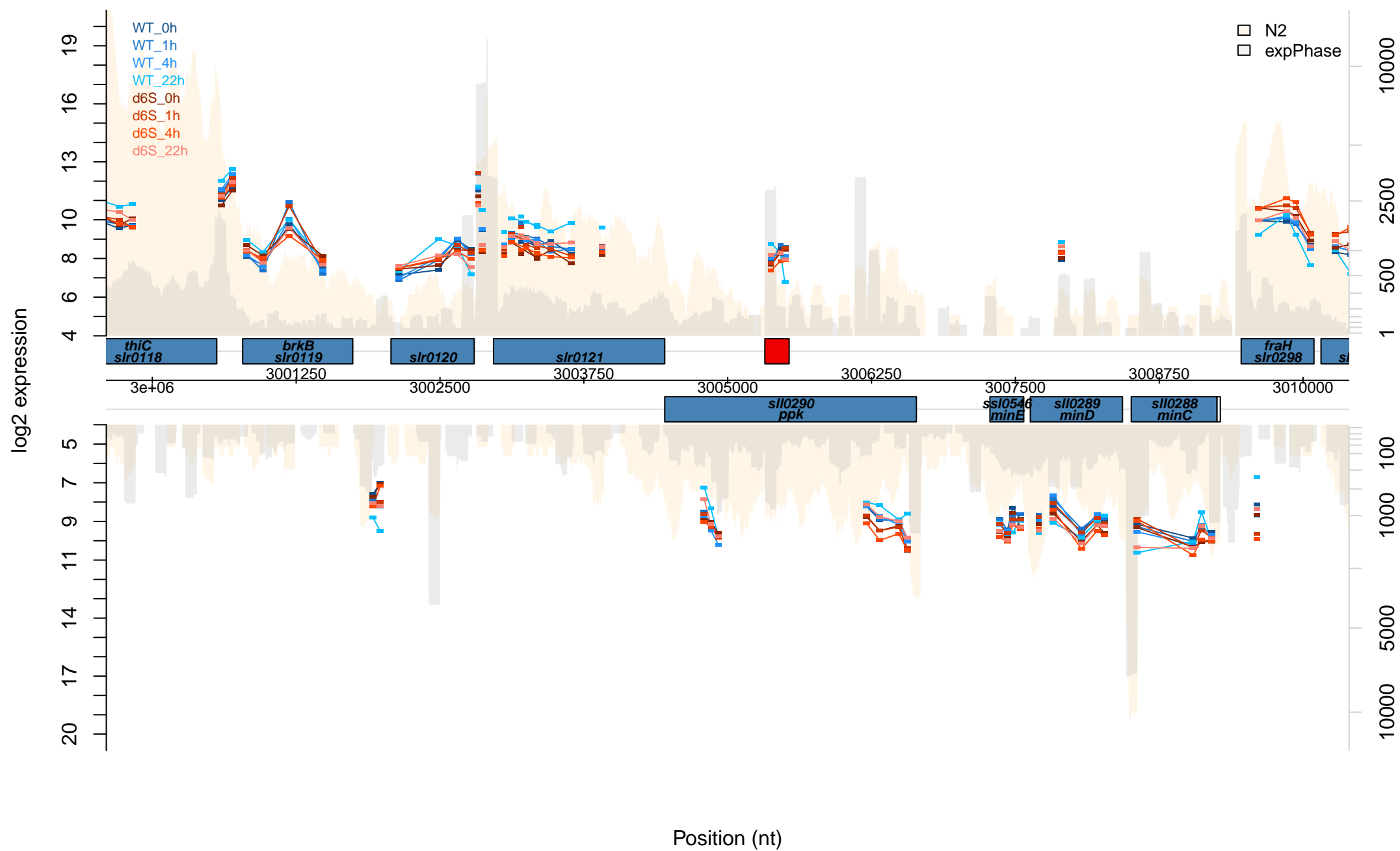

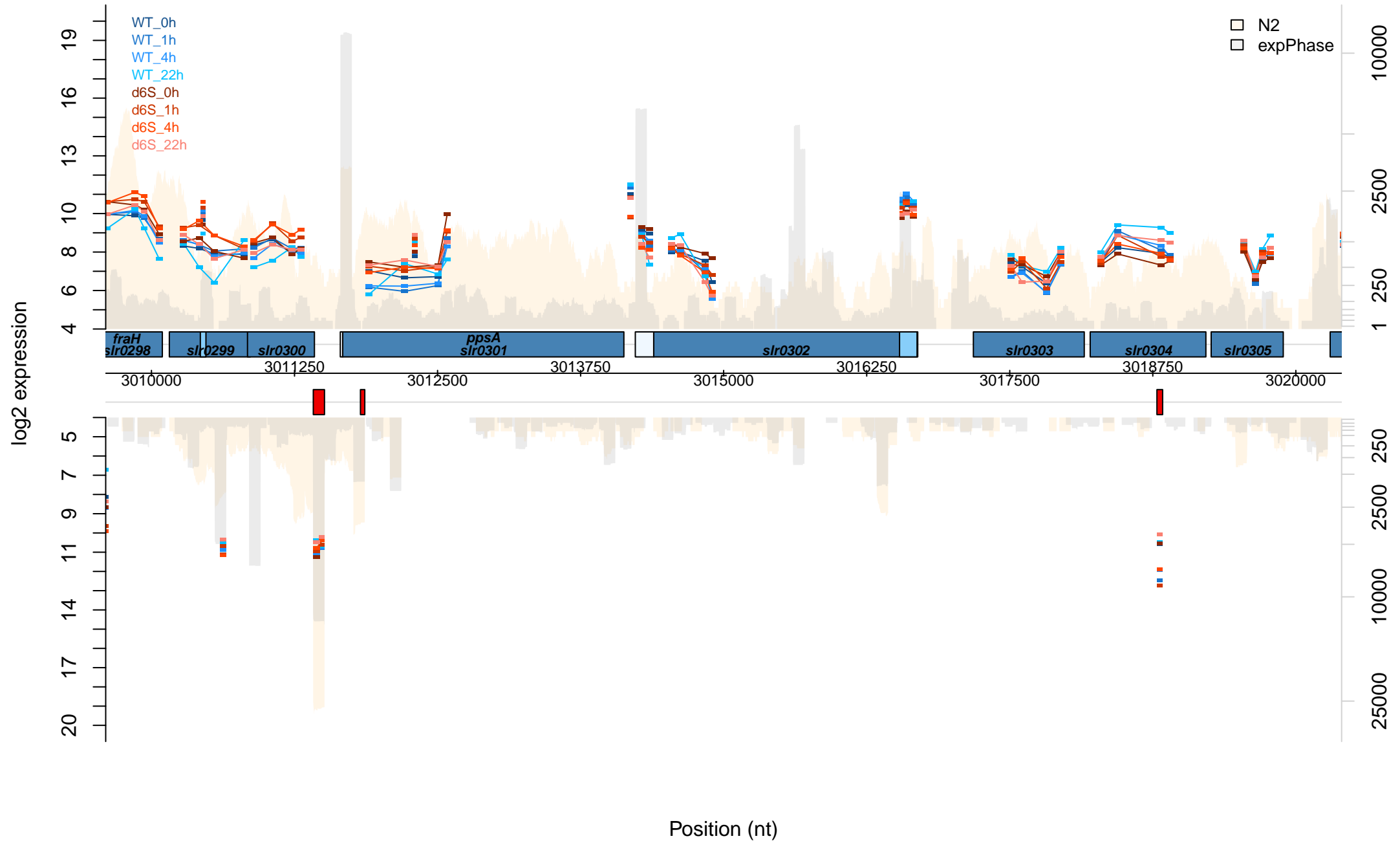

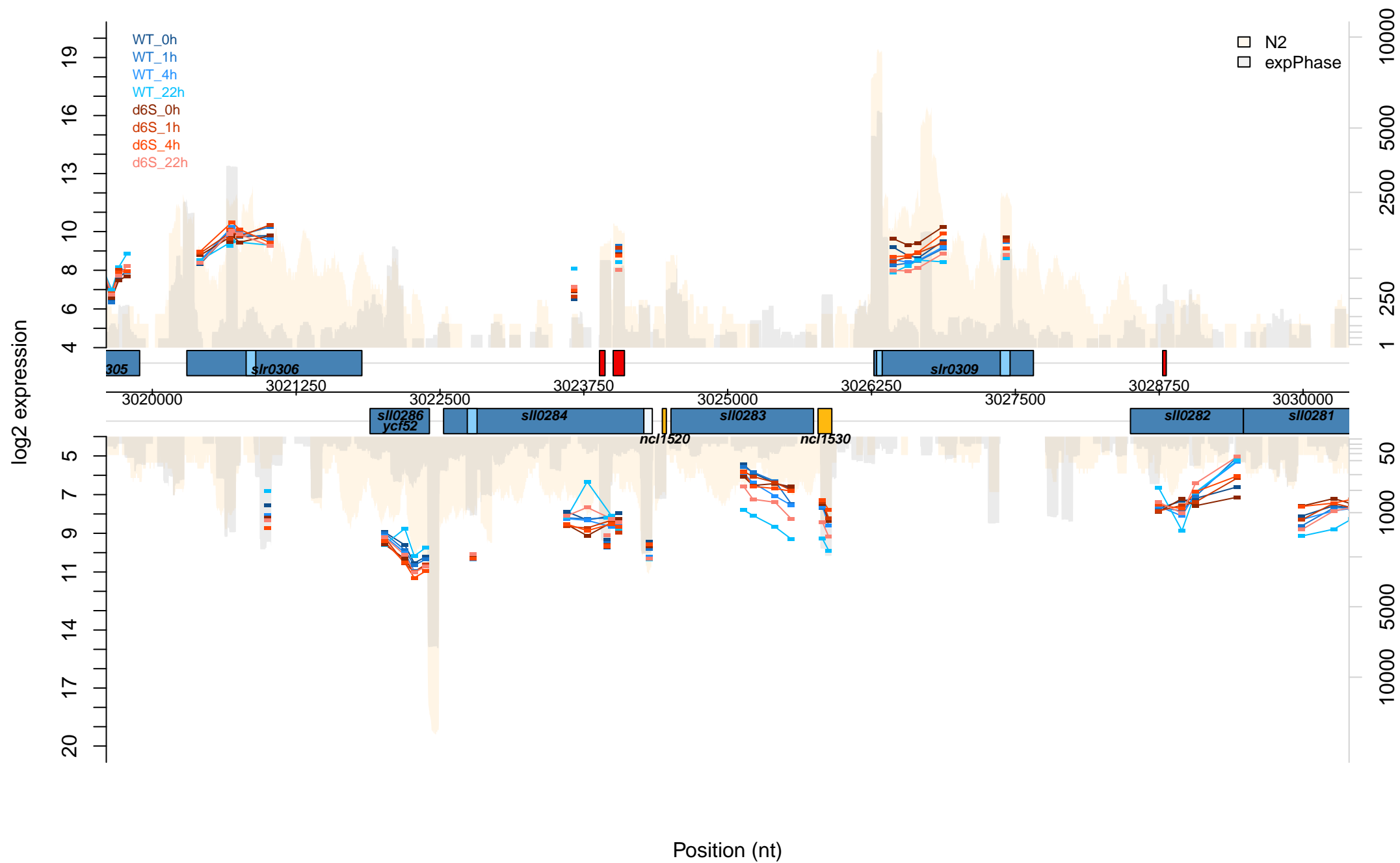

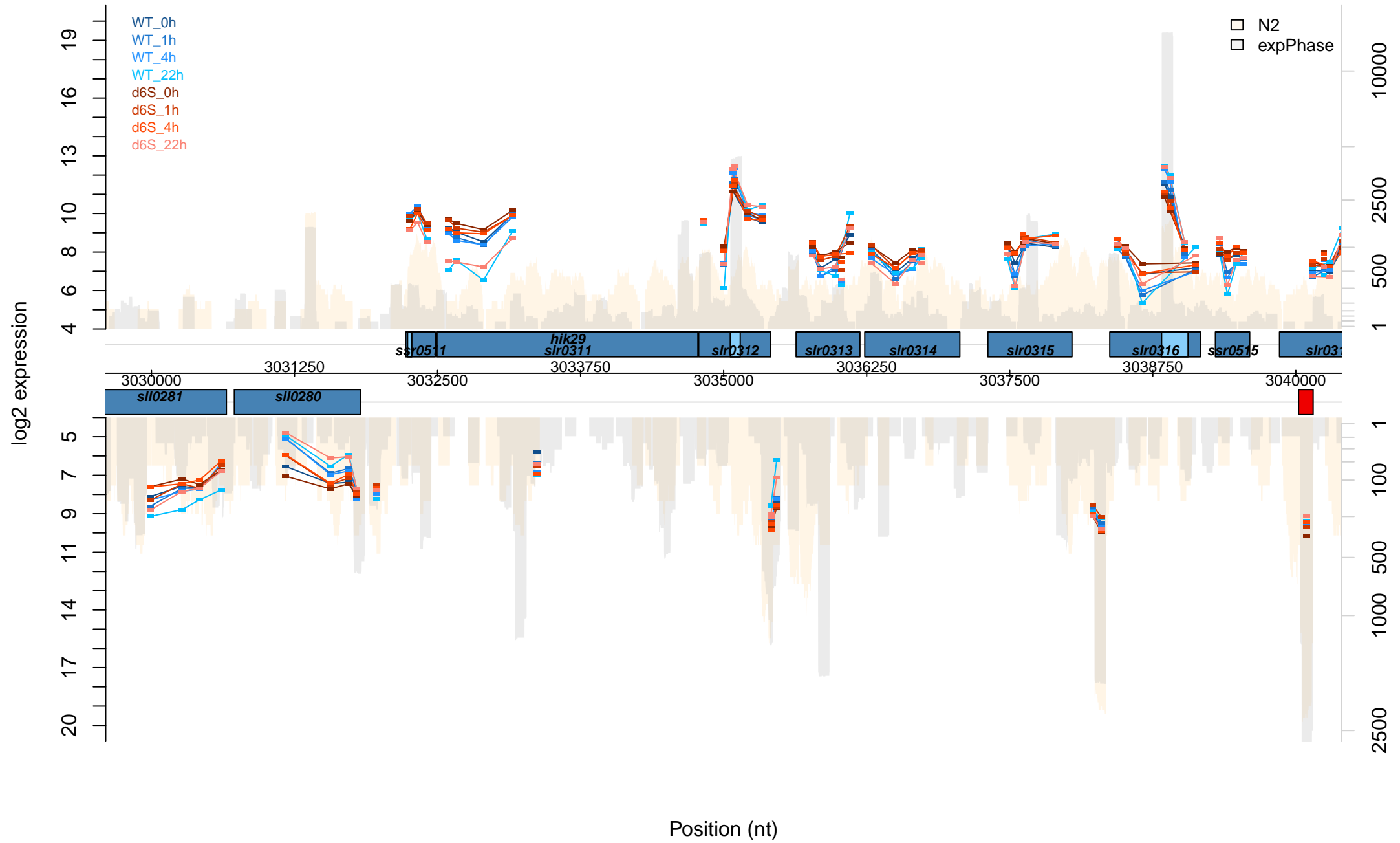

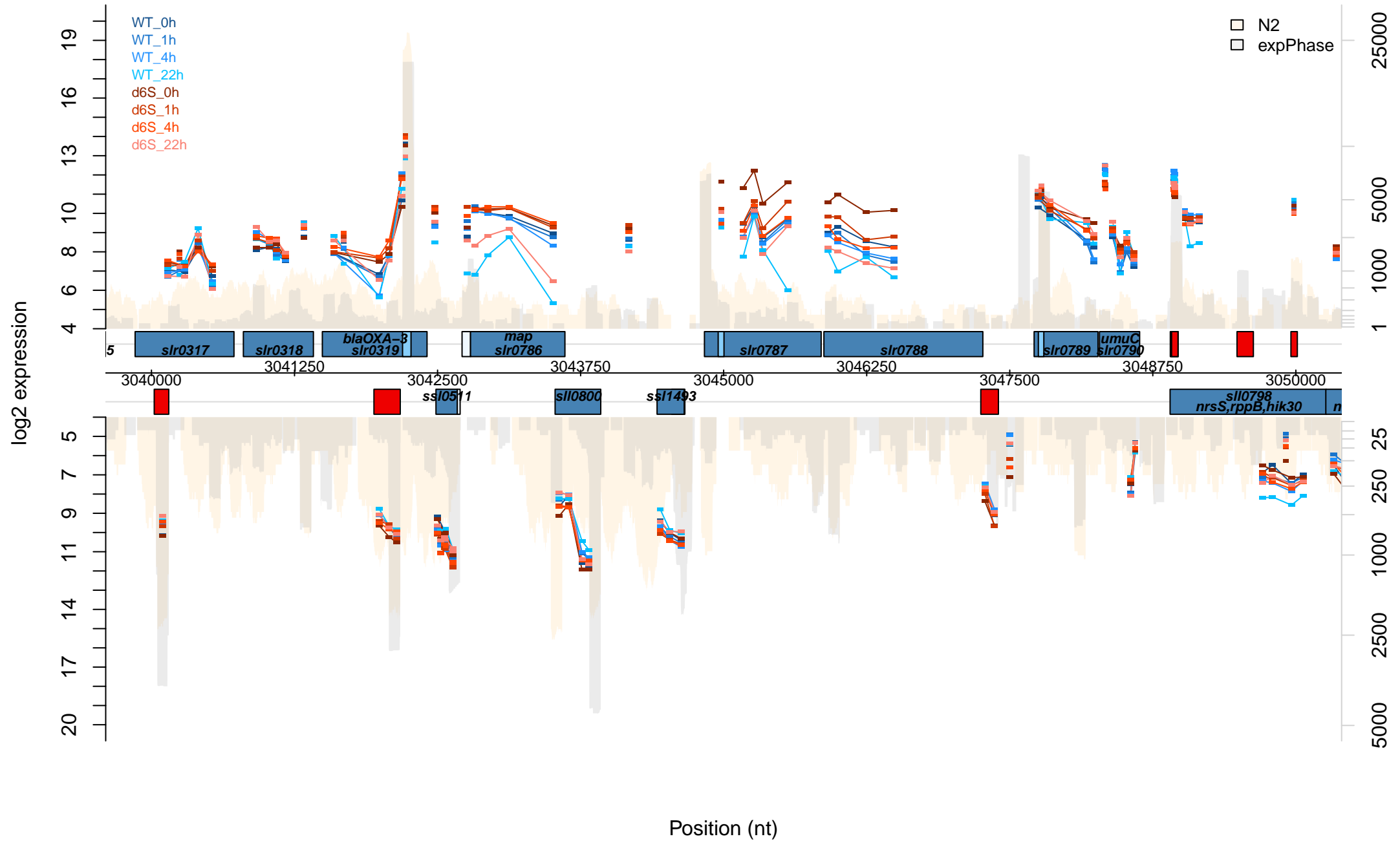

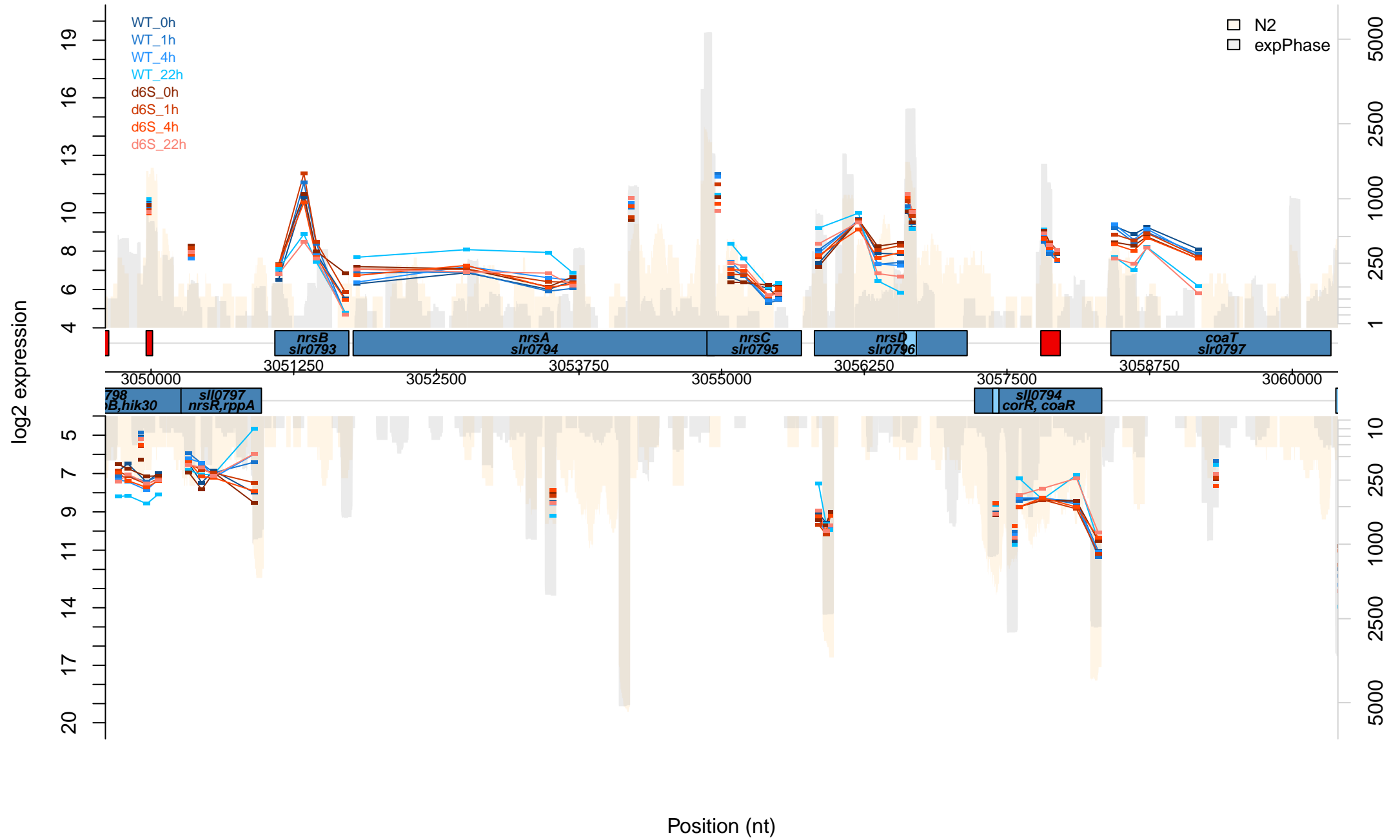

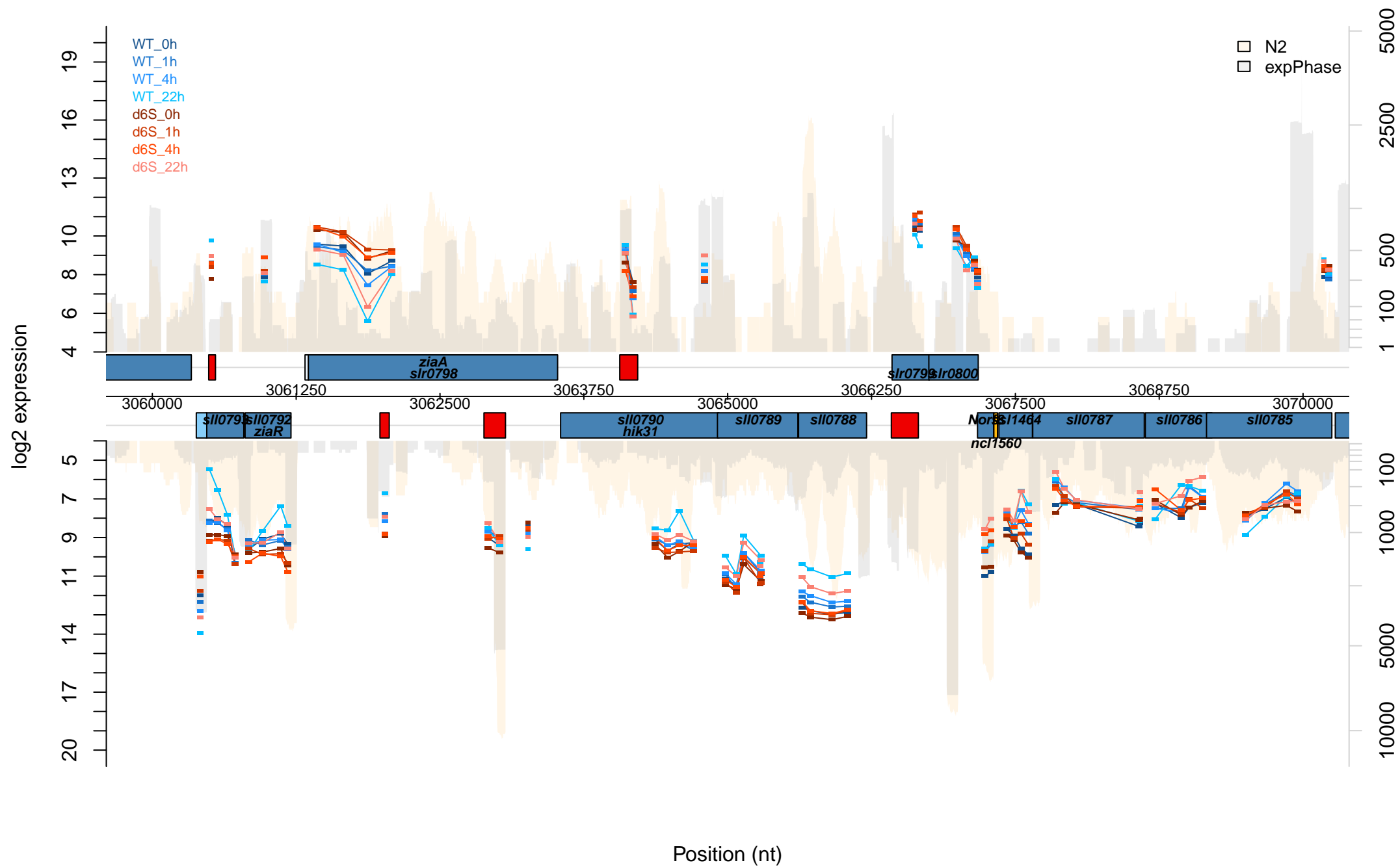

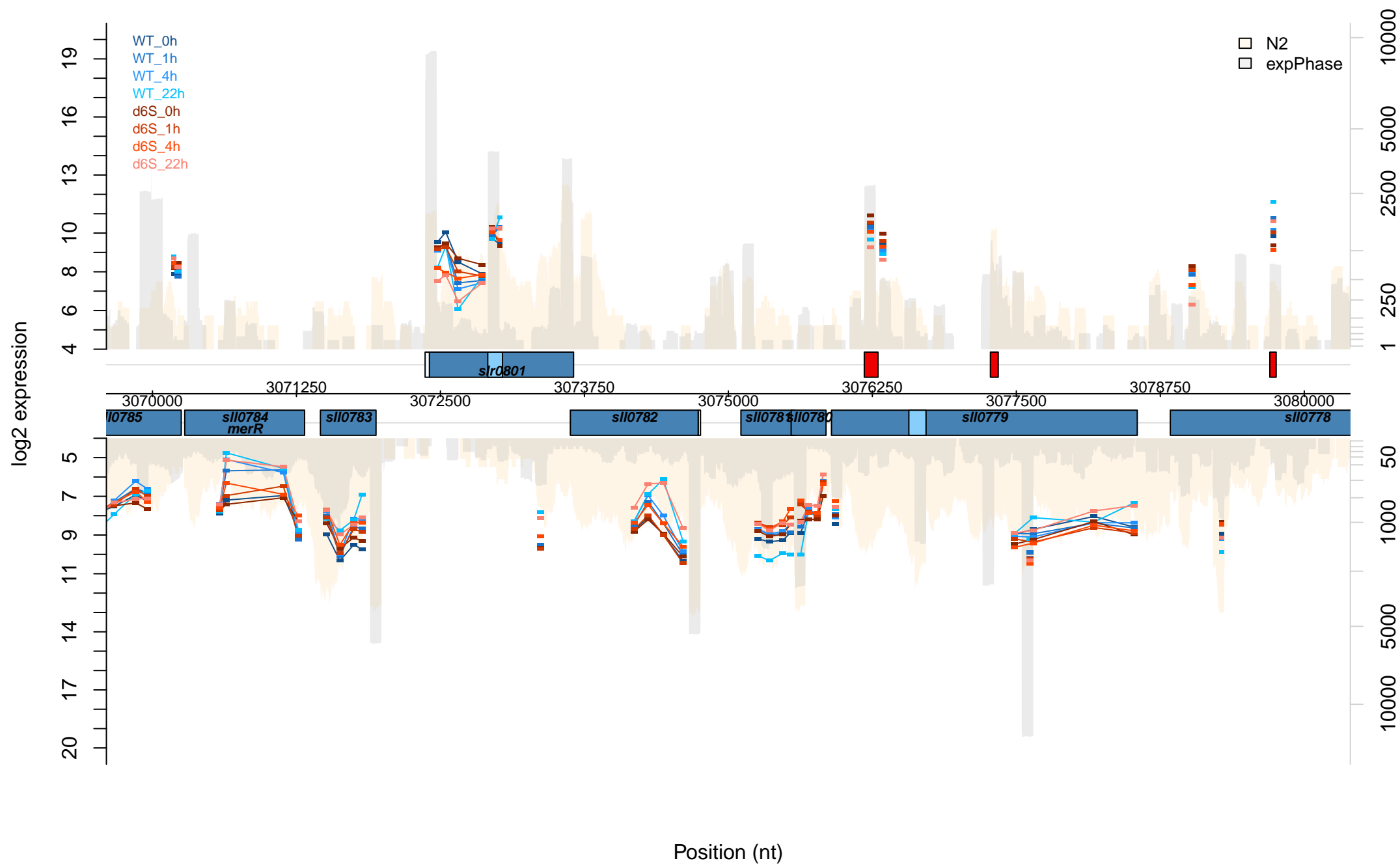

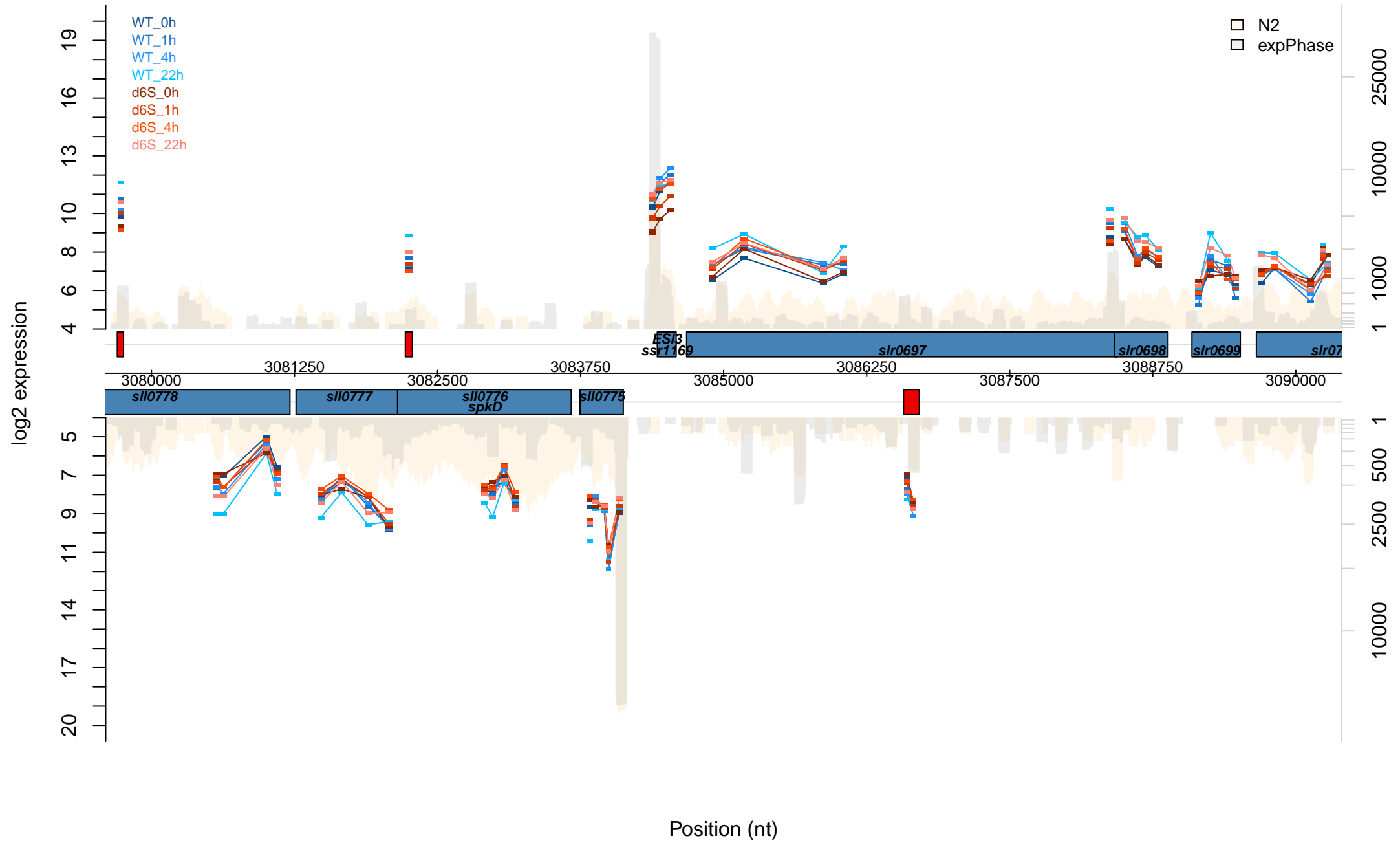

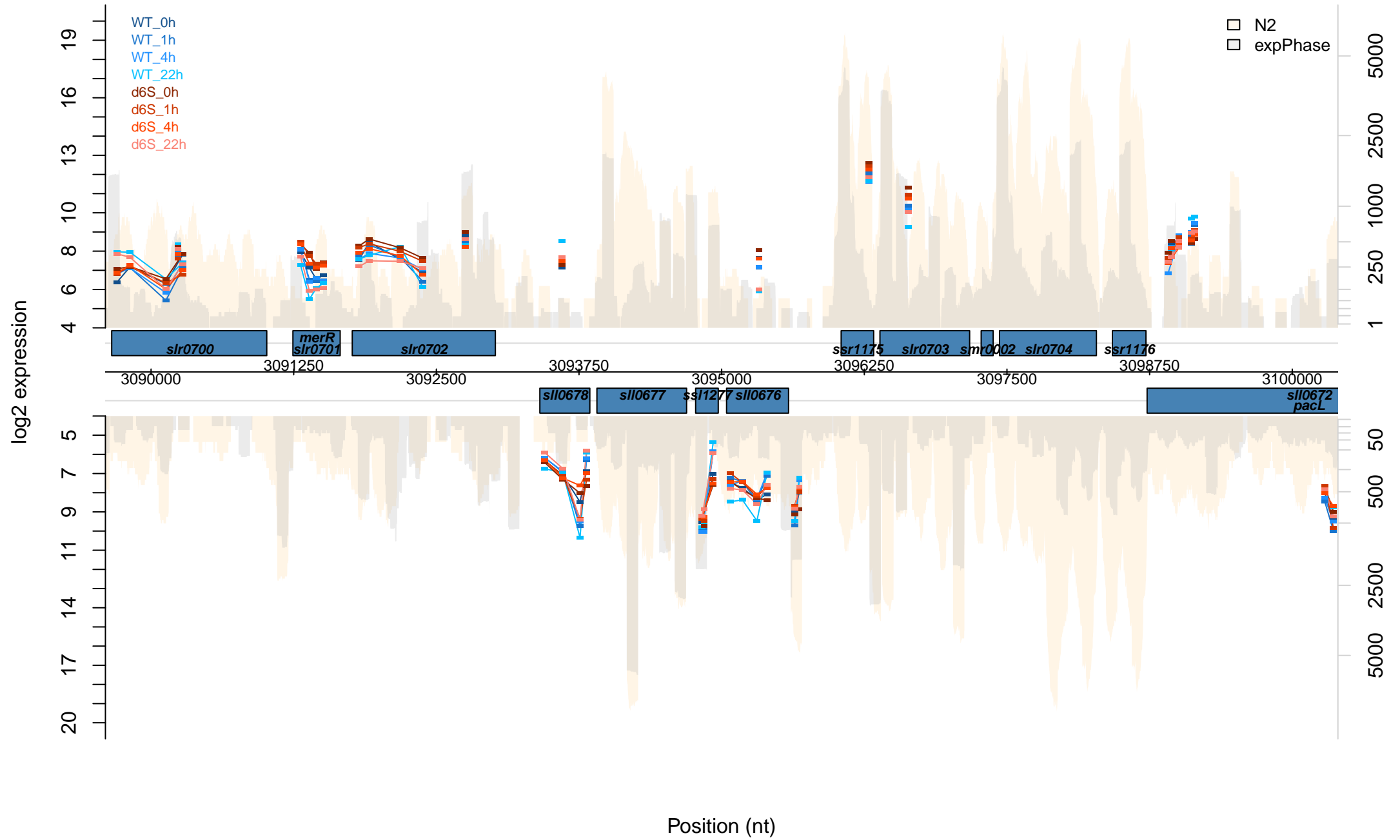

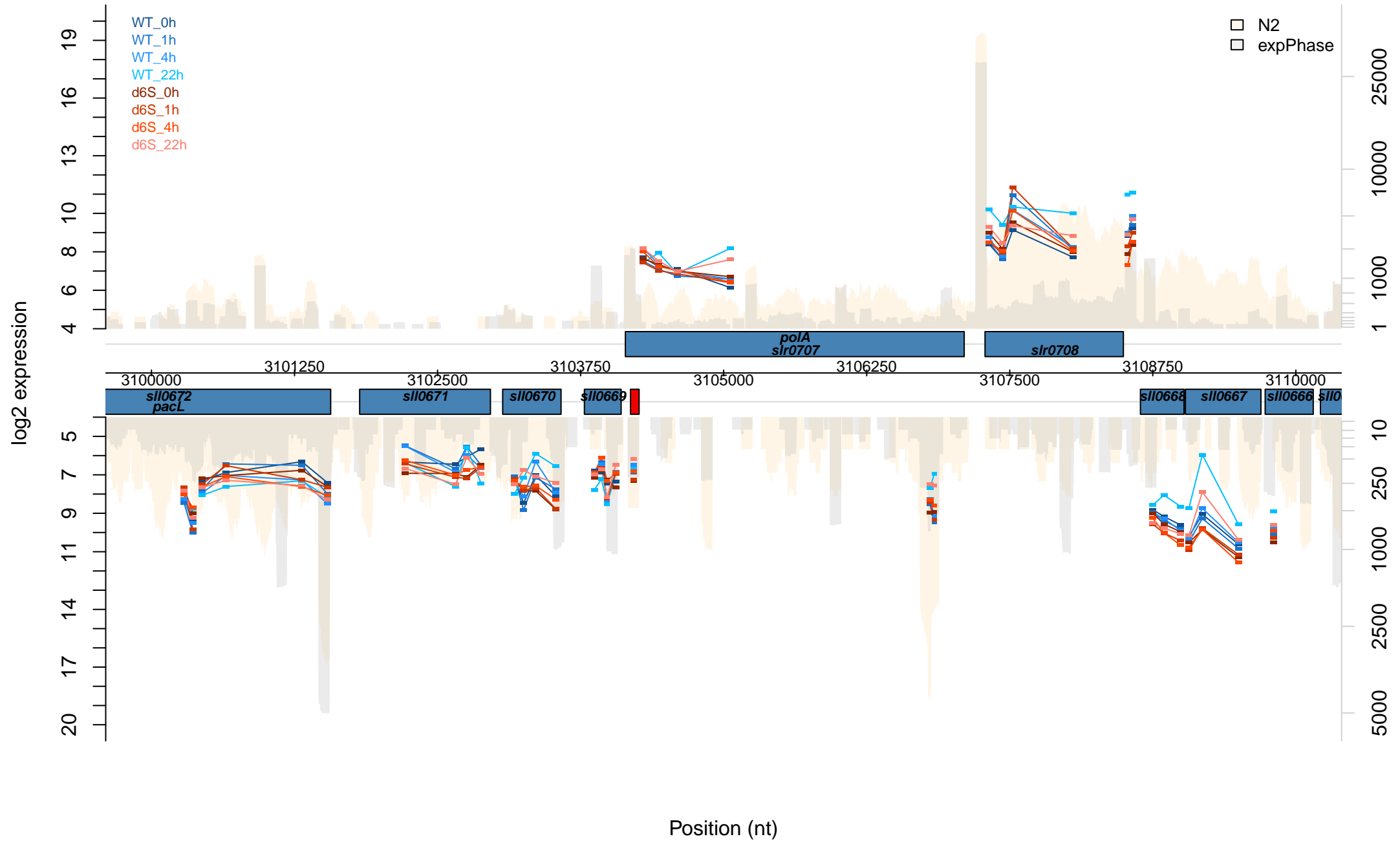



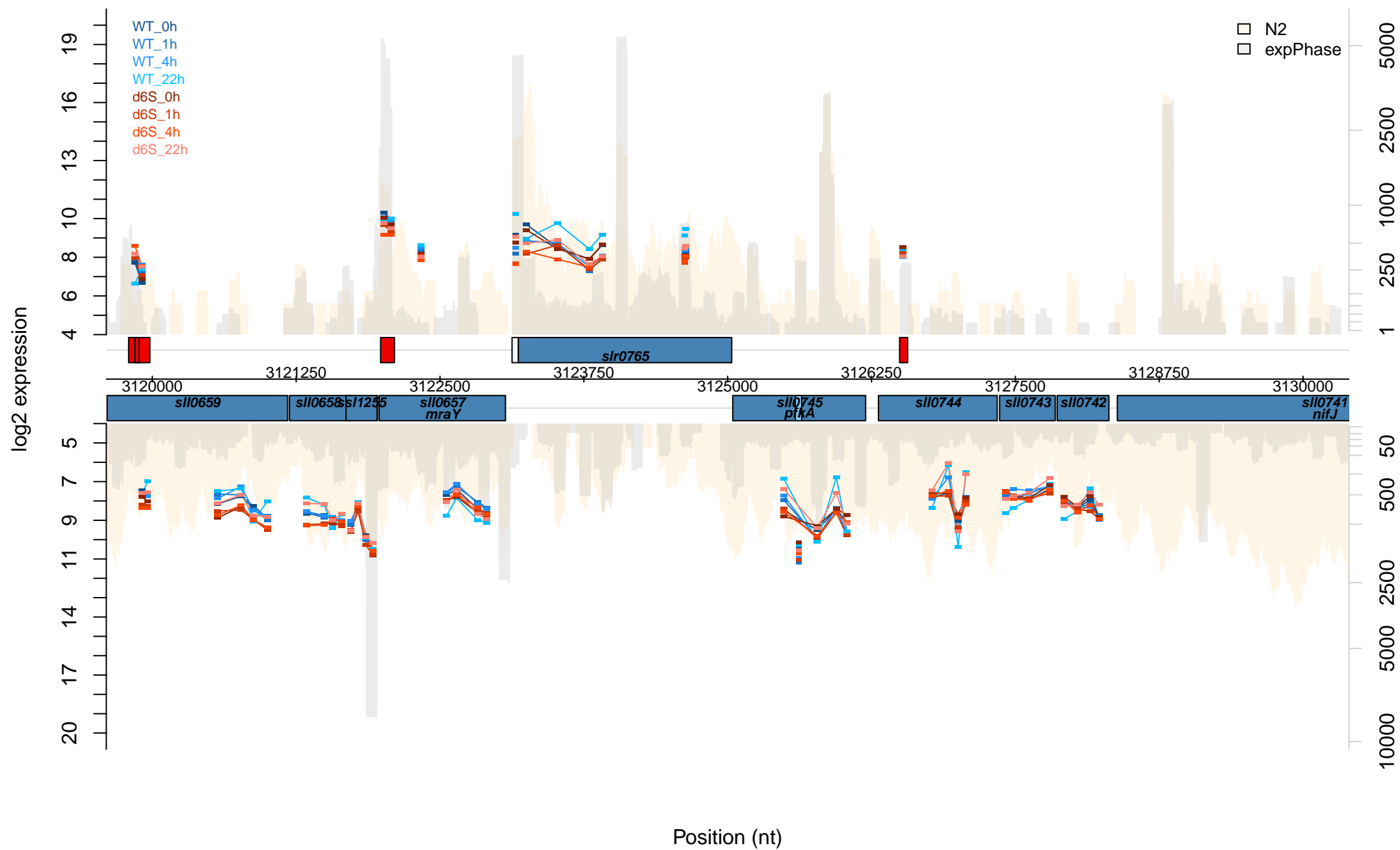

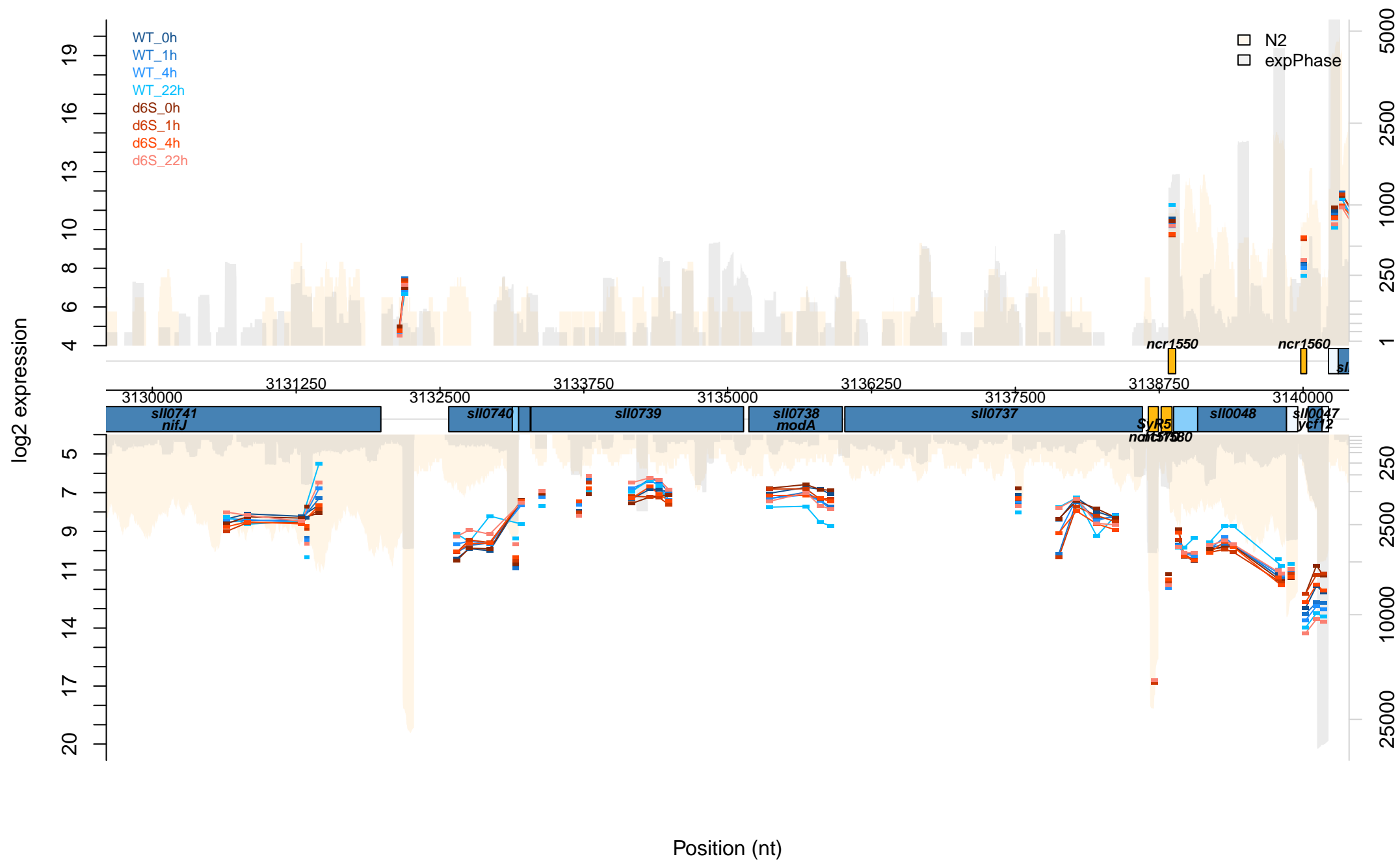

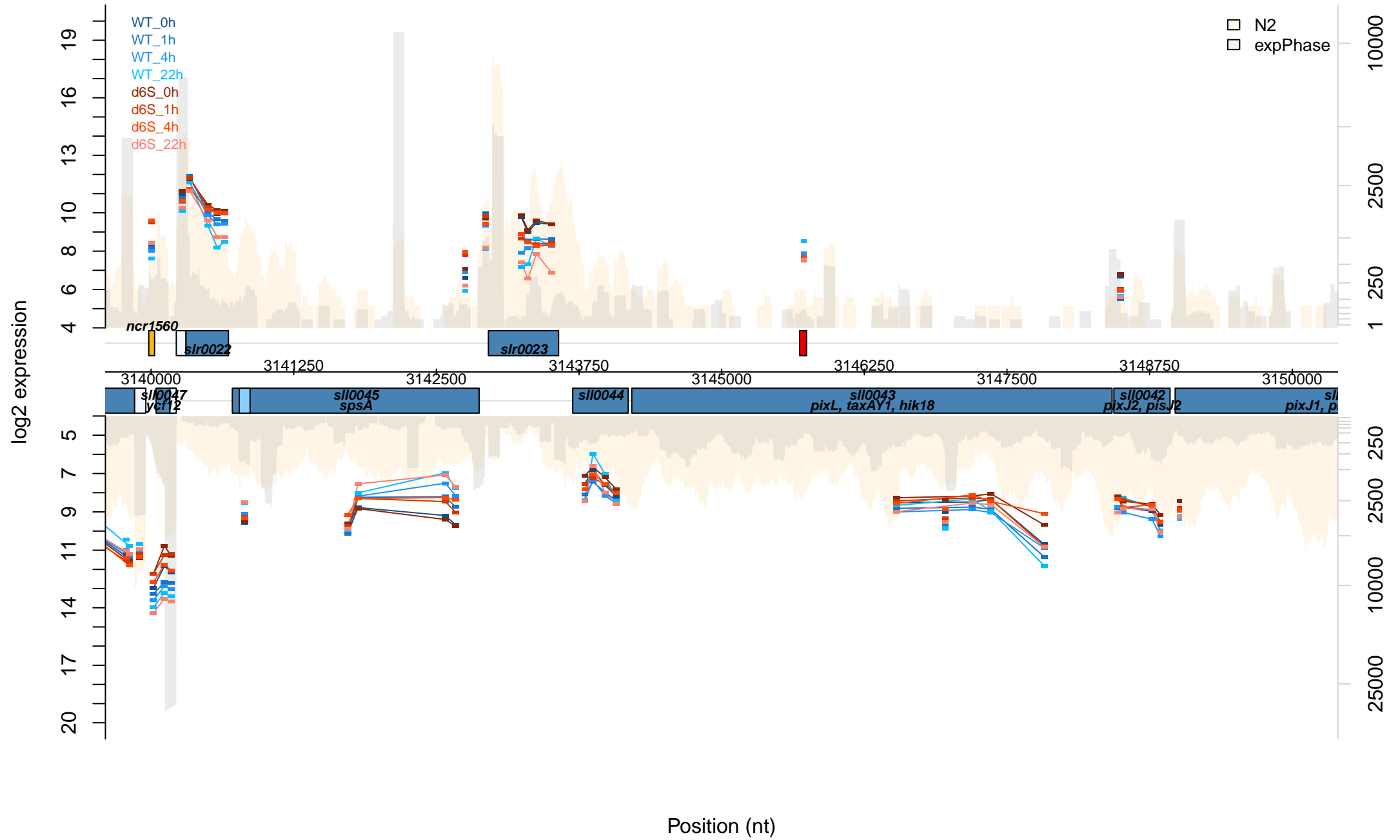

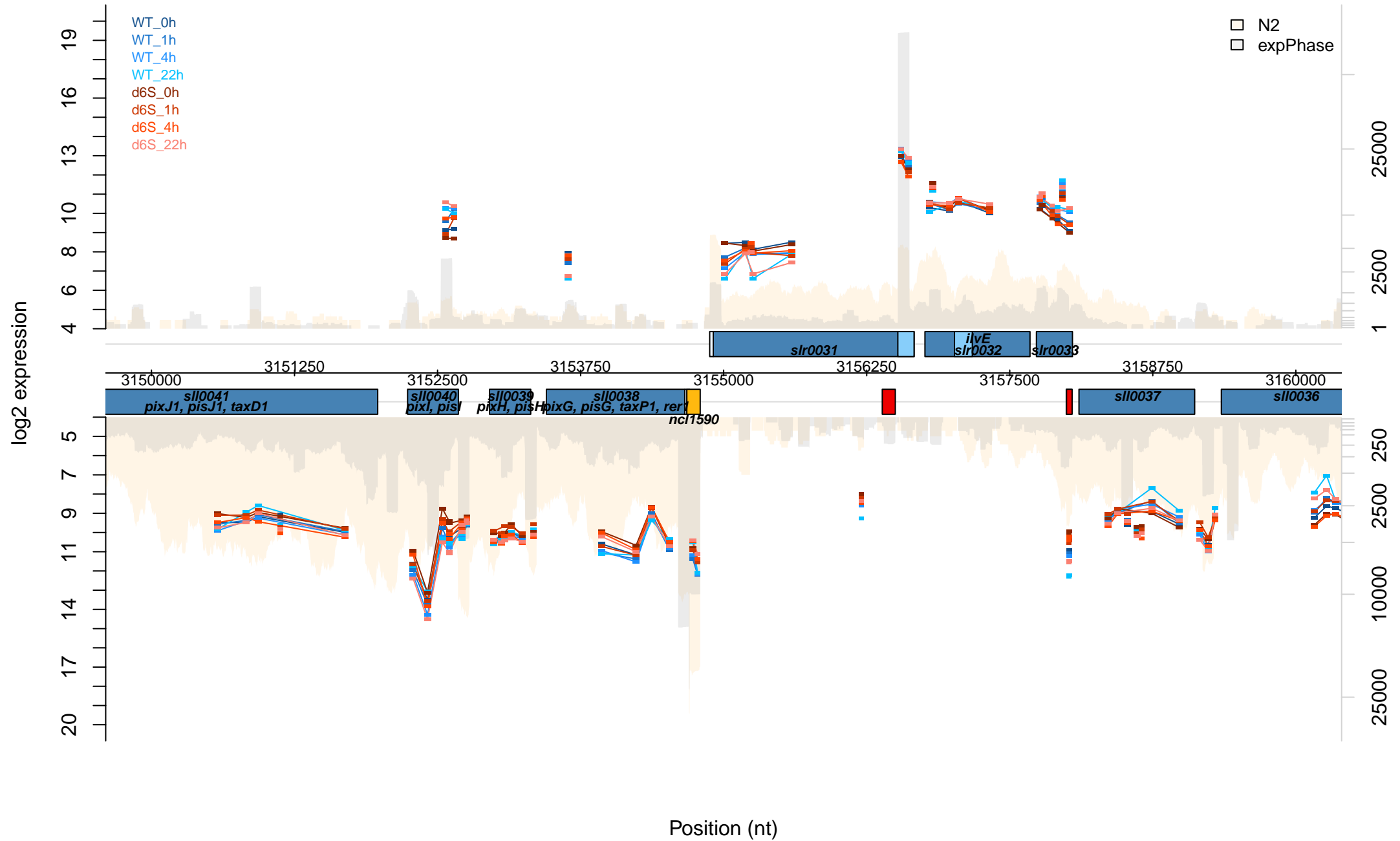

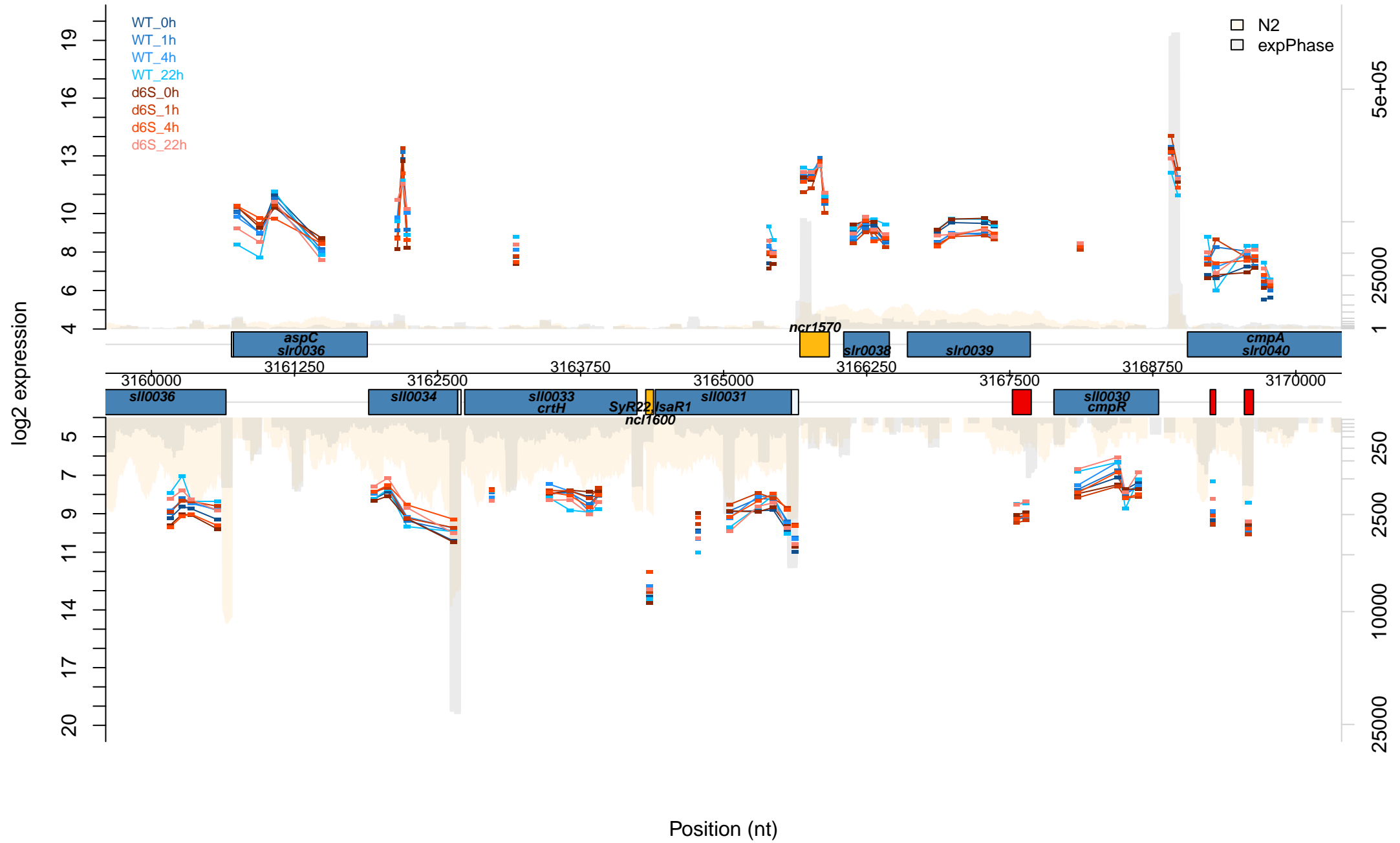

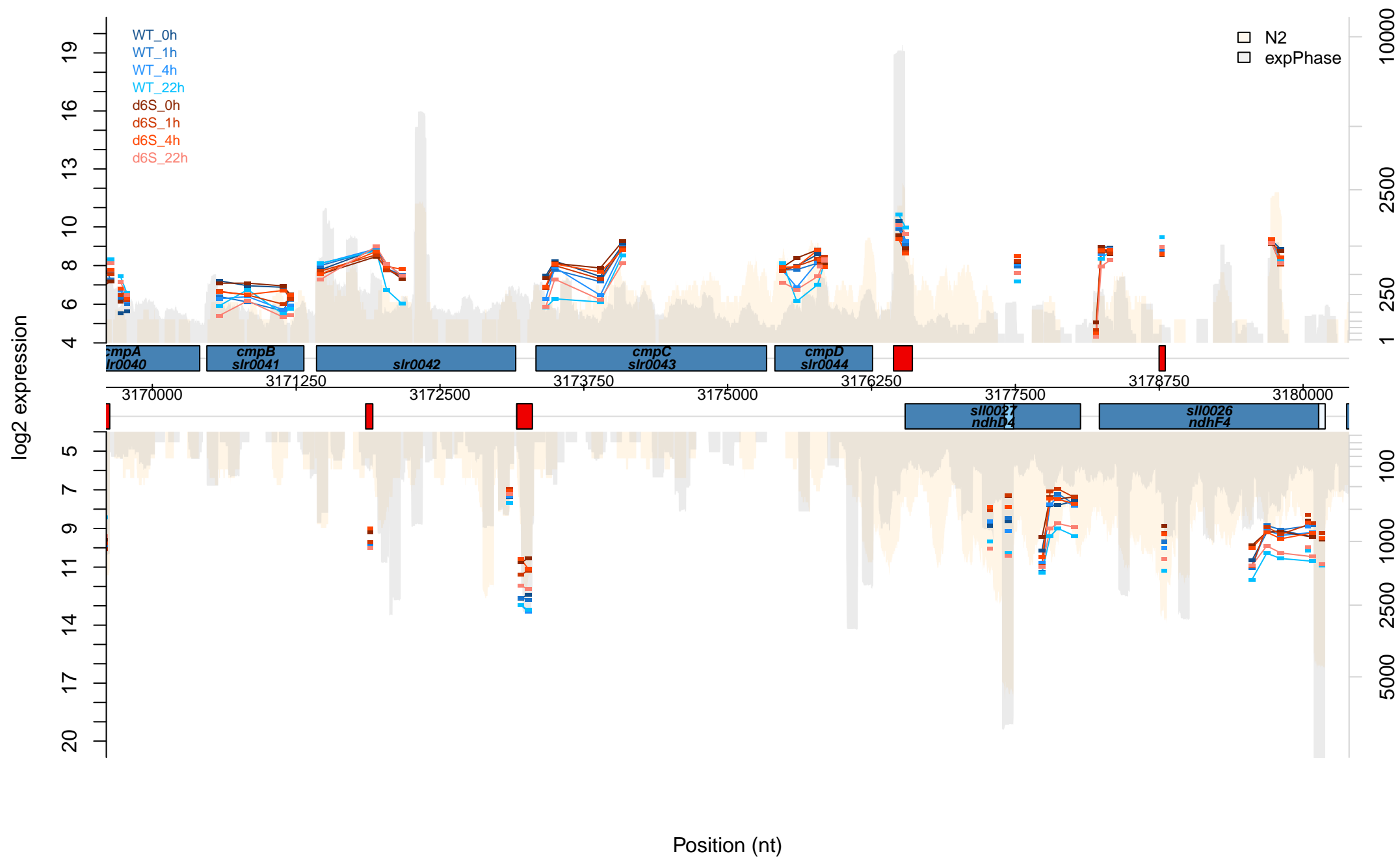

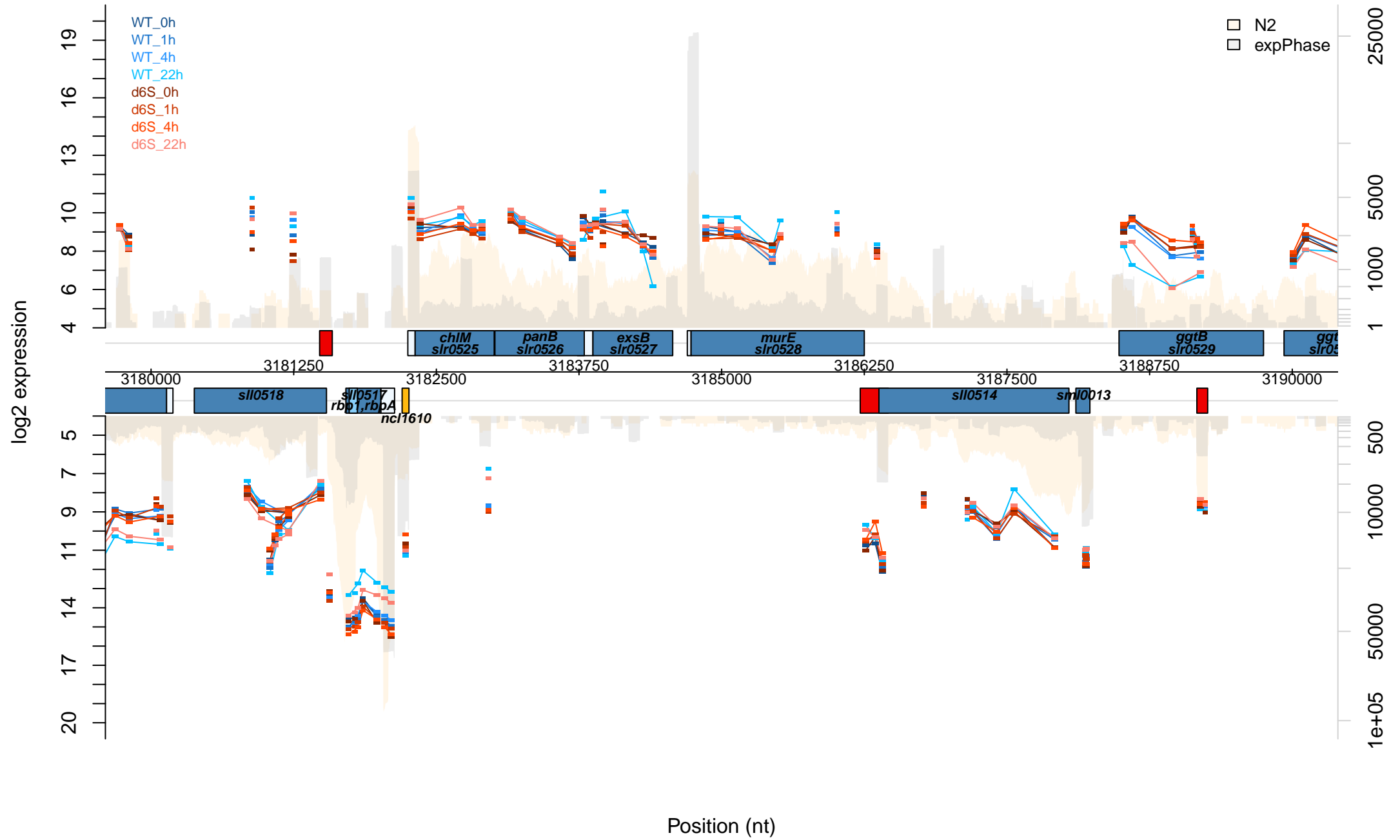

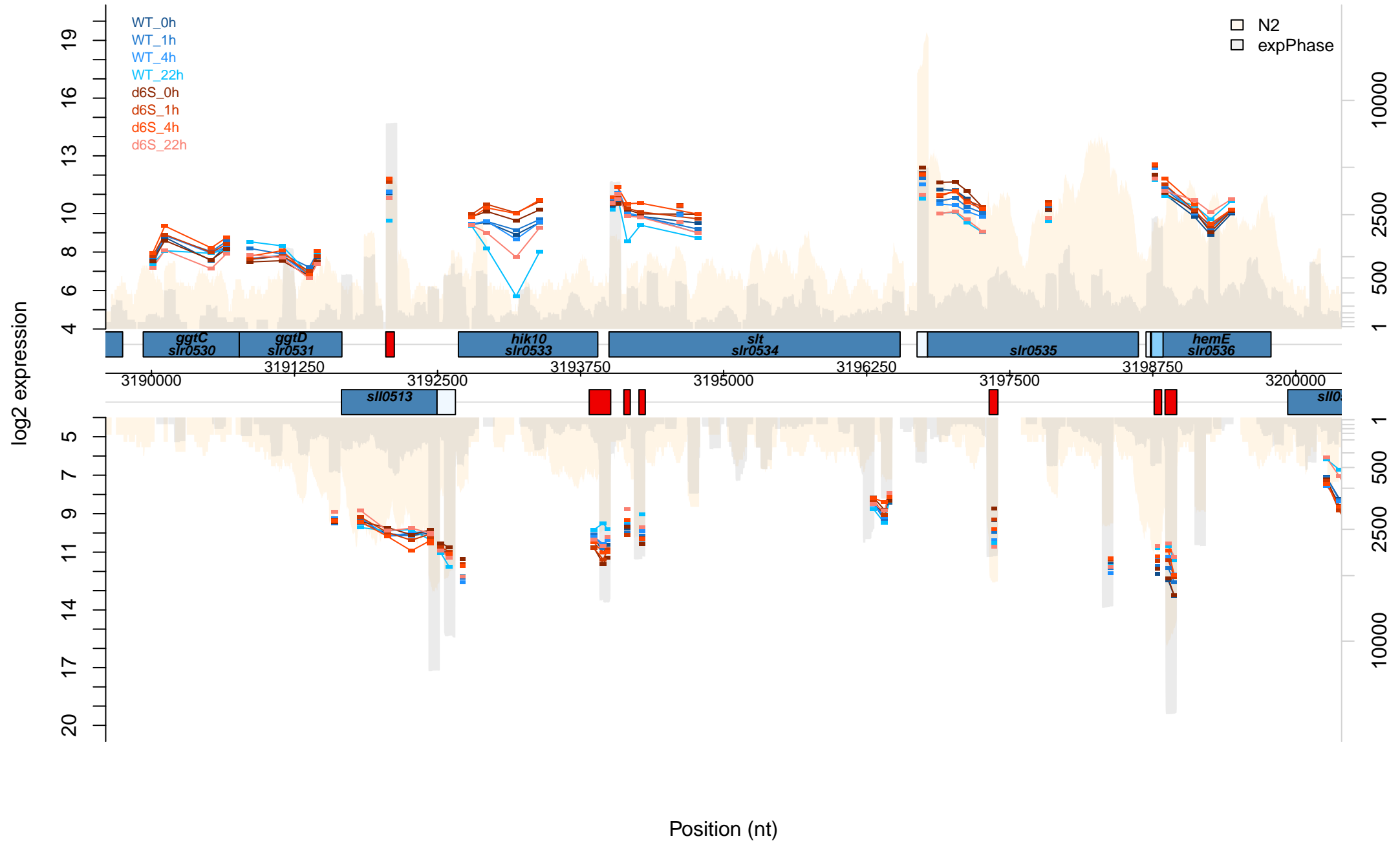

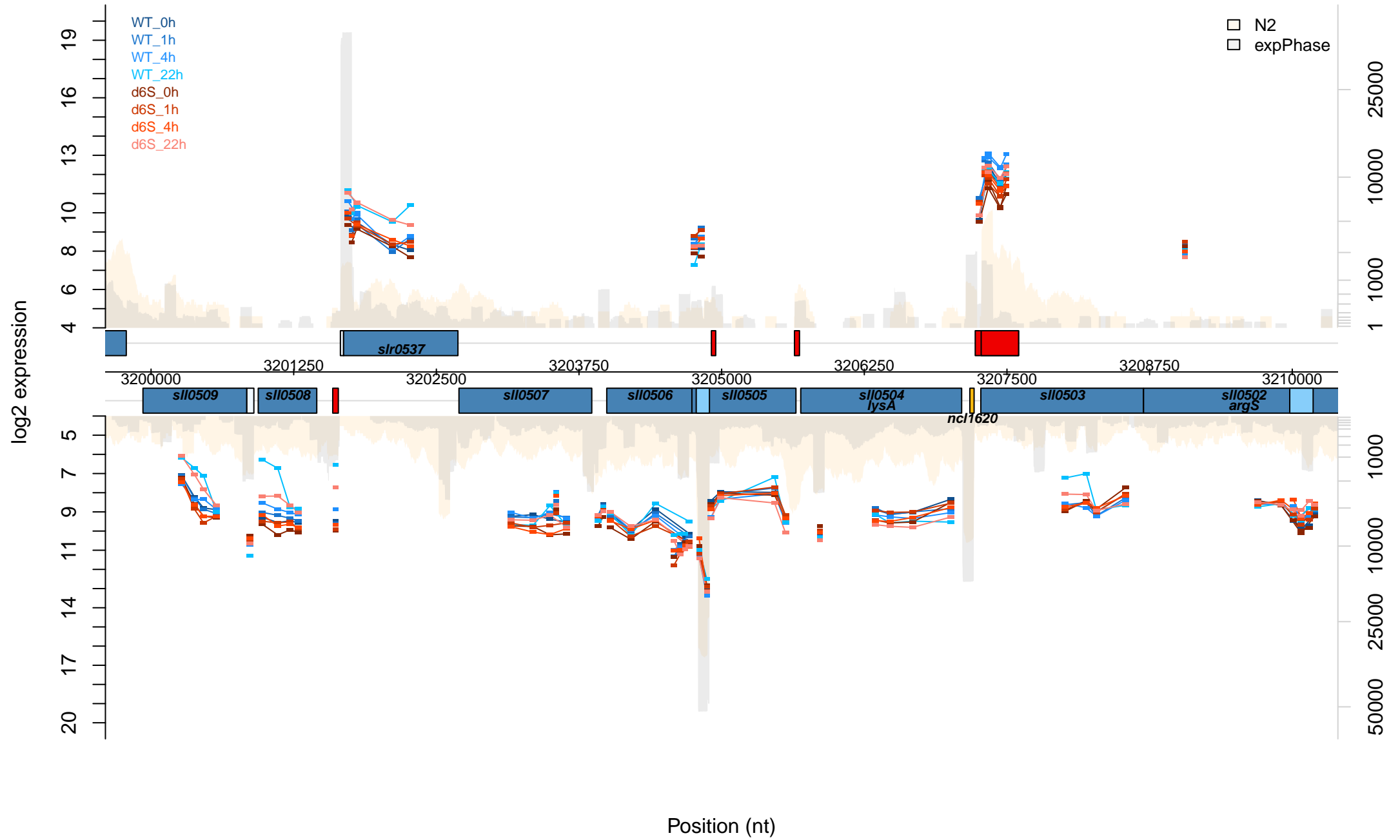

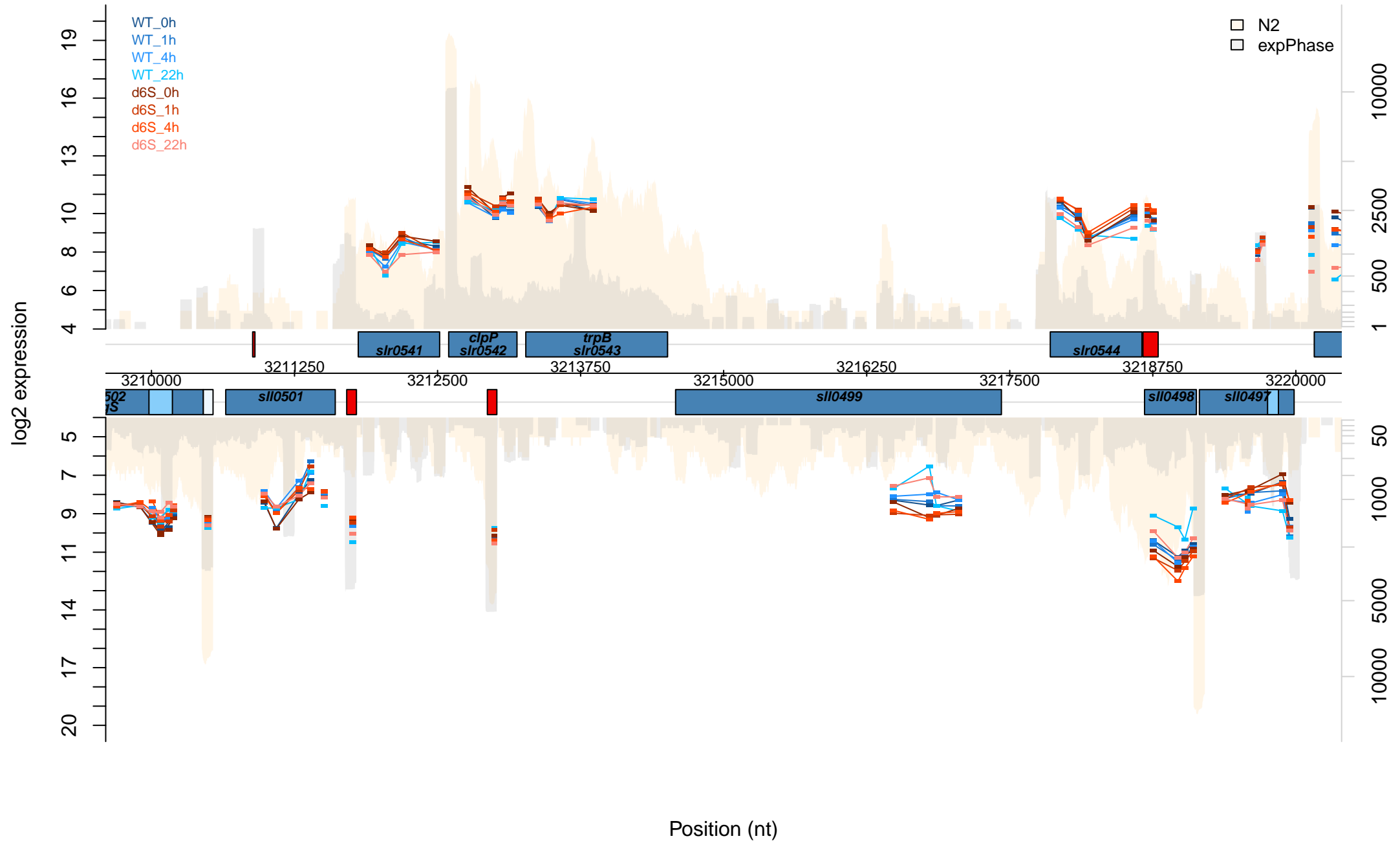

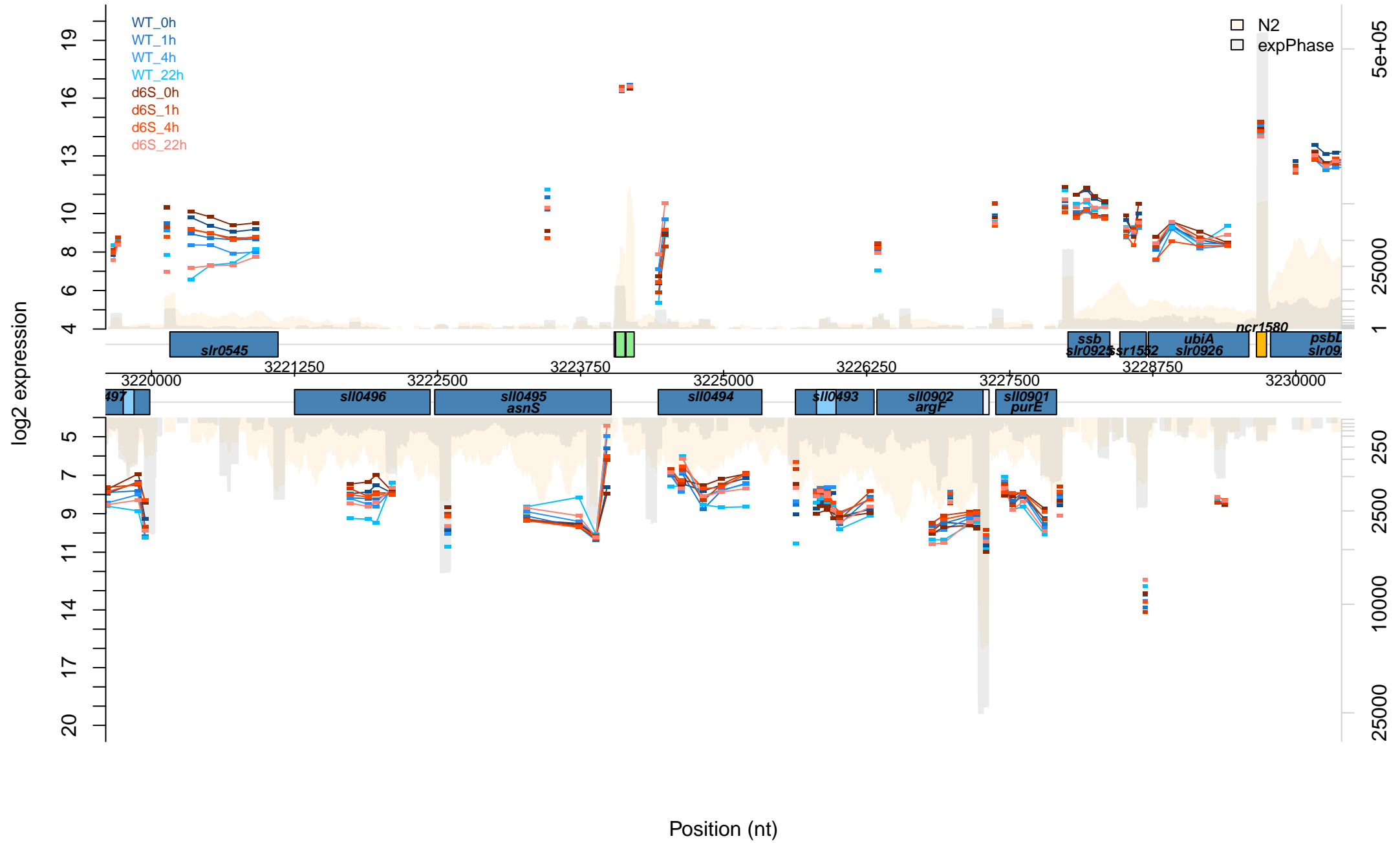

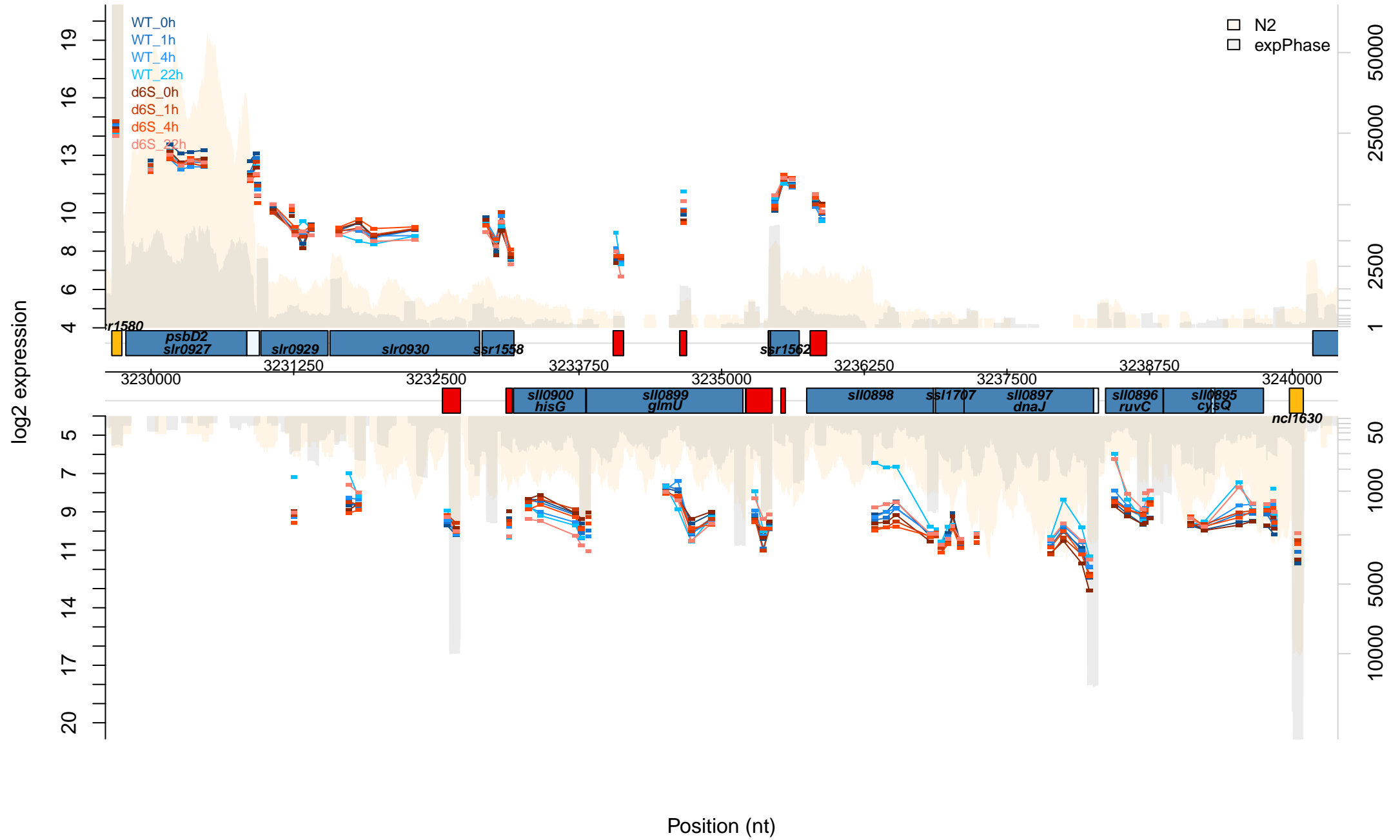

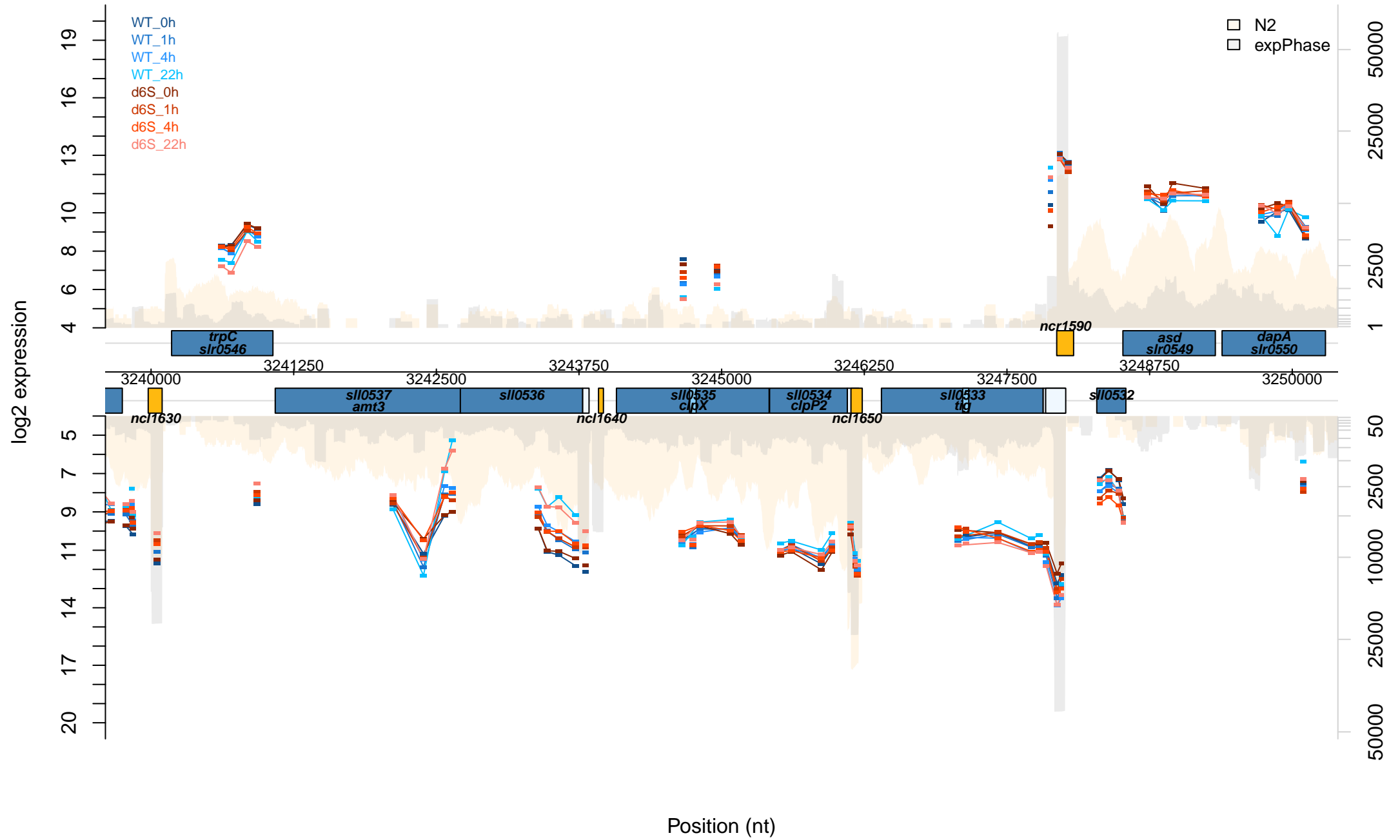

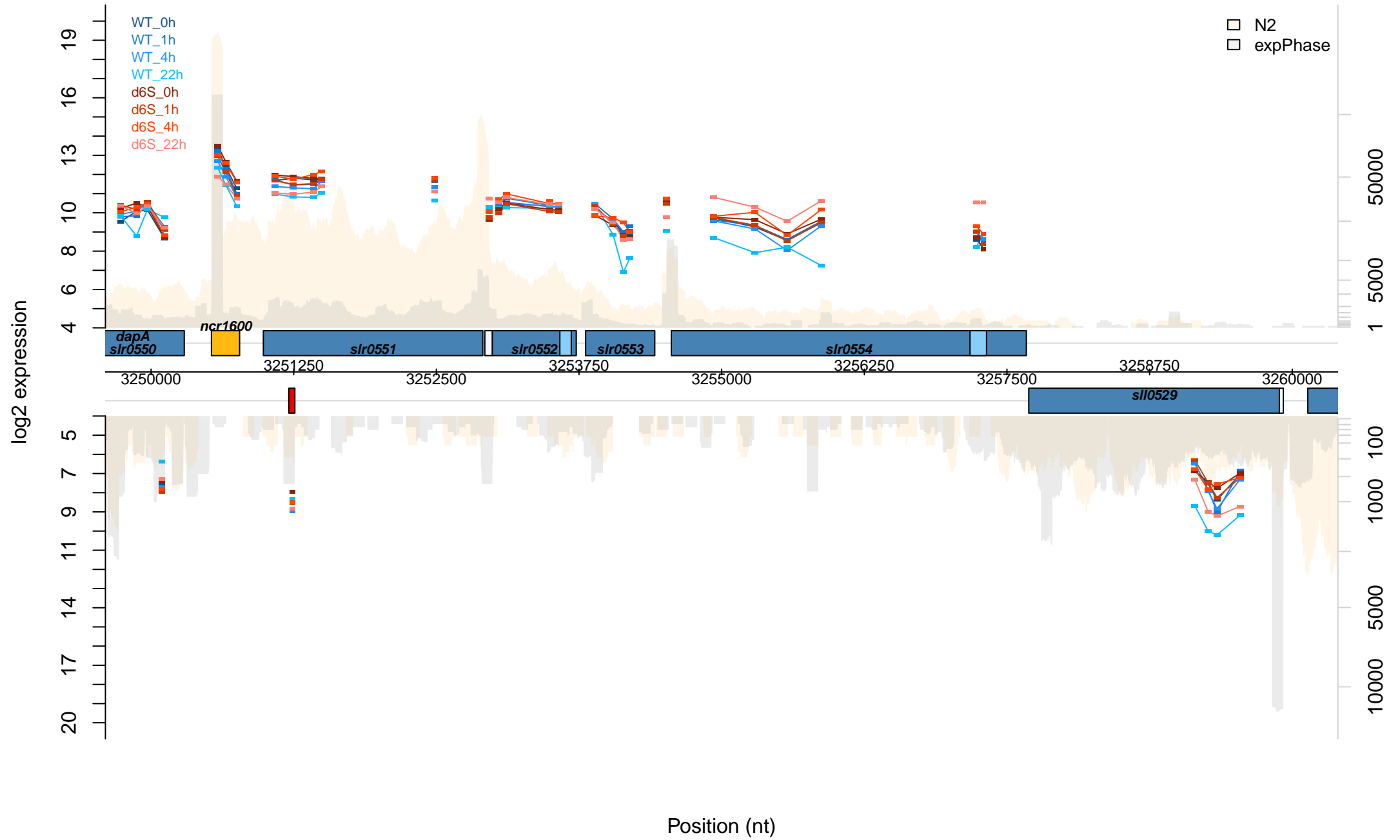

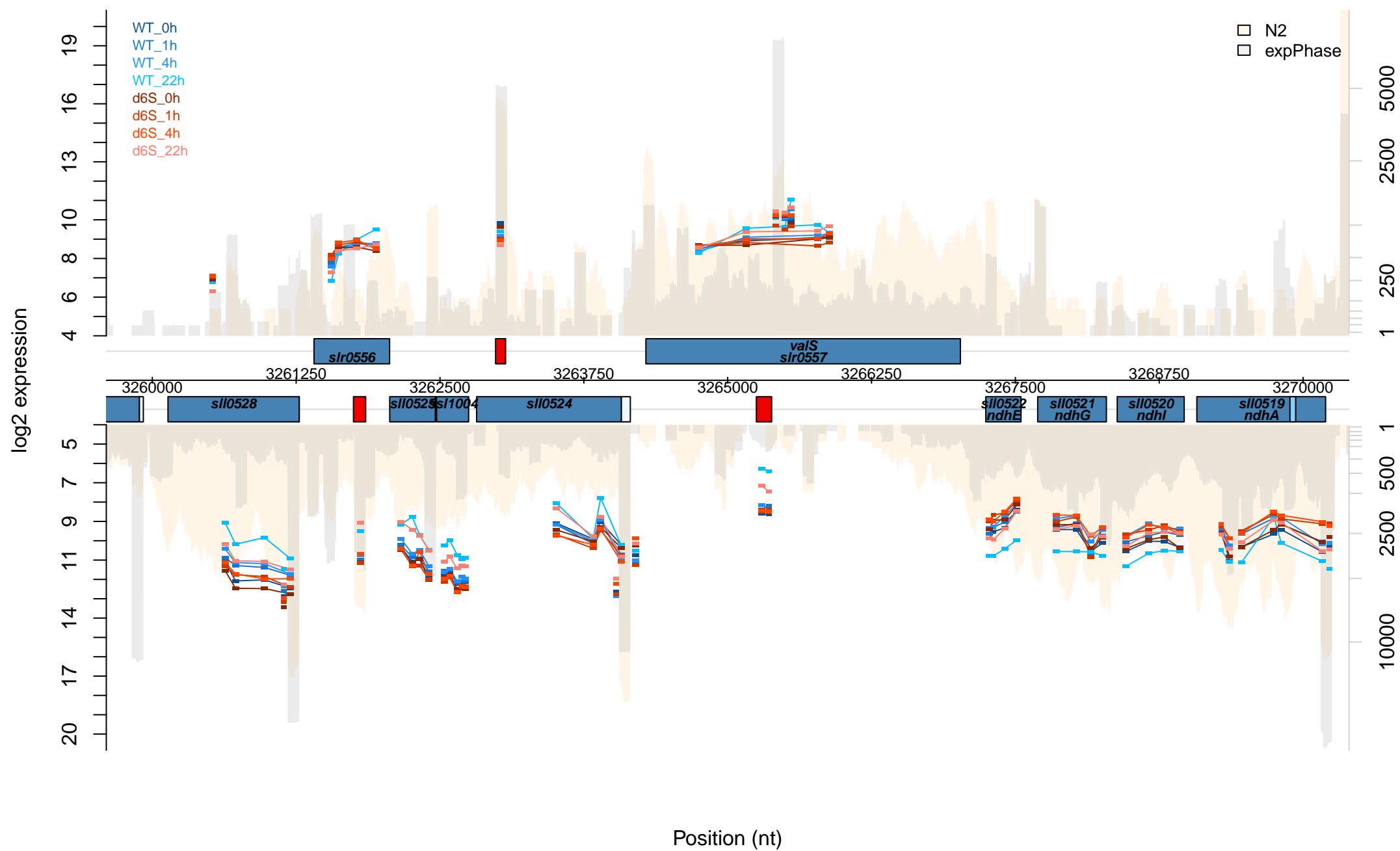

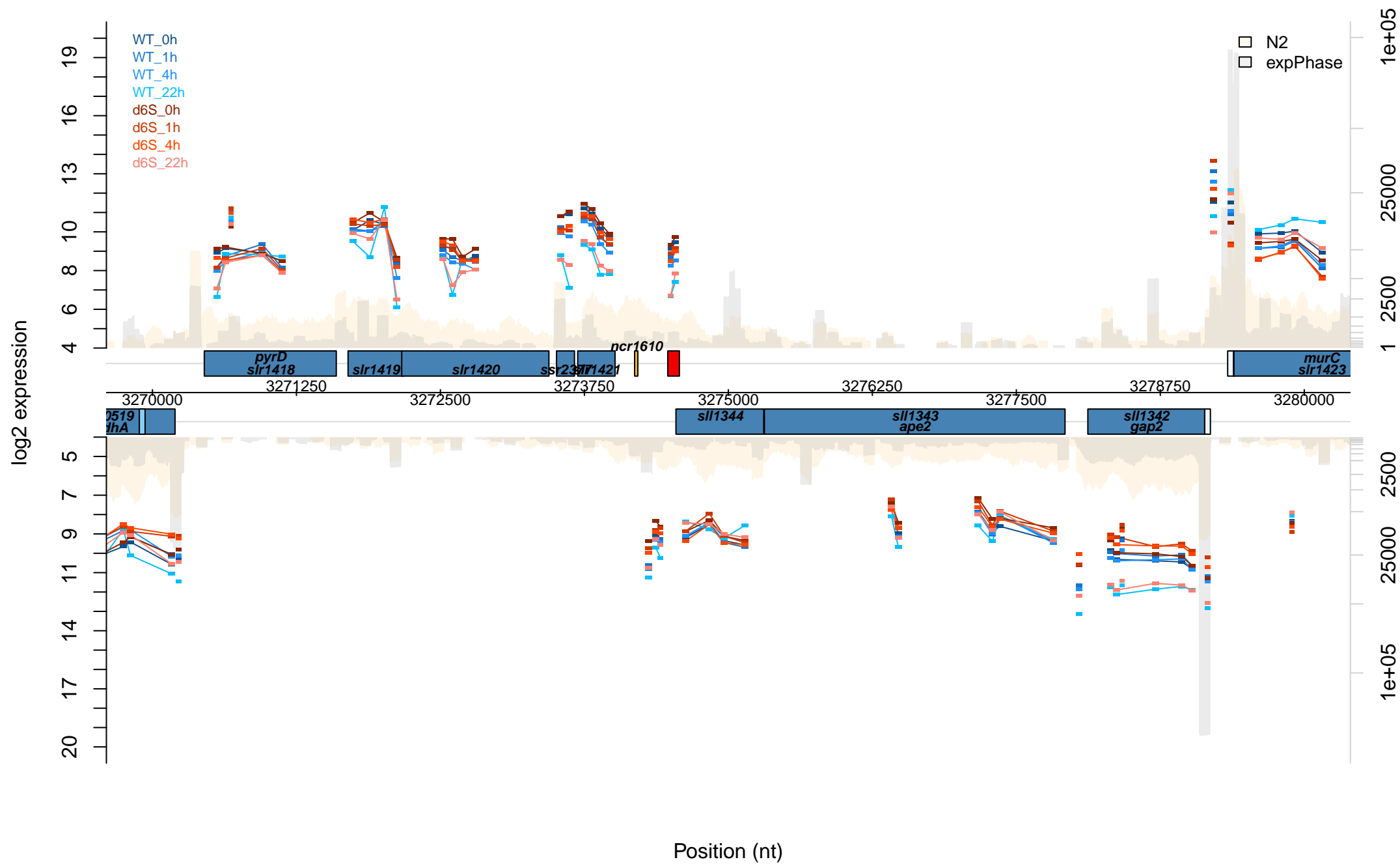

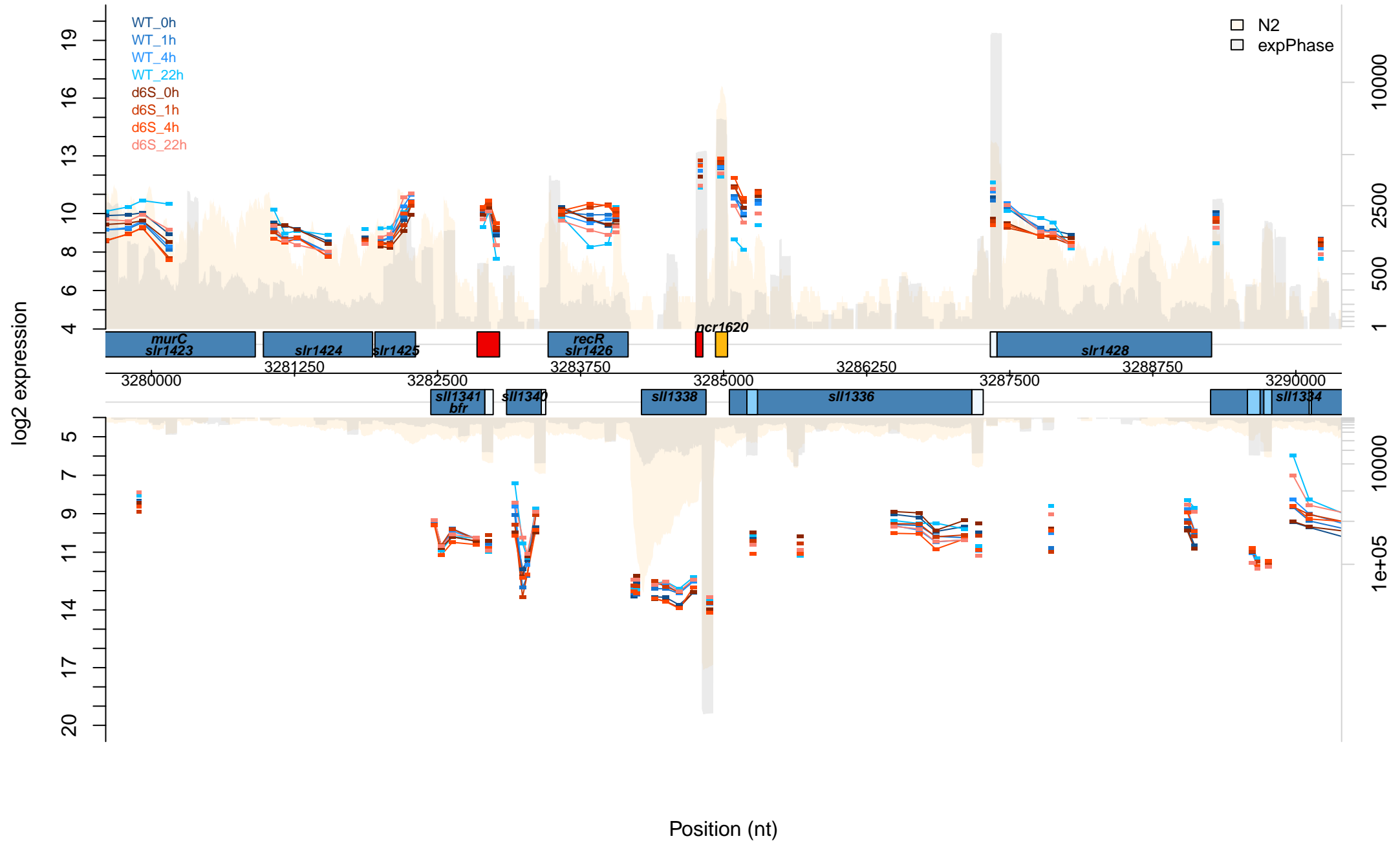

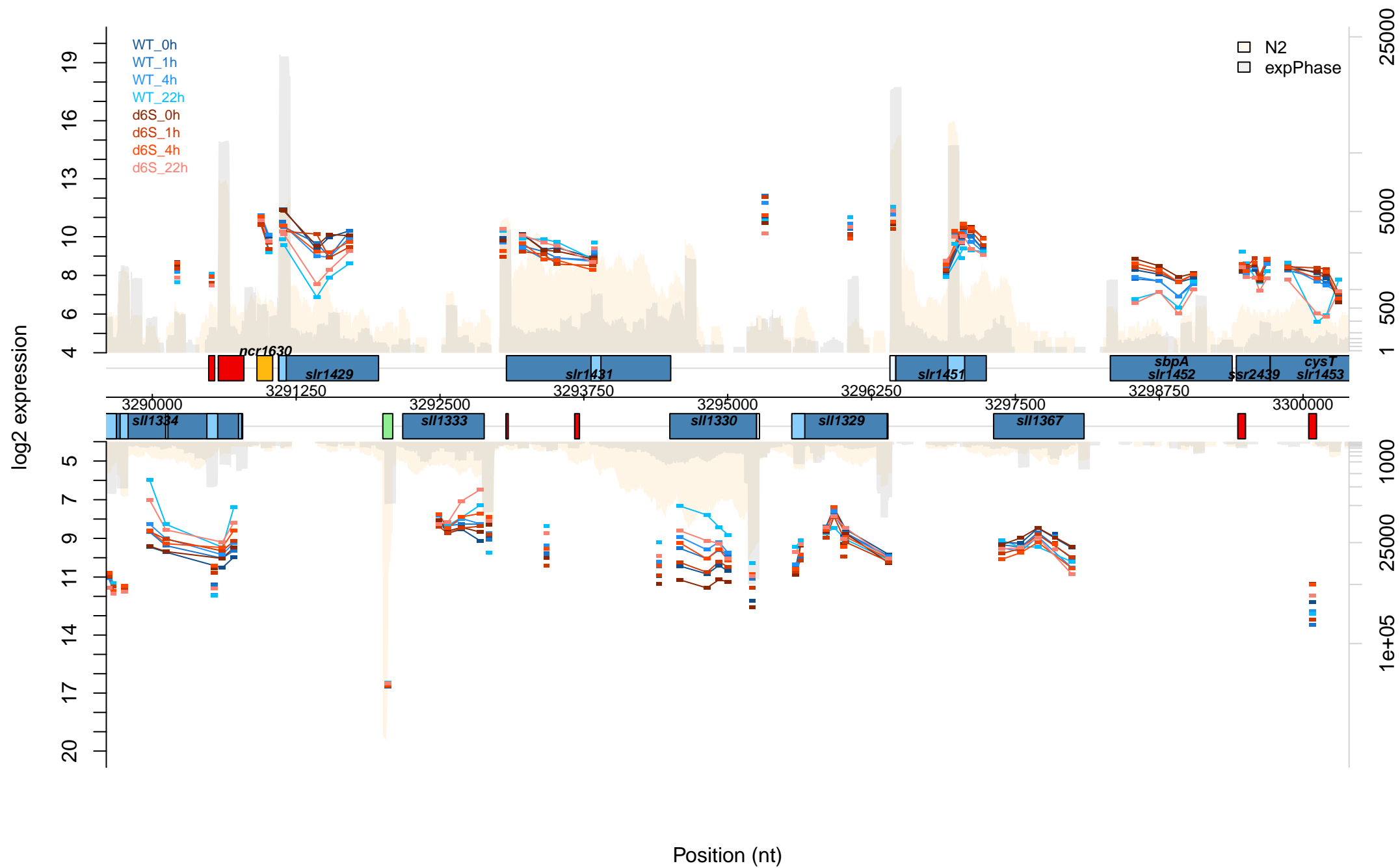

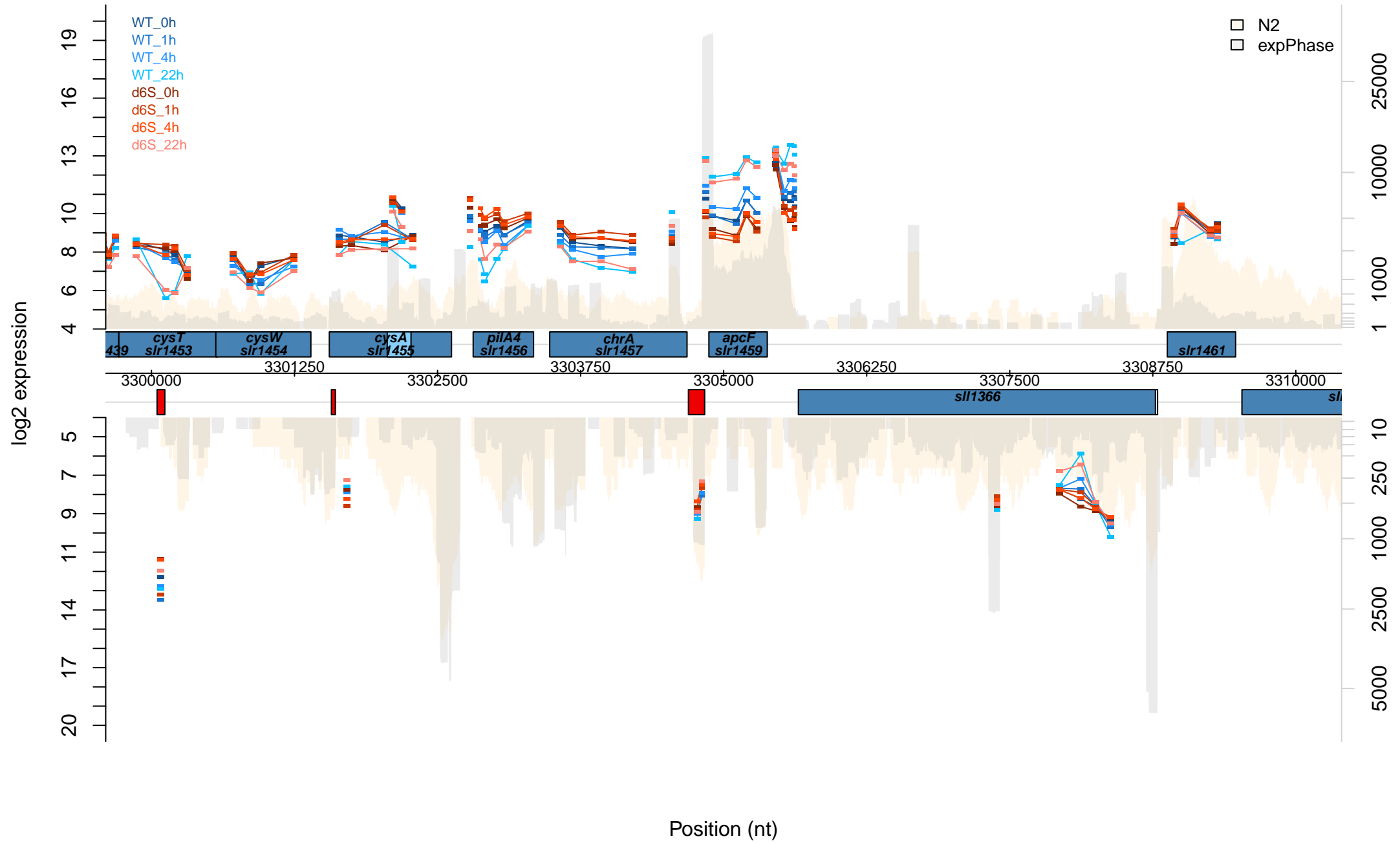

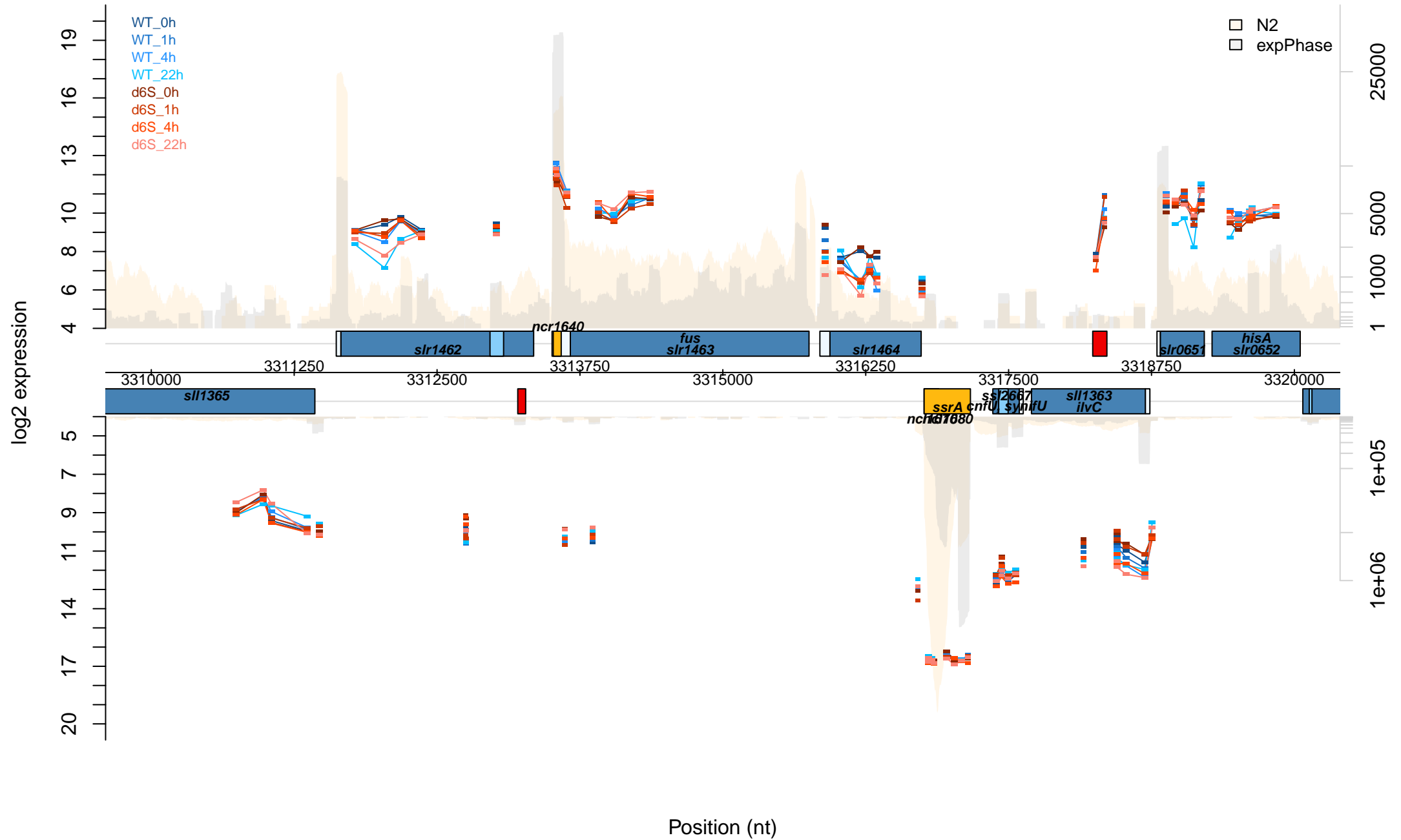

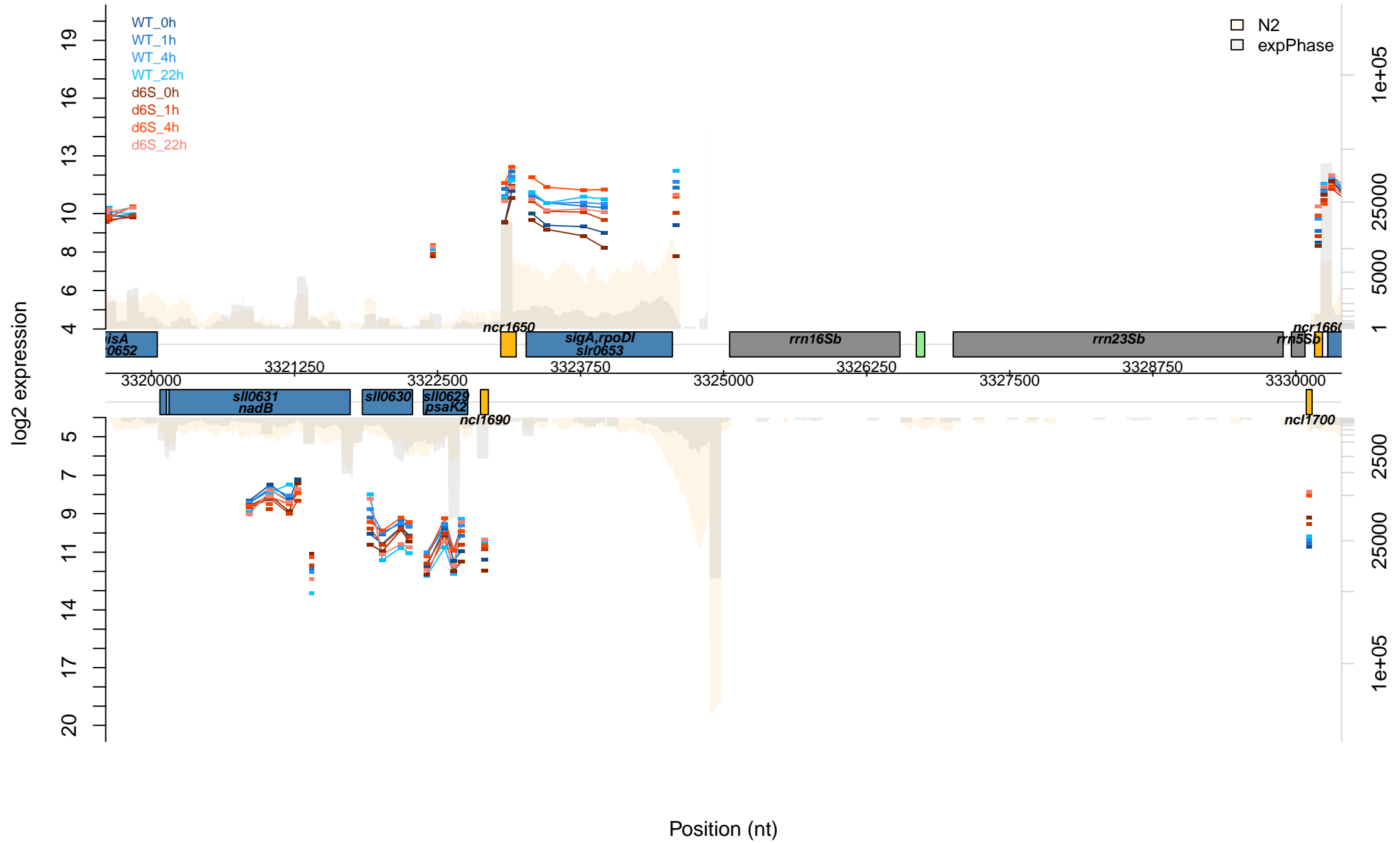

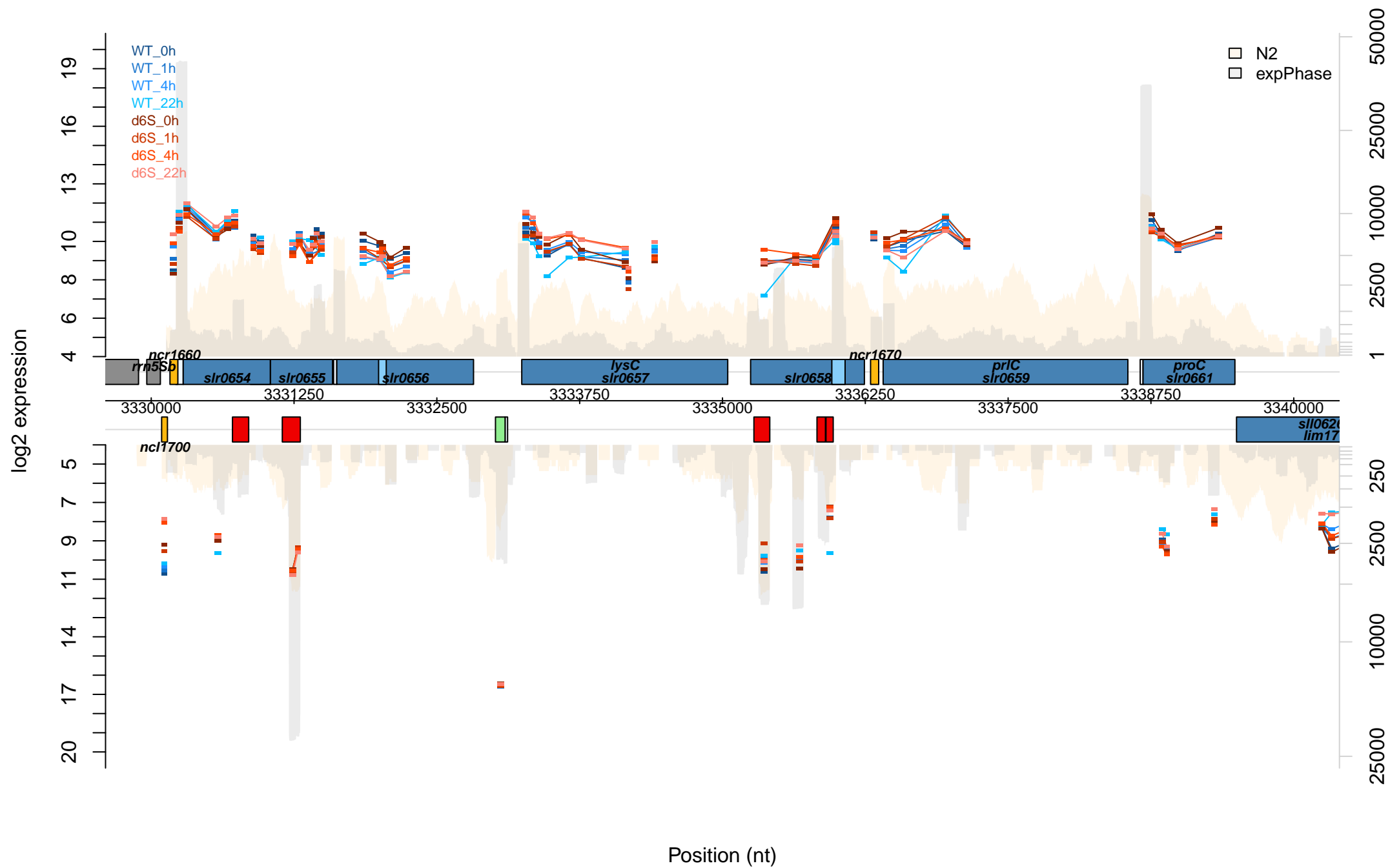

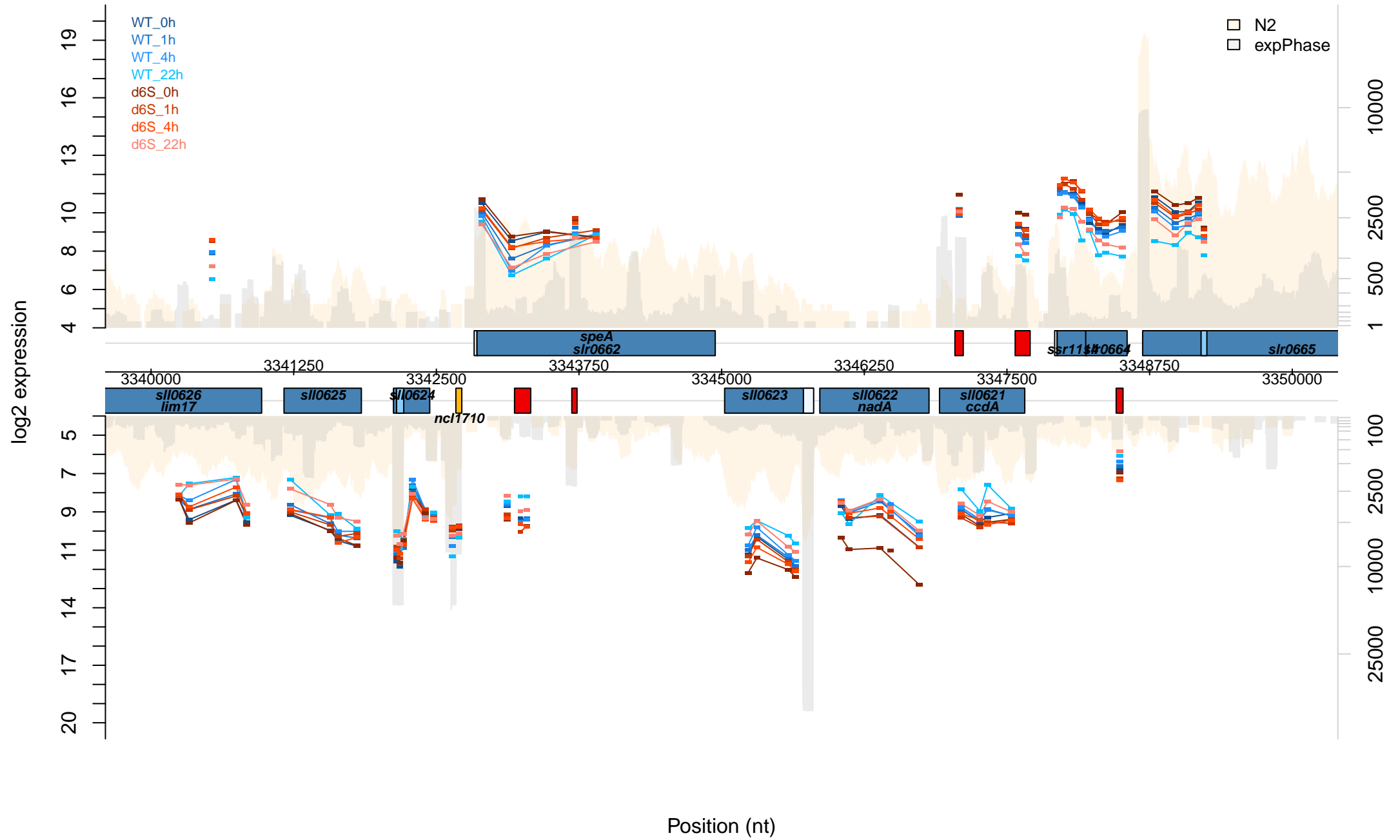

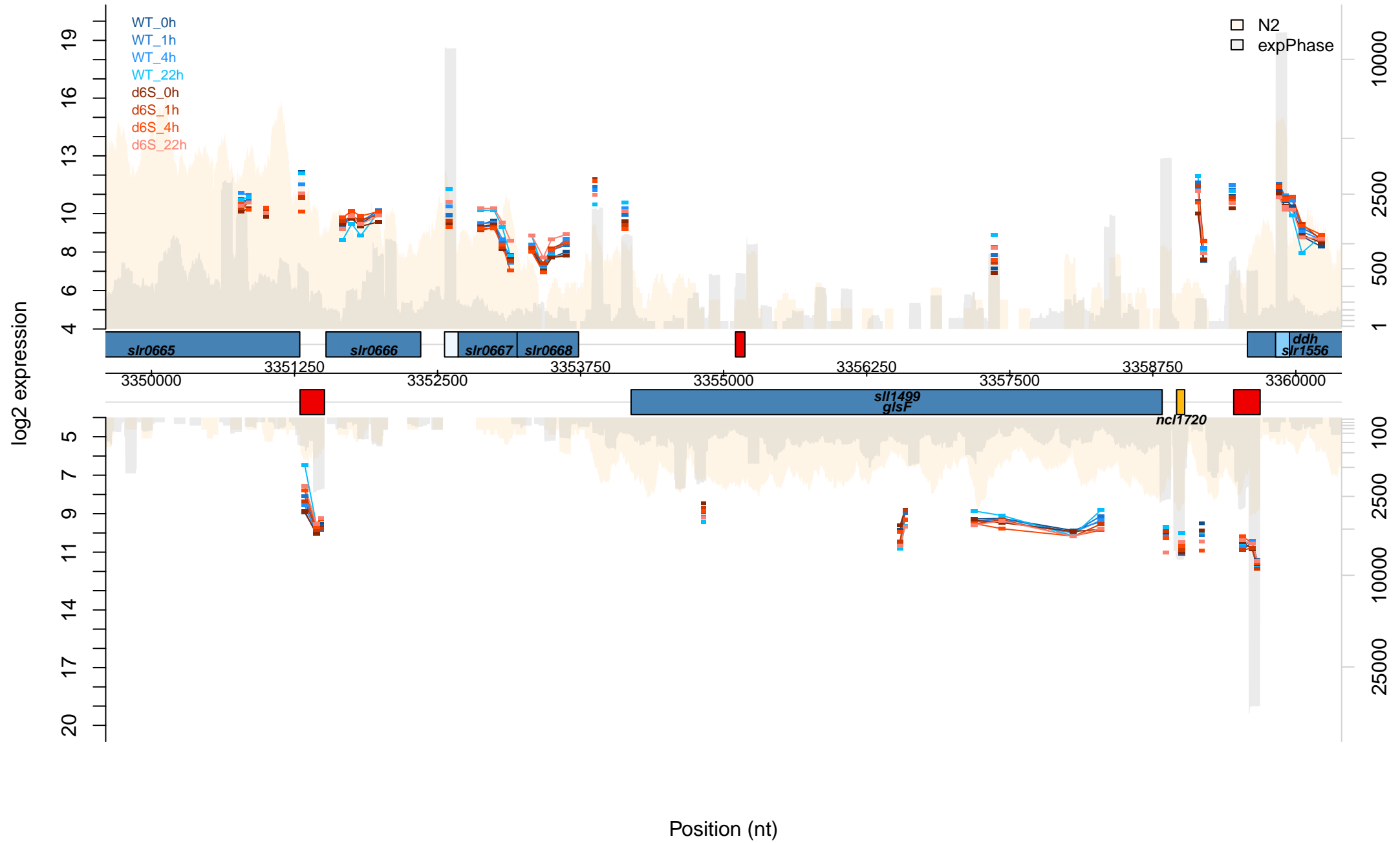

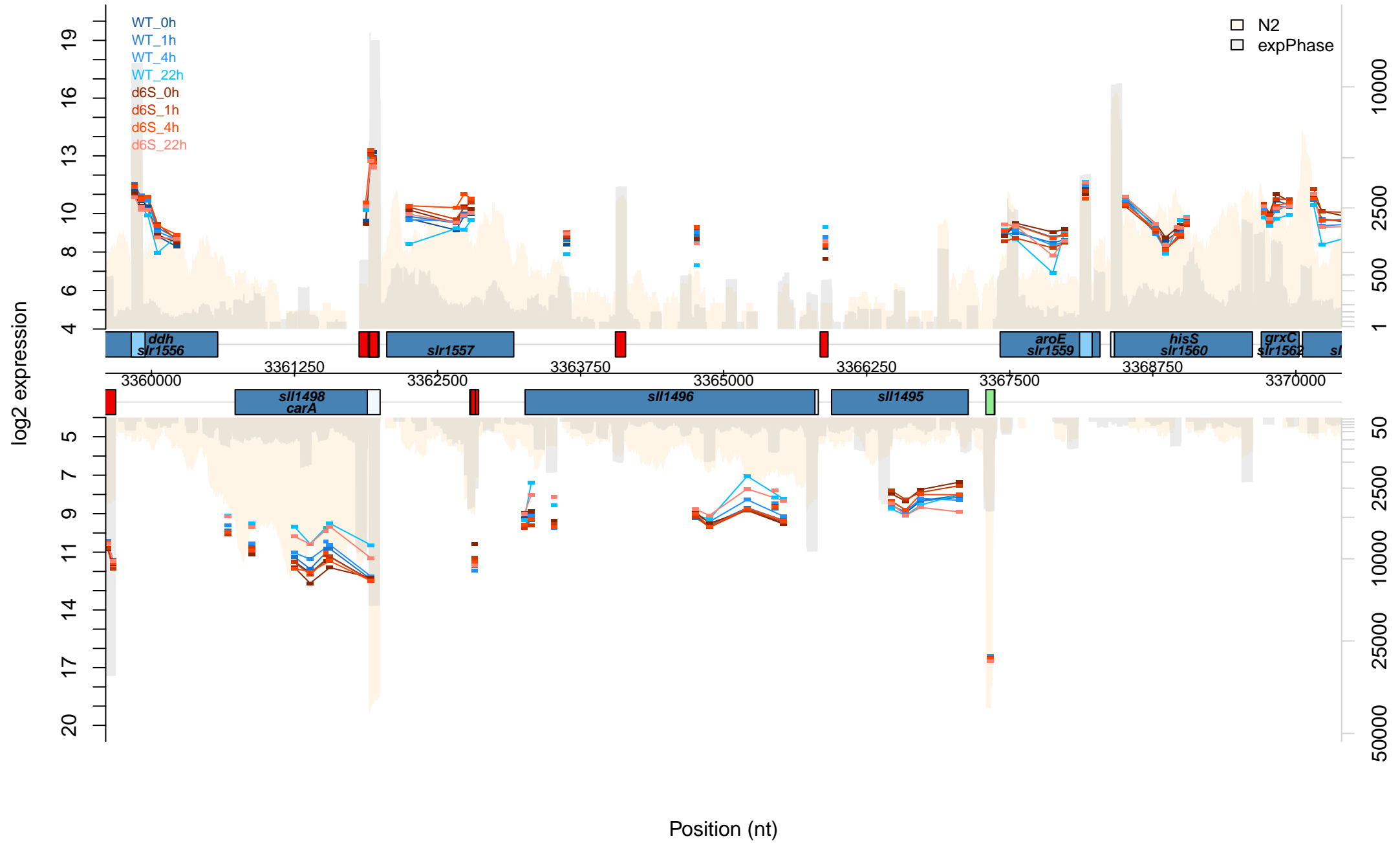

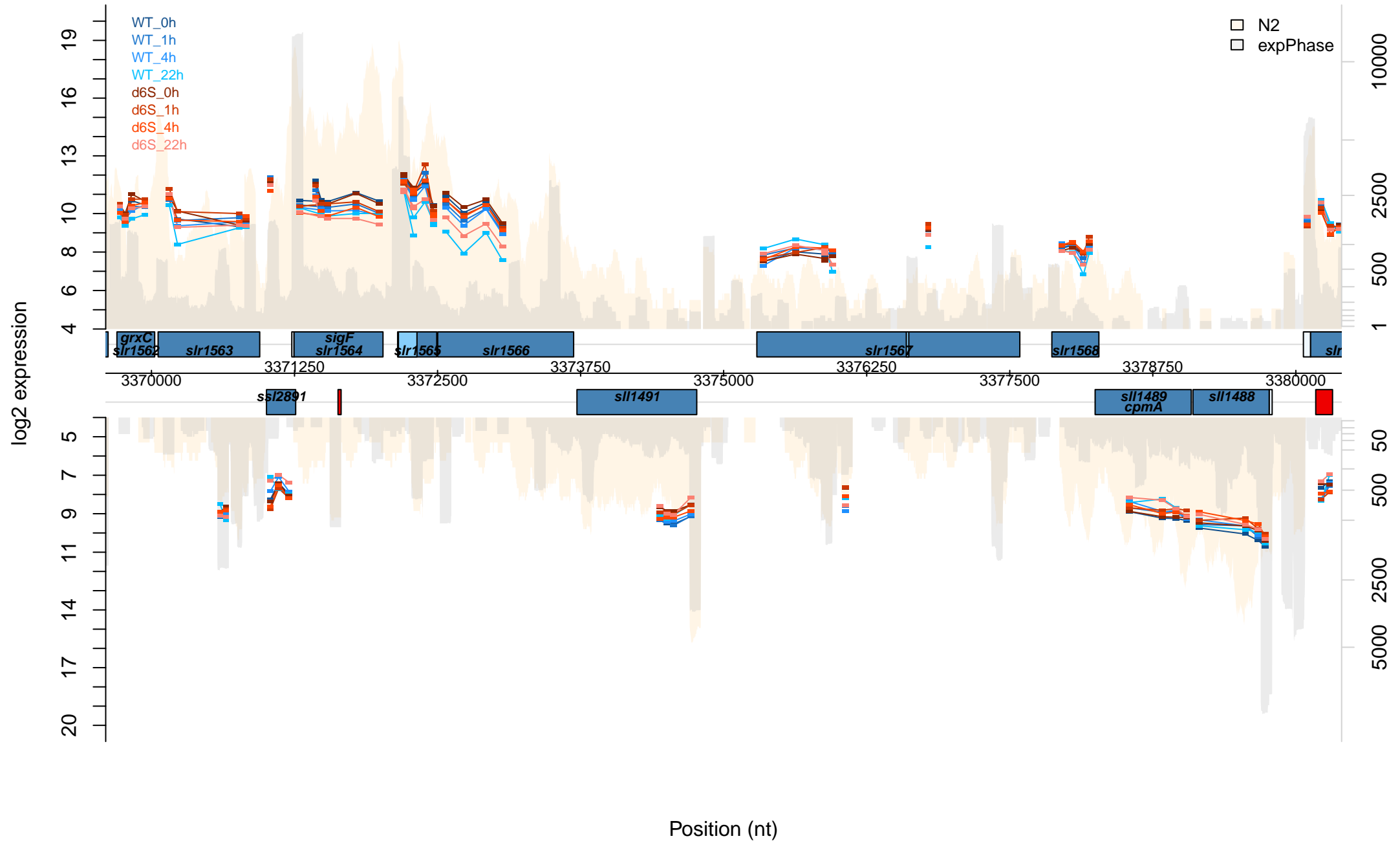

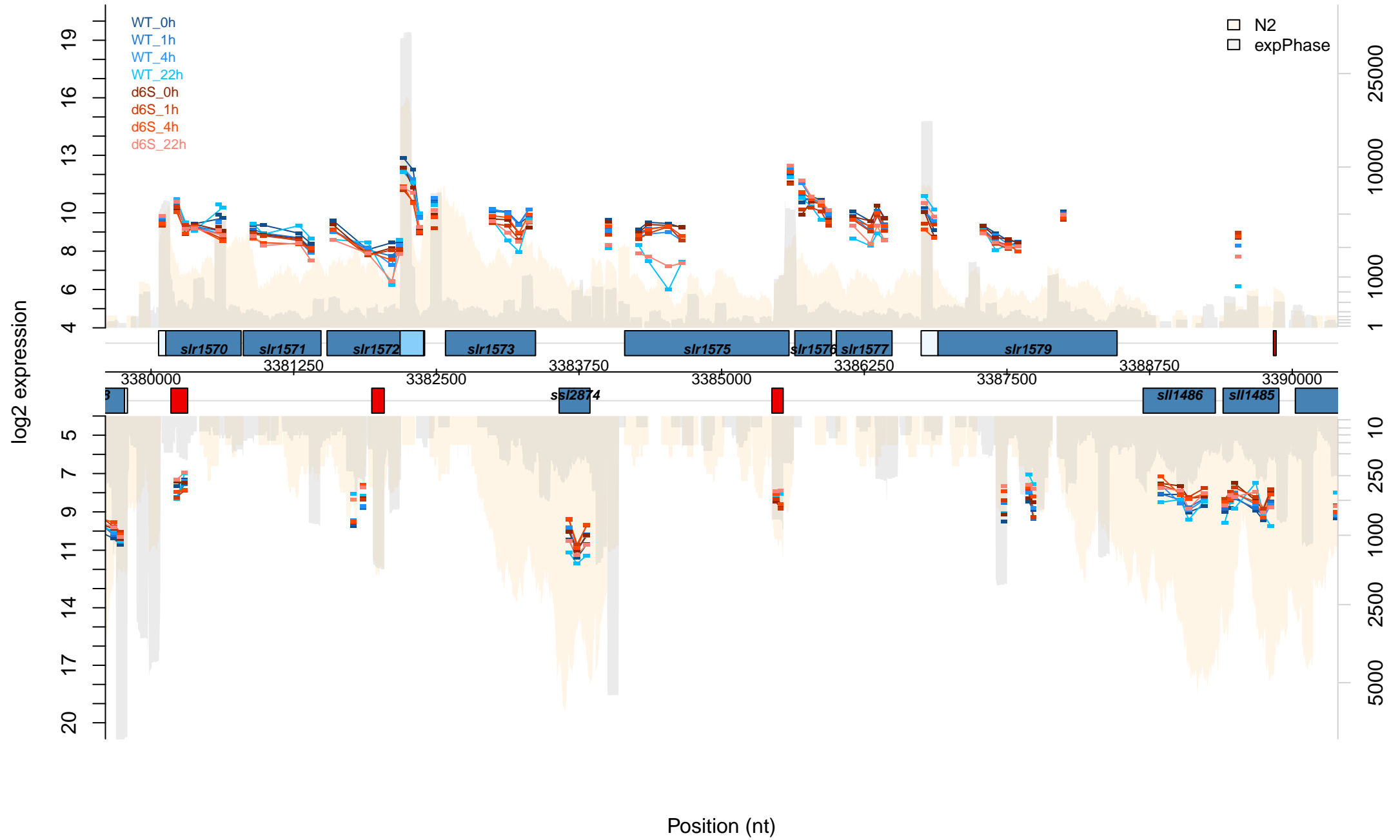

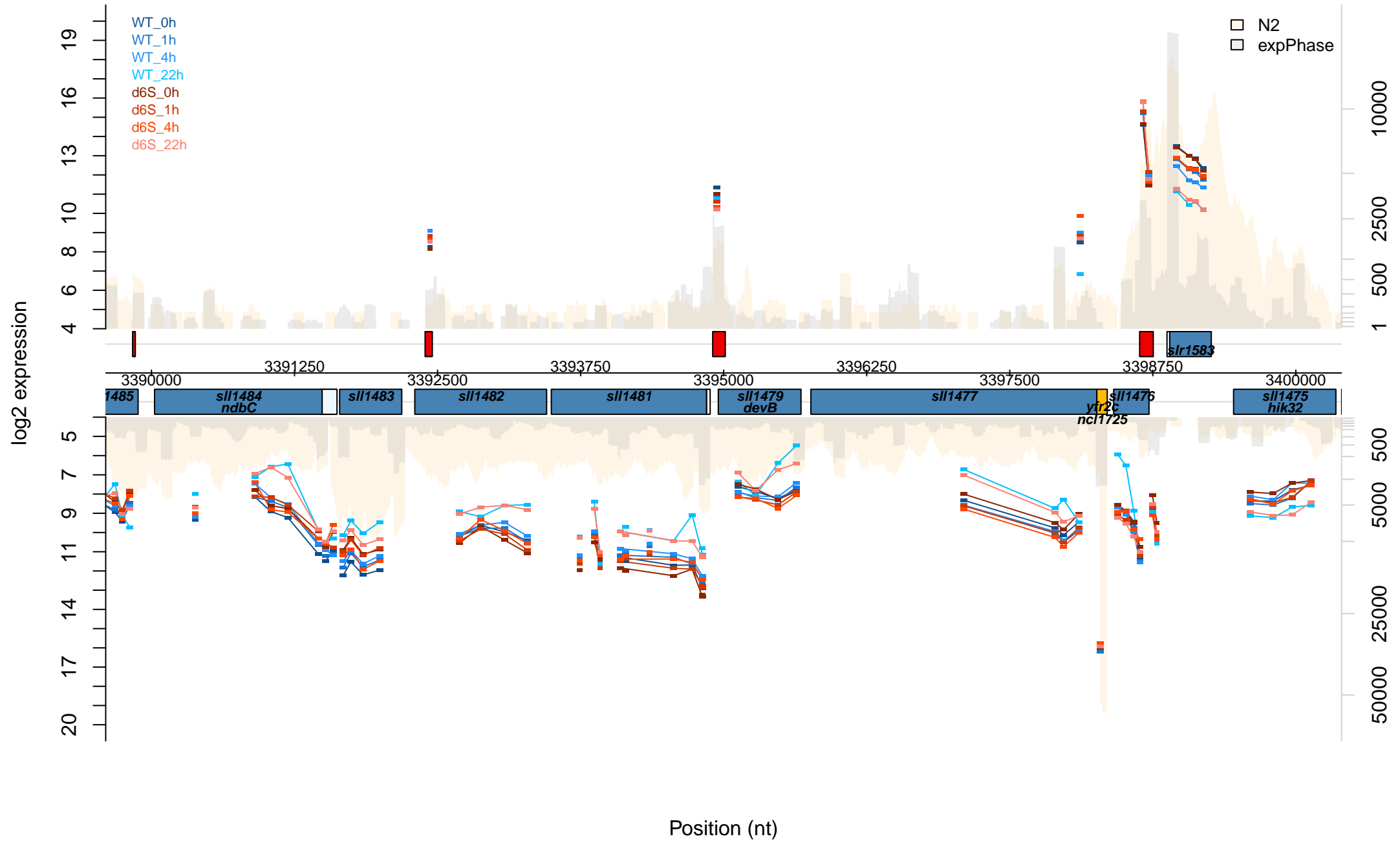

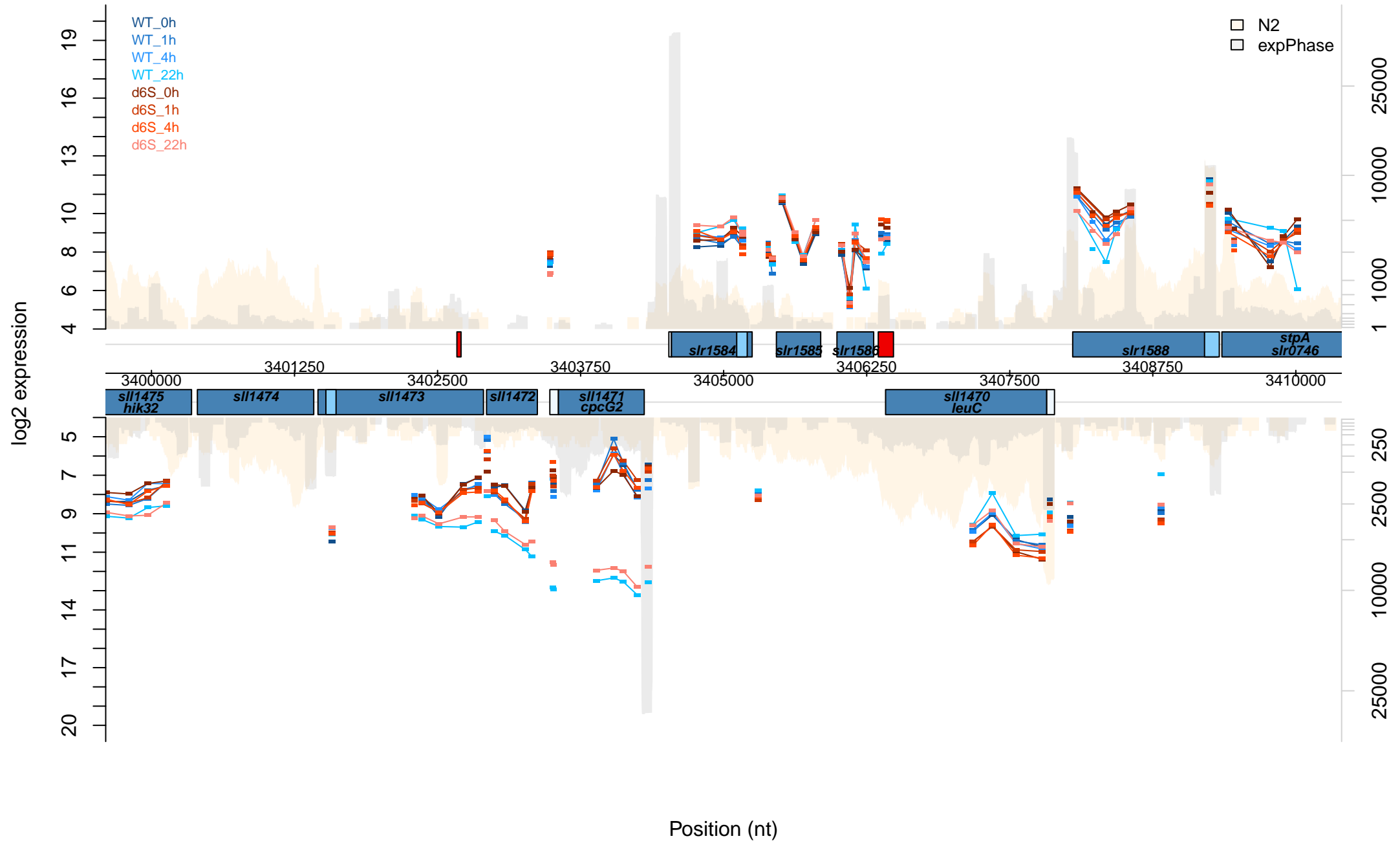

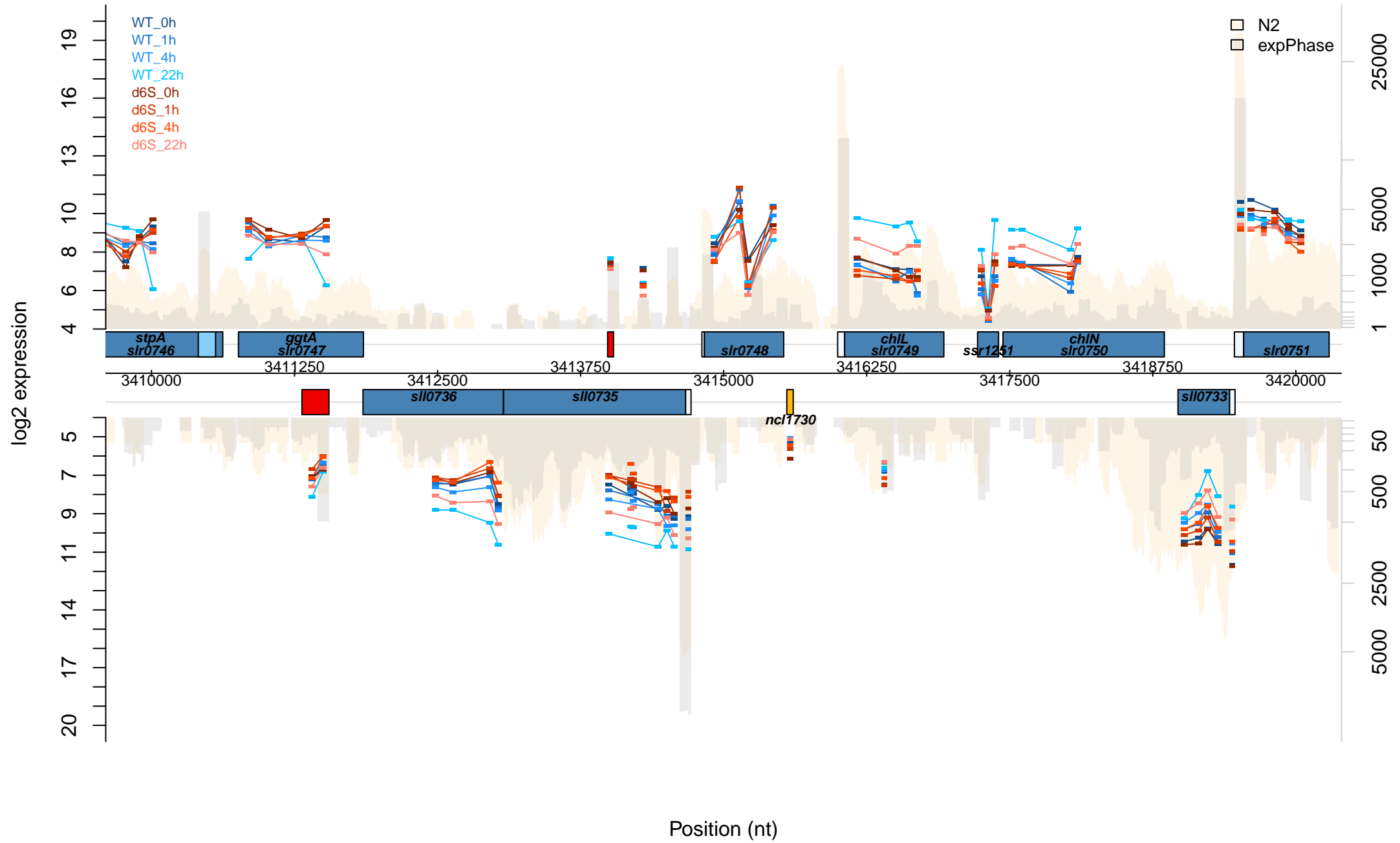

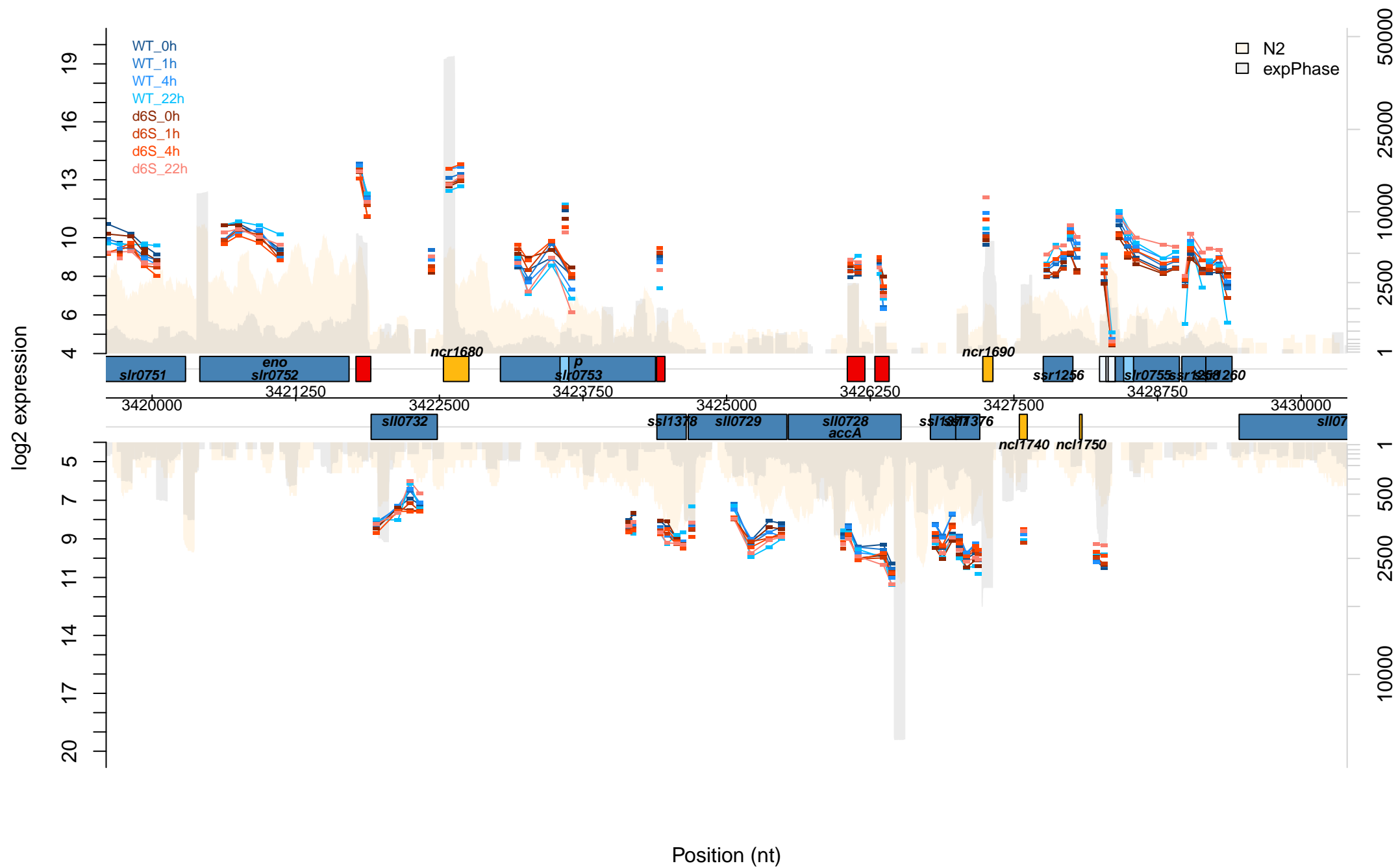

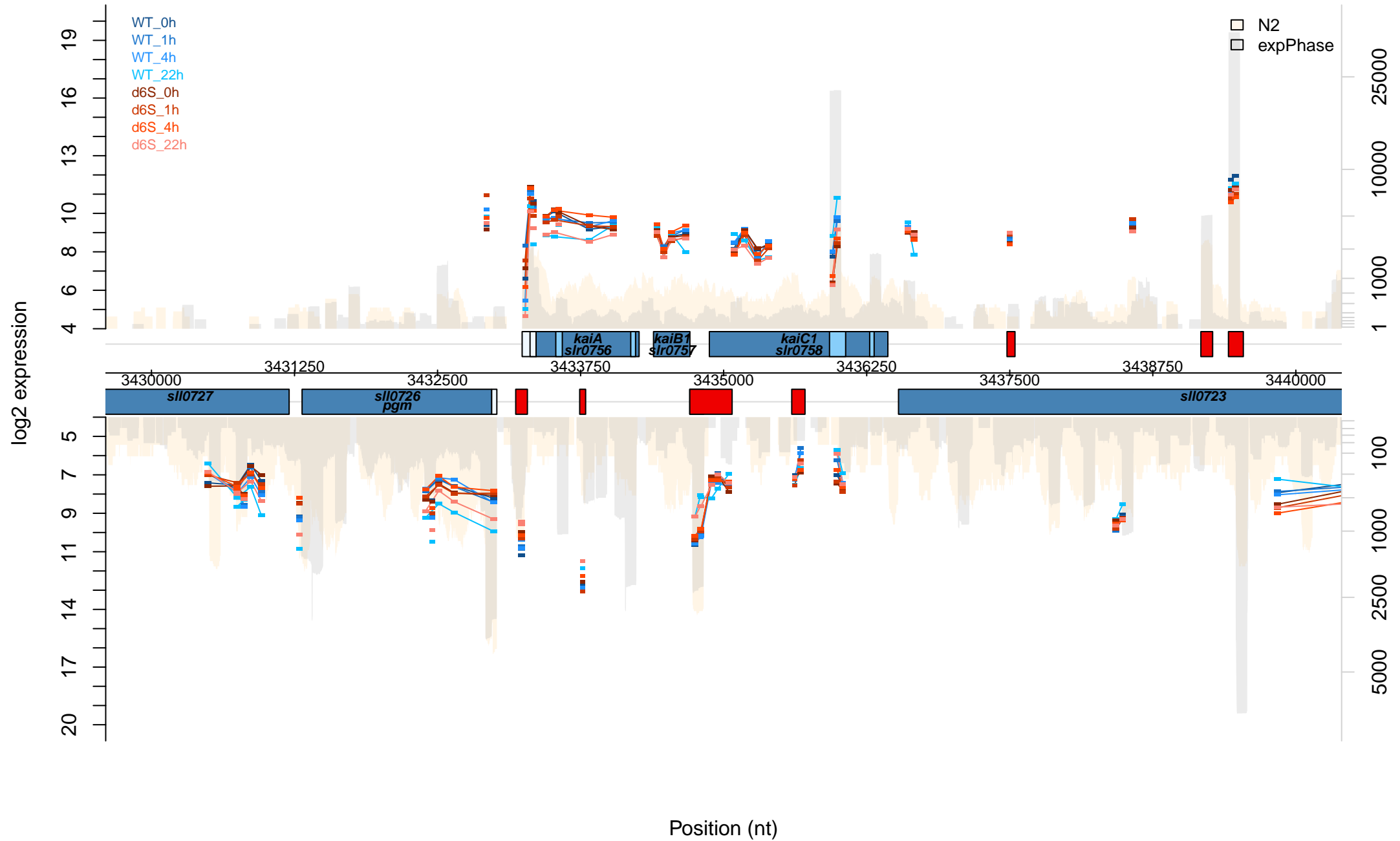

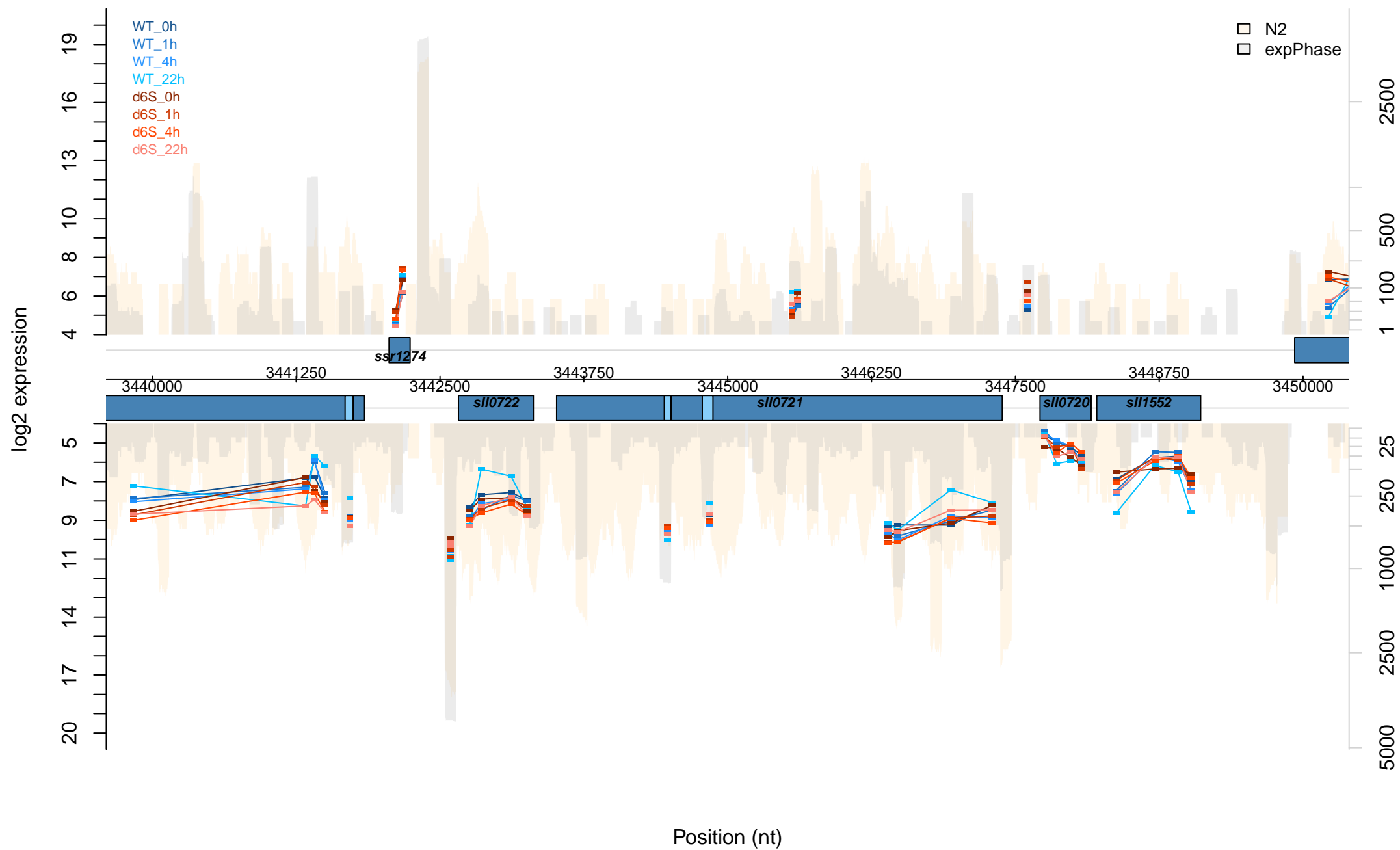

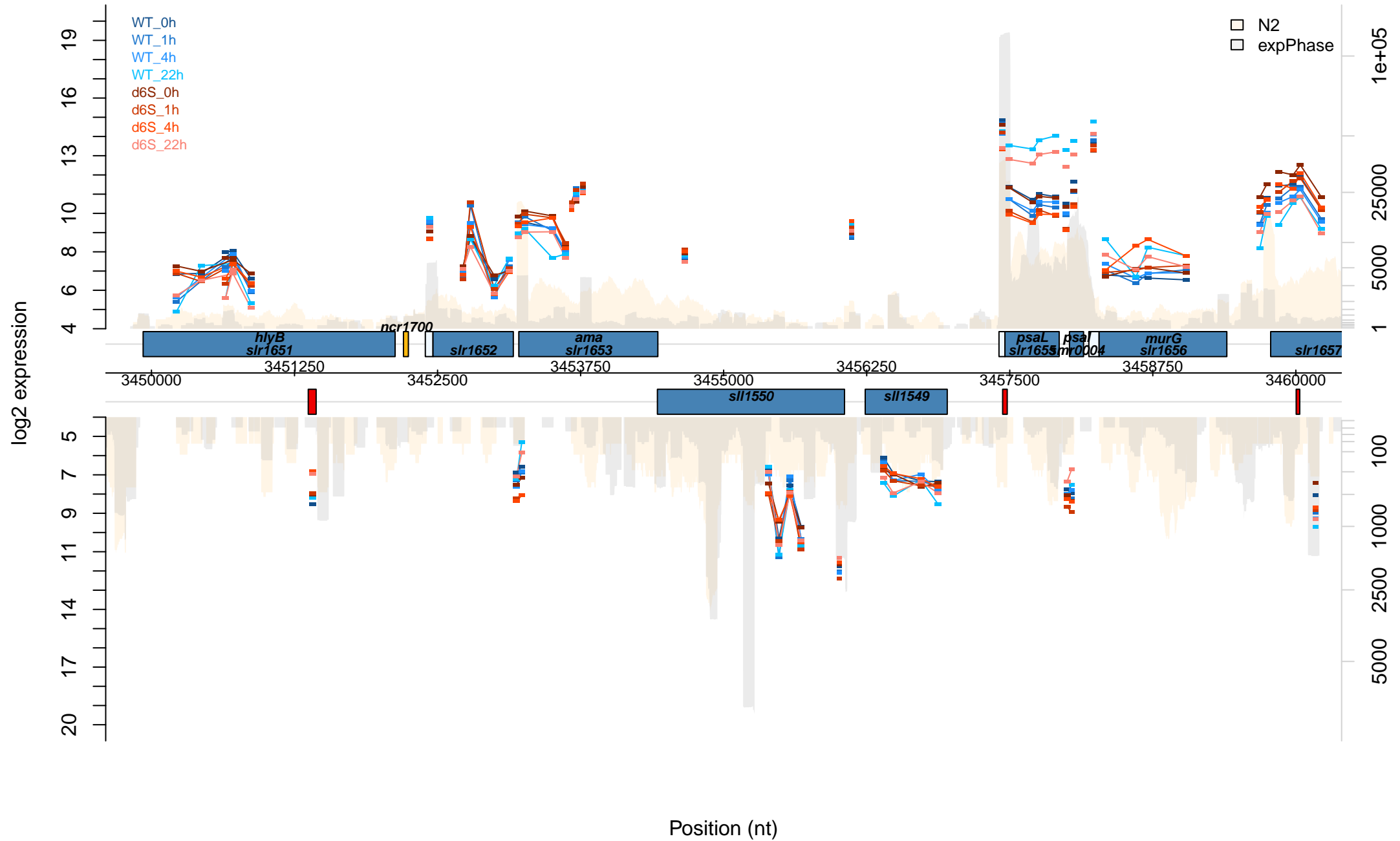

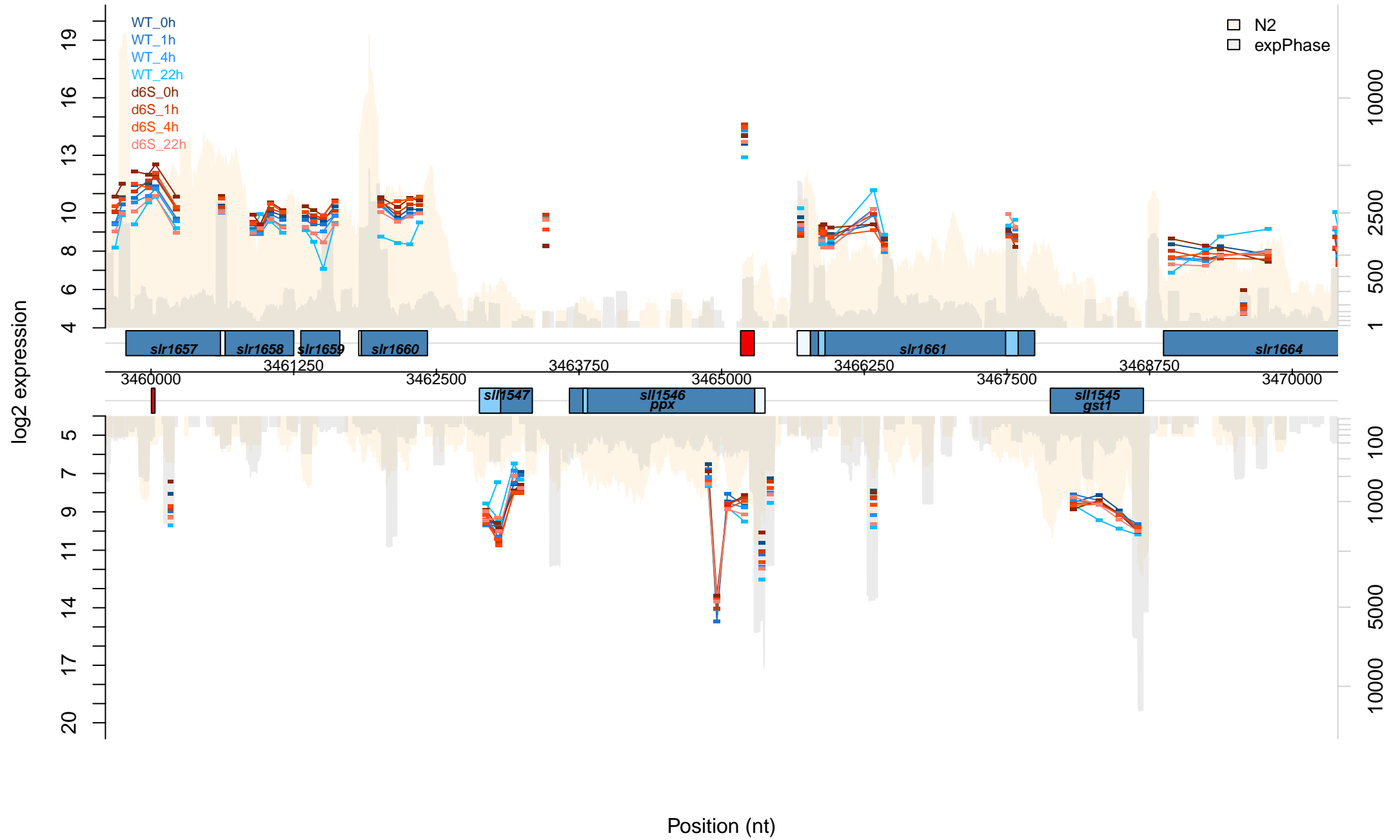

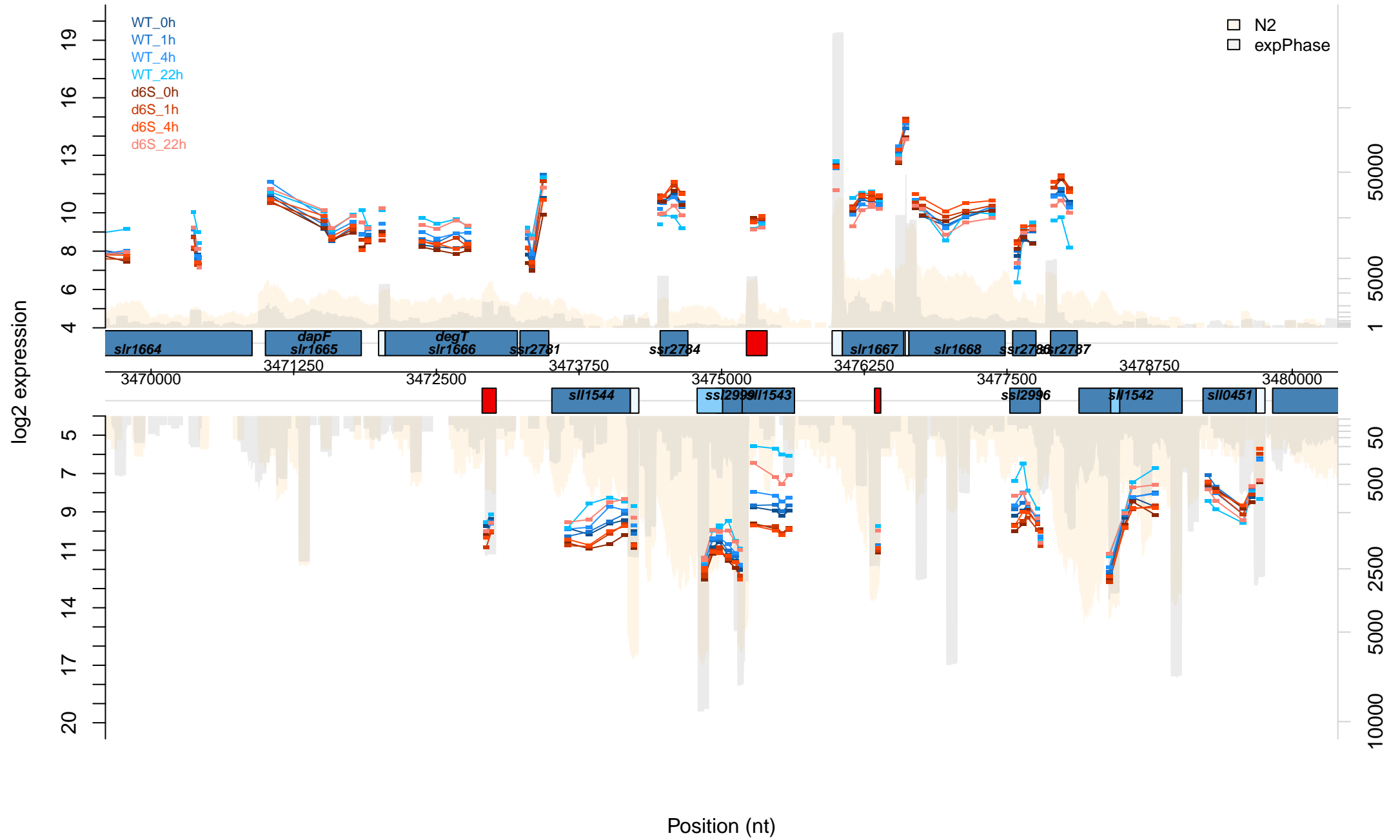

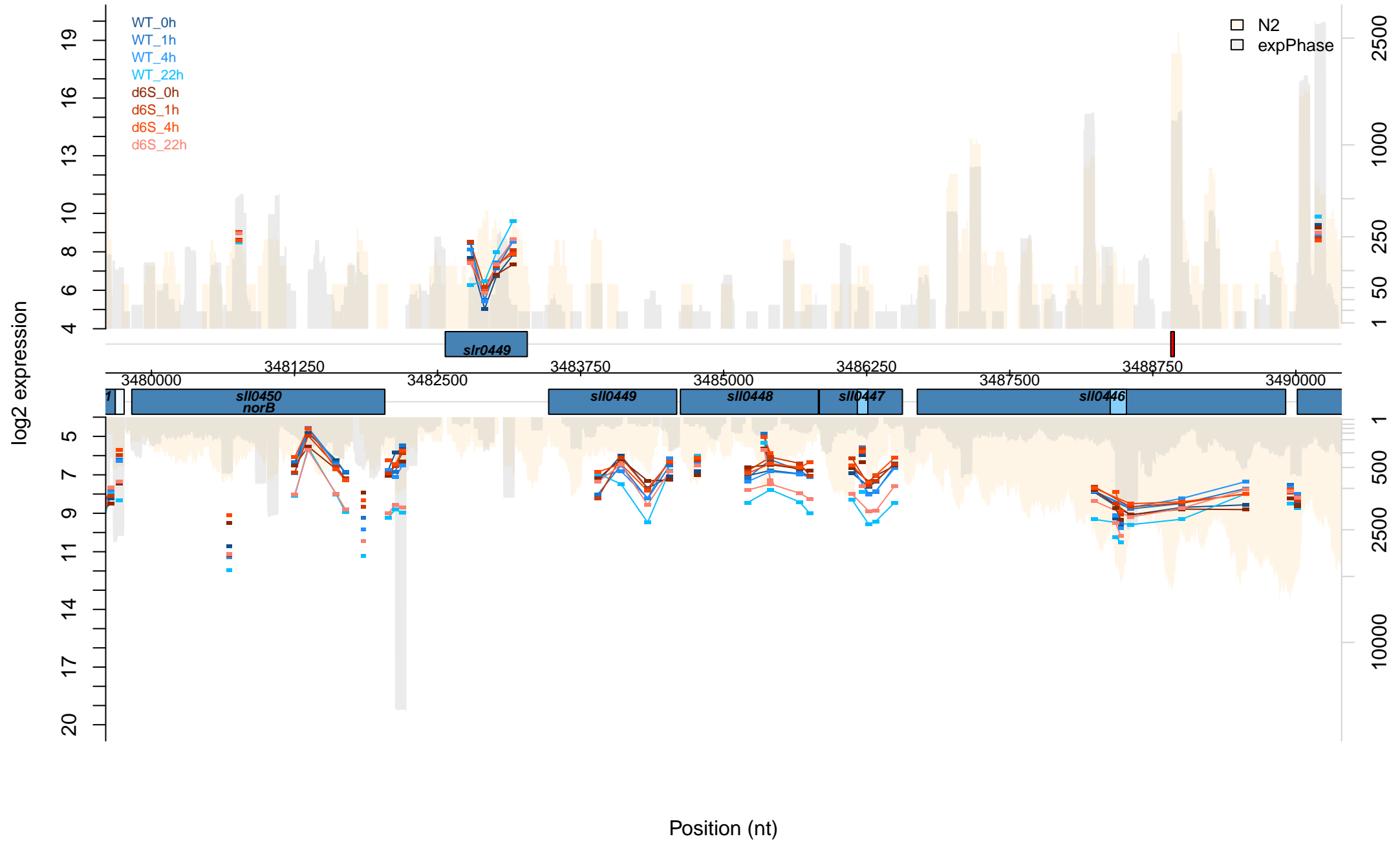

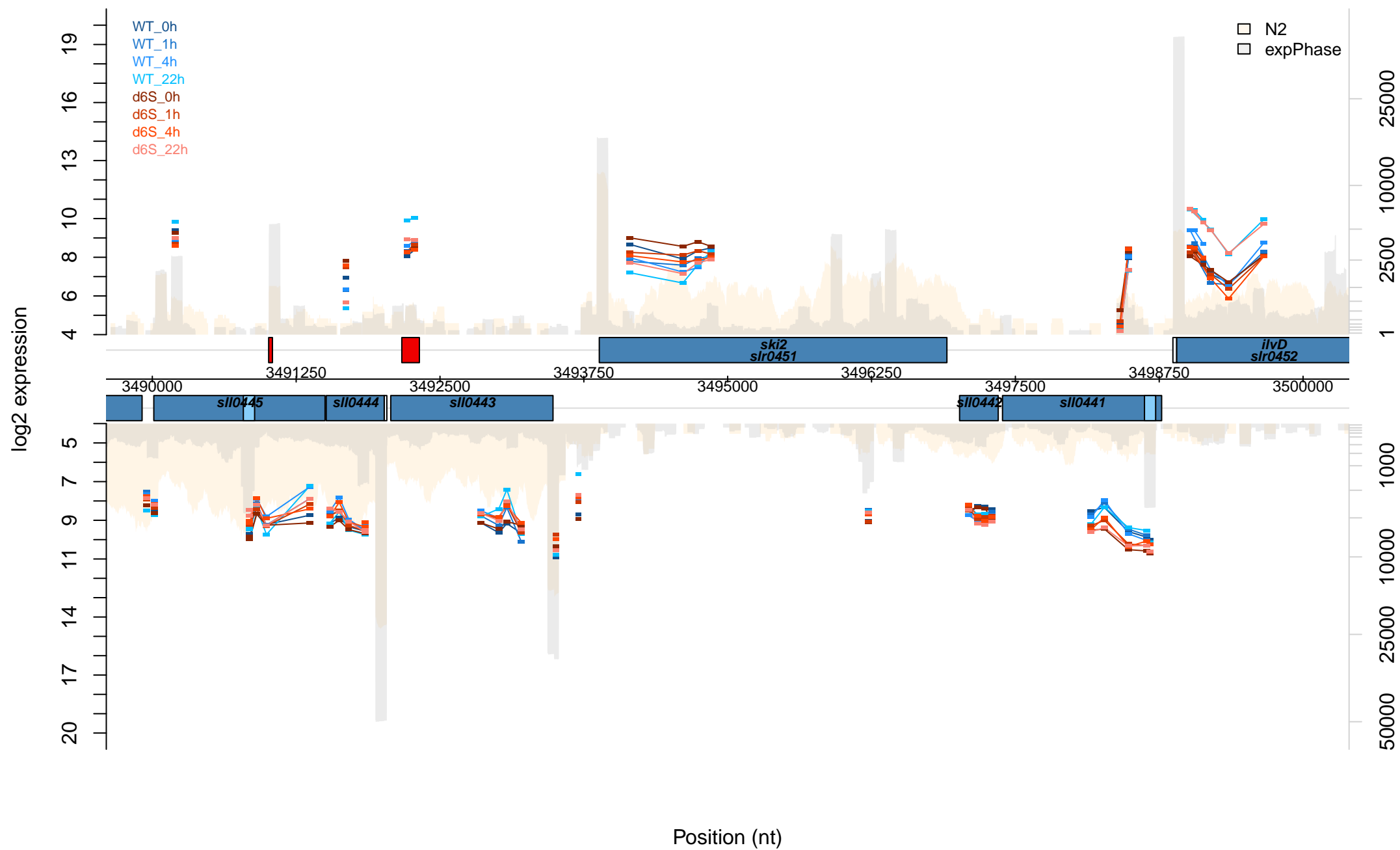

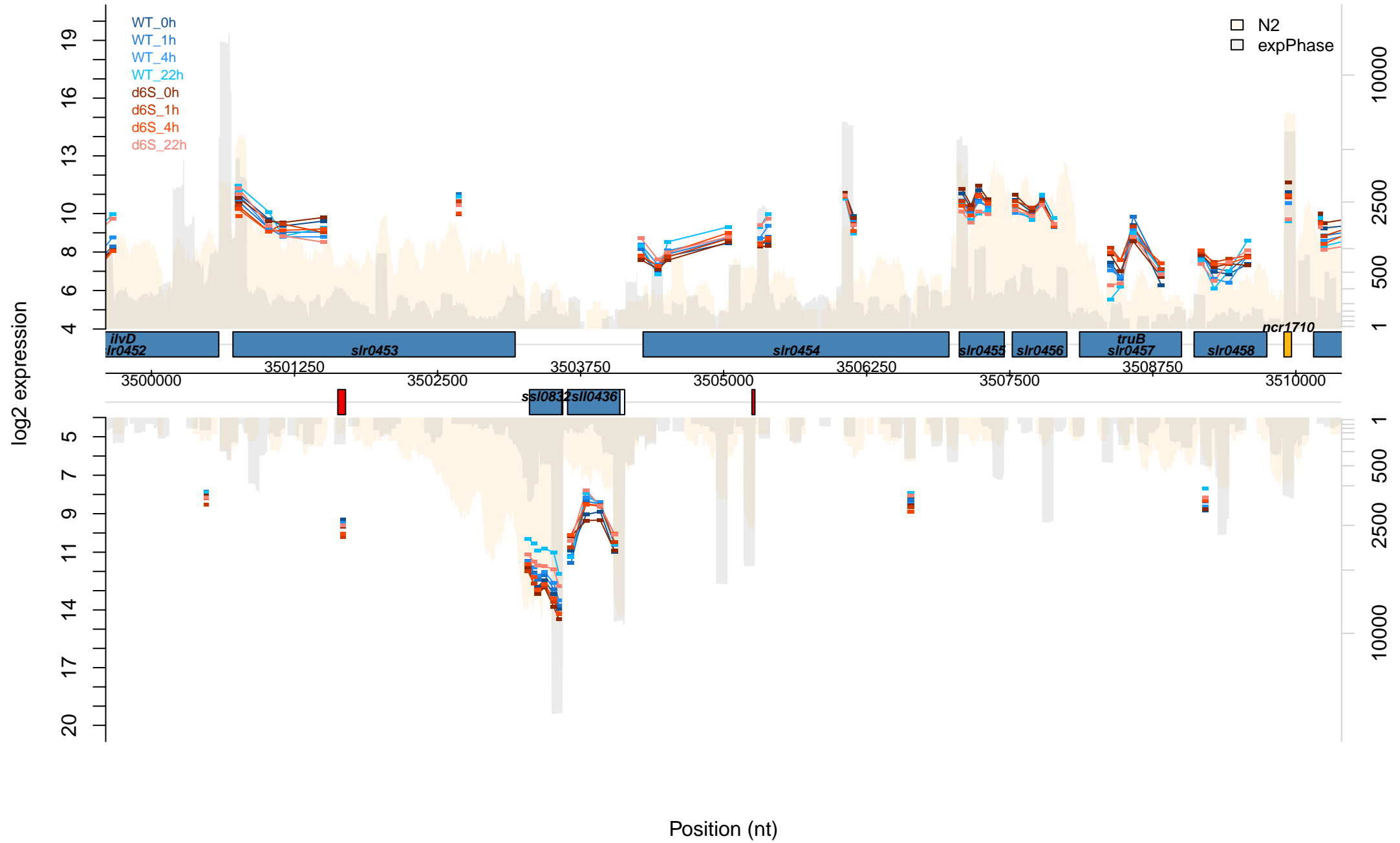

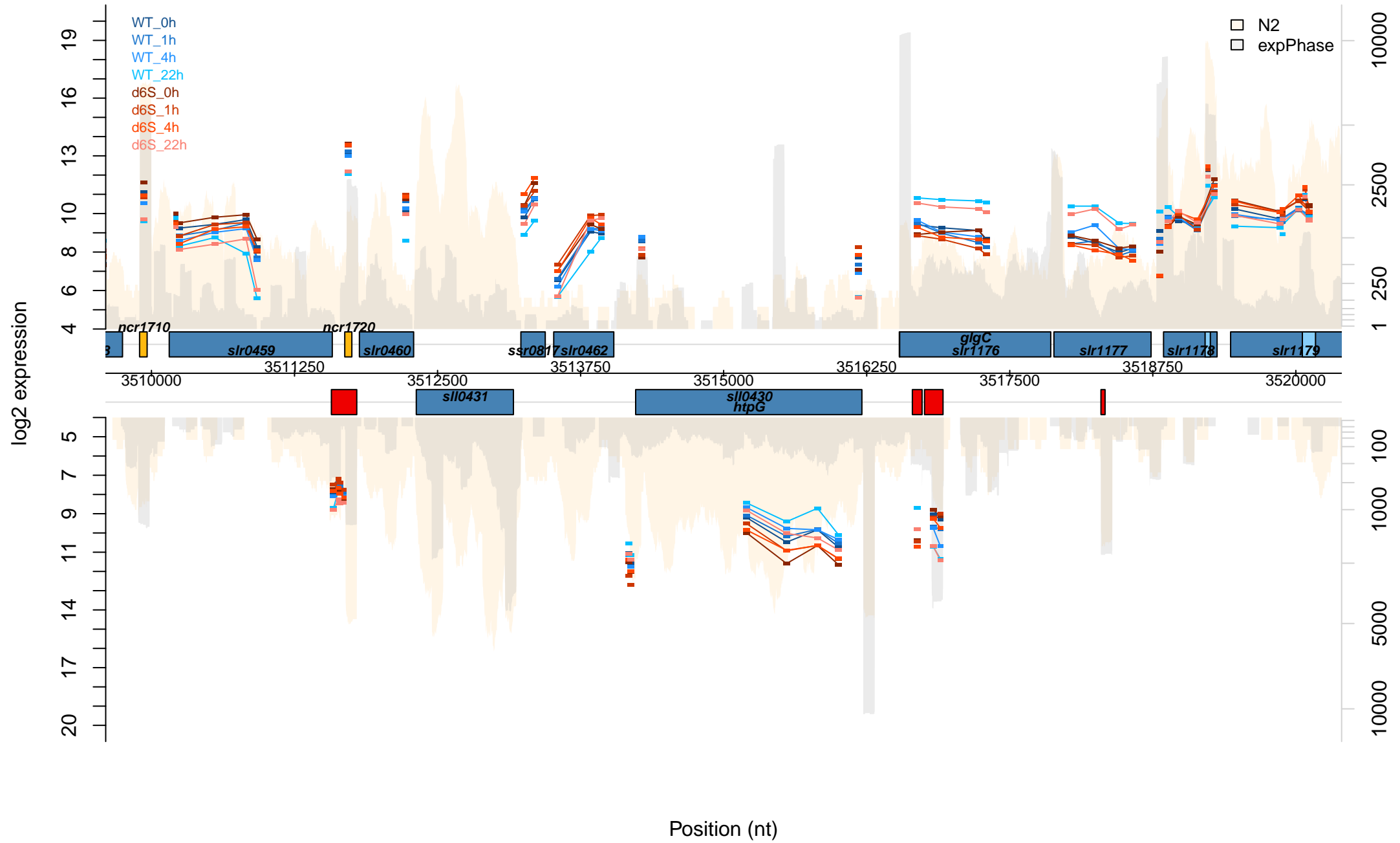

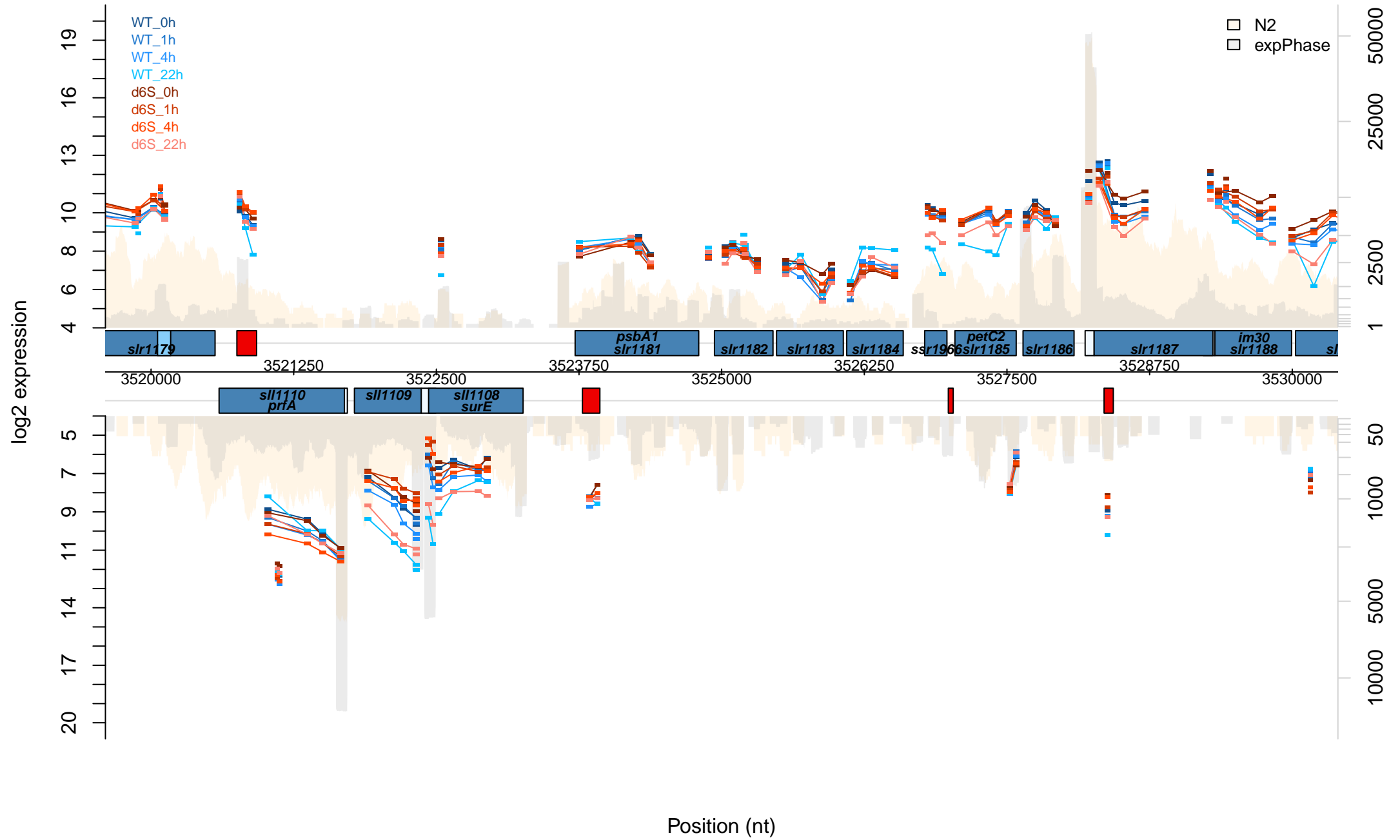

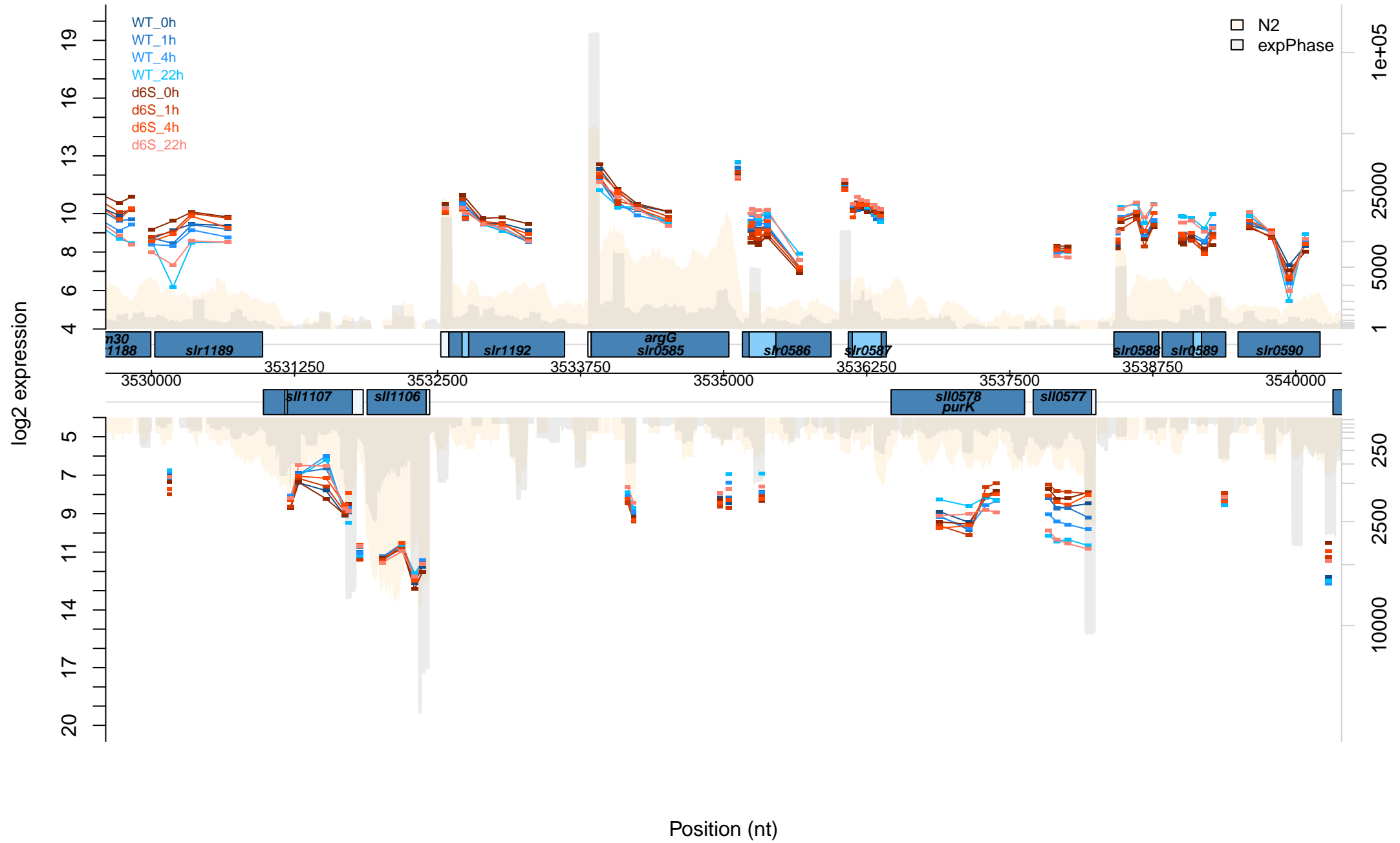

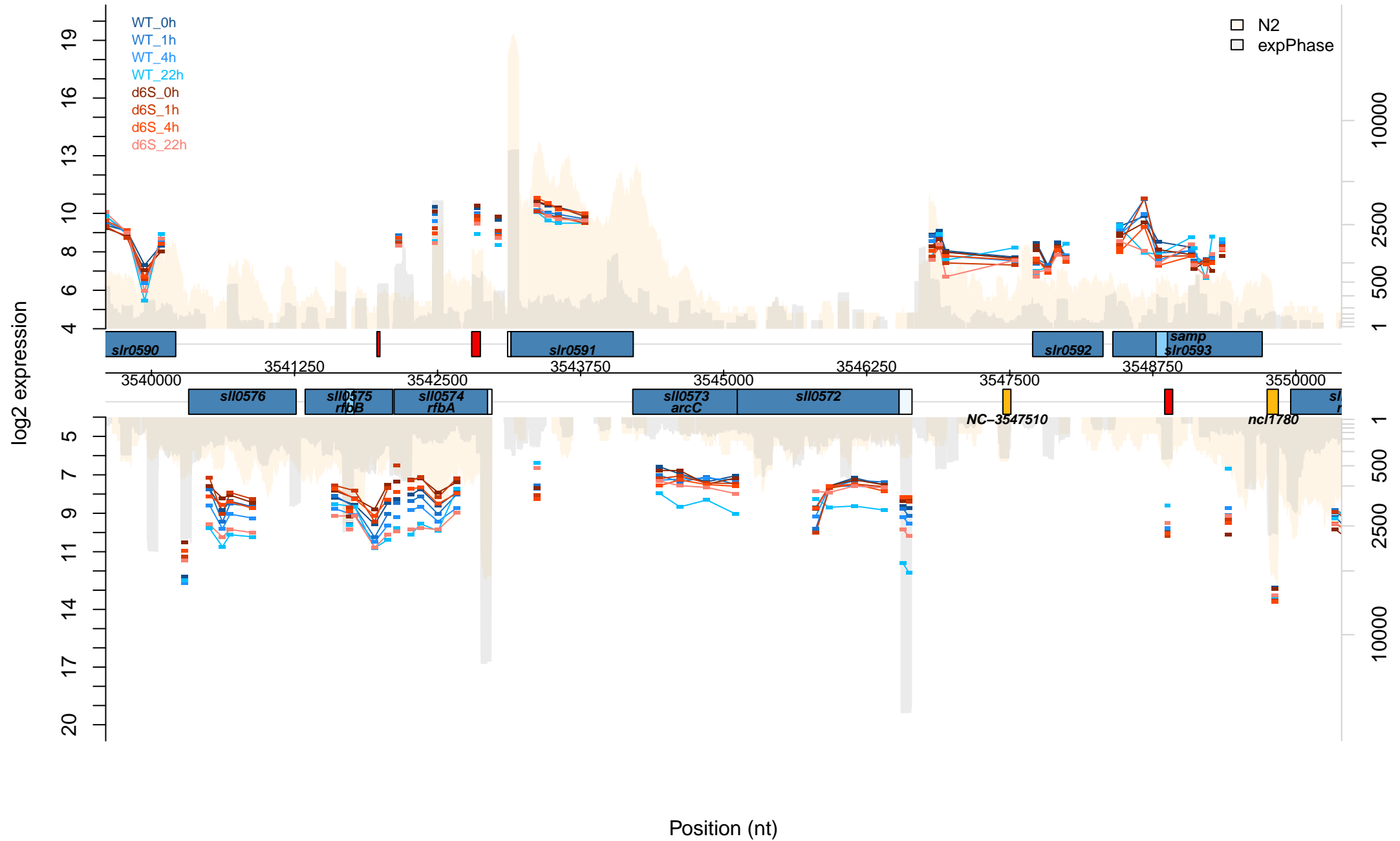

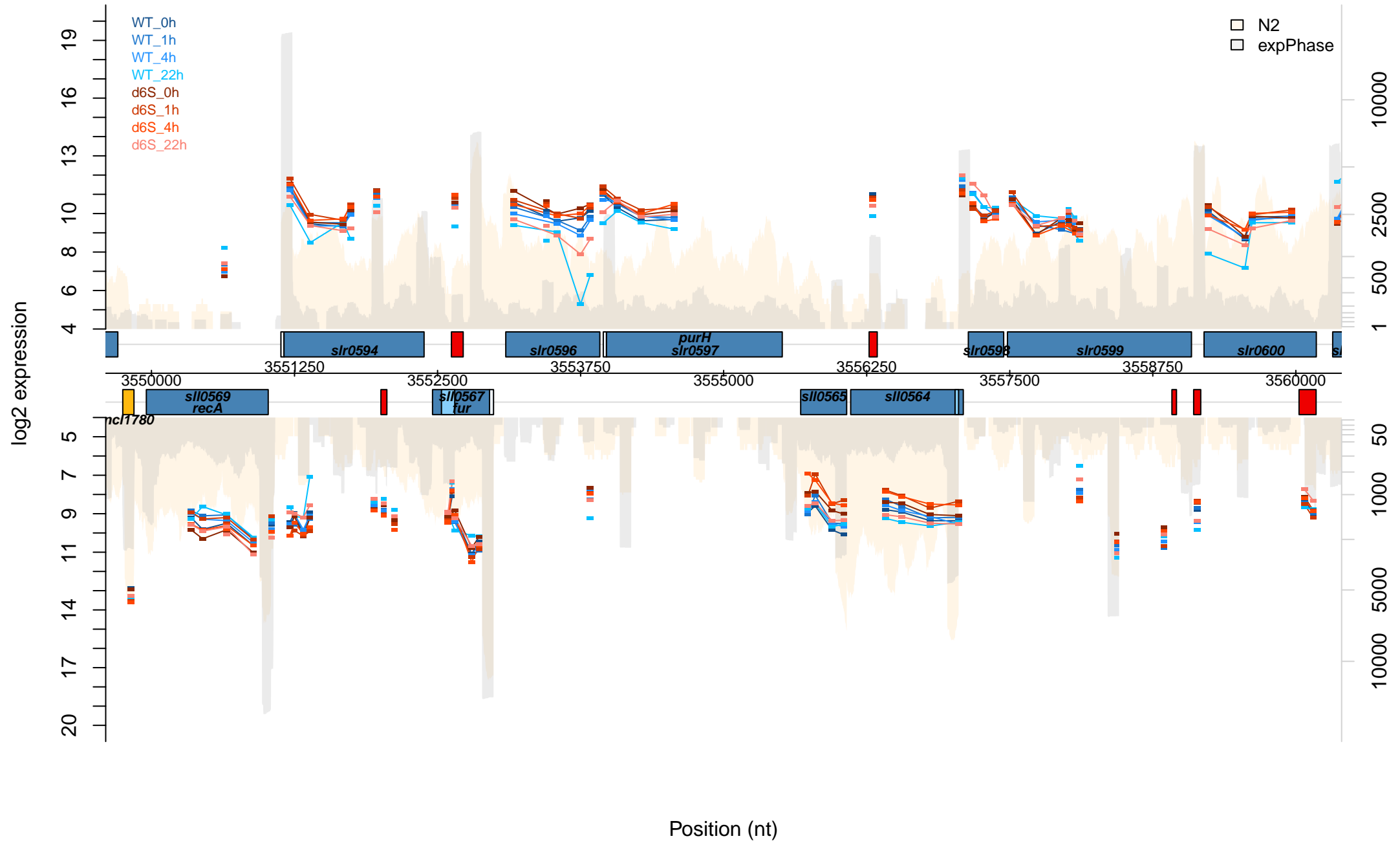

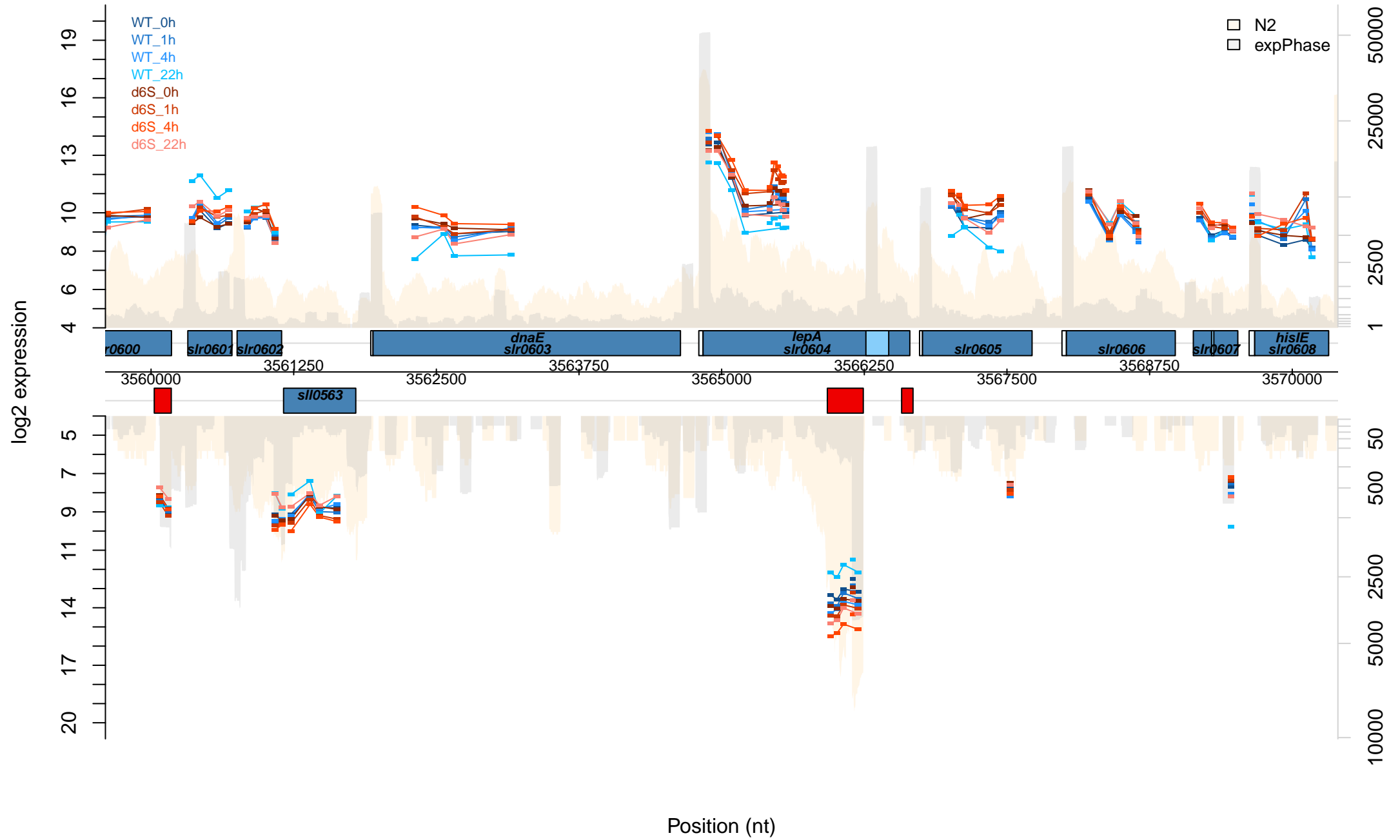

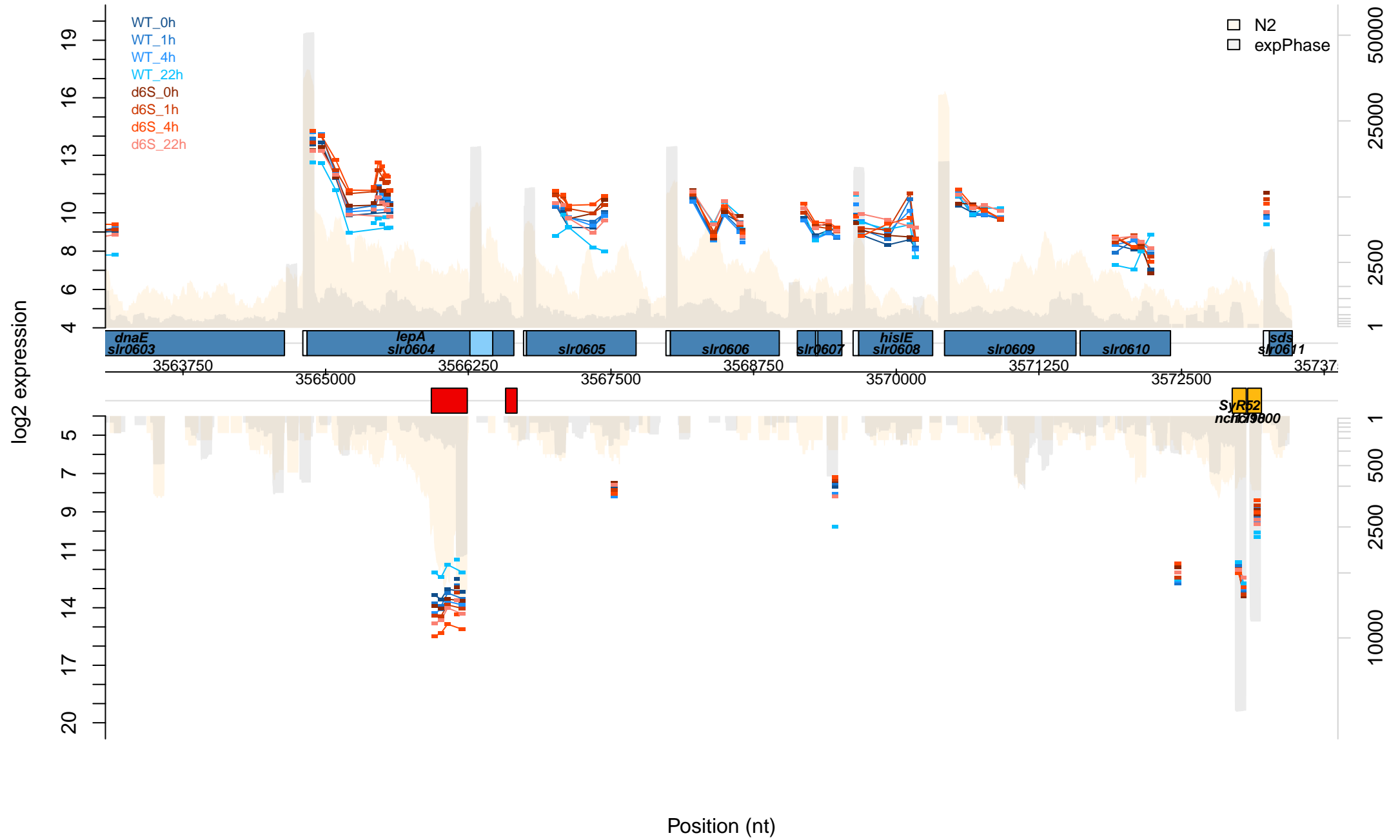

Supplement: Supplementary file 2 — Overview of the genome-wide expression profile of Synechocystis 6803 wild type (WT) and ΔssaA (d6S) strain under nitrogen depleted conditions (t1 = 0 h + N) and at recovery from nitrogen depletion (t2 = 1 h + N, t3 = 4 h + N, t4 = 22 h + N) by combining the log2 expression values (left scale) with read numbers of 454 sequencing (right scale). (PDF 42993 kb) [file 12866_2017_1137_MOESM2_ESM.pdf]
